# Revision of torrent mites (Parasitengona, Torrenticolidae, *Torrenticola*) of the United States and Canada: 90 descriptions, molecular phylogenetics, and a key to species

**DOI:** 10.3897/zookeys.701.13261

**Published:** 2017-09-21

**Authors:** J. Ray Fisher, Danielle M. Fisher, Michael J. Skvarla, Whitney A. Nelson, Ashley P.G. Dowling

**Affiliations:** 1 Department of Entomology, University of Arkansas, Fayetteville, AR 72701, USA; 2 Systematic Entomology Laboratory, Agricultural Research Service, U.S. Department of Agriculture, Beltsville, MD 20705, USA

**Keywords:** Acari, Acariformes, Hydrachnidiae, water mites, integrative taxonomy, turbo-taxonomy

## Abstract

The descriptive biology of torrent mites (Parasitengona: Torrenticolidae: *Torrenticola*) of North America (north of Mexico) is investigated using integrative methods. Material examined includes approximately 2,300 specimens from nearly 500 localities across the United States and Canada, and a few collections in Mexico and Central America. Species hypotheses are derived from a phylogenetic analysis of the barcoding region of cytochrome *c* oxidase subunit 1 (COI) for 476 specimens and supported with morphology and biogeography. Relationships between species are examined with a combined analysis of COI and two expansion regions (D2–3) of the large ribosomal subunit (28S rDNA) for 57 specimens. All previously described species from the US and Canada are examined. Our results indicate the need to synonymize four species: *T. mercedensis* (Marshall, 1943) is a junior synonym of *T. sierrensis* (Marshall, 1943); *T. rectiforma* Habeeb, 1974 is a junior synonym of *T. ellipsoidalis* (Marshall, 1943); *T. neoconnexa* Habeeb, 1957 is a junior synonym of *T. magnexa* Habeeb, 1955; and *T. esbelta* Cramer, 1992 is a junior synonym of *T. boettgeri* KO Viets, 1977. We describe 66 new species and re-describe all previously described regional species. Our findings indicate that total diversity of *Torrenticola* in the United States and Canada comprises 90 species, 57 known from the east and 33 from the west. We organize these species into four species complexes that include 13 identification groups. An additional 13 species do not fit within an identification group. The southern Appalachians are suspected to contain the highest concentration of remaining undescribed diversity. A key is provided to all known species in the US and Canada.

## Introduction

**Figure 1.**
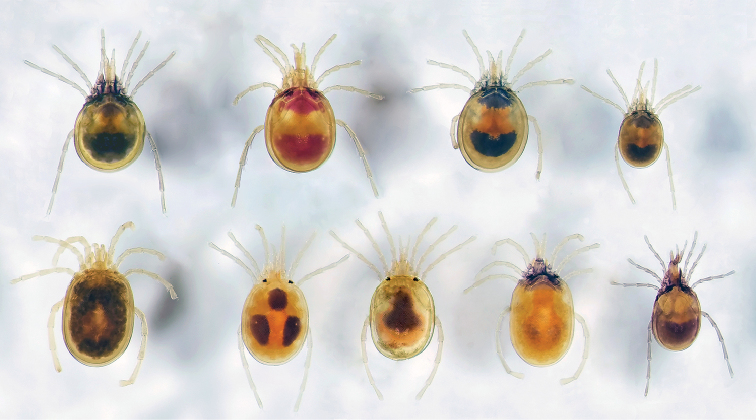
Diversity of *Torrenticola* in Ouachita National Forest, Arkansas. Species from top-left to bottom-right: *T. irapalpa* sp. n., *T. interiorensis* sp. n., *T. biscutella* sp. n. female, *T. biscutella* sp. n. male, *T. pearsoni* sp. n., *T. trimaculata* Fisher & Dowling 2016, *T. unimaculata* sp. n., *T. larvata* Cherri et al. 2016, *T. solisorta* sp. n. Note that, for a given locality, many characters necessary for identification can be seen under low magnification. For example, species in the bottom row can be readily differentiated using color pattern. In contrast, species in the top row have similar color patterns; however, even they can be readily identified: *T. irapalpa* have dorsal glandularia (Dgl-4) closer together than the other species and *T. biscutella* have fused anterio-lateral platelets and strong sexual dimorphism (neither character is present in *T. interiorensis*). Only the identification of *T. interiorensis* is tentative based on this image, because *T. neoanomala* (not pictured), which are also found in this locality, can only be differentiated from *T. interiorensis* by comparing precise measurements of the anterio-lateral platelets, necessitating examining slide-prepared material under higher magnification. Also, note that some species can appear differently than what is depicted in species descriptions. For example, although *T. pearsoni* sp. n. has a nearly colorless cuticle, it can appear dark due to gut contents, as shown here. Image is a composite of stacked images from a Samsung GS7 and edited in Adobe Photoshop.

Torrenticolidae Piersig, 1902 are known as “torrent mites” due to the typical habitat of most species—fast-flowing, rocky or sandy-bottom streams (Goldschmidt 2007, Smith et al. 2010, Proctor et al. 2015). In fact, in many streams, torrenticolids are among the most abundant of all arthropods. Adults are active predators that crawl through sandy sediment with stout legs and sclerotized bodies and roam interstitial zones in search of micro-crustaceans (Goldschmidt 2007, Smith et al. 2010, Proctor et al. 2015). The dorsal cuticle of adults is often ornamented with colorful patterns that can be useful in differentiating species, especially locally (Figure 1). Larvae are ecto-parasites of chironomids, but cannot yet be identified to the species-level, so questions such as host specificity and loss of parasitism remain open areas of investigation. This difficulty is compounded by the fact that larval and post-larval stages are difficult to link due to disparate morphologies, although future efforts employing molecular techniques may resolve this issue (e.g., Stålstedt et al. 2016).

Torrenticolidae comprises two subfamilies (Wiles 1997): Testudacarinae Cook, 1974 and Torrenticolinae Piersig, 1902. Testudacarinae comprises two genera (O’Neill et al. 2016)—*Debsacarus* Habeeb, 1961 (one species) and *Testudacarus* Walter, 1928 (20 species)—and Torrenticolinae comprises five genera: *Monatractides* Viets, 1926 (approximately 100 species); *Neoatractides* Lundblad, 1941 (11 species); *Pseudotorrenticola* Walter, 1906 (eight species); *Stygotorrenticola* Pešić & Gerecke, 2014 (one species); and *Torrenticola* Piersig, 1896 (approximately 250 species, not including species described herein).

*Torrenticola*—the focus of this paper—are found worldwide, excepting Antarctica. In North America, 79 species have been described, most of which are known from Central America due to the efforts of Goldschmidt (2007). North of Mexico, only 23 *Torrenticola* have been described (Marshall 1929, 1930, 1933, 1943; Habeeb 1955, 1957, 1961, 1973, 1974; Crowell 1960; Fisher et al. 2015; and Cherri et al. 2016) and no comprehensive review is available. Most species are known from only one or a few specimens and sampling has been greatly limited.

The present study is the fourth in a series of ongoing taxonomic investigations of North American Torrenticolidae. The first study offered a detailed description of a single species, diagnoses of higher ranks, and a historical review (Fisher et al. 2015); the second investigated the systematics of Testudacarinae (O’Neill etal. 2016); and the third allowed an undergraduate researcher to describe a species endemic to the Ouachita Mountains, USA (Cherri et al. 2016). Herein, we describe 66 new species and re-describe 24 species, totaling 90 species. The ability to efficiently test species boundaries of such a widespread and diverse genus is a feat made possible with molecular data and by the researchers who contributed to the immense holdings of the Canadian National Collection (CNC), especially Ian Smith, who collected fresh material for molecular analyses. These collections were supplemented with local collections in Arkansas and four expeditions (Rocky Mountains, Pacific Northwest, California, and Alaska).

## Methods

**Curation**: Mites were collected and curated using the protocol detailed by Proctor et al. (2015, p. 649–655) and summarized by Fisher et al. (2015). Specimens collected for molecular analyses were preserved in 95% ethanol, whereas others were preserved in GAW (50% glycerol, 10% glacial acetic acid, and 40% water by volume; also referred to as Koenike’s solution). Slide preparations were created after extraction of genomic DNA (see Molecular phylogenetics below) using either glycerin jelly or Hoyer’s medium. Where available, holotypes and allotypes were designated with glycerin jelly slide preparations, rather than Hoyer’s slide preparations. Unlike Hoyer’s, glycerin jelly does not obliterate body coloration, which can be important in identifying many *Torrenticola*. However, Hoyer’s medium has superior optical properties, which enables investigations at higher magnification (i.e., greater than 400×). Therefore, when available, some paratypes were prepared with Hoyer’s medium.

**Figure 2.**
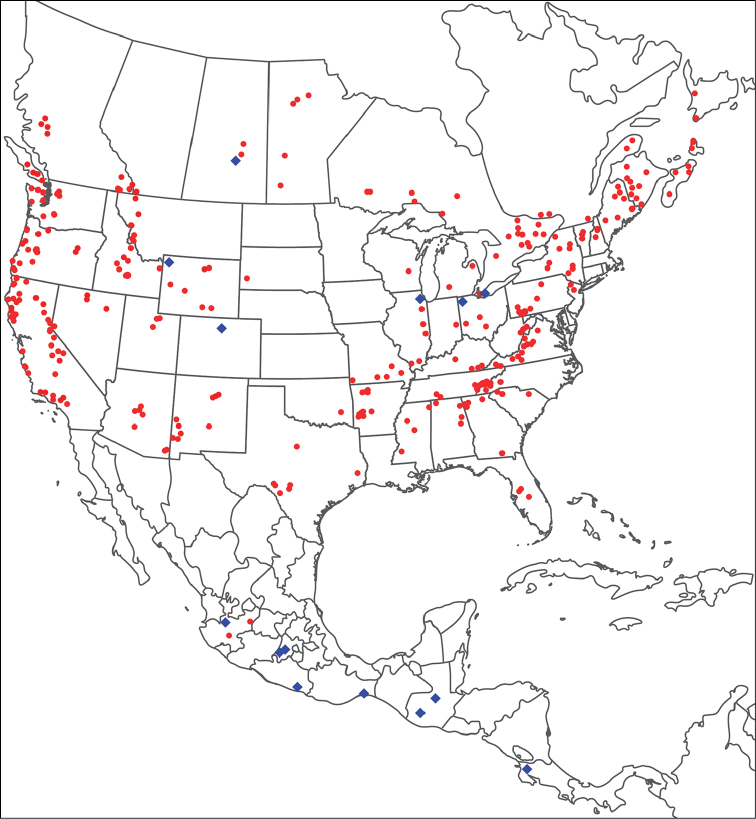
Summary of all sampling localities. 327 red dots represent material examined; 14 blue diamonds represent published accounts where material was not available for direct examination. Each of the 341 localities represent a generalized locality, not an individual collection event, as multiple events often occured in close proximity. In total, this map represents nearly 500 collection events. Note the absence of material from the Great Plains (e.g., Oklahoma, Kansas, Nebraska, and the Dakotas) and Alaska; these are areas that were sampled for water mites, but *Torrenticola* were not found.

**Geographic coverage**: North America, north of Mexico (Figure 2). Importantly, Alaska, which is not represented in Figure 2, was sampled for water mites, but no Torrenticolidae were found. Similarly, the Great Plains region was sampled, but few *Torrenticola* specimens were found. These regions likely represent areas where *Torrenticola*, which appear to prefer cool, clean, mountainous streams, are not present or at least not common.

**Figure 3.**
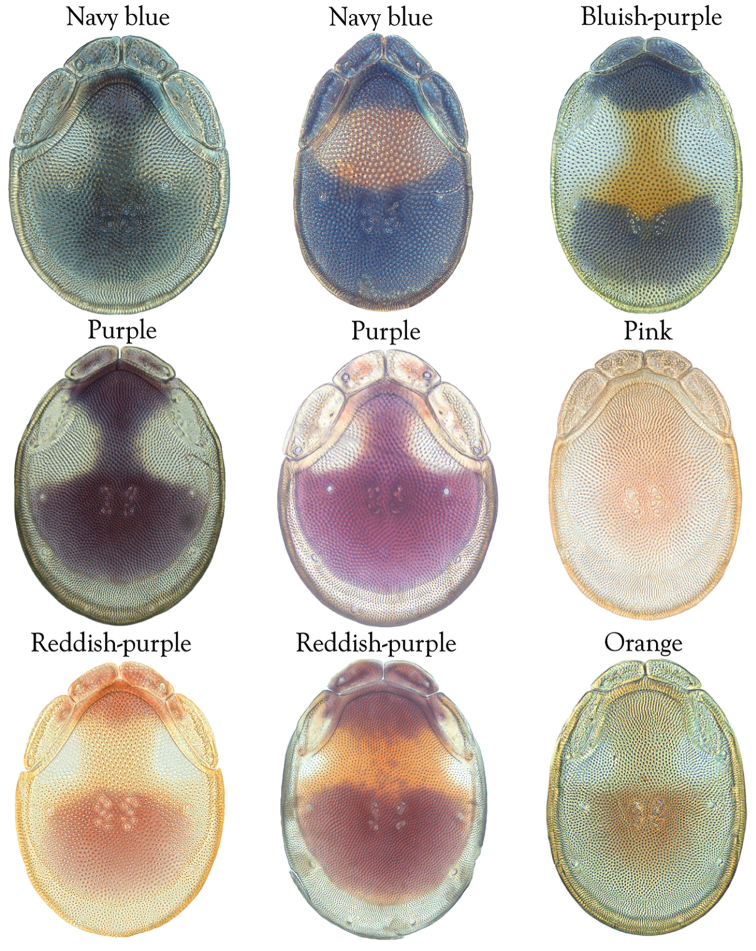
Terminology for coloration: blue – bluish-purple – purple-pink – reddish-purple – orange. Species depicted from top-left to bottom-right: *T. gorti*; *T. erectirostra*; *T. biscutella*; *T. neoanomala*; *T. nigroalba*; *T. gorti*; *T. skvarlai*; *T. magnexa*, and *T. delicatexa*.

**Terminology**: We follow the terminology of Fisher et al. (2015), who modified Goldschmidt (2007) and Proctor et al. (2015), with the addition of a new term, “anterior venter”, used to describe the distance between the gnathosomal bay to the genital field (Figure 6C). Colorful species generally have dorsal shades that ranges from bluish to orangish, and a medial stripe that ranges from orangish to reddish. We use the following terminology regarding the darker shade: navy blue – bluish-purple – purple – reddish-purple – orange (Figure 3).

**Images**: Color micrographs of slide preparations were taken with a Leica DFC 300× camera using Leica Application Suite software and montaged with Helicon Focus 6 to ensure certain structures (e.g., glandularia) were visible. Resulting image stacks were then edited in Adobe Photoshop CS6 to remove debris, repair damaged specimens, and remove structures that are unnecessary for identification. For the latter, legs were removed from the venter and most setae were removed from the pedipalp. In addition to these structures not being useful for identification, they are also not depicted accurately with photographs. Final images were compared with the specimen and edited accordingly to best approximate realistic coloration. In some cases, specimens prepared in glycerin jelly (i.e., coloration preserved) were not available and only the Hoyer’s slide preparations (i.e., coloration destroyed) were available. For these, micrographs were taken of the Hoyer’s slides and coloration was added in Photoshop (Figure 69, 71). Note that color micrographs depict the best representation of specific characters (venter, dorsum, pedipalp, subcapitulum), so a given figure may be a composite of multiple specimens (e.g., dorsum and subcapitulum of specimen A; venter of specimen B).

**Descriptions**: The descriptions contained herein are streamlined to include information that best diagnoses a given species, so we depart from the standard of previous descriptions in two important ways. First, we depict species with photomicrographs rather than line drawings, which greatly reduces the time necessary for a given description. Although this is not generally possible with mites, torrenticolids lend themselves to such representation because the characters needed to diagnose species, such as color pattern, are viewable from such images—a condition not common in most mite groups. Second, we depart from the suggestion of Goldschmidt (2007) and recent torrenticolid descriptions (e.g., Pešić 2014; Pešić and Gerecke 2014; Pešić and Smit 2014) in that we do not depict the ejaculatory complex or legs as these systems were not useful in differentiating species that were easily distinguished by other systems (e.g., color, various measurements and proportions). In fact, in those cases where two or more species were difficult to differentiate using our methods (e.g., Miniforma Group), we examined the ejaculatory complex and legs for additional characters. However, in no case could species that are difficult to identify be separated by ejaculatory complex or leg character systems. Therefore, although such systems are morphologically interesting, we decline to describe them here.

**Species delimitation**: An integrative approach to species delimitation was employed, which included a combination of morphological, molecular, and biogeographic characters. The development of species hypotheses was an iterative process. Initial hypotheses were created during the sorting stage with a stereomicroscope by grouping specimens into recognizable units of overall similarity (i.e., morphotypes). These hypotheses were tested with early iterations of phylogenetic analyses, which was the basis for identifying preliminary clades in need of further sampling. For example, distinctive morphotypes recovered as monophyletic, and with low genetic variability across their distribution (i.e., less than one percent difference in COI), were considered as putative species; additional specimens were not added unless found far outside the known geographic range. In contrast, when morphotypes were found to span multiple clades in COI trees, additional specimens from those lineages were included to generate more data in order to test hypotheses for those morphologically cryptic clades.

**Figure 4.**
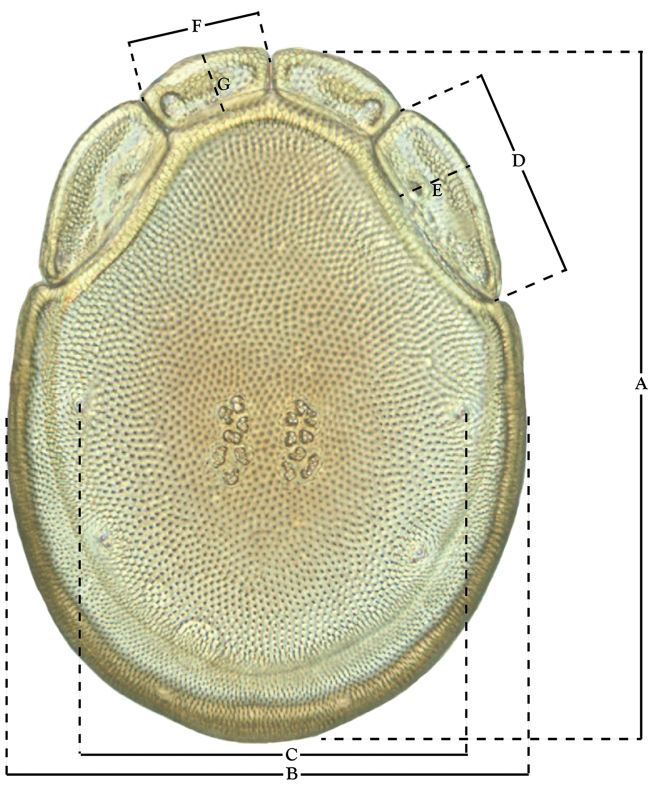
Dorsal measurements: **A** dorsal length **B** dorsal width **C** distance between dorsal glandularia, Dgl-4 **D** anterio-lateral platelet length **E** anterio-lateral platelet width **F** anterio-medial platelet length **G** anterio-medial platelet width. *T. multiforma* Habeeb, 1974 depicted.

**Figure 5.**
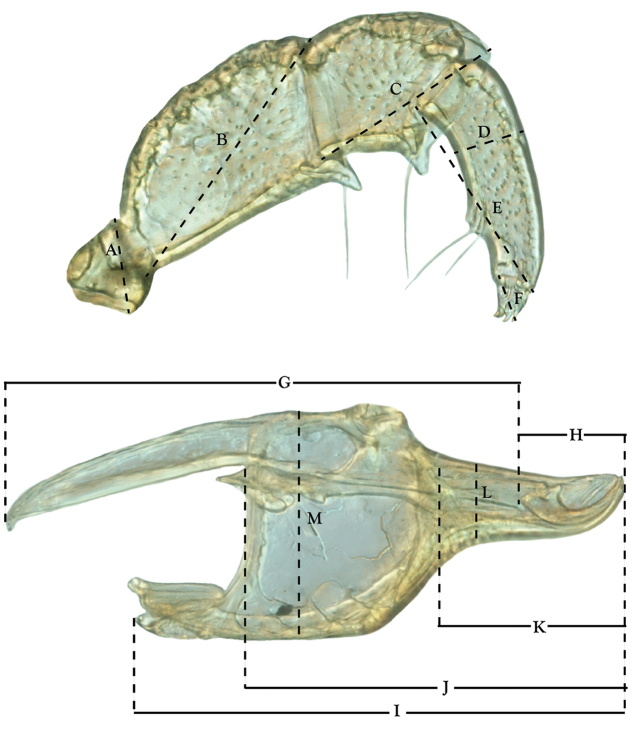
Gnathosomal measurements. Palpomeres – **A** trochanter length **B** femur length **C** genu length **D** tibia length **E** tibia width **F** tarsus length. Chelicerae – **G** cheliceral base length **H** fang length. Subcapitulum – **I** ventral length **J** dorsal length **K** rostrum length **L** rostrum width **M** height. *T. multiforma* Habeeb, 1974 depicted.

**Figure 6.**
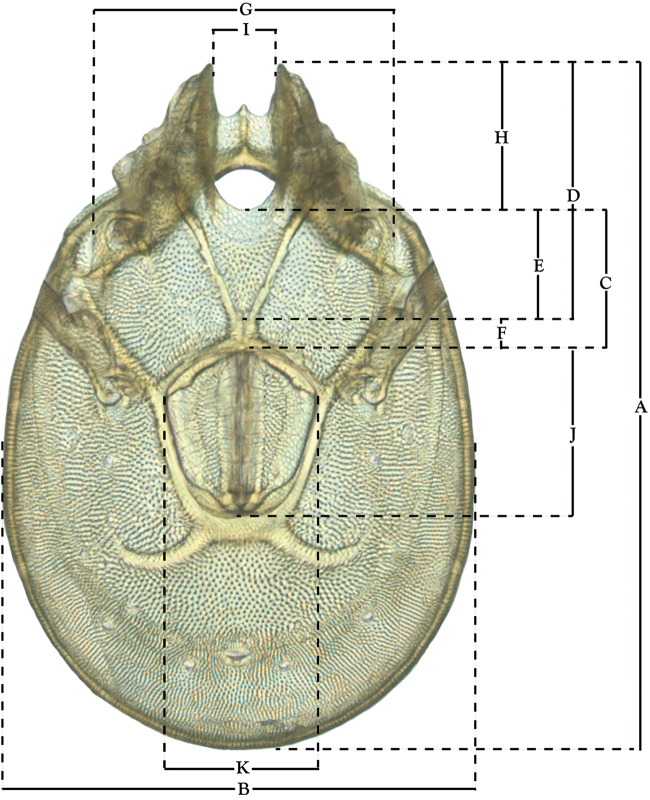
Venter measurements: **A** ventral length **B** ventral width **C** anterior venter **D** coxa-I total length **E** coxa-I medial length **F** medial suture length **G** coxa-III width **H** gnathosomal bay length **I** gnathosomal bay width **J** genital field length **K** genital field width. *T. multiforma* Habeeb, 1974 depicted.

**Measurements (Figure 4–6)**: We generally follow Fisher et al. (2015), who modified Goldschmidt (2007), but with the following modifications. Two measurements were added: the distance between Dgl-4 and the height of the rostrum. The following measurements were excluded as they were not found to be important in diagnosing species: distance from genital field to excretory pore; distance from genital field to cauda; dorsal plate length; gnathosomal bend depth; cheliceral height; width of palpomeres, except for the tibia; and all genital skeleton measurements. For each species, when available, a minimum of five individuals of each sex were measured. An effort was made to include members that spanned the range of variability (morphological and molecular) from across the geographic range.

To increase time-efficiency, Fisher et al. (2015) suggested digitally measuring compound light micrographs of specimens. However, we abandon that method herein, as many closely-related species of North American *Torrenticola* are identifiable only with precisely-measured ratios. Measurements were found to be highly inaccurate if taken from images that were not in crisp focus. High-quality, stacked images of each structure can negate this problem (e.g., Hamilton et al. 2016). However, creating such images of slide-prepared mites greatly increases the time invested per specimen and was beyond the scope of this study. Therefore, our measurements were taken with an objective micrometer on a Leica DM 2500 compound microscope.

**Molecular phylogenetics**: Samples from most sites were sorted to morphotype. Male and female representatives of each morphotype, per sample locality, were chosen for extraction. This resulted in clades containing members from across the geographic range, regardless of our initial speculations on species hypotheses. However, the high abundance of specimens in samples from the far west (California, Oregon, Washington) necessitated that each sample be scanned for morphotypes rather than sorting the entire sample. This was likely sufficient to find most species, but increased the likelihood of missing species present in low abundance. For example, despite obtaining abundant specimens of the Miniforma Group in nearly every sample, we were unable to re-collect *T. miniforma* Habeeb, 1974, even within the type locality.

To test species hypotheses and guide the description process, the barcoding region of COI was analyzed. We then used the resulting tree as a guide to increase sampling in certain geographic regions, or within certain morphotypes. The result is that many species are represented by specimens from across their distributions and spanning morphological variation. We also analyzed the D2-3 region of 28S rDNA in combination with COI to resolve relationships between species. This analysis included representatives of every species group. All sequences have been deposited in GenBank and accession numbers are located in Suppl. material 1. We follow the recommendation of Chakrabarty et al. (2013) in using GenSeq nomenclature.

Genomic DNA was extracted using Qiagen DNeasy Tissue Kit (Qiagen Inc., Valencia, Calif.). The target regions of COI (450 bp) and 28S (725 bp) were amplified with LCOI and HCOI (Folmer et al. 1994) and D23F and D6R (Park and Ó Foighill 2000), respectively, and purified with Qiagen QIAquick PCR Purification Kits. Test gels (1.5% agarose) confirmed PCR product quality. Purified PCR products were sequenced by Macrogen USA (Rockville, Maryland). Forward and reverse sequences were reconciled with DNASTAR© Lasergene SeqMan (Madison, Wisconsin). Resulting contigs were checked for contamination with BLASTn searches of NCBI’s nr database. Sequences were assessed for the presence of nuclear mitochondrial DNA segments (NUMT) by scanning for in-frame stop codons and indels (Song et al. 2008). COI sequences were aligned with Clustal X (Thompson et al. 1997) and 28S sequences were aligned with MAFFT Version 7 (Katoh and Standley 2013); both alignments were conservatively edited with BioEdit (Hall 1999). Bayesian analyses were performed with MrBayes (3.2.2) using the Extreme Science and Engineering Discovery Environment (XSEDE) infrastructure on the Cipres Portal (Miller et al. 2010), which submits jobs to the Gordon Compute Cluster, a network of 16 supercomputers sponsored by NSF XSEDE at the University of California, San Diego. Each analysis consisted of four simultaneous runs, each with four chains sampling every 1000 generations for 20 million generations, under a GTR+I+Γ model of molecular evolution; 50,000 trees were discarded as burn-in. The resulting majority-rule consensus trees were viewed with Dendroscope 3 (v. 3.5.7) (Huson and Scornavacca 2012); tree image files were then exported in PDF format and edited for final figures in Adobe Illustrator CS6.

**Type designation**: Earlier works have suggested that males should be used as holotypes due to the importance of characters of the genital skeleton (e.g., Goldschmidt 2007). At least with North American fauna, this is not the case, as females are often more distinctive than males, especially within difficult-to-identify lineages (e.g., Rusetria Complex). Therefore, we have chosen female specimens for most holotypes. Of all species in the region, only nine have male holotypes. Of these nine, four are only known from males (*T. anoplopalpa* Fisher & Dowling, sp. n.; *T. bittikoferae* Crowell, 1960; *T. dolichodactyla* Fisher & Dowling, sp. n.; and *T. longitibia* Fisher & Dowling, sp. n.) and four were previously described (*T. delicatexa* Habeeb, 1955; *T. neoanomala* Habeeb, 1957; *T. rufoalba* Habeeb, 1955; *T. tricolor* Habeeb, 1957). Only one species (*T. interiorensis* Fisher & Dowling, sp. n.) has been deliberately chosen herein to be represented by a male holotype, which we have done to reflect that this species can only be readily-differentiated from its sister species, *T. neoanomala*, with male characters.

When possible, allotypes (a paratypic member of the opposite sex as the holotype) that best embody the proposed species hypotheses have been selected from the paratypic series. Allotypes are connected to the holotype based upon sampling location, genetic variation, and character consistency. Allotypes are considered to be the best representatives of the paratypic series that are also connected to the holotype with greater confidence than other paratypes.

When possible, the entire type series is represented in our phylogenetic analyses. However, we have found that coloration is valuable for species identification, yet for some lineages a given sex is represented only by specimens preserved in Hoyer’s medium, which destroys coloration. To resolve this problem, we searched GAW-preserved material for representatives. These specimens lack molecular data, but in each instance, specimens were selected that adhere to our species hypothesis with high confidence. For example, specimens were preferred if from the same sample or region, and especially from collection events that also lacked similar species.

**Deposition**: Material examined for both newly-described and previously-described species (holotypes, allotypes, most paratypes, and most other material examined) were deposited in the Canadian National Collection of Insects, Arachnids, and Nematodes (CNC) in Ottawa, Canada. An exception is *T. bittikoferae* Crowell, 1960, for which we examined two paratypes deposited in the Ohio State University Acarology Laboratory (OSAL) collection. For all species, when additional specimens were available, representative paratypes and other material examined were also deposited in the Acari Collection of the University of Arkansas (ACUA) in Fayetteville, Arkansas.

## Results and discussion

**Summary of material**: Approximately 2,300 specimens representing 90 species were examined from nearly 500 localities spanning the United States, Canada, and a few collections in Mexico and Central America (Figure 2). Specimens preserved in ethanol (for morphology and molecular data) and GAW (for morphology alone) were available for most locations across the US and Canada, but only GAW samples were available from Mexico and Central America.

Herein, we discuss 90 species of *Torrenticola* from North America. Previously, 23 species were known from the United States and Canada. Herein we propose the following four synonymies: *T. mercedensis* (Marshall, 1943) is a junior synonym of *T. sierrensis* (Marshall, 1943); *T. rectiforma* Habeeb, 1974 is a junior synonym of *T. ellipsoidalis* (Marshall, 1943); *T. neoconnexa* Habeeb, 1957 is a junior synonym of *T. magnexa* Habeeb, 1955; *T. esbelta* Cramer, 1992 is a junior synonym of *T. boettgeri* KO Viets, 1977. Five species previously known only from Mexico and Central America are reported from the southwestern U.S.: *T. boettgeri* KO Viets, 1977; *T. keesdavidsi* Cramer, 1992; *T. kurtvietsi* Cramer, 1992; *T. lamellipalpis* Viets, 1997; and *T. rala* Cook, 1980. This raises the number of previously described species known from North America north of Mexico to 24. We also describe 66 new species, which raises the total number of species known from the U.S. and Canada to 90. All previously described species are re-described with color images and updated information.

**Figure 7.**
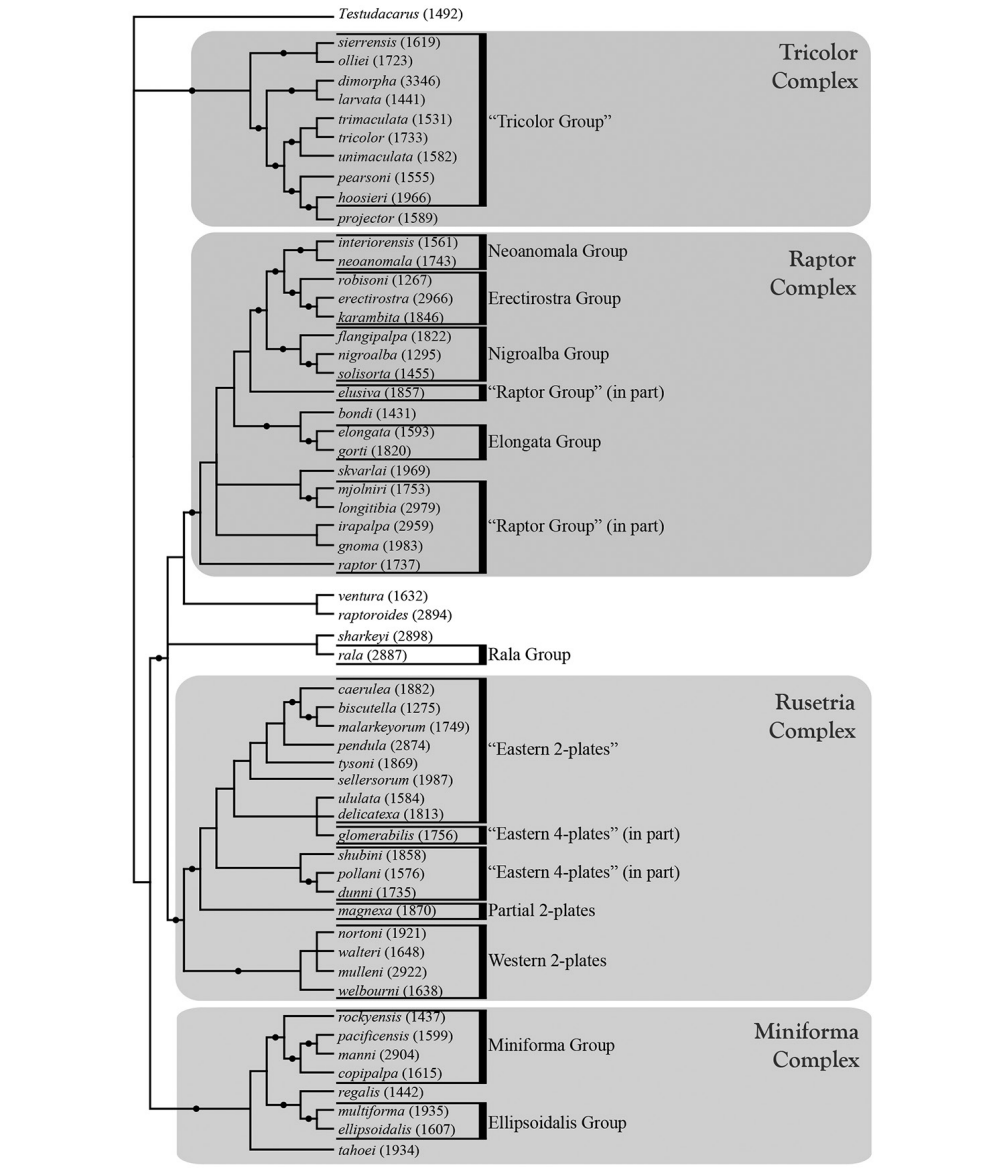
Bayesian inferred cladogram for combined analysis (28S+COI) of North American *Torrenticola*: species complexes and identification groups. Four well-supported complexes are organized into 13 groups that are helpful for identification. Note that 1) not all identification groups are monophyletic (in quotation marks); 2) not all species are placed in a complex; and 3) some species are not placed within a group, reflecting their identification difficulty. Numbers are DNA identification numbers. Dots denote posterior probably >95%.

**Figure 8.**
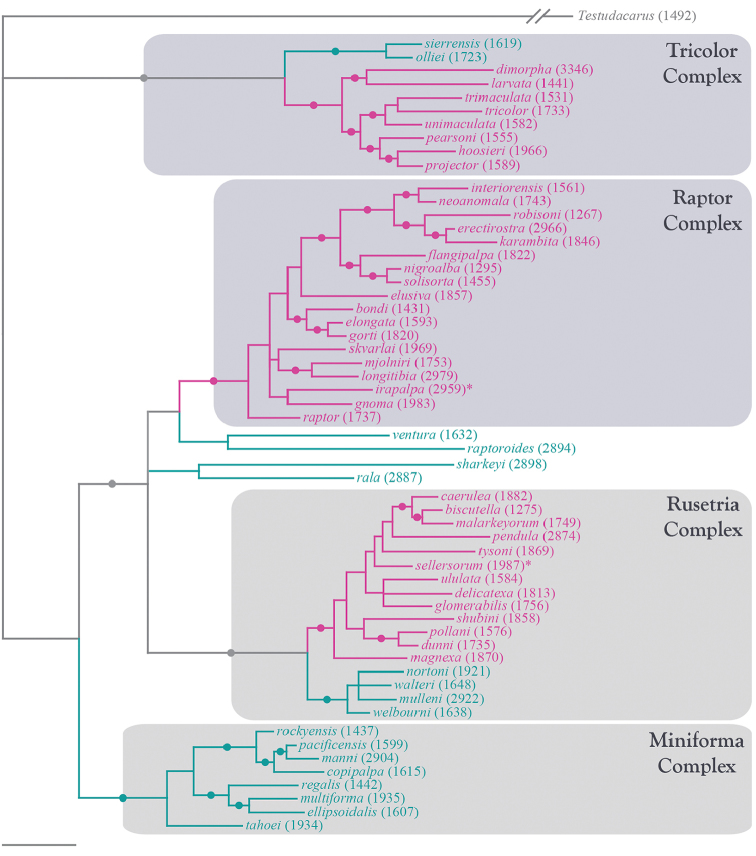
Bayesian inferred phylogram for combined analysis (28S+COI) of North American *Torrenticola*: distributions of 57 species. Pink represents lineages distributed in eastern North America and blue represents lineages distributed in western North America (gray branches represent lineages distributed in both regions). Species marked with an asterisk are primarily eastern but have distributions extending into the west. Note that larger lineages contain species distributed in either the east or west, but not both. Numbers are DNA identification numbers. Scale bar indicates 0.1 substitutions per site. Dots denote posterior probably >95%.

**Summary of phylogenetic analyses**: A combined dataset of 28S (725 bp) and COI (450 bp) of 42 species (Suppl. material 1) recovered four species complexes (described below) (Figure 7–8). These complexes were determined based upon a combination of monophyly and note-worthy characters (e.g., postero-lateral plate fusion in the Rusetria Complex; short, conical rostrum in the Tricolor Complex). The Rusetria and Raptor Complexes were recovered as closely related with strong support. However, we consider relationships among species complexes tentative, pending worldwide sampling. Within these complexes, 12 species groups are noted that are helpful for identification (an additional group, the Rala Group, does not fit within a complex), although many of these groups are not monophyletic.

**Figure 9.**
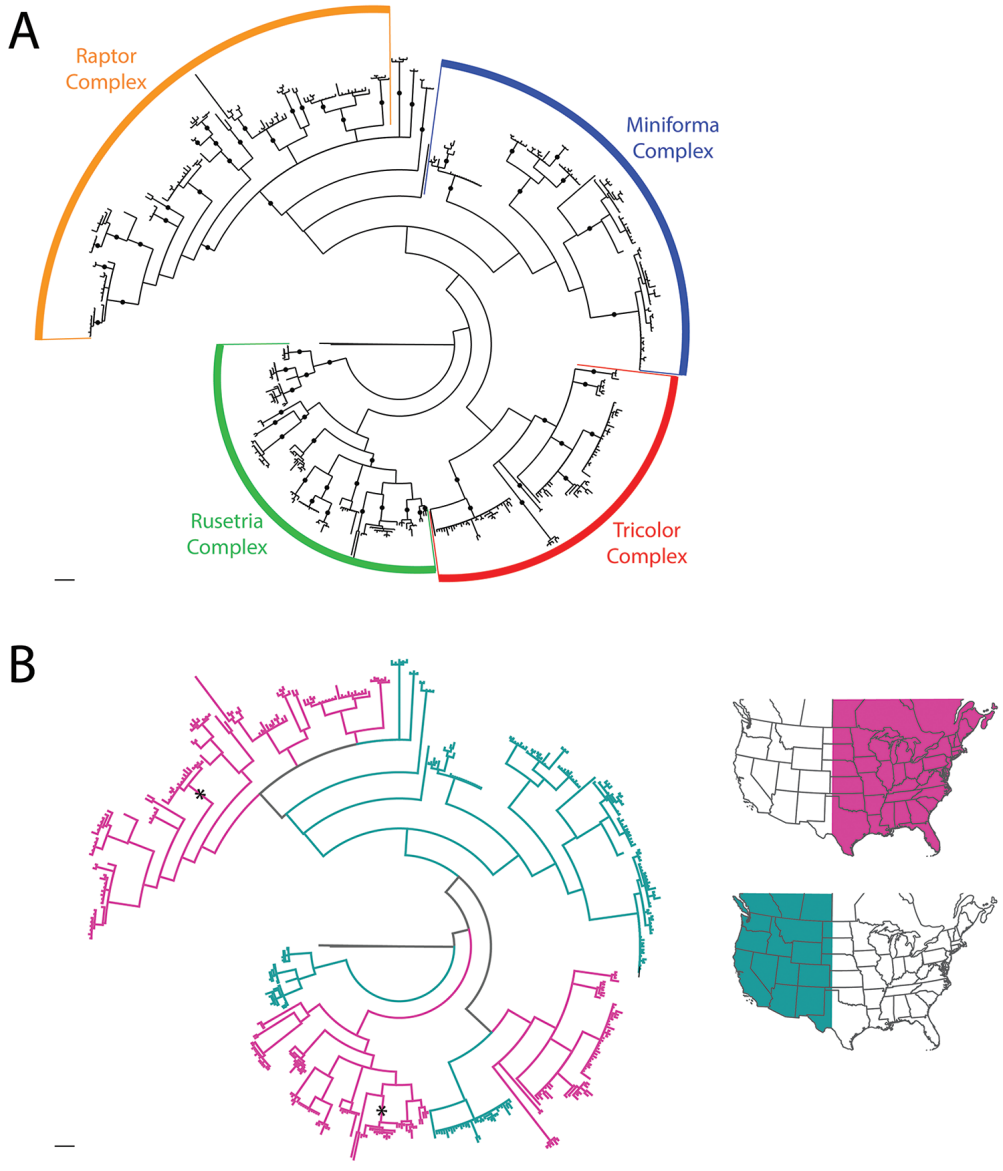
Bayesian inferred circular phylograms of North American *Torrenticola*, COI only. Scale bars indicate 1.0 substitutions per site. **A** Overall tree depicting the four complexes recovered in combined analysis. Dots denote posterior probability >95% **B** Species distributions; branch colors correspond to regions of North America depicted on the right (eastern, pink; western teal). Asterisks denote eastern species that extend into the west. Note that lineages tend to contain species distributed in either the east or west, but not both. Detail of this figure is presented in Figures 10–18.

**Figure 10.**
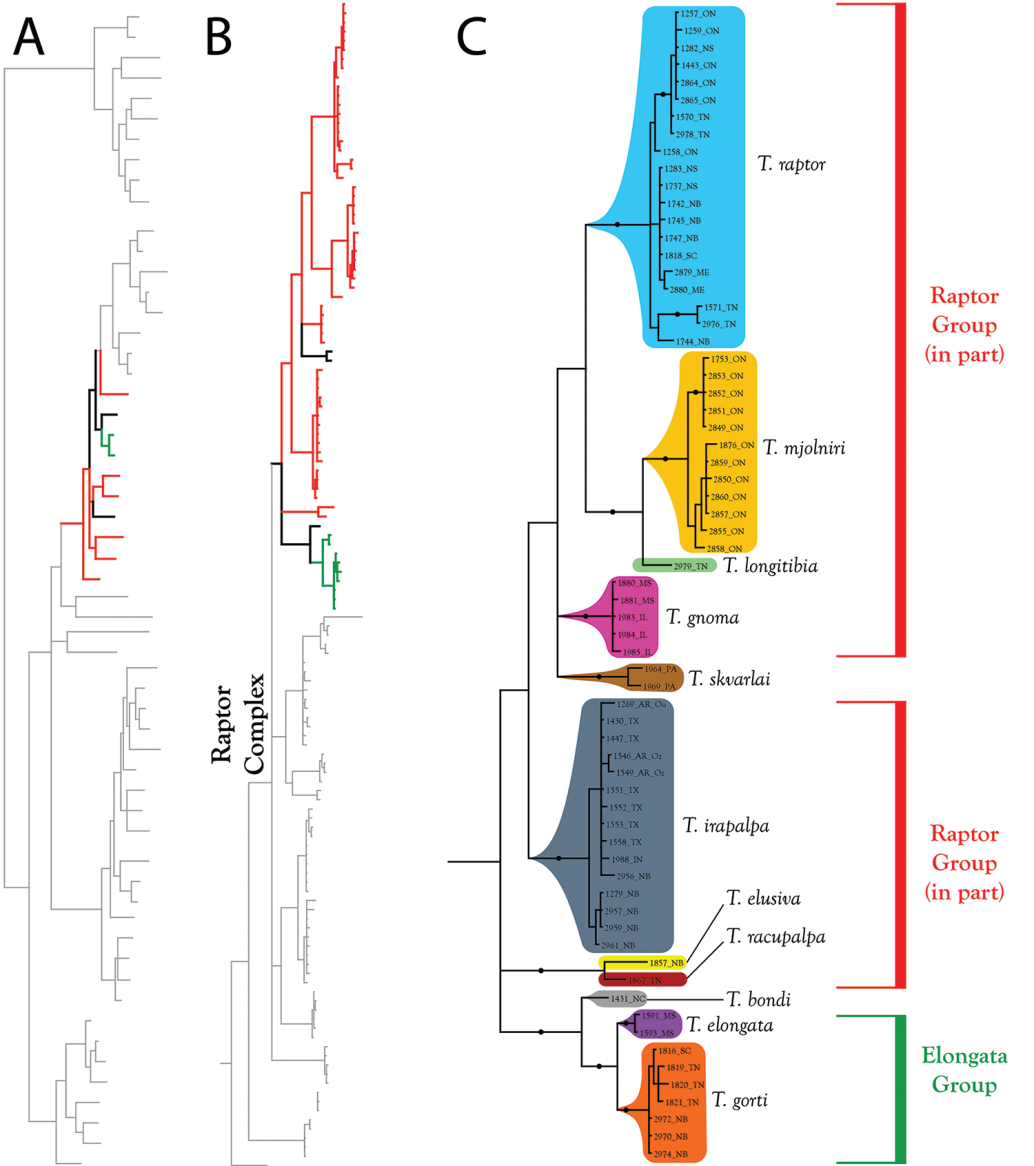
Raptor Complex (part I). These species are eastern, although *T. irapalpa* extends further west. Note that two species (*T. skvarlai* and *T. bondi*) do not fit into identification groups. **A** Species guide (overview of 28S+COI analysis from Figure 7–8) **B** Overview of Raptor Complex from Figure 9 (COI-only analysis from Figure 9) **C** Detail of Raptor Complex (in part) from B. Colored lineages in A and B correspond to Group names and brackets in C. Dots denote posterior probably of greater than 95%. Taxa are displayed by DNA number and state or territory abbreviation.

**Figure 11.**
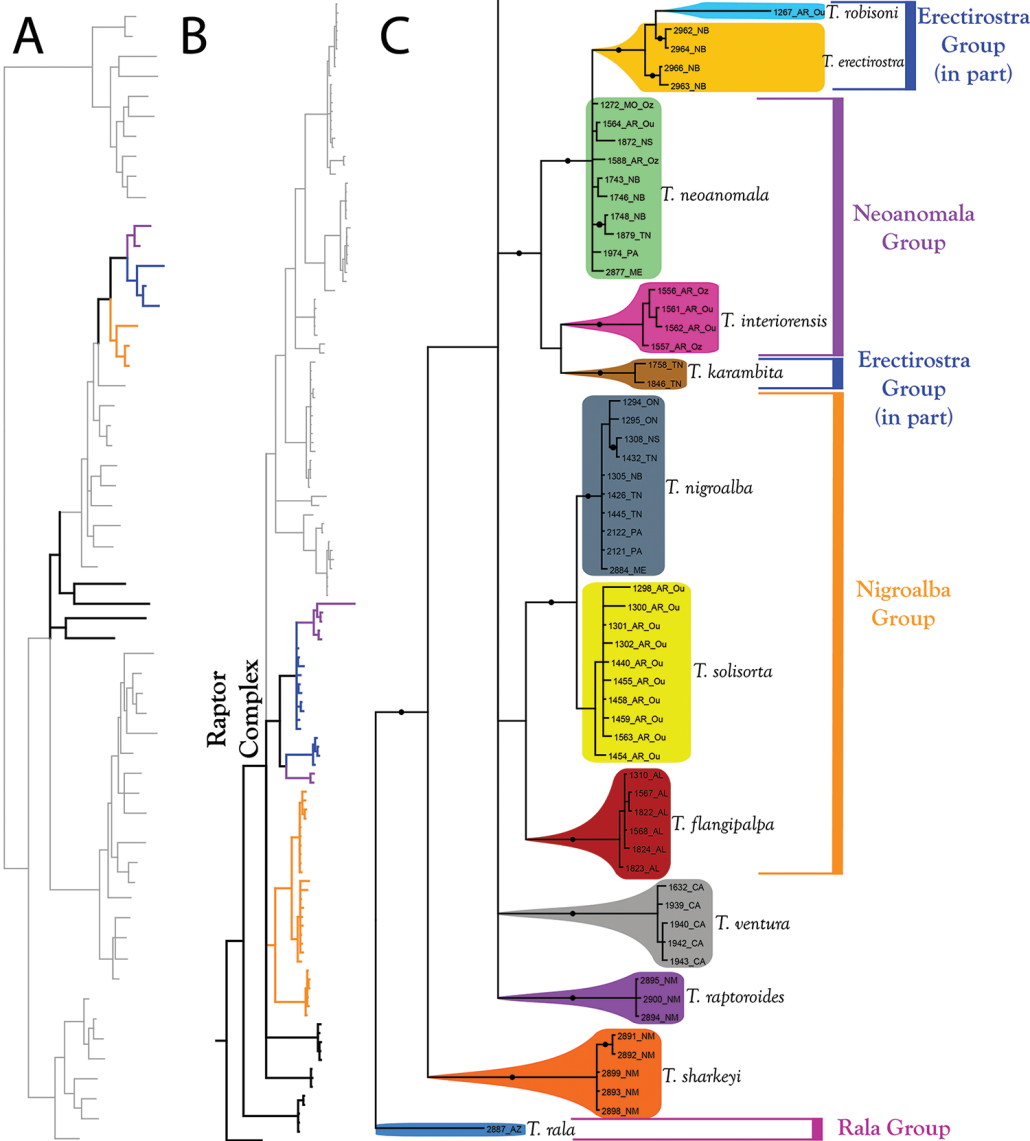
Raptor Complex (part II). Species of the Raptor Complex are eastern, but the species that do not fit into the complex (*T. rala*; *T. sharkeyi*; *T. raptoroides*; & *T. skvarlai*) are western. **A** Species guide (overview of 28S+COI analysis from Figure 7–8) **B** Overview of Raptor Complex from Figure 9 (COI-only analysis from Figure 9) **C** Detail of Raptor Complex (in part) from B. Colored lineages in A and B correspond to Group names and brackets in C. Dots denote posterior probably of greater than 95%. Taxa are displayed by DNA number and state or territory abbreviation.

**Figure 12.**
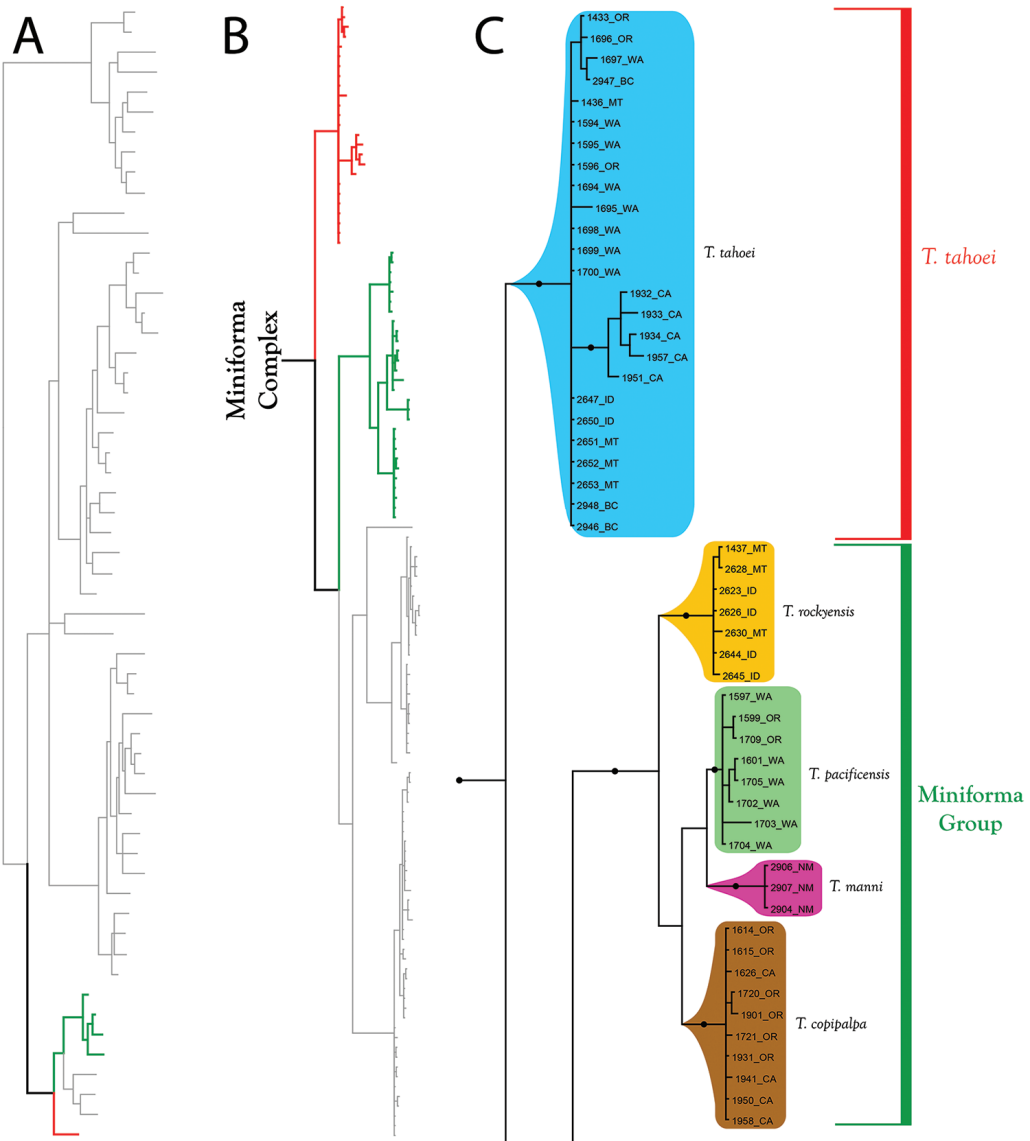
Miniforma Complex (part I). All species are western. Note that the phylogenetic structure in the Miniforma Group corresponds to biographic regions (Rockies, Pacific Ranges, southwest). **A** Species guide (overview of 28S+COI analysis from Figure 7–8) **B** Overview of Miniforma Complex from Figure 9 (COI-only analysis from Figure 9) **C** Detail of Miniforma Complex (in part) from B **C** Detail of Miniforma Complex (in part) from Figure 9. Colored lineages in A and B correspond to Group names and brackets in C. Dots denote posterior probably of greater than 95%. Taxa are displayed by DNA number and state (U.S.) or territory (Canada) abbreviation.

**Figure 13.**
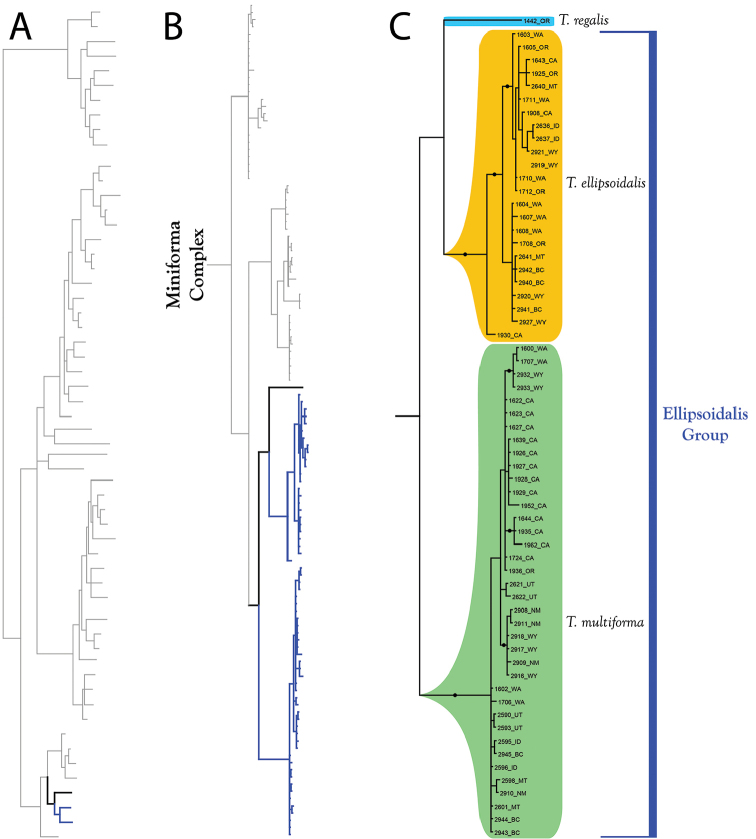
Miniforma Complex (part II). All species are western. One species (*T. regalis*) does not fit within a species group. Note that *T. ellipsoidalis* and *T. multiforma* are widespread in the west (C). **A** Species guide (overview of 28S+COI analysis from Figure 7–8) **B** Overview of Miniforma Complex from Figure 9 (COI-only analysis from Figure 9) **C** Detail of Miniforma Complex (in part) from B **C** Detail of Miniforma Complex (in part) from Figure 9. Colored lineages in A and B correspond to Group names and brackets in C. Dots denote posterior probably of greater than 95%. Taxa are displayed by DNA number and state (U.S.) or territory (Canada) abbreviation.

**Figure 14.**
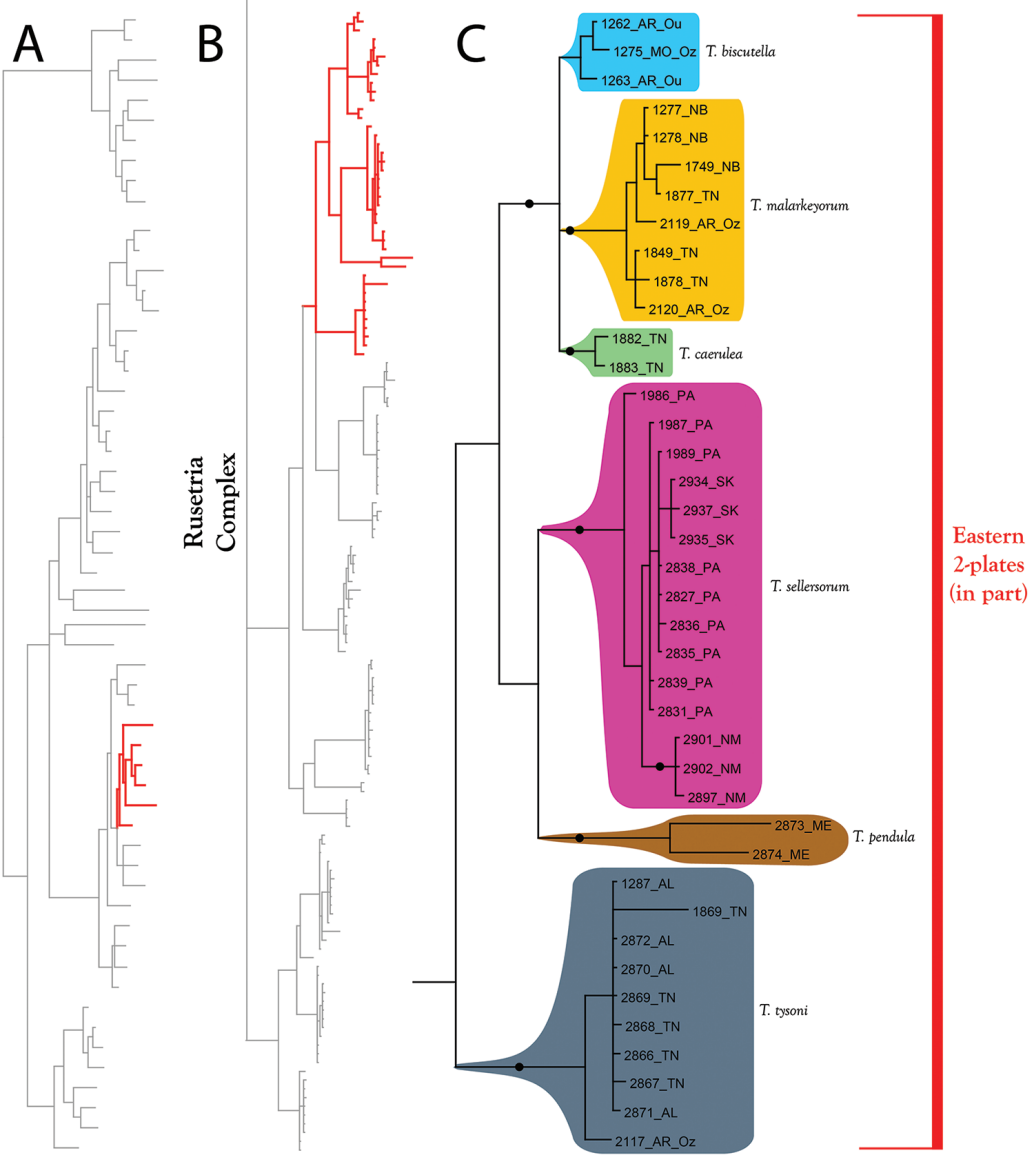
Rusetria Complex (part I). All species are eastern, but note the distribution of *T. sellersorum* extends into New Mexico and Saskatchewan. **A** Species guide (overview of 28S+COI analysis from Figure 7–8) **B** Overview of Rusetria Complex from Figure 9 (COI-only analysis from Figure 9) **C** Detail of Rusetria Complex (in part) from B **C** Detail of Rusetria Complex (in part) from Figure 9. Colored lineages in A and B correspond to Group names and brackets in C. Dots denote posterior probably of greater than 95%. Taxa are displayed by DNA number and state (U.S.) or territory (Canada) abbreviation.

**Figure 15.**
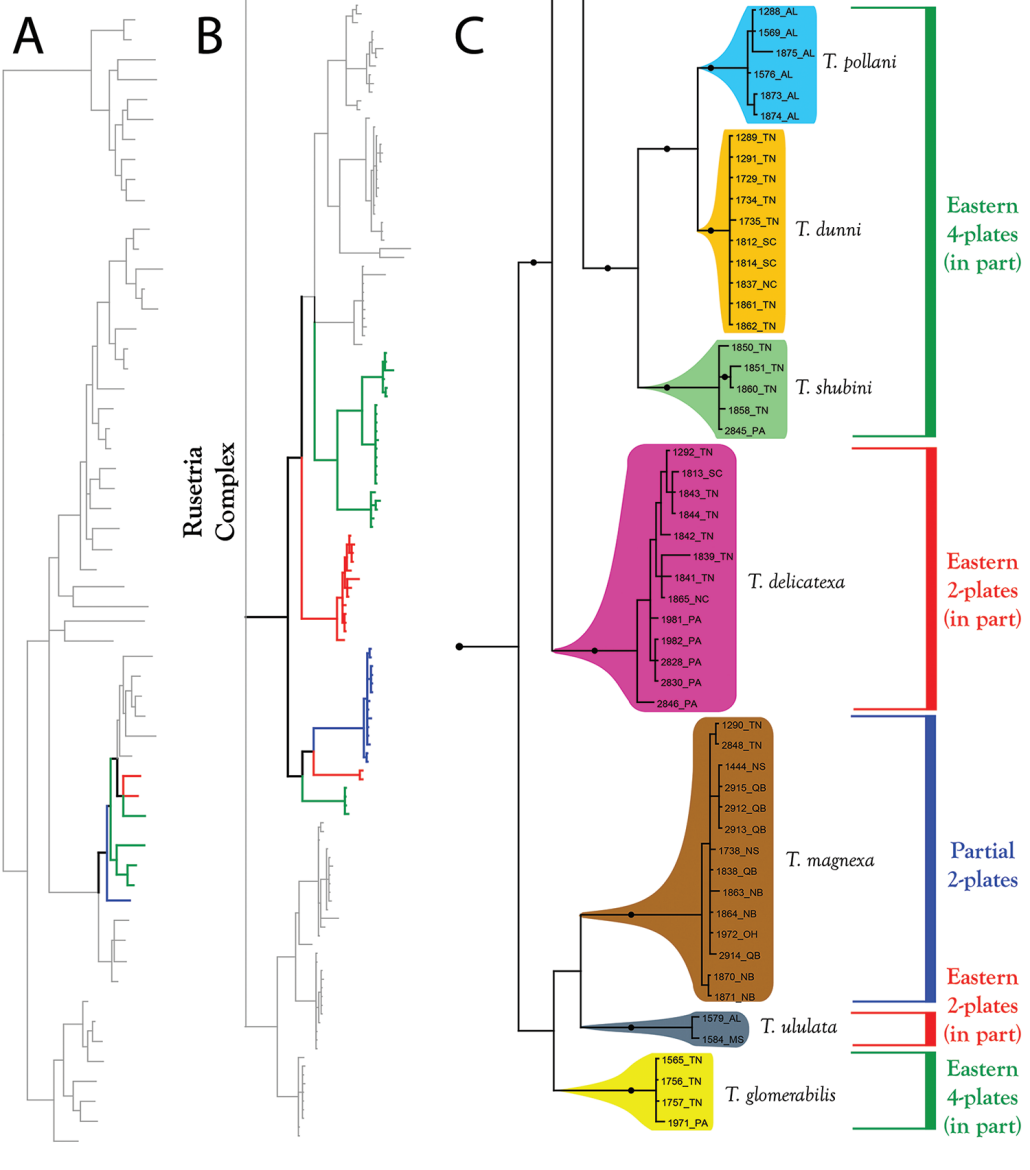
Rusetria Complex (part II). All species are eastern. Relationships among species groups remain unresolved, except for three species (*T. dunni*; *T. pollani*; and *T. shubini*), which form a well-supported clade in all analyses. **A** Species guide (overview of 28S+COI analysis from Figure 7–8) **B** Overview of Rusetria Complex from Figure 9 (COI-only analysis from Figure 9) **C** Detail of Rusetria Complex (in part) from B **C** Detail of Rusetria Complex (in part) from Figure 9. Colored lineages in A and B correspond to Group names and brackets in C. Dots denote posterior probably of greater than 95%. Taxa are displayed by DNA number and state (U.S.) or territory (Canada) abbreviation.

**Figure 16.**
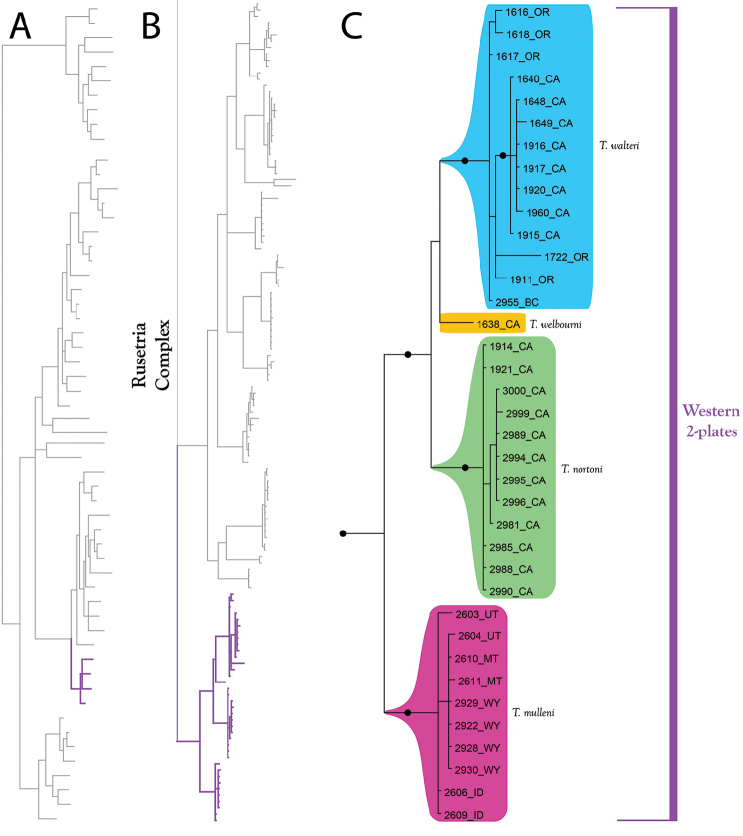
Rusetria Complex (part III). All species are western. **A** Species guide (overview of 28S+COI analysis from Figure 7–8) **B** Overview of Rusetria Complex from Figure 9 (COI-only analysis from Figure 9) **C** Detail of Rusetria Complex (in part) from B. **D** Detail of Rusetria Complex (in part) from Figure 9. Colored lineages in A and B correspond to Group names and brackets in C. Dots denote posterior probably of greater than 95%. Taxa are displayed by DNA number and state (U.S.) or territory (Canada) abbreviation.

**Figure 17.**
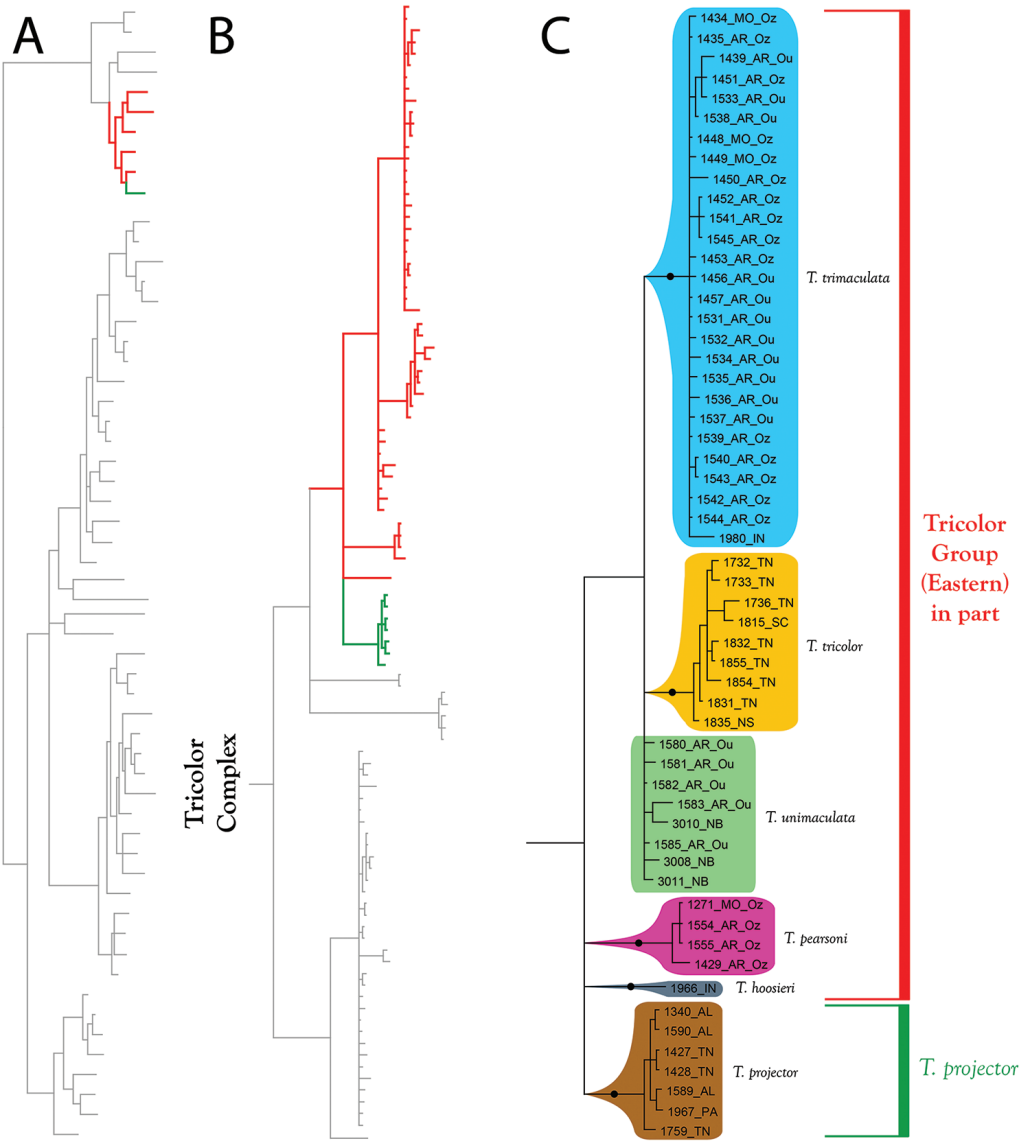
Tricolor Complex (part I). All species are eastern. These are some of the most recognizable of all *Torrenticola*, namely the elongate *T. projector* and a clade of species with diagnostic color patterns (*T. trimaculata*, *T. unimaculata*, and *T. tricolor*). Note that three species (*T. pearsoni*, *T. hoosieri*, and *T. projector*) are repeated in Figure 18. **A** Species guide (overview of 28S+COI analysis from Figure 7–8) **B** Overview of Tricolor Complex from Figure 9 (COI-only analysis from Figure 9) **C** Detail of Tricolor Complex (in part) from B **C** Detail of Tricolor Complex (in part) from Figure 9. Colored lineages in A and B correspond to Group names and brackets in C. Dots denote posterior probably of greater than 95%. Taxa are displayed by DNA number and state (U.S.) or territory (Canada) abbreviation.

**Figure 18.**
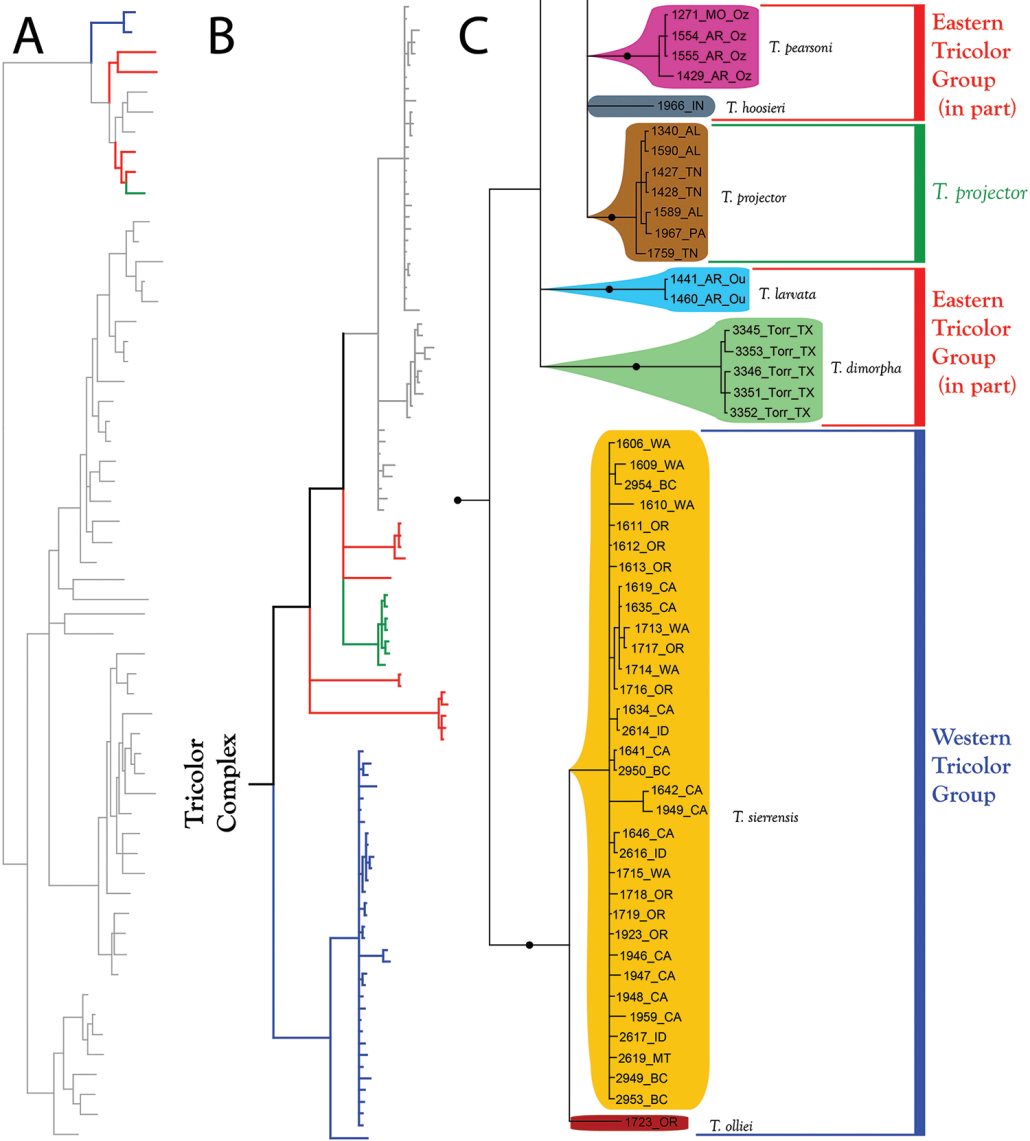
Tricolor Complex (part II). Most species of the Tricolor Complex are eastern, but one lineage is western: *T. sierrensis*, widespread; and *T. olliei*, north Pacific coast. The most apomorphic *Torrrenticola* (*T. dimorpha*) is recovered here in all analyses. Note that three species (*T. pearsoni*, *T. hoosieri*, and *T. projector*) are repeated in Figure 17. **A** Species guide (overview of 28S+COI analysis from Figure 7–8) **B** Overview of Tricolor Complex from Figure 9 (COI-only analysis from Figure 9) **C** Detail of Tricolor Complex (in part) from B **C** Detail of Tricolor Complex (in part) from Figure 9. Colored lineages in A and B correspond to Group names and brackets in C. Dots denote posterior probably of greater than 95%. Taxa are displayed by DNA number and state (U.S.) or territory (Canada) abbreviation.

COI sequence data were obtained for 481 individuals from across the United States and Canada (Suppl. material 1), including all specimens from the combined analysis. These 481 individuals group into 57 clades that represent well-supported species hypotheses (Figures 9–18). Of these 57 species hypotheses, 13 clades are identifiable to previously-described species: *T. delicatexa* Habeeb, 1955; *T. ellipsoidalis* (Marshall, 1943); *T. larvata* Cherri, Fisher, & Dowling, 2016; *T. multiforma* Habeeb, 1974; *T. neoanomala* Habeeb, 1957; *T. magnexa* Habeeb, 1955; *T. nigroalba* Habeeb, 1955; *T. projector* Habeeb, 1961; *T. rala* Cook, 1980; *T. sierrensis* (Marshall, 1943); *T. tahoei* (Marshall, 1943), *T. tricolor* Habeeb, 1957, *T. trimaculata* Fisher, 2015; *T. ventura* Habeeb, 1973. Two clades include multiple previously-named species, necessitating the synonymization of three species. Ethanol-preserved specimens of seven previously described species, known from the United States and Canada (*T. bittikoferae* Crowell, 1960; *T. indistincta* (Marshall, 1929); *T. kittatinniana* Habeeb, 1955; *T. miniforma* Habeeb, 1974; *T. occidentalis* (Marshall, 1933); and *T. rufoalba*), were not available, but the type series for each of these were examined morphologically. Of the 57 species in our molecular analysis, 44 represent new species.

**Species Complexes and Groups (Table 1)**: As considered herein, species complexes are monophyletic collections of closely-related species recovered by our combined (COI+28S) phylogenetic analyses (Figure 7–8). In addition, most species known only from morphology are placed within the tree in suspected complexes. Species complexes proposed herein and their relationships to each other should be considered a first step in need of future exploration that will greatly benefit from worldwide sampling. Our analyses recover four species complexes: Miniforma; Raptor; Rusetria; and Tricolor.

**Table 1.** Distribution of the 90 species of *Torrenticola* from the US and Canada among Species Complexes and Groups. Number of species within a group are denoted parenthetically. Species for which molecular data was available are denoted with an asterisk. Species known only from one sex are denoted with sex symbols (♀ or ♂). Species Groups in quotation marks were **not** recovered as monophyletic in our molecular analyses.

**Tricolor Complex (14 sp.)** 1. “Tricolor Group” (13 sp.) a. *T. bittikoferae* ♂ b. *T. cardia* c. T. dimorpha* d. T. hoosieri* e. *T. kringi* f. T. larvata* g. *T. mohawk* h. T. olliei* i. T. pearsoni* j. T. sierrensis* k. T. tricolor* l. T. trimaculata* m. T. unimaculata* 2. Unplaced (1 sp.) a. T. projector* **Miniforma Complex (13 sp.)** 1. Ellipsoidalis Group (4 sp.) a. T. ellipsoidalis* b. *T. leviathan* c. T. multiforma* d. *T. occidentalis* ♀ 2. Miniforma Group (7 sp.) a. T. copipalpa* b. T. manni* c. *T. miniforma* d. *T. oliveri* e. T. pacificensis* f. *T. pinocchio* g. T. rockyensis* 3. Unplaced (2 sp.) a. T. regalis* b. T. tahoei*	**Raptor Complex (24 sp.)** 1. Elongata Group (3 sp.) a. T. elongata* b. T. gorti* c. *T. reduncarostra* 2. Erectirostra Group (3 sp.) a. T. erectirostra* b. T. karambita* c. T. robisoni* 3. Neoanomala Group (2 sp.) a. T. interiorensis* b. T. neoanomala* 4. Nigroalba Group (4 sp.) a. *T. dentirostra* b. T. flangipalpa* c. T. nigroalba* d. T. solisorta* 5. “Raptor Group” (10 sp.) a. *T. daemon* b. *T. danielleae* c. T. elusiva* ♀ d. T. gnoma* e. T. irapalpa* f. *T. ivyae* g. T. longitibia* ♂ h. T. mjolniri* i. T. racupalpa* j. T. raptor* 6. Unplaced (2 sp.) a. T. bondi* b. T. skvarlai*	**Rusetria Complex (26 sp.)** 1. “Eastern 2-plates” (12 sp.) a. T. bicutella* b. T. caerulea* c. T. delicatexa* d. *T. feminellai* e. *T. indistincta* f. T. malarkeyorum* g. T. microbiscutella* h. T. pendula* i. T. sellersorum* j. T. tysoni* k. T. ululata* l. *T. whitneyae* 2. “Eastern 4-plates” (6 sp.) a. T. dunni* b. T. glomerabilis* c. *T. kittatinniana* d. T. pollani* e. *T. rufoalba* f. T. shubini* 3. Partial 2-plates (4 sp.) a. *T. folkertsae* b. T. magnexa* c. *T. priapus* d. *T. pulchra* 4. Western 2-plates (4 sp.) a. T. mulleni* b. T. nortoni* c. T. walteri* d. T. welbourni* ♀
	Unplaced (13 sp.) 1. Rala Group (7 sp.) a. *T. anoplopalpa* ♂ b. *T. boettgeri* c. *T. dolichodactyla* ♂ d. *T. keesdavidsi* e. *T. kurtvietsi* f. *T. lamellipalpis* b. T. rala*	2. Unplaced (6 sp.) a. *T. arktonyx* b. T. raptoroides* c. T. sharkeyi* d. T. ventura* e. *T. wiedenmanni* f. *T. oregonensis*

Four western species included in the molecular analysis do not fit into a species complex: *T. ventura* Habeeb, 1973; *T. raptoroides* Fisher & Dowling, sp. n.; *T. sharkeyi* Fisher & Dowling, sp. n.; and *T. rala* Cook, 1980. Additionally, fresh material for molecular analysis was not available for six other species that are suspected to be closely related to *T. rala* and therefore included in the Rala Group (below): *T. boettgeri* KO Viets, 1977; *T. kurtvietsi* Cramer, 1992; *T. lamellipalpis* KO Viets, 1977; *T. keesdavidsi* Cramer, 1992; *T. dolichodactyla* Fisher & Dowling, sp. n.; and *T. anoplopalpa* Fisher & Dowling, sp. n. Collectively these species (*T. ventura*, *T. raptoroides*, *T. sharkeyi* and the Rala Group), resemble fauna from south of the US. The phylogenetic affinity of these species can only be determined with the addition of worldwide fauna, especially from Mexico and Central America.

As considered herein, “species groups” represent closely related species that are readily identifiable to the species group level, often even under low magnification (i.e., stereoscope). The function of these species groups is to aid identification, as often species of a group are easier to recognize at the group-level than the species-level. Occasionally, identifying a given specimen to species-level requires merely combining group-level identification with locality. Importantly, four species groups are not monophyletic: Tricolor Group, Raptor Group, Eastern 4-Plates, and Eastern 2-Plates. This is because these species groups serve to ease identification, not to inform relationship. However, despite the utility of learning these species groups, they are meant to merely augment the key. Accurate identification of most species still requires keying slide preparations under higher magnification (i.e., compound microscope) with precise measurements.

Thirteen species do not fit into species groups. Three of these species are among the most recognizable species in the genus, but are single-species and thus not “groups”: *T. arktonyx* Fisher & Dowling, sp. n.; *T. projector* Habeeb, 1961; and *T. tahoei* (Marshall, 1943). Nine of the 13 species are not placed within a species group because they are difficult to identify under lower magnification and have little in common with sister species. The reader is referred to the key and diagnoses for these species: *T. oregonensis* Fisher & Dowling, sp. n.; *T. wiedenmanni* Fisher & Dowling, sp. n.; *T. bondi* Fisher & Dowling, sp. n.; *T. occidentalis* (Marshall, 1933); *T. raptoroides* Fisher & Dowling, sp. n.; *T. regalis* Fisher & Dowling, sp. n.; *T. sharkeyi* Fisher & Dowling, sp. n.; *T. skvarlai* Fisher & Dowling, sp. n.; and *T. ventura* Habeeb, 1973.

The **Rala Group** (Figure 7–9, 11) is the only species group that does not fit within a species complex. This group comprises seven species: *T. boettgeri*; *T. kurtvietsi*; *T. lamellipalpis*; *T. keesdavidsi*; *T. rala*; *T. dolichodactyla*; and *T. anoplopalpa*. These species are colorless and have incomplete or indistinct hind coxal margins. They are southwestern (Arizona, New Mexico, and Texas), with a few extending into Mexico and Guatemala. However, we suspect all seven species extend well into Mexico and perhaps further south, with US populations representing the northern-most extents of their distributions.

**Raptor Complex (Figure 7–11)**: Molecular analyses recovered 19 species in this complex. One of these species, *T. racupalpa* Fisher & Dowling, sp. n., could not be included in the combined dataset, but was included in the COI analysis, where it was recovered as sister to *T. elusiva* Fisher & Dowling, sp. n. Additionally, fresh material for molecular analysis was not available for five other species suspected as being within this complex based upon morphology: *T. danielleae* Fisher & Dowling, sp. n.; *T. daemon* Fisher & Dowling, sp. n.; *T. ivyae* Fisher & Dowling, sp. n.; *T. reduncarostra* Fisher & Dowling, sp. n.; and *T. dentirostra* Fisher & Dowling, sp. n. In total, there are 24 species within the Raptor Complex.

All members of the Raptor Complex are eastern. A collection of western species (*T. raptoroides* Fisher & Dowling, sp. n.; *T. sharkeyi* Fisher & Dowling, sp. n.; *T. ventura* Habeeb, 1973; and the Rala Group) that do not form a monophyletic group, are often recovered as sister to the Raptor Complex. This suggests that the Raptor Complex may have originated from a Neotropical ancestor that dispersed into the eastern US and subsequently diversified. However, this speculation requires worldwide sampling for support.

Basal lineages within the Raptor Complex remain unsupported/unresolved, but a few relationships within the complex were recovered with high support. First, the Erectirostra Group was not recovered as monophyletic in the COI analysis, but is well-supported in the combined analysis. Second, the Neoanomala and Erectirostra Groups are closest relatives; indeed, apart from the apomorphic subcapitulum of the Erectirostra Group, the two groups are otherwise similar. Third, a sister relationship between the Erectirostra + Neoanomala lineage and the Nigroalba Group was recovered with high support. Finally, both analyses support a sister relationship between *T. bondi* Fisher & Dowling, sp. n. and the Elongata Group.

22 of the 24 species of the Raptor Complex are organized into five species groups: Raptor Group, Elongata Group, Nigroalba Group, Erectirostra Group, and Neoanomala Group. Two species do not fit within an identification group: *T. bondi* Fisher & Dowling, sp. n. and *T. skvarlai* Fisher & Dowling, sp. n.

The **Raptor Group** (Figure 10) comprises ten eastern species: *T. danielleae* Fisher & Dowling, sp. n.; *T. daemon* Fisher & Dowling, sp. n.; *T. ivyae* Fisher & Dowling, sp. n.; *T. gnoma* Fisher & Dowling, sp. n.; *T. irapalpa* Fisher & Dowling, sp. n.; *T. longitibia* Fisher & Dowling, sp. n.; *T. mjolniri* Fisher & Dowling, sp. n.; *T. elusiva* Fisher & Dowling, sp. n.; *T. racupalpa* Fisher & Dowling, sp. n.; and *T. raptor* Fisher & Dowling, sp. n. These closely related species are readily identifiable with the following combination of characters: round bodies; Dgl-4 close to muscles scars; long thin subcapitular rostra; and long, thin pedipalp tibiae. This group was not recovered as monophyletic in our analyses; however, relationships within this group are also not well-supported, so neither monophyly nor non-monophyly can be rejected.

The **Elongata Group** (Figure 10) comprises three eastern species: *T. elongata* Fisher & Dowling, sp. n.; *T. reduncarostra* Fisher & Dowling, sp. n.; and *T. gorti* Fisher & Dowling, sp. n. Members of this distinctive group are readily identifiable by the combination of characters: small body size; bold, distinctive coloration; and elongate, ovoid body shape. No other species share this combination of characters. Furthermore, no other *Torrenticola* have similar coloration to the dark morph of *T. gorti* and no other *Torrenticola* are as elongate as *T. elongata*.

The **Nigroalba Group** (Figure 11) comprises four eastern species: *T. flangipalpa* Fisher & Dowling, sp. n.; *T. dentirostra* Fisher & Dowling, sp. n.; *T. nigroalba* Habeeb, 1955; and *T. solisorta* Fisher & Dowling, sp. n. These closely related species are readily identifiable with the following combination of characters: small body size; long, thin subcapitular rostra and pedipalp tibiae; dorsal purple coloration restricted to posterior half; somewhat elongate bodies; and hind coxal margins that are distinctly present, but the margins of are poorly defined. Several western species can superficially resemble members of the Nigroalba Group under low magnification (e.g., *T. tahoei* and *T. regalis*).

The **Erectirostra Group** (Figure 11) comprises three eastern species: *T. erectirostra* Fisher & Dowling, sp. n.; *T. karambita* Fisher & Dowling, sp. n.; and *T. robisoni* Fisher & Dowling, sp. n. These closely related species are readily identifiable with the following combination of characters: thick subcapitular rostra (when viewed ventrally) that is strongly upturned and dentate and Cxgl-4 nearly halfway down gnathosomal bay. No other *Torrenticola* in North America have similar subcapitular rostra and members of the Erectirostra Group can be readily sorted under low magnification. In terms of body shape and coloration, they superficially resemble Neoanomala Group, to which they are closely related.

The **Neoanomala Group** (Figure 11) comprises two eastern species: *T. interiorensis* Fisher & Dowling, sp. n. and *T. neoanomala* Habeeb, 1957. These closely related species are readily identifiable with the following combination of characters: dorsal coloration often purplish, separated into anterior and posterior portions; hind coxal apodemes distinct; and other characters relatively unmodified. Members of the Neoanomala Group are readily differentiated from the superficially similar Rusetria Group by having distinct hind coxal apodemes, whereas all members of the Rusetria Group have indistinct hind coxal apodemes. Another species—*T. bondi* Fisher & Dowling, sp. n.—resembles members of this group, but is not closely related. *T. bondi* have a shorter medial suture (10–15 µm in female *T. bondi*, 22–40 µm in female Neoanomala Group; 55–70 µm in male *T. bondi*, 75–110 µm in male Neoanomala Group). Also, *T. bondi* is currently known only from Haywood County (North Carolina), which implies that this species is either rare, or has a restricted distribution.

**Miniforma Complex (Figure 7–9, 12–13)**: Molecular analyses recovered eight western species in this complex. Additionally, fresh material for molecular analysis was not available for five other species (*T. occidentalis* (Marshall, 1933); *T. miniforma* Habeeb, 1974; *T. oliveri* Fisher & Dowling, sp. n.; *T. leviathan* Fisher & Dowling, sp. n.; and *T. pinocchio* Fisher & Dowling, sp. n.) that are suspected to be within this complex based upon morphology. Another species, *T. oregonensis* Fisher & Dowling, sp. n., superficially resembles members of this complex, but is divergent enough that we avoid placing this species pending molecular support. Therefore, in total, we propose 13 species in this complex.

These species are organized into two monophyletic species groups: Ellipsoidalis Group and Miniforma Group. The two members of this complex that are not placed within a species group include *T. tahoei* (Marshall, 1943), which is among the most recognizable species of the genus, and *T. regalis* Fisher & Dowling, sp. n., which can only be confidently identified by keying slide-mounted specimens. *Torrenticola tahoei* is consistently recovered as sister to the rest of the species in this complex and *T. regalis* is consistently recovered sister to Ellipsoidalis Group.

The **Miniforma Group** (Figure 12) comprises seven western species: *T. manni* Fisher & Dowling, sp. n.; *T. oliveri* Fisher & Dowling, sp. n.; *T. pinocchio* Fisher & Dowling, sp. n.; *T. miniforma* Habeeb, 1974; *T. pacificensis* Fisher & Dowling, sp. n.; *T. rockyensis* Fisher & Dowling, sp. n.; and *T. copipalpa* Fisher & Dowling, sp. n. Members of this group are often readily identified by their smaller body size than other *Torrenticola* within their range except for Wester 2-Plates (*T. mulleni* Fisher & Dowling, sp. n.; *T. nortoni* Fisher & Dowling, sp. n.; *T. walteri* Fisher & Dowling, sp. n.; and *T. welbourni* Fisher & Dowling, sp. n.). Members of the Miniforma Group are easily distinguished from Western 2-Plates, which have dorso-lateral platelets fused to the dorsal plate. The Miniforma Group can be further differentiated from all other *Torrenticola* by their distinctive pedipalp genual extensions. Females of this group have a relatively large genital field, which we suspect coincides with loss of larval parasitism.

The **Ellipsoidalis Group** (Figure 13) comprises four western species: *T. occidentalis* (Marshall, 1933); *T. ellipsoidalis* (Marshall, 1943); *T. leviathan* Fisher & Dowling, sp. n.; and *T. multiforma* Habeeb, 1974. Members of this group are readily identified from co-occurring *Torrenticola* via their larger body size than other species in a given sample. *Torrenticola occidentalis* may fit within this species group based upon overall similarity and by having a short, conical rostrum, but we were not able to obtain fresh material to confirm this hypothesis.

**Rusetria Complex (Figure 7–9, 14–16)**: Molecular analyses recovered 17 species in this complex. Additionally, fresh material for molecular analysis was not available for nine other species that are included here based upon morphology: *T. rufoalba* Habeeb, 1955; *T. kittatinniana* Habeeb, 1955; *T. indistincta* (Marshall, 1929); *T. whitneyae* Fisher & Dowling, sp. n.; *T. microbiscutella* Fisher & Dowling, sp. n.; *T. feminellai* Fisher & Dowling, sp. n.; *T. priapus* Fisher & Dowling, sp. n.; *T. folkertsae* Fisher & Dowling, sp. n.; and *T. pulchra* Fisher & Dowling, sp. n. In total, there are 26 species in this complex.

Unlike other species complexes, the Rusetria Complex corresponds to a previously recognized subgenus—“*Rusetria*”—that occurs worldwide and is identified by the fusion of the lateral platelets. Goldschmidt (2007) called species with fused lateral platelets “*Rusetria*-like species” and we continue that terminology here. However, our analyses indicate that a reversal to free lateral platelets occurred twice: once in a clade sister to the Eastern 2-Plates and once within the Eastern 2-Plates (*T. glomerabilis*). Members of the Rusetria Complex can also be differentiated from superficially similar species by having indistinct hind coxae (distinct in most similar species, such as the Neoanomala and Raptor Groups). Only a few other species have indistinct hind coxal margins (*T. dolichodactyla*, *T. skvarlai*, and occasionally *T. flangipalpa*) or partial hind coxal margins (*T. regalis*, *T. sharkeyi*, and membes of the Nigroalba and Rala Groups,). This character is particularly useful when differentiating Eastern 4-Plates from similar species.

Two interesting evolutionary stories are suggested by the combined molecular analysis. The first involves the fusion of the lateral platelets, which seems to have a complex evolutionary history. For example, if this character is synapomorphic for the Rusetria Complex, then there have been multiple reversals. Furthermore, the position of the Partial 2-Plates suggests that the partial fusion of their lateral platelets represents a transitional step to complete fusion. But this story is even more complicated by *T. glomerabilis*, which has regained free lateral platelets separately from the rest of the Eastern 4-Plates. Ultimately, understanding the evolution of lateral platelet fusion, even within the eastern U.S., will depend on a much greater sampling of this worldwide species complex. Second, significant sexual size dimorphism (i.e., males 20–30% smaller than females), which is found in most members of this complex worldwide, likely arose once within the complex basally, but was subsequently and independently lost (i.e., males 5–15% smaller than females) three times: twice within the Eastern 4-Plates (once with the sister species *T. dunni* Fisher & Dowling, sp. n. and *T. pollani* Fisher & Dowling, sp. n., and once with *T. glomerabilis* Fisher & Dowling, sp. n.) and once with *T. ululata* Fisher & Dowling, sp. n.

The 23 species of the Rusetria Complex are organized into four species groups: Eastern 4-Plates, Eastern 2-Plates, Partial 2-Plates, and Western 2-Plates.

The **Eastern 2-Plate Group** (Figure 14–15) comprises 12 eastern species: *T. biscutella* Fisher & Dowling, sp. n.; *T. whitneyae* Fisher & Dowling, sp. n.; *T. microbiscutella* Fisher & Dowling, sp. n.; *T. feminellai* Fisher & Dowling, sp. n.; *T. caerulea* Fisher & Dowling, sp. n.; *T. delicatexa* Habeeb, 1955; *T. indistincta* (Marshall, 1929); *T. malarkeyorum* Fisher & Dowling, sp. n.; *T. pendula* Fisher & Dowling, sp. n.; *T. sellersorum* Fisher & Dowling, sp. n.; *T. tysoni* Fisher & Dowling, sp. n.; and *T. ululata* Fisher & Dowling, sp. n. This group is not monophyletic because of the exclusion of *T. glomerabilis* Fisher & Dowling, sp. n., which is considered an Eastern 4-Plate based on morphology. Members of this group are readily identifiable by having dorso-lateral platelets fused with the dorsal plate, thus giving the appearance of having only two plates, the anterio-medial platelets. Most members of this group are colorful (except for *T. indistincta*, which resembles Western 2-Plates) and nearly all have similar dorsal patterns that are separated into anterior and posterior portions, which are occasionally connected medially (except *T. ululata*, Figures 262–263). Members of the Eastern 2-Plate group are similar to the western counterpart (below) in having fused lateral platelets. However, the majority of eastern species are distinctly more colorful than most western species, which are either colorless or only faintly colored, and the groups exhibit non-overlapping ranges.

The **Western 2-Plate Group** (Figure 16) comprises four western species: *T. mulleni* Fisher & Dowling, sp. n.; *T. nortoni* Fisher & Dowling, sp. n.; *T. walteri* Fisher & Dowling, sp. n.; and *T. welbourni* Fisher & Dowling, sp. n. Members of this group are readily identifiable by having dorso-lateral platelets fused with the dorsal plate, thus giving the appearance of having only two plates. Although resembling Eastern 2-Plates, ranges are non-overlapping and western species are immediately identifiable by being colorless or nearly so, whereas most eastern species (except *T. indistincta*) are colorful. Besides range information, *Torrenticola indistincta* can be differentiated from Western 2-Plates by having coxal apodemes I-II not meeting posteriorly (meeting in western-two plates, usually with an accompanying medial suture).

The **Partial 2-Plate Group** (Figure 15) comprises four eastern species: *T. priapus* Fisher & Dowling, sp. n.; *T. folkertsae* Fisher & Dowling, sp. n.; *T. pulchra* Fisher & Dowling, sp. n.; and *T. magnexa* Habeeb, 1955. These species are readily distinguished from all other *Torrenticola* by having dorso-lateral platelets only partially fused to the dorsal plate. In practice, when sorting under low magnification (i.e., stereoscope), this group appears to be Eastern 2-Plates, but closer inspection will show that the lateral platelet borders are distinct, even under low magnification. This group will likely not be confused with members of the Rusetria 4-Plates (below), as the latter do not appear to have lateral plate fusion, even under low magnification.

The **Eastern 4-Plate Group** (Figure 15) comprises six eastern species: *T. dunni* Fisher & Dowling, sp. n.; *T. glomerabilis* Fisher & Dowling, sp. n.; *T. kittatinniana* Habeeb,1955; *T. pollani* Fisher & Dowling, sp. n.; *T. rufoalba* Habeeb, 1955; and *T. shubini* Fisher & Dowling, sp. n. This group is not monophyletic because *T. glomerabilis* is more closely related to Eastern 2-Plates. Members of this group can be readily differentiated from all other species of the Rusetria Complex by having anterio-lateral platelets completely free from the dorsal plate, hence “4-Plates”. All members of this group are colorful and have similar dorsal patterns that are separated into anterior and posterior portions and occasionally connected medially. Members of this group resemble the Neoanomala Group in overall appearance and coloration; however, they can be readily differentiated by having indistinct hind coxal margins (distinct in Neoanomala Group). Members of this group also resemble *T. skvarlai* in terms of overall appearance and coloration, and in having indistinct hind coxal margins; however, they can be readily differentiated by having conical, tuberculate pedipalp femoral extensions (broadly tuberculate in *T. skvarlai*).

**Tricolor Complex (Figure 7–9, 17–18)**: Molecular analyses recovered 10 species in this complex. Fresh material for molecular analysis was not available for four other species (*T. bittikoferae* Crowell, 1960; *T. cardia* Fisher & Dowling, sp. n.; *T. kringi* Fisher & Dowling, sp. n.; and *T. mohawk* Fisher & Dowling, sp. n.) that are grouped within this complex based upon morphology. In total, there are 14 species within the Tricolor Complex.

This complex is divided into eastern and western lineages. The eastern lineage contains species that are among the most distinctive of all *Torrenticola* (e.g., *T. trimaculata* Fisher, 2015; *T. dimorpha* Fisher & Dowling, sp. n.; and *T. projector* Habeeb, 1961). This complex contains one species group, the Tricolor Group, which is paraphyletic with respect to *T. projector*. This species has an elongate morphology that is unique among species of the Tricolor Complex.

The **Tricolor Group** (Figure 17–18) comprises 13 species: *T. bittikoferae* Crowell, 1960; *T. cardia* Fisher & Dowling, sp. n.; *T. kringi* Fisher & Dowling, sp. n.; *T. dimorpha* Fisher & Dowling, sp. n.; *T. mohawk* Fisher & Dowling, sp. n.; *T. hoosieri* Fisher & Dowling, sp. n.; *T. larvata* Cherri, Fisher, & Dowling, 2016; *T. olliei* Fisher & Dowling, sp. n.; *T. pearsoni* Fisher & Dowling, sp. n.; *T. projector* Habeeb, 1961; *T. sierrensis* (Marshall, 1943); *T. tricolor* Habeeb, 1957; *T. trimaculata* Fisher, 2015; and *T. unimaculata* Fisher & Dowling, sp. n. Most species are distributed in eastern North America, except for *T. sierrensis* and *T. olliei*, which are western. The most distinguishing characteristic of this group is a short, conical rostrum, which is noticeably downturned in males. The only exception is *T. kringi*, which has a conical rostrum that is longer than other members of the group. Five members of the Tricolor Group form a monophyletic subgroup and can be readily recognized by having dorsal coloration composed of one or more spots (*T. kringi*, *T. mohawk*, *T. tricolor*, *T. trimaculata*, *T. unimaculata*). The Tricolor Group also contains a morphologically enigmatic species that has strong sexual dimorphism: *T. dimorpha*. Finally, *T. bittikoferae* likely fits within this complex, but fresh material was not available to test this hypothesis.

### Biogeography

Of the 90 species discussed herein, 57 are primarily eastern (east of the 100^th^ Meridian) and 33 are western (west of the 100^th^ Meridian). Only a few species are distributed on either side of this barrier: *T. irapalpa* is widespread in the east and has populations in Saskatchewan; *T. rala* is widespread from Costa Rica to Arizona and is also found in south-central Texas; and *T. sellersorum* has a widespread, but sporadic, distribution spanning Arizona to Manitoba and Ohio to Pennsylvania. Aside from these few species, the Great Plains acts as a biogeographic barrier for most species and *Torrenticola* are not common within this region.

Much of the discrepancy between the eastern and western diversity is due to increased diversity in the Appalachians, particularly due to increased speciation within the Raptor and Rusetria Complexes, which have 17 (14 endemic) and 15 (12 endemic) species found in the Appalachians, respectively. The Southern Appalachian region is particularly diverse, with 14 endemics (*T. arktonyx*, *T. bondi*, *T. karambita*, *T. longitibia*, *T. danielleae*, *T. daemon*, *T. dentirostra*, *T. racupalpa*, *T. dunni*, *T. glomerabilis*, *T. whitneyae*, *T. microbiscutella*, *T. feminellai*, and *T. pollani*). The Northern Appalachian region contains six species known only from that area (*T. elusiva*, *T. kittatinniana*, *T. folkertsae*, *T. pendula*, *T. rufoalba*, and *T. mohawk*); however, two of these are known only from their type localities in northern New Jersey (*T. kittatinniana* and *T. rufoalba*). Eight species are restricted to the Appalachians, but range throughout both northern and southern regions (*T. erectirostra*, *T. gorti*, *T. reduncarostra*, *T. skvarlai*, *T. delicatexa*, *T. shubini*, *T. cardia*, *T. projector*). Three species appear to be widespread in the northeast (i.e., extending into the Great Lakes region in Ottawa), but also extend southward throughout the Appalachians (*T. nigroalba*, *T. raptor*, and *T. tricolor*). The remaining eastern diversity includes seven widespread species (*T. malarkeyorum*, *T. trimaculata*, *T. irapalpa*, *T. neoanomala*, *T. magnexa*, *T. priapus*, and *T. sellersorum*); seven species endemic to the Interior Highlands (*T. biscutella*, *T. interiorensis*, *T. robisoni*, *T. solisorta*, *T. larvata*, *T. pearsoni*, and *T. unimaculata*); six species from the non-mountainous southeast (*T. caerulea*, *T. elongata*, *T. flangipalpa*, *T. gnoma*, *T. tysoni*, and *T. ululata*); four species known only from the Midwest (*T. bittikoferae*, *T. indistincta*, *T. hoosieri*, and *T. pulchra*); one species from Florida (*T. ivyae*); and one species from eastern Texas (*T. kringi*).

Our results point to two eastern regions that have high diversity and an increased proportion of endemic species: the southern Appalachians (including southern Pennsylvania) and the Interior Highlands (Ozarks and Ouachitas of Missouri, Arkansas, and Oklahoma). Of the 31 species known from the southern Appalachians, 14 are endemic. Of the 13 species known from the Interior Highlands, seven are endemic. Each of these regions is well-known for its diversity and endemism of aquatic taxa, due to a complex biogeographic history where they have acted as refugia (Robison & Allen 1995; Radwell et al. 2011; Skvarla et al. 2015).

By contrast, western *Torrenticola* are less diverse, with 32 species known from the region. Four species are widespread (*T. ellipsoidalis*, *T. multiforma*, *T. sierrensis*, and *T. tahoei*), although only one of these, *T. multiforma*, is also found in the southwest. Two species are found throughout much of California (*T. nortoni* and *T. ventura*) with only *T. ventura* extending northward into southwestern Oregon. One species is known from south-central Texas (*T. dimorpha*). The remaining western diversity is distributed into patterns that roughly correspond to three major ecoregions: Rocky Mountains, Pacific Ranges, and the arid southwest. The Rockies contain three endemics (*T. occidentalis*, *T. rockyensis*, and *T. mulleni*). The Pacific Ranges of British Columbia, Washington, Oregon, and northern California contain nine endemics (*T. miniforma*, *T. oregonensis*, *T. oliveri*, *T. leviathan*, *T. pinocchio*, *T. pacificensis*, *T. regalis*, *T. welbourni*, and *T. olliei*), and two species that also range into the Sierra Nevada (*T. copipalpa* and *T. walteri*), although the latter (*T. walteri*) is also known from one locality in the Rockies of southern British Columbia. The arid southwest (southern California, Arizona, and New Mexico) contains eleven species restricted to that region in the US (*T. boettgeri*, *T. keesdavidsi*, *T. kurtvietsi*, *T. lamellipalpis*, *T. dolichodactyla*, *T. anoplopalpa*, *T. raptoroides*, *T. sharkeyi*, *T. wiedenmanni*, and *T. rala*), although five of these extend southward into Mexico and Central America (*T. boettgeri*, *T. keesdavidsi*, *T. kurtvietsi*, *T. lamellipalpis*, and *T. rala*) and one is also found in southern Texas (*T. rala*).

Our results suggest the diversity of *Torrenticola* in eastern North America can be explained by as few as three dispersal events, followed by subsequent radiations (Figure 8). Western diversity is more complex and was likely influenced from southern species extending northward and also by northern species that crossed Beringia and extended southward. However, even these results are speculative, pending worldwide sampling.

The distributions described above show the variable dispersal capabilities of different species within the genus. For instance, some species are wide-ranging, with distributions that span multiple topographic barriers (e.g., *T. irapalpa*, *T. multiforma*, and *T. ellipsoidalis*), whereas other groups are endemic to specific geographic areas and so are not able to cross such barriers (e.g., *T. pacificensis*, *T. rockyensis*, *T. arktonyx*, and *T. solisorta*). To explain this variation, adequate knowledge of the dispersal capabilities of both the adult and larval stages is required.

It seems well-understood that water mite larvae utilize the dispersal capabilities of their winged insect hosts. Indeed, it is normally inferred that dispersal is the primary function of the parasitic larvae, as is exemplified by the following passage from Proctor et al. (2015, pg. 639):

“The dominant strategic role of the larval instar in the life history of most Hydryphantoidea, Lebertioidea, Hygrobatoidea, and Arrenuroidea appears to be dispersal rather than growth. Species that have the parasitic larval stage suppressed illustrate that development of large eggs can obviate the need for larval feeding. However, the relative rarity of this phenomenon attests to the crucial role of larval dispersal in more derived water mite species.”

Consistent with this view, many lentic water mites utilize far-flying hosts such as dragonflies (e.g., *Arrenurus*), various true bugs (e.g., *Hydrachna*), and beetles (e.g., *Eylais*) (Proctor et al. 2015). However, if *Torrenticola* were utilizing such hosts, we would expect fewer instances of endemism, as well as more dispersal events across the east-west divide. Instead, all eastern *Torrenticola* diversity could be explained by as little as three dispersal events. We speculate that this pattern can be explained by *Torrenticola* larval ecology. *Torrenticola*, like most lotic water mites, parasitize nematocerous flies, especially Chironomidae. *Torrenticola* are reported from the thoraces (rarely abdomen) of three chironomid subfamilies and ten genera: Tanypodinae (1 genus), Orthocladiinae (6 genera), and Chironominae (3 genera) (Smith & Oliver 1976, 1986). In the proper habitat (clean fast-flowing streams and riffles), *Torrenticola* adults are typically far more abundant than other water mites and larvae are easily identified by possessing fused coxal plates (unlike all other Lebertioidea [Smith 1982]), which should increase both the likelihood of larvae being sampled on a host and being identified by researchers. However, larval *Torrenticola* are rarely reported. Unfortunately, *Torrenticola* larvae are not currently correlated with adults, so there is no way to link species to hosts and we can only conclude that at least some unidentified species parasitize chironomids.

This lack of knowledge has important ramifications because chironomids appear to have different inter- and intra-species natal fidelity. Although only a few studies have addressed adult chironomid dispersal (e.g., Delettre and Morvan 2000, Krosch et al. 2011), they suggest that the propensity for adult chironomids to disperse decreases when the natal stream is bordered by dense vegetation, which appears to confine them to the stream from which they emerged. This trend is not restricted to chironomids as other aquatic insects that inhabit flowing water are also unable to disperse through dense riparian vegetation (Titmus 1980, Jackson and Resh 1989, Peterson et al. 1999, Delettre and Morvan 2000). Conversely, in areas with low vegetation, chironomids have been shown to disperse further (Delettre and Morvan 2000), and under such conditions can even cross into nearby catchments (Krosch et al. 2011).

We speculate that broadly distributed water mite species (e.g., *T. ellipsoidalis*, *T. multiforma*, *T. sierrensis*, *T. tahoei*, *T. malarkeyorum*, *T. trimaculata*, *T. irapalpa*, *T. neoanomala*, *T. magnexa*, *T. priapus*, and *T. sellersorum*) disperse primarily during the larval stage by utilizing chironomids with low natal fidelity, whereas species that are confined to a smaller geographic region (e.g., *T. solisorta*, *T. larvata*, *T. pacificensis*, *T. rockyensis*, *T. arktonyx*) either utilize chironomids that have high natal fidelity, or they have lost parasitism altogether. As discussed previously, loss of parasitism is especially likely with members of the Miniforma Group, which in addition to high endemism, also possess characters that have been proposed for species that have lost parasitism, such as smaller body size and a larger female genital opening (Smith 1998). In either case (host natal fidelity or loss of parasitism), the result is that species confined to a smaller geographic region may be more dependent on the adult stage for dispersal. A sound understanding of *Torrenticola* larval ecology, including host associations, is critical to understanding the biology of the genus. Unfortunately, this area of inquiry is inhibited by our limited understanding, not only of water mites, but also chironomids, which are also understudied in North America, leaving most aspects of the biology of nearly all species involved a mystery.

To fully understand the evolution of *Torrenticola* within North America, analyses are needed that include worldwide taxon sampling and robust analyses of multiple genes, as well as a comprehensive understanding of larval ecology. To this end, we recommend the following areas of study for future investigation in North America: 1) surveys that correlate larval and adult chironomids; 2) dispersal studies on lotic chironomids; 3) surveys that correlate larval and adult water mites; and 4) surveys that investigate host specificity (i.e., identify both water mite larvae on hosts as well as the species of the hosts).

## Taxonomy

### Torrenticolidae Piersig, 1902

**Familial diagnosis.** See Fisher et al. (2015).

#### Torrenticolinae Piersig, 1902

**Subfamilial diagnosis.** See Fisher et al. (2015)

##### *Torrenticola* Piersig, 1896

**Type species.** *T. anomala* (Koch, 1837), originally *Atractides anomalus*

**Generic diagnosis.** See Fisher et al. (2015)

###### Descriptions

####### Torrenticola anoplopalpa

Taxon classificationAnimaliaTrombidiformesTorrenticolidae

Fisher & Dowling sp. n.

http://zoobank.org/F4A21077-83A3-4FD9-A0B6-F95AF29F730E

######## Material examined.

HOLOTYPE (♂): USA, New Mexico, Catron County, Glenwood; Whitewater Picnic Area, 8 km east of Rt. 180, (33°22'22"N, 108°50'50"W), 12 July 1987, by IM Smith, IMS870084.

PARATYPES (0 ♀; 0 ♂):

######## Type deposition.

Holotype (♂) deposited in the CNC.

######## Diagnosis.

*Torrenticola anoplopalpa* are similar to other members of the Rala Group (*T. boettgeri*, *T. dolichodactyla*, *T. kurtvietsi*, *T. keesdavidsi*, *T. lamellipalpis*, and *T. rala*) by being colorless, having incomplete hind coxal margins and being distributed in the southwest. *Torrenticola anoplopalpa* can be differentiated from all other Rala Group by having a more elongate subcapitulum (ventral length/width ♂ = 4.16 in *T. anoplopalpa*, 2.04–3.56 in others). Additionally, *T. anoplopalpa* can be differentiated from all other Rala Group by femur/genu (♂ 1.94 in *T. anoplopalpa*, 0.98–1.86 in others), except *T. keesdavidsi* (1.84–1.96).

######## Description.

**Female** unknown.

**Male (Figure 20)** (n = 1) (holotype only) with characters of the genus with following specifications.

**Dorsum** — (640 long; 440 wide) ellipsoid and colorless. Anterio-medial platelets (152.5 long; 60 wide). Anterio-lateral platelets (205 long; 72.5 wide) free from dorsal plate. Dgl-4 much closer to the edge of the dorsum than to the muscle scars (distance between Dgl-4 375). Dorsal plate proportions: dorsum length/width 1.45; dorsal width/distance between Dgl-4 1.17; anterio-medial platelet length/width 2.54; anterio-lateral platelet length/width 2.83; anterio-lateral/anterio-medial length 1.34.

**Gnathosoma — Subcapitulum** (322.5 long (ventral); 235 long (dorsal); 77.5 tall) elongate and colorless. Rostrum (110 long; 32.5 wide). Chelicerae (275 long) with curved fangs (40 long). Subcapitular proportions: ventral length/height 4.16; rostrum length/width 3.38. **Pedipalps** short and stocky (especially tibiae) without extensions on femora and genua. Palpomeres: trochanter (35 long); femur (77.5 long); genu (40 long); tibia (47.5 long; 17.5 wide); tarsus (12.5 long). Palpomere proportions: femur/genu 1.94; tibia/femur 0.61; tibia length/width 2.71.

**Venter** — (775 long; 510 wide) colorless. Gnathosomal bay (127.5 long; 70 wide). Cxgl-4 subapical. **Medial suture** (70 long). **Genital plates** (160 long; 120 wide). Additional measurements: Cx-1 (310 long (total); 180 long (medial)); Cx-3 (330 wide); anterior venter (270 long). Ventral proportions: gnathosomal bay length/width 1.82; anterior venter/genital field length 1.69; anterior venter length/genital field width 2.25; anterior venter/medial suture 3.86.

**Immatures** unknown.

######## Etymology.

Specific epithet (*anoplopalpa*) refers to the pedipalps, which lack tubercles on the femora and genua, an uncommon condition in *Torrenticola*, which usually have tuberculate ventral extensions (*anoplos*, G. unarmed; *palpus*, L. hand, feeler).

######## Distribution.

**Figure 19.**
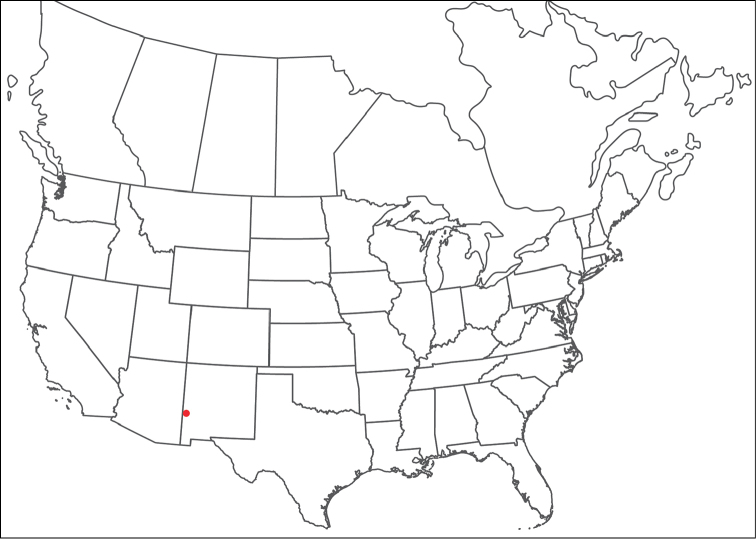
*Torrenticola anoplopalpa* sp. n. distribution.

Southwest. New Mexico (probably also Arizona) (Figure 19).

**Figure 20.**
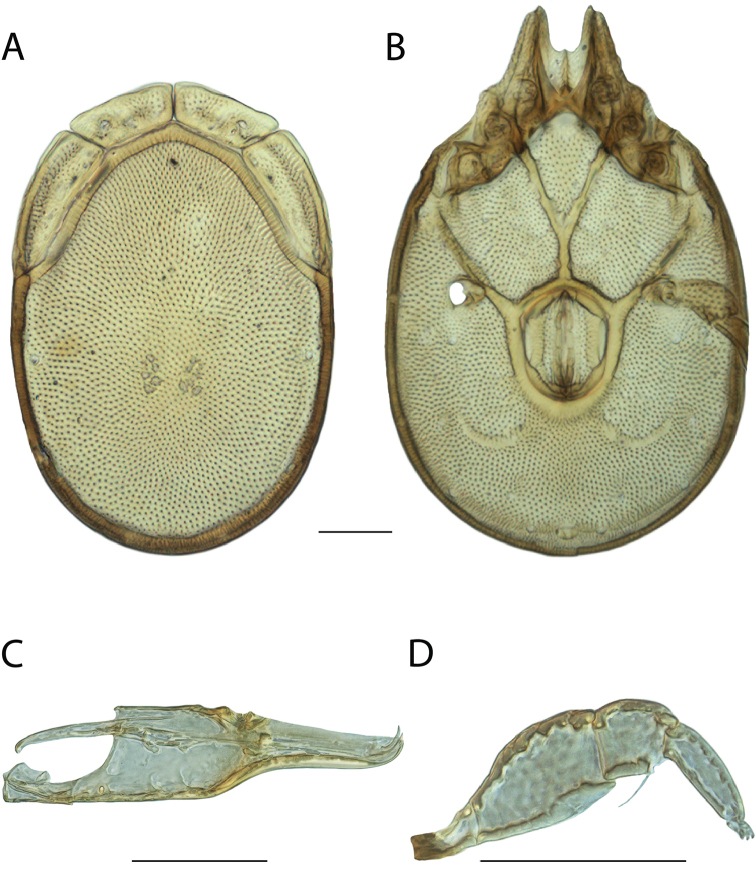
*Torrenticola anoplopalpa* sp. n. male: **A** dorsal plates **B** venter (legs removed) **C** subcapitulum **D** pedipalp (setae not accurately depicted). Scale = 100 µm.

######## Remarks.

Unfortunately, we were unable to acquire fresh material of *Torrenticola anoplopalpa* and so this species is not included in our phylogenetic analyses. However, we were able to examine material preserved in GAW for morphology. The overall appearance, incomplete hind coxal margins, lack of coloration, and distribution, are consistent with placing this species in the Rala Identification Group.

####### Torrenticola arktonyx

Taxon classificationAnimaliaTrombidiformesTorrenticolidae

Fisher & Dowling sp. n.

http://zoobank.org/4EC35C82-DF55-4F95-887C-534A4088A27C

######## Material examined.

HOLOTYPE (♀): USA, North Carolina, Macon County, Rainbow Springs; beside Forest Route 67, 2.0 km south of road to Standing Indian Campground, (35°3'3"N, 83°31'31"W), 1 July 1990, by IM Smith, IMS900072

PARATYPES (4 ♀; 5 ♂): **Georgia, USA**: 1 ♀ from White County, Helen; beside Road to Anna Ruby Falls just north of Unicoi State Park, (34°44'44"N, 83°43'43"W), 24 September 1992, by IM Smith, IMS920051 • **North Carolina, USA**: 1 ♀ from Haywood County, Great Smokey Mountains National Park, Big Creek downstream of the bridge at picnic area, (35°45'45"N, 83°6'6"W), 15 September 2009, by AJ Radwell, AJR090008A • 1 ♀ and 2 ♂ from Haywood County, Great Smoky Mountains National Park; Cataloochee; beside Mt. Sterling Rd. near bridge 1.7 km n. of road to Campground, (35°38'38"N, 83°4'4"W), 6 September 2009, by IM Smith, IMS090099 • 2 ♂ from Macon County, Rainbow Springs; beside Forest Route 67, 2.0 km south of road to Standing Indian Campground, (35°3'3"N, 83°31'31"W), 20 September 1991, by IM Smith, IMS910054 • 1 ♂ (ALLOTYPE) from Macon County, Rainbow Springs; beside Forest Route 67, 2.0 km south of road to Standing Indian Campground, (35°3'3"N, 83°31'31"W), 1 July 1990, by IM Smith, IMS900072 • **Tennessee, USA**: 1 ♀ from Blount County, Middle Prong of the Little River at Tremont, (35°38'38"N, 83°41'41"W), 16 September 2009, by AJ Radwell, AJR090009

######## Type deposition.

Holotype (♀), allotype (♂), and some paratypes (2 ♀; 2 ♂) deposited in the CNC; other paratypes (2 ♀; 2 ♂) deposited in the ACUA.

######## Diagnosis.

*Torrenticola arktonyx* are similar to species with similar dorsal patterning, such as the Rusetria “4-Plate” group (*T. dunni*, *T. glomerabilis*, *T. kittatinniana*, *T. pollani*, *T. rufoalba*, and *T. shubini*), Elongata Group (*T. elongata*, *T. gorti*, and *T. reduncarostra*), Neoanomala Group (*T. interiorensis* and *T. neoanomala*), and *T. erectirostra*, *T. robisoni*, *T. irapalpa*, *T. racupalpa*, *T. skvarlai*, and *T. bondi*. *Torrenticola arktonyx* can be differentiated from all other *Torrenticola* by having distinctive longitudinal dark markings on the anterior portion of the dorsal plate that fade posteriorly.

######## Description.

**Female (Figure 22)** (n = 4) (holotype measurements in parentheses when available) with characters of the genus with following specifications.

**Dorsum** — (645–680 (670) long; 480–520 (510) wide) ovoid with purple coloration separated into anterior and posterior portions with faint orange medially, also with distinctive longitudinal dark markings on the anterior portion of the dorsal plate that fade posteriorly. Anterio-medial platelets (137.5–145 (137.5) long; 65–70 (65) wide). Anterio-lateral platelets (200–207.5 (200) long; 80–90 (80) wide) free from dorsal plate. Dgl-4 much closer to the edge of the dorsum than to the muscle scars (distance between Dgl-4 335–375 (375)). Dorsal plate proportions: dorsum length/width 1.29–1.34 (1.31); dorsal width/distance between Dgl-4 1.36–1.43 (1.36); anterio-medial platelet length/width 2.07–2.23 (2.12); anterio-lateral platelet length/width 2.31–2.56 (2.50); anterio-lateral/anterio-medial length 1.38–1.48 (1.45).

**Gnathosoma — Subcapitulum** (345–362.5 (355) long (ventral); 260–270 (265) long (dorsal); 117.5–120 (117.5) tall) with purple coloration. Rostrum (135–135 (135) long; 45–47.5 (45) wide). Chelicerae (335–350 (350) long) with curved fangs (45–55 (45) long). Subcapitular proportions: ventral length/height 2.88–3.09 (3.02); rostrum length/width 2.84–3.00 (3.00). **Pedipalps** with dentate, flanged ventral extensions on femora and genua. Palpomeres: trochanter (40–42.5 (41.25) long); femur (107.5–111.25 (111.25) long); genu (75–80 (77.5) long); tibia (92.5–95 (95) long; 25–27.5 (25) wide); tarsus (25–25 (25) long). Palpomere proportions: femur/genu 1.38–1.44 (1.44); tibia/femur 0.85–0.86 (0.85); tibia length/width 3.36–3.80 (3.80).

**Venter** — (815–840 (815) long; 510–600 (600) wide) with purple coloration. Gnathosomal bay (137.5–155 (137.5) long; 82.5–92.5 (92.5) wide). Cxgl-4 subapical. **Medial suture** (50–60 (50) long). **Genital plates** (180–187.5 (180) long; 155–162.5 (155) wide). Additional measurements: Cx-1 (300–310 (300) long (total); 150–165 (165) long (medial)); Cx-3 (350–385 (385) wide); anterior venter (230–232.5 (230) long). Ventral proportions: gnathosomal bay length/width 1.49–1.82 (1.49); anterior venter/genital field length 1.24–1.28 (1.28); anterior venter length/genital field width 1.42–1.48 (1.48); anterior venter/medial suture 3.83–4.60 (4.60).

**Male (Figure 23)** (n = 5) (allotypic measurements in parentheses when available) with characters of the genus with following specifications.

**Dorsum** — (500–570 (570) long; 400–450 (450) wide) ovoid with purple coloration separated into anterior and posterior portions with faint orange medially, also with distinctive longitudinal dark markings on the anterior portion of the dorsal plate that fade posteriorly. Anterio-medial platelets (110–125 (125) long; 55–62.5 (62.5) wide). Anterio-lateral platelets (155–180 (180) long; 65–75 (75) wide) free from dorsal plate. Dgl-4 much closer to the edge of the dorsum than to the muscle scars (distance between Dgl-4 305–345 (345)). Dorsal plate proportions: dorsum length/width 1.23–1.35 (1.27); dorsal width/distance between Dgl-4 1.30–1.35 (1.30); anterio-medial platelet length/width 2.00–2.18 (2.00); anterio-lateral platelet length/width 2.21–2.69 (2.40); anterio-lateral/anterio-medial length 1.41–1.59 (1.44).

**Gnathosoma — Subcapitulum** (275–300 (300) long (ventral); 210–235 (235) long (dorsal); 92.5–102.5 (102.5) tall) with purple coloration. Rostrum (110–125 (125) long; 35–40 (40) wide). Chelicerae (270–305 (305) long) with curved fangs (40–45 (40) long). Subcapitular proportions: ventral length/height 2.75–3.08 (2.93); rostrum length/width 3.00–3.20 (3.13). **Pedipalps** with dentate, flanged ventral extensions on femora and genua. Palpomeres: trochanter (32.5–37.5 (35) long); femur (85–97.5 (97.5) long); genu (60–68.75 (65) long); tibia (75–85 (85) long; 22.5–26.25 (25) wide); tarsus (21.25–25 (22.5) long). Palpomere proportions: femur/genu 1.42–1.50 (1.50); tibia/femur 0.81–0.91 (0.87); tibia length/width 3.10–3.40 (3.40).

**Venter** — (650–725 (725) long; 430–490 (490) wide) with purple coloration. Gnathosomal bay (125–135 (135) long; 65–72.5 (72.5) wide). Cxgl-4 subapical. **Medial suture** (75–90 (80) long). **Genital plates** (160–172.5 (172.5) long; 117.5–130 (130) wide). Additional measurements: Cx-1 (250–290 (290) long (total); 130–150 (145) long (medial)); Cx-3 (295–350 (350) wide); anterior venter (220–260 (260) long). Ventral proportions: gnathosomal bay length/width 1.85–2.08 (1.86); anterior venter/genital field length 1.38–1.55 (1.51); anterior venter length/genital field width 1.87–2.17 (2.00); anterior venter/medial suture 2.72–3.25 (3.25).

**Immatures** unknown.

######## Etymology.

Specific epithet (*arktonyx*) refers to the distinctive longitudinal markings on the anterior dorsal plate, which resemble claw marks from a bear (*árktos*, G. bear; *ónyx*, G. claw).

######## Distribution.

**Figure 21.**
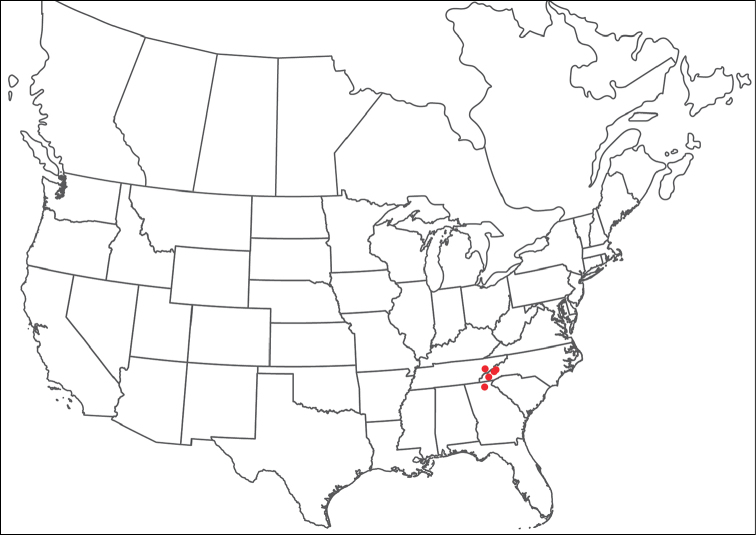
*Torrenticola arktonyx* sp. n. distribution.

Southern Appalachians (Figure 21).

**Figure 22.**
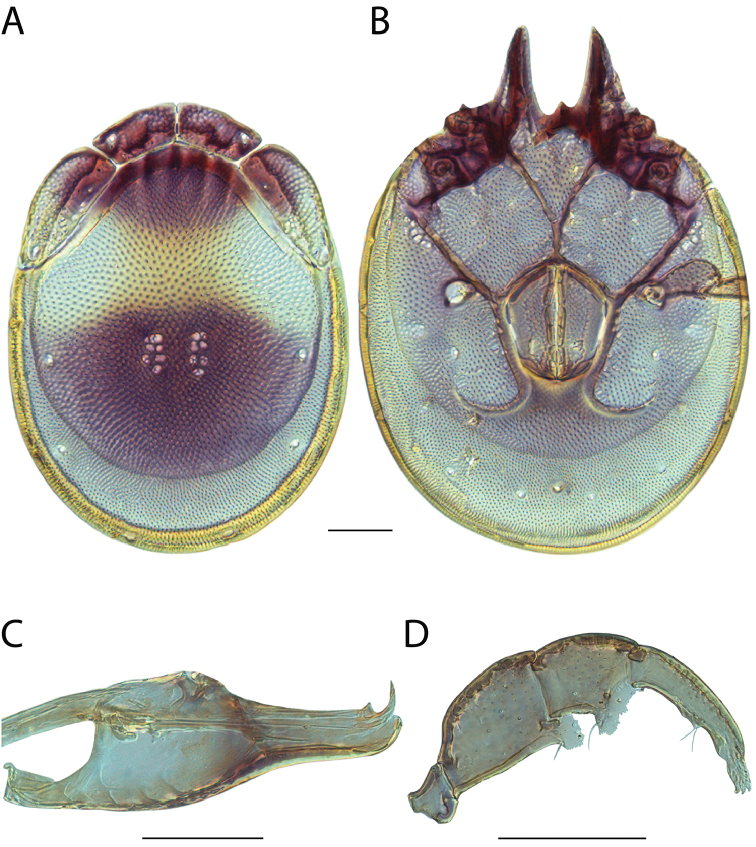
*Torrenticola arktonyx* sp. n. female: **A** dorsal plates **B** venter (legs removed) **C** subcapitulum **D** pedipalp (setae not accurately depicted). Scale = 100 µm.

**Figure 23.**
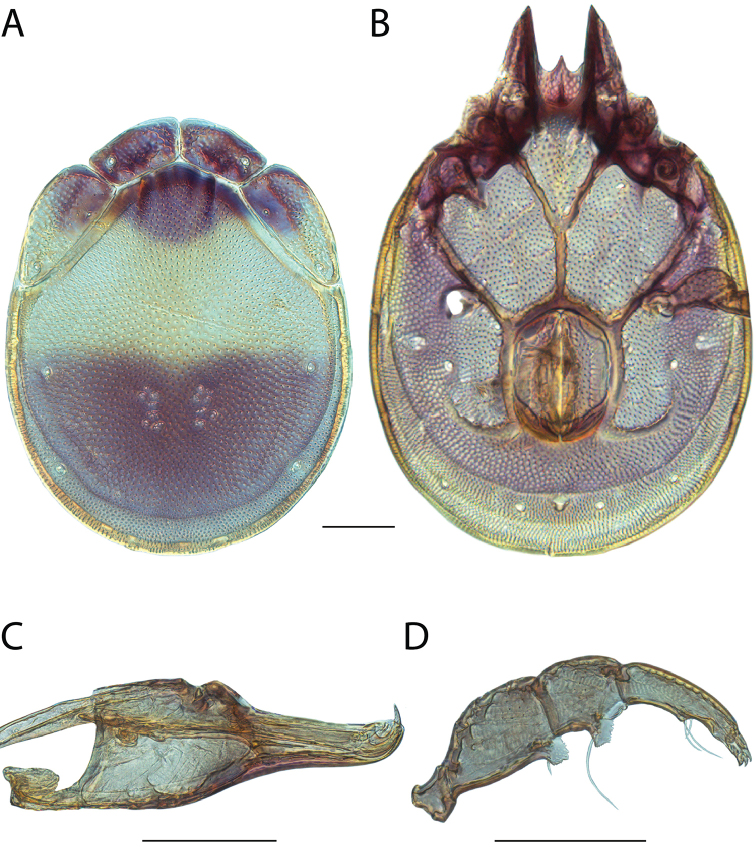
*Torrenticola arktonyx* sp. n. male: **A** dorsal plates **B** venter (legs removed) **C** subcapitulum **D** pedipalp (setae not accurately depicted). Scale = 100 µm.

######## Remarks.

Unfortunately, we were unable to acquire fresh material of *Torrenticola arktonyx* and therefore this species is not included in our phylogenetic analyses. We were able to examine material preserved in GAW for morphology, but due its unique characteristics, we are unable to place this species into either a species complex or identification group. However, based upon coloration, distribution, and gnathosomal shape, we speculate that future analyses will place this species in the Raptor Complex.

####### Torrenticola biscutella

Taxon classificationAnimaliaTrombidiformesTorrenticolidae

Fisher & Dowling sp. n.

http://zoobank.org/F45BF84D-35BF-4498-A610-0EB69342AF30

######## Material examined.

HOLOTYPE (♀): from USA, Arkansas, Montgomery County, South Fork Ouachita River, access off County Road 17 at Forest Road 903, 29 Jul 2011, by IM Smith, IMS110040, DNA 1263.

PARATYPES (4 ♀; 3 ♂): **Arkansas, USA**: 1 ♂ (ALLOTYPE) from Montgomery County, Ouachita River (34°34'53.20"N, 93°53'0.16"W), 5 Oct 2007, by AJ Radwell, & HW Robison, AJR070300A • 2 ♂ Montgomery County, Ouachita River (34°34'53.20"N, 93°53'0.16"W), 5 Oct 2007, by AJ Radwell, & HW Robison, AJR070300A • 3 ♀ from Montgomery County, South Fork Ouachita River, access off County Road 17 at Forest Road 903, 29 Jul 2011, by IM Smith, IMS110040 • **Missouri, USA**: 1 ♀ from Crawford County, Huzzah Creek, Red Bluff campground, off Road V east of Davisville, 23 Jul 2011, by IM Smith, IMS110029.

######## Type deposition.

Holotype (♀), allotype (♂), and some paratypes (2 ♀; 1 ♂) deposited in the CNC; other paratypes (2 ♀; 1 ♂) deposited in the ACUA.

######## Diagnosis.

*Torrenticola biscutella* are similar to other members of the Rusetria “Eastern 2-Plates” group (*T. caerulea*, *T. delicatexa*, *T. feminellai*, *T. indistincta*, *T. malarkeyorum*, *T. microbiscutella*, *T. pendula*, *T. sellersorum*, *T. tysoni*, *T. ululata*, and *T. whitneyae*) in having anterio-lateral platelets fused to the dorsal plate, having dorsal coloration separated into anterior and posterior portions (except *T. indistincta* and *T. ululata*), and being distributed in the east. It is one of only four Eastern 2-Plates that have dark, bold, bluish-purple coloration (also *T. pendula*, *T. sellersorum*, and *T. tysoni*). *Torrenticola biscutella* can be differentiated from *T. caerulea*, *T. ululata*, *T. indistincta*, and *T. feminellai* by dorsal coloration and pattern. *T. biscutella* can be differentiated from *T. tysoni* by having a stockier rostrum (length/width = 2.55–2.83 in *T. biscutella*, 3.06–3.50 in *T. tysoni*). Female *T. biscutella* can be differentiated from female *T. malarkeyorum* by having a shorter subcapitulum (ventral length = 290–315 in *T. biscutella*, 317.5–335 in *T. malarkeyorum*). Male *T. biscutella* can be differentiated from male *T. malarkeyorum* by having a slightly rounder dorsum (length/width 1.37–1.42 in *T. biscutella*, 1.42–1.56 in *T. malarkeyorum*). Additionally, although *T. biscutella* and *T. malarkeyorum* have the same dorsal coloration and pattern, often the coloration is bold in *T. biscutella* and faint in *T. malarkeyorum*. Female *T. biscutella* can be differentiated from female *T. delicatexa* by having a shorter genital field (152.5–167.5 in *T. biscutella*, 175–198 in *T. delicatexa*) and male *T. biscutella* can be differentiated from male *T. delicatexa* by having a slightly rounder dorsum (length/width = 1.37–1.42 in *T. biscutella*, 1.44–1.56 in *T. delicatexa*). Female *T. biscutella* can be differentiated from female *T. sellersorum* by anterior venter/genital field length (0.82–0.88 in *T. biscutella*, 0.69–0.77 in *T. sellersorum*). Male *T. biscutella* can be differentiated from male *T. sellersorum* by having slightly stockier anterio-lateral platelets (length/width = 2.58–2.74 in *T. biscutella*, 2.76–3.00 in *T. sellersorum*). *T. biscutella* can be differentiated from *T. pendula* by having a stockier gnathosomal bay (1.55–1.85 in *T. biscutella*, 2.42–2.9 in *T. pendula*); more elongate tibiae (3.11–3.45 in *T. biscutella*, 2.78–3.05 in *T. biscutella*); and by dorsal pattern. *T. biscutella* can be differentiated from *T. microbiscutella* by having a more ovoid dorsum (length/width = 1.33–1.42 in *T. biscutella*, 1.63–1.75 in *T. microbiscutella*) and by anterior venter/genital field width (♀ = 0.84–0.91 in *T. biscutella*, 1.25–1.33 in *T. microbiscutella*; ♂ = 1.68–1.80 in *T. biscutella* and 1.95–2.29 in *T. microbiscutella*). *T. biscutella* can be differentiated from *T. whitneyae* by having more elongate pedipalpal tibiae (3.11–3.45 in *T. biscutella*, 2.42–2.95 in *T. whitneyae*) and by anterior venter/genital field length (♀ = 0.82–0.88 in *T. biscutella*, 0.59–0.75 in *T. whitneyae*; ♂ = 1.55–1.76 in *T. biscutella* and 1.37–1.43 in *T. whitneyae*).

######## Description.

**Female (Figure 25)** (n = 4) (holotype measurements in parentheses when available) with characters of the genus with following specifications.

**Dorsum** — (560–630 (560) long; 420–455 (420) wide) ovoid with bluish-purple coloration separated into anterior and posterior portions, and bold or faint orange medially. Anterio-medial platelets (122.5–135 (122.5) long; 40–45 (40) wide). Anterio-lateral platelets (140–170 (140) long; 62.5–75 (62.5) wide) fused to dorsal plate. Dgl-4 much closer to the edge of the dorsum than to the muscle scars (distance between Dgl-4 300–330 (300)). Dorsal plate proportions: dorsum length/width 1.33–1.38 (1.33); dorsal width/distance between Dgl-4 1.38–1.40 (1.40); anterio-medial platelet length/width 3.00–3.31 (3.06); anterio-lateral platelet length/width 2.24–2.48 (2.24); anterio-lateral/anterio-medial length 1.14–1.28 (1.14).

**Gnathosoma — Subcapitulum** (290–315 (290) long (ventral); 207–240 (208) long (dorsal); 137.5–155 (137.5) tall) colorless. Rostrum (110–125 (110) long; 42.5–47.5 (42.5) wide). Chelicerae (286–335 (286) long) with curved fangs (55–70 (56) long). Subcapitular proportions: ventral length/height 2.02–2.11 (2.11); rostrum length/width 2.56–2.67 (2.59). **Pedipalps** with tuberculate ventral extensions on femora and genua. Palpomeres: trochanter (43.75–50 (43.75) long); femur (107.5–122.5 (107.5) long); genu (65–72.5 (65) long); tibia (80–86.25 (80) long; 23.75–25 (23.75) wide); tarsus (20–20 (20) long). Palpomere proportions: femur/genu 1.59–1.74 (1.65); tibia/femur 0.69–0.74 (0.74); tibia length/width 3.35–3.45 (3.37).

**Venter** — (660–740 (660) long; 488–544 (489) wide) with faint bluish-purple coloration. Gnathosomal bay (151.25–172.5 (151.25) long; 97.5–100 (100) wide). Cxgl-4 subapical. **Medial suture** absent. **Genital plates** (152.5–167.5 (152.5) long; 142.5–160 (142.5) wide). Additional measurements: Cx-1 (274–309 (275) long (total); 118–135 (121) long (medial)); Cx-3 (319–392 (319) wide); anterior venter (130–147.5 (130) long). Ventral proportions: gnathosomal bay length/width 1.55–1.73 (1.55); anterior venter/genital field length 0.82–0.88 (0.85); anterior venter length/genital field width 0.84–0.91 (0.91).

**Male (Figure 26)** (n = 3) (allotypic measurements in parentheses when available) with characters of the genus with following specifications.

**Dorsum** — (430–445 (440) long; 310–315 (310) wide) ovoid with bluish-purple coloration separated into anterior and posterior portions, and bold or faint orange medially. Anterio-medial platelets (97.5–97.5 (97.5) long; 33.75–36.25 (35) wide). Anterio-lateral platelets (122.5–130 (130) long; 45–50 (47.5) wide) fused to dorsal plate. Dgl-4 much closer to the edge of the dorsum than to the muscle scars (distance between Dgl-4 230–242.5 (230)). Dorsal plate proportions: dorsum length/width 1.37–1.42 (1.42); dorsal width/distance between Dgl-4 1.30–1.35 (1.35); anterio-medial platelet length/width 2.69–2.89 (2.79); anterio-lateral platelet length/width 2.58–2.74 (2.74); anterio-lateral/anterio-medial length 1.26–1.33 (1.33).

**Gnathosoma — Subcapitulum** (230–235 (235) long (ventral); 175–177.5 (177) long (dorsal); 20–20 (20) tall) colorless. Rostrum (85–92.5 (92.5) long; 30–36.25 (36.25) wide). Chelicerae (225–241 (241) long) with curved fangs (45–50 (46) long). Subcapitular proportions: ventral length/height 2.29–2.47 (2.29); rostrum length/width 2.55–2.83 (2.55). **Pedipalps** with tuberculate ventral extensions on femora and genua. Palpomeres: trochanter (23.75–37.5 (37.5) long); femur (85–90 (90) long); genu (52.5–55 (55) long); tibia (68.75–72.5 (72.5) long; 21.25–22.5 (22.5) wide); tarsus (17.5–20 (20) long). Palpomere proportions: femur/genu 1.62–1.64 (1.64); tibia/femur 0.81–0.82 (0.81); tibia length/width 3.11–3.24 (3.22).

**Venter** — (510–525 (525) long; 335–380 (336) wide) with faint bluish-purple coloration. Gnathosomal bay (115–122.5 (122.5) long; 65–67.5 (67.5) wide). Cxgl-4 subapical. **Medial suture** (60–65 (65) long). **Genital plates** (102.5–110 (102.5) long; 100–100 (100) wide). Additional measurements: Cx-1 (215–226 (226) long (total); 99–110 (100) long (medial)); Cx-3 (252–275 (252) wide); anterior venter (167.5–180 (180) long). Ventral proportions: gnathosomal bay length/width 1.70–1.85 (1.81); anterior venter/genital field length 1.55–1.76 (1.76); anterior venter length/genital field width 1.68–1.80 (1.80); anterior venter/medial suture 2.68–2.83 (2.77).

**Immatures** unknown.

######## Etymology.

Specific epithet (*biscutella*) refers to the appearance of only two anterio-dorsal platelets due to the fusion of lateral platelets with the dorsal shield (*bi*-, L. two; *scutella*, L. little plate).

######## Distribution.

**Figure 24.**
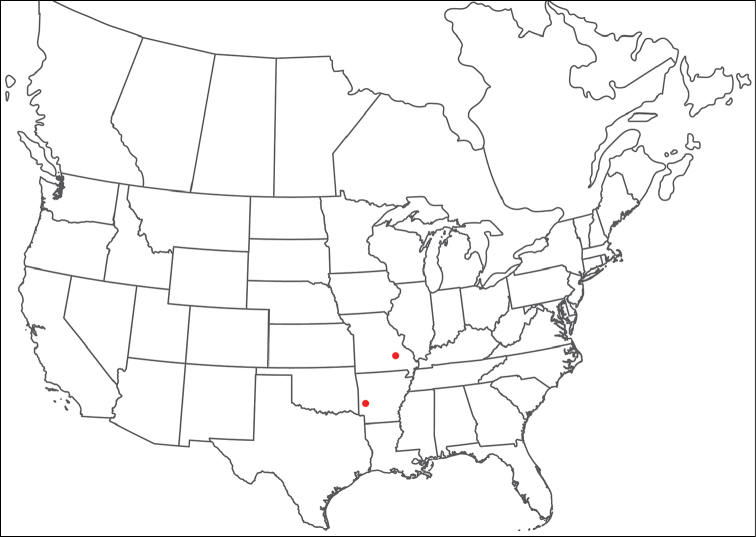
*Torrenticola biscutella* sp. n. distribution.

Interior Highlands (both Ozarks and Ouachitas), likely endemic (Figure 24).

**Figure 25.**
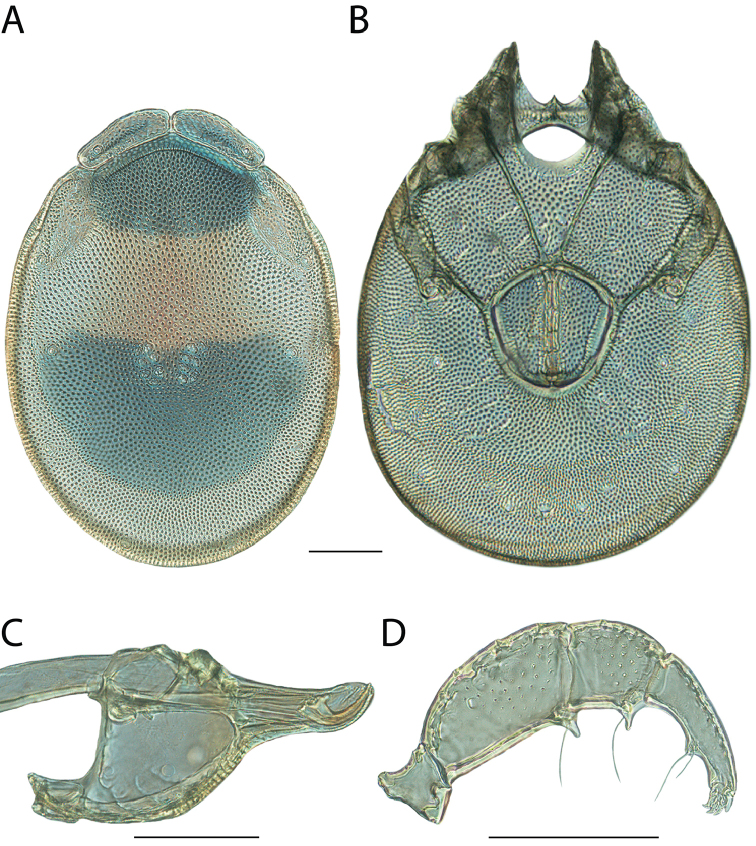
*Torrenticola biscutella* sp. n. female: **A** dorsal plates **B** venter (legs removed) **C** subcapitulum **D** pedipalp (setae not accurately depicted). Scale = 100 µm.

**Figure 26.**
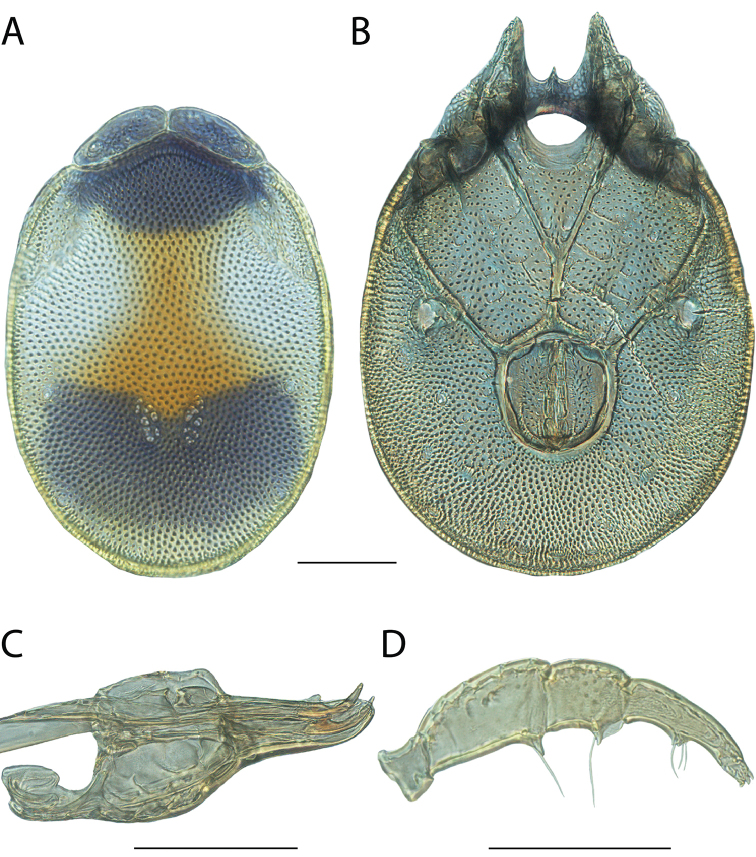
*Torrenticola biscutella* sp. n. male: **A** dorsal plates **B** venter (legs removed) **C** subcapitulum **D** pedipalp (setae not accurately depicted). Scale = 100 µm.

######## Remarks.

*Torrenticola biscutella* groups with other members of the Rusetria Complex with high support and specimens of this species were less than 2% different in COI sequence from each other. In all analyses, *T. biscutella* groups with two other morphologically similar species: *T. malarkeyorum* and *T. caerulea*. These three species are 3–5% different from each other in COI sequence. The three of these species are morphologically similar to the more distantly-related *T. delicatexa*. Of these four species, the range of *T. biscutella* only overlaps with *T. malarkeyorum* in the Ozark Mountains and these species are easily differentiated by color. *T. biscutella* is the only one of these four species known from the Ouachita Mountains, and it is not known from east of the Mississippi River, where the other three species are distributed (only *T. malarkeyorum* is known from west of the Mississippi River).

Based upon overall similarity, dorso-lateral platelet fusion, and distribution, we were able to place this species within the Eastern 2-Plate Identification Group.

This species hypothesis is supported by biogeography, low COI divergence within the species (0–2%) and high divergence between species (3–15%), and by morphological characters outlined in the diagnoses.

####### Torrenticola bittikoferae

Taxon classificationAnimaliaTrombidiformesTorrenticolidae

Crowell, 1960

T. bittikoferae : Crowell 1960: 36; 1961: 330 • Johnston 1965: 44 • Modlin and Gannon 1973: 219, 221 • Viets 1987: 756.

######## Material examined.

PARATYPES (0 ♀; 2 ♂): **Ohio, USA**: 2 ♂ from Ottawa County, Middle Bass Island, rubble beach, 29 June 1954, by R Crowell.

######## Type deposition.

Holotype (♀) and some paratypes (unspecified number) deposited in the Chicago Natural History Museum (unexamined; types not located); other paratypes (2 ♂) deposited in the OSUAC.

######## Diagnosis.

*Torrenticola bittikoferae* are similar to other members of the Tricolor Group (*T. cardia*, *T. dimorpha*, *T. hoosieri*, *T. kringi*, *T. larvata*, *T. mohawk*, *T. olliei*, *T. pearsoni*, *T. sierrensis*, *T. tricolor*, *T. trimaculata*, and *T. unimaculata*) in having a short, conical rostrum. *Torrenticola bittikoferae* can be differentiated from most other Tricolor Complex (except *T. hoosieri*, *T. pearsoni*, and *T. dimorpha*) by being colorless, whereas most other members have bold patterning. *T. bittikoferae* can be differentiated from *T. hoosieri* by having ventral extensions on the pedipalp femora and genua (lacking in *T. hoosieri*) and having stockier pedipalp tibiae (length/width = 2.7–2.8 in *T. bittikoferae*, 3.6–4.4 in *T. hoosieri*). *T. bittikoferae* can be differentiated from *T. pearsoni* by having Dgl-4 further from the dorsal edge (dorsal width/distance between Dgl-4 = 1.6–1.7 in *T. bittikoferae*, 1.2–1.3 in *T. pearsoni*); stockier pedipalp tibiae (length/width = 2.7–2.8 in *T. bittikoferae*, 3.0–3.3 in *T. pearsoni*); and a more elongate rostrum (length/width = 1.8–1.9 in *T. bittikoferae*, 2.0–2.4 in *T. pearsoni*). *Torrenticola bittikoferae* can be differentiated from *T. dimorpha* by having an unmodified dorsal plate (*T. dimorpha* has a dorsal plate medial extension covering nearly half the length of the anterio-medial platelets) and by males having unmodified pedipalps (male *T. dimorpha* have large, highly modified pedipalps which are expanded vertically and laterally).

######## Re-description.

**Male (Figure 28)** (n = 2) with characters of the genus with following specifications.

**Dorsum** — (620–670 long; 500–530 wide) circular and colorless. Anterio-medial platelets (132.5–137.5 long; 70–70 wide). Anterio-lateral platelets (192.5–202.5 long; 90–92.5 wide) free from dorsal plate. Dgl-4 approaching midway between muscle scars and dorsum edge (distance between Dgl-4 305–330). Dorsal plate proportions: dorsum length/width 1.24–1.26; dorsal width/distance between Dgl-4 1.61–1.64; anterio-medial platelet length/width 1.89–1.96; anterio-lateral platelet length/width 2.14–2.19; anterio-lateral/anterio-medial length 1.40–1.53.

**Gnathosoma — Subcapitulum** (265 long (ventral); 202.5 long (dorsal); 125 tall) colorless. Rostrum (95–100 long; 52.5–52.5 wide). Chelicerae (260 long) with curved fangs (50 long) short and conical. Subcapitular proportions: ventral length/height 2.12; rostrum length/width 1.81–1.90. **Pedipalps** with tuberculate ventral extensions with dentate tip on femora and tuberculate ventral extensions on genua. Palpomeres: trochanter (42.5–42.5 long); femur (101.25–107.5 long); genu (72.5–75 long); tibia (87.5–90 long; 32.5–32.5 wide); tarsus (25–35 long). Palpomere proportions: femur/genu 1.40–1.43; tibia/femur 0.81–0.89; tibia length/width 2.69–2.77.

**Venter** — (790–800 long; 610–680 wide) colorless. Gnathosomal bay (122.5–125 long; 87.5–100 wide). **Medial suture** (102.5–117.5 long). **Genital plates** (137.5–142.5 long; 115–115 wide). Additional measurements: Cx-1 (270–280 long (total); 152.5–152.5 long (medial)); Cx-3 (405–410 wide); anterior venter (270–287.5 long). Ventral proportions: gnathosomal bay length/width 1.23–1.43; anterior venter/genital field length 1.96–2.02; anterior venter length/genital field width 2.35–2.50; anterior venter/medial suture 102.5–117.5.

**Female** type specimens unavailable for present study.

**Immatures** unknown.

######## Etymology.

Robert Crowell (1960) named the specific epithet (*bittikoferae*) after Lelia Bittikofer, his high school biology teacher.

######## Distribution.

**Figure 27.**
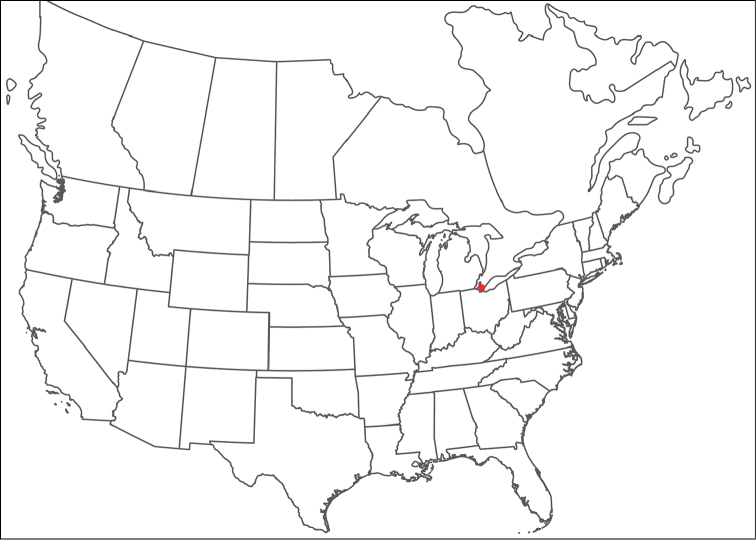
*Torrenticola bittikoferae* distribution.

Known only from type locality: Lake Erie, Ohio (Figure 27).

**Figure 28.**
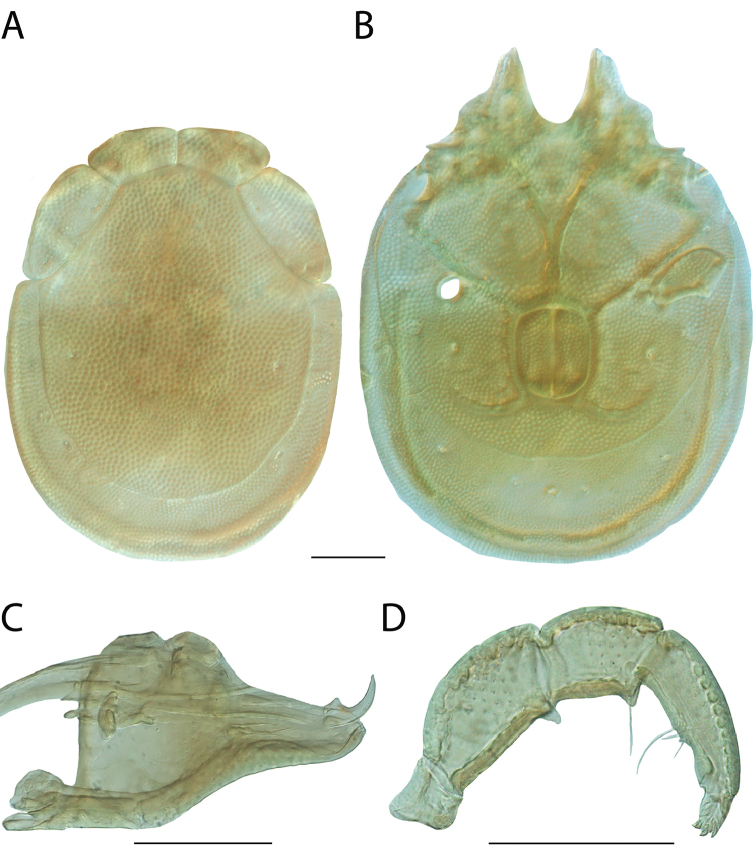
*Torrenticola bittikoferae* male: **A** dorsal plates **B** venter (legs removed) **C** subcapitulum **D** pedipalp (setae not accurately depicted). Scale = 100 µm.

######## Remarks.

Unfortunately, we were unable to acquire fresh specimens of *Torrenticola bittikoferae* and therefore this species was not included in our phylogenetic analyses. We were able to examine paratypes of two males, neither of which were dissected during slide preparation, making precise examination difficult. These specimens were remounted, but proper dissection risked fully damaging the specimen and was therefore avoided. The images in Figure 28A–B that appear to display a properly dissected specimen, were created by photographing each section (dorsum and venter) of the fully intact specimen and digitally editing the photographs so that the dorsum and venter could be easily compared with other species.

The overall appearance, short conical rostrum, and distribution of this species allows us to places it within the Tricolor Complex and Tricolor Identification Group.

####### Torrenticola boettgeri

Taxon classificationAnimaliaTrombidiformesTorrenticolidae

K.O. Viets, 1977

Torrenticola boettgeri K.O. Viets, 1977a: 89.
Torrenticola esbelta Cramer, 1992: 22.

######## Material examined

**(1** ♀; **4** ♂) **. New Mexico, USA**: 1 ♀ and 1 ♂ from Catron County, Glenwood; Whitewater Picnic Area 8 km east of Rt. 180, (33°22'22"N, 108°50'50"W), 12 July 1987, by IM Smith, IMS870084 • 3 ♂ from Catron County, beside Rt. 15, 65 km north of Rt. 180, Silver City, (33°12'12"N, 108°13'13"W), 10 July 1987, by IM Smith, IMS870081A

######## Type deposition.

Holotype (♀), prep. no. 6381 SMF, Viets collection (not examined).

######## Diagnosis.

*Torrenticola boettgeri* are similar to other members of the Rala Group (*T. anoplopalpa*, *T. dolichodactyla*, *T. keesdavidsi*, *T. kurtvietsi*, *T. lamellipalpis*, and *T. rala*) by being colorless, having incomplete hind coxal margins and being distributed in the southwest. *T. boettgeri* can be differentiated from all other Rala Group by having a more elongate dorsum (length/width ♀ = 1.74–1.82 in *T. boettgeri*, 1.21–1.60 in others) and a stockier subcapitulum (ventral length/width = 1.96 in *T. boettgeri*, 2.06–3.52 in others; ♂ = 2.04–2.07 in *T. boettgeri*, 2.14–4.16 in others).

######## Re-description.

**Female (Figure 30)** (n = 3: one specimen examined from New Mexico; measurements from two additional specimens are included based upon those listed in Goldschmidt (2007) for K.O. Viets’s (1977a) specimen from Guatemala and Cramer’s (1992) specimen from Mexico) with characters of the genus with following specifications. Note: measurements below are from the above three combined sources; those in parentheses are from the Guatemalan holotype (Viets 1977a) as listed in Goldschmidt (2007).

**Dorsum** — (668–800 (675) long; 367–440 (440) wide) ovoid, elongate, and colorless. Anterio-medial platelets (103–135 (126) long; 38–52.5 wide). Anterio-lateral platelets (179–200 (199) long; 47–70 wide) free from dorsal plate. Dgl-4 much closer to the edge of the dorsum than to the muscle scars (distance between Dgl-4 370). Dorsal plate proportions: dorsum length/width 1.53–1.82 (1.82); dorsal width/distance between Dgl-4 1.19; anterio-medial platelet length/width 2.57–2.71; anterio-lateral platelet length/width 2.86–3.81; anterio-lateral/anterio-medial length 1.48–1.74 (1.58).

**Gnathosoma — Subcapitulum** (245–280 (245) long (ventral); 185 long (dorsal); 142.5 tall) colorless. Rostrum (70–87.5 (70) long; 40 wide). Chelicerae with curved fangs (65 long). Subcapitular proportions: ventral length/height 1.96; rostrum length/width 2.19. **Pedipalps** short and stocky (especially tibiae) without extensions on femora and genua. Palpomeres: trochanter (32.5 long); femur (76.25 long); genu (65 long); tibia (32.5 long; 17.5 wide); tarsus (15 long). Palpomere proportions: femur/genu 1.17; tibia/femur 0.43; tibia length/width 1.86.

**Venter** — (668–955 (845) long; 452–570 (500) wide) colorless. Gnathosomal bay (164–200 (164) long; 45–50 (47) wide). Cxgl-4 apical. **Medial suture** (65–73 (73) long). **Genital plates** (170–188 (170) long; 148–160 (148) wide). Additional measurements: Cx-1 (340 long (total); 133–135 (133) long (medial)); Cx-3 (350 wide); anterior venter (212.5 long). Ventral proportions: gnathosomal bay length/width 3.49–4.44 (3.49); anterior venter/genital field length 1.15; anterior venter length/genital field width 1.33; anterior venter/medial suture 3.27.

**Male (Figure 31)** (n = 4: new specimens from New Mexico)

**Dorsum** — (710–780 long; 400–430 wide) ovoid, elongate, and colorless. Anterio-medial platelets (122.5–140 long; 45–50 wide). Anterio-lateral platelets (175–192.5 long; 55–62.5 wide) free from dorsal plate. Dgl-4 much closer to the edge of the dorsum than to the muscle scars (distance between Dgl-4 335–365). Dorsal plate proportions: dorsum length/width 1.74–1.81; dorsal width/distance between Dgl-4 1.16–1.19; anterio-medial platelet length/width 2.68–2.95; anterio-lateral platelet length/width 2.96–3.27 (3.08); anterio-lateral/anterio-medial length 1.25–1.47.

**Gnathosoma — Subcapitulum** (255–285 long (ventral); 165–187.5 long (dorsal); 125–137.5 tall) colorless. Rostrum (80–92.5 long; 35–40 wide). Chelicerae (285–310 long) with curved fangs (62.5–65 long). Subcapitular proportions: ventral length/height 2.04–2.07; rostrum length/width 2.13–2.36. **Pedipalps** short and stocky (especially tibiae) without extensions on femora and genua. Palpomeres: trochanter (30–31.25 long); femur (58.75–72.5 long); genu (60–62.5 long); tibia (32.5–37.5 long; 17.5–17.5 wide); tarsus (15–15 long). Palpomere proportions: femur/genu 0.98–1.16; tibia/femur 0.48–0.55; tibia length/width 1.86–2.14.

**Venter** — (800–960 long; 485–510 wide) colorless. Gnathosomal bay (192.5–205 long; 40–50 wide). Cxgl-4 apical. **Medial suture** (105–125 long). **Genital plates** (160–177.5 long; 120–125 wide). Additional measurements: Cx-1 (305–330 long (total); 115–130 long (medial)); Cx-3 (320–325 wide); anterior venter (235–262.5 long). Ventral proportions: gnathosomal bay length/width 3.85–5.00; anterior venter/genital field length 1.38–1.59; anterior venter length/genital field width 1.96–2.13; anterior venter/medial suture 2.00–2.24.

**Immatures** unknown.

######## Etymology.

Viets (1977a) named the specific epithet (*boettgeri*) in honor of Klaus Böttger of the University of Kiel, Germany, who collected the type specimen in Rio Chilax near Cobán, Guatemala.

######## Distribution.

**Figure 29.**
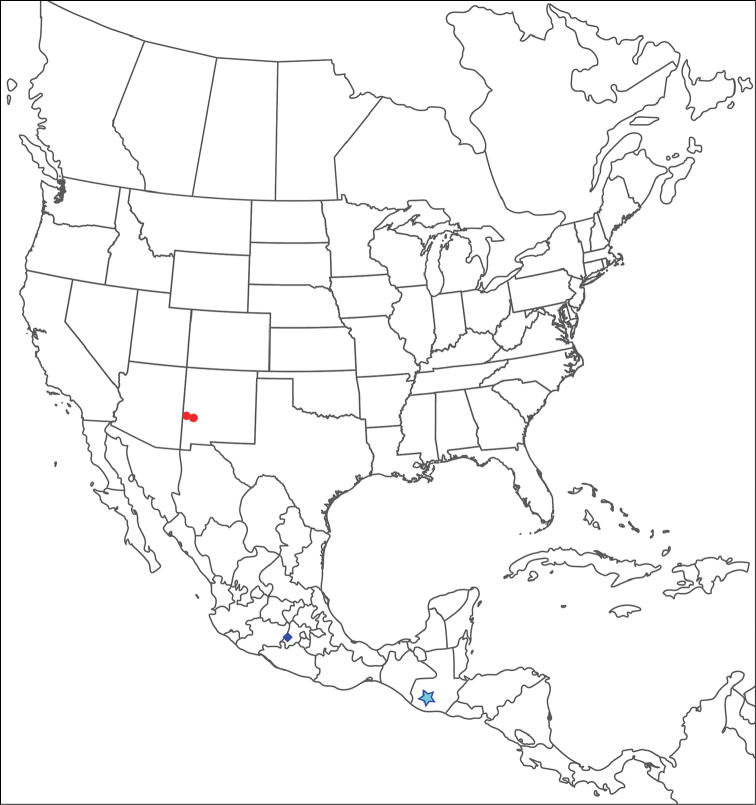
*Torrenticola boettgeri* distribution. Blue star represents type locality (Viets 1977a); blue diamond represents additional published record (Cramer 1992, as *T. esbelta* comb. n.); and red circles represent new records and material examined.

New Mexico (probably also Arizona) and extending southward into Mexico and Guatemala (Figure 29).

**Figure 30.**
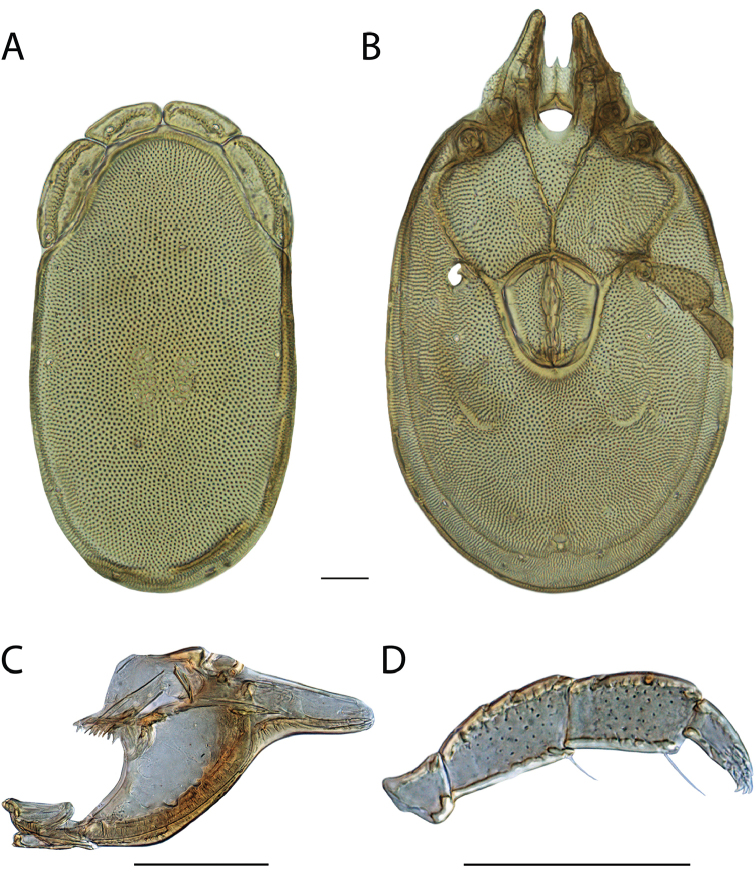
*Torrenticola boettgeri* female: **A** dorsal plates **B** venter (legs removed) **C** subcapitulum **D** pedipalp (setae not accurately depicted). Scale = 100 µm.

**Figure 31.**
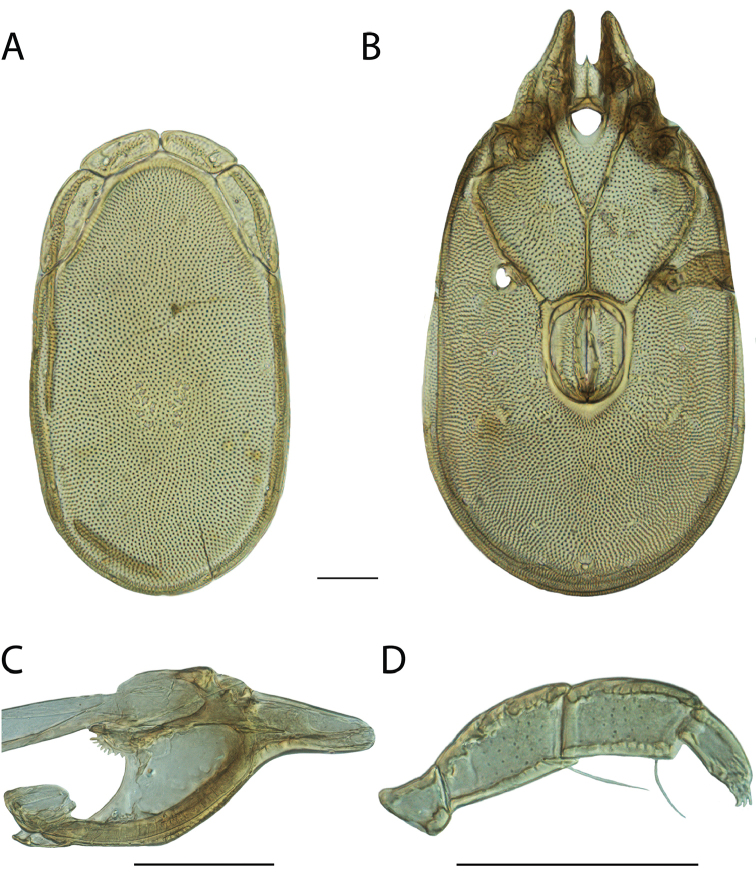
*Torrenticola boettgeri* male: **A** dorsal plates **B** venter (legs removed) **C** subcapitulum **D** pedipalp (setae not accurately depicted). Scale = 100 µm.

######## Remarks.

Unfortunately, we were unable to acquire fresh material of *Torrenticola boettgeri* and therefore this species is not included in our phylogenetic analyses. We were also unable to examine types, but were able to examine new material from New Mexico. The overall appearance, incomplete hind coxal margins, distribution, and lack of coloration are consistent with placing this species in the Rala Identification Group.

Viets (1977a) described *T. boettgeri* from a single female collected in Guatemala. Cramer (1992) described *T. esbelta* from three females collected in one stream (Peña Blanca) in San Francisco Oxtotilpan, State of Mexico, Mexico. Cramer (1992) differentiated *T. esbelta* from *T. boettgeri* by pedipalpal tibia length: 36 µm in *T. esbelta*; 26-27 µm (right, left, respectively). Given our experience with the variability of tibial length across species, which often range well over 10 µm, and especially considering the very few number of specimens examined, we do not consider slight variations in pedipalp tibial length to be good evidence for separate species. Furthermore, our material from New Mexico includes specimens with tibiae in between the previously recorded specimens (32.5 µm in our single female specimen; 32.5–37.7 µm in males). Therefore, we consider *T. esbelta* as a junior synonym of *T. boettgeri*.

####### Torrenticola bondi

Taxon classificationAnimaliaTrombidiformesTorrenticolidae

Fisher & Dowling sp. n.

http://zoobank.org/41C5A481-7EF0-4E1E-AD87-CD12DBFBA107

######## Material examined.

HOLOTYPE (♀): from USA, North Carolina, Haywood County, Great Smokey Mountains National Park, Cataloochee (35°37'31"N, 83°6'46"W), 20 Sep 2010, by IM Smith, IMS100148, DNA 1431.

PARATYPES (4 ♀; 5 ♂): **North Carolina, USA**: 1 ♂ (ALLOTYPE) from Haywood County, Great Smoky Mountains National Park; Cataloochee; beside road to Nellie 0.2 km west of Pretty Hollow Gap Trailhead, (35°37'37"N, 83°6'6"W), 16 June 2006, by IM Smith, IMS060012 • 1 ♀ from Haywood County, Great Smoky Mountains National Park; Cataloochee; beside road to Nellie 0.2 km west of Pretty Hollow Gap Trailhead, (35°37'37"N, 83°6'6"W), 16 June 2006, by IM Smith, IMS060012 • 1 ♀ and 1 ♂ from Haywood County, Great Smoky Mountains National Park; Cataloochee; beside road to Nellie 0.8 km west of Pretty Hollow Gap Trailhead, (35°38'38"N, 83°4'4"W), 20 June 2006, by IM Smith, IMS060022 • 1 ♂ from Haywood County, Great Smoky Mountains National Park; Cataloochee; beside road to Nellie 0.4 km west of Pretty Hollow Gap Trailhead, (35°37'37"N, 83°6'6"W), 3 October 2007, by IM Smith, IMS0701002 • ♀ and 2 ♂ from Haywood County, Great Smoky Mountains National Park; Waterville; Big Creek Picnic Area, (35°45'45"N, 83°6'6"W), 26 September 2007, by IM Smith, IMS070089

######## Type deposition.

Holotype (♀), allotype (♂), and some paratypes (2 ♀; 2 ♂) deposited in the CNC; other paratypes (2 ♀; 2 ♂) deposited in the ACUA.

######## Diagnosis.

*Torrenticola bondi* are similar to species with similar dorsal patterning, such as the Rusetria “4-Plate” group (*T. dunni*, *T. glomerabilis*, *T. kittatinniana*, *T. pollani*, *T. rufoalba*, and *T. shubini*), Elongata Group (*T. elongata*, *T. gorti*, and *T. reduncarostra*), Neoanomala Group (*T. interiorensis* and *T. neoanomala*), and *T. erectirostra*, *T. robisoni*, *T. irapalpa*, *T. racupalpa*, *T. skvarlai*, and *T. arktonyx*. They can be differentiated from Rusetria 4-Plates and *T. skvarlai* by having distinct hind coxal margins. *T. bondi* can be differentiated from *T. erectirostra* and *T. robisoni* by having a straight, anteriorly-directed rostrum (upturned in others). *T. bondi* can be differentiated from *T. arktonyx* by having an unmodified dorsal plate (*T. arktonyx* has distinctive longitudinal dark markings on the anterior portion of the dorsal plate that fade posteriorly). *T. bondi* can be differentiated from *T. racupalpa* and *T. irapalpa* by having a more ovoid dorsum (dorsum length/width ♀ = 1.35–1.41 in *T. bondi*, 1.17–1.28 in others; ♂ = 1.32–1.45 in *T. bondi*, 1.20–1.30 in others) and shorter pedipalpal tibiae (♀ = 90–98 in *T. bondi*, 100–125 in others, ♂ = 77–83 in *T. bondi*, 87–110 in others). *T. bondi* can be differentiated from the Elongata Group by having a less elongate dorsum (length/width ♀ = 1.35–1.41 in *T. bondi*, 1.45–2.08 in Elongata Group; ♂ = 1.32–1.45 in *T. bondi*, 1.51–1.70 in Elongata Group) and having a stockier rostrum (length/width = 2.76–3.13 in *T. bondi*, 3.24–4.00 in Elongata Group). *T. bondi* can be differentiated from the Neoanomala Group by having a shorter medial suture (♀ = 10–15 in *T. bondi*, 22–40 in Neoanomala Group; ♂ = 55–70 in *T. bondi*, 75–108 in Neoanomala Group) and anterior venter/genital field width (♀ = 1.15–1.25 in *T. bondi*, 1.31–1.45 in Neoanomala Group; ♂ = 1.95–2.05 in *T. bondi*, 2.09–2.66 in Neoanomala Group).

######## Description.

**Female (Figure 33)** (n = 5) (holotype measurements in parentheses when available) with characters of the genus with following specifications.

**Dorsum** — (620–670 (620) long; 440–490 (440) wide) ovoid with bluish-purple coloration separated into anterior and posterior portions and faint orange medially. Anterio-medial platelets (132.5–147.5 (132.5) long; 55–62.5 (55) wide). Anterio-lateral platelets (192.5–200 (192.5) long; 67.5–77.5 (67.5) wide) free from dorsal plate. Dgl-4 approaching midway between muscle scars and dorsum edge (distance between Dgl-4 270–290 (270)). Dorsal plate proportions: dorsum length/width 1.35–1.41 (1.41); dorsal width/distance between Dgl-4 1.63–1.72 (1.63); anterio-medial platelet length/width 2.20–2.52 (2.41); anterio-lateral platelet length/width 2.58–2.85 (2.85); anterio-lateral/anterio-medial length 1.34–1.41 (1.45).

**Gnathosoma — Subcapitulum** (355–380 (355) long (ventral); 255–295 (255) long (dorsal); 135–150 (135) tall) faint bluish purple coloration. Rostrum (145–155 (145) long; 50–55 (52.5) wide). Chelicerae (354–385 (354) long) with curved fangs 52–75 (52) long). Subcapitular proportions: ventral length/height 2.50–2.63 (2.63); rostrum length/width 2.76–3.00 (2.76). **Pedipalps** with tuberculate ventral extensions on femora and genua. Palpomeres: trochanter 47.5–52.5 (47.5) long); femur (120–140 (120) long); genu (67.5–77.5 (67.5) long); tibia (90–97.5 (92.5) long; 22.5–25 (22.5) wide); tarsus (17.5–20 (17.5) long). Palpomere proportions: femur/genu 1.74–1.83 (1.78); tibia/femur 0.67–0.77 (0.77); tibia length/width 3.90–4.11 (4.11).

**Venter** — (760–840 (760) long; 509–580 (509) wide) with faint bluish-purple coloration. Gnathosomal bay (170–190 (170) long; 90–102.5 (102.5) wide). Cxgl-4 subapical. **Medial suture** (10–15 (10) long). **Genital plates** (175–180 (175) long; 150–160 (150) wide). Additional measurements: Cx-1 (306–330 (306) long (total); 108–155 (108) long (medial)); Cx-3 (365–410 (382) wide); anterior venter (177.5–187.5 (187.5) long). Ventral proportions: gnathosomal bay length/width 1.66–2.11 (1.66); anterior venter/genital field length 0.99–1.07 (1.07); anterior venter length/genital field width 1.15–1.25 (1.25); anterior venter/medial suture 12–18.75 (18.75).

**Male (Figure 34)** (n = 5) (allotypic measurements in parentheses when available) with characters of the genus with following specifications.

**Dorsum** — (515–550 (550) long; 380–410 (380) wide) ovoid with bluish-purple coloration separated into anterior and posterior portions and faint orange medially. Anterio-medial platelets (112.5–122.5 (122.5) long; 47.5–57.5 (57.5) wide). Anterio-lateral platelets (162.5–190 (180) long; 55–67.5 (67.5) wide) free from dorsal plate. Dgl-4 approaching midway between muscle scars and dorsum edge (distance between Dgl-4 235–260 (250)). Dorsal plate proportions: dorsum length/width 1.32–1.45 (1.45); dorsal width/distance between Dgl-4 1.48–1.62 (1.52); anterio-medial platelet length/width 2.13–2.47 (2.13); anterio-lateral platelet length/width 2.65–3.17 (2.67); anterio-lateral/anterio-medial length 1.44–1.57 (1.47).

**Gnathosoma — Subcapitulum** (295–305 (305) long (ventral); 220–230 (225) long (dorsal); 107.5–112.5 (112.5) tall) faint bluish purple coloration. Rostrum (120–125 (125) long; 40–40 (40) wide). Chelicerae (285–295 (290) long) with curved fangs 50–55 (55) long). Subcapitular proportions: ventral length/height 2.68–2.79 (2.71); rostrum length/width 3.00–3.13 (3.13). **Pedipalps** with tuberculate ventral extensions on femora and genua. Palpomeres: trochanter 40–42.5 (42.5) long); femur (102.5–107.5 (105) long); genu (60–62.5 (60) long); tibia (77.5–82.5 (77.5) long; 21.25–23.75 (22.5) wide); tarsus (17.5–20 (20) long). Palpomere proportions: femur/genu 1.68–1.75 (1.75); tibia/femur 0.74–0.80 (0.74); tibia length/width 3.44–3.88 (3.44).

**Venter** — (660–670 (665) long; 420–490 (460) wide) with faint bluish-purple coloration. Gnathosomal bay (140–150 (142.5) long; 75–90 (80) wide). Cxgl-4 subapical. **Medial suture** (55–70 (60) long). **Genital plates** (132.5–142.5 (140) long; 107.5–112.5 (110) wide). Additional measurements: Cx-1 (270–290 (285) long (total); 125–140 (135) long (medial)); Cx-3 (310–340 (335) wide); anterior venter (215–220 (220) long). Ventral proportions: gnathosomal bay length/width 1.61–1.93 (1.78); anterior venter/genital field length 1.54–1.66 (1.57); anterior venter length/genital field width 1.95–2.05 (2.00); anterior venter/medial suture 3.07–3.91 (3.67).

**Immatures** unknown.

######## Etymology.

Specific epithet (*bondi*) named in honor of arachnologist Jason Bond, for his research on species delimitation and integrative taxonomy, which has been an inspiration to JRF, and for his thoughtful career advice, which was greatly appreciated.

######## Distribution.

**Figure 32.**
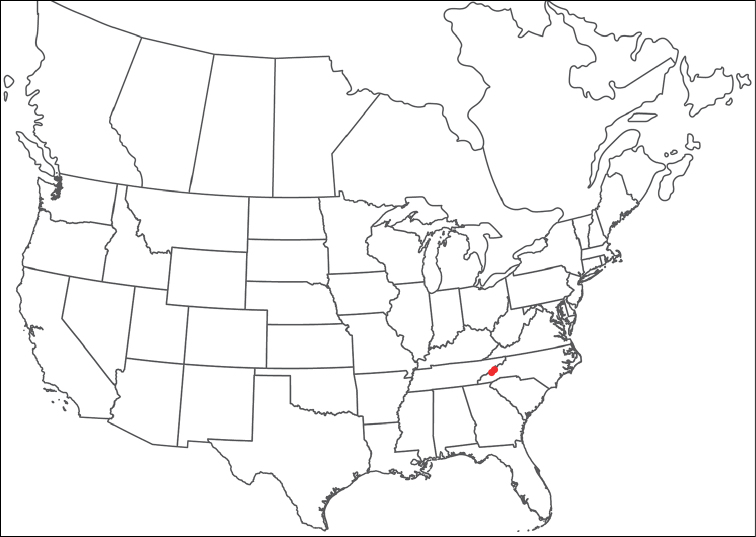
*Torrenticola bondi* sp. n. distribution.

Known only from Great Smoky Mountains National Park, Haywood County, North Carolina (Figure 32).

**Figure 33.**
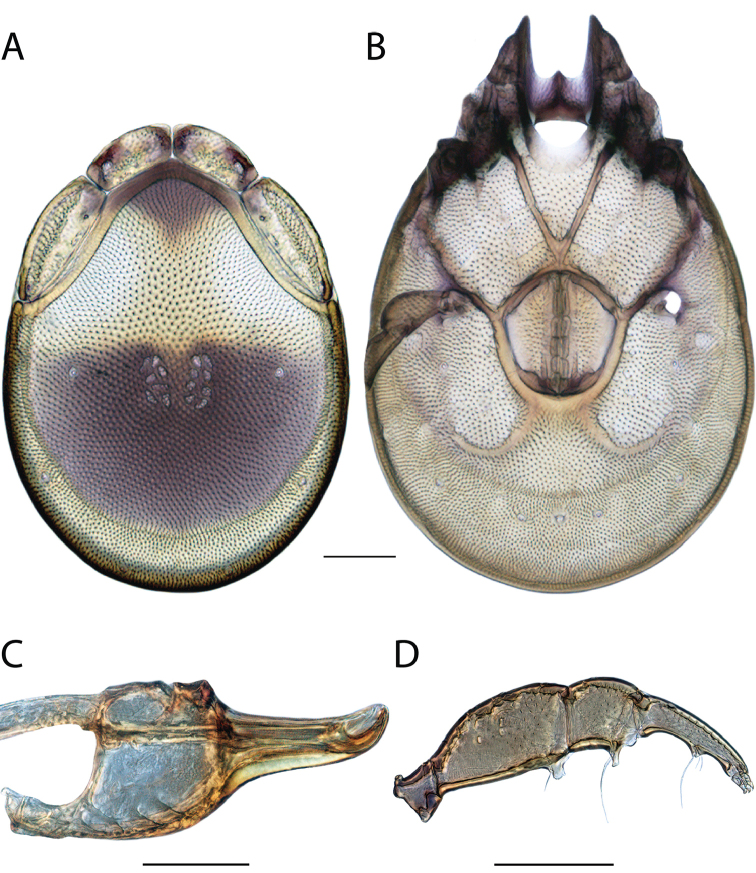
*Torrenticola bondi* sp. n. female: **A** dorsal plates **B** venter (legs removed) **C** subcapitulum **D** pedipalp (setae not accurately depicted). Scale = 100 µm.

**Figure 34.**
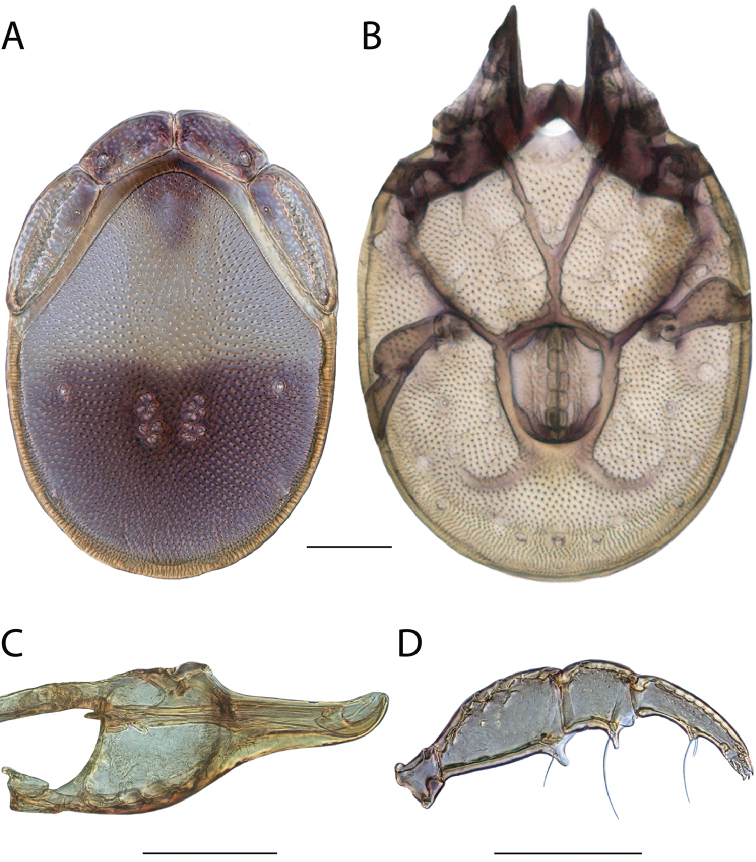
*Torrenticola bondi* sp. n. male: **A** dorsal plates **B** venter (legs removed) **C** subcapitulum **D** pedipalp (setae not accurately depicted). Scale = 100 µm.

######## Remarks.

*Torrenticola bondi* groups with other members of the Raptor Complex in all analyses with high support. Only one specimen could be acquired for use in our analyses, so differences in COI sequence across specimens could not be investigated, but this single specimen was greater than 5% different in COI sequence from sister species. In all analyses, *T. bondi* grouped with members of the Elongata Identification Group (*T. elongata* and *T. gorti*). However, the position of this clade varied with analysis.

This species hypothesis is supported by high divergence between species (3–15%) and by the morphological characters outlined in the diagnosis.

####### Torrenticola caerulea

Taxon classificationAnimaliaTrombidiformesTorrenticolidae

Fisher & Dowling sp. n.

http://zoobank.org/3222693B-DEA0-4BC2-94B1-CC061A2C9730

######## Material examined.

HOLOTYPE (♀): from USA, Tennessee, Wayne County, beside service road parallel to Natchez Trace Parkway (35°15'9"N, 87°37'53"W), 27 Sep 2010, by IM Smith, IMS100160, DNA 1882.

PARATYPES (5 ♀; 3 ♂): **Tennessee, USA**: 2 ♀ from Wayne County, beside Natchez Trace Parkway at Lower Glenrock Branch Picnic Area, (35°15'15"N, 87°37'37"W), 2 June 1992, by IM Smith, IMS920021 • 1 ♂ (ALLOTYPE) from Wayne County, Lower Glenrock Picnic Area off Natchez Trace Parkway, (35°15'15"N, 87°37'37"W), 5 October 2005, by IM Smith, IMS050119A • 1 ♀ and 2 ♂ from Wayne County, Lower Glenrock Picnic Area off Natchez Trace Parkway, (35°15'15"N, 87°37'37"W), 5 October 2005, by IM Smith, IMS050119A • 2 ♀ from Wayne County, beside service road parallel to Natchez Trace Parkway (35°15'9"N, 87°37'53"W), 27 Sep 2010, by IM Smith, IMS100160.

######## Type deposition.

Holotype (♀), allotype (♂), and some paratypes (3 ♀; 1 ♂) deposited in the CNC; other paratypes (2 ♀; 1 ♂) deposited in the ACUA.

######## Diagnosis.

*Torrenticola caerulea* are similar to other members of the Rusetria “Eastern 2-Plates” group (*T. biscutella*, *T. delicatexa*, *T. feminellai*, *T. indistincta*, *T. malarkeyorum*, *T. microbiscutella*, *T. pendula*, *T. sellersorum*, *T. tysoni*, *T. ululata*, and *T. whitneyae*) in having anterio-lateral platelets fused to the dorsal plate, having dorsal coloration separated into anterior and posterior portions (except *T. ululata* and *T. indistincta*), and being distributed in the east. *T. caerulea* can be differentiated from all other Eastern 2-Plates by having faint blue coloration. *T. caerulea* can be differentiated from *T. ululata* and *T. feminellai* by dorsal coloration and pattern. *T. caerulea* can be differentiated from *T. tysoni* by having a less elongate rostrum (length/width = 2.67–2.96 in *T. caerulea*, 3.06–3.50 in *T. tysoni*). *T. caerulea* can be differentiated from *T. pendula* by having a more elongate gnathosomal bay (1.40–2.09 in *T. caerulea*, 2.42–2.90 in *T. pendula*) and more elongate pedipalpal tibiae (3.11–3.83 in *T. caerulea*, 2.78–3.05 in *T. pendula*). *T. caerulea* can be differentiated from *T. microbiscutella* by having a less elongate dorsum (length/width = 1.32–1.56 in *T. caerulea*, 1.63–1.75 in *T. microbiscutella*). *T. caerulea* can be differentiated from *T. whitneyae* by having more elongate pedipalpal tibiae (length/width = 3.11–3.83 in *T. caerulea*, 2.42–2.95 in *T. whitneyae*) and by anterior venter/genital field width (♀ = 0.9–1.04 in *T. caerulea*, 0.67–0.80 in *T. whitneyae*; ♂ = 1.71–1.83 in *T. caerulea*, 1.5–1.54 in *T. whitneyae*). Female *T. caerulea* can be differentiated from female *T. biscutella* by having slightly more elongate pedipalpal tibae (length/width = 3.5–3.83 in *T. caerulea*, 3.35–3.45 in *T. biscutella*). Male *T. caerulea* can be differentiated from male *T. biscutella* by anterior venter/medial suture (2.4–2.57 in *T. caerulea*, 2.68–2.83 in *T. biscutella*). Female *T. caerulea* can be differentiated from female *T. malarkeyorum*, *T. sellersorum*, *T. delicatexa*, and *T. indistincta* by having a thinner genital field (120–145 in *T. caerulea*, 150–205 in others). Male *T. caerulea* can be differentiated from male *T. indistincta* by having a smaller dorsum (length = 405–460 in *T. caerulea*, 480–645 in *T. indistincta*; width = 260–305 in *T. caerulea*, 315–470 in *T. indistincta*). Body proportions of male *T. caerulea* do not differ from male *T. malarkeyorum*, *T. sellersorum*, and *T. delicatexa*, but can be differentiated by dorsal coloration.

######## Description.

**Female (Figure 36)** (n = 5) (holotype measurements in parentheses when available) with characters of the genus with following specifications.

**Dorsum** — (550–600 (580) long; 400–440 (440) wide) ovoid with faint blue coloration anteriorly and posteriorly, broadly connected medially. Anterio-medial platelets (125–130 (128.75) long; 40–45 (42.5) wide). Anterio-lateral platelets (145–157.5 (145) long; 57.5–65 (62.5) wide) fused to dorsal plate. Dgl-4 much closer to the edge of the dorsum than to the muscle scars (distance between Dgl-4 275–320 (320)). Dorsal plate proportions: dorsum length/width 1.32–1.48 (1.32); dorsal width/distance between Dgl-4 1.25–1.51 (1.38); anterio-medial platelet length/width 2.78–3.25 (3.03); anterio-lateral platelet length/width 2.32–2.48 (2.32); anterio-lateral/anterio-medial length 1.12–1.21 (1.13).

**Gnathosoma — Subcapitulum** (310–330 (320) long (ventral); 225–245 (240) long (dorsal); 137.5–156.25 (155) tall) colorless. Rostrum (120–135 (125) long; 45–47.5 (45) wide). Chelicerae (310–330 (320) long) with curved fangs (62–70 (65) long). Subcapitular proportions: ventral length/height 2.06–2.25 (2.06); rostrum length/width 2.67–2.84 (2.78). **Pedipalps** with tuberculate ventral extensions on femora and genua. Palpomeres: trochanter (40–48.75 (48.75) long); femur (115–120 (120) long); genu (67.5–72.5 (72.5) long); tibia (85–87.5 (87.5) long; 22.5–25 (25) wide); tarsus (17.5–20 (20) long). Palpomere proportions: femur/genu 1.66–1.78 (1.66); tibia/femur 0.71–0.76 (0.73); tibia length/width 3.50–3.83 (3.50).

**Venter** — (620–750 (660) long; 400–580 (660) wide) with faint blue coloration. Gnathosomal bay (145–175 (145) long; 82.5–116.25 (95) wide). Cxgl-4 subapical. **Medial suture** absent. **Genital plates** (155–165 (155) long; 140–145 (145) wide). Additional measurements: Cx-1 (257.5–305 (257.5) long (total); 125–135 (125) long (medial)); Cx-3 (310–380 (380) wide); anterior venter (130–150 (137.5) long). Ventral proportions: gnathosomal bay length/width 1.40–1.94 (1.53); anterior venter/genital field length 0.82–0.97 (0.89); anterior venter length/genital field width 0.90–1.04 (0.95).

**Male (Figure 36)** (n = 3) (allotypic measurements in parentheses when available) with characters of the genus with following specifications.

**Dorsum** — (405–460 (460) long; 260–305 (300) wide) ovoid with faint blue coloration anteriorly and posteriorly, broadly connected medially. Anterio-medial platelets (95–106.25 (106.25) long; 35–37.5 (35) wide). Anterio-lateral platelets (117.5–130 (125) long; 40–47.5 (45) wide) fused to dorsal plate. Dgl-4 much closer to the edge of the dorsum than to the muscle scars (distance between Dgl-4 190–240 (230)). Dorsal plate proportions: dorsum length/width 1.51–1.56 (1.53); dorsal width/distance between Dgl-4 1.27–1.37 (1.30); anterio-medial platelet length/width 2.53–3.04 (3.04); anterio-lateral platelet length/width 2.74–2.94 (2.78); anterio-lateral/anterio-medial length 1.18–1.24 (1.18).

**Gnathosoma — Subcapitulum** (220–242.5 (227.5) long (ventral); 165–185 (165) long (dorsal); 87.5–97.5 (97.5) tall) colorless. Rostrum (88.75–92.5 (92.5) long; 30–35 (33.75) wide). Chelicerae (225–232.5 (227.5) long) with curved fangs (42.5–50 (45) long). Subcapitular proportions: ventral length/height 2.33–2.51 (2.33); rostrum length/width 2.64–2.96 (2.74). **Pedipalps** with tuberculate ventral extensions on femora and genua. Palpomeres: trochanter (37.5–37.5 (37.5) long); femur (83.75–87.5 (87.5) long); genu (52.5–55 (55) long); tibia (67.5–70 (67.5) long; 20–22.5 (20) wide); tarsus (15–20 (17.5) long). Palpomere proportions: femur/genu 1.59–1.60 (1.59); tibia/femur 0.77–0.81 (0.77); tibia length/width 3.11–3.38 (3.38).

**Venter** — (485–550 (550) long; 305–340 (330) wide) with faint blue coloration. Gnathosomal bay (120–127.5 (125) long; 57.5–65 (60) wide). Cxgl-4 subapical. **Medial suture** (65-75 (75)). **Genital plates** (110–117.5 (117.5) long; 90–105 (102.5) wide). Additional measurements: Cx-1 (220–220 (220) long (total); 85–95 (95) long (medial)); Cx-3 (240–260 (260) wide); anterior venter (165–180 (180) long). Ventral proportions: gnathosomal bay length/width 1.96–2.09 (2.08); anterior venter/genital field length 1.50–1.57 (1.53); anterior venter length/genital field width 1.71–1.83 (1.76).

**Immatures** unknown.

######## Etymology.

Specific epithet (*caerulea*) refers to the overall and diagnostic bluish appearance of this species (*caeruleus*, L. sky-blue).

######## Distribution.

**Figure 35.**
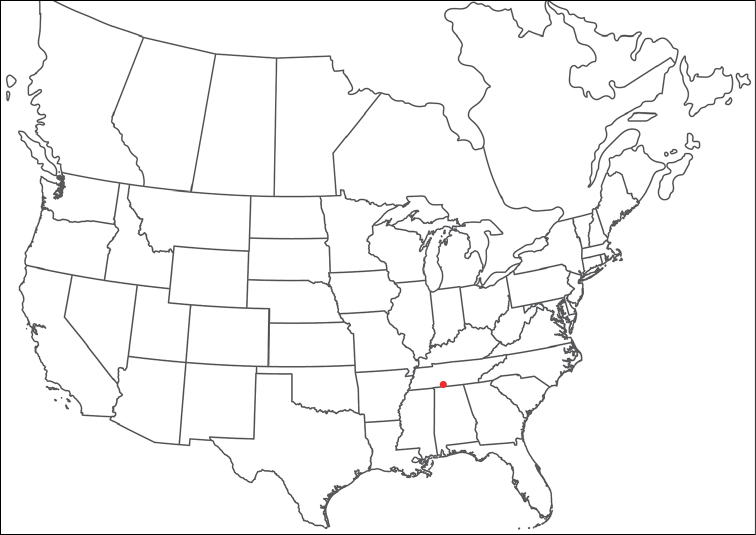
*Torrenticola caerulea* sp. n. distribution.

Known only from Wayne County, Tennessee (Figure 35). *T. caerulea* has been collected so rarely that comments about distribution are speculative, but given our collection efforts, it is reasonable to speculate that this species is at least restricted to the southern Appalachians.

**Figure 36.**
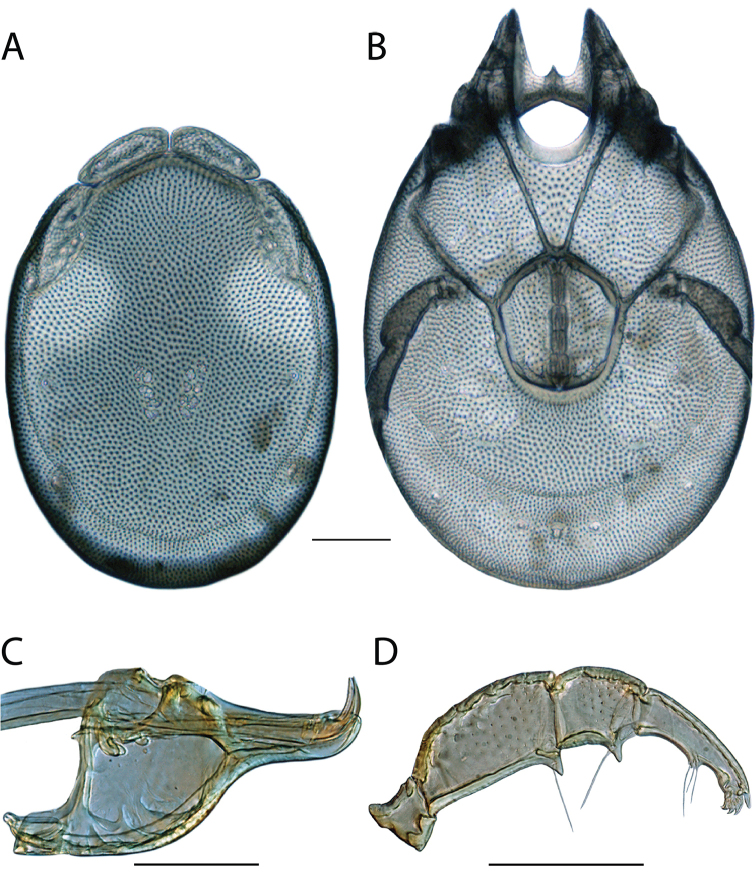
*Torrenticola caerulea* sp. n. female: **A** dorsal plates **B** venter (legs removed) **C** subcapitulum **D** pedipalp (setae not accurately depicted). Scale = 100 µm.

**Figure 37.**
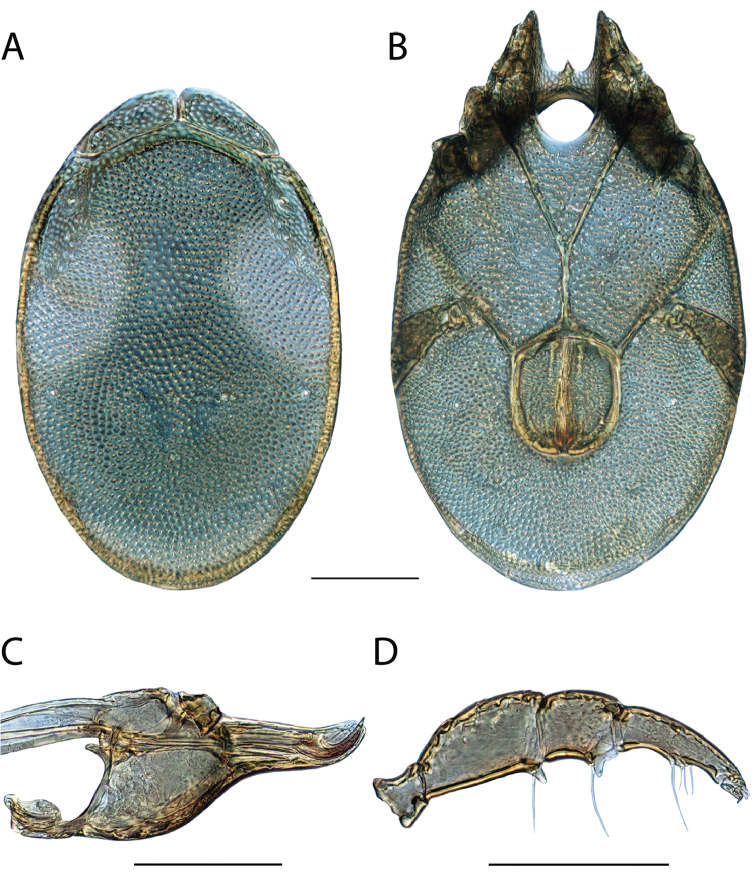
*Torrenticola caerulea* sp. n. male: **A** dorsal plates **B** venter (legs removed) **C** subcapitulum **D** pedipalp (setae not accurately depicted). Scale = 100 µm.

######## Remarks.

*Torrenticola caerulea* groups with other members of the Rusetria Complex with high support and specimens of this species were less than 1% different in COI sequence from each other. In all analyses, *T. caerulea* groups with two other morphologically similar species: *T. biscutella* and *T. malarkeyorum*. These three species are 3–5% different from each other in COI sequence. The three of these species are morphologically similar to the more distantly-related *T. delicatexa*, but *T. caerulea* can be differentiated from all of these by color. The range of *T. caerulea* overlaps with each of these except for *T. biscutella*, which is not known from east of the Mississippi River.

This species hypothesis is supported by biogeography, low COI divergence within the species (0–2%) and high divergence between species (3–15%), and by the morphological characters outlined in the diagnosis.

####### Torrenticola cardia

Taxon classificationAnimaliaTrombidiformesTorrenticolidae

Fisher & Dowling sp. n.

http://zoobank.org/25AFD6CD-A326-41FE-BB54-F917F101C9E6

######## Material examined.

HOLOTYPE (♀): from USA, New York, Greene County, beside Rt. 23A, 9.6 km west of Rt. 296 (Hunter), (42°14'14"N, 74°19'19"W), 22 June 1990, by IM Smith, IMS900052

PARATYPES (9 ♀; 8 ♂): **New York, USA**: 3 ♀ and 3 ♂ from Cayuga County, beside Route 38A at Niles, (42°50'50"N, 76°25'25"W), 22 July 1990, by IM Smith, IMS900113A • (allotype) 1 ♂ from Greene, beside Rt. 23A, 9.6 km west of Rt. 296 (Hunter), (42°14'14"N, 74°19'19"W), 22 June 1990, by IM Smith, IMS900052 • 2 ♀ and 1 ♂ from Greene County, beside Rt. 23A, 9.6 km west of Rt. 296 (Hunter), (42°14'14"N, 74°19'19"W), 22 June 1990, by IM Smith, IMS900052 • 1 ♀ from Schuyler County, beside Town Line Road off Route 228, 0.6 km south of Perry City, (42°29'29"N, 76°42'42"W), 21 July 1990, by IM Smith, IMS900112A • **Ohio, USA**: 1 ♀ and 1 ♂ from Hocking County, beside road near Ash Cave, (39°24'24"N, 82°33'33"W), 5 May 1993, by IM Smith, DR Cook, IMS930001A • **Virginia, USA**: 2 ♀ and 2 ♂ from Bath County, beside Rt. 687, 2.4 km south of Bacova, (38°2'2"N, 79°51'51"W), 15 July 1990, by IM Smith, IMS900097

######## Type deposition.

Holotype (♀), allotype (♂), and some paratypes (5 ♀; 4 ♂) deposited in the CNC; other paratypes (4 ♀; 3 ♂) deposited in the ACUA.

######## Diagnosis.

*Torrenticola cardia* are similar to other members of the Tricolor Complex (*T. bittikoferae*, *T. dimorpha*, *T. hoosieri*, *T. kringi*, *T. larvata*, *T. mohawk*, *T. pearsoni*, *T. olliei*, *T. sierrensis*, *T. tricolor*, *T. trimaculata*, and *T. unimaculata*,) in having a short, conical rostrum. *T. cardia* can be differentiated from most *Torrenticola*, including other members of the Tricolor Complex, by having a distinct dorsal pattern. *T. cardia* are most similar to other members of the Tricolor Complex that have bold patterning (*T. larvata*, *T. tricolor*, *T. unimaculata*, *T. trimaculata*, *T. kringi*, and *T. mohawk*). *T. cardia* can be differentiated from *T. tricolor*, *T. trimaculata*, *T. kringi*, and *T. mohawk* by having a more ovoid dorsum (length/width ♀ = 1.39–1.47 in *T. cardia*, 1.15–1.35 in others; ♂ = 1.43–1.54 in *T. cardia*, 1.19–1.39 in others). *T. cardia* can be differentiated from *T. unimaculata* by dorsal pattern. Female *T. cardia* can be differentiated from female *T. larvata* by having a shorter subcapitulum (♀ = 265–273 in *T. cardia*, 275–288 in *T. larvata*) and a larger genital field (length ♀ = 190–198 in *T. cardia*, 182–188 in *T. larvata*; width ♀ = 160–175 in *T. cardia*, 145–153 in *T. larvata*). Male *T. cardia* can be differentiated from male *T. larvata* by having less elongate pedipalpal tibiae (length/width ♂ = 2.82–3.05 in *T. cardia*, 3.10–3.20 in *T. larvata*) and a larger dorsum (length ♂ = 625–670 in *T. cardia*, 550–610 in *T. larvata*; width ♂ = 405–445 in *T. cardia*, 350–400 in *T. larvata*).

######## Description.

**Female (Figure 39)** (n = 5) (holotype measurements in parentheses when available) with characters of the genus with following specifications.

**Dorsum** — (710–785 (750) long; 510–555 (510) wide) ellipsoid with reddish-purple, bluish-purple or bright orange spot medially extending in a strip anteriorly often to the anterior-medial platelets. Anterio-medial platelets (130–140 (140) long; 70–75 (75) wide). Anterio-lateral platelets (195–207.5 (197.5) long; 70–80 (80) wide) free from dorsal plate. Dgl-4 much closer to the edge of the dorsum than to the muscle scars (distance between Dgl-4 390–410 (395)). Dorsal plate proportions: dorsum length/width 1.39–1.47 (1.47); dorsal width/distance between Dgl-4 1.29–1.35 (1.29); anterio-medial platelet length/width 1.86–2.00 (1.87); anterio-lateral platelet length/width X=2.47–2.93 (2.47); anterio-lateral/anterio-medial length 1.39–1.50 (1.41).

**Gnathosoma — Subcapitulum** (265–272.5 (272.5) long (ventral); 180–190 (190) long (dorsal); 125–132.5 (130) tall) with reddish-purple or bluish purple coloration. Rostrum (90–100 (100) long; 40–42.5 (40) wide) short and conical. Chelicerae (250–265 (260) long) with curved fangs (60–60 (60) long). Subcapitular proportions: ventral length/height 2.04–2.12 (2.10); rostrum length/width 2.24–2.50 (2.50). **Pedipalps** with stocky, tuberculate ventral extensions on femora and genua. Palpomeres: trochanter (38.75–42.5 41.25) long); femur (97.5–107.5 (102.5) long); genu (65–70 (67.5) long); tibia (80–91.25 (80) long; 26.25–27.5 (27.5) wide); tarsus (22.5–25 (22.5) long). Palpomere proportions: femur/genu 1.50–1.56 (1.52); tibia/femur 0.78–0.87 (0.78); tibia length/width 2.91–3.32 (2.91).

**Venter** — (830–925 (850) long; 565–600 (565) wide) with reddish-purple or bluish-purple coloration restricted to the edges of the gnathosomal bay, coxal plates, and genital plates. Gnathosomal bay (150–162.5 (155) long; 72.5–80 (72.5) wide). Cxgl-4 subapical. **Medial suture** (25–50 (50) long). **Genital plates** (190–197.5 (190) long; 160–175 (162.5) wide). Additional measurements: Cx-1 (290–310 (290) long (total); 135–165 (135) long (medial)); Cx-3 (355–380 (355) wide); anterior venter (185–212.5 (205) long). Ventral proportions: gnathosomal bay length/width 1.94–2.17 (2.14); anterior venter/genital field length 0.94–1.09 (1.08); anterior venter length/genital field width 1.12–1.26 (1.26); anterior venter/medial suture 4.10–7.60 (4.10).

**Male (Figure 40)** (n = 5) (allotypic measurements in parentheses when available) with characters of the genus with following specifications.

**Dorsum** — (625–670 (670) long; 405–445 (445) wide) ellipsoid with reddish-purple, bluish-purple or bright orange spot medially extending in a strip anteriorly often to the anterior-medial platelets. Anterio-medial platelets (120–130 (125) long; 65–72.5 (70) wide). Anterio-lateral platelets (170–197.5 (197.5) long; 75–80 (75) wide) free from dorsal plate. Dgl-4 much closer to the edge of the dorsum than to the muscle scars (distance between Dgl-4 325–380 (380)). Dorsal plate proportions: dorsum length/width 1.43–1.54 (1.51); dorsal width/distance between Dgl-4 1.17–1.28 (1.17); anterio-medial platelet length/width 1.72–2.00 (1.79); anterio-lateral platelet length/width 2.25–2.63 (2.63); anterio-lateral/anterio-medial length 1.35–1.58 (1.58).

**Gnathosoma — Subcapitulum** (227.5–250 (250) long (ventral); 165–180 (170) long (dorsal); 90–112.5 (110) tall) with reddish-purple or bluish purple coloration. Rostrum (77.5–92.5 (90) long; 32.5–37.5 (37.5) wide) short and conical. Chelicerae (212.5–230 (225) long) with curved fangs (50–55 (50) long). Subcapitular proportions: ventral length/height 2.17–2.56 (2.27); rostrum length/width 2.27–2.47 (2.40). **Pedipalps** with stocky, tuberculate ventral extensions on femora and genua. Palpomeres: trochanter (36.25–38.75 (36.25) long); femur (87.5–97.5 (92.5) long); genu (60–67.5 (62.5) long); tibia (73.75–82.5 (77.5) long; 25–27.5 (27.5) wide); tarsus (20–25 (22.5) long). Palpomere proportions: femur/genu 1.38–1.48 (1.48); tibia/femur 0.82–0.90 (0.84); tibia length/width 2.82–3.05 (2.82).

**Venter** — (710–780 (780) long; 460–495 (490) wide) with reddish-purple or bluish-purple coloration restricted to the edges of the gnathosomal bay, coxal plates, and genital plates. Gnathosomal bay (125–137.5 (135) long; 67.5–72.5 (70) wide). Cxgl-4 subapical. **Medial suture** (107.5–130 (125) long). **Genital plates** (150–175 (175) long; 97.5–115 (105) wide). Additional measurements: Cx-1 (265–280 (280) long (total); 140–150 (145) long (medial)); Cx-3 (325–355 (350) wide); anterior venter (265–290 (290) long). Ventral proportions: gnathosomal bay length/width 1.85–1.93 (1.93); anterior venter/genital field length 1.66–1.81 (1.66); anterior venter length/genital field width 2.52–2.77 (2.76); anterior venter/medial suture 2.21–2.51 (2.32).

**Immatures** unknown.

######## Etymology.

Specific epithet (*cardia*) refers to the dorsal coloration, which is either a heart-shaped or resembles a bleeding heart (*kardiá*, G. heart).

######## Distribution.

**Figure 38.**
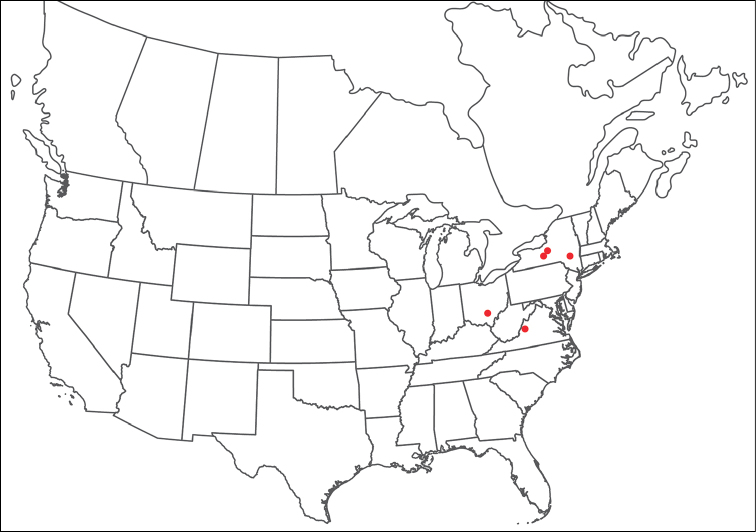
*Torrenticola cardia* sp. n. distribution.

Appalachians (Figure 38).

**Figure 39.**
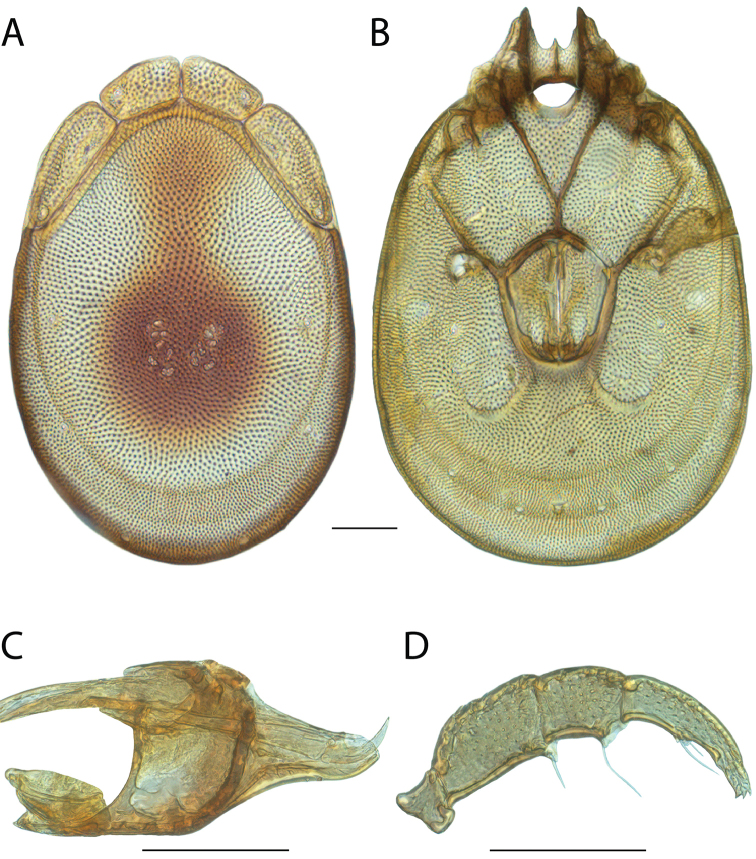
*Torrenticola cardia* sp. n. female: **A** dorsal plates **B** venter (legs removed) **C** subcapitulum **D** pedipalp (setae not accurately depicted). Scale = 100 µm.

**Figure 40.**
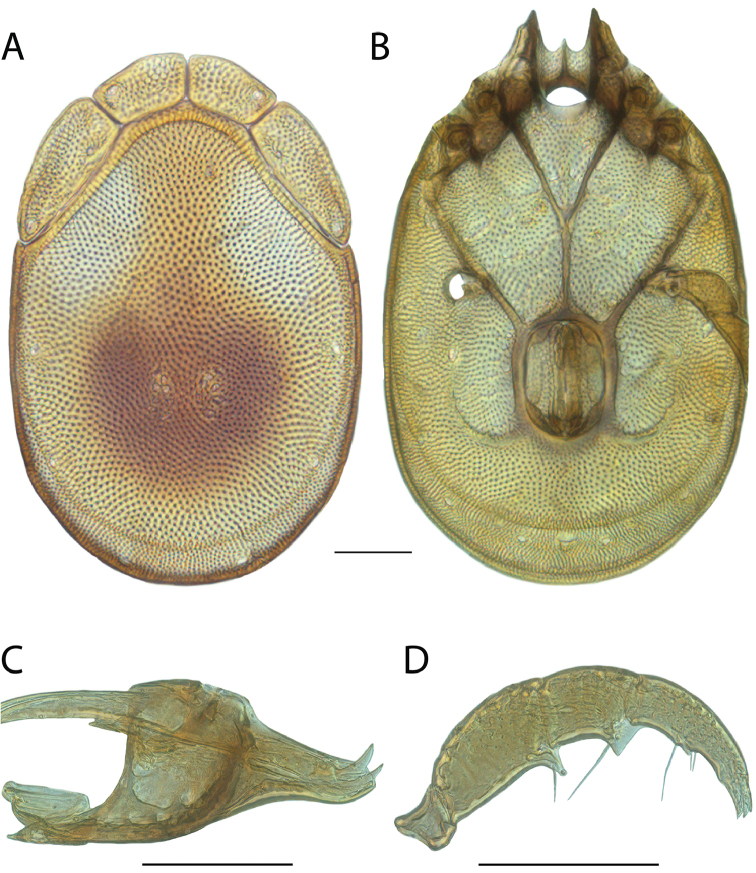
*Torrenticola cardia* sp. n. male: **A** dorsal plates **B** venter (legs removed) **C** subcapitulum **D** pedipalp (setae not accurately depicted). Scale = 100 µm.

######## Remarks.

Unfortunately, we were unable to acquire fresh material of *Torrenticola cardia* and therefore this species is not included in our phylogenetic analyses. However, we were able to examine morphology with material preserved in GAW. The overall appearance, short conical rostrum that is downturned in the male, and distribution, are consistent with placing this species in the Tricolor Complex and Tricolor Identification Group.

####### Torrenticola copipalpa

Taxon classificationAnimaliaTrombidiformesTorrenticolidae

Fisher & Dowling sp. n.

http://zoobank.org/11564193-319A-4192-927A-890726F7D52D

######## Material examined.

HOLOTYPE (♀): from USA, Oregon, Lane County, Gate Creek (44°8'48"N, 122°34'20"W), 11 Aug 2013, by JC O’Neill, & WA Nelson, JNOW 13-0811-001.

PARATYPES (20 ♀; 26 ♂): **California, USA**: 1 ♀ from Alpine County, Markleeville Creek (38°41'39"N, 119°46'41"W), 30 Aug 2013, by JR Fisher, JRF 13-0830-001 • 1 ♀ from Del Norte County, Six Rivers National Forest, Middle Fork Smith River (41°51'20"N, 123°53'10"W), 15 Aug 2013, by JR Fisher, JRF 13-0815-002 • 5 ♂ from El Dorado County, El Dorado National Forest, Taylor Creek (38°55'59"N, 120°3'21"W), 27 Aug 2013, by JR Fisher, JRF 13-0827-003 • 1 ♀ from Mendocino County, Cottaneva Creek, beside Route 1, 21.8 kilometers southwest of Route 101, 5 Aug 1987, by IM Smith, IMS870129A • 2 ♂ from Trinity County, small cascading trickle beside Route 36, 5.2 kilometers west of Forest Glen Station, 6 Aug 1987, by IM Smith, IMS870132 • 1 ♂ from Trinity County, South Fork of Trinity River, beside Route 36 at Forest Glen campground, 6 Aug 1987, by IM Smith, IMS870131 • **Oregon, USA**: 2 ♂ from Coos County, Gaylord, Coquille Myrtle Grove State Park, Coquille River, 2 Jul 1983, by IM Smith, IMS830014 • 3 ♀ and 3 ♂ from Coos County, Siskiyou National Forest, Road 33 between Powers & Agness, Coal Creek, 2 Jul 1983, by IM Smith, IMS830015 • 2 ♂ from Coos County, Siskiyou National Forest, Road 33 between Powers & Agness, Daphne Grove campground, Coquille River, 2 Jul 1983, by IM Smith, IMS830016 • 1 ♂ from Coos County, Siskiyou National Forest, Road 33 between Powers & Agness, Daphne Grove campground, 2 Jul 1983, by IM Smith, IMS830017 • 1 ♀ and 1 ♂ from Curry County, Port Orford, Butler Bar campground, Elk River, 25 Jun 1976, by IM Smith, IMS760162 • 1 ♀ from Curry County, Port Orford, Butler Bar campground, Elk River, 25-26 Jun 1976, by IM Smith, IMS760163 • 2 ♂ from Curry County, Port Orford, small spring run beside road from Humbug Mountain State Park to McGribble campground, 25 Jun 1976, by IM Smith, IMS760161 • 3 ♂ from Curry County, Port Orford, Humbug Mountain State Park Picnic Area, beside Route 1, Brush Creek, 1 Jul 1983, by IM Smith, IMS830012 • 1 ♀ from Curry County, Port Orford, Humbug Mountain State Park Picnic Area, beside Route 1, Brush Creek, 3 Jul 1983, by IM Smith, IMS830020A • 2 ♀ from Curry County, Quosatana Creek (42°29'21"N, 124°14'2"W), 14 Aug 2013, JR Fisher, JRF 13-0814-003 • 1 ♀ from Curry County, Rogue River National Forest, Elk River (42°42'46"N, 124°18'41"W), 13 Aug 2013, by JR Fisher, JRF 13-0813-003 • 4 ♀ and 3 ♂ from Curry County, Sixes, Sixes River, beside road at mouth of Edson Creek, 4 Jul 1983, by IM Smith, IMS830021A • 1 ♂ (ALLOTYPE) from Lane County, Gate Creek (44°8'48"N, 122°34'20"W), 11 Aug 2013, by JC O’Neill, & WA Nelson, JNOW 13-0811-001 • 4 ♀ from Lane County, Gate Creek (44°8'48"N, 122°34'20"W), 11 Aug 2013, by JC O’Neill, & WA Nelson, JNOW 13-0811-001.

######## Type deposition.

Holotype (♀), allotype (♂), and other paratypes (15 ♀; 20 ♂) deposited in the CNC; other paratypes (5 ♀; 5 ♂) deposited in ACUA.

######## Diagnosis.

*Torrenticola copipalpa* are similar to members of the Miniforma group (*T. manni*, *T. miniforma*, *T. oliveri*, *T. pacificensis*, *T. pinocchio*, and *T. rockyensis*) in having short, stocky pedipalps (except *T. oliveri* and *T. pinocchio*); similar pedipalpal extensions (unique to members of this group); and being among the smallest *Torrenticola* in the west (dorsum 500–625 long) (except *T. oliveri*). *T. copipalpa* are best differentiated from all other Miniforma group (except *T. pinocchio*) by having broad, flat pedipalp femoral tubercles (conical/tuberculate in all others). *T. copipalpa* can be differentiated from *T. pinocchio* by having a less elongate rostrum (length/width = 2.5–3.0 in *T. copipalpa*, 4.5–4.9 in *T. pinocchio*) and less elongate pedipalpal tibiae (length/width = 2.4–2.9 in *T. copipalpa*, 3.1–3.5 in *T. pinocchio*).

######## Description.

**Female (Figure 42)** (n = 6) (holotype measurements in parentheses when available) with characters of the genus with following specifications.

**Dorsum** — (555–605 (605) long; 380–420 (420) wide) ovoid and usually colorless, occasionally with faint purple coloration without distinct pattern. Anterio-medial platelets (115–127.5 (127.5) long; 47.5–57.5 (57.5) wide). Anterio-lateral platelets (162.5–180 (180) long; 53.75–62.5 (62.5) wide) free from dorsal plate. Dgl-4 much closer to the edge of the dorsum than to the muscle scars (distance between Dgl-4 290–335 (335)). Dorsal plate proportions: dorsum length/width 1.39–1.47 (1.44); dorsal width/distance between Dgl-4 1.25–1.32 (1.25); anterio-medial platelet length/width 2.13–2.42 (2.22); anterio-lateral platelet length/width 2.87–3.04 (2.88); anterio-lateral/anterio-medial length 1.35–1.46 (1.41).

**Gnathosoma — Subcapitulum** (312.5–337.5 (337.5) long (ventral); 228–257.5 (257.5) long (dorsal); 117.5–130 (125) tall) colorless. Rostrum (122.5–135 (130) long; 42.5–47.5 (45) wide). Chelicerae (313–341 (340) long) with curved fangs (50–59 (55) long). Subcapitular proportions: ventral length/height 2.58–2.70 (2.70); rostrum length/width 2.72–2.94 (2.89). **Pedipalps** short and stocky (especially tibiae) with broad, dentate, and anteriorly-directed ventral extensions on femora and dentate, flanged ventral extensions on genua. Palpomeres: trochanter (30–35 (35) long); femur (90–100 (97.5) long); genu (62.5–67.5 (67.5) long); tibia (52.5–58.75 (57.5) long; 20–22.5 (21.25) wide); tarsus (15–17.5 (15) long). Palpomere proportions: femur/genu 1.44–1.51 (1.41); tibia/femur 0.55–0.61 (0.59); tibia length/width 2.59–2.71 (2.71).

**Venter** — (690–760 (760) long; 438–520 (520) wide) colorless. Gnathosomal bay (136.25–152.5 (152.5) long; 75–82.5 (80) wide). Cxgl-4 subapical. **Medial suture** (40–45 (45) long). **Genital plates** (152.5–165 (165) long; 137.5–160 (160) wide). Additional measurements: Cx-1 (261–290 (290) long (total); 108–155 (155) long (medial)); Cx-3 (268–320 (320) wide); anterior venter (187.5–210 (210) long). Ventral proportions: gnathosomal bay length/width 1.65–1.97 (1.91); anterior venter/genital field length 1.19–1.28 (1.27); anterior venter length/genital field width 1.31–1.40 (1.31); anterior venter/medial suture 4.53–4.88 (4.67).

**Male (Figure 43)** (n = 6) (allotypic measurements in parentheses when available) with characters of the genus with following specifications.

**Dorsum** — (500–570 (520) long; 355–390 (360) wide) ovoid and usually colorless, occasionally with faint purple coloration without distinct pattern. Anterio-medial platelets (105–117.5 (105) long; 45–56.25 (45) wide). Anterio-lateral platelets (155–167.5 (155) long; 50–60 (50) wide) free from dorsal plate. Dgl-4 much closer to the edge of the dorsum than to the muscle scars (distance between Dgl-4 285–315 (290)). Dorsal plate proportions: dorsum length/width 1.39–1.54 (1.44); dorsal width/distance between Dgl-4 1.18–1.37 (1.24); anterio-medial platelet length/width 2.09–2.37 (2.33); anterio-lateral platelet length/width 2.79–3.10 (3.10); anterio-lateral/anterio-medial length 1.32–1.48 (1.48).

**Gnathosoma — Subcapitulum** (280–307.5 (295) long (ventral); 215–253 (220) long (dorsal); 105–115 (105) tall) colorless. Rostrum (110–120 (115) long; 40–46.25 (40) wide). Chelicerae (280–328 (295) long) with curved fangs (45–65 (55) long). Subcapitular proportions: ventral length/height 2.67–2.81 (2.81); rostrum length/width 2.54–2.88 (2.88). **Pedipalps** short and stocky (especially tibiae) with broad, dentate, and anteriorly-directed ventral extensions on femora and dentate, flanged ventral extensions on genua. Palpomeres: trochanter (32.5–32.5 (32.5) long); femur (82.5–92.5 (87.5) long); genu (57.5–65 (60) long); tibia (52.5–57.5 (52.5) long; 18.75–21.25 (21.25) wide); tarsus (15–17.5 (15) long). Palpomere proportions: femur/genu 1.35–1.54 (1.46); tibia/femur 0.60–0.64 (0.60); tibia length/width 2.47–2.88 (2.47).

**Venter** — (610–700 (670) long; 420–496 (440) wide) colorless. Gnathosomal bay (110–140 (130) long; 67.5–75 (75) wide). Cxgl-4 subapical. **Medial suture** (77.5–97.5 (77.5) long). **Genital plates** (130–137.5 (130) long; 100–112.5 (105) wide). Additional measurements: Cx-1 (235–263 (245) long (total); 115–130 (130) long (medial)); Cx-3 (270–300 (280) wide); anterior venter (210–232.5 (217.5) long). Ventral proportions: gnathosomal bay length/width 1.63–1.87 (1.73); anterior venter/genital field length 1.62–1.77 (1.67); anterior venter length/genital field width 2.04–2.21 (2.07); anterior venter/medial suture 2.36–2.88 (2.81).

**Immatures** unknown.

######## Etymology.

Specific epithet (*copipalpa*) refers to the blade-like pedipalp femoral tubercles (*copis*, L. small knife; *palpus*, L. hand, feeler), which distinguish them from similar, co-occurring species.

######## Distribution.

**Figure 41.**
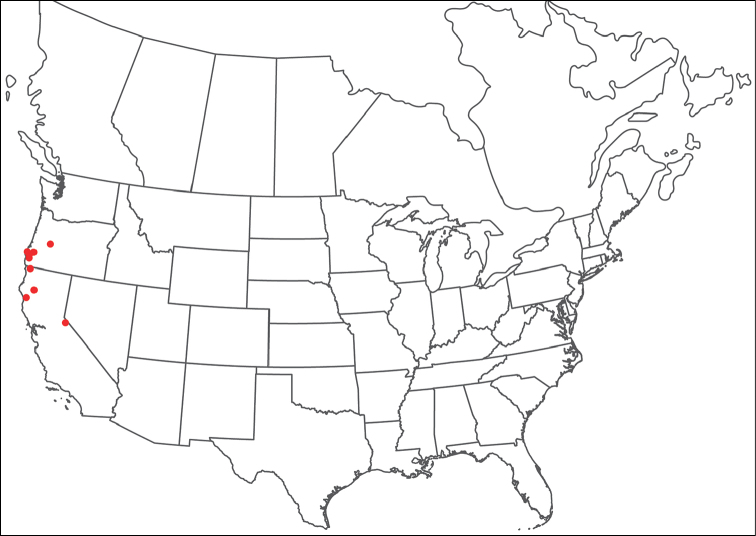
*Torrenticola copipalpa* sp. n. distribution.

Northern California and western Oregon (Figure 41).

**Figure 42.**
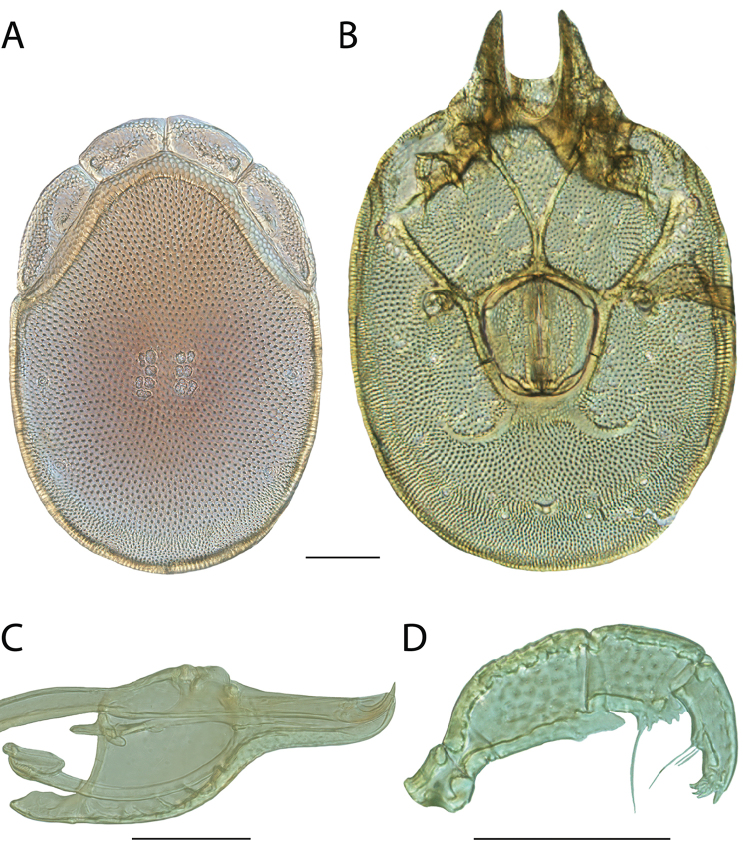
*Torrenticola copipalpa* sp. n. female: **A** dorsal plates **B** venter (legs removed) **C** subcapitulum **D** pedipalp (setae not accurately depicted). Scale = 100 µm.

**Figure 43.**
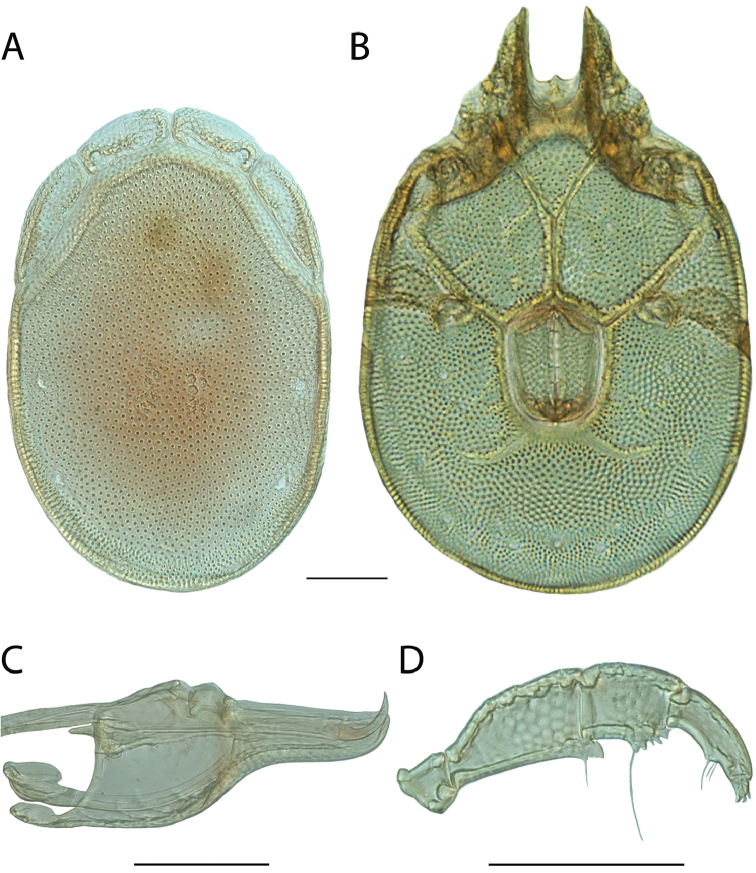
*Torrenticola copipalpa* sp. n. male: **A** dorsal plates **B** venter (legs removed) **C** subcapitulum **D** pedipalp (setae not accurately depicted). Scale = 100 µm.

######## Remarks.

*Torrenticola copipalpa* groups with other members of the Miniforma Complex with high support and specimens of this species are less than 1% different in COI sequence from each other. In all analyses, *T. copipalpa* groups with three other morphologically similar species: *T. pacificensis*, *T. manni*, and *T. rockyensis*. These three species are greater than 4% different from each other. This species overlaps with *T. miniforma* in California and with *T. pacificensis* in west-central Oregon.

Based upon overall similarity, the pedipalp genu extensions, and western distribution, we were able to place this species in the Miniforma Identification Group.

This species hypothesis is supported by biogeography, low COI divergence within the species (0–2%) and high divergence between species (3–15%), and by the morphological characters outlined in the diagnosis.

####### Torrenticola daemon

Taxon classificationAnimaliaTrombidiformesTorrenticolidae

Fisher & Dowling sp. n.

http://zoobank.org/5ACD786A-B621-48B1-9480-CB7B58CE3438

######## Material examined.

HOLOTYPE (♀): from USA, Alabama, Clay County, beside Forest Route 649, 0.8 km northeast of road from Campbell Springs to Forest Route 600, (33°22'22"N, 85°52'52"W), 3 July 1990, by IM Smith, IMS900075A.

PARATYPES (4 ♀; 4 ♂): **Alabama, USA**: 1 ♂ (ALLOTYPE) from Clay County, beside Forest Route 649, 0.8 km northeast of road from Campbell Springs to Forest Route 600, (33°22'22"N, 85°52'52"W), 3 July 1990, by IM Smith, IMS900075A • 4 ♀ and 3 ♂ from Clay County, beside Forest Route 649, 0.8 km northeast of road from Campbell Springs to Forest Route 600, (33°22'22"N, 85°52'52"W), 3 July 1990, by IM Smith, IMS900075A

######## Type deposition.

Holotype (♀), allotype (♂), and some paratypes (2 ♀; 2 ♂) deposited in the CNC; other paratypes (2 ♀; 1 ♂) deposited in the ACUA.

######## Diagnosis.

*Torrenticola daemon* are similar to other members of the Raptor Group (*T. danielleae*, *T. elusiva*, *T. gnoma*, *T. irapalpa*, *T. ivyae*, *T. longitibia*, *T. mjolniri*, *T. racupalpa*, and *T. raptor*) in having round bodies; Dgl-4 close to muscles scars; long, thin subcapitular rostra; and long, thin pedipalp tibiae. *T. daemon* can be differentiated from all other Raptor Group by having Dgl-4 closer to the edge of the dorsum (dorsum width/distance between Dgl-4 ♀ = 1.59–1.67 in *T. daemon*, 1.80–3.29 in others; ♂ = 1.45–1.65 in *T. daemon*, 1.66–2.73 in others), except *T. irapalpa* (♀ = 1.81–2.09, ♂ = 1.58–1.86) and *T. danielleae* (♀ = 1.57–1.70, ♂ = 1.42–1.52). *T. daemon* can be differentiated from *T. longitibia*, *T. mjolniri*, *T. elusiva*, *T. racupalpa*, *T. raptor*, *T. danielleae*, and *T. ivyae* by having a less elongate rostrum (length/width = 2.91–3.31 in *T. daemon*, 3.43–4.40 in others). Female *T. daemon* can be differentiated from female *T. irapalpa* by having Dgl-4 closer to the dorsal edge (dorsal width/distance between Dgl-4 ♀ = 1.59–1.67 in *T. daemon*, 1.81–2.09 in *T. irapalpa)* and a more elongate gnathosomal bay (length/width ♀ = 1.95–2.42 in *T. daemon*, 1.35–1.86 in *T. irapalpa*). Additionally, *T. daemon* can be differentiated from *T. irapalpa* by dorsal coloration and pattern.

######## Description.

**Female (Figure 45)** (n = 5) (holotype measurements in parentheses when available) with characters of the genus with following specifications.

**Dorsum** — (590–655 (640) long; 460–500 (490) wide) circular with faint reddish-purple coloration separated into anterior and posterior portions, with bright reddish-purple coloration on the anterior-medial platelets, occasionally extending onto the dorsal plate. Anterio-medial platelets (127.5–147.5 (145) long; 57.5–67.5 (65) wide). Anterio-lateral platelets (182.5–210 (210) long; 72.5–82.5 (82.5) wide) free from dorsal plate. Dgl-4 approximately halfway between the edge of the dorsum and the muscle scars (distance between Dgl-4 285–300 (300)). Dorsal plate proportions: dorsum length/width 1.26–1.34 (1.31); dorsal width/distance between Dgl-4 1.59–1.67 (1.63); anterio-medial platelet length/width 2.04–2.28 (2.23); anterio-lateral platelet length/width 2.28–2.66 (2.55); anterio-lateral/anterio-medial length 1.29–1.51 (1.45).

**Gnathosoma — Subcapitulum** (370–395 (395) long (ventral); 285–300 (300) long (dorsal); 145–157.5 (157.5) tall) colorless, occasionally with faint reddish-purple coloration. Rostrum (155–165 (165) long; 50–55 (55) wide). Chelicerae (375–390 (390) long) with curved fangs (65–75 (75) long). Subcapitular proportions: ventral length/height 2.39–2.57 (2.51); rostrum length/width 2.91–3.20 (3.00). **Pedipalps** with tuberculate ventral extensions on femora and genua. Palpomeres: trochanter (50–52.5 (50) long); femur (137.5–146.25 (145) long); genu (75–80 (80) long); tibia (97.5–105 (105) long; 22.5–25 (25) wide); tarsus (20–22.5 (20) long). Palpomere proportions: femur/genu 1.81–1.90 (1.81); tibia/femur 0.67–0.72 (0.72); tibia length/width 4.05–4.33 (4.20).

**Venter** — (710–800 (800) long; 515–570 (545) wide) colorless. Gnathosomal bay (182.5–200 (190) long; 80–97.5 (97.5) wide). Cxgl-4 subapical. **Medial suture** (10–15 (15) long). **Genital plates** (160–175 (175) long; 145–150 (150) wide). Additional measurements: Cx-1 (310–345 (345) long (total); 125–155 (155) long (medial)); Cx-3 (350–375 (375) wide); anterior venter (155–185 (185) long). Ventral proportions: gnathosomal bay length/width 1.95–2.42 (1.95); anterior venter/genital field length 0.97–1.09 (1.06); anterior venter length/genital field width 1.07–1.23 (1.23); anterior venter/medial suture 10.33–17.50 (12.33).

**Male (Figure 46)** (n = 4) (allotypic measurements in parentheses when available) with characters of the genus with following specifications.

**Dorsum** — (545–570 (570) long; 410–425 (425) wide) circular with faint reddish-purple coloration separated into anterior and posterior portions. Anterio-medial platelets (115–125 (122.5) long; 52.5–58.75 (58.75) wide). Anterio-lateral platelets (175–188.75 (188.75) long; 67.5–75 (75) wide) free from dorsal plate. Dgl-4 approximately halfway between the edge of the dorsum and the muscle scars (distance between Dgl-4 255–290 (275)). Dorsal plate proportions: dorsum length/width 1.31–1.34 (1.34); dorsal width/distance between Dgl-4 1.45–1.65 (1.55); anterio-medial platelet length/width 2.09–2.22 (2.09); anterio-lateral platelet length/width 2.50–2.78 (2.52); anterio-lateral/anterio-medial length 1.40–1.63 (1.54).

**Gnathosoma — Subcapitulum** (320–335 (335) long (ventral); 247.5–255 (255) long (dorsal); 120–122.5 (120) tall) colorless, occasionally with faint reddish-purple coloration. Rostrum (132.5–137.5 (137.5) long; 40–45 (45) wide). Chelicerae (310–330 (330) long) with curved fangs (60–65 (60) long). Subcapitular proportions: ventral length/height 2.67–2.79 (2.79); rostrum length/width 3.06–3.31 (3.06). **Pedipalps** with tuberculate ventral extensions on femora and genua. Palpomeres: trochanter (42.5–46.25 (45) long); femur (117.5–122.5 (120) long); genu (67.5–70 (67.5) long); tibia (90–92.5 (91.25) long; 21.25–22.5 (22.5) wide); tarsus (20–20 (20) long). Palpomere proportions: femur/genu 1.74–1.78 (1.78); tibia/femur 0.76–0.77 (0.76); tibia length/width 4.00–4.35 (4.06).

**Venter** — (655–705 (705) long; 460–470 (470) wide) colorless. Gnathosomal bay (165–165 (165) long; 70–80 (75) wide). Cxgl-4 subapical. **Medial suture** (50–55 (55) long). **Genital plates** (145–155 (155) long; 115–120 (120) wide). Additional measurements: Cx-1 (290–315 (290) long (total); 130–150 (130) long (medial)); Cx-3 (320–340 (340) wide); anterior venter (210–225 (225) long). Ventral proportions: gnathosomal bay length/width 2.06–2.36 (2.20); anterior venter/genital field length 1.42–1.55 (1.45); anterior venter length/genital field width 1.75–1.91 (1.88); anterior venter/medial suture 4.00–4.20 (4.09).

**Immatures** unknown.

######## Etymology.

Specific epithet (*daemon*) refers to the diagnostic red coloration on the anterio-medial platelets, which resemble the red eyes of an evil demon (*daemon*, L. originally benevolent or benign nature spirits, but were characterized as dangerous or evil by the writings of Plato and later used in Christian literature, popularizing the idea of demons as evil; noun in apposition).

######## Distribution.

**Figure 44.**
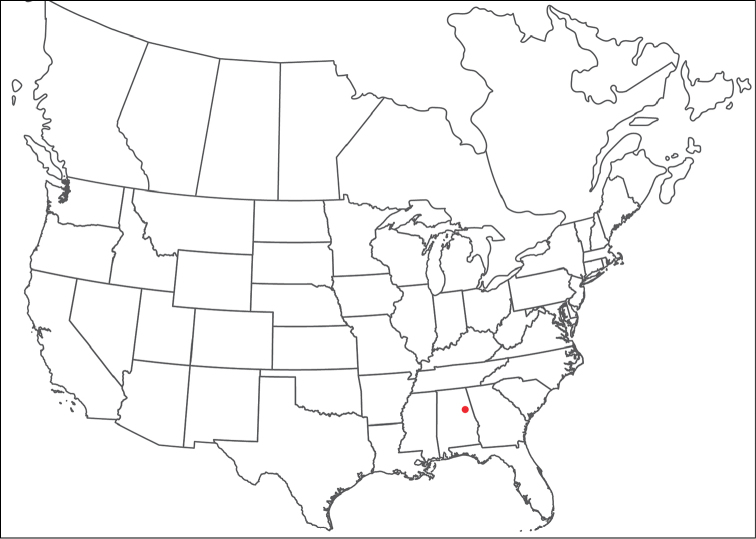
*Torrenticola daemon* sp. n. distribution.

Alabama (Figure 44).

**Figure 45.**
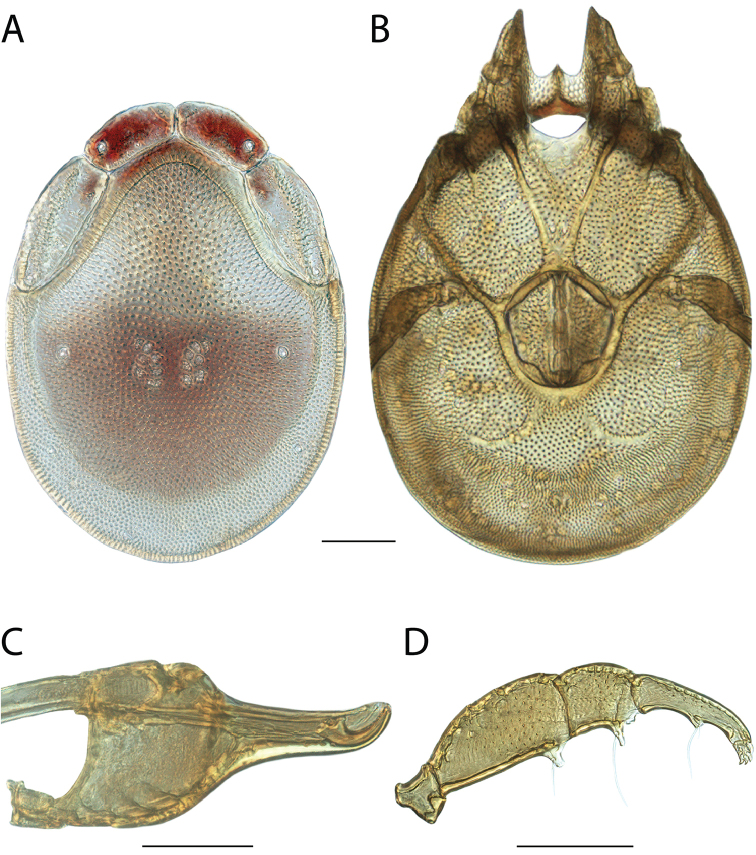
*Torrenticola daemon* sp. n. female: **A** dorsal plates **B** venter (legs removed) **C** subcapitulum **D** pedipalp (setae not accurately depicted). Scale = 100 µm.

**Figure 46.**
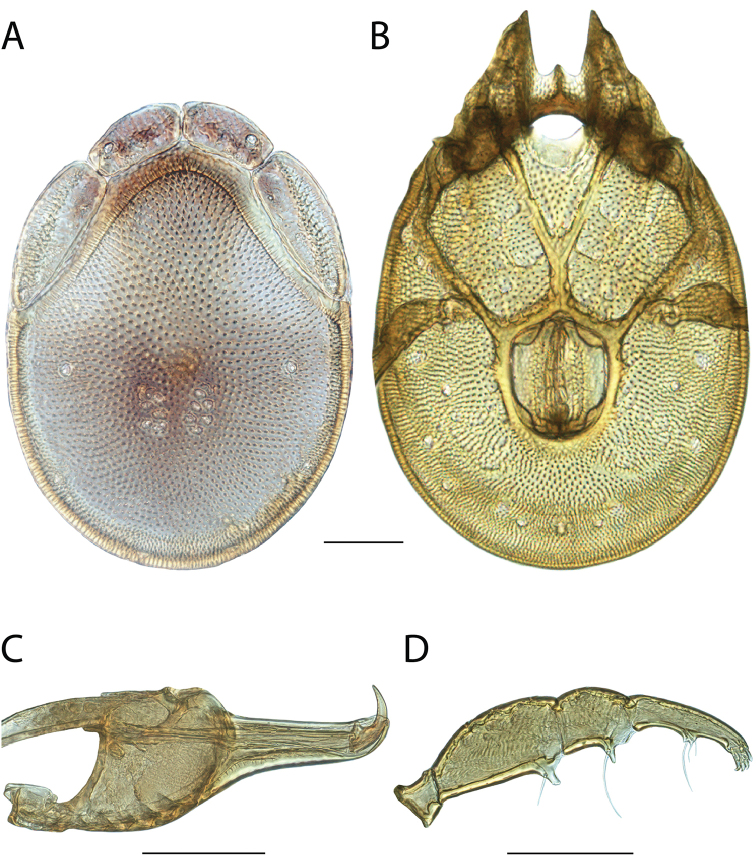
*Torrenticola daemon* sp. n. male: **A** dorsal plates **B** venter (legs removed) **C** subcapitulum **D** pedipalp (setae not accurately depicted). Scale = 100 µm.

######## Remarks.

Unfortunately, we were unable to acquire fresh material of *Torrenticola daemon* and therefore this species is not included in our phylogenetic analyses. However, we were able to examine morphology with material preserved in GAW. The overall appearance, elongate subcapitular rostra, elongate pedipalpal tibiae, and Dgl-4 close to the muscle scars, are consistent with placing this species in the Raptor Complex and Raptor Identification Group.

####### Torrenticola danielleae

Taxon classificationAnimaliaTrombidiformesTorrenticolidae

Fisher & Dowling sp. n.

http://zoobank.org/386C2F49-C148-4A74-BCC2-22BD45B18FAA

######## Material examined.

HOLOTYPE (♀): from USA, Georgia, Floyd County, The Pocket Campground beside road from Everett Springs to Villanow, (34°35'35"N, 85°5'5"W), 2 July 1990, by IM Smith, IMS900073A.

PARATYPES (4 ♀; 5 ♂): **Georgia, USA**: 2 ♀ and 1 ♂ from Chattooga County, beside road from Everett Springs to Villanow 1.8 km south of The Pocket Recreation Area, (34°34'34"N, 80°5'5"W), 4 July 1990, by IM Smith, IMS900076 • 1 ♂ (ALLOTYPE) from Floyd County, The Pocket Campground beside road from Everett Springs to Villanow, (34°35'35"N, 85°5'5"W), 2 July 1990, by IM Smith, IMS900073A • 2 ♀ and 3 ♂ from Floyd County, The Pocket Campground beside road from Everett Springs to Villanow, (34°35'35"N, 85°5'5"W), 2 July 1990, by IM Smith, IMS900073A

######## Type deposition.

Holotype (♀), allotype (♂), and some paratypes (2 ♀; 2 ♂) deposited in the CNC; other paratypes (2 ♀; 2 ♂) deposited in the ACUA.

######## Diagnosis.

*Torrenticola danielleae* are similar to other members of the Raptor Group (*T. daemon*, *T. elusiva*, *T. gnoma*, *T. irapalpa*, *T. ivyae*, *T. longitibia*, *T. mjolniri*, *T. racupalpa*, and *T. raptor*) in having round bodies; Dgl-4 close to muscles scars; long, thin subcapitular rostra; and long, thin pedipalp tibiae. *T. danielleae* can be differentiated from all other Raptor Group by having Dgl-4 closer to the edge of the dorsum (dorsum width/distance between Dgl-4 ♀ = 1.57–1.70 in *T. danielleae*, 1.80–3.29 in others; ♂ = 1.42–1.52 in *T. danielleae*, 1.58–2.73 in others), except *T. daemon* (♀ = 1.59–1.67 ♂ = 1.45–1.65). *T. danielleae* can be differentiated from *T. daemon* by having a more elongate rostrum (length/width = 3.43–3.75 in *T. danielleae*, 2.91–3.31 in *T. daemon*) and dorsal pattern.

######## Description.

**Female (Figure 48)** (n = 5) (holotype measurements in parentheses when available) with characters of the genus with following specifications.

**Dorsum** — (635–680 (635) long; 510–545 (510) wide) circular with reddish-purple coloration posteriorly extending in a strip anteriorly to the edge of the dorsal plate. Anterio-medial platelets (135–155 (147.5) long; 55–65 (62.5) wide). Anterio-lateral platelets (170–195 (180) long; 70–80 (80) wide) free from dorsal plate. Dgl-4 approximately halfway between the edge of the dorsum and the muscle scars (distance between Dgl-4 320–335 (320)). Dorsal plate proportions: dorsum length/width 1.20–1.30 (1.25); dorsal width/distance between Dgl-4 1.57–1.7 (1.59); anterio-medial platelet length/width 2.25–2.73 (2.36); anterio-lateral platelet length/width 2.25–2.79 (2.25); anterio-lateral/anterio-medial length 1.15–1.44 (1.22).

**Gnathosoma — Subcapitulum** (340–370 (365) long (ventral); 260–280 (270) long (dorsal); 135–145 (145) tall) colorless. Rostrum (150–160 (155) long; 40–45 (42.5) wide). Chelicerae (350–380 (380) long) with curved fangs (50–62.5 (62.5) long). Subcapitular proportions: ventral length/height 2.52–2.65 (2.52); rostrum length/width 3.44–3.75 (3.65). **Pedipalps** with long, tuberculate ventral extensions on femora and genua. Palpomeres: trochanter (40–45 (42.5) long); femur (127.5–141.25 (137.5) long); genu (67.5–75 (75) long); tibia (92.5–107.5 (102.5) long; 20–22.5 (21.25) wide); tarsus (17.5–20 (20) long). Palpomere proportions: femur/genu 1.77–1.89 (1.83); tibia/femur 0.73–0.76 (0.75); tibia length/width 4.63–5.00 (4.82).

**Venter** — (780–835 (780) long; 560–610 (560) wide) with reddish-purple coloration, occasionally faint. Gnathosomal bay (170–185 (185) long; 75–85 (80) wide). Cxgl-4 subapical. **Medial suture** (25–27.5 (27.5) long). **Genital plates** (165–185 (185) long; 155–160 (160) wide). Additional measurements: Cx-1 (300–340 (315) long (total); 135–155 (135) long (medial)); Cx-3 (345–360 (355) wide); anterior venter (170–195 (185) long). Ventral proportions: gnathosomal bay length/width 2.00–2.31 (2.31); anterior venter/genital field length 1.00–1.12 (1.00); anterior venter length/genital field width 1.10–1.24 (1.16); anterior venter/medial suture 6.73–7.80 (6.73).

**Male (Figure 49)** (n = 5) (allotypic measurements in parentheses when available) with characters of the genus with following specifications.

**Dorsum** — (490–530 (510) long; 370–385 (370) wide) circular with reddish-purple coloration posteriorly extending in a strip anteriorly to the edge of the dorsal plate. Anterio-medial platelets (115–125 (120) long; 45–52.5 (52.5) wide). Anterio-lateral platelets (150–175 (150) long; 57.5–65 (65) wide) free from dorsal plate. Dgl-4 approximately halfway between the edge of the dorsum and the muscle scars (distance between Dgl-4 250–265 (260)). Dorsal plate proportions: dorsum length/width 1.27–1.39 (1.38); dorsal width/distance between Dgl-4 1.42–1.52 (1.42); anterio-medial platelet length/width 2.29–2.72 (2.29); anterio-lateral platelet length/width 2.31–2.92 (2.31); anterio-lateral/anterio-medial length 1.25–1.43 (1.25).

**Gnathosoma — Subcapitulum** (285–295 (290) long (ventral); 210–220 (215) long (dorsal); 100–105 (105) tall) colorless. Rostrum (120–125 (120) long; 35–35 (35) wide). Chelicerae (270–300 (280) long) with curved fangs (40–50 (50) long). Subcapitular proportions: ventral length/height 2.74–2.85 (2.76); rostrum length/width 3.43–3.57 (3.43). **Pedipalps** with long, tuberculate ventral extensions on femora and genua. Palpomeres: trochanter (33.75–37.5 (36.25) long); femur (100–110 (107.5) long); genu (60–62.5 (62.5) long); tibia (80–82.5 (82.5) long; 20–20 (20) wide); tarsus (17.5–20 (17.5) long). Palpomere proportions: femur/genu 1.67–1.79 (1.72); tibia/femur 0.74–0.83 (0.77); tibia length/width 4.00–4.13 (4.13).

**Venter** — (620–670 (650) long; 410–430 (420) wide) with reddish-purple coloration, occasionally faint. Gnathosomal bay (135–145 (142.5) long; 62.5–70 (67.5) wide). Cxgl-4 subapical. **Medial suture** (60–65 (60) long). **Genital plates** (132.5–145 (140) long; 102.5–107.5 (105) wide). Additional measurements: Cx-1 (260–280 (280) long (total); 125–135 (135) long (medial)); Cx-3 (290–310 (310) wide); anterior venter (205–220 (205) long). Ventral proportions: gnathosomal bay length/width 2.07–2.23 (2.11); anterior venter/genital field length 1.46–1.57 (1.46); anterior venter length/genital field width 1.91–2.05 (1.95); anterior venter/medial suture 3.15–3.52 (3.42).

**Immatures** unknown.

######## Etymology.

Specific epithet (*danielleae*) named in honor of Danielle Fisher—lab technician, environmental educator, colleague, friend, wife (of JRF), and mother of Ivy, our beautiful daughter—for her tireless and immense contributions to this research, and for bettering the lives of all those around her. Thank you, Danielle.

######## Distribution.

**Figure 47.**
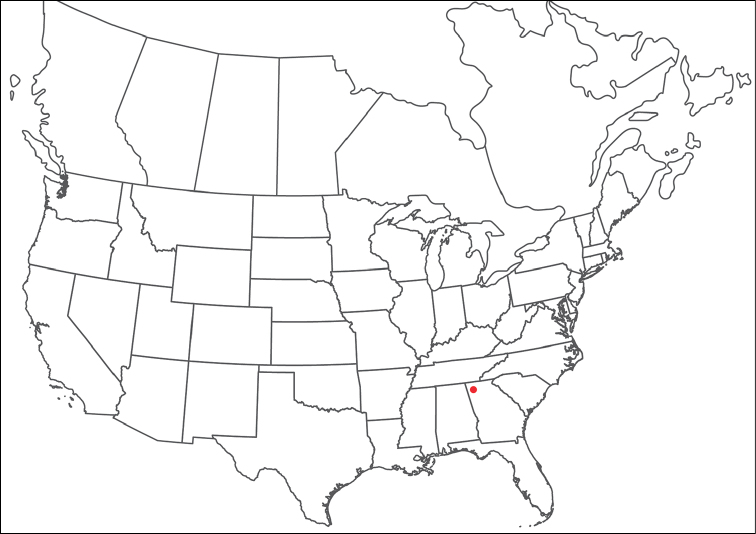
*Torrenticola danielleae* sp. n. distribution.

Southern Appalachians, northeastern Georgia (Figure 47).

**Figure 48.**
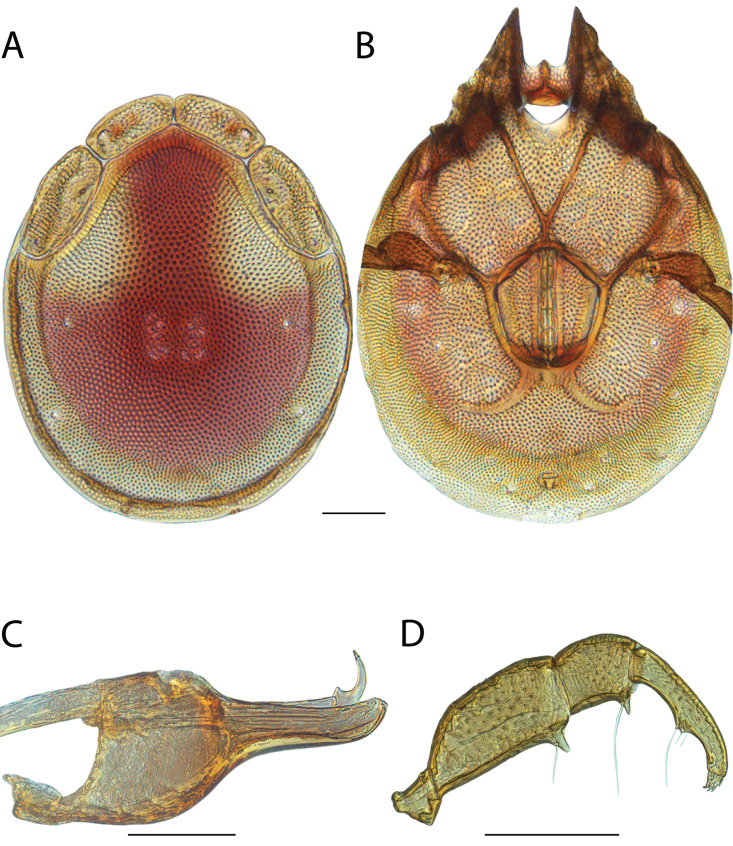
*Torrenticola danielleae* sp. n. female: **A** dorsal plates **B** venter (legs removed) **C** subcapitulum **D** pedipalp (setae not accurately depicted). Scale = 100 µm.

**Figure 49.**
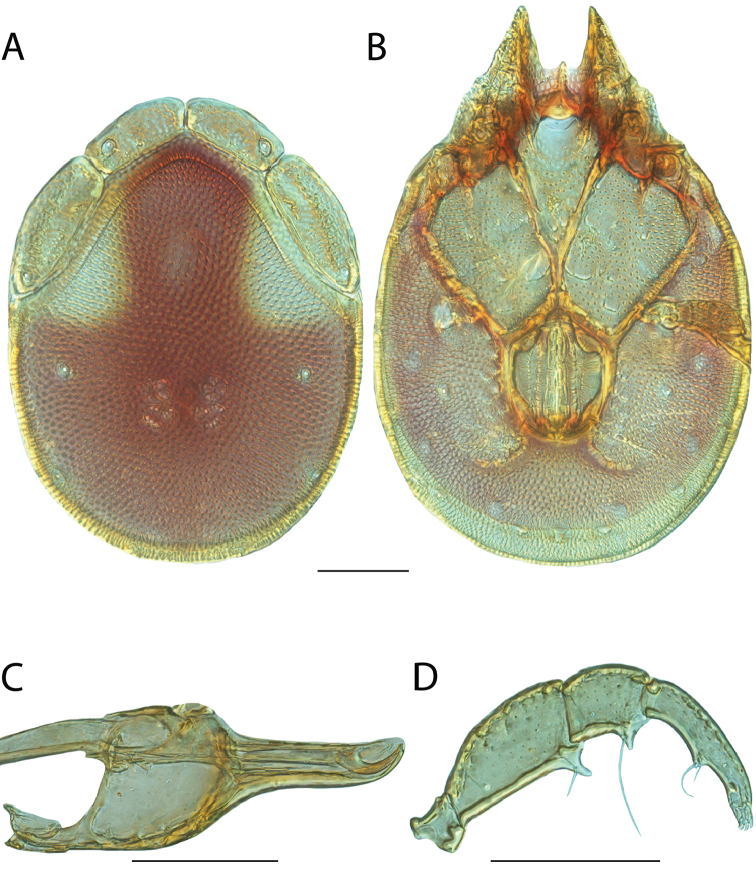
*Torrenticola danielleae* sp. n. male: **A** dorsal plates **B** venter (legs removed) **C** subcapitulum **D** pedipalp (setae not accurately depicted). Scale = 100 µm.

######## Remarks.

Unfortunately, we were unable to acquire fresh material of *Torrenticola danielleae* and therefore this species is not included in our phylogenetic analyses. However, we were able to examine morphology with material preserved in GAW. The overall appearance, elongate subcapitular rostra, and elongate pedipalpal tibiae, are consistent with placing this species in the Raptor Complex and the Raptor Identification Group.

####### Torrenticola delicatexa

Taxon classificationAnimaliaTrombidiformesTorrenticolidae

Habeeb, 1955

T. amplexa delicatexa : Habeeb 1955: 4; 1957: 1.
T. delicatexa : Habeeb 1961: 2; 1967: 3 • Viets 1987: 759.

######## Material examined.

**Type series.** HOLOTYPE (♂): from USA, New Jersey, Sussex County, Flatbrookeville, Flatbrook, 12 Oct 1953, by H Habeeb, HH530113.

PARATYPES (1 ♀; 0 ♂): **New Jersey, USA**: 1 ♀ (ALLOTYPE) from Sussex County, Flatbrookeville, Flatbrook, 12 Oct 1953, by H Habeeb, HH530113.

OTHER MATERIAL (19 ♀; 7 ♂): **North Carolina, USA**: 1 ♀ from Haywood County, Great Smokey Mountains National Park, Cataloochee River (35°38'45"N, 83°4'34"W), 6 Sep 2009, by IM Smith, IMS090099 • **Maine, USA**: 1 ♀ and 1 ♂ from Franklin County, Small Falls picnic area beside Route 4, Sandy River (44°52'N, 70°31'W), 5 Jul 1989, by IM Smith, IMS890069 • **New Hampshire, USA**: 1 ♀ and 1 ♂ from Woodstock County, beside Route 118, Jackman Brook (44°0'N, 71°45'W), 11 Sep 1992, by IM Smith, IMS920036 • **Pennsylvania, USA**: 1 ♀ from Fayette County, Dunbar Creek (39°57'50"N, 79°35'8.70"W), 10 Aug 2014, by MJ Skvarla, MS 14-0810-001 • 2 ♀ and 2 ♂ from Fayette County, Ohiopyle State Park, Laurel Run (39°50'58"N, 79°30'51"W), 10 Aug 2014, by MJ Skvarla, MS 14-0810-005 • 2 ♀ from Somerset County, Laurel Hill State Park, Laurel Hill Creek (40°1'6"N, 79°14'4"W), 8 Aug 2014, by MJ Skvarla, MS 14-0808-001 • **Quebec, Canada**: 1 ♀ from Stanstead County, 1 kilometer south of Rock Island, Tomifobia River, Tompkin Stream, (45°0'31"N, 72°7'6"W), 20 Aug 1996, by IM Smith & M MacKenzie, IMS960056 • **Tennessee, USA**: 1 ♀ from Blount County, Great Smokey Mountains National Park, Abrams River (35°35'31"N, 83°51'21"W), 17 Sep 2010, by IM Smith, IMS100141 • 1 ♀ from Blount County, Great Smokey Mountains National Park, Little River (35°40'55"N, 83°39'6"W), 8 Sep 2009, by IM Smith, IMS090102 • 2 ♀ from Sevier County, Great Smokey Mountains National Park, middle prong of Little Pigeon River (35°43'34"N, 83°24'2"W), 10 Sep 2010, by IM Smith, IMS100127 • 1 ♀ from Sevier County, Great Smokey Mountains National Park, middle prong of Little Pigeon River (35°43'34"N, 83°24'2"W), 10 Sep 2010, by IM Smith, IMS100128 • 1 ♀ from Sevier County, Great Smokey Mountains National Park, Sugarlands Nature Trail, spring (35°40'47"N, 83°31'52"W), 18 Sep 2010, by IM Smith, IMS100147 • **South Carolina, USA**: 1 ♀ from Greenville County, Matthews Creek, 24 Apr 2014, by D Eargle, JRF 14-0424-001 • **Vermont, USA**: 1 ♀ and 1 ♂ from Addison County, beside road from Lincoln, Middlebury River, (44°0'N, 73°1'W), 6 Jul 1989, by IM Smith, IMS890075 • **Virginia, USA**: 1 ♀ and 1 ♂ from Montgomery County, Blacksburg, beside Route 321 at Caldwell, Craig Creek (37°20'0"N, 80°20'0"W), 12 Jul 1990, by IM Smith, IMS900089A • 1 ♀ and 1 ♂ from Patrick County, Round Meadow Creek (36°42'59"N, 80°25'29"W), 10 Jun 2006, by IM Smith, IMS060005A.

######## Type deposition.

Holotype (♀) and allotype (♂) deposited in CNC.

######## Diagnosis.

*Torrenticola delicatexa* are similar to other members of Rusetria “Eastern 2-Plates” group (*T. biscutella*, *T. caerulea*, *T. feminellai*, *T. indistincta*, *T. malarkeyorum*, *T. microbiscutella*, *T. pendula*, *T. sellersorum*, *T. tysoni*, *T. ululata*, and*T. whitneyae*) in having anterio-lateral platelets fused to the dorsal plate, having dorsal coloration separated into anterior and posterior portions (except *T. indistincta* and *T. ululata*), and being distributed in the east. *T. delicatexa* can be differentiated from *T. ululata*, *T. indistincta*, and *T. feminellai* by dorsal coloration and pattern. *T. delicatexa* can be differentiated from *T. tysoni* by having a stockier rostrum (length/width = 2.33–3.00 in *T. delicatexa*, 3.06–3.50 in *A34*). *T. delicatexa* can be differentiated from *T. pendula* by having a stockier gnathosomal bay (length/width = 1.28–2.22 in *T. delicatexa*, 2.42–2.90 in *T. pendula*), and a longer dorsum (♀ = 560–620 in *T. delicatexa*, 630–650 in *T. pendula*; ♂ = 420–465 in *T. delicatexa*, 500 in *T. pendula*). *T. delicatexa* can be differentiated from *T. microbiscutella* by having a less elongate dorsum (length/width = 1.38–1.56 in *T. delicatexa*, 1.63–1.75 in *T. microbiscutella*). Female *T. delicatexa* can be differentiated from female *T. malarkeyorum*, *T. biscutella*, and *T. caerulea* by having a longer genital field (175–185 in *T. delicatexa*, 153–170 in others). Male *T. delicatexa* can be differentiated from male *T. biscutella* by having a more ovoid dorsum (length/width = 1.44–1.56 in *T. delicatexa*; 1.37–1.42 in *T. biscutella*). Female *T. delicatexa* can be differentiated from female *T. sellersorum* by having a slightly more ovoid dorsum (length/width = 1.38–1.44 in *T. delicatexa*, 1.23–1.37 in *T. sellersorum*). Male *T. delicatexa* do not have any measurement differences with male *T. malarkeyorum*, *T. caerulea*, and *T. sellersorum*; however, they can be differentiated by dorsal coloration. *T. delicatexa* can be differentiated from *T. whitneyae* by having a slightly more ovoid dorsum (length/width ♀ = 1.38–1.44 in *T. delicatexa*, 1.26–1.38 in *T. whitneyae*; ♂ = 1.44–1.56 in *T. delicatexa*, 1.35–1.37 in *T. whitneyae*) and by dorsal coloration. Additionally, male *T. delicatexa* can be differentiated from male *T. whitneyae* by having more elongate pedipalpal tibiae (length/width = 2.89–3.63 in *T. delicatexa*, 2.48–2.70 in *T. whitneyae*).

######## Re-description.

**Male (Figure 51)** (n = 5) (holotype measurements in parentheses when available) with characters of the genus with following specifications.

**Dorsum** — (410–465 (465) long; 270–320 (320) wide) ovoid with highly variable coloration, reddish-purple to purple (occasionally bluish-purple) separated into anterior and posterior portions. Anterio-medial platelets (83.75–102.5 (102.5) long; 30–32.5 (32.5) wide). Anterio-lateral platelets (122.5–132.5 (132.5) long; 40–55 (52.5) wide) fused to dorsal plate. Dgl-4 much closer to the edge of the dorsum than to the muscle scars (distance between Dgl-4 205–255 (255)). Dorsal plate proportions: dorsum length/width 1.44–1.56 (1.45); dorsal width/distance between Dgl-4 1.23–1.37 (1.25); anterio-medial platelet length/width 2.79–3.17 (3.15); anterio-lateral platelet length/width 2.36–3.06 (2.52); anterio-lateral/anterio-medial length 1.29–1.46 (1.29).

**Gnathosoma — Subcapitulum** (235–247.5 (247.5) long (ventral); 172.5–193.75 (194) long (dorsal); 97.5–107.5 (107.5) tall) colorless. Rostrum (90–100 (100) long; 32.5–37.5 (37.5) wide). Chelicerae (220-230 long) with curved fangs (45–52.5 (50) long). Subcapitular proportions: ventral length/height 2.30–2.45 (2.30); rostrum length/width 2.67–2.81 (2.67). **Pedipalps** with tuberculate ventral extensions on femora and genua. Palpomeres: trochanter (33.75–40 (40) long); femur (82.5–92.5 (92.5) long); genu (50–55 (55) long); tibia (62.5–72.5 (65) long; 20–22.5 (22.5) wide); tarsus (15–17.5 (17.5) long). Palpomere proportions: femur/genu 1.59–1.75 (1.68); tibia/femur 0.70–0.83 (0.70); tibia length/width 2.89–3.63 (2.89).

**Venter** — (490–540 (540) long; 311–435 (435) wide) usually colorless; occasionally with faint reddish-purple coloration. Gnathosomal bay (120–135 (135) long; 57.5–72.5 (72.5) wide). Cxgl-4 subapical. **Medial suture** (55–75 (62.5) long). **Genital plates** (105–110 (106.25) long; 95–102.5 (102.5) wide). Additional measurements: Cx-1 (220–235 (235) long (total); 78–100 (100) long (medial)); Cx-3 (240–297.5 (297.5) wide); anterior venter (160–175 (170) long). Ventral proportions: gnathosomal bay length/width 1.86–2.22 (1.86); anterior venter/genital field length 1.52–1.60 (1.60); anterior venter length/genital field width 1.64–1.79 (1.66); anterior venter/medial suture 2.33–2.91 (2.72).

**Female (Figure 52)** (n = 6) (allotypic measurements in parentheses when available) with characters of the genus with following specifications.

**Dorsum** — (560–620 (620) long; 390–440 (435) wide) ovoid with highly variable coloration, reddish-purple to purple (occasionally bluish-purple) separated into anterior and posterior portions. Anterio-medial platelets (125–140 (140) long; 36.25–45 (42.5) wide). Anterio-lateral platelets (152.5–172.5 (162.5) long; 57.5–70 (70) wide) fused to dorsal plate. Dgl-4 much closer to the edge of the dorsum than to the muscle scars (distance between Dgl-4 295–350 (330)). Dorsal plate proportions: dorsum length/width 1.38–1.44 (1.43); dorsal width/distance between Dgl-4 1.26–1.33 (1.32); anterio-medial platelet length/width 2.83–3.52 (3.29); anterio-lateral platelet length/width 2.26–2.88 (2.32); anterio-lateral/anterio-medial length 1.16–1.33 (1.16).

**Gnathosoma — Subcapitulum** (305–345 (345) long (ventral); 216–260 (260) long (dorsal); 145–165 (160) tall) colorless. Rostrum (115–135 (135) long; 45–55 (45) wide). Chelicerae (312–350 (350) long) with curved fangs (54–75 (70) long). Subcapitular proportions: ventral length/height 1.97–2.22 (2.16); rostrum length/width 2.33–3.00 (3.00). **Pedipalps** with tuberculate ventral extensions on femora and genua. Palpomeres: trochanter (42.5–52.5 (47.5) long); femur (105–130 (128.75) long); genu (67.5–75 (72.5) long); tibia (81.25–90 (87.5) long; 22.5–30 (30) wide); tarsus (20–22.5 (22.5) long). Palpomere proportions: femur/genu 1.56–1.78 (1.78); tibia/femur 0.65–0.77 (0.68); tibia length/width 2.92–3.61 (2.92).

**Venter** — (640–690 (690) long; 431–540 (540) wide) usually colorless; occasionally with faint reddish-purple coloration. Gnathosomal bay (132.5–175 (157.5) long; 85–117.5 (117.5) wide). Cxgl-4 subapical. **Medial suture** absent. **Genital plates** (175–197.5 (197.5) long; 150–172.5 (170) wide). Additional measurements: Cx-1 (216–295 (295) long (total); 93–135 (135) long (medial)); Cx-3 (304–400 (400) wide); anterior venter (115–135 (135) long). Ventral proportions: gnathosomal bay length/width 1.28–2.06 (1.34); anterior venter/genital field length 0.64–0.76 (0.68); anterior venter length/genital field width 0.71–0.83 (0.79).

**Immatures** unknown.

######## Etymology.

Habeeb (1955) did not offer an explanation for the specific epithet (*delicatexa*) and we are unable to offer helpful speculation.

######## Distribution.

**Figure 50.**
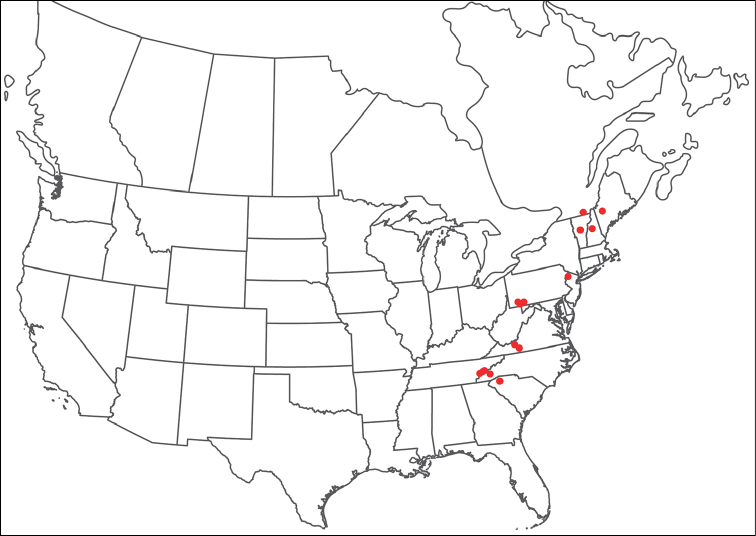
*Torrenticola delicatexa* distribution.

Appalachians (Figure 50).

**Figure 51.**
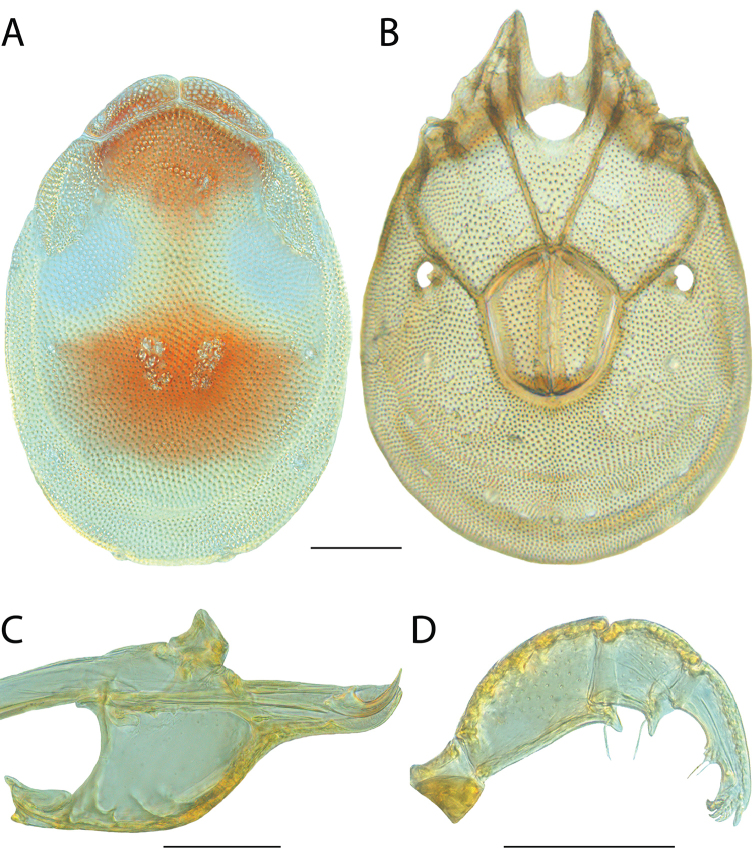
*Torrenticola delicatexa* female: **A** dorsal plates **B** venter (legs removed) **C** subcapitulum **D** pedipalp (setae not accurately depicted). Scale = 100 µm.

**Figure 52.**
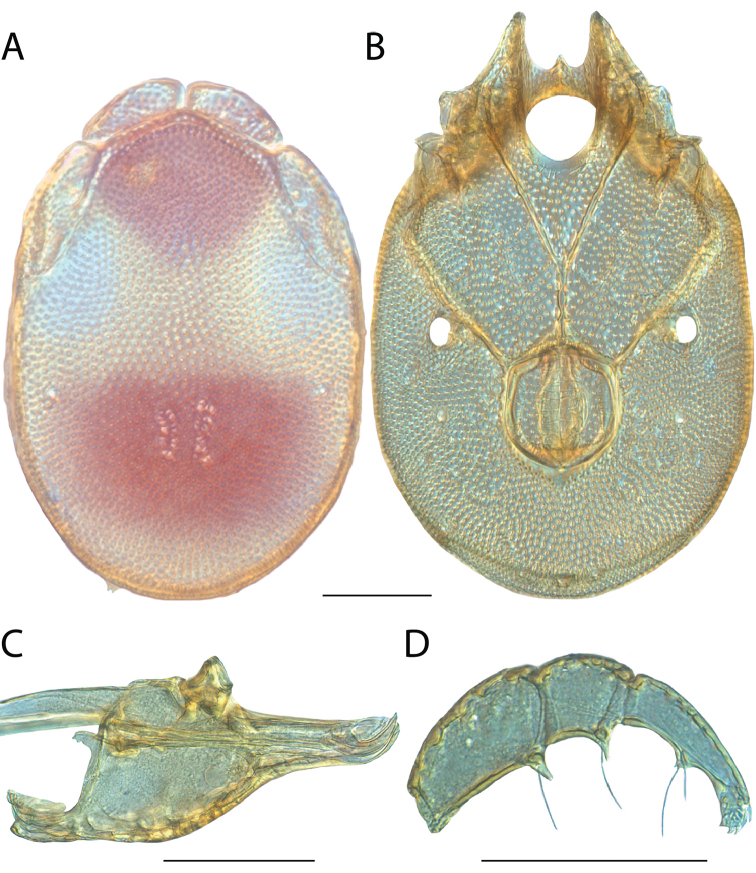
*Torrenticola delicatexa* male: **A** dorsal plates **B** venter (legs removed) **C** subcapitulum **D** pedipalp (setae not accurately depicted). Scale = 100 µm.

######## Remarks.

In all analyses, *Torrenticola delicatexa* groups with members of the Rusetria Complex with high support and specimens of this species were less than 2% different in COI sequence from each other. However, one specimen from Tennessee (DNA#1839) was 5% different; this specimen was collected from the same river as the other specimens and was indistinguishable morphologically. We refrain from speculating on this COI variation, but consider this specimen as an outlier, and thus within our hypothesis for *T. delicatexa*. The position of this species was only strongly supported in our combined analysis, where it is recovered as sister to two other species: *T. ululata* and *T. glomerabilis*. However, *T. delicatexa* does not resemble these species morphologically, and instead is quite similar to three more distantly-related species (*T. biscutella*, *T. malarkeyorum*, and *T. caerulea*).

This species hypothesis is supported by low COI divergence within the species (0–2%) and high divergence between species (3–15%), and by the morphological characters outlined in the diagnosis.

####### Torrenticola dentirostra

Taxon classificationAnimaliaTrombidiformesTorrenticolidae

Fisher & Dowling sp. n.

http://zoobank.org/4D2ADBB1-C62F-4080-8E5E-3CA8F7BB626F

######## Material examined.

HOLOTYPE (♀): from USA, Georgia, Chattooga County, beside road from Everett Springs to Villanow 1.4 km south of The Pocket Recreation Area, 4 July 1990, by IM Smith, IMS900077.

PARATYPES (4 ♀; 2 ♂): **Georgia, USA**: 1 ♂ (ALLOTYPE) from Chattooga County, beside road from Everett Springs to Villanow 1.4 km south of The Pocket Recreation Area, 4 July 1990, by IM Smith, IMS900077 • 2 ♀ and 1 ♂ from Chattooga County, beside road from Everett Springs to Villanow 1.4 km south of The Pocket Recreation Area, 4 July 1990, by IM Smith, IMS900077 • **Tennessee, USA**: 1 ♀ from Monroe County, beside Forest Route 35, 2.0 km northeast of road from Rt. 165 to Miller Chapel Baptist Church, (35°21'21"N, 84°9'9"W), 5 July 1990, by IM Smith, IMS900078 • **Virginia, USA**: 1 ♀ from Alleghany County, Covington; beside Rt. 18, 0.5 km north of Rt. 657, (37°44'44"N, 80°2'2"W), 13 July 1990, by IM Smith, IMS900091A.

######## Type deposition.

Holotype (♀), allotype (♂), and some paratypes (2 ♀) deposited in the CNC; other paratypes (2 ♀; 1 ♂) deposited in the ACUA.

######## Diagnosis.

*Torrenticola dentirostra* are similar to other members of the Nigroalba Group (*T. flangipalpa*, *T. nigroalba*, and *T. solisorta*) in being small, slightly elongate, and having purple dorsal coloration restricted posteriorly. *T. dentirostra* can be differentiated from most Torrenticola (except *T. erectirostra*, *T. karambita*, and *T. robisoni*) by having a dentate bump midway on the dorsal edge of the rostrum. *T. dentirostra* can be differentiated from *T. karambita*, *T. erectirostra*, and *T. robisoni* by having a straight rostrum (others have upturned rostra) and by dorsal pattern.

######## Description.

**Female (Figure 54)** (n = 5) (holotype measurements in parentheses when available) with characters of the genus with following specifications.

**Dorsum** — (470–560 (470) long; 340–390 (340) wide) ovoid with faint purple coloration restricted posteriorly. Anterio-medial platelets (107.5–120 (107.5) long; 45–47.5 (45) wide). Anterio-lateral platelets (157.5–177.5 (157.5) long; 55–62.5 (55) wide) free from dorsal plate. Dgl-4 closer to the edge of the dorsum than to the muscle scars (distance between Dgl-4 240–275 (240)). Dorsal plate proportions: dorsum length/width 1.38–1.45 (1.38); dorsal width/distance between Dgl-4 1.38–1.43 (1.42); anterio-medial platelet length/width 2.32–2.67 (2.39); anterio-lateral platelet length/width 2.84–3.05 (2.86); anterio-lateral/anterio-medial length 1.43–1.59 (1.47).

**Gnathosoma — Subcapitulum** (275–330 (275) long (ventral); 200–247.5 (200) long (dorsal); 77.5–102.5 (77.5) tall) colorless. Rostrum (100–120 (100) long; 35–40 (35) wide) with dentate bump midway on dorsal edge. Chelicerae (255–320 (255) long) with curved fangs (45–55 (45) long). Subcapitular proportions: ventral length/height 3.22–3.73 (3.55); rostrum length/width 2.80–3.00 (2.86). **Pedipalps** with tuberculate ventral extensions dentate apically on femora and genua. Palpomeres: trochanter (30–35 (30) long); femur (92.5–110 (92.5) long); genu (55–67.5 (55) long); tibia (80–92.5 (80) long; 17.5–20 (17.5) wide); tarsus (12.5–15 (13.75) long). Palpomere proportions: femur/genu 1.62–1.68 (1.68); tibia/femur 0.84–0.88 (0.86); tibia length/width 4.57–4.93 (4.57).

**Venter** — (610–685 (610) long; 390–425 (390) wide) mostly colorless with faint purple genital plates. Gnathosomal bay (85–110 (85) long; 60–80 (60) wide). Cxgl-4 far from apex and ventral. **Medial suture** (60–70 (60) long). **Genital plates** (142.5–165 (142.5) long; 120–135 (120) wide). Additional measurements: Cx-1 (230–260 (230) long (total); 140–160 (140) long (medial)); Cx-3 (270–300 (270) wide); anterior venter (210–240 (210) long). Ventral proportions: gnathosomal bay length/width 1.30–1.50 (1.42); anterior venter/genital field length 1.42–1.64 (1.47); anterior venter length/genital field width 1.70–1.83 (1.75); anterior venter/medial suture 3.29–3.69 (3.50).

**Male (Figure 55)** (n = 2) (allotypic measurements in parentheses when available) with characters of the genus with following specifications.

**Dorsum** — (480–505 (505) long; 360–360 (360) wide ovoid with faint purple coloration restricted posteriorly. Anterio-medial platelets (110–110 (110) long; 40–42.5 (42.5) wide). Anterio-lateral platelets (162.5–165 (165) long; 50–52.5 (52.5) wide) free from dorsal plate. Dgl-4 closer to the edge of the dorsum than to the muscle scars (distance between Dgl-4 240–245 (240)). Dorsal plate proportions: dorsum length/width 1.33–1.40 (1.40); dorsal width/distance between Dgl-4 1.47–1.50 (1.50); anterio-medial platelet length/width 2.59–2.75 (2.59); anterio-lateral platelet length/width 3.14–3.25 (3.14); anterio-lateral/anterio-medial length 1.48–1.50 (1.50).

**Gnathosoma — Subcapitulum** (285–287.5 (287.5) long (ventral); 210–217.5 (217.5) long (dorsal); 77.5–80 (77.5) tall) colorless. Rostrum (100–105 (100) long; 32.5–35 (32.5) wide) with dentate bump midway on dorsal edge. Chelicerae (255–265 (265) long) with curved fangs (40–45 (45) long). Subcapitular proportions: ventral length/height 3.56–3.71 (3.71); rostrum length/width 3.00–3.08 (3.08). **Pedipalps** with tuberculate ventral extensions dentate apically on femora and genua. Palpomeres: trochanter (30–32.5 (32.5) long); femur (90–92.5 (90) long); genu (57.5–57.5 (57.5) long); tibia (82.5–85 (85) long; 17.5–18.75 (18.75) wide); tarsus (12.5–13.75 (13.75) long). Palpomere proportions: femur/genu 1.57–1.61 (1.57); tibia/femur 0.89–0.94 (0.94); tibia length/width 4.53–4.71 (4.53).

**Venter** — (610–645 (645) long; 390–400 (400) wide) mostly colorless with faint purple genital plates. Gnathosomal bay (100–107.5 (407.5) long; 70–70 (70) wide). Cxgl-4 far from apex and ventral. **Medial suture** (75–85 (85) long). **Genital plates** (130–135 (135) long; 102.5–102.5 (102.5) wide). Additional measurements: Cx-1 (240–250 (250) long (total); 140–147.5 (147.5) long (medial)); Cx-3 (275–280 (275) wide); anterior venter (235–247.5 (247.5) long). Ventral proportions: gnathosomal bay length/width 1.43–1.54 (1.54); anterior venter/genital field length 1.81–1.83 (1.83); anterior venter length/genital field width 2.29–2.41 (2.41); anterior venter/medial suture 2.91–3.13 (2.91).

**Immatures** unknown.

######## Etymology.

Specific epithet (*dentirostra*) refers to the diagnostic tooth-like serrations on the dorsal surface of the rostrum (*dentis*, L. tooth; *rostrum*, L. snout).

######## Distribution.

**Figure 53.**
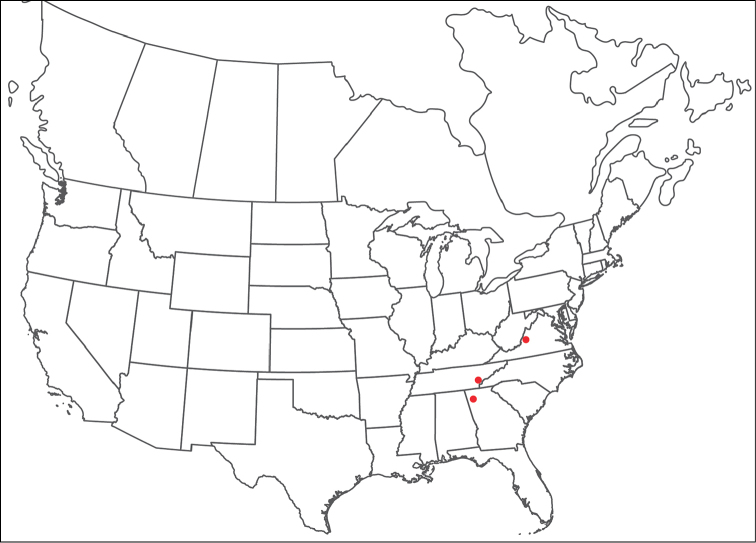
*Torrenticola dentirostra* sp. n. distribution.

Southern Appalachians (Figure 53).

**Figure 54.**
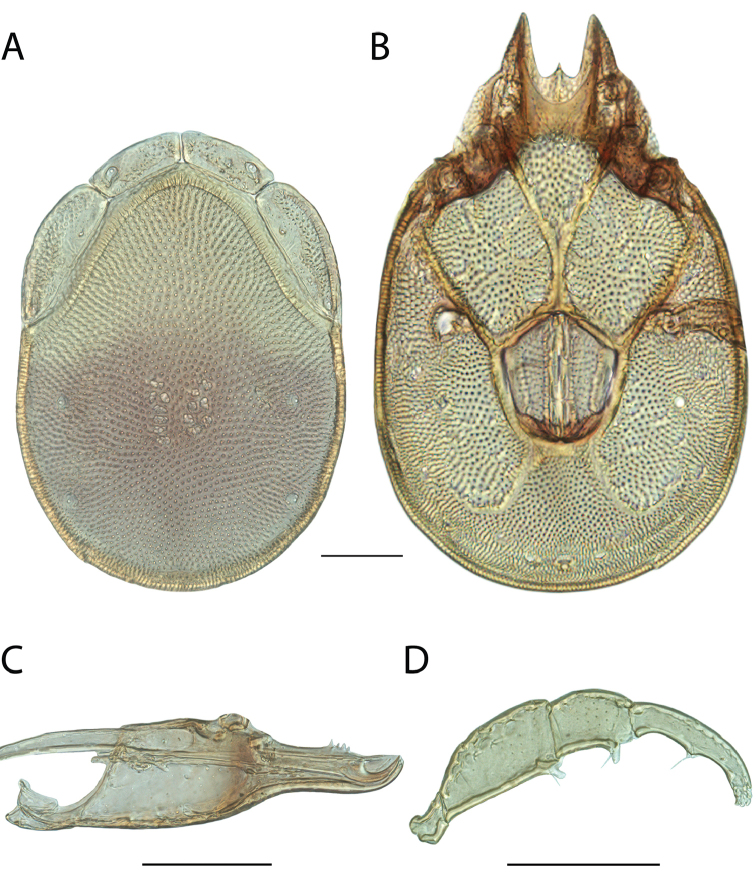
*Torrenticola dentirostra* sp. n. female: **A** dorsal plates **B** venter (legs removed) **C** subcapitulum **D** pedipalp (setae not accurately depicted). Scale = 100 µm.

**Figure 55.**
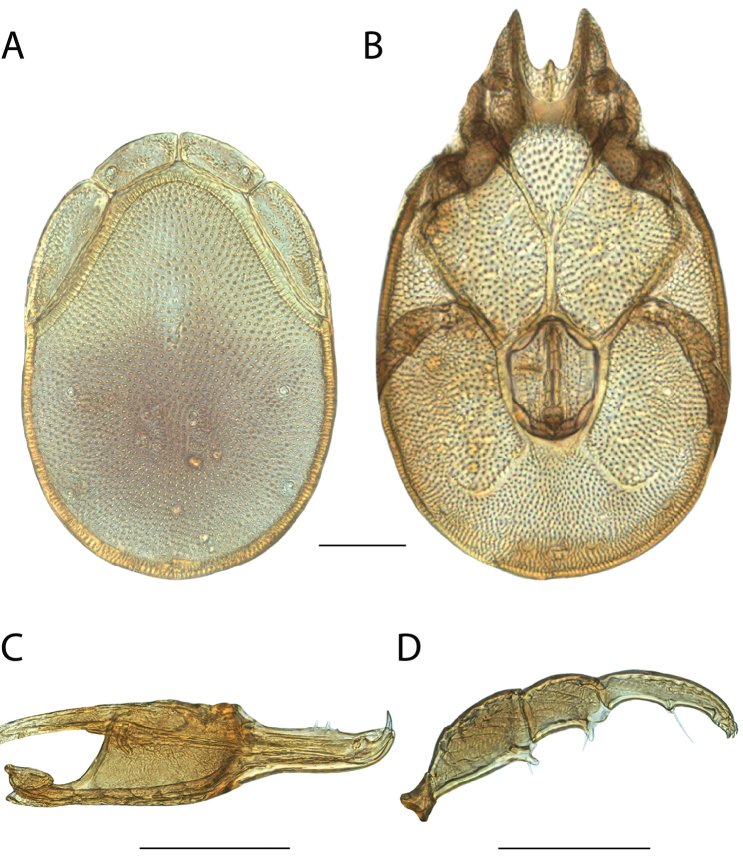
*Torrenticola dentirostra* sp. n. male: **A** dorsal plates **B** venter (legs removed) **C** subcapitulum **D** pedipalp (setae not accurately depicted). Scale = 100 µm.

######## Remarks.

Unfortunately, we were unable to acquire fresh material of *Torrenticola dentirostra* and therefore this species is not included in our phylogenetic analyses. However, we were able to examine morphology with material preserved in GAW. The overall similarity, small size, elongate subcapitular rostra, elongate pedipalpal tibiae, and dorsal purple coloration restricted to posterior half, place this species in the Raptor Complex and the Nigroalba Identification Group.

####### Torrenticola dimorpha

Taxon classificationAnimaliaTrombidiformesTorrenticolidae

Fisher & Dowling sp. n.

http://zoobank.org/C76F82BC-F0A2-40CC-8914-DBA542272577

######## Material examined.

HOLOTYPE (♀): from USA, Texas, Bandera County, Lost Maples State Natural Area, north of Vanderpool; picnic area, (29°48'48"N, 99°34'34"W), 10 October 2010, by IM Smith, IMS100184.

PARATYPES (8 ♀; 5 ♂): **Texas, USA**: 1 ♂ (ALLOTYPE) from Bandera County, Lost Maples State Natural Area, north of Vanderpool; picnic area, (29°48'48"N, 99°34'34"W), 10 October 2010, by IM Smith, IMS100184 • 1 ♀ and 1 ♂ from Bandera County, Lost Maples State Natural Area, north of Vanderpool; picnic area, (29°48'48"N, 99°34'34"W), 10 October 2010, by IM Smith, IMS100184 • 2 ♀ and 3 ♂ from Bandera County, Lost Maples State Natural Area north of Vanderpool; picnic area, (29°48'48"N, 99°34'34"W), 7 October 2010, by IM Smith, IMS100178 • 2 ♀ from Bandera County, Lost Maples State Natural Area; picnic area, (29°48'48"N, 99°34'34"W), 27 May 1997, by IM Smith, IMS970008 • 1 ♀ from Bandera County, Vanderpool; beside Rt. 187, 0.7 km south of entrance to Lost Maples State Natural Area, (29°48'48"N, 99°34'34"W), 2 May 2009, by IM Smith, IMS090007 • 1 ♀ from Kinney County, Brackettville; beside Rt. 90, 12.1 km west of Rt. 131, (29°20'20"N, 100°32'32"W), 4 May 2003, by IM Smith, IMS030007 • 1 ♀ from Uvalde County, Garner State Park; river crossing site, (29°35'35"N, 99°44'44"W), 28 May 1998, by IM Smith, IMS980027A.

######## Type deposition.

Holotype (♀), allotype (♂), and some paratypes (5 ♀; 2 ♂) deposited in the CNC; other paratypes (3 ♀; 2 ♂) deposited in the ACUA.

######## Diagnosis.

*Torrenticola dimorpha* are similar to other members of the Tricolor Complex (*T. bittikoferae*, *T. cardia*, *T. hoosieri*, *T. kringi*, *T. larvata*, *T. mohawk*, *T. pearsoni*, *T. olliei*, *T. sierrensis*, *T. tricolor*, *T. trimaculata*, and *T. unimaculata*) in having a short, conical rostrum. *T. dimorpha* can be differentiated from all other *Torrenticola* by having a dorsal plate with a medial extension covering nearly half the length of the anterio-medial platelets and by males having large, highly modified pedipalps, which are expanded vertically and laterally. Additionally, *T. dimorpha* can be differentiated from most other Tricolor Complex (except *T. bittikoferae*, *T. hoosieri*, and *T. pearsoni*) by being colorless, whereas most other members have bold patterning.

######## Description.

**Female (Figure 57)** (n = 5) (holotype measurements in parentheses when available) with characters of the genus with following specifications.

**Dorsum** — (590–670 (650) long; 455–520 (490) wide) colorless and ovoid. Anterior dorsal plate with medial extension covering nearly half the length of the anterio-medial platelets. Anterio-medial platelets (117.5–135 (135) long; 52.5–57.5 (57.5) wide). Anterio-lateral platelets (145–172.5 (172.5)) long; 55–65 (62.5) wide) free from dorsal plate. Dgl-4 approximately halfway in between the edge of the dorsum and the muscle scars (distance between Dgl-4 320–360 (360)). Dorsal plate proportions: dorsum length/width 1.29–1.33 (1.33); dorsal width/distance between Dgl-4 1.36–1.48 (1.36); anterio-medial platelet length/width 2.24–2.35 (2.35); anterio-lateral platelet length/width 2.46–2.78 (2.76); anterio-lateral/anterio-medial length 1.19–1.33 (1.28).

**Gnathosoma — Subcapitulum** (230–260 (255) long (ventral); 150–170 (170) long (dorsal); 110–132.5 (132.5) tall) colorless. Posterior dorsal apodeme long. Rostrum (65–70 (70) long; 42.5–50 (45) wide) very short. Chelicerae (210–245 (235) long) with curved fangs (60–70 (70) long). Subcapitular proportions: ventral length/height 1.90–2.09 (1.92); rostrum length/width 1.40–1.56 (1.56). **Pedipalps** with long, tuberculate ventral extensions on femora and no ventral extensions on genua. Palpomeres: trochanter (37.5–40 (37.5) long); femur (76.25–85 (82.5) long); genu (52.5–62.5 (60) long); tibia (65–72.5 (70) long; 22.5–22.5 (22.5) wide); tarsus (20–22.5 (22.5) long). Palpomere proportions: femur/genu 1.36–1.48 (1.38); tibia/femur 0.84–0.87 (0.85); tibia length/width 2.89–3.22 (3.11).

**Venter** — (720–800 (770) long; 505–600 (580) wide) colorless. Gnathosomal bay (120–145 (145) long; 72.5–85 (85) wide). Cxgl-4 apical. **Medial suture** (25–30 (30)). **Genital plates** (170–195 (185) long; 162.5–180 (180) wide). Additional measurements: Cx-1 (240–270 (270) long (total); 110–130 (125) long (medial)); Cx-3 (315–350 (350) wide); anterior venter (137.5–155 (155) long). Ventral proportions: gnathosomal bay length/width 1.60–1.79 (1.71); anterior venter/genital field length 0.78–0.84 (0.84); anterior venter length/genital field width 0.85–0.86 (0.86).

**Male (Figure 58)** (n = 5) (allotypic measurements in parentheses when available) with characters of the genus with following specifications.

**Dorsum** — (465–510 (500) long; 310–330 (320) wide) colorless and ovoid. Anterior dorsal plate with medial extension covering nearly half the length of the anterio-medial platelets. Muscle scars absent or very faint. Anterio-medial platelets (102.5–115 (107.5) long; 42.5–45 (45) wide). Anterio-lateral platelets (177.5–197.5 (195) long; 42.5–55 (55) wide) free from dorsal plate. Dgl-4 much closer to the edge of the dorsum than to the muscle scars (distance between Dgl-4 225–245 (245)). Dorsal plate proportions: dorsum length/width 1.50–1.58 (1.56); dorsal width/distance between Dgl-4 1.31–1.41 (1.31); anterio-medial platelet length/width 2.34–2.63 (2.39); anterio-lateral platelet length/width 3.38–4.65 (3.55); anterio-lateral/anterio-medial length 1.64–1.81 (1.81).

**Gnathosoma — Subcapitulum** (227.5–255 (240) long (ventral); 160–177.5 (165) long (dorsal); 100–110 (100) tall) colorless. Rostrum (45–50 (47.5) long; 32.5–37.5 (32.5) wide) very short. Chelicerae (195–235 (205) long) with curved fangs (55–65 (60) long). Subcapitular proportions: ventral length/height 2.17–2.40 (2.40); rostrum length/width 1.29–1.46 (1.46). **Pedipalps** highly modified and expanded, with long, tuberculate ventral extensions on femora and no ventral extensions on genua. Palpomeres: trochanter (53.75–57.5 (55) long); femur (107.5–112.5 (112.5) long); genu (92.5–103.75 (103.75) long); tibia (77.5–85 (85) long; 30–35 (35) wide); tarsus (20–25 (22.5) long). Palpomere proportions: femur/genu 1.08–1.22 (1.08); tibia/femur 0.69–0.77 (0.76); tibia length/width 2.36–2.67 (2.43).

**Venter** — (550–620 (580) long; 350–370 (370) wide) colorless and highly modified with coxae II–IV forming a large ventral plate that covers the insertions of legs IV. Suture dividing coxae III and IV incomplete. Apodemes expanded internally. Gnathosomal bay (110–115 (112.5) long; 90–110 (90) wide) expanded to accommodate large pedipalps. Cxgl-4 apical. **Medial suture** (155–165 (155) long). **Genital plates** (100–120 (105) long; 105–110 (105) wide) triangular. Additional measurements: Cx-1 (230–260 (250) long (total); 130–145 (130) long (medial)); Cx-3 (335–345 (335) wide); anterior venter (297.5–315 (300) long). Ventral proportions: gnathosomal bay length/width 1.05–1.25 (1.25); anterior venter/genital field length 2.63–2.98 (2.86); anterior venter length/genital field width 2.70–2.86 (2.86); anterior venter/medial suture 1.88–2.03 (1.94).

**Immatures** unknown.

######## Etymology.

Specific epithet (*dimorpha*) refers to the sexual dimorphism in terms of size and morphology (*di*, G. two; *morphḗ*, G. form), the resulting morphology is so unlike all other Torrenticolidae that upon first glance specimens appear to be a different family altogether.

######## Distribution.

**Figure 56.**
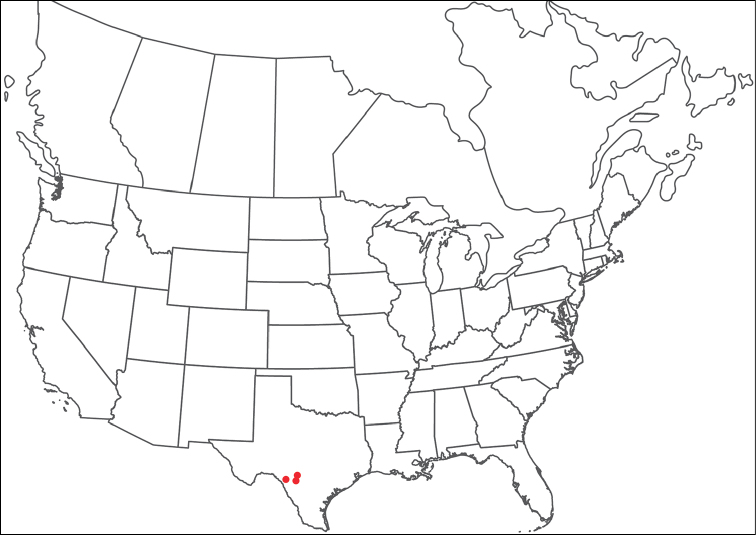
*Torrenticola dimorpha* sp. n. distribution.

Texas (probably also extending southward into Mexico) (Figure 56).

**Figure 57.**
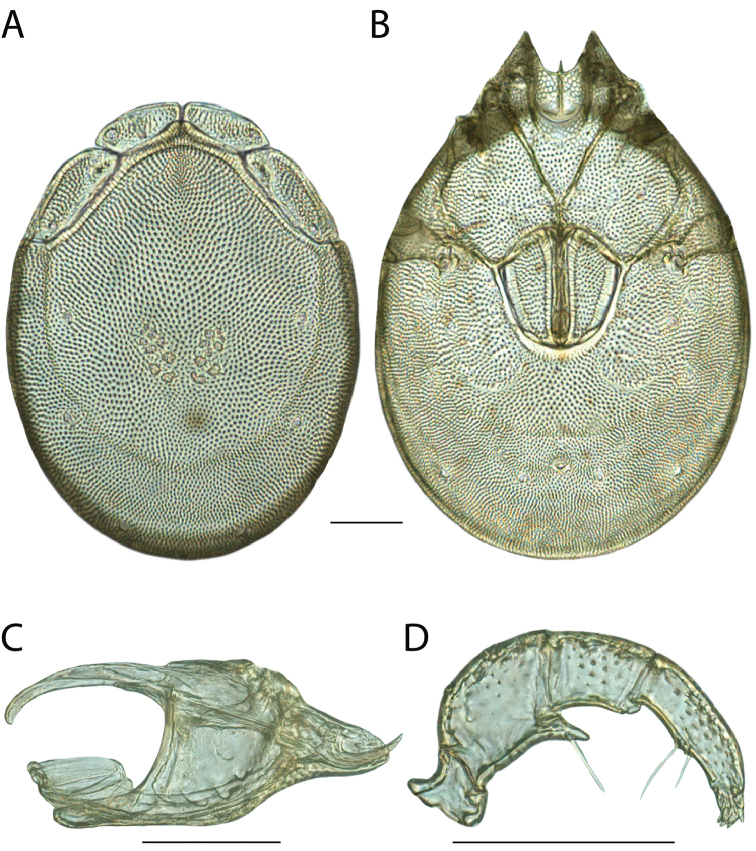
*Torrenticola dimorpha* sp. n. female: **A** dorsal plates **B** venter (legs removed) **C** subcapitulum **D** pedipalp (setae not accurately depicted). Scale = 100 µm.

**Figure 58.**
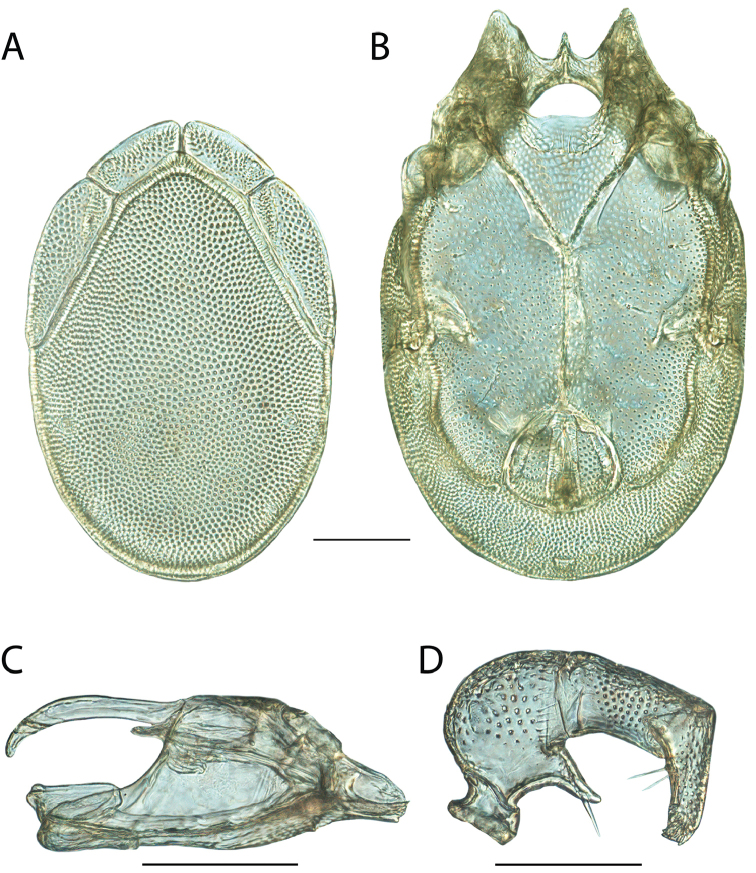
*Torrenticola dimorpha* sp. n. male: **A** dorsal plates **B** venter (legs removed) **C** subcapitulum **D** pedipalp (setae not accurately depicted). Scale = 100 µm.

######## Remarks.

*Torrenticola dimorpha* groups with other members of the Tricolor Complex with high support and specimens of this species were less than 1% different in COI sequence from each other. In the combined analysis, *T. dimorpha* groups with *T. larvata* and these species are greater than 16% different in COI sequence from each other. This clade (*T. dimorpha* + *T. larvata*) is sister to all other eastern members of the Tricolor Complex.

This species is so unique that upon initial observation, specimens appear to be members of a different genus, especially the males. However, the short conical rostrum that is downturned in males is characteristic of other members of the Tricolor Identification Group.

This species hypothesis is supported by biogeography, high divergence between species, and by highly distinctive morphological characteristics outlined in the diagnosis. The high degree of sexual size dimorphism (males are 15–30% smaller than females) is only matched by most members of the Rusetria Complex, where males are usually 20–30% smaller than females.

####### Torrenticola dolichodactyla

Taxon classificationAnimaliaTrombidiformesTorrenticolidae

Fisher & Dowling sp. n.

http://zoobank.org/38ADA75D-A5EA-42BC-8362-28666ADC8C69

######## Material examined.

HOLOTYPE (♂): from USA, New Mexico, Catron County, Cottonwood Campground beside Rt. 180 south of Rt. 12, (33°37'37"N, 108°54'54"W), 12 July 1987, by IM Smith, IMS870086.

PARATYPES (0 ♀; 0 ♂):

######## Type deposition.

Holotype (♂) deposited in the CNC.

######## Diagnosis.

*Torrenticola dolichodactyla* are similar to other members of the Rala Group (*T. rala*, *T. lamellipalpis*, *T. boettgeri*, *T. kurtvietsi*, *T. keesdavidsi*, and *T. anoplopalpa*) by being colorless, having incomplete hind coxal margins and being distributed in the southwest. *T. dolichodactyla* can be differentiated from all other Rala Group by having longer pedipalpal tarsi (♂ = 52.5 in *T. dolichodactyla*, 10–20 in others) and Dgl-4 closer to the muscle scars (dorsum width/distance between Dgl-4 ♂ = 1.74 in *T. dolichodactyla*, 1.16–1.48 in others).

######## Description.

**Female** unknown.

**Male (Figure 60)** (n = 1) (holotype only) with characters of the genus with following specifications.

**Dorsum** — (735 long; 590 wide) circular and colorless. Anterio-medial platelets (177.5 long; 77.5 wide). Anterio-lateral platelets (227.5 long; 97.5 wide) free from dorsal plate. Dgl-4 halfway between the muscle scars and the edge of the dorsum (distance between Dgl-4 340). Dorsal plate proportions: dorsum length/width 1.25; dorsal width/distance between Dgl-4 1.74; anterio-medial platelet length/width 2.29; anterio-lateral platelet length/width 2.33; anterio-lateral/anterio-medial length 1.28.

**Gnathosoma — Subcapitulum** (325 long (ventral); 235 long (dorsal); 135 tall) colorless. Rostrum (120 long; 50 wide). Chelicerae (325 long) with curved fangs (65 long). Subcapitular proportions: ventral length/height 2.41; rostrum length/width 2.40. **Pedipalps** with elongate tarsi and tuberculate ventral extensions on femora and genua. Palpomeres: trochanter (55 long); femur (140 long); genu (80 long); tibia (110 long; 32.5 wide); tarsus (52.5 long). Palpomere proportions: femur/genu 1.75; tibia/femur 0.79; tibia length/width 3.38.

**Venter** — (850 long; 720 wide) colorless. Gnathosomal bay (170 long; 115 wide). Cxgl-4 apical. **Medial suture** (95 long). **Genital plates** (220 long; 170 wide). Additional measurements: Cx-1 (350 long (total); 175 long (medial)); Cx-3 (480 wide); anterior venter (305 long). Ventral proportions: gnathosomal bay length/width 1.48; anterior venter/genital field length 1.39; anterior venter length/genital field width 1.79; anterior venter/medial suture 3.21.

**Immatures** unknown.

######## Etymology.

Specific epithet (*dolichodactyla*) refers to elongate pedipalp tarsus (*dolichos*, G. long; *daktylos*, G. finger), which is the most elongate of all Torrenticolidae.

######## Distribution.

**Figure 59.**
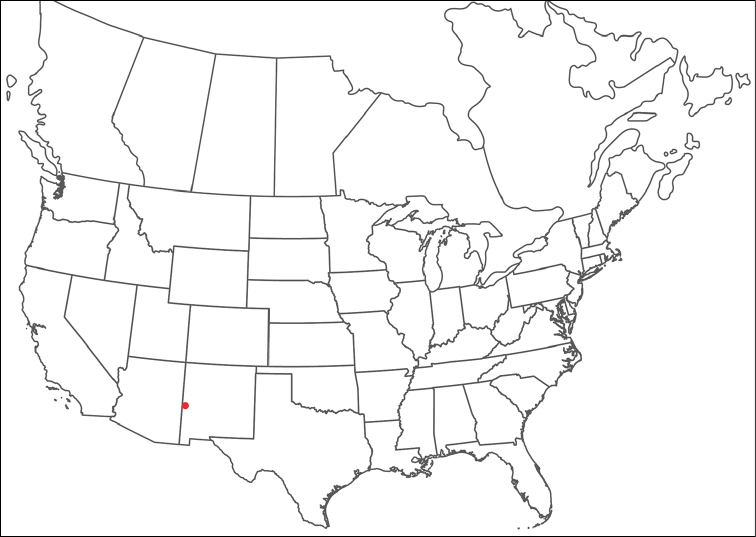
*Torrenticola dolichodactyla* sp. n. distribution.

New Mexico (probably also Arizona) (Figure 59).

**Figure 60.**
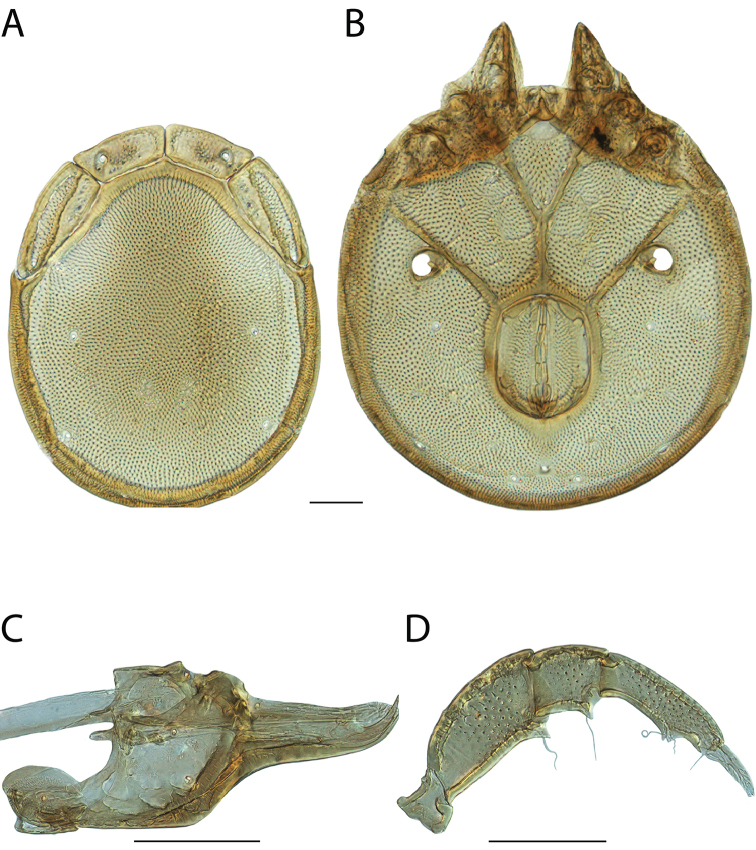
*Torrenticola dolichodactyla* sp. n. male: **A** dorsal plates **B** venter (legs removed) **C** subcapitulum **D** pedipalp (setae not accurately depicted). Scale = 100 µm.

######## Remarks.

Unfortunately, we were unable to acquire fresh material of *Torrenticola dolichodactyla* and therefore this species is not included in our phylogenetic analyses. However, we were able to examine morphology with material preserved in GAW. The overall appearance, incomplete hind coxal margins, distribution, and lack of color are consistent with placing this species in the Rala Identification Group.

####### Torrenticola dunni

Taxon classificationAnimaliaTrombidiformesTorrenticolidae

Fisher & Dowling sp. n.

http://zoobank.org/333688CE-B5DB-4930-B13D-FCFF639E54A7

######## Material examined.

**Type series.** HOLOTYPE (♀): from USA, Tennessee, Sevier County, Great Smokey Mountains National Park, Cosby Recreation Area (35°46'54"N, 83°13'2"W), 16 Sep 2010, by IM Smith, IMS100140, DNA 1289.

PARATYPES (9 ♀; 5 ♂): **North Carolina, USA**: 1 ♂ (ALLOTYPE) from Haywood County, Great Smokey Mountains National Park, Rough Fork Creek (35°37'31"N, 83°6'46"W), 20 Sep 2010, by IM Smith, IMS100148 • 2 ♀ and 2 ♂ from Haywood County, Great Smokey Mountains National Park, Rough Fork Creek (35°37'31"N, 83°6'46"W), 20 Sep 2010, by IM Smith, IMS100148 • 1 ♀ from Haywood County, Great Smokey Mountains National Park, Waterville (35°44'59"N, 83°6'42"W), 16 Sep 2010, by IM Smith, IMS100138 • **South Carolina, USA**: 2 ♀ from Greenville County, Matthews Creek, 24 Apr 2014, by D Eargle, JRF 14-0424-001 • **Tennessee, USA**: 1 ♀ from Blount County, Great Smokey Mountains National Park, Abrams River (35°35'31"N, 83°51'21"W), 17 Sep 2010, by IM Smith, IMS100141 • 1 ♀ and 1 ♂ from Sevier County, Great Smokey Mountains National Park (35°40'47"N, 83°31'48"W), 3 Sep 2009, by IM Smith, IMS090096 • 2 ♀ and 1 ♂ from Sevier County, Great Smokey Mountains National Park (35°43'33"N, 83°24'1"W), 12 Sep 2010, by IM Smith, IMS100131 • **Virginia, USA**: 1 ♀ from Smyth County, Mount Rogers National Recreation Area, beside Route 600, Little Laurel Creek, 10 Jul 1990, by IM Smith, IMS900086.

######## Type deposition.

Holotype (♀), allotype (♂), and most paratypes (4 ♀; 2 ♂) deposited in the CNC; other paratypes (5 ♀; 2 ♂) deposited in the ACUA.

######## Diagnosis.

*Torrenticola dunni* are similar to other members of the Rusetria “4-Plates” group (*T. dunni*, *T. glomerabilis*, *T. kittatinniana*, *T. pollani*, *T. rufoalba* and *T. shubini*) and *T. skvarlai* in having anterio-lateral platelets free from the dorsal plate, dorsal coloration separated into anterior and posterior portions, and indistinct hind coxal margins. *T. dunni* can be differentiated from *T. pollani* by having a larger dorsum (length ♀ = 605–680 in *T. dunni*, 535–560 in *T. pollani*; ♂ = 500–540 in *T. dunni*, 440–492 in *T. pollani*; width ♀ = 440–490 in *T. dunni*, 410–420 in *T. pollani*; ♂ = 350–370 in *T. dunni*, 310–340 in *T. pollani*) ; and a stockier rostrum (length/width = 2.80–3.14 in *T. dunni*, 3.27–3.82 in *T. pollani*). Female *T. dunni* can be differentiated from female *T. shubini* by having a thinner rostrum (length/width = 2.8–3.0 in *T. dunni*, 2.5–2.7 in *T. shubini*). Male *T. dunni* can be differentiated from male *T. shubini* by having a longer anterior venter (277–285 in *T. dunni*, 215–238 in *T. shubini*). *T. dunni* can be differentiated from *T. glomerabilis* by having Dgl-4 closer to the dorsal edge (dorsal width/distance between Dgl-4 = 1.2–1.4 in *T. dunni*, 1.5–1.7 in *T. glomerabilis*) and stockier tibiae (length/width ♀ = 3.27–3.50 in *T. dunni*, 4.11–4.50 in *T. glomerabilis*, ♂ = 3.25–3.44 in *T. dunni*, 3.55–4.38 in *T. glomerabilis*). Female *T. dunni* can be differentiated from female *T. kittatinniana* by having a longer pedipalp genu (70–75 in *T. dunni*, 64 in *T. kittatinniana*); a longer subcapitulum (ventral length = 330–355 in *T. dunni*, 310 in *T. kittatinniana*); and anterio-medial platelets more elongate (length/width = 2.33–2.54 in *T. dunni*, 2.83 in *T. kittatinniana*). Male *T. dunni* can be differentiated from male *T. kittatinniana* by having a longer anterior venter (277–285 in *T. dunni*, 235 in *T. kittatinniana*) and wider dorsum (350–370 in *T. dunni*, 340 in *T. kittatinniana*). *T. dunni* can be differentiated from *T. rufoalba* by having a larger dorsum (length ♀ = 605–680 in *T. dunni*, 550 in *T. rufoalba*; ♂ = 500–540 in *T. dunni*, 440 in *T. rufoalba*; width ♀ = 440–490 in *T. dunni*, 400 in *T. rufoalba*; ♂ = 350–370 in *T. dunni*; 320 in *T. rufoalba*). *T. dunni* can be differentiated from *T. skvarlai* by having a conical pedipalpal femoral tubercle, whereas *T. skvarlai* has a broad and flat pedipalpal femoral tubercle, and by having a longer anterior venter (♀ = 160–190 in *T. dunni*, 140–152.5 in *T. skvarlai*; ♂ = 277.5–285 in *T. dunni*, 177.5–205 in *T. skvarlai*).

######## Description.

**Female (Figure 62)** (n = 5) (holotype measurements in parentheses when available) with characters of the genus with following specifications.

**Dorsum**— (605–680 (655) long; 440–490 (460) wide) ovoid with purple or reddish-purple coloration separated into anterior and posterior portions, and occasionally with faint orange medially. Anterio-medial platelets (117.5–125 (125) long; 46.25–52.5 (50) wide). Anterio-lateral platelets (172.5–197.5 (192.5) long; 62.5–68.75 (62.5) wide) free from dorsal plate. Dgl-4 much closer to the edge of the dorsum than to the muscle scars (distance between Dgl-4 315–380 (340)). Dorsal plate proportions: dorsum length/width 1.37–1.42 (1.42); dorsal width/distance between Dgl-4 1.29–1.40 (1.35); anterio-medial platelet length/width 2.33–2.54 (2.50); anterio-lateral platelet length/width 2.76–3.08 (3.08); anterio-lateral/anterio-medial length 1.44–1.61 (1.54).

**Gnathosoma — Subcapitulum** (330–355 (345) long (ventral); 250–265 (255) long (dorsal); 132.5–150 (150) tall) colorless. Rostrum (130–140 (135) long; 45–50 (45) wide). Chelicerae (325–355 (350) long) with curved fangs (60–65 (60) long). Subcapitular proportions: ventral length/height 2.30–2.53 (2.30); rostrum length/width 2.80–3.00 (3.00). **Pedipalps** with tuberculate ventral extensions on femora and genua. Palpomeres: trochanter (42.5–50 (48.75) long); femur (117.5–132.5 (131.25) long); genu (70–75 (75) long); tibia (85–95 (90) long; 25–27.5 (27.5) wide); tarsus (20–20 (20) long). Palpomere proportions: femur/genu 1.68–1.83 (1.75); tibia/femur 0.69–0.72 (0.69); tibia length/width 3.27–3.50 (3.27).

**Venter** — (710–810 (780) long; 540–600 (600) wide) with faint bluish-purple or reddish purple coloration or colorless. Gnathosomal bay (157.5–175 (175) long; 92.5–115 (115) wide). Cxgl-4 subapical. **Medial suture** (20–25 (20) long). **Genital plates** (160–185 (177.5) long; 145–160 (152.5) wide). Additional measurements: Cx-1 (290–330 (330) long (total); 140–160 (155) long (medial)); Cx-3 (365–410 (400) wide); anterior venter (160–190 (190) long). Ventral proportions: gnathosomal bay length/width 1.50–1.70 (1.52); anterior venter/genital field length 0.99–1.07 (1.07); anterior venter length/genital field width 1.08–1.25 (1.25); anterior venter/medial suture 7.60–9.50 (9.50).

**Male (Figure 63)** (n = 5) (allotypic measurements in parentheses when available) with characters of the genus with following specifications.

**Dorsum** — (500–540 (540) long; 350–370 (360) wide) ovoid with purple or reddish-purple coloration separated into anterior and posterior portions, and occasionally with faint orange medially. Anterio-medial platelets (95–102.5 (100) long; 37.5–42.5 (41.25) wide). Anterio-lateral platelets (165–172.5 (172.5) long; 55–60 (55) wide) free from dorsal plate. Dgl-4 much closer to the edge of the dorsum than to the muscle scars (distance between Dgl-4 265–295 (285)). Dorsal plate proportions: dorsum length/width 1.35–1.53 (1.50); dorsal width/distance between Dgl-4 1.24–1.32 (1.26); anterio-medial platelet length/width 2.41–2.56 (2.42); anterio-lateral platelet length/width 2.88–3.14 (3.14); anterio-lateral/anterio-medial length 1.66–1.74 (1.73).

**Gnathosoma — Subcapitulum** (275–285 (285) long (ventral); 205–215 (215) long (dorsal); 102.5–115 (105) tall) colorless. Rostrum (105–112.5 (110) long; 35–38.75 (35) wide). Chelicerae (265–280 (275) long) with curved fangs (50–55 (52.5) long). Subcapitular proportions: ventral length/height 2.43–2.71 (2.71); rostrum length/width 2.90–3.14 (3.14). **Pedipalps** with tuberculate ventral extensions on femora and genua. Palpomeres: trochanter (40–47.5 (40) long); femur (105–107.5 (107.5) long); genu (62.5–66.25 (65) long); tibia 77.5–85 (77.5) long; 22.5–25 (22.5) wide); tarsus (17.5–20 (20) long). Palpomere proportions: femur/genu 1.58–1.72 (1.65); tibia/femur 0.72–0.79 (0.72); tibia length/width 3.25–3.44 (3.44).

**Venter** — (640–660 (655) long; 440–470 (460) wide) with faint bluish-purple or reddish purple coloration or colorless. Gnathosomal bay (125–135 (130) long; 75–82.5 (82.5) wide). Cxgl-4 subapical. **Medial suture** (120–135 (125) long). **Genital plates** (130–137.5 (135) long; 85–90 (87.5) wide). Additional measurements: Cx-1 (260–275 (275) long (total); 135–145 (145) long (medial)); Cx-3 (330–350 (335) wide); anterior venter (277.5–285 (285) long). Ventral proportions: gnathosomal bay length/width 1.56–1.80 (1.58); anterior venter/genital field length 2.04–2.19 (2.11); anterior venter length/genital field width 3.11–3.35 (3.26); anterior venter/medial suture 2.06–2.33 (2.28).

**Immatures** unknown.

######## Etymology.

Specific epithet (*dunni*) named in honor of Rob Dunn of North Carolina State University, for his exceptional writings and research that personalize ecology by bringing nature indoors; and particularly for his storytelling ability, in which he wonderfully conveys that we humans, rather than being separate from nature, are indeed just as wild as what we perceive outdoors—a sentiment exemplified by his book, *Wild Life of Our Bodies: Predators*, *Parasites*, *and Partners That Shape Who We Are Today* (2011).

######## Distribution.

**Figure 61.**
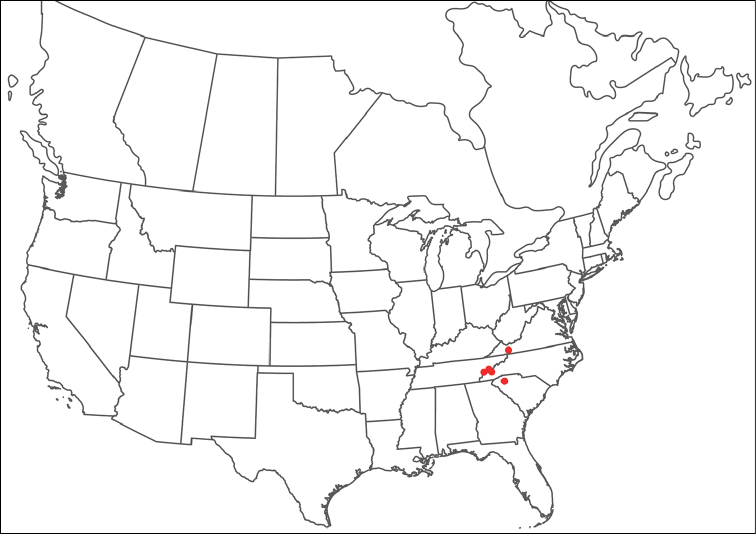
*Torrenticola dunni* sp. n. distribution.

Southeastern Appalachians (Figure 61).

**Figure 62.**
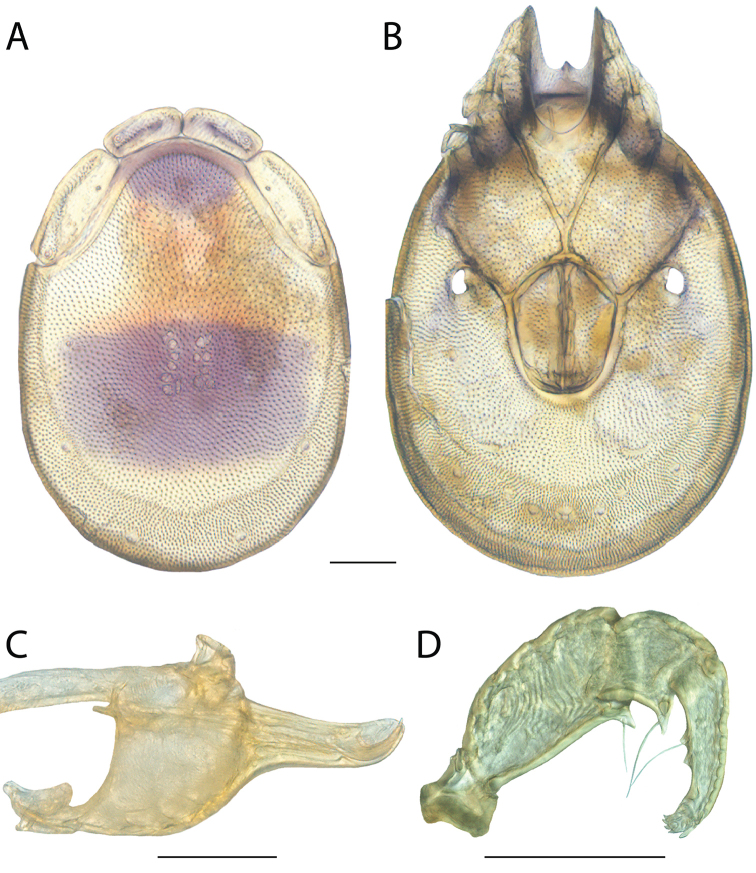
*Torrenticola dunni* sp. n. female: **A** dorsal plates **B** venter (legs removed) **C** subcapitulum **D** pedipalp (setae not accurately depicted). Scale = 100 µm.

**Figure 63.**
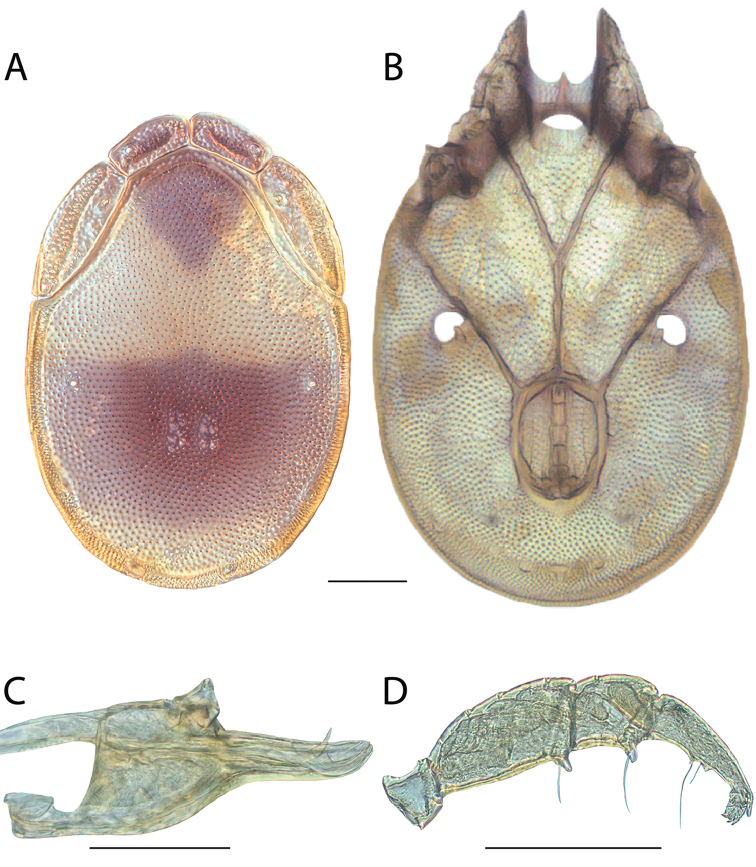
*Torrenticola dunni* sp. n. male: **A** dorsal plates **B** venter (legs removed) **C** subcapitulum **D** pedipalp (setae not accurately depicted). Scale = 100 µm.

######## Remarks.

In all analyses, *Torrenticola dunni* groups with two other members of the Rusetria Complex with high support and specimens are less than 1% different in COI sequence from each other. In all analyses, *T. dunni* groups with two other species: *T. pollani* and *T. shubini*. These species are greater than 5% different from each other in COI sequence and the ranges of *T. T. dunni* overlaps with *T. shubini*, but the ranges of these species do not overlap with *T. pollani*. Given our collection efforts across the Appalachians, it is reasonable to speculate that *T. dunni* is restricted to the southern Appalachians.

Based upon overall similarity, dorso-lateral platelet fusion, and distribution, we were able to place this species within the Eastern 2-Plate Identification Group

This species hypothesis is supported by biogeography, low COI divergence within the species (0–2%) and high divergence between species (3–15%), and by the morphological characters outlined in the diagnosis.

####### Torrenticola ellipsoidalis

Taxon classificationAnimaliaTrombidiformesTorrenticolidae

(Marshall, 1943)

Atractides ellipsoidalis : Marshall 1943: 308.
T. ellipsoidalis : Mitchell 1954: 40 • Habeeb 1955: 2 • Viets 1956: 241 • Crowell 1961: 330 • Habeeb 1967: 3 • Conroy 1968: 28 • Habeeb 1974: 1 • Conroy and Scudder 1975: 307 • Quaglia and Conroy 1984: 89 • Viets 1987: 759.
Torrenticola rectiforma Habeeb, 1974: 1.

######## Material examined.

**Type series.** HOLOTYPE (♀): from USA, California, Nevada County, north of Lake Tahoe, Martis Creek, Jun 1933, by PR Needham, RM330010.

PARATYPES (1 ♀; 0 ♂): California, USA: 1 ♀ from Nevada County, north of Lake Tahoe, Martis Creek, Jun 1933, by PR Needham, RM330010.

OTHER MATERIAL (94 ♀; 94 ♂): **Alberta, Canada**: 1 ♀ and 2 ♂ from Waterton Lakes National Park, Cameron Creek, beside Akamina Parkway (49°5'N, 113°52'W), 2 Aug 1985, by IM Smith, IMS850133 • 2 ♀ and 2 ♂ from Waterton Lakes National Park, Cameron Creek, beside Akamina Parkway, west of Rowe Creek, 27 Jun 1980, by IM Smith, IMS800086A • 1 ♀ and 2 ♂ from Waterton Lakes National Park, Cameron Creek, beside Akamina Parkway, 12-15 Jun 1980, by IM Smith, IMS800069A & IMS800069B • 2 ♀ and 2 ♂ from Waterton Lakes National Park, Little Prairie Picnic Area, Cameron Creek, 5-9 Jun 1980, by IM Smith, IMS800053B • **British Columbia, Canada**: 1 ♀ and 1 ♂ from Bella Coola Valley, Tweedsmuir Provincial Park, Atnarko River, at campground, 28 Jul 1983, by IM Smith & AB Smith, IMS830054A • 1 ♀ and 1 ♂ from Bella Coola Valley, Tweedsmuir Provincial Park, Atnarko Slough, beside Highway 20, west of Youngs Creek Picnic Area, 4 Aug 1983, by IM Smith, IMS830064 • 1 ♀ from Bella Coola Valley, Tweedsmuir Provincial Park, Belarko, Atnarko River, 24-26 Jul 1983, by IM Smith, IMS830049B • 1 ♀ and 2 ♂ from Bella Coola Valley, Tweedsmuir Provincial Park, Hotnarko River, at end of Atnarko tote road, 27 Jul 1983, by IM Smith, IMS830052 • 2 ♀ and 3 ♂ from Bella Coola Valley, Tweedsmuir Provincial Park, Hotnarko River, at end of Atnarko tote road, 31 Jul 1983, by IM Smith, IMS830059A & IMS830059B • 3 ♂ from Bella Coola Valley, Tweedsmuir Provincial Park, Youngs Creek Picnic Area, Atnarko Slough, 24-27 Jul 1983, by IM Smith, IMS830048A • 2 ♀ and 1 ♂ from Fernie, Lizard Creek, beside Highway 3, 1.8 km west of Fernie Mountain Provincial Park, 16 Aug 2012, by IM Smith, IMS120073 • 1 ♀ and 1 ♂ from Tweedsmuir Provincial Park, Youngs Creek, beside Highway 20, between Heckman Pass & Bella Coola Valley, 5 Aug 1983, by IM Smith, IMS830065 • 2 ♀ and 2 ♂ from Vancouver Island, Caycuse, Nixon Creek, 8 Jul 1976, by IM Smith, IMS760197 & IMS760198 • 1 ♀ and 2 ♂ from Vancouver Island, beside Highway 4, 35.6 kilometers east of Pacific Rim Road, 9 Jul 1976, by IM Smith, IMS760206 • 1 ♀ and 1 ♂ from Vancouver Island, Lake Cowichan, Cowichan River, above Skutz Falls, 9 Jul 1979, by IM Smith, IMS790035 • 1 ♀ and 1 ♂ from Vancouver Island, Lake Cowichan, Skutz Falls, Skutz Creek, near Cowichan River, 9 Jul 1979, by IM Smith, IMS790036A & IMS790036B • 1 ♀ and 2 ♂ from Vancouver Island, Lake Cowichan, South Shore Road, north of Mesachie Lake, Robertson River, 4 Jul 1976, by IM Smith, IMS760183A • 1 ♀ from Vancouver Island, Lake Cowichan, South Shore Road, north of Mesachie Lake, tributary of Robertson River, 4-10 Jul 1976, by IM Smith, IMS760182 • 1 ♀ and 2 ♂ from Vancouver Island, Lost Shoe Creek, beside Highway 4, 1.3 kilometers east of Pacific Rim Road, 9 Jul 1976, by IM Smith, IMS760202 • 1 ♀ and 2 ♂ from Vancouver Island, Malahat, Goldstream Provincial Park, Goldstream River, 26 Jun 1979, by IM Smith, IMS790028A • 1 ♂ from Vancouver Island, spring run beside North Shore Road, 1.7 kilometers north of Lake Cowichan, 7 Jun 1979, by IM Smith, IMS790008A • 3 ♀ and 2 ♂ from Vancouver Island, North Shore Road, 3.2 kilometers south of Youbou, 4 Jul 1976, by IM Smith, IMS760190 • 1 ♀ from Vancouver Island, Youbou, Shaw Creek, North Shore Road, 4.3 kilometers south of north end of Cowichan Lake, 8 Jul 1976, by IM Smith, IMS760196 • 1 ♀ from Vancouver Island, spring run beside South Shore Road, 2.3 kilometers north of Lake Cowichan, 6 Jun 1979, by IM Smith, IMS790007 • 1 ♀ and 1 ♂ from Vancouver Island, Ucluelet, beside Highway 4, 16.6 kilometers east of Pacific Rim Road, 18-19 Jul 1979, by IM Smith, IMS790047 • **California, USA**: 1 ♀ and 1 ♂ from Humboldt County, Prairie Creek State Park, Prairie Creek, 12 Jul 1964, by H Habeeb, HH640021 • 1 ♀ from Humboldt County, Trinidad, stream beside Patrick Point Road, near Bishop Pine Lodge, 7 Aug 1987, by IM Smith, IMS870134 • 1 ♀ from Inyo County, Inyo National Forest, Bishop Creek (37°17'23"N, 118°33'14"W), 2 Sep 2013, by JR Fisher, JRF 13-0902-003 • 1 ♂ from Mendocino County, Cottaneva Creek, beside Route 1, 21.8 kilometers southwest of Route 101, 5 Aug 1987, by IM Smith, IMS870129A • 2 ♀ and 3 ♂ from Mendocino County, small stream at beach access road, off Route 1, 2.6 kilometers south of Westport, 5 Aug 1987, by IM Smith, IMS870128A • 1 ♀ from Mono County, Humboldt-Toiyabe National Forest, Leavitt Creek (38°18'40"N, 119°34'49"W), 31 Aug 2013, by JR Fisher, JRF 13-0831-004 • 1 ♂ from Monterey County, Los Padres National Forest, Salmon Creek (35°48'57"N, 121°21'29"W), 6 Sep 2013, by JR Fisher, JRF 13-0906-003 • 2 ♀ and 2 ♂ from Monterey County, Nacimiento River, beside Nacimiento-Ferguson Road at Nacimiento campground, 30 Jul 1987, by IM Smith, IMS870120A • 2 ♀ and 2 ♂ from Monterey County, Salmon Creek, beside Route 1, south of Gorda, 28 Jul 1987, by IM Smith, IMS870114A • 1 ♀ from Nevada County, beside Route 89, north of Hobart Mills, 13 Jun 1976, by IM Smith, IMS760109 • 1 ♀ and 2 ♂ from San Bernardino County, Claremont, Mt Baldy, 3.5 kilometers above Mt Baldy Village, 24 Jul 1987, by IM Smith, IMS870107 • 1 ♀ from San Bernardino County, Claremont, Mt Baldy, San Antonio Falls, above Monker Flats, 24 Jul 1987, by IM Smith, IMS870105 • 3 ♀ and 2 ♂ from San Bernardino County, Claremont, Mt Baldy, stream below San Antonio Falls, above Monker Flats, 24 Jul 1987, by IM Smith, IMS870106 • 2 ♀ and 2 ♂ from Shasta County, Battle Creek, beside Route 44, 5.6 kilometers west of Viola, 10 Aug 1987, by IM Smith, IMS870139A • 1 ♀ and 1 ♂ from Trinity County, small cascading trickle beside Route 36, 5.2 kilometers west of Forest Glen Station, 6 Aug 1987, by IM Smith, IMS870132 • 3 ♂ from Tulare County, Stony Creek at Stony Creek Picnic Area, east of Sequoia National Park, 1 Aug 1987, by IM Smith, IMS870124A • 1 ♀ and 1 ♂ from Ventura County, Ojai, North Fork of Ventura River, beside Route 33, just above Wheeler Gorge, 25-26 Jul 1987, by IM Smith, IMS870109A • **Idaho, USA**: 1 ♀ from Custer County, Challis National Forest, Stanley Creek (44°15'12"N, 115°0'19"W), 30 Jul 2012, by JR Fisher, WA Nelson, & JC O’Neill, ROW 12-0730-005 • 1 ♂ from Custer County, Salmon River (44°12'31"N, 114°55'51"W), 29 Jul 2012, by JR Fisher, WA Nelson, & JC O’Neill, ROW 12-0729-003 • 2 ♀ and 3 ♂ from Lemhi County, North Fork of Salmon River, beside Route 93, 15 kilometers north of North Fork, 1 Jul 1985, by IM Smith, IMS850062 • **Montana, USA**: 2 ♀ from Flathead County, stream beside Route 487 near north end of Whitefish Lake, 29 Jun 1985, by IM Smith, IMS850057 • 3 ♀ and 4 ♂ from Lake County, stream beside Route 83, 39.5 kilometers north of Condon, 30 Jun 1985, by IM Smith, IMS850059A • 1 ♀ from Missoula County, Lolo National Forest, Lolo Creek (46°46'7"N, 114°27'53"W), 7 Aug 2012, by JR Fisher, WA Nelson, & JC O’Neill, ROW 12-0807-003 • 1 ♂ from Ravalli County, Bitterroot National Forest, East Fork Bitterroot River (45°51'40"N, 114°1'46"W), 3 Aug 2012, by JR Fisher, WA Nelson, & JC O’Neill, ROW 12-0803-005 • **Oregon, USA**: 1 ♀ and 1 ♂ from Benton County, Marys Peak near Philomath, Parker Creek, 27-28 Jun 1983, by IM Smith & AB Smith, IMS830006 • 1 ♀ from Clackamas County, Rhododendron Pioneer Tollgate campground, Zigzag River, 27 Jun 1976, by IM Smith, IMS760164 • 1 ♀ and 2 ♂ from Curry County, Port Orford, Butler Bar campground, Elk River, 25 Jun 1976, by IM Smith, IMS760162 • 1 ♂ from Coos County, Siskiyou National Forest, Road 33 between Powers & Agness, Daphne Grove campground, Coquille River, 2 Jul 1983, by IM Smith, IMS830016 • 1 ♀ and 1 ♂ from Coos County, Siskiyou National Forest, Road 33 between Powers & Agness, Daphne Grove campground, 2 Jul 1983, by IM Smith, IMS830017 • 2 ♀ and 5 ♂ from Curry County, Port Orford, Humbug Mountain State Park Picnic Area, beside Route 1, Brush Creek, 1 Jul 1983, by IM Smith, IMS830012 • 4 ♀ and 4 ♂ from Curry County, Port Orford, Humbug Mountain State Park Picnic Area, Brush Creek, beside Route 1, 3 Jul 1983, by IM Smith, IMS830020A & IMS830020B • 1 ♀ from Curry County, Port Orford, Humbug Mountain State Park Picnic Area, beside Route 1, 1 Jul 1983, by IM Smith, IMS830013 • 1 ♀ from Curry County, Rogue River National Forest, Elk River (42°42'46"N, 124°18'41"W), 13 Aug 2013, by JR Fisher, JRF 13-0813-003 • 2 ♀ and 2 ♂ from Curry County, Siskiyou National Forest, road 33 between Powers and Agness, North Fork of Foster Creek, 2 Jul 1983, by IM Smith, IMS830019 • 1 ♀ from Curry County, Sixes, Sixes River, beside road at mouth of Edson Creek, 4 Jul 1983, by IM Smith, IMS830021A • 1 ♀ from Grant County, Prairie City, Lunch Creek, beside Route 26. east of Dixie Pass, 17-20 Jun 1976, by IM Smith, IMS760125 • 1 ♀ and 1 ♂ from Grant County, Prairie City, Strawberry Forest Camp, Strawberry Creek, 17-20 Jun 1976, by IM Smith, IMS760126 • 2 ♀ from Lane County, Gate Creek (44°8'48"N, 122°34'20"W), 11 Aug 2013, by JC O’Neill, & WA Nelson, JNOW 13-0811-001 • 3 ♀ and 1 ♂ from Marion County, Marion Forks Riverside campground, North Fork of Santiam River, 22 Jun 1976, by IM Smith, IMS760145 • 3 ♀ and 4 ♂ from Multnomah County, Columbia River Scenic Highway, Horsetail Falls, 27 Jun 1983, by IM Smith, IMS830005 • 1 ♂ from Tillamook County, Siuslaw National Forest, Alder Creek (45°9'27"N, 123°47'60"W), 6 Aug 2013, by JC O’Neill, & WA Nelson, JNOW 13-0806-002 • **Washington, USA**: 2 ♀ from Lewis County, Gifford Pinchot National Forest (46°39'49"N, 121°41'11"W), 23 Jul 2013, by JC O’Neill, & WA Nelson, JNOW 13-0723-005 • 2 ♀ from Mason County, Olympic National Forest, Cabin Creek (47°35'44"N, 123°7'39"W), 22 Jul 2013, by JC O’Neill, & WA Nelson, JNOW 13-0722-004 • 2 ♀ from Snohomish County, Mount Baker National Forest, Marten River (48°4'19"N, 121°36'24"W), 28 Jul 2013, by JC O’Neill, & WA Nelson, JNOW 13-0728-002 • **Wyoming, USA**: 1 ♂ from Johnson County, Bighorn Mountains, Clear Creek, west of Buffalo Mosier Gulch Picnic Area, 28 Jul 2012, by IM Smith, IMS120041 • 1 ♀ and 1 ♂ from Johnson County, Crazy Woman Creek, beside Route 16, 14.9 kilometers west of road to South Fork campground, 18 Aug 1987, by IM Smith, IMS870158A • 1 ♀ and 2 ♂ from Washakie County, Ten Sleep, Ten Sleep Creek, beside Route 16, 4 kilometers west of Route 435, 18 Aug 1987, by IM Smith, IMS870157 • 2 ♀ and 1 ♂ from Washakie County, Ten Sleep Creek, Ten Sleep Wigwam Rearing Station, 26 Jul 2012, by IM Smith, IMS120044.

######## Type deposition.

Holotype (♀) and paratype (1 ♀) deposited in the CNC.

######## Diagnosis.

*Torrenticola ellipsoidalis* are similar to other members of the Ellipsoidalis Group (*T. multiforma*, *T. occidentalis*, and *T. leviathan*), in being among the largest *Torrenticola* in the west (dorsum length ♀ = 700–885; ♂ = 665–850), although *T. sierrensis* are also large (dorsum length ♀ = 700–880; ♂ = 590–735) but can easily be distinguished from the Ellipsoidalis Group by being circular instead of ellipsoid or rectangular (dorsum length/width = 1.17–1.28 in *T. sierrensis*, 1.30–1.67 in Ellipsoidalis Group). *T. ellipsoidalis* can be differentiated from *T. multiforma* by having stockier subcapitular rostra (length/width = 1.8–2.1 in *T. ellipsoidalis*, 2.5–2.8 in *T. multiforma*). *T. ellipsoidalis* can be differentiated from *T. occidentalis* (only known from females) by having a longer medial suture (40–57.5 in *T. ellipsoidalis*, 20 in *T. occidentalis*) and by having stockier anterio-lateral platelets (length/width = 2.00–2.39 in *T. ellipsoidalis*, 2.54 in *T. occidentalis*). *T. ellipsoidalis* can be differentiated from *T. leviathan* by having less elongate pedipalpal tibiae (length/width = 2.6–3.3 in *T. ellipsoidalis*, 3.4–4.2 in *T. leviathan*) and stockier anterio-medial platelets (length/width ♀ = 1.43–1.72 in *T. ellipsoidalis*, 1.94–2.14 in *T. leviathan*; ♂ = 1.53–2.00 in *T. ellipsoidalis*, 2.15 in *T. leviathan*).

######## Re-description.

**Female (Figure 65)** (n = 8) (holotype measurements in parentheses when available) with characters of the genus with following specifications.

**Dorsum** — (765–885 (800) long; 520–605 (540) wide) rectangular and usually colorless, occasionally with faint purple coloration without distinct pattern. Anterio-medial platelets (127.5–147.5 137.5) long; 80–97.5 (80) wide). Anterio-lateral platelets (207.5–235 (217.5) long; 90–115 (92.5) wide) free from dorsal plate. Dgl-4 much closer to the edge of the dorsum than to the muscle scars (distance between Dgl-4 390–470 (410)). Dorsal plate proportions: dorsum length/width 1.41–1.64 (1.48); dorsal width/distance between Dgl-4 1.27–1.40 (1.32); anterio-medial platelet length/width 1.43–1.72 (1.72); anterio-lateral platelet length/width 2.00–2.39 (2.35); anterio-lateral/anterio-medial length 1.48–1.77 (1.58).

**Gnathosoma — Subcapitulum** (285–315 (310) long (ventral); 194–219 (215) long (dorsal); 145–165 (165) tall) colorless. Rostrum (115–127.5 (117.5) long; 57.5–62.5 (60) wide) short and conical. Chelicerae (261–289 long) with curved fangs (61–74 long). Subcapitular proportions: ventral length/height 1.82–2.07 (1.88); rostrum length/width 1.84–2.09 (1.96). **Pedipalps** with tuberculate ventral extensions on femora and genua. Palpomeres: trochanter (37.5–42.5 (40) long); femur (101.25–107.5 (102.5) long); genu (70–77.5 (77.5) long); tibia (80–90 (85) long; 26.25–27.5 (27.5) wide); tarsus (17.5–21.25 (20) long). Palpomere proportions: femur/genu 1.32–1.46 (1.32); tibia/femur 0.76–0.85 (0.83); tibia length/width 2.91–3.27 (3.09).

**Venter** — (885–1000 (935) long; 605–700 (605) wide) colorless. Gnathosomal bay (157.5–180 (177.5) long; 80–105 (85) wide). Cxgl-4 subapical. **Medial suture** (40–57.5 (47.5) long). **Genital plates** (201.25–222.5 (205) long; 167.5–195 (172.5) wide). Additional measurements: Cx-1 (308–337.5 (335) long (total); 122–162.5 (162.5) long (medial)); Cx-3 (393–440 (405) wide); anterior venter (210–237.5 (225) long). Ventral proportions: gnathosomal bay length/width 1.50–2.12 (2.09); anterior venter/genital field length 1.01–1.13 (1.10); anterior venter length/genital field width 1.13–1.30 (1.30); anterior venter/medial suture 3.83–5.94 (4.74).

**Male (Figure 66)** (n = 6) with characters of the genus with following specifications.

**Dorsum** — (725–850 long; 450–565 wide) rectangular and usually colorless, occasionally with faint purple coloration without distinct pattern. Anterio-medial platelets (122.5–165 long; 72.5–95 wide). Anterio-lateral platelets (195–230 long; 85–107.5 wide) free from dorsal plate. Dgl-4 much closer to the edge of the dorsum than to the muscle scars (distance between Dgl-4 350–460). Dorsal plate proportions: dorsum length/width 1.38–1.67; dorsal width/distance between Dgl-4 1.22–1.43; anterio-medial platelet length/width 1.53–2.00; anterio-lateral platelet length/width 2.07–2.36; anterio-lateral/anterio-medial length 1.39–1.67.

**Gnathosoma — Subcapitulum** (280–290 long (ventral); 196–203.75 long (dorsal); 138.75–155 tall) colorless. Rostrum (102.5–115 long; 52.5–60 wide) short and conical. Chelicerae (263–280 long) with curved fangs (60–74 long). Subcapitular proportions: ventral length/height 1.87–2.04; rostrum length/width 1.86–2.02. **Pedipalps** with tuberculate ventral extensions on femora and genua. Palpomeres: trochanter (35–41.25 long); femur 92.5–100 long); genu (65–72.5 long); tibia (72.5–80 long; 25–30 wide); tarsus (17.5–20 long). Palpomere proportions: femur/genu 1.31–1.46; tibia/femur 0.74–0.84; tibia length/width 2.64–3.1.

**Venter** — (840–980 long; 469–653 wide) colorless. Gnathosomal bay (147.5–177.5 long; 77.5–90 wide). Cxgl-4 subapical. **Medial suture** (70–90 long). **Genital plates** (177.5–236.25 long; 131.25–162.5 wide). Additional measurements: Cx-1 (283–345 long (total); 117–167.5 long (medial)); Cx-3 (348–432.5 wide); anterior venter (245–270 long). Ventral proportions: gnathosomal bay length/width 1.69–2.15; anterior venter/genital field length 1.14–1.44; anterior venter length/genital field width 1.66–1.96; anterior venter/medial suture 2.72–3.71.

**Immatures** unknown.

######## Etymology.

Marshall (1943) presumably named the specific epithet (*ellipsoidalis*) after the elongate body of this species, as she wrote, “the body is an ellipse.”

######## Distribution.

**Figure 64.**
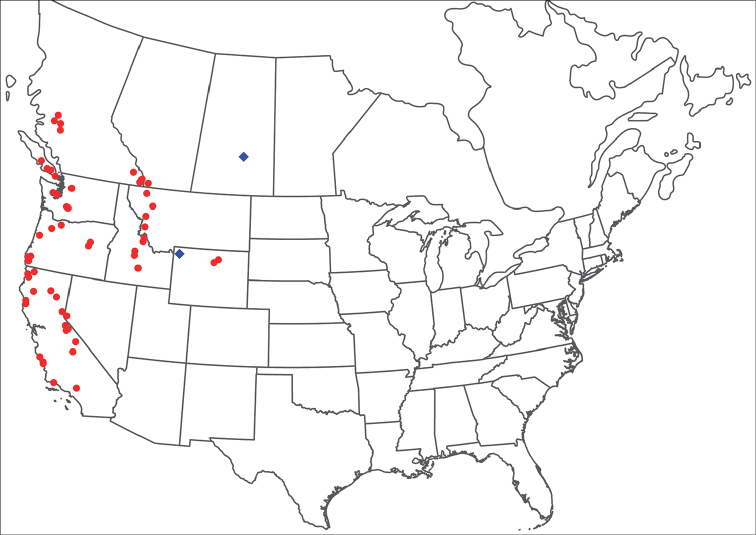
*Torrenticola ellipsoidalis* distribution. Red dots indicate material examined. Blue crosses indicate additional previous records (Marshall 1943; Quaglia & Conroy 1984).

Western (Figure 64). *T. ellipsoidalis* was previously recorded from Martis Creek and Gibbon River, Wyoming (Marshall 1943); and from Torch River, Saskatchewan (Quaglia & Conroy 1984). We expand the range into most of western North America. However, *T. ellipsoidalis* is not known from the southwest.

**Figure 65.**
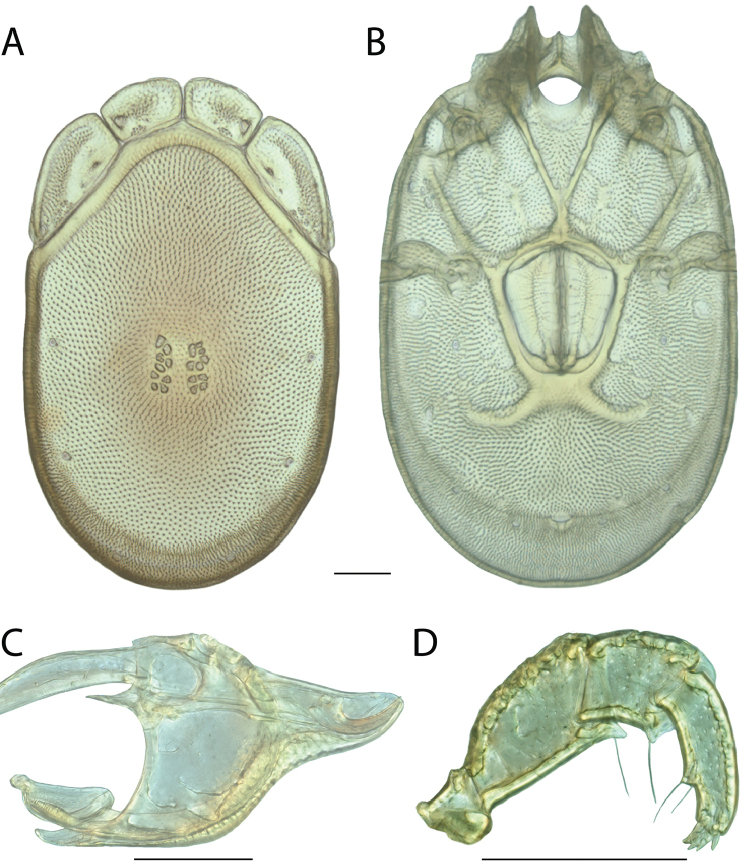
*Torrenticola ellipsoidalis* female: **A** dorsal plates **B** venter (legs removed) **C** subcapitulum **D** pedipalp (setae not accurately depicted). Scale = 100 µm.

**Figure 66.**
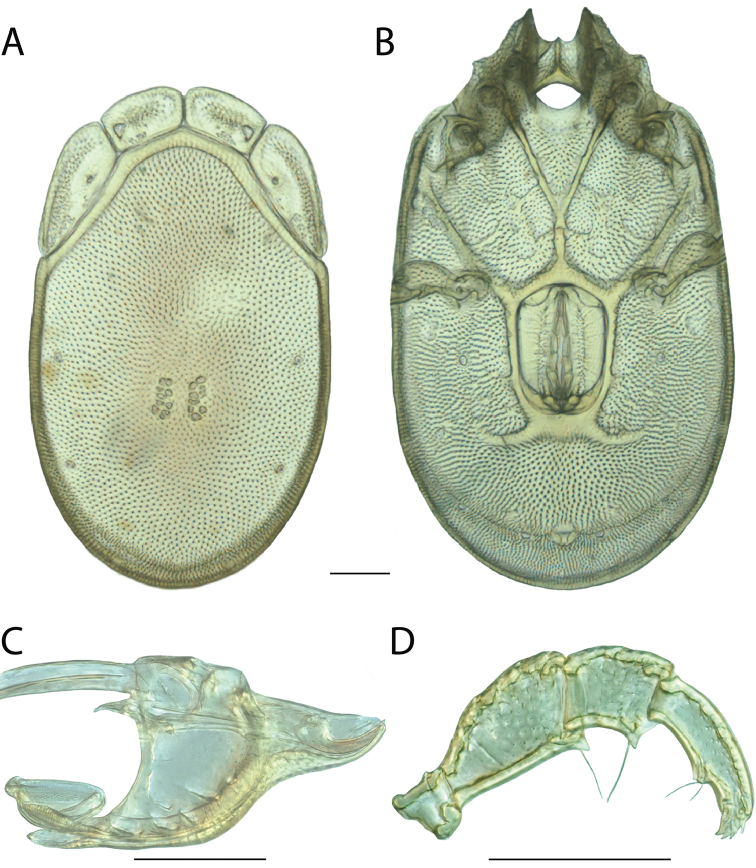
*Torrenticola ellipsoidalis* male: **A** dorsal plates **B** venter (legs removed) **C** subcapitulum **D** pedipalp (setae not accurately depicted). Scale = 100 µm.

######## Remarks.

*Torrenticola ellipsoidalis* groups other members of the Miniforma Complex with high support and most specimens are 0–3% different in COI sequence. This is higher sequence variability than in many species hypotheses presented herein. However, given the topology in the COI tree (Figure 10, 16) and morphological similarity, it seems apparent that the variability represents a continuum across a large distribution, rather than isolated species. An exception is that a single specimen (DNA#1930) is 2.9–3.6% different from the rest. This specimen is indistinguishable from other specimens and is collected from the same location. We do not find evidence to propose it as a separate species and therefore include it within *T. ellipsoidalis*.

In all analyses, *T. ellipsoidalis* groups with *T. multiforma* and *T. regalis*, which are greater than 10% different from each other. Based upon overall similarity, body size, and distribution, we place this species within the Ellipsoidalis Identification Group.

Upon examining the types of *T. ellipsoidalis* and *T. rectiforma*, all characters for both species overlap with members of only one clade in our analyses. Furthermore, the main character Habeeb (1974) used to differentiate *T. rectiforma* from *T. ellipsoidalis* was body size, which is known to be a highly variable character. Therefore, we synonymize *T. rectiforma* as the junior synonym of *T. ellipsoidalis*.

####### Torrenticola elongata

Taxon classificationAnimaliaTrombidiformesTorrenticolidae

Fisher & Dowling sp. n.

http://zoobank.org/85ABDA54-25DD-44C9-B075-C344261B7347

######## Material examined.

HOLOTYPE (♀): from USA, Mississippi, Tishomingo County, Tishomingo State Park, Rock Quarry Branch (34°36'N, 88°11'W), 18 Sep 1991, by IM Smith, IMS910049.

PARATYPES (1 ♀; 2 ♂): **Mississippi, USA**: 1 ♂ (ALLOTYPE) from Tishomingo County, Tishomingo State Park, Rock Quarry Branch (34°36'43"N, 88°12'4"W), 20 Sep 2009, by IM Smith, IMS090115, DNA 1593 • 1 ♀ and 1 ♂ from Tishomingo County, Tishomingo State Park, Rock Quarry Branch (34°36'43"N, 88°12'4"W), 20 Sep 2009, by IM Smith, IMS090115.

######## Type deposition.

Holotype (♀), allotype (♂) deposited in the CNC; other paratypes (1 ♀; 1 ♂) deposited in the ACUA.

######## Diagnosis.

*Torrenticola elongata* are similar to species with similar dorsal patterning, such as the Rusetria “4-Plate” group (*T. dunni*, *T. glomerabilis*, *T. kittatinniana*, *T. pollani*, *T. rufoalba* and *T. shubini*), Neoanomala Group (*T. interiorensis* and *T. neoanomala*), and *T. bondi*, *T. erectirostra*, *T. robisoni*, *T. gorti*, *T. reduncarostra*, *T. irapalpa*, *T. racupalpa*, *T. skvarlai*, and *T. arktonyx*. *T. elongata* can be differentiated from all other *Torrenticola* with similar dorsal patterning by having a more elongate dorsum (length/width ♀ = 1.92–2.08 in *T. elongata*, 1.17–1.58 in others; ♂ = 1.70–1.70 in *T. elongata*, 1.20–1.68 in others). Additionally, they can be differentiated from Rusetria 4-Plates and *T. skvarlai* by having distinct hind coxal margins, they can be differentiated from *T. erectirostra* and *T. robisoni* by having a straight, anteriorly-directed rostrum (upturned in others), and they can be differentiated from *T. arktonyx* by having an unmodified dorsal plate (*T. arktonyx* has distinctive longitudinal dark markings on the anterior portion of the dorsal plate that fade posteriorly).

######## Description.

**Female (Figure 68)** (n = 2) (holotype measurements in parentheses when available) with characters of the genus with following specifications.

**Dorsum** — (540–565 (540) long; 260–295 (260) wide) ovoid and elongate with purple coloration separated into anterior and posterior portions. Anterio-medial platelets (105–105 (105) long; 47.5–50 (47.5) wide). Anterio-lateral platelets (150–157.5 (150) long; 42.5–45 (45) wide) free from dorsal plate. Dgl-4 closer to the edge of the dorsum than to the muscle scars (distance between Dgl-4 180–200 (180)). Dorsal plate proportions: dorsum length/width 1.92–2.08 (1.92); dorsal width/distance between Dgl-4 1.44–1.48 (1.48); anterio-medial platelet length/width 2.10–2.21 (2.21); anterio-lateral platelet length/width 3.33–3.71 (3.33); anterio-lateral/anterio-medial length 1.43–1.50 (1.43).

**Gnathosoma — Subcapitulum** (285–295 (285) long (ventral); 210–222 (210) long (dorsal); 101.25–102.5 (101.25) tall) colorless. Rostrum (115–117.5 (117.5) long; 32.5–36.25 (32.5) wide) elongate. Chelicerae ((290) long) with curved fangs (45–47.5 (45) long). Subcapitular proportions: ventral length/height 2.81–2.88 (2.81); rostrum length/width 3.24–3.54 (3.24). **Pedipalps** with tuberculate ventral extensions on femora and on genua. Palpomeres: trochanter (35–37.5 (37.5) long); femur (96.25–100 (96.25) long); genu (55–57.5 (55) long); tibia (61.25–62.5 (61.25) long; 20–21.25 (20) wide); tarsus (15–17.5 (17.5) long). Palpomere proportions: femur/genu 1.74–1.75 (1.75); tibia/femur 0.63–0.64 (0.64); tibia length/width 2.94–3.06 (3.06).

**Venter** — (690–690 (690) long; 300–350 (300) wide) colorless. Gnathosomal bay (130–135 (130) long; 56.25–60 (56.25) wide). Cxgl-4 subapical. **Medial suture** (52.5–60 (60) long). **Genital plates** (142.5–145 (142.5) long; 120–122.5 (120) wide). Additional measurements: Cx-1 (250–270 (250) long (total); 120–125 (120) long (medial)); Cx-3 (240–258 (240) wide); anterior venter (205–207.5 (205) long). Ventral proportions: gnathosomal bay length/width 2.25–2.31 (2.31); anterior venter/genital field length 1.43–1.44 (1.44); anterior venter length/genital field width 1.69–1.71 (1.71); anterior venter/medial suture 3.42–3.95 (3.42).

**Male (Figure 69)** (n = 2) (allotypic measurements in parentheses when available) with characters of the genus with following specifications.

**Dorsum** — (450–460 (460) long; 265–270 (270) wide) ovoid and elongate with purple coloration separated into anterior and posterior portions. Anterio-medial platelets (92.5–100 (100) long; 45–47.5 (45) wide). Anterio-lateral platelets (130–142.5 (130) long; 43.75–45 (43.75) wide) free from dorsal plate. Dgl-4 closer to the edge of the dorsum than to the muscle scars (distance between Dgl-4 175–180 (180)). Dorsal plate proportions: dorsum length/width 1.70–1.70 (1.70); dorsal width/distance between Dgl-4 1.50–1.51 (1.50); anterio-medial platelet length/width 1.95–2.22 (2.22); anterio-lateral platelet length/width 2.97–3.17 (2.97); anterio-lateral/anterio-medial length 1.30–1.54 (1.30).

**Gnathosoma — Subcapitulum** (250–255 (255) long (ventral); 184–187 (184) long (dorsal); 85–87.5 (85) tall) colorless. Rostrum (97.5–102.5 (102.5) long; 30–30 (30) wide) elongate. Chelicerae (243–262 (243) long) with curved fangs (34–42 (42) long). Subcapitular proportions: ventral length/height 2.83–3.00 (3.00); rostrum length/width 3.25–3.42 (3.42). **Pedipalps** with tuberculate ventral extensions on femora and on genua. Palpomeres: trochanter (31.25–37.5 (31.25) long); femur (83.75–85 (83.75) long); genu (47.5–47.5 (47.5) long); tibia (57.5–60 (57.5) long; 18.75–20 (20) wide); tarsus (15–15 (15) long). Palpomere proportions: femur/genu 1.76–1.79 (1.76); tibia/femur 0.69–0.71 (0.69); tibia length/width 2.88–3.20 (2.88).

**Venter** — (565–570 (570) long; 325–329 (329) wide) colorless. Gnathosomal bay (105–110 (105) long; 50–55 (55) wide). Cxgl-4 subapical. **Medial suture** (55–80 (55) long). **Genital plates** (107.5–113.75 (113.75) long; 92.5–92.5 (92.5) wide). Additional measurements: Cx-1 (207–232 (231) long (total); 82–98 (98) long (medial)); Cx-3 (257–266 (266) wide); anterior venter (215–220 (215) long). Ventral proportions: gnathosomal bay length/width 1.91–2.20 (1.91); anterior venter/genital field length 1.89–2.05 (1.89); anterior venter length/genital field width 2.32–2.38 (2.32); anterior venter/medial suture 2.75–3.91 (3.91).

**Immatures** unknown.

######## Etymology.

Specific epithet (*elongata*) refers to elongated bodies of this species, which is more pronounced than in all other North American *Torrenticola* (*elongatus*, L. prolonged).

######## Distribution.

**Figure 67.**
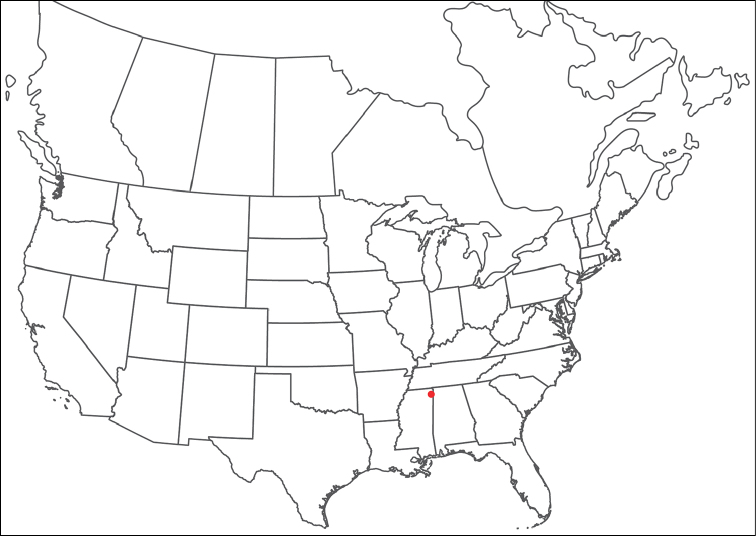
*Torrenticola elongata* sp. n. distribution.

Known only from Tishomingo County, Mississippi (Figure 67).

**Figure 68.**
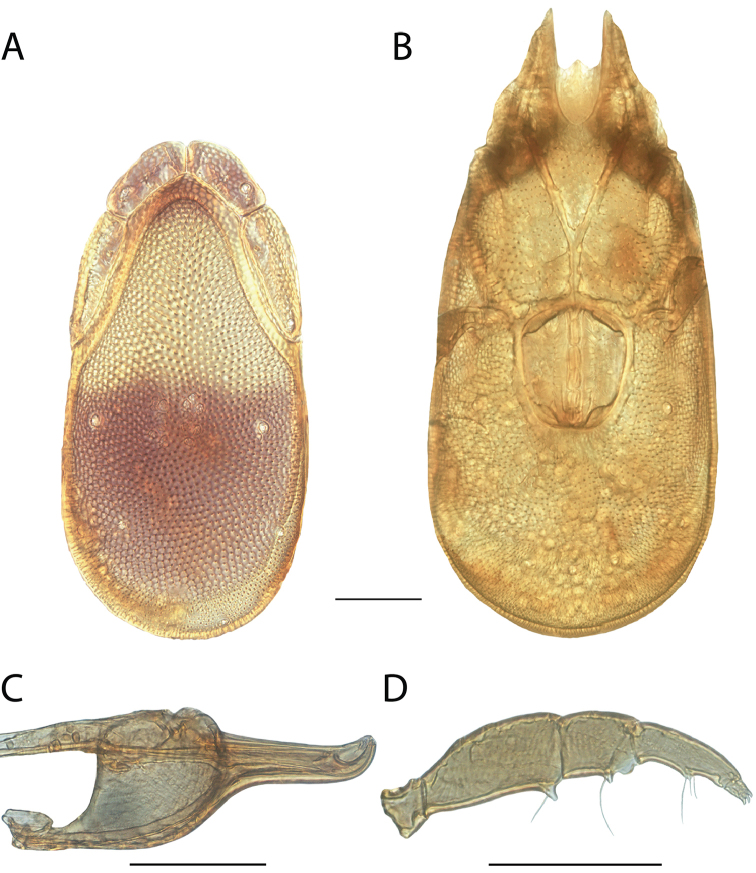
*Torrenticola elongata* sp. n. female: **A** dorsal plates **B** venter (legs removed) **C** subcapitulum **D** pedipalp (setae not accurately depicted). Scale = 100 µm.

**Figure 69.**
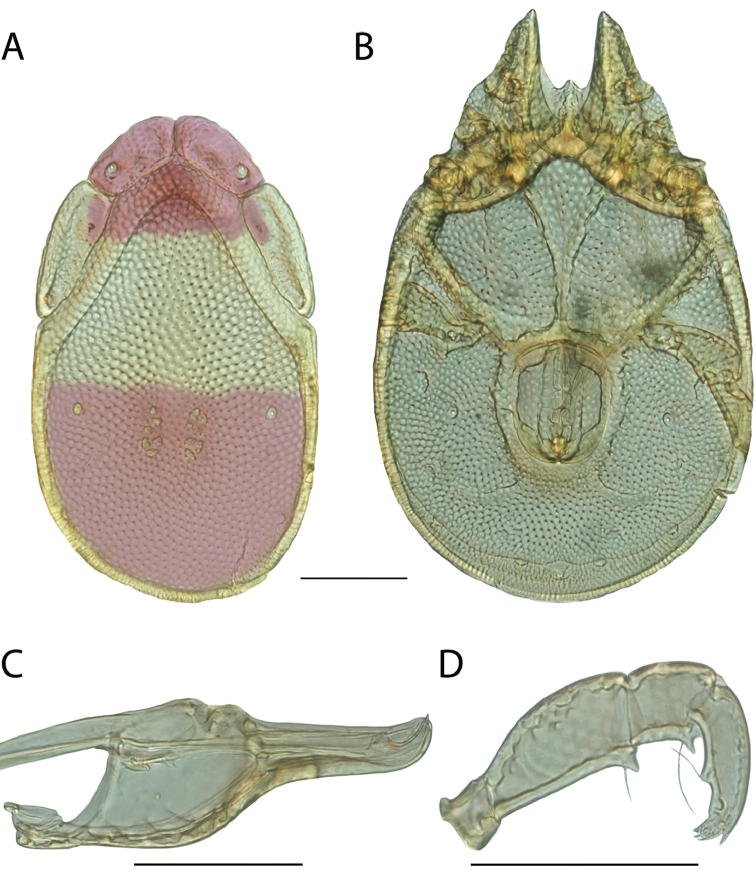
*Torrenticola elongata* sp. n. male: **A** dorsal plates, coloration added **B** venter (legs removed) **C** subcapitulum **D** pedipalp (setae not accurately depicted). Scale = 100 µm.

######## Remarks.

*Torrenticola elongata* groups with other members of the Raptor Complex with high support and specimens are less than 1% different in COI sequence from each other. In all analyses, *T. elongata* groups with two other species (*T. gorti* and *T. bondi*) which are 4% different from each other and have non-overlapping ranges.

Based upon overall similarity, an elongate body, and distribution, we place this species in the Elongata Identification Group.

This species hypothesis is supported by low COI divergence within the species (0–2%) and high divergence between species (3–15%), and by the morphological characters outlined in the diagnosis.

####### Torrenticola elusiva

Taxon classificationAnimaliaTrombidiformesTorrenticolidae

Fisher & Dowling sp. n.

http://zoobank.org/74B322FC-CA98-4BE9-AA6C-B819FD821C93

######## Material examined.

HOLOTYPE (♀): from Canada, New Brunswick, Charlotte County, Rollingham, Whittier Ridge, Highway 770, 6.6 km east of covered bridge, 3 Oct 2011, by IM Smith, IMS110120, DNA 1857.

######## Type deposition.

Holotype (♀) deposited in the CNC.

######## Diagnosis.

*Torrenticola elusiva* are similar to other members of the Raptor Group (*T. gnoma*, *T. irapalpa*, *T. longitibia*, *T. mjolniri*, *T. racupalpa*, *T. raptor*, *T. danielleae*, *T. daemon*, and *T. ivyae*) in having round bodies; Dgl-4 close to muscles scars; long, thin subcapitular rostra; and long, thin pedipalp tibiae. *T. elusiva* can be differentiated from *T. racupalpa* by having a stockier subcapitulum (ventral length/height = 2.39 in *T. elusiva*, 2.48–2.73 in *T. racupalpa*); and by dorsal pattern. *T. elusiva* can be differentiated from *T. irapalpa* and *T. daemon* by having Dgl-4 closer to the muscle scars (dorsal width/distance between Dgl-4 = 2.5 in *T. elusiva*, 1.59–2.09 in others); a more elongate rostrum (length/width = 3.65 in *T. elusiva*, 2.66–3.39 in others); and by dorsal coloration and pattern. *T. elusiva* can be differentiated from *T. gnoma* by being larger (dorsum length = 645 in *T. elusiva*, 540–595 in *T. gnoma*); having a more elongate rostrum (length/width = 3.65 in *T. elusiva*, 2.74–3.13 in *T. gnoma*); and dorsal coloration. *T. elusiva* can be differentiated from *T. mjolniri* and *T. ivyae* by having stockier pedipalp tibiae (length/width = 4.42 in *T. elusiva*, 5.00–6.00 in others); and a stockier rostrum (length/width = 3.65 in *T. elusiva*, 3.81–4.32 in others). *T. elusiva* can be differentiated from *T. raptor* by having Dgl-4 closer to the muscle scars (dorsal width/distance between Dgl-4 = 2.50 in *T. elusiva*, 1.8–2.02 in *T. raptor*); shorter anterior venter (163.75 in *T. elusiva*, 205–240 in *T. raptor*); and stockier pedipalp tibiae (length/width = 4.42 in *T. elusiva*, 6–7.54 in *T. raptor*). *T. elusiva* can be differentiated from *T. danielleae* by having Dgl-4 closer to the muscle scars (dorsal width/distance between Dgl-4 = 2.5 in *T. elusiva*, 1.57–1.70 in *T. danielleae*) and by dorsal coloration. *T. elusiva* cannot be confidently differentiated from *T. longitibia* because *T. elusiva* is only known from a single female and *T. longitibia* is only known from two males; however, *T. elusiva* is only known from Charlotte County, New Brunswick, whereas *T. longitibia* is only known from Monroe County, Tennessee. Additionally, two character systems that vary minimally between sexes are rostrum and pedipalp tibiae proportions, which do differ between *T. elusiva* and *T. longitibia* as follows: pedipalp tibia stockier (4.42 in *T. elusiva*, 5.5–5.5 in *T. longitibia*) and rostrum stockier (3.65 in *T. elusiva*, 4.15–4.23 in *T. longitibia*).

######## Description.

**Female (Figure 71)** (n = 1) (holotype only) with characters of the genus with following specifications.

**Dorsum** — (645 long; 500 wide) circular with bluish-purple coloration posteriorly with a broad anterior extension reaching the anterior edge of the dorsal plate. Anterio-medial platelets (152.5 long; 70 wide). Anterio-lateral platelets (182.5 long; 87.5 wide) free from dorsal plate. Dgl-4 much closer to the muscle scars than to edge of dorsum (distance between Dgl-4 200). Dorsal plate proportions: dorsum length/width 1.29; dorsal width/distance between Dgl-4 2.50; anterio-medial platelet length/width 2.18; anterio-lateral platelet length/width 2.09; anterio-lateral/anterio-medial length 1.20.

**Gnathosoma — Subcapitulum** (340 long (ventral); 259 long (dorsal); 142.5 tall) colorless. Rostrum (155 long; 42.5 wide) elongate. Chelicerae (333 long) with curved fangs (59 long). Subcapitular proportions: ventral length/height 2.39; rostrum length/width 3.65. **Pedipalps** elongate (especially tibiae) with tuberculate ventral extensions with dentate tip on femora and tuberculate ventral extensions on genua. Palpomeres: trochanter (48.75 long); femur (132.5 long); genu (72.5 long); tibia (105 long; 23.75 wide); tarsus (17.5 long). Palpomere proportions: femur/genu 1.83; tibia/femur 0.79; tibia length/width 4.42.

**Venter** — (730 long; 554 wide) colorless. Gnathosomal bay (176.25 long; 87.5 wide). Cxgl-4 subapical. **Medial suture** (17.5 long). **Genital plates** (167.5 long; 150 wide). Additional measurements: Cx-1 (288 long (total); 115 long (medial)); Cx-3 (384 wide); anterior venter (163.75 long). Ventral proportions: gnathosomal bay length/width 2.01; anterior venter/genital field length 0.98; anterior venter length/genital field width 1.09; anterior venter/medial suture 9.36.

**Male** unknown.

**Immatures** unknown.

######## Etymology.

Specific epithet (*elusiva*) refers to the fact that we were only able to find a single specimen of this species, despite extensive searching among the abundant samples taken from the type locality in New Brunswick.

######## Distribution.

**Figure 70.**
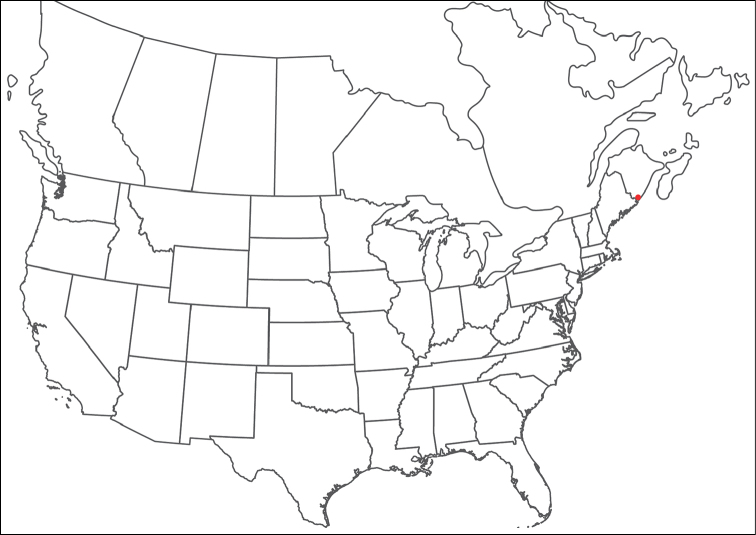
*Torrenticola elusiva* sp. n. distribution.

Known only from Charlotte County, New Brunswick (Figure 70).

**Figure 71.**
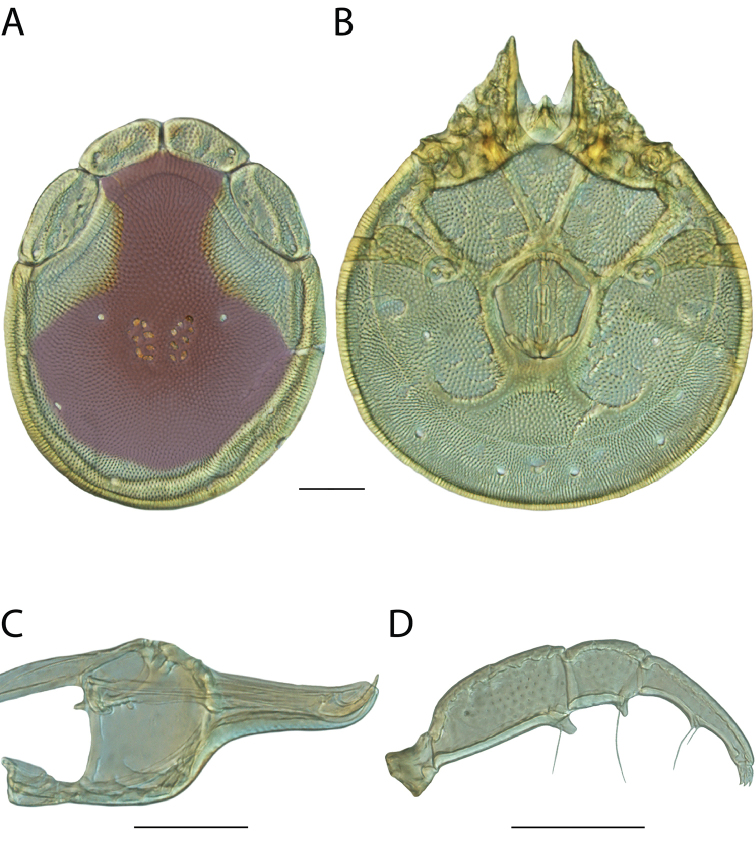
*Torrenticola elusiva* sp. n. female: **A** dorsal plates, coloration added **B** venter (legs removed) **C** subcapitulum **D** pedipalp (setae not accurately depicted). Scale = 100 µm.

######## Remarks.

*Torrenticola elusiva* groups with other members of the Raptor Complex in all analyses with high support. Only one specimen could be acquired for use in our analyses, so differences in COI sequence across specimens could not be investigated, but this single specimen was greater than 4% different in COI sequence from sister species. Furthermore, this species is known from only a single female, so morphological variation could not be investigated. However, this specimen was different enough in terms of morphology and sequence to warrant a separate description. We place this species within the Raptor Identification group based upon similarity with those species.

This species hypothesis is supported by high divergence between species (3–15%), and by the morphological characters outlined in the diagnosis.

####### Torrenticola erectirostra

Taxon classificationAnimaliaTrombidiformesTorrenticolidae

Fisher & Dowling sp. n.

http://zoobank.org/CD5D74ED-B8CA-44A9-863F-15C3028F6580

######## Material examined.

HOLOTYPE (♀): from Canada, New Brunswick, York County, Stanley, Nashwaak River, Stanley Municipal Park, 19 Jun 2012, by IM Smith, IMS120031, DNA 2962.

PARATYPES (9 ♀; 9 ♂): **New Brunswick, Canada**: 3 ♂ from Charlotte County, Rollingham, Digdeguash River, beside Highway 770 at covered bridge, 30 Jun 1989, by IM Smith, IMS890053 • 1 ♀ and 2 ♂ from Charlotte County, Digdeguash River, beside Sorrel Ridge Road west of Whittier Road, 10 Jun 2012, by IM Smith, IMS120015 • 4 ♀ and 4 ♂ from York County, Magaguadavic River, beside Highway 3 just east of Thomaston Corners, 1 Jul 1989, by IM Smith, IMS890055A • 1 ♂ (ALLOTYPE) from York County, Stanley, Davis Brook, beside Highway 3, 3.5 km south of Highway 4 at Thomaston Corner, 11 Jun 2012, by IM Smith, IMS120017, DNA 2964 • 2 ♀ and 1 ♂ from New Brunswick, York County, Stanley, Davis Brook, beside Highway 3, 3.5 km south of Highway 4 at Thomaston Corner, 11 Jun 2012, by IM Smith, IMS120017 • **Maine, USA**: 1 ♀ from Aroostook County, Ashland, beside Route 11, Aroostook River (46°38'N 68°24'W), 4 Jul 1989, by IM Smith, IMS890067 • **New York, USA**: 1 ♂ from Cayuga County, Dutch Hollow Brook, beside Route 38A at Niles, 22 Jul 1990, by IM Smith, IMS900113A • **Virginia, USA**: 1 ♀ from Amherst County, beside Blue Ridge, Otter Creek (37°36'57"N, 79°19'27"W), 7 Sep 2007, by IM Smith, IMS070056A.

######## Type deposition.

Holotype (♀), allotype (♂), and most paratypes (6 ♀; 4 ♂) deposited in the CNC; other paratypes (3 ♀; 4 ♂) deposited in the ACUA.

######## Diagnosis.

*Torrenticola erectirostra* are similar to other members of the Erectirostra Group (*T. karambita* and *T. robisoni*), species with similar dorsal patterning, such as Rusetria “4-Plate” group (*T. dunni*, *T. glomerabilis*, *T. kittatinniana*, *T. pollani*, *T. rufoalba* and *T. shubini*), Elongata Group (*T. gorti* and *T. elongata*), Neoanomala Group (*T. interiorensis* and *T. neoanomala*), *T. bondi*, *T. irapalpa*, *T. racupalpa*, and *T. skvarlai*. They can be differentiated from all other *Torrenticola*, except *T. karambita* and *T. robisoni*, by having a dentate, upturned rostrum that is wide when viewed ventrally. *T. erectirostra* can be differentiated from *T. karambita* by having dorsal coloration (*T. karambita* is colorless) and a slightly more elongate rostrum (length/width ♀ = 1.72–1.91 in *T. erectirostra*, 1.57–1.62 in *T. karambita*; ♂ = 2.0–2.2 in *T. erectirostra*, 1.6–1.95 in *T. karambita*). *T. erectirostra* can be differentiated from *T. robisoni* by having less elongate anterio-lateral platelets (length/width ♀ = 2.52–2.69 in *T. erectirostra*, 2.96–3.00 in *T. robisoni*) and by being distributed in the Appalachians, while *T. robisoni* is in the Interior Highlands.

######## Description.

**Female (Figure 73)** (n = 5) (holotype measurements in parentheses when available) with characters of the genus with following specifications.

**Dorsum**— (690–750 (735) long; 480–510 (510) wide) ovoid with bluish-purple or purple coloration separated into anterior and posterior portions with orange medially. Anterio-medial platelets (150–165 (162.5) long; 62.5–75 (75) wide). Anterio-lateral platelets (195–225 (220) long; 77.5–83.75 (82.5) wide) free from dorsal plate. Dgl-4 closer to the edge of the dorsum than to the muscle scars (distance between Dgl-4 325–370 (370)). Dorsal plate proportions: dorsum length/width 1.41–1.47 (1.44); dorsal width/distance between Dgl-4 1.38–1.51 (1.38); anterio-medial platelet length/width 2.17–2.48 (2.17); anterio-lateral platelet length/width 2.52–2.69 (2.67); anterio-lateral/anterio-medial length 1.26–1.36 (1.35).

**Gnathosoma — Subcapitulum** (315–350 (350) long (ventral); 225–247.5 (247.5) long (dorsal); 130–140 (130) tall) colorless. Rostrum (105–125 125) long; 55–72.5 (72.5) wide) wide and upturned with dentation. Chelicerae (320–345 (345) long) with curved fangs (45–55 (45) long). Subcapitular proportions: ventral length/height 2.42–2.69 (2.69); rostrum length/width 1.72–1.91 (1.72). **Pedipalps** short and stocky (especially tibiae) with tuberculate ventral extensions on femora and genua. Palpomeres: trochanter (45–55 (50) long); femur (100–107.5 (102.5) long); genu (57.5–67.5 (57.5) long); tibia (50–65 (62.5) long; 27.5–30 (28.75) wide); tarsus (17.5–20 (20) long). Palpomere proportions: femur/genu 1.48–1.78 (1.78); tibia/femur 0.50–0.61 (0.61); tibia length/width 1.82–2.17 (2.17).

**Venter** — (860–920 (900) long; 580–650 (650) wide) colorless. Gnathosomal bay (210–220 long; 105–150 wide). Cxgl-4 far from apex. **Medial suture** (17.5–27.5 (22.5) long). **Genital plates** (187.5–202.5 (202.5) long; 162.5–180 (180) wide). Additional measurements: Cx-1 (330–360 (350) long (total); 140–160 (160) long (medial)); Cx-3 (410–460 (460) wide); anterior venter (192.5–220 (220) long). Ventral proportions: gnathosomal bay length/width 1.47–2.00; anterior venter/genital field length 0.98–1.09 (1.09); anterior venter length/genital field width 1.13–1.25 (1.22); anterior venter/medial suture 7.09–12.14 (9.78).

**Male (Figure 74)** (n = 5) (allotypic measurements in parentheses when available) with characters of the genus with following specifications.

**Dorsum** — (580–640 (620) long; 400–430 (400) wide) ovoid with bluish-purple or purple coloration separated into anterior and posterior portions with orange medially. Anterio-medial platelets (130–150 (138.75) long; 52.5–58.75 (58.75) wide). Anterio-lateral platelets (187.5–205 (205) long; 62.5–70 (68.75) wide) free from dorsal plate. Dgl-4 closer to the edge of the dorsum than to the muscle scars (distance between Dgl-4 275–305 (300)). Dorsal plate proportions: dorsum length/width 1.41–1.55 (1.55); dorsal width/distance between Dgl-4 1.33–1.45 (1.33); anterio-medial platelet length/width 2.26–2.73 (2.36); anterio-lateral platelet length/width 2.78–3.20 (2.98); anterio-lateral/anterio-medial length 1.37–1.48 (1.48).

**Gnathosoma — Subcapitulum** (270–292.5 (285) long (ventral); 175–215 (197.5) long (dorsal); 96.25–110 (105) tall) colorless. Rostrum (90–107.5 (98.75) long; 45–50 (46.25) wide) wide and upturned with dentation. Chelicerae (265–285 (265) long) with curved fangs (45–50 (50) long). Subcapitular proportions: ventral length/height 2.45–2.86 (2.71); rostrum length/width 2.00–2.17 (2.14). **Pedipalps** short and stocky (especially tibiae) with tuberculate ventral extensions on femora and genua. Palpomeres: trochanter (42.5–47.5 (42.5) long); femur (80–91.25 (87.5) long); genu (55–57.5 (55) long); tibia (50–57.5 (50) long; 23.75–27.5 (23.75) wide); tarsus (15–17.5 (15) long). Palpomere proportions: femur/genu 1.39–1.59 (1.59); tibia/femur 0.57–0.63 (0.57); tibia length/width 2.00–2.11 (2.11).

**Venter** — (720–780 (750) long; 470–495 (470) wide) colorless. Gnathosomal bay (167.5–177.5 (172.5) long; 100–105 (105) wide). Cxgl-4 far from apex. **Medial suture** (75–82.5 (75) long). **Genital plates** (152.5–165 (157.5) long; 112.5–125 (112.5) wide). Additional measurements: Cx-1 (290–330 (310) long (total); 125–150 (140) long (medial)); Cx-3 (360–390 (360) wide); anterior venter (232.5–250 (250) long). Ventral proportions: gnathosomal bay length/width 1.60–1.78 (1.64); anterior venter/genital field length 1.47–1.64 (1.59); anterior venter length/genital field width 1.94–2.22 (2.22); anterior venter/medial suture 2.94–3.33 (3.33).

**Immatures** unknown.

######## Etymology.

Specific epithet (*erectirostra*) refers to the upturned rostrum characteristic of members of the Erectirostra Group (*erectus*, raised up; *rostrum*, L. snout).

######## Distribution.

**Figure 72.**
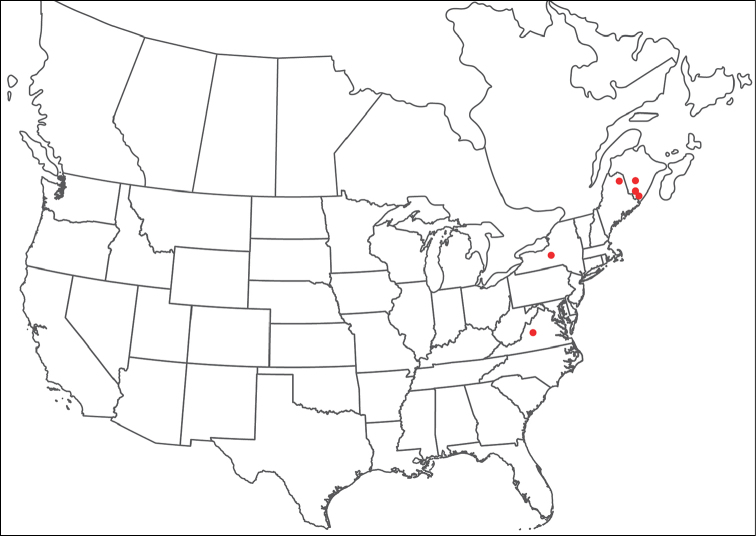
*Torrenticola erectirostra* sp. n. distribution.

Appalachians (Figure 72).

**Figure 73.**
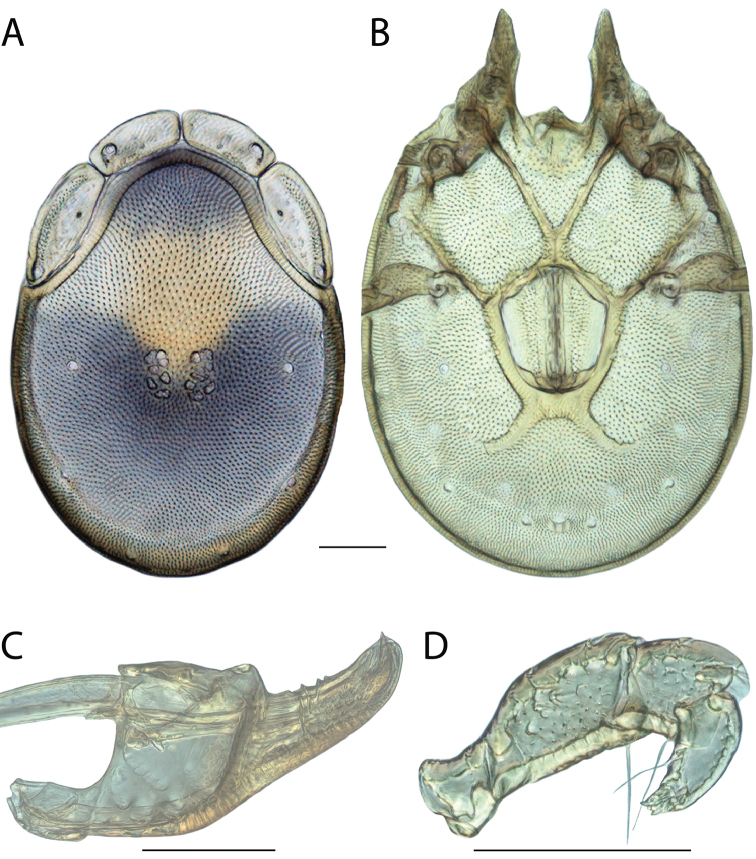
*Torrenticola erectirostra* sp. n. female: **A** dorsal plates **B** venter (legs removed) **C** subcapitulum **D** pedipalp (setae not accurately depicted). Scale = 100 µm.

**Figure 74.**
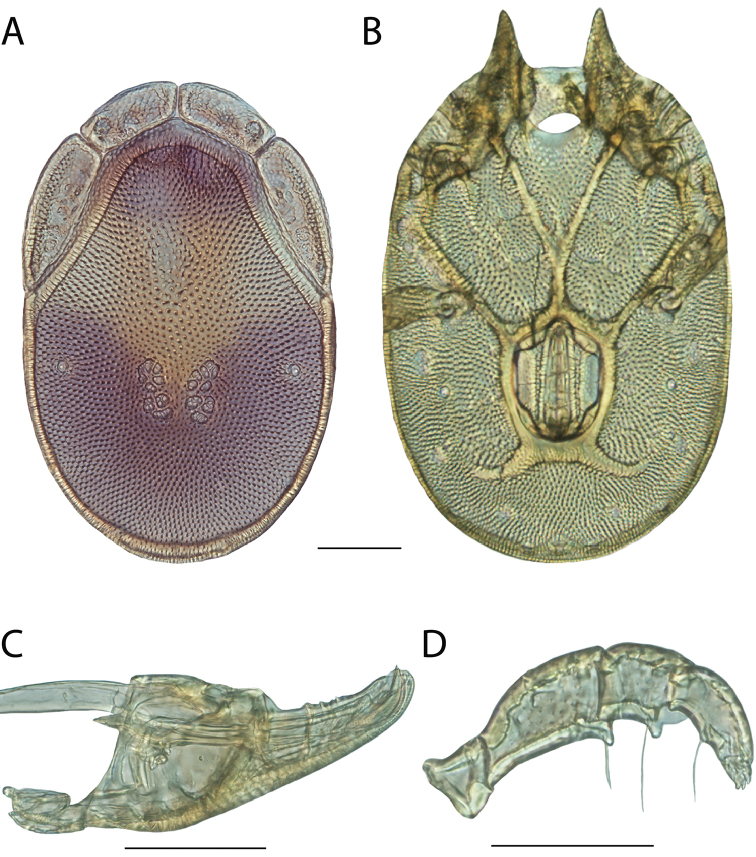
*Torrenticola erectirostra* sp. n. male: **A** dorsal plates **B** venter (legs removed) **C** subcapitulum **D** pedipalp (setae not accurately depicted). Scale = 100 µm.

######## Remarks.

*Torrenticola erectirostra* groups with other members of the Raptor Complex with high support and specimens are 2.7% different from each other in COI sequence. This variation in COI is higher than in most species hypotheses proposed herein, especially since those specimens were form the same region (New Brunswick). However, we could not find morphological differences that corresponded to clades in our analysis, and we were only able to examine four sequence, therefore, we consider these specimens to represent a single species hypothesis.

This species groups with *T. karambita* and *T. robisoni* to form the Erectirostra Identification Group, which can be readily identified by the shape of the rostrum.

This species hypothesis is supported by biogeography, molecular divergence (although COI variation is greater than most hypotheses herein), and by the morphological characters outlined in the diagnosis.

####### Torrenticola feminellai

Taxon classificationAnimaliaTrombidiformesTorrenticolidae

Fisher & Dowling sp. n.

http://zoobank.org/2978C7F1-76AE-4DAE-BDB8-550F75271E50

######## Material examined.

HOLOTYPE (♀): from USA, Georgia, Chattooga County, Cloudland; beside Rt. 48 just east of Alabama state line, (34°31'31"N, 85°30'30"W), 29 September 2005, by IM Smith, IMS050110A.

PARATYPES (4 ♀; 6 ♂): **Georgia, USA** : 2 ♀ and 4 ♂ from Chattooga County, Cloudland; beside Rt. 48 just east of Alabama State line, (34°31'31"N, 85°30'30"W), 28 September 1992, by IM Smith, IMS920056A • 1 ♂ (ALLOTYPE) from Chattooga County, Cloudland; beside Rt. 48 just east of Alabama state line, (34°31'31"N, 85°30'30"W), 29 September 2005, by IM Smith, IMS050110A • 2 ♀ and 1 ♂ from Chattooga County, Cloudland; beside Rt. 48 just east of Alabama state line, (34°31'31"N, 85°30'30"W), 29 September 2005, by IM Smith, IMS050110A.

######## Type deposition.

Holotype (♀), allotype (♂), and some paratypes (2 ♀; 3 ♂) deposited in the CNC; other paratypes (2 ♀; 2 ♂) deposited in the ACUA.

######## Diagnosis.

*Torrenticola feminellai* are similar to other members of the Rusetria “Eastern 2-Plates” group (*T. biscutella*, *T. caerulea*, *T. delicatexa*, *T. indistincta*, *T. malarkeyorum*, *T. pendula*, *T. sellersorum*, *T. tysoni*, *T. ululata*, *T. whitneyae*, and *T. microbiscutella*) in having anterio-lateral platelets fused to the dorsal plate, having dorsal coloration separated into anterior and posterior portions (except *T. ululata* and *T. indistincta*), and being distributed in the east. *T. feminellai* can be differentiated from all other Eastern 2-Plates by having a more elongate rostrum (length/width ♀ = 3.05–3.38 in *T. feminellai*, 2.33–3.00 in others; ♂ = 3.14–3.38 in *T. feminellai*, 2.50–3.05 in others), except *T. tysoni* (3.06–3.50). Additionally, *T. feminellai* can be differentiated from all other Eastern 2-Plates by having a distinct dorsal pattern.

######## Description.

**Female (Figure 76)** (n = 5) (holotype measurements in parentheses when available) with characters of the genus with following specifications.

**Dorsum** — (590–690 (640) long; 470–540 (500) wide) circular with reddish-purple coloration in the shape of an hourglass. Anterio-medial platelets (137.5–150 (150) long; 52.5–55 (55) wide). Anterio-lateral platelets (185–202.5 (202.5) long; 75–80 (75) wide) partially fused to dorsal plate. Dgl-4 approximately halfway between the edge of the dorsum and the muscle scars (distance between Dgl-4 290–310 (290)). Dorsal plate proportions: dorsum length/width 1.24–1.33 (1.28); dorsal width/distance between Dgl-4 1.59–1.74 (1.72); anterio-medial platelet length/width 2.62–2.76 (2.73); anterio-lateral platelet length/width 2.34–2.70 (2.70); anterio-lateral/anterio-medial length 1.25–1.36 (1.35).

**Gnathosoma — Subcapitulum** (320–357.5 (352.5) long (ventral); 245–275 (267.5) long (dorsal); 140–160 (160) tall) colorless. Rostrum (135–157.5 (145) long; 40–47.5 (47.5) wide). Chelicerae (335–375 (375) long) with curved fangs (65–75 (70) long). Subcapitular proportions: ventral length/height 2.17–2.30 (2.20); rostrum length/width 3.05–3.38 (3.05). **Pedipalps** with tuberculate ventral extensions on femora and genua. Palpomeres: trochanter (46.25–52.5 (52.5) long); femur (117.5–140 (140) long); genu (65–77.5 (75) long); tibia (90–100 (100) long; 22.5–25 (25) wide); tarsus (17.5–20 (20) long). Palpomere proportions: femur/genu 1.73–1.87 (1.87); tibia/femur 0.71–0.77 (0.71); tibia length/width 3.85–4.11 (4.00).

**Venter** — (670–760 (700) long; 550–690 (550) wide) colorless. Gnathosomal bay (177.5–195 (195) long; 80–115 (80) wide). Cxgl-4 subapical. **Medial suture** absent. **Genital plates** (185–195 (195) long; 167.5–180 (170) wide). Additional measurements: Cx-1 (310–320 (320) long (total); 120–140 (125) long (medial)); Cx-3 (340–410 (360) wide); anterior venter (130–140 (130) long). Ventral proportions: gnathosomal bay length/width 1.61–2.44 (2.44); anterior venter/genital field length 0.67–0.76 (0.67); anterior venter length/genital field width 0.74–0.82 (0.76).

**Male (Figure 77)** (n = 5) (allotypic measurements in parentheses when available) with characters of the genus with following specifications.

**Dorsum** — (520–565 (545) long; 370–410 (390) wide) circular with reddish-purple coloration in the shape of an hourglass. Anterio-medial platelets (115–126.25 (122.5) long; 42.5–50 (45) wide). Anterio-lateral platelets (150–180 (155) long; 55–67.5 (60) wide) partially fused to dorsal plate. Dgl-4 approximately halfway between the edge of the dorsum and the muscle scars (distance between Dgl-4 215–250 (245)). Dorsal plate proportions: dorsum length/width 1.36–1.42 (1.40); dorsal width/distance between Dgl-4 1.54–1.72 (1.59); anterio-medial platelet length/width 2.42–2.82 (2.72); anterio-lateral platelet length/width 2.58–2.77 (2.58); anterio-lateral/anterio-medial length 1.27–1.43 (1.27).

**Gnathosoma — Subcapitulum** (270–300 (290) long (ventral); 200–227.5 (215) long (dorsal); 105–120 (115) tall) colorless. Rostrum (110–120 (120) long; 32.5–37.5 (37.5) wide). Chelicerae (270–310 (295) long) with curved fangs (50–60 (55) long). Subcapitular proportions: ventral length/height 2.50–2.59 (2.52); rostrum length/width 3.14–3.38 (3.20). **Pedipalps** with tuberculate ventral extensions on femora and genua. Palpomeres: trochanter (35–45 (42.5) long); femur (95–112.5 (105) long); genu (60–65 (62.5) long); tibia (77.5–86.25 (85) long; 20–23.75 (21.25) wide); tarsus (15–17.5 (17.5) long). Palpomere proportions: femur/genu 1.58–1.75 (1.68); tibia/femur 0.76–0.82 (0.81); tibia length/width 3.63–4.00 (4.00).

**Venter** — (610–685 (640) long; 415–470 (470) colorless. Gnathosomal bay (135–160 (155) long; 62.5–70 (62.5) wide). Cxgl-4 subapical. **Medial suture** (65–75 (70) long). **Genital plates** (130–140 (140) long; 115–125 (120) wide). Additional measurements: Cx-1 (245–290 (270) long (total); 105–130 (120) long (medial)); Cx-3 (285–315 (315) wide); anterior venter (190–205 (195) long). Ventral proportions: gnathosomal bay length/width 2.00–2.48 (2.48); anterior venter/genital field length 1.39–1.49 (1.39); anterior venter length/genital field width 1.58–1.72 (1.63); anterior venter/medial suture 2.60–2.93 (2.79).

**Immatures** unknown.

######## Etymology.

Specific epithet (*feminellai*) named in honor of Jack Feminella, professor of biology at Auburn University, who believed in me (JRF) enough to employ me as a lab technician in stream ecology, write a winning recommendation letter for graduate school, and was the first to teach me how to conduct self-directed research.

######## Distribution.

**Figure 75.**
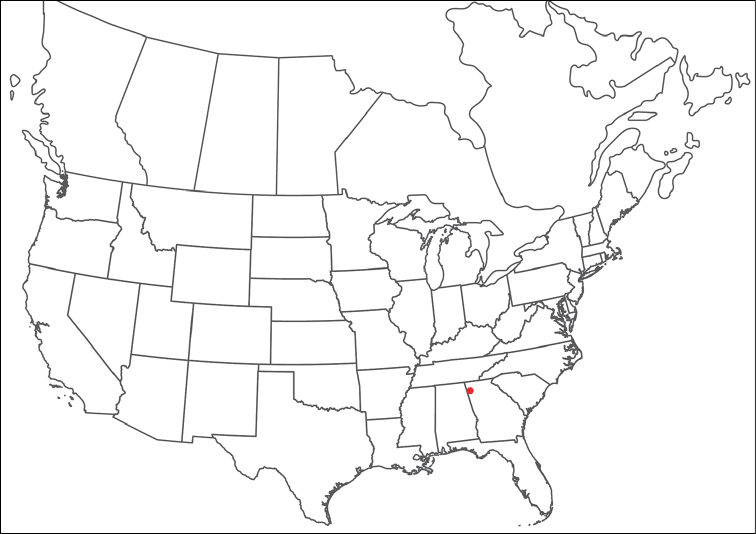
*Torrenticola feminellai* sp. n. distribution.

Southern Appalachians, Georgia (Figure 75).

**Figure 76.**
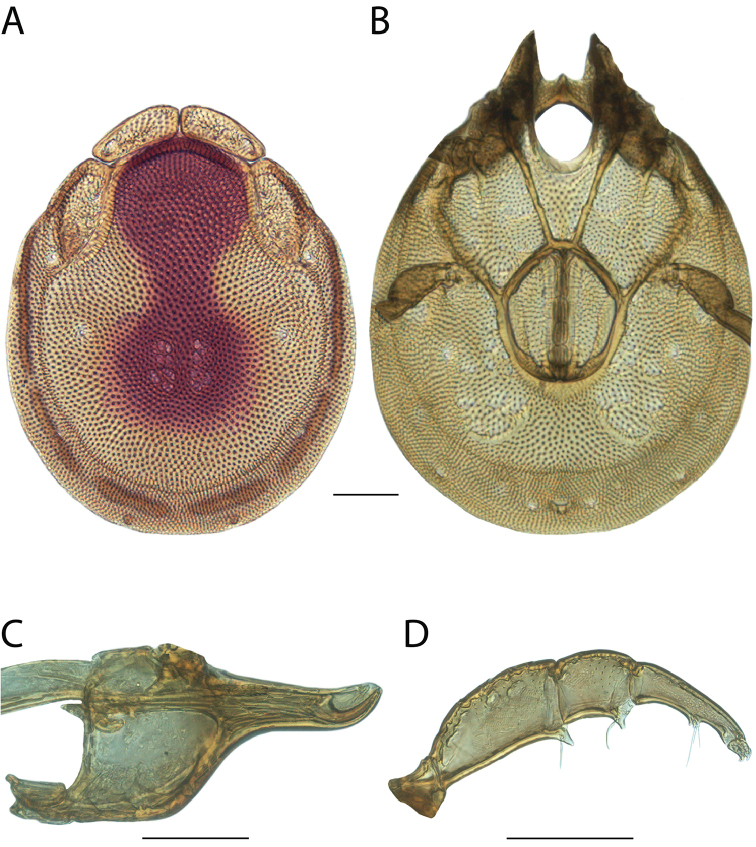
*Torrenticola feminellai* sp. n. female: **A** dorsal plates **B** venter (legs removed) **C** subcapitulum **D** pedipalp (setae not accurately depicted). Scale = 100 µm.

**Figure 77.**
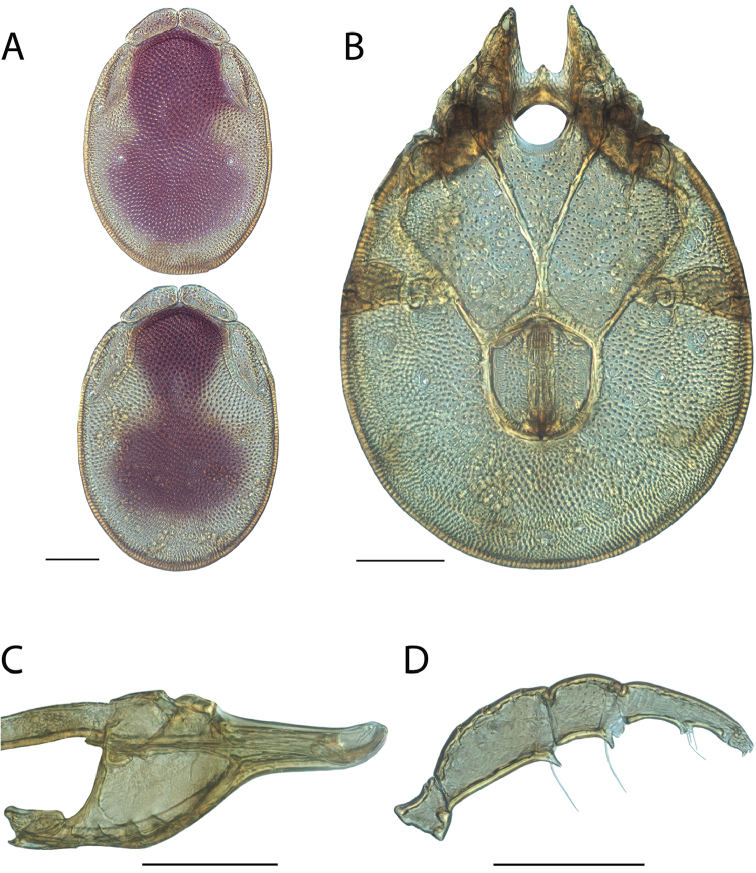
*Torrenticola feminellai* sp. n. male: **A** dorsal plates **B** venter (legs removed) **C** subcapitulum **D** pedipalp (setae not accurately depicted). Scale = 100 µm.

######## Remarks.

Unfortunately, we were unable to acquire fresh material of *Torrenticola feminellai* and therefore this species is not included in our phylogenetic analyses. However, we were able to examine morphology with material preserved in GAW. The overall similarity, distribution in the east, and fusion of the dorso-lateral platelets to the dorsal plate, are consistent with placing this species in the Rusetria Complex and the Eastern 2-Plate Identification Group.

####### Torrenticola flangipalpa

Taxon classificationAnimaliaTrombidiformesTorrenticolidae

Fisher & Dowling sp. n.

http://zoobank.org/FDF11FC8-56B5-49BE-B5FC-CF7D42E9213E

######## Material examined.

HOLOTYPE (♀): from USA, Alabama, Lauderdale County, Natchez Trace Parkway, (34°56'31"N, 87°49'41"W), 27 Sep 2010, by IM Smith, IMS100162, DNA 1310.

PARATYPES (5 ♀; 7 ♂): **Alabama, USA**: 3 ♂ from Lauderdale County, Natchez Trace Parkway, (34°56'31"N, 87°49'41"W), 24 Sep 2009, by IM Smith, IMS090121 • 1 ♂ (ALLOTYPE) from Lauderdale County, Natchez Trace Parkway, (34°56'31"N, 87°49'41"W), 27 Sep 2010, by IM Smith, IMS100162, DNA 1309 • 1 ♀ and 2 ♂ from Lauderdale County, Natchez Trace Parkway, (34°56'31"N, 87°49'41"W), 27 Sep 2010, by IM Smith, IMS100162 • **Arkansas, USA**: 1 ♀ from Carroll County, beside Route 62, Kings River, 21 Jul 1960, by DR Cook, DRC600026 • **Kentucky, USA**: 1 ♀ from Larue County, beside Route 31E, 4 kilometers south of Route 61 at Hodgenville (37°35'N, 85°42'W), 28 May 1992, by IM Smith, IMS920014 • **North Carolina, USA**: 1 ♀ and 1 ♂ from Haywood County, Great Smokey Mountains National Park, Big Creek (35°45'3.92"N, 83°6'31.67"W), 15 Sep 2009, by AJ Radwell, AJR090008A • **Tennessee, USA**: 1 ♀ from Sevier County, Great Smokey Mountains National Park, Sugarlands Nature Trail (35°40'47"N, 83°31'52"W), 7 Sep 2009, by IM Smith, IMS090101.

######## Type deposition.

Holotype (♀), allotype (♂), and most paratypes (3 ♀; 4 ♂) deposited in the CNC; other paratypes (2 ♀; 2 ♂) deposited in the ACUA.

######## Diagnosis.

*Torrenticola flangipalpa* are similar to other members of the Nigroalba Group (*T. nigroalba*, *T. solisorta*, and *T. dentirostra*) in being small, slightly elongate, having purple dorsal coloration restricted posteriorly, and having distinct yet poorly-defined hind coxal margins (can be indistinct, at least in males). *T. flangipalpa* are best differentiated from other members of the Nigroalba Group by having a flange-like, anteriorly-directed pedipalp femoral extension (this extension is tuberculate in other members of the Nigroalba Group). Additionally, *T. flangipalpa* can be differentiated from *T. nigroalba* and *T. solisorta* by having a longer anterior venter (235–265 in *T. flangipalpa*; 192–225 in others) and stockier pedipalpal tibiae (length/width ♀ = 4.79–5.0 in *T. flangipalpa*, 5.38–5.83 in others; length/width ♂ = 4.4–4.86 in *T. flangipalpa*, 5.08–5.33 in others). *T. flangipalpa* can be differentiated from *T. dentirostra* by having a smooth rostrum (*T. dentirostra* has a dentate bump midway on the dorsal edge of the rostrum). Other *Torrenticola* with purple dorsal coloration restricted posteriorly, such as *T. tahoei* and *T. oregonensis* are larger (dorsum length ♀ = 530–565 in *T. flangipalpa*, 600–810 in others; ♂ = 480–510 in *T. flangipalpa*, 560–820 in others) and distributed in the west (*T. flangipalpa* is only known from Alabama and Tennessee).

######## Description.

**Female (Figure 79)** (n = 5) (holotype measurements in parentheses when available) with characters of the genus with following specifications.

**Dorsum** — (530–580 (545) long; 365–425 (380) wide) ovoid with purple or bluish-purple coloration restricted posteriorly. Anterio-medial platelets (112.5130 (127.5) long; 47.5–57.5 (47.5) wide). Anterio-lateral platelets (157.5–175 (172.5) long; 55–62.5 (55) wide) free from dorsal plate. Dgl-4 closer to the edge of the dorsum than to the muscle scars (distance between Dgl-4 255–295 (255)). Dorsal plate proportions: dorsum length/width 1.33–1.45 (1.43); dorsal width/distance between Dgl-4 1.39–1.49 (1.49); anterio-medial platelet length/width 2.25–2.74 (2.68); anterio-lateral platelet length/width 2.86–3.14 (3.14); anterio-lateral/anterio-medial length 1.29–1.40 (1.35).

**Gnathosoma — Subcapitulum** (305–330 (307.5) long (ventral); 225–255 (225) long (dorsal); 90–95 (90) tall) elongate and colorless. Rostrum (112.5–130 (117.5) long; 37.5–42.5 (37.5) wide) elongate. Chelicerae (285–320 (286) long) with curved fangs (43–50 (44) long). Subcapitular proportions: ventral length/height 3.39–3.47 (3.42); rostrum length/width 2.82–3.06 (3.00). **Pedipalps** elongate (especially tibiae) with broad, dentate, anteriorly-directed flange on femora and with variable, dentate flange-like extension on genua. Palpomeres: trochanter (32.5–35 (35) long); femur (93.75–102.5 (93.75) long); genu (55–62.5 (55) long); tibia (80–90 (83.75) long; 16.25–18.75 (17.5) wide); tarsus (15–17.5 (15) long). Palpomere proportions: femur/genu 1.60–1.70 (1.70); tibia/femur 0.82–0.90 (0.89); tibia length/width 4.79–5.00 (4.79).

**Venter** — (680–750 (680) long; 430–495 (436) wide) with faint purple or bluish-purple coloration. Gnathosomal bay (112.5–140 (120) long; 67.5–75 (67.5) wide). Cxgl-4 far from apex. **Medial suture** (67.5–80 (67.5) long). **Genital plates** (145–160 (151.25) long; 125–132.5 (125) wide). Additional measurements: Cx-1 (266–310 (266) long (total); 150–170 (156) long (medial)); Cx-3 (278–321 (278) wide); anterior venter (235–255 (245) long). Ventral proportions: gnathosomal bay length/width 1.50–2.00 (1.78); anterior venter/genital field length 1.55–1.76 (1.62); anterior venter length/genital field width 1.86–2.04 (1.96); anterior venter/medial suture 3.06–3.78 (3.63).

**Male (Figure 80)** (n = 6) (allotypic measurements in parentheses when available) with characters of the genus with following specifications.

**Dorsum** — (480–510 (480) long; 330–370 (330) wide) ovoid with purple or bluish-purple coloration restricted posteriorly. Anterio-medial platelets (112.5–122.5 (115) long; 41.25–47.5 (41.25) wide). Anterio-lateral platelets (152.5–162.5 (152.5) long; 50–53.75 (50) wide) free from dorsal plate. Dgl-4 closer to the edge of the dorsum than to the muscle scars (distance between Dgl-4 235–260 (235)). Dorsal plate proportions: dorsum length/width 1.38–1.45 (1.45); dorsal width/distance between Dgl-4 1.39–1.44 (1.40); anterio-medial platelet length/width 2.42–2.79 (2.79); anterio-lateral platelet length/width 3.02–3.10 (3.05); anterio-lateral/anterio-medial length 1.33–1.44 (1.33).

**Gnathosoma — Subcapitulum** (272.5–290 (272.5) long (ventral); 200–209 (200) long (dorsal); 75–87.5 (75) tall) elongate and colorless. Rostrum (102.5–107.5 (105) long; 32.5–35 (35) wide) elongate. Chelicerae (250–272 (260) long) with curved fangs (39–62 (40) long). Subcapitular proportions: ventral length/height 3.31–3.68 (3.63); rostrum length/width 2.93–3.31 (3.00). **Pedipalps** elongate (especially tibiae) with broad, dentate, anteriorly-directed flange on femora and with variable, dentate flange-like extension on genua. Palpomeres: trochanter (27.5–31.25 (27.5) long); femur (85–90 (87.5) long); genu 47.5–57.5 (55) long); tibia (77.5–85 (80) long; 17.5–18.75 (17.5) wide); tarsus (12.5–15 (12.5) long). Palpomere proportions: femur/genu 1.52–1.79 (1.59); tibia/femur 0.91–0.94 (0.91); tibia length/width 4.40–4.86 (4.57).

**Venter** — (600–640 (600) long; 356–420 (380) wide) with faint purple or bluish-purple coloration. Gnathosomal bay (100–112.5 (105) long; 65–72.5 (65) wide). Cxgl-4 far from apex. **Medial suture** (82.5–107.5 (95) long). **Genital plates** (122.5–127.5 (122.5) long; 92.5–100 (95) wide). Additional measurements: Cx-1 (240–258 (240) long (total); 122–160 (135) long (medial)); Cx-3 (251–291 (265) wide); anterior venter (245–265 (245) long). Ventral proportions: gnathosomal bay length/width 1.48–1.62 (1.62); anterior venter/genital field length 1.98–2.12 (2.00); anterior venter length/genital field width 2.55–2.68 (2.58); anterior venter/medial suture 2.42–3.00 (2.58).

**Immatures** unknown.

######## Etymology.

Specific epithet (*flangipalpa*) refers the expanded (i.e., flanged) pedipalp femoral tubercle (*flange*, English; *palpus*, L. hand, feeler), which distinguishes this species from other members of the Nigroalba Group.

######## Distribution.

**Figure 78.**
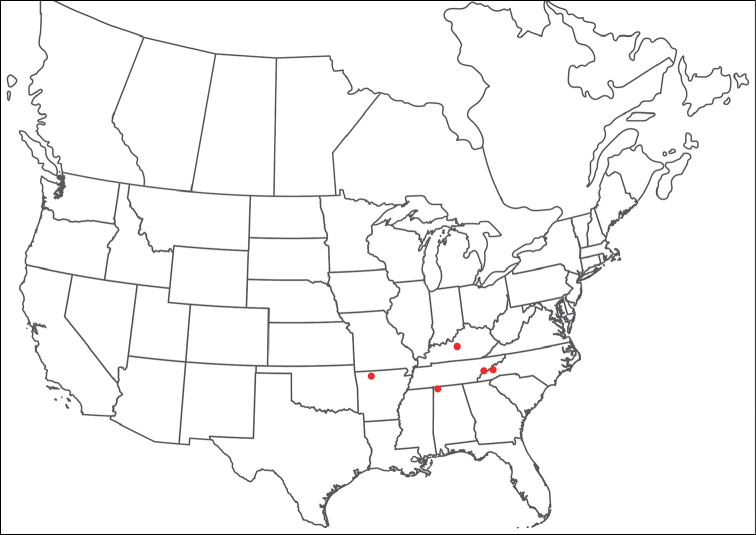
*Torrenticola flangipalpa* sp. n. distribution.

Southeastern (Figure 78).

**Figure 79.**
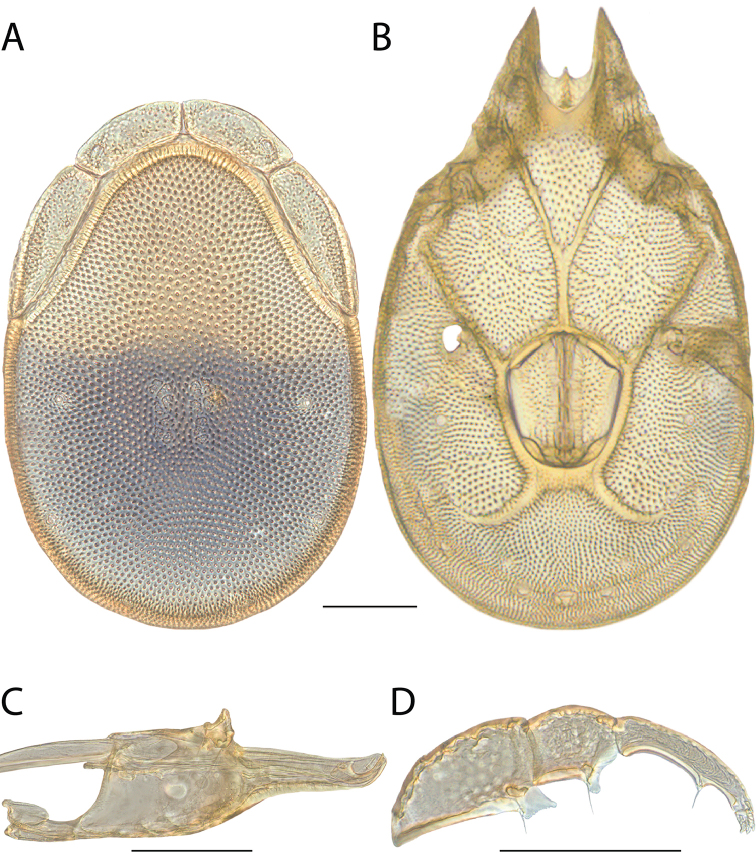
*Torrenticola flangipalpa* sp. n. female: **A** dorsal plates **B** venter (legs removed) **C** subcapitulum **D** pedipalp (setae not accurately depicted). Scale = 100 µm.

**Figure 80.**
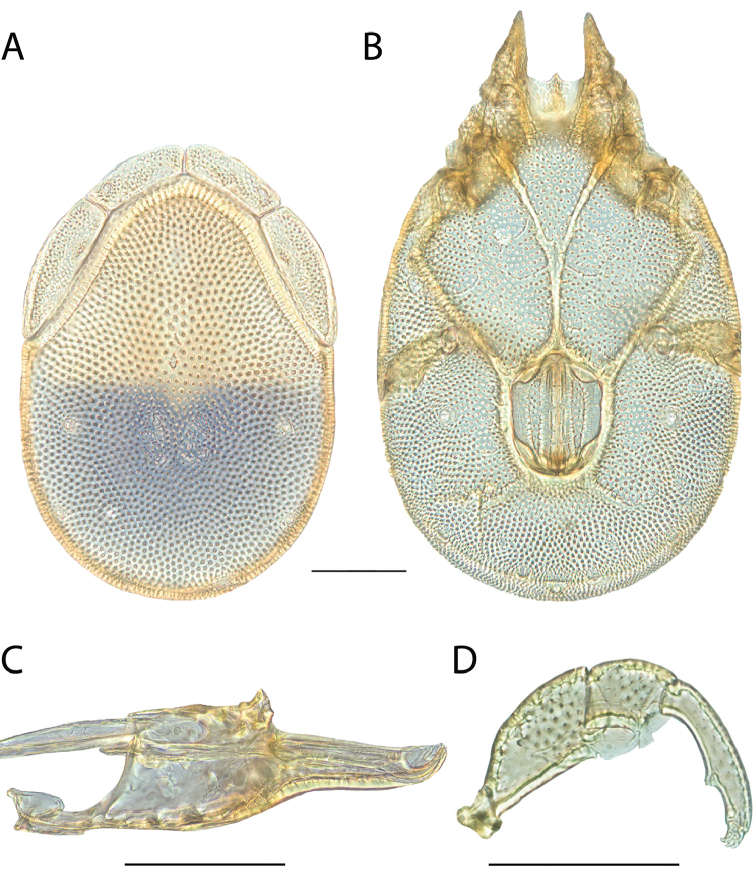
*Torrenticola flangipalpa* sp. n. male: **A** dorsal plates **B** venter (legs removed) **C** subcapitulum **D** pedipalp (setae not accurately depicted). Scale = 100 µm.

######## Remarks.

*Torrenticola flangipalpa* groups with other members of the Raptor Complex with high support in all analyses and specimens are less than 1% different in COI sequence from each other. In all analyses, *T. flangipalpa* groups with two other morphologically similar species: *T. nigroalba* and *T. solisorta*. These three species are greater than 12% different from each other in COI sequence.

That clade of three species corresponds to an identification group, Nigroalba Group, the members of which are easily differentiated by their size, coloration, long medial suture in females, and overall appearance.

This species hypothesis is supported by low COI divergence within the species (0–2%) and high divergence between species (3–15%), and by the morphological characters outlined in the diagnosis.

####### Torrenticola folkertsae

Taxon classificationAnimaliaTrombidiformesTorrenticolidae

Fisher & Dowling sp. n.

http://zoobank.org/9AB5AC1A-6839-4690-8E1B-0945B5942351

######## Material examined.

HOLOTYPE (♀): from USA, New Hampshire, Coos County, picnic area beside Rt. 110 ca. 1 km east of Stark, (44°36'36"N, 71°24'24"W), 5 July 1989, by IM Smith, IMS890071

PARATYPES (4 ♀; 4 ♂): 1 ♂ (ALLOTYPE) from Coos County, New Hampshire picnic area beside Rt. 110 ca. 1 km east of Stark, (44°36'36"N, 71°24'24"W), 5 July 1989, by IM Smith, IMS890071 • 4 ♀ and 4 ♂ from Coos County, picnic area beside Rt. 110 ca. 1 km east of Stark, (44°36'36"N, 71°24'24"W), 5 July 1989, by IM Smith, IMS890071.

######## Type deposition.

Holotype (♀), allotype (♂), and some paratypes (2 ♀; 2 ♂) deposited in the CNC; other paratypes (2 ♀; 1 ♂) deposited in the ACUA.

######## Diagnosis.

*Torrenticola folkertsae* are similar to other members of the Partial 2-Plate Group (*T. magnexa*, *T. priapus*, and *T. pulchra*) in having anterio-lateral platelets partially fused to the dorsal plate and being distributed in the east. *T. folkertsae* can be differentiated from *T. magnexa* and *T. pulchra* by having more elongate pedipalpal tibiae (length/width = 4.05–4.83 in *T. folkertsae*, 2.61–4.00 in others) and by dorsal coloration and pattern. *T. folkertsae* can be differentiated from *T. priapus* by having more elongate pedipalpal tibiae (length/width ♀ = 4.5–4.83 in *T. folkertsae*, 3.9–4.22 in *T. priapus* ♂ = 4.05–4.33 in *T. folkertsae*, 3.5–3.78 in *T. priapus*) and a less elongate rostrum (length/width = 2.55–3.00 in *T. folkertsae*, 3.17–3.39 in *T. priapus*).

######## Description.

**Female (Figure 82)** (n = 5) (holotype measurements in parentheses when available) with characters of the genus with following specifications.

**Dorsum** — (600–720 (720) long; 505–600 (580) wide) ovoid with faint reddish-purple medially. Anterio-medial platelets (140–167.5 (156.25) long; 57.5–70 (65) wide). Anterio-lateral platelets (180–205 (185) long; 85–95 (85) wide) partially fused to dorsal plate. Dgl-4 closer to the edge of the dorsum than to the muscle scars (distance between Dgl-4 340–380 (380)). Dorsal plate proportions: dorsum length/width 1.11–1.24 (1.24); dorsal width/distance between Dgl-4 1.40–1.75 (1.53); anterio-medial platelet length/width 2.32–2.50 (2.40); anterio-lateral platelet length/width 2.12–2.31 (2.18); anterio-lateral/anterio-medial length 1.12–1.41 (1.18).

**Gnathosoma — Subcapitulum** (310–345 (345) long (ventral); 240–270 (270) long (dorsal); 145–162.5 (157.5) tall) colorless. Rostrum (135–150 (140) long; 50–55 (55) wide). Chelicerae (320–415 (415) long) with curved fangs (65–70 (70) long). Subcapitular proportions: ventral length/height 2.07–2.19 (2.19); rostrum length/width 2.55–2.86 (2.55). **Pedipalps** with tuberculate ventral extensions on femora and genua. Palpomeres: trochanter (48.75–52.5 (52.5) long); femur (130–145 (145) long); genu (77.5–87.5 (87.5) long); tibia (108.75–120 (118.75) long; 22.5–26.25 (26.25) wide); tarsus (17.5–22.5 (22.5) long). Palpomere proportions: femur/genu 1.65–1.68 (1.66); tibia/femur 0.82–0.87 (0.82); tibia length/width 4.50–4.83 (4.52).

**Venter** — (680–880 (860) long; 560–675 (650) wide) colorless. Gnathosomal bay (167.5–185 (182.5) long; 80–95 (95) wide). Cxgl-4 far from apex. **Medial suture** (15–20 (20) long). **Genital plates** (175–197.5 (197.5) long; 167.5–175 (167.5) wide). Additional measurements: Cx-1 (300–340 (330) long (total); 127.5–157.5 (145) long (medial)); Cx-3 (380–400 (400) wide); anterior venter (155–180 (180) long). Ventral proportions: gnathosomal bay length/width 1.92–2.13 (1.92); anterior venter/genital field length 0.79–1.00 (0.91); anterior venter length/genital field width 0.91–1.07 (1.07); anterior venter/medial suture 8.38–12.00 (9.00).

**Male (Figure 83)** (n = 5) (allotypic measurements in parentheses when available) with characters of the genus with following specifications.

**Dorsum** — (535–580 (570) long; 420–450 (445) wide) ovoid with faint reddish-purple medially. Anterio-medial platelets (125–135 (135) long; 55–65 (55) wide). Anterio-lateral platelets (180–187.5 (180) long; 62.5–77.5 (77.5) wide) partially fused to dorsal plate. Dgl-4 closer to the edge of the dorsum than to the muscle scars (distance between Dgl-4 280–310 (310)). Dorsal plate proportions: dorsum length/width 1.27–1.29 (1.28); dorsal width/distance between Dgl-4 1.44–1.54 (1.44); anterio-medial platelet length/width 2.04–2.45 (2.45); anterio-lateral platelet length/width 2.32–2.88 (2.32); anterio-lateral/anterio-medial length 1.33–1.48 (1.33).

**Gnathosoma — Subcapitulum** (265–292.5 (292.5) long (ventral); 210–227.5 (227.5) long (dorsal); 110–115 (110) tall) colorless. Rostrum (110–120 (120) long; 40–42.5 (40) wide). Chelicerae (275–295 (295) long) with curved fangs (55–57.5 (55) long). Subcapitular proportions: ventral length/height 2.36–2.66 (2.66); rostrum length/width 2.75–3.00 (3.00). **Pedipalps** with tuberculate ventral extensions on femora and genua. Palpomeres: trochanter (42.5–46.25 (45) long); femur (110–116.25 (116.25) long); genu (67.5–72.5 (72.5) long); tibia (97.5–102.5 (102.5) long; 22.5–25 (23.75) wide); tarsus (17.5–20 (17.5) long). Palpomere proportions: femur/genu 1.55–1.67 (1.60); tibia/femur 0.88–0.90 (0.88); tibia length/width 4.05–4.33 (4.32).

**Venter** — (640–685 (680) long; 470–570 (510) wide) colorless. Gnathosomal bay (132.5–150 (142.5) long; 70–80 (75) wide). Cxgl-4 subapical. **Medial suture** (105–125 (115) long). **Genital plates** (130–145 (145) long; 125–130 (130) wide). Additional measurements: Cx-1 (255–295 (295) long (total); 130–150 (150) long (medial)); Cx-3 (330–365 (340) wide); anterior venter (250–275 (267.5) long). Ventral proportions: gnathosomal bay length/width 1.81–2.00 (1.90); anterior venter/genital field length 1.79–1.96 (1.84); anterior venter length/genital field width 2.00–2.16 (2.06); anterior venter/medial suture 2.17–2.45 (2.33).

**Immatures** unknown.

######## Etymology.

Specific epithet (*folkertsae*) named in honor of Debbie Folkerts, professor of biology at Auburn University, who, together with her late husband George Folkerts, were instrumental to JRF in channeling his passion for natural history and teaching into a career path.

######## Distribution.

**Figure 81.**
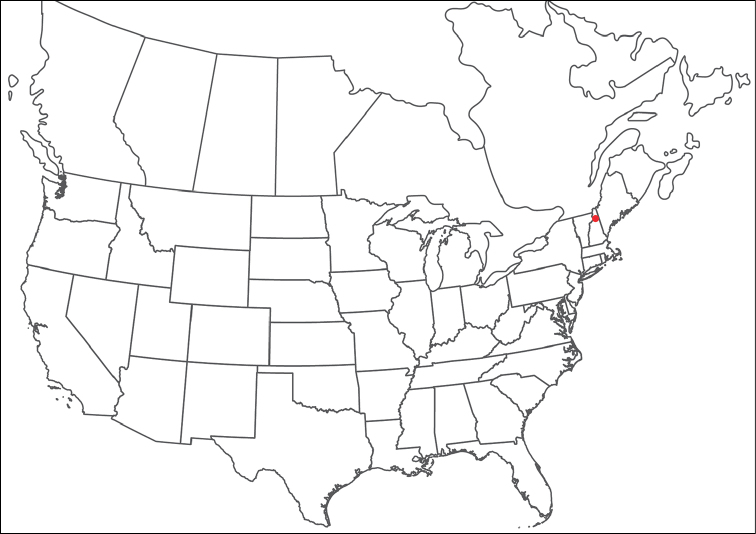
*Torrenticola folkertsae* sp. n. distribution.

New Hampshire (Figure 81).

**Figure 82.**
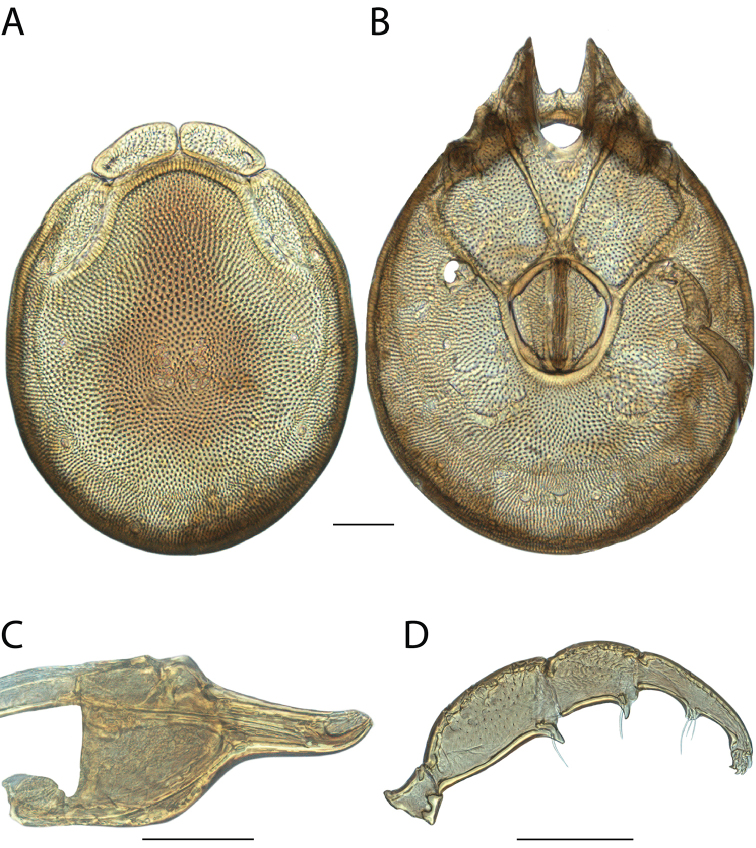
*Torrenticola folkertsae* sp. n. female: **A** dorsal plates **B** venter (legs removed) **C** subcapitulum **D** pedipalp (setae not accurately depicted). Scale = 100 µm.

**Figure 83.**
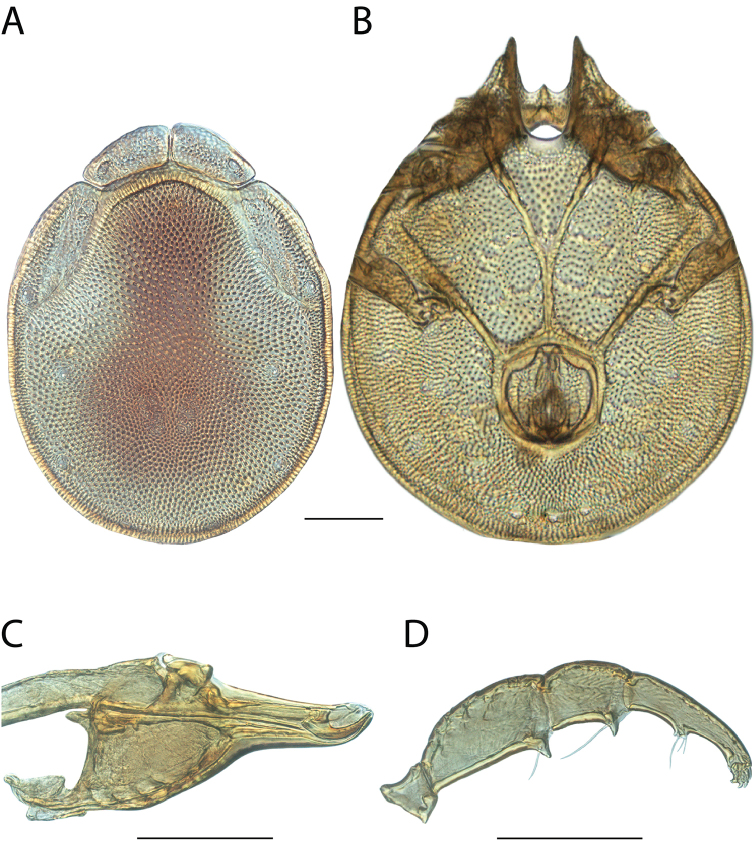
*Torrenticola folkertsae* sp. n. male: **A** dorsal plates **B** venter (legs removed) **C** subcapitulum **D** pedipalp (setae not accurately depicted). Scale = 100 µm.

######## Remarks.

Unfortunately, we were unable to acquire fresh material of *Torrenticola folkertsae* and therefore this species is not included in our phylogenetic analyses. However, we were able to examine morphology with material preserved in GAW. The overall similarity, distribution, and partial fusion of the dorso-lateral platelets to the dorsal plate, are consistent with placing this species in the Rusetria Complex and the Partial 2-Plate Identification Group.

####### Torrenticola glomerabilis

Taxon classificationAnimaliaTrombidiformesTorrenticolidae

Fisher & Dowling sp. n.

http://zoobank.org/CF3075B3-56EC-4A3C-95E5-C0DEF4C6AD8A

######## Material examined.

HOLOTYPE (♀): from USA, Tennessee, Sevier County, Great Smokey Mountains National Park, Sugarlands Nature Trail (35°40'47"N, 83°31'51"W), 10 Sep 2010, by IM Smith, IMS100125.

PARATYPES (23 ♀; 25 ♂): **Georgia, USA**: 2 ♀ and 2 ♂ from Floyd County, beside road from Everett Spring to Villanow, 1.4 kilometers south of The Pocket Campground, tributary of Johns Creek, 4 Jul 1990, by IM Smith, IMS900077 • **Kentucky, USA**: 2 ♀ and 3 ♂ from Bell County, Middlesboro, near north boundary of Cumberland Gap National Historical Park, Sugar Run, 9 Jul 1990, by IM Smith, IMS900084 • 2 ♀ and 1 ♂ from Bell County, Pineville, Pine Mountain State Resort Park, Laurel Cove, Lower shelter Picnic Area, 9 Jul 1990, by IM Smith, IMS900083 • **Pennsylvania, USA**: 1 ♀ from Fayette County, Dunbar Creek (39°57'50"N, 79°35'8.70"W), 10 Aug 2014, by MJ Skvarla, MS 14-0810-001 • 1 ♀ from Huntingdon County, Alan Seeger Natural Area, beside road from McAlevys Fort to Route 322, Stone Creek, 19 Jul 1990, by IM Smith, IMS900107 • **Tennessee, USA**: 1 ♂ (ALLOTYPE) from Sevier County, Great Smokey Mountains National Park, Sugarlands Nature Trail (35°40'47"N, 83°31'51"W), 10 Sep 2010, by IM Smith, IMS100125 • 1 ♂ from Sevier County, Great Smokey Mountains National Park, Laurel Creek (35°39'7"N, 83°42'32"W), 17 Sep 2010, by IM Smith, IMS100145 • 4 ♀ and 5 ♂ from Sevier County, Great Smokey Mountains National Park, Sugarlands Nature Trail (35°40'47"N, 83°31'51"W), 10 Sep 2010, by IM Smith, IMS100125 • **Virginia, USA**: 1 ♀ and 1 ♂ from Alleghany County, Clifton Forge, beside Route 606, 1.2 kilometers southeast of Forest Route 125, Smith Creek, 13 Jul 1990, by IM Smith, IMS900093 • 1 ♀ and 1 ♂ from Alleghany County, Longdale Furnace, beside Forest Route 108, 1.7 kilometers west of Route 850, Simpson Creek, 14 Jul 1990, by IM Smith, IMS900094 • 1 ♀ and 1 ♂ from Amherst County, Upper Otter Creek Overlook beside Blue Ridge, Otter Creek (37°36'57"N, 79°19'27"W), 7 Sep 2007, by IM Smith, IMS070056A • 2 ♀ and 2 ♂ from Augusta County, beside Forest Road 42, 15.9 kilometers east of Vesuvius, Coles Run, 26 Jul 1990, by IM Smith, IMS900060 • 2 ♂ from Bath County, beside Forest Route 1744 at Route 39, between Warm Springs and Mountain Grove, O’Roarke Draw, 15 Jul 1990, by IM Smith, IMS900098 • 1 ♀ and 2 ♂ from Bath County, beside Forest Route 364, off Route 39 east of Warm Springs, Panther Run, 15 Jul 1990, by IM Smith, IMS900099 • 1 ♀ and 1 ♂ from Giles County, Mechanicsburg, beside Dismal Creek Road, Standrock Brook (37°11'38"N, 80°53'26"W), 9 Sep 2005, by IM Smith, IMS050066 • 2 ♀ and 1 ♂ from Giles County, Mechanicsburg, beside Dismal Creek Road, Standrock Brook (37°11'38"N, 80°53'26"W), 11 Jul 1990, IMS900088 • 3 ♂ from Montgomery County, Blacksburg, beside Route 621 at Caldwell Fields Campground, Craig Creek (37°20'N, 80°20'W), 12 Jul 1990, by IM Smith, IMS900089A • 1 ♀ and 1 ♂ from Page County, beside Route 730, 0.2 kilometers west of Route 675, Passage Creek, 25 Jun 1990, by IM Smith, IMS900059 • 1 ♀ from Rock Bridge County, Vesuvius, beside Route 56, 2.2 kilometers west of Blue Ridge Parkway, Little Marys Creek, 26 Jun 1990, by IM Smith, IMS900062.

######## Type deposition.

Holotype (♀), allotype (♂), and most paratypes (18 ♀; 19 ♂) deposited in the CNC; other paratypes (5 ♀; 5 ♂) deposited in the ACUA.

######## Diagnosis.

*Torrenticola glomerabilis* are similar to other members of the Rusetria “4-Plate” group (*T. dunni*, *T. glomerabilis*, *T. kittatinniana*, *T. pollani*, *T. rufoalba* and *T. shubini*) and *T. skvarlai* in having anterio-lateral platelets free from the dorsal plate, dorsal coloration separated into anterior and posterior portions, and indistinct hind coxal margins. *T. glomerabilis* can be differentiated from *T. dunni*, *T. shubini*, *T. kittatinniana*, by having Dgl-4 closer to the dorsal edge (dorsal width/distance between Dgl-4 = 1.53–1.66 in *T. glomerabilis*, 1.20–1.42 in others) and stockier tibiae (length/width ♀ = 4.11–4.50 in *T. glomerabilis*, 3.27–3.60 in others; ♂ = 3.55–4.38 in *T. glomerabilis*, 2.80–3.45 in others). *T. glomerabilis* can be differentiated from *T. pollani* and *T. rufoalba* by having stockier anterio-medial platelets (length/width ♀ = 1.9–2.3 in *T. glomerabilis*, 2.5–3.0 in others; ♂ = 1.9–2.2 in *T. glomerabilis*, 2.3–2.9 in others) and wider dorsum (♀ = 460–490 in *T. glomerabilis*, 400–420 in others; ♂ = 395–430 in *T. glomerabilis*, 310–340 in others). *T. glomerabilis* can be differentiated from *T. skvarlai* by having a conical pedipalpal femoral tubercle, whereas *T. skvarlai* has a broad and flat pedipalpal femoral tubercle, and by having a longer anterior venter (♀ = 202–213 in *T. glomerabilis*, 140–153 in *T. skvarlai*; ♂ = 240–280 in *T. glomerabilis*, 177–205 in *T. skvarlai*).

######## Description.

**Female (Figure 85)** (n = 5) (holotype measurements in parentheses when available) with characters of the genus with following specifications.

**Dorsum** — (580–615 (605) long; 460–490 (475) wide) circular with bold bluish-purple or reddish-purple coloration separated into anterior and posterior portions. Anterio-medial platelets (125–132.5 (132.5) long; 55–65 (60) wide). Anterio-lateral platelets (172.5–195 (180) long; 70–82.5 (82.5) wide) free from dorsal plate. Dgl-4 approaching midway between muscle scars and dorsum edge (distance between Dgl-4 280–310 (310)). Dorsal plate proportions: dorsum length/width 1.23–1.32 (1.27); dorsal width/distance between Dgl-4 1.53–1.66 (1.53); anterio-medial platelet length/width 1.96–2.27 (2.21); anterio-lateral platelet length/width 2.17–2.48 (2.18); anterio-lateral/anterio-medial length 1.36–1.53 (1.36).

**Gnathosoma — Subcapitulum** (320–330 (320) long (ventral); 223–243 (223) long (dorsal); 112.5–120 (117.5) tall) colorless. Rostrum (132.5–137.5 (132.5) long; 40–47.5 (40) wide). Chelicerae (320–330 (321) long) with curved fangs (50–55 (53) long). Subcapitular proportions: ventral length/height 2.72–2.89 (2.72); rostrum length/width 2.89–3.34 (3.31). **Pedipalps** with tuberculate ventral extensions on femora and genua. Palpomeres: trochanter (37.5–42.5 (40) long); femur (112.5–117.5 (116.25) long); genu (67.5–70 (67.5) long); tibia (88.75–95 (88.75) long; 20–22.5 (20) wide); tarsus (17.5–17.5 (17.5) long). Palpomere proportions: femur/genu 1.67–1.74 (1.72); tibia/femur 0.76–0.83 (0.76); tibia length/width 4.11–4.50 (4.44).

**Venter** — (710–730 (730) long; 512–550 (513) wide) bold bluish-purple or reddish-purple coloration. Gnathosomal bay (130–155 (155) long; 72.5–95 (72.5) wide). Cxgl-4 subapical. **Medial suture** (22.5–50 (27.5) long). **Genital plates** (167.5–177.5 (175) long; 150–157.5 (157.5) wide). Additional measurements: Cx-1 (276–305 (291) long (total); 122–160 (149) long (medial)); Cx-3 (320–370 (321) wide); anterior venter (202.5–212.5 (202.5) long). Ventral proportions: gnathosomal bay length/width 1.37–2.14 (2.14); anterior venter/genital field length 1.14–1.24 (1.16); anterior venter length/genital field width 1.29–1.40 (1.29); anterior venter/medial suture 4.15–9.00 (7.36).

**Male (Figure 86)** (n = 6) (allotypic measurements in parentheses when available) with characters of the genus with following specifications.

**Dorsum**— (495–575 (530) long; 395–430 (420) wide) circular with bold bluish-purple or reddish-purple coloration separated into anterior and posterior portions. Anterio-medial platelets (112.5–120 (120) long; 52.5–60 (55) wide). Anterio-lateral platelets (165–187.5 (167.5) long; 60–67.5 (60) wide) free from dorsal plate. Dgl-4 approaching midway between muscle scars and dorsum edge (distance between Dgl-4 235–280 (275)). Dorsal plate proportions: dorsum length/width 1.25–1.34 (1.26); dorsal width/distance between Dgl-4 1.50–1.68 (1.53); anterio-medial platelet length/width 1.96–2.18 (2.18); anterio-lateral platelet length/width 2.54–2.88 (2.79); anterio-lateral/anterio-medial length 1.40–1.67 (1.40).

**Gnathosoma — Subcapitulum** (260–297.5 (290) long (ventral); 188–225 (212.5) long (dorsal); 93.75–103.75 (93.75) tall) colorless. Rostrum (105–120 (120) long; 35–40 (35) wide). Chelicerae (249–298 (285) long) with curved fangs (40–50 (50) long). Subcapitular proportions: ventral length/height 2.77–3.09 (3.09); rostrum length/width 2.80–3.43 (3.43). **Pedipalps** with tuberculate ventral extensions on femora and genua. Palpomeres: trochanter (35–35 (35) long); femur (95–105 (102.5) long); genu (57.5–65 (60) long); tibia (80–88.75 (82.5) long; 20–25 (20) wide); tarsus (15–17.5 (15) long). Palpomere proportions: femur/genu 1.58–1.71 (1.71); tibia/femur 0.80–0.87 (0.80); tibia length/width 3.55–4.38 (4.13).

**Venter** — (600–690 (670) long; 443–540 (460) wide) bold bluish-purple or reddish-purple coloration. Gnathosomal bay (107.5–135 (132.5) long; 70–80 (70) wide). Cxgl-4 subapical. **Medial suture** (85–107.5 (92.5) long). **Genital plates** (135–147.5 (140) long; 110–120 (117.5) wide). Additional measurements: Cx-1 (224–280 (280) long (total); 88–160 (150) long (medial)); Cx-3 (292–342 (300) wide); anterior venter (240–280 (260) long). Ventral proportions: gnathosomal bay length/width 1.48–1.93 (1.89); anterior venter/genital field length 1.78–1.90 (1.86); anterior venter length/genital field width 2.13–2.41 (2.21); anterior venter/medial suture 2.47–2.87 (2.81).

**Immatures** unknown.

######## Etymology.

Specific epithet (*glomerabilis*) refers to the rounded body of this species compared to all other members of the Rusetria Complex (*glomerabilis*, L. round).

######## Distribution.

**Figure 84.**
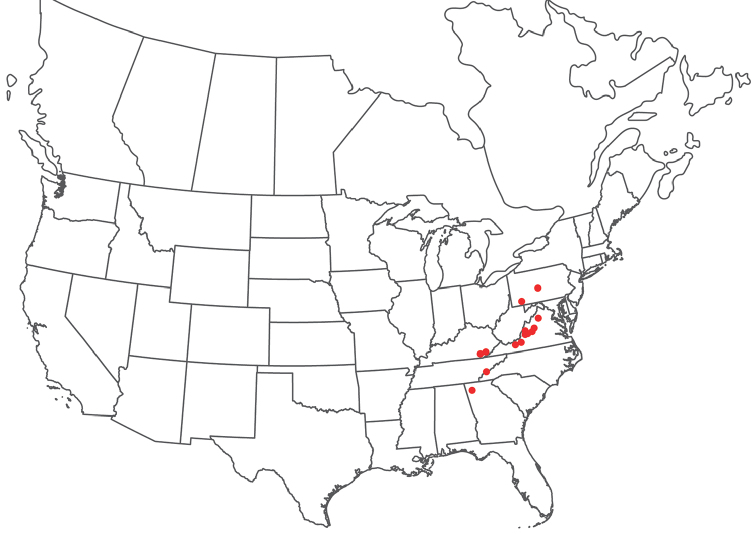
*Torrenticola glomerabilis* sp. n. distribution.

Appalachians (Figure 84).

**Figure 85.**
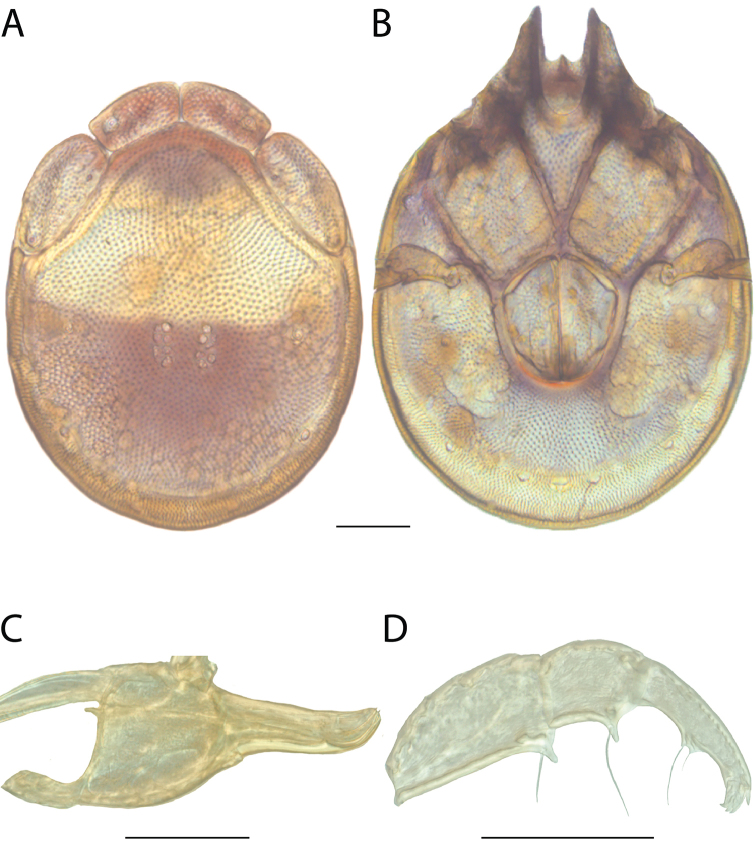
*Torrenticola glomerabilis* sp. n. female: **A** dorsal plates **B** venter (legs removed) **C** subcapitulum **D** pedipalp (setae not accurately depicted). Scale = 100 µm.

**Figure 86.**
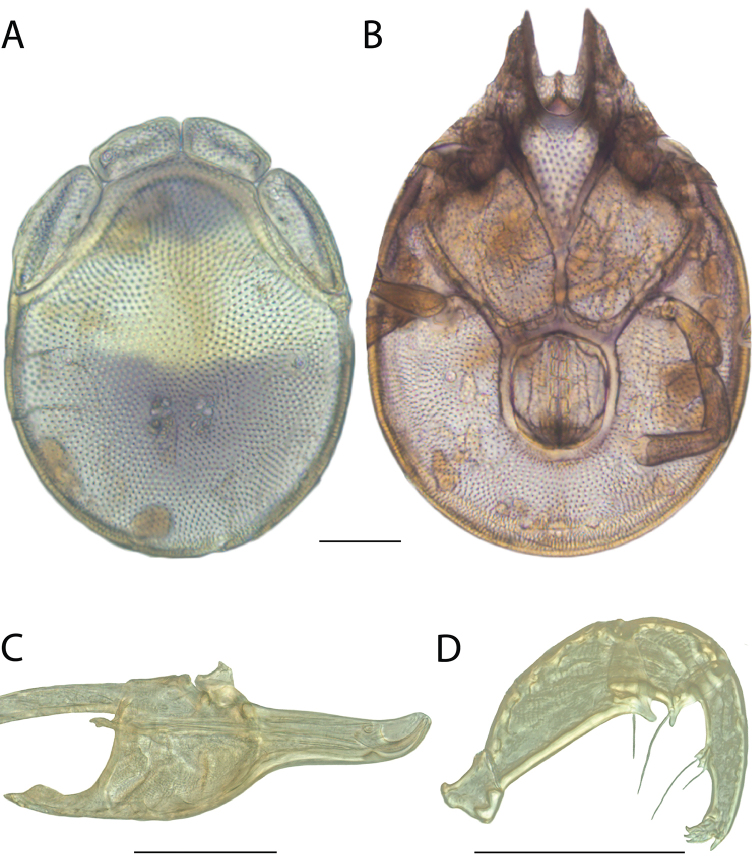
*Torrenticola glomerabilis* sp. n. male: **A** dorsal plates **B** venter (legs removed) **C** subcapitulum **D** pedipalp (setae not accurately depicted). Scale = 100 µm.

######## Remarks.

*Torrenticola glomerabilis* groups with other members of the Rusetria Complex with high support and specimens are less than 1% different in COI sequence from each other. In all analyses, *T. glomerabilis* groups with two other species, *T. delicatexa* and *T. ululata*, which are 10–13% different from each other. Of these species, *T. glomerabilis* is the only one with the lateral platelets free from the dorsal plate. Because of this, we place *T. glomerabilis* within the Eastern 4-Plate Identification Group, which can be differentiated by having rounder bodies than any other species in the Rusetria Complex.

*Torrenticola glomerabilis* occupy an interesting position phylogenetically by being nested between the Eastern 2-Plate and Eastern 4-Plate Identification Groups (Figure 6, 12). Their unique shape and interesting phylogenetic affinity flag this species as important to future studies on the evolution of eastern members of the Rusetria Complex.

This species hypothesis is supported by biogeography, low COI divergence within the species (0–2%) and high divergence between species (3–15%), and the morphological characters outlined in the diagnosis.

####### Torrenticola gnoma

Taxon classificationAnimaliaTrombidiformesTorrenticolidae

Fisher & Dowling sp. n.

http://zoobank.org/3B101F81-1A6A-487B-8EE3-F87ED3C295C7

######## Material examined.

HOLOTYPE (♀): from USA, Mississippi, Attala County, Hurricane Creek (33°4'57"N, 89°31'29"W), 12 Sep 2008, by IM Smith, IMS080052.

PARATYPES (6 ♀; 10 ♂): **Georgia, USA**: 1 ♂ from Lowndes County, Withlacoochee River, beside Route 84 at Brooks County line, 13 Sep 1968, by DR Cook, DRC680075 • **Illinois, USA**: 1 ♀ and 2 ♂ from Clark County, Big Creek (32°25'59"N, 87°41'15"W), 30 Jul 2014, by MJ Skvarla, MS 14-0730-001 • 1 ♂ from Vermilion County, Fairmount, beside Vermilion County Road 680E, Jordan Creek (40°4'N, 87°50'W), 10 Sep 1991, by IM Smith, IMS910030 • **Mississippi, USA**: 1 ♂ (ALLOTYPE) from USA, Mississippi, Attala County, Hurricane Creek (33°4'57"N, 89°31'29"W), 12 Sep 2008, by IM Smith, IMS080052 • 1 ♀ and 1 ♂ from Attala County, Hurricane Creek (33°4'58"N, 89°31'31"W), 30 Sep 2010, by IM Smith, IMS100168 • 1 ♀ and 1 ♂ from Grenada County, Leflore, beside Black Creek Road, Black Creek (33°43'N, 90°3'W), 16 Sep 1991, by IM Smith, IMS910044 • 1 ♀ and 1 ♂ from Jefferson County, off Natchez Trace Parkway, Coles Creek (31°41'26"N, 91°10'52"W), 2 Oct 1994, by IM Smith, IMS940029A • 1 ♀ and 1 ♂ from Tishomingo County, Tishomingo State Park, Bear Creek, (34°36'N, 88°11'W), 18 Sep 1991, by IM Smith, IMS910047A • **Oklahoma, USA**: 2 ♀ and 2 ♂ from Pushmataha County, beside Route 271, South of Albion, Walnut Creek (34°39'N, 95°7'W), 1 Jul 1987, by IM Smith, IMS870063A.

######## Type deposition.

Holotype (♀), allotype (♂), and some paratypes (4 ♀; 5 ♂) deposited in the CNC; other paratypes (2 ♀; 3 ♂) deposited in ACUA.

######## Diagnosis.

*Torrenticola gnoma* are similar to other members of the Raptor Group (*T. irapalpa*, *T. longitibia*, *T. mjolniri*, *T. elusiva*, *T. racupalpa*, *T. raptor*, *T. danielleae*, *T. daemon*, and *T. ivyae*) in having round bodies; Dgl-4 close to muscles scars; long, thin subcapitular rostra; and long, thin pedipalp tibiae. *T. gnoma* can be differentiated from *T. elusiva* by being smaller (dorsum length = 540–595 in *T. gnoma*, 645 in *T. elusiva*); having a stockier rostrum (length/width = 2.74–3.13 in *T. gnoma*, 3.65 in *T. elusiva*); and by dorsal coloration. *T. gnoma* can be differentiated from *T. racupalpa* by having a stockier rostrum (length/width = 2.74–3.13 in *T. gnoma*, 3.56–3.88 in *T. racupalpa*) and by dorsal coloration and pattern. *T. gnoma* can be differentiated from *T. irapalpa*, *T. danielleae*, and *T. daemon* by dorsal coloration and pattern. Additionally, female *T. gnoma* can be differentiated from female *T. irapalpa*, *T. danielleae*, and *T. daemon* by having Dgl-4 closer to the muscle scars (dorsal width/distance between Dgl-4 ♀ = 2.65–3.29 in *T. gnoma*, 1.57–2.09 in others). *T. gnoma* can be differentiated from *T. mjolniri*, *T. longitibia*, *T. raptor*, and *T. ivyae* by having stockier pedipalp tibiae (length/width = 3.88–4.67 in *T. gnoma*, 4.75–7.54 in others) and a stockier rostrum (length/width = 2.56–3.23 in *T. gnoma*, 3.44–4.4 in others).

######## Description.

**Female (Figure 88)** (n = 5) (holotype measurements in parentheses when available) with characters of the genus with following specifications.

**Dorsum** — (540–595 (550) long; 440–500 (455) wide) circular with a large spot of coloration medially extending in a thin strip anteriorly to the edge of the dorsal plate, coloration variable from navy blue to purple to pink. Anterio-medial platelets (122.5–137.5 (131.25) long; 55–62.5 (60) wide). Anterio-lateral platelets (152.5–187.5 (167.5) long; 67.5–75 (70) wide) free from dorsal plate. Dgl-4 much closer to the muscle scar than to dorsum edge (distance between Dgl-4 140–185 (155)). Dorsal plate proportions: dorsum length/width 1.17–1.30 (1.21); dorsal width/distance between Dgl-4 2.65–3.29 (2.94); anterio-medial platelet length/width 2.06–2.39 (2.19); anterio-lateral platelet length/width 2.26–2.59 (2.39); anterio-lateral/anterio-medial length 1.11–1.46 (1.28).

**Gnathosoma — Subcapitulum** (285–305 (290) long (ventral); 225–239 (226) long (dorsal); 115–135 (120) tall) colorless. Rostrum (122.5–130 (125) long; 40–47.5 (40) wide) elongate. Chelicerae (285–310 (286) long) with curved fangs (53–60 (54) long). Subcapitular proportions: ventral length/height 2.26–2.52 (2.42); rostrum length/width 2.74–3.13 (3.13). **Pedipalps** elongate with tuberculate ventral extensions on femora and genua. Palpomeres: trochanter (40–45 (40) long); femur (110–120 (112.5) long); genu (60–67.5 (62.5) long); tibia (87.5–105 (92.5) long; 20–22.5 (20) wide); tarsus (17.5–20 (17.5) long). Palpomere proportions: femur/genu 1.63–1.88 (1.80); tibia/femur 0.76–0.88 (0.82); tibia length/width 4.17–4.67 (4.63).

**Venter** — (660–730 (680) long; 500–575 (500) wide) colorless. Gnathosomal bay (142.5–172.5 (152.5) long; 75–92.5 (75) wide). Cxgl-4 subapical. **Medial suture** (15–27.5 (27.5) long). **Genital plates** (152.5–165 (157.5) long; 142.5–152.5 (142.5) wide). Additional measurements: Cx-1 (252–285 (257) long (total); 84–122 (108) long (medial)); Cx-3 (317–377 (318) wide); anterior venter (160–167.5 (165) long). Ventral proportions: gnathosomal bay length/width 1.57–2.16 (2.03); anterior venter/genital field length 0.97–1.10 (1.05); anterior venter length/genital field width 1.05–1.16 (1.16); anterior venter/medial suture 6.00–10.67 (6.00).

**Male (Figure 89)** (n = 5) (allotypic measurements in parentheses when available) with characters of the genus with following specifications.

**Dorsum** — (420–495 (450) long; 355–375 (375) wide) circular with a large spot of coloration medially extending in a thin strip anteriorly to the edge of the dorsal plate, coloration variable from navy blue to purple to pink. Anterio-medial platelets (1.80–2.30 (2.18) long; 50–62.5 (55) wide). Anterio-lateral platelets (135–152.5 (152.5) long; 60–65 (60) wide) free from dorsal plate. Dgl-4 much closer to the muscle scar than to dorsum edge (distance between Dgl-4 130–180 (165)). Dorsal plate proportions: dorsum length/width 1.17–1.32 (1.20); dorsal width/distance between Dgl-4 2.06–2.73 (2.27); anterio-medial platelet length/width 1.80–2.30 (2.18); anterio-lateral platelet length/width 2.16–2.54 (2.54); anterio-lateral/anterio-medial length 1.17–1.36 (1.27).

**Gnathosoma— Subcapitulum** (240–265 (265) long (ventral); 175–196 (196) long (dorsal); 97.5–105 (105) tall) colorless. Rostrum (98.75–107.5 (107.5) long; 35–40 (35) wide) elongate. Chelicerae (225–257 (256) long) with curved fangs (41–51 (50) long). Subcapitular proportions: ventral length/height 2.29–2.56 (2.52); rostrum length/width 2.56–3.07 (3.07). **Pedipalps** elongate with tuberculate ventral extensions on femora and genua. Palpomeres: trochanter (35–47.5 (47.5) long); femur (96.25–103.75 (103.75) long); genu (52.5–57.5 (57.5) long); tibia (77.5–90 (88.75) long; 20–22.5 (20) wide); tarsus (17.5–20 (18.75) long). Palpomere proportions: femur/genu 1.72–1.83 (1.80); tibia/femur 0.81–0.91 (0.86); tibia length/width 3.88–4.44 (4.44).

**Venter** — (560–590 (581) long; 354–440 (395) wide) colorless. Gnathosomal bay (105–130 (127.5) long; 62.5–77.5 (62.5) wide). Cxgl-4 subapical. **Medial suture** (67.5–80 (70) long). **Genital plates** (122.5–135 (127.5) long; 100–110 (105) wide). Additional measurements: Cx-1 (217–269 (255) long (total); 90–119 (115) long (medial)); Cx-3 (264–312 (295) wide); anterior venter (197.5–222.5 (207.5) long). Ventral proportions: gnathosomal bay length/width 1.40–2.04 (2.04); anterior venter/genital field length 1.49–1.65 (1.63); anterior venter length/genital field width 1.80–2.12 (1.98); anterior venter/medial suture 2.53–2.96 (2.96).

**Immatures** unknown.

######## Etymology.

Specific epithet (*gnoma*) refers to the dorsal pattern, which, although variable, resembles the head and cap of a gnome (*gnoma*, L. diminutive fabled being, dwarf).

######## Distribution.

**Figure 87.**
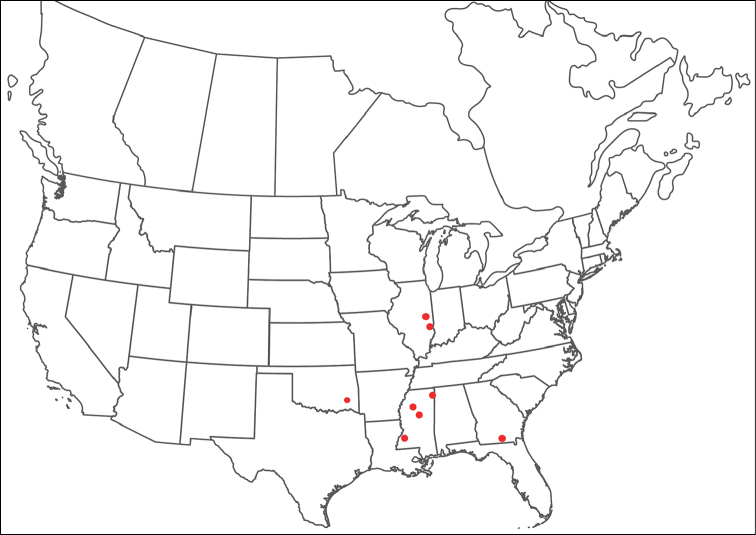
*Torrenticola gnoma* sp. n. distribution.

Eastern, but apparently absent from Appalachians and Northeast (Figure 87).

**Figure 88.**
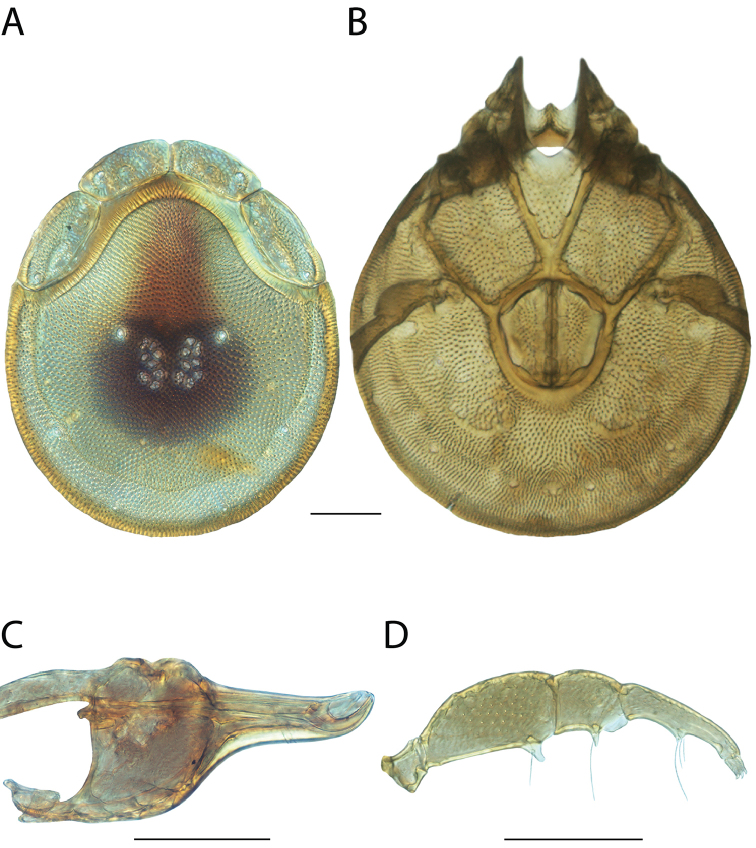
*Torrenticola gnoma* sp. n. female: **A** dorsal plates **B** venter (legs removed) **C** subcapitulum **D** pedipalp (setae not accurately depicted). Scale = 100 µm.

**Figure 89.**
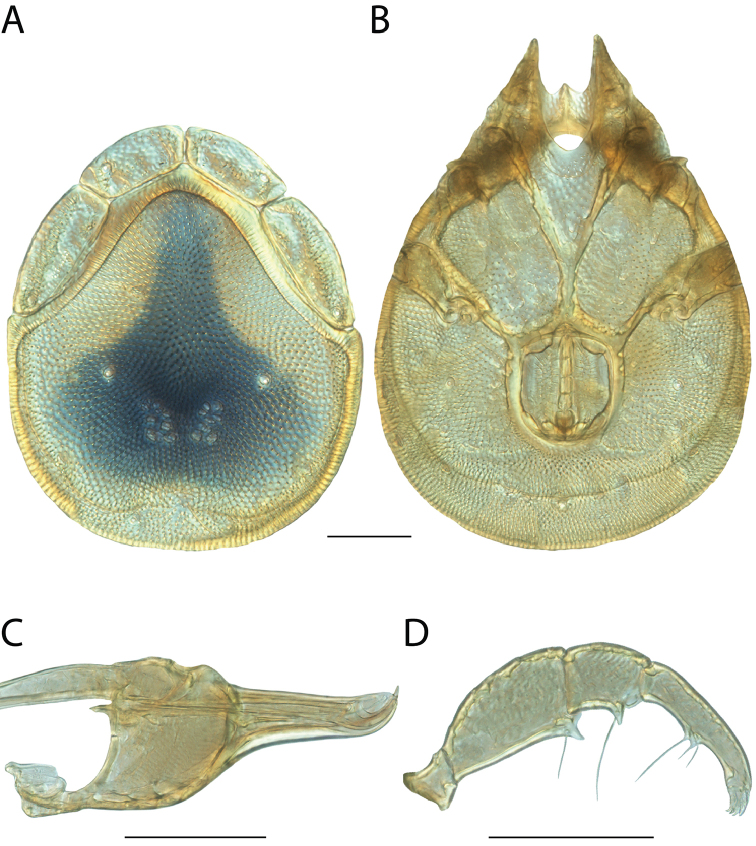
*Torrenticola gnoma* sp. n. male: **A** dorsal plates **B** venter (legs removed) **C** subcapitulum **D** pedipalp (setae not accurately depicted). Scale = 100 µm.

######## Remarks.

*Torrenticola gnoma* groups with other members of the Raptor Complex with high support and specimens of this species were less than 1% different in COI sequence from each other. In the combined analysis, *T. gnoma* groups with *T. irapalpa* with high support, but the position of this clade was not recovered. These species are greater than 9% from each other. Based upon overall similarity, distribution, and phylogenetic position, this species is placed within the Raptor Identification Group.

This species hypothesis is supported by low COI divergence within the species (0–2%) and high divergence between species (3–15%), and by the morphological characters outlined in the diagnosis.

####### Torrenticola gorti

Taxon classificationAnimaliaTrombidiformesTorrenticolidae

Fisher & Dowling sp. n.

http://zoobank.org/68090171-7471-45F7-92EC-98FEFA289568

######## Material examined.

HOLOTYPE (♀): from Canada, New Brunswick, York County, Davis Brook, beside Hwy 3, 3.5 km south of Hwy 4 at Thomaston Corner, 11 Jun 2012, by IM Smith, IMS120017, DNA 2970.

PARATYPES (24 ♀; 22 ♂): **Alabama, USA**: 1 ♀ and 1 ♂ from Cleburne County, beside Route 431, 3.3 kilometers southeast of Calhoun, Jackson Creek (33°36'N, 85°42'W), 2 Jul 1990, by IM Smith, IMS900074 • 2 ♀ and 1 ♂ from DeKalb County, Desoto State Park, beside Trail Y, West Fork of Little River (34°29'N, 85°32'W), 26 Sep 1992, by IM Smith, IMS920053A • **Georgia, USA**: 2 ♀ and 2 ♂ from Chattooga County, Cloudland, beside Route 48, East Fork of Little River (34°31'25"N, 85°30'23"W), 28 Sep 1992, by IM Smith, IMS920056A • 4 ♀ and 1 ♂ from Floyd County, Johns Creek, beside road from Everett Springs to Villanow, south of The Pocket campground, 4 Jul 1990, by IM Smith, IMS900076 & IMS900077 • **Kentucky, USA**: 3 ♀ and 2 ♂ from McCreary County, Rock Creek, White Oak Junction, beside Forest Route 556, south of Route 1363, 8 Jul 1990, by IM Smith, IMS900082A & IMS900082B • **Maine, USA**: 2 ♀ and 3 ♂ from Aroostook County, Ashland, beside Route 11 at bridge, Aroostook River (46°38'N, 68°24'W), 4 Jul 1989, by IM Smith, IMS890067 • **New Brunswick, Canada**: 1 ♂ (ALLOTYPE) from York County, Davis Brook, beside Hwy 3, 3.5 km south of Hwy 4 at Thomaston Corner, 11 Jun 2012, by IM Smith, IMS120017, DNA 2972 • 2 ♀ and 2 ♂ from York County, Davis Brook, beside Hwy 3, 3.5 km south of Hwy 4 at Thomaston Corner, 11 Jun 2012, by IM Smith, IMS120017 • 1 ♀ and 1 ♂ from York County, Magaguadavic River, beside Highway 3 just east of Thomaston Corners, 1 Jul 1989, by IM Smith, IMS890055A • **South Carolina, USA**: 1 ♂ from Greenville County, Matthews Creek, 24 Apr 2014, by D Eargle, JRF 14-0424-001 • **Tennessee, USA**: 4 ♀ and 1 ♂ from Monroe County, Tellico River (35°19'N, 84°10'W), 5 Jun 1990, by IM Smith, IMS900079 • 2 ♂ from Monroe County, Turkey Creek, beside Forest Route 35, northeast of road from Route 165 to Miller Chapel Church, 5 Jul 1990, by IM Smith, IMS900078 • 2 ♀ and 4 ♂ from Monroe County, Tellico River (35°20'27"N, 84°11'31"W), 12 Sep 2009, by IM Smith, IMS090111 • **Virginia, USA**: 1 ♀ from Scott County, beside Route 58/421,) .9 kilometers east of Route 709, North Fork of Hoiston River (36°39'N, 82°28'W), 7 Jul 1990, by IM Smith, IMS900080.

######## Type deposition.

Holotype (♀), allotype (♂), and most paratypes (19 ♀; 16 ♂) deposited in the CNC; other paratypes (5 ♀; 5 ♂) deposited in the ACUA.

######## Diagnosis.

*Torrenticola gorti* specimens from Tellico River system in Monroe County (Tennessee) can be differentiated from all other *Torrenticola* by the distinctively dark coloration with a red spot dorsally. Other color morphs are similar to species with similar dorsal patterning, such as the Rusetria “4-Plate” group (*T. dunni*, *T. glomerabilis*, *T. kittatinniana*, *T. pollani*, *T. rufoalba* and *T. shubini*), Neoanomala Group (*T. interiorensis* and *T. neoanomala*), and *T. bondi*, *T. elongata*, *T. reduncarostra*, *T. erectirostra*, *T. robisoni*, *T. irapalpa*, *T. racupalpa*, *T. skvarlai*, and *T. arktonyx*. They can be differentiated from Rusetria 4-Plates and *T. skvarlai* by having distinct hind coxal margins. *T. gorti* can be differentiated from *T. erectirostra*, *T. robisoni*, and *T. reduncarostra* by having a straight, anteriorly-directed rostrum (upturned in others). *T. gorti* can be differentiated from *T. arktonyx* by having an unmodified dorsal plate (*T. arktonyx* has distinctive longitudinal dark markings on the anterior portion of the dorsal plate that fade posteriorly). *T. gorti* can be differentiated from *T. racupalpa* and *T. irapalpa* by being more elongate (dorsum length/width = 1.47–1.6 in *T. gorti*, 1.15–1.3 in others) and tibia/femur (0.65–0.73 in *T. gorti*, 0.77–0.91 in others). *T. gorti* can be differentiated from *T. elongata* by having a more ovoid dorsum (length/width = 1.47–1.58 in *T. gorti*, 1.7–2.1 in *T. elongata*) and larger dorsum (length ♀ = 570–600 in *T. gorti*, 540–565 in *T. elongata*; ♂ = 500–525 in *T. gorti*, 450–460 in *T. elongata*). *T. gorti* can be differentiated from the Neoanomala Group by having a more elongate dorsum (length/width ♀ = 1.47–1.58 in *T. gorti*, 1.29–1.43 in Neoanomala Group; ♂ = 1.54–1.58 in *T. gorti*, 1.34–1.50 in Neoanomala Group) and having a more elongate rostrum (length/width = 3.29–3.73 in *T. gorti*, 2.59–2.90 in Neoanomala Group). *T. gorti* can be differentiated from *T. bondi* by having a more elongate dorsum (length/width = 1.47–1.58 in *T. gorti*, 1.32–1.45 in *T. bondi*) and having a more elongate rostrum (length/width = 3.32–3.73 in *T. gorti*, 2.76–3.13 in *T. bondi*).

######## Description.

**Female (Figure 91)** (n = 5) (holotype measurements in parentheses when available) with characters of the genus with following specifications.

**Dorsum** — (570–600 (600) long; 380–390 (380) wide) ovoid and elongate with three distinct color morphs: 1) navy blue coloration separated into anterior and posterior portions that meet or nearly meet laterally, and with bold orange coloration in between, from Tellico River, Monroe County, Tennessee; 2) purple coloration separated into anterior and posterior portions; 3) purple coloration separated into anterior and posterior portions with a strip of bold orange medially. Anterio-medial platelets (117.5–137.5 (130) long; 52.5–57.5 (57.5) wide). Anterio-lateral platelets (162.5–172.5 (172.5) long; 55–60 (60) wide) free from dorsal plate. Dgl-4 approaching midway between muscle scars and dorsum edge (distance between Dgl-4 220–260 (260)). Dorsal plate proportions: dorsum length/width 1.47–1.58 (1.58); dorsal width/distance between Dgl-4 1.46–1.73 (1.46); anterio-medial platelet length/width 2.24–2.45 (2.26); anterio-lateral platelet length/width 2.75–3.09 (2.88); anterio-lateral/anterio-medial length 1.20–1.45 (1.33).

**Gnathosoma — Subcapitulum** (327.5–342.5 (337.5) long (ventral); 247.75–265 (252.5) long (dorsal); 122.5–127.5 (122.5) tall) colorless. Rostrum (137.5–142.5 (142.5) long; 37.5–42.5 (40) wide) elongate. Chelicerae 330–345 (340) long) with curved fangs (56–60 (60) long). Subcapitular proportions: ventral length/height 2.59–2.76 (2.76); rostrum length/width 3.32–3.73 (3.56). **Pedipalps** with tuberculate ventral extensions on femora and genua. Palpomeres: trochanter (37.5–45 (45) long); femur (117.5–123.75 (120) long); genu (62.5–67.5 (67.5) long); tibia (77.5–82.5 (80) long; 20–25 (25) wide); tarsus (17.5–20 (20) long). Palpomere proportions: femur/genu 1.78–1.90 (1.78); tibia/femur 0.65–0.68 (0.67); tibia length/width 3.20–4.13 (3.20).

**Venter (Figure 57)** — (695–750 (750) long; 410–494 (430) wide) with three distinct color morphs: 1) navy-blue coloration; 2) colorless; 3) purple coloration. Gnathosomal bay (142.5–172.5 (172.5) long; 65–77.5 (70) wide). Cxgl-4 subapical. **Medial suture** (25–37.5 (27.5) long). **Genital plates** (160–167.5 (167.5) long; 135–140 (137.5) wide). Additional measurements: Cx-1 (285–300 (300) long (total); 107–140 (130) long (medial)); Cx-3 (290–353 (300) wide); anterior venter (187.5–195 (187.5) long). Ventral proportions: gnathosomal bay length/width 1.90–2.46 (2.46); anterior venter/genital field length 1.12–1.22 (1.12); anterior venter length/genital field width 1.36–1.44 (1.36); anterior venter/medial suture 5.20–7.80 (6.82).

**Male (Figure 92)** (n = 6) (allotypic measurements in parentheses when available) with characters of the genus with following specifications.

**Dorsum** — (500–525 (520) long; 320–340 (330) wide) ovoid and elongate with three distinct color morphs: 1) navy blue coloration separated into anterior and posterior portions that meet or nearly meet laterally, and with bold orange coloration in between; 2) purple coloration separated into anterior and posterior portions; 3) purple coloration separated into anterior and posterior portions with a strip of bold orange medially. Anterio-medial platelets (108.75–117.5 (115) long; 47.5–57.5 (50) wide). Anterio-lateral platelets (156.25–165 (157.5) long; 52.5–57.5 (53.75) wide) free from dorsal plate. Dgl-4 approaching midway between muscle scars and dorsum edge (distance between Dgl-4 210–230 (230)). Dorsal plate proportions: dorsum length/width 1.54–1.58 (1.58); dorsal width/distance between Dgl-4 1.43–1.58 (1.43); anterio-medial platelet length/width 2.04–2.35 (2.30); anterio-lateral platelet length/width 2.78–2.98 (2.93); anterio-lateral/anterio-medial length 1.36–1.44 (1.37).

**Gnathosoma — Subcapitulum** (280–292.5 (282.5) long (ventral); 211–231 (215) long (dorsal); 92.5–1.5 (95) tall) colorless. Rostrum (115–127.5 (122.5) long; 33.75–37.5 (35) wide) elongate. Chelicerae (275–302 (280) long) with curved fangs (36–50 (50) long). Subcapitular proportions: ventral length/height 2.76–3.16 (2.97); rostrum length/width 3.29–3.50 (3.50). **Pedipalps** with tuberculate ventral extensions on femora and genua. Palpomeres: trochanter (35–37.5 (35) long); femur (87.5–105 (95) long); genu (52.5–58.75 (56.25) long); tibia (62.5–72.5 (67.5) long; 18.75–22.5 (18.75) wide); tarsus (15–17.5 (15) long). Palpomere proportions: femur/genu 1.67–1.79 (1.69); tibia/femur 0.67–0.73 (0.71); tibia length/width 3.13–3.63 (3.60).

**Venter** — (630–680 (640) long; 380–438 (380) wide) with three distinct color morphs: 1) navy-blue coloration; 2) colorless; 3) purple coloration. Gnathosomal bay (122.5–142.5 (142.5) long; 60–67.5 (60) wide). Cxgl-4 subapical. **Medial suture** (62.5–87.5 (65) long). **Genital plates** (125–135 (135) long; 102.5–111.25 (102.5) wide). Additional measurements: Cx-1 (255–280 (280) long (total); 100–140 (140) long (medial)); Cx-3 (285–304 (290) wide); anterior venter (215–250 (215) long). Ventral proportions: gnathosomal bay length/width 1.89–2.38 (2.38); anterior venter/genital field length 1.59–1.89 (1.59); anterior venter length/genital field width 1.96–2.27 (2.10); anterior venter/medial suture 2.86–3.48 (3.31).

**Immatures** unknown.

######## Etymology.

Specific epithet (*gorti*) refers to Gort, the fictional giant robot of *The Day the Earth Stood Still*. In the 2008 film, Gort was depicted with a dark body and a single red eye that shot a destructive beam. This species is named for the resemblance that the distinctive specimens from Tennessee have to Gort’s red eye.

######## Distribution.

**Figure 90.**
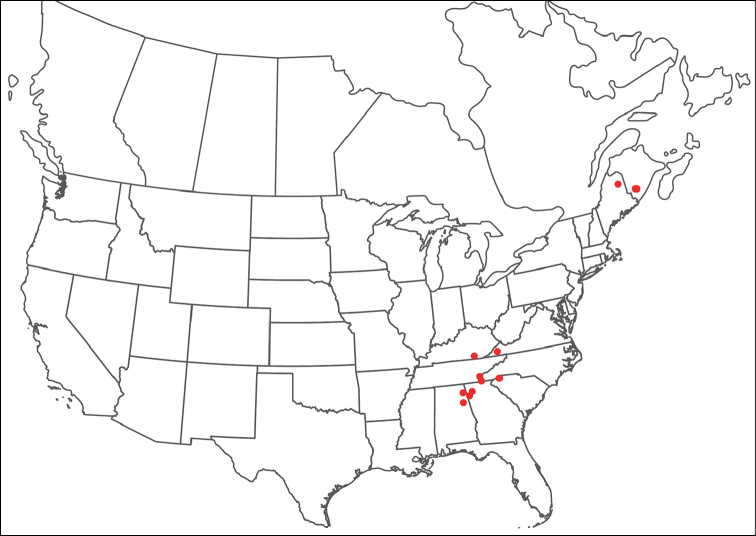
*Torrenticola gorti* sp. n. distribution.

Appalachians (Figure 90).

**Figure 91.**
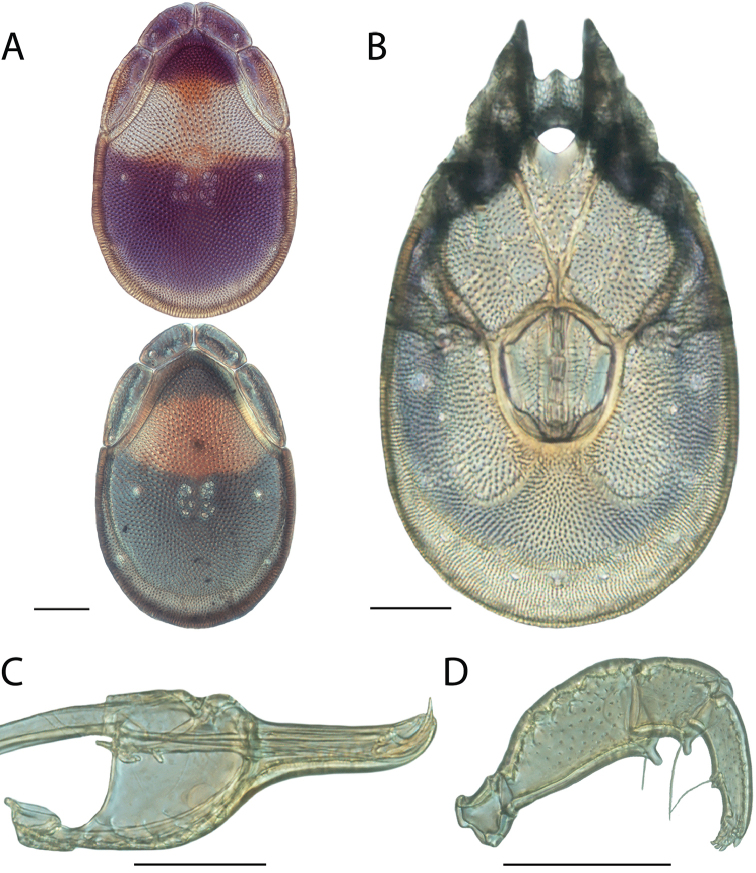
*Torrenticola gorti* sp. n. female, Tennessee specimen depicted except for A (top): **A** dorsal plates, note variation in color between Tennessee specimens (bottom) and elsewhere (top) **B** venter (legs removed) **C** subcapitulum **D** pedipalp (setae not accurately depicted). Scale = 100 µm.

**Figure 92.**
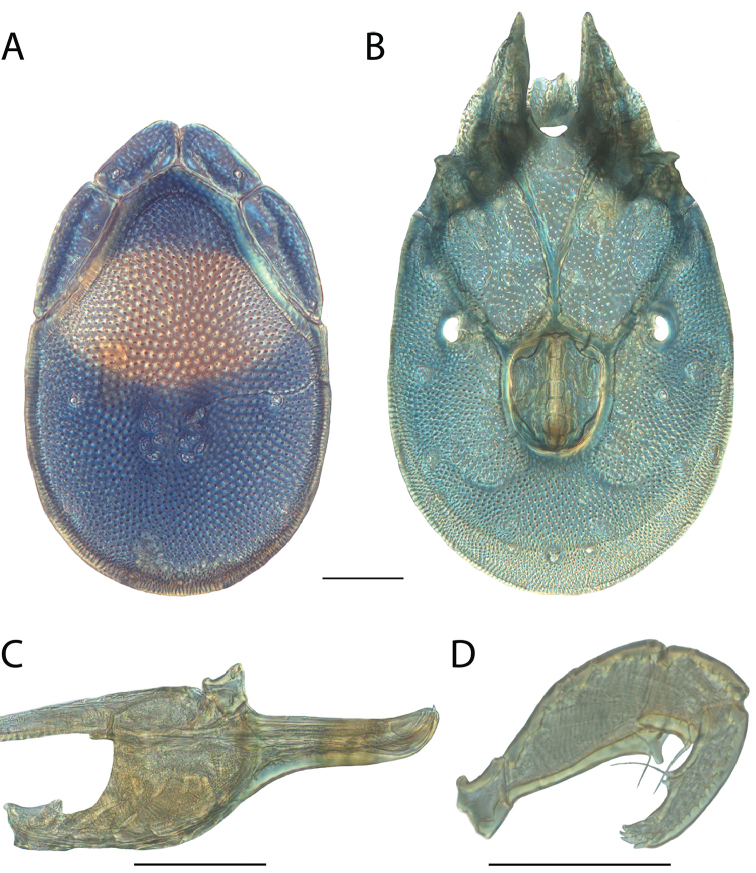
*Torrenticola gorti* sp. n. male, Tennessee specimen depicted: **A** dorsal plates **B** venter (legs removed) **C** subcapitulum **D** pedipalp (setae not accurately depicted). Scale = 100 µm.

######## Remarks.

*Torrenticola gorti* groups with other members of the Raptor Complex with high support and specimens of this species were less than 1% different in COI sequence from each other. In all analyses, *T. elongata* groups with two other species (*T. elongata* and *T. bondi*) which are 4% different from each other and have non-overlapping ranges.

*T. elongata* to form the Elongata Group in all analyses with high support.

Based upon overall similarity, an elongate body, and distribution, we place this species in the Elongata Identification Group.

This species hypothesis is supported by low COI divergence within the species (0–2%) and high divergence between species (3–15%), and by the morphological characters outlined in the diagnosis.

Members of this species can be highly variable in color. Some members have reddish-purple or purple dorsal coloration that is separated into anterior and posterior portions. Ventral coloration can be bold, faint, or absent. Members from Tellico River, Monroe County (Tennessee), can be readily differentiated from all other *Torrenticola* by being dark navy blue with a red dorsal oval.

####### Torrenticola hoosieri

Taxon classificationAnimaliaTrombidiformesTorrenticolidae

Fisher & Dowling sp. n.

http://zoobank.org/F64DDF23-2E2B-45EC-980E-06E07A3EEE0B

######## Material examined.

HOLOTYPE (♀): from USA, Indiana, Wayne County, south of I-70 (39°51'13"N, 85°8'4"W), 31 Jul 2014, by MJ Skvarla, MS 14-0731-001.

PARATYPES (1 ♀; 5 ♂): **Indiana**: 1 ♂ (ALLOTYPE) from Wayne County, south of I-70 (39°51'13"N, 85°8'4"W), 31 Jul 2014, by MJ Skvarla, MS 14-0731-001 • 1 ♀ and 4 ♂ from Wayne County, south of I-70 (39°51'13"N, 85°8'4"W), 31 Jul 2014, by MJ Skvarla, MS 14-0731-001.

######## Type deposition.

Holotype (♀), allotype (♂), and some paratypes (3 ♂) deposited in the CNC; other paratypes (1 ♀; 2 ♂) deposited in the ACUA.

######## Diagnosis.

*Torrenticola hoosieri* are similar to other members of the Tricolor Complex (*T. bittikoferae*, *T. larvata*, *T. pearsoni*, *T. olliei*, *T. sierrensis*, *T. tricolor*, *T. trimaculata*, and *T. unimaculata*, *T. cardia*, *T. kringi*, *T. dimorpha*, and *T. mohawk*) in having a short, conical rostrum. *T. hoosieri* can be differentiated from all other Tricolor Complex, and nearly all other *Torrenticola*, by lacking pedipalp ventral extensions on femora and genua. Additionally, *T. hoosieri* can be differentiated from all other Tricolor Complex by having more elongate pedipalp tibiae (3.67–4.33 in *T. hoosieri*, 2.65–3.55 in others) and by being colorless (rarely with diffuse pink dorsal coloration), except *T. bittikoferae*, *T. pearsoni*, and *T. dimorpha*.

######## Description.

**Female (Figure 94)** (n = 2) (holotype measurements in parentheses when available) with characters of the genus with following specifications.

**Dorsum** — (650–700 (650) long; 450–470 (450) wide) ellipsoid and colorless, occasionally with pink coloration without a distinct pattern. Anterio-medial platelets (115–125 (115) long; 55–60 (55) wide). Anterio-lateral platelets (177.5–180 (177.5) long; 67.5–67.5 (67.5) wide) free from dorsal plate. Dgl-4 much closer to the edge of the dorsum than to the muscle scars (distance between Dgl-4 345–375 (345)). Dorsal plate proportions: dorsum length/width 1.44–1.49 (1.44); dorsal width/distance between Dgl-4 1.25–1.30 (1.30); anterio-medial platelet length/width 2.08–2.09 (2.09); anterio-lateral platelet length/width 2.63–2.67 (2.63); anterio-lateral/anterio-medial length 1.44–1.54 (1.54).

**Gnathosoma — Subcapitulum** (277.5–300 (277.5) long (ventral); 202.5–221.23 (202.5) long (dorsal); 127.5–138.75 (127.5) tall) colorless. Rostrum (120–132.5 (120) long; 52.5–55 (52.5) wide) short and conical. Chelicerae (260–291 (260) long) with curved fangs (75–77 (75) long). Subcapitular proportions: ventral length/height 2.16–2.18 (2.18); rostrum length/width 2.29–2.41 (2.29). **Pedipalps** without extensions on femora and genua. Palpomeres: trochanter (50–55 (50) long); femur (130–137.5 (130) long); genu (72.5–80 (72.5) long); tibia (102.5–110 (102.5) long; 27.5–30 (27.5) wide); tarsus (25–27.5 (27.5) long). Palpomere proportions: femur/genu 1.72–1.79 (1.79); tibia/femur 0.79–0.80 (0.79); tibia length/width 3.67–3.73 (3.73).

**Venter** — (790–800 (800) long; 480–551 (480) wide) colorless. Gnathosomal bay (122.5–130 (130) long; 85–105 (85) wide). Cxgl-4 subapical. **Medial suture** (30–30 (30) long). **Genital plates** (182.5–188.75 (188.75) long; 150–152.5 (150) wide). Additional measurements: Cx-1 (257–260 (260) long (total); 120–129 (120) long (medial)); Cx-3 (330–390 (330) wide); anterior venter (162.5–167.5 (162.5) long). Ventral proportions: gnathosomal bay length/width 1.17–1.53 (1.53); anterior venter/genital field length 0.86–0.92 (0.86); anterior venter length/genital field width 1.08–1.10 (1.08); anterior venter/medial suture 5.42–5.58 (5.42).

**Male (Figure 95)** (n = 5) (allotypic measurements in parentheses when available) with characters of the genus with following specifications.

**Dorsum** — (580–640 (640) long; 390–410 (400) wide) ellipsoid and colorless. Anterio-medial platelets (110–117.5 (115) long; 55–60 (60) wide). Anterio-lateral platelets (167.5–175 (175) long; 65–72.5 (70) wide) free from dorsal plate. Dgl-4 much closer to the edge of the dorsum than to the muscle scars (distance between Dgl-4 320–360 (325)). Dorsal plate proportions: dorsum length/width 1.43–1.60 (1.60); dorsal width/distance between Dgl-4 1.14–1.23 (1.23); anterio-medial platelet length/width 1.91–2.05 (1.92); anterio-lateral platelet length/width 2.31–2.58 (2.50); anterio-lateral/anterio-medial length 1.43–1.59 (1.52).

**Gnathosoma — Subcapitulum** (245–280 (280) long (ventral); 185–202.5 (200) long (dorsal); 107.5–120 (120) tall) colorless. Rostrum (107.5–110 (110) long; 42.5–45 (45) wide). Chelicerae (245–260 (260) long) with curved fangs (65–70 (67.5) long). Subcapitular proportions: ventral length/height 2.23–2.36 (2.33); rostrum length/width 2.44–2.59 (2.44). **Pedipalps** without extensions on femora and genua. Palpomeres: trochanter (37.5–47.5 (45) long); femur (117.5–125 (125) long); genu (70–75 (75) long); tibia (97.5–100 (100) long; 22.5–25 (25) wide); tarsus (22.5–27.5 (27.5) long). Palpomere proportions: femur/genu 1.67–1.73 (1.67); tibia/femur 0.79–0.83 (0.80); tibia length/width 3.90–4.33 (4.00).

**Venter** — (670–740 (740) long; 450–495 (460) wide) colorless. Gnathosomal bay (120–130 (125) long; 80–85 (85) wide). Cxgl-4 subapical. **Medial suture** (102.5–122.5 (117.5) long). **Genital plates** (140–150 (147.5) long; 102.5–110 (105) wide). Additional measurements: Cx-1 (240–260 (260) long (total); 120–150 (130) long (medial)); Cx-3 (330–350 (345) wide); anterior venter (237.5–270 (270) long). Ventral proportions: gnathosomal bay length/width 1.47–1.59 (1.47); anterior venter/genital field length 1.70–1.84 (1.83); anterior venter length/genital field width 2.21–2.57 (2.57); anterior venter/medial suture 2.14–2.51 (2.30).

**Immatures** unknown.

######## Etymology.

Specific epithet (*hoosieri*) refers to Hoosier, the English demonym for a person from Indiana, the type locality.

######## Distribution.

**Figure 93.**
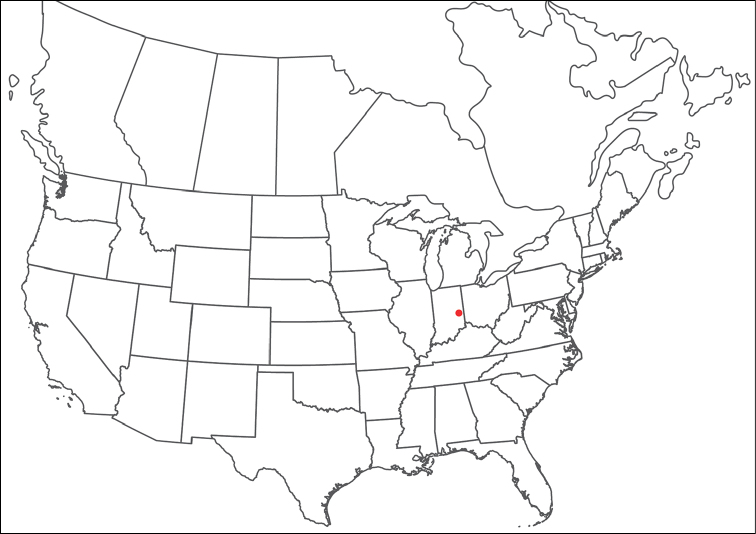
*Torrenticola hoosieri* sp. n. distribution.

Known only from Wayne County, Indiana (Figure 93).

**Figure 94.**
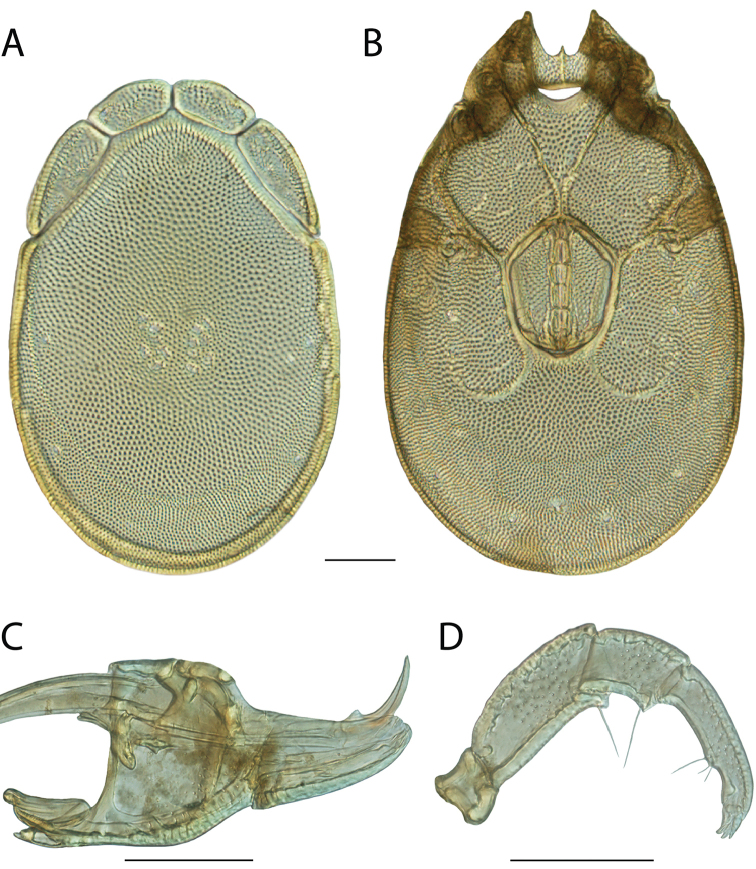
*Torrenticola hoosieri* sp. n. female: **A** dorsal plates **B** venter (legs removed) **C** subcapitulum, note damaged rostrum **D** pedipalp (setae not accurately depicted). Scale = 100 µm.

**Figure 95.**
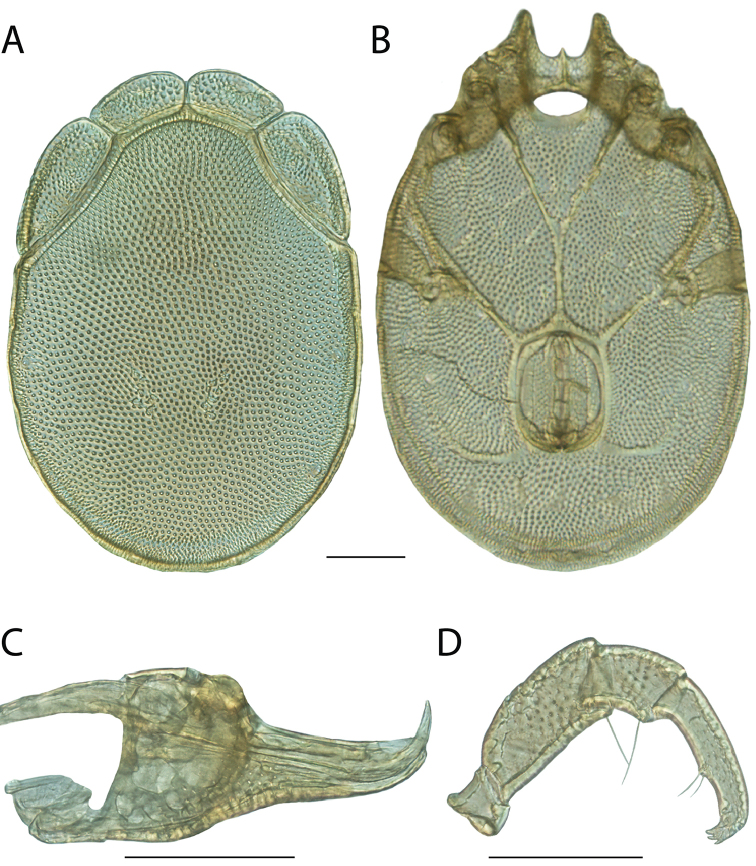
*Torrenticola hoosieri* sp. n. male: **A** dorsal plates **B** venter (legs removed) **C** subcapitulum **D** pedipalp (setae not accurately depicted). Scale = 100 µm.

######## Remarks.

*Torrenticola hoosieri* group with other members of the Tricolor Complex with high support and all specimens are less than 1% different in COI sequence from each other. In the combined analysis, *T. hoosieri* groups with other members of the Tricolor Complex from eastern North America that are not colorful, *T. projector* and *T. pearsoni*, and are greater than 4% different in COI from these species. This species is placed in the Tricolor Identification Group.

This species hypothesis is supported by low COI divergence within the species (0–2%), high divergence between species (3–15%), and the morphological characters outlined in the diagnosis.

####### Torrenticola indistincta

Taxon classificationAnimaliaTrombidiformesTorrenticolidae

(Marshall, 1929)

Atractides indistinctus Marshall, 1929: 317.
T. indistincta : Mitchell 1954: 40 • Viets 1956: 253 • Crowell 1960: 35, 37 • Crowell 1961: 330 • Habeeb 1967: 3 • Weaver 1967: 223 • Conroy 1968: 28 • Young 1969: 373-386 • Modlin and Gannon 1973: 219, 221 • Conroy and Scudder 1975: 307.

######## Material examined.

LECTOTYPE (1 ♀): from USA, Wisconsin, Green Lake County, Green Lake, Aug 1921, by C Juday, RM210013.

PARALECTOTYPE (1 ♂):from USA, Wisconsin, Green Lake County, Green Lake, Aug 1921, by C Juday, RM210013.

OTHER MATERIAL (5 ♀; 4 ♂): **Manitoba, Canada**: 1 ♂ from Fidler Lake; Station 7., (57°11'11"N, 96°56'56"W), 20 June 1977, by Freshwater Institute, IMS770231 • 1 ♀ from Northern Indian Lake; Station 9., (56°47'47"N, 98°56'56"W), 20 June 1977, by Freshwater Institute, IMS770224 • 1 ♂ from Northern Indian Lake; Station 1., (56°47'47"N, 98°56'56"W), 20 June 1977, by Freshwater Institute, IMS770232 • 1 ♀ from North Pine River near Pine River, 29 May 1981, by P Schefter, E Fuller, ROM810578 • 1 ♀ and 1 ♂ from Southern Indian Lake, (57°10'10"N, 98°29'29"W), 1 July 1977, by Freshwater Institute, IMS770234 • 1 ♀ and 1 ♂ from Southern Indian Lake, (56°47'47"N, 98°56'56"W), 27 July 1977, by Freshwater Institute., IMS770215 • 1 ♀ from Southern Indian Lake, (57°10'10"N, 98°29'29"W), 5 September 1978, by Freshwater Institute., IMS780049

######## Type deposition.

Types (1 ♀, 1 ♂); and most other material (3 ♀; 2 ♂) deposited in the CNC; other paratypes (2 ♀; 2 ♂) deposited in the ACUA.

######## Diagnosis.

*Torrenticola indistincta* are similar to other members of the Rusetria “Eastern 2-Plates” group (*T. biscutella*, *T. caerulea*, *T. delicatexa*, *T. malarkeyorum*, *T. pendula*, *T. sellersorum*, *T. tysoni*, *T. ululata*, *T. whitneyae*, *T. microbiscutella*, and *T. feminellai*) in having anterio-lateral platelets fused to the dorsal plate, and being distributed in the east. *T. indistincta* can be differentiated from other Eastern 2-Plates by having faint coloration separated into anterior and posterior portions connected medially. Female *T. indistincta* can be differentiated from female *T. caerulea* by having a larger genital field (length = 185–225 in *T. indistincta*, 155–165 in *T. caerulea*; width = 185–205 in *T. indistincta*, 120–145 in *T. caerulea*). Male *T. indistincta* can be differentiated from male *T. caerulea* by having a larger dorsum (length = 480–645 in *T. indistincta*, 405–460 in *T. caerulea*; width = 315–470 in *T. indistincta*, 260–305 in *T. caerulea*). *T. indistincta* can be differentiated from *T. microbiscutella* by having a less elongate dorsum (length/width = 1.21–1.52 in *T. indistincta*; 1.63–1.75 in *T. microbiscutella*).

######## Re-description.

**Female (Figure 97)** (n = 5) (lectotypic measurements in parentheses when available) with characters of the genus with following specifications.

**Dorsum** — (590–880 (640) long; 460–720 (485) wide) ovoid with faint coloration separated into anterior and posterior portions and connected medially. Anterio-medial platelets (125–195 (125) long; 45–77.5 (47.5) wide). Anterio-lateral platelets (172.5–235 (172.5) long; 75–115 (80) wide) fused to dorsal plate. Dgl-4 much closer to the edge of the dorsum than to the muscle scars (distance between Dgl-4 340–485 (340)). Dorsal plate proportions: dorsum length/width 1.21–1.32 (1.32); dorsal width/distance between Dgl-4 1.32–1.48 (1.43); anterio-medial platelet length/width 2.35–2.94 (2.63); anterio-lateral platelet length/width 2.02–2.33 (2.16); anterio-lateral/anterio-medial length 1.19–1.38 (1.38).

**Gnathosoma — Subcapitulum** (300–395 (322.5) long (ventral); 225–295 (237.5) long (dorsal); 145–195 (150) tall) tall and colorless. Rostrum (120–160 (125) long; 45–62.5 (47.5) wide). Chelicerae (300–405 (335) long) with curved fangs (60–80 (62.5) long). Subcapitular proportions: ventral length/height 1.97–2.15 (2.15); rostrum length/width 2.50–2.78 (2.63). **Pedipalps** with tuberculate ventral extensions on femora and genua. Palpomeres: trochanter (45–65 (47.5) long); femur (112.5–160 (117.5) long); genu (62.5–90 (62.5) long); tibia (87.5–122.5 (87.5) long; 25–31.25 (25) wide); tarsus (17.5–30 (17.5) long). Palpomere proportions: femur/genu 1.67–1.88 (1.88); tibia/femur 0.74–0.80 (0.74); tibia length/width 3.50–3.92 (3.50).

**Venter** — (650–995 (800) long; 535–880 (565) wide) colorless. Gnathosomal bay (175–237.5 (175) long; 90–130 (97.5) wide). Cxgl-4 subapical. **Medial suture** absent. **Genital plates** (185–225 (205) long; 185–205 (190) wide). Additional measurements: Cx-1 (295–385 (305) long (total); 120–155 (135) long (medial)); Cx-3 (345–505 (345) wide); anterior venter (125–167.5 (135) long). Ventral proportions: gnathosomal bay length/width 1.79–2.02 (1.79); anterior venter/genital field length 0.66–0.83 (0.66); anterior venter length/genital field width 0.66–0.83 (0.71).

**Male (Figure 98)** (n = 5) (lectotypic measurements in parentheses when available) with characters of the genus with following specifications.

**Dorsum** — (480–645 (480) long; 315–470 (315) wide) ovoid with faint coloration separated into anterior and posterior portions and connected medially. Anterio-medial platelets (102.5–147.5 (102.5) long; 35–55 (35) wide). Anterio-lateral platelets (137.5–180 (150) long; 55–82.5 (55) wide) fused to dorsal plate. Dgl-4 much closer to the edge of the dorsum than to the muscle scars (distance between Dgl-4 235–350 (235)). Dorsal plate proportions: dorsum length/width 1.37–1.52 (1.52); dorsal width/distance between Dgl-4 1.31–1.34 (1.34); anterio-medial platelet length/width 2.33–3.05 (2.93); anterio-lateral platelet length/width 2.12–2.73 (2.73); anterio-lateral/anterio-medial length 1.19–1.46 (1.46).

**Gnathosoma — Subcapitulum** (238.75–317.5 (238.75) long (ventral); 167.5–232.5 (167.5) long (dorsal); 102.5–137.5 (102.5) tall) tall and colorless. Rostrum (85–125 (85) long; 32.5–50 (32.5) wide). Chelicerae (232.5–320 (232.5) long) with curved fangs (47.5–65 (47.5) long). Subcapitular proportions: ventral length/height 2.22–2.33 (2.33); rostrum length/width 2.50–2.86 (2.62). **Pedipalps** with tuberculate ventral extensions on femora and genua. Palpomeres: trochanter (37.5–52.5 (37.5) long); femur (86.25–117.5 (86.25) long); genu (50–77.5 (50) long); tibia (70–100 (70) long; 22.5–28.75 (22.5) wide); tarsus (17.5–22.5 (17.5) long). Palpomere proportions: femur/genu 1.52–1.73 (1.73); tibia/femur 0.80–0.85 (0.81); tibia length/width 3.11–3.48 (3.11).

**Venter** — (570–780 (570) long; 370–510 (370) wide) colorless. Gnathosomal bay (127.5–180 (127.5) long; 65–100 (65) wide). Cxgl-4 subapical. **Medial suture** (65–75 (75) long). **Genital plates** (117.5–165 (117.5) long; 112.5–150 (112.5) wide). Additional measurements: Cx-1 (235–315 (235) long (total); 107.5–150 (107.5) long (medial)); Cx-3 (250–395 (277.5) wide); anterior venter (190–235 (190) long). Ventral proportions: gnathosomal bay length/width 1.80–1.96 (1.96); anterior venter/genital field length 1.38–1.68 (1.62); anterior venter length/genital field width 1.53–1.69 (1.69); anterior venter/medial suture 2.53–3.54 (2.53).

**Immatures** unknown.

######## Etymology.

Marshall (1929) named the specific epithet (*indistincta*) in reference to the Rusetria Complex character of fused (“indistinct”) lateral platelets.

######## Distribution.

**Figure 96.**
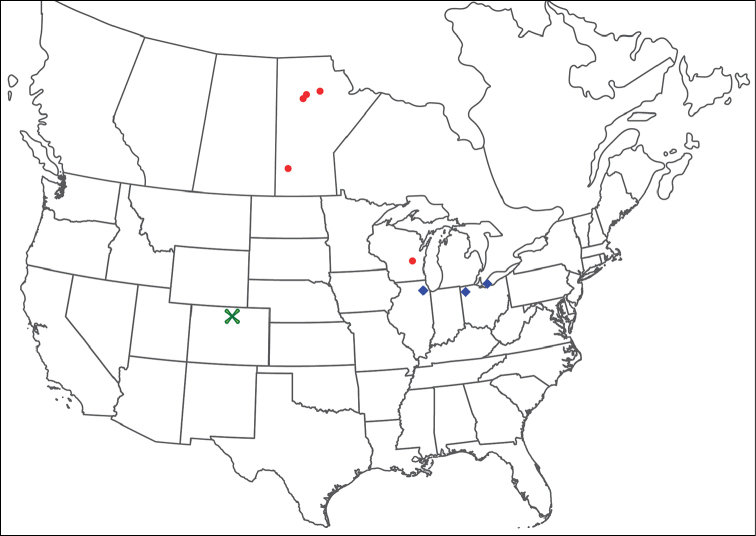
*Torrenticola indistincta* distribution. Red dots indicate material examined. Blue diamonds indicate previously published reports. Green “X” indicates previously published report (Young 1969) that requires verification of identification.

Midwest and into Manitoba (Figure 96). Young (1969) reported this species from Colorado, but this likely represents *T. mulleni* rather than *T. indistincta*.

**Figure 97.**
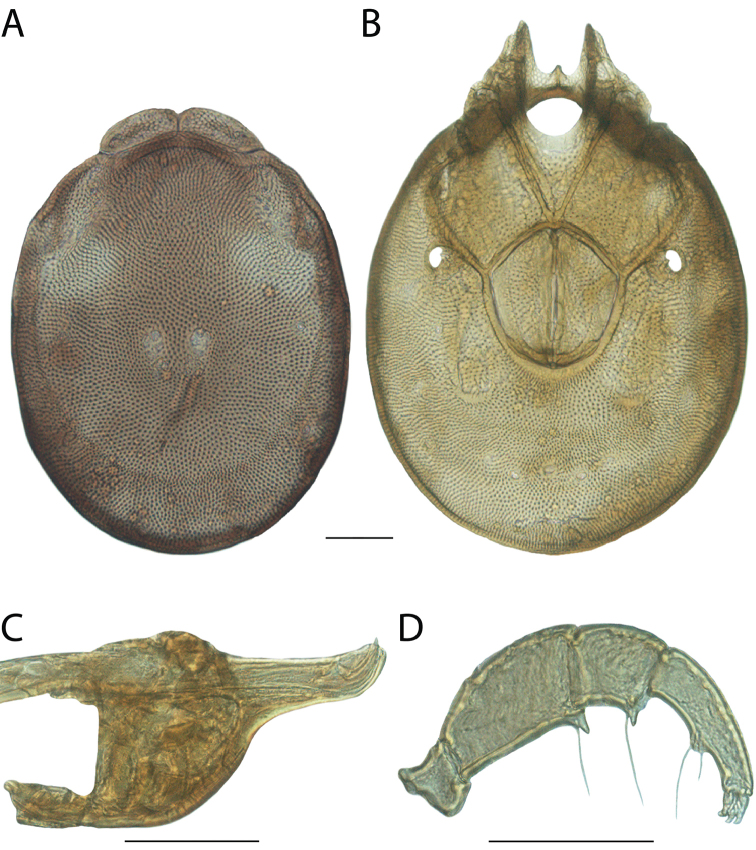
*Torrenticola indistincta* female: **A** dorsal plates **B** venter (legs removed) **C** subcapitulum **D** pedipalp (setae not accurately depicted). Scale = 100 µm.

**Figure 98.**
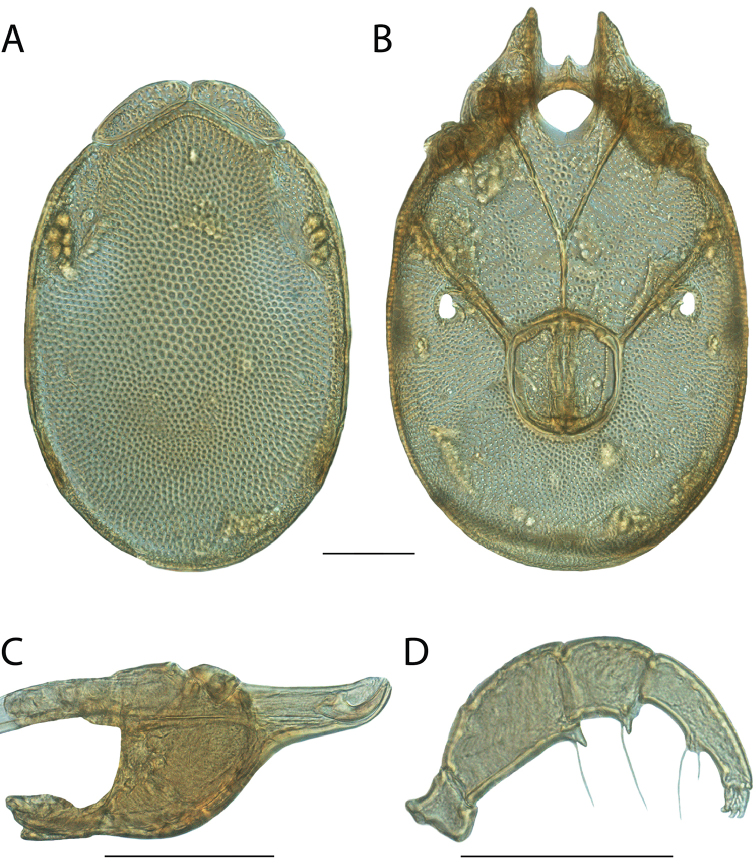
*Torrenticola indistincta* male: **A** dorsal plates **B** venter (legs removed) **C** subcapitulum **D** pedipalp (setae not accurately depicted). Scale = 100 µm.

######## Remarks.

Unfortunately, we were unable to acquire fresh material of *Torrenticola indistincta* and therefore this species was not included in our phylogenetic analyses. However, we were able to examine type material and additional material preserved in GAW. The fusion of the lateral platelets to the dorsal plate clearly places this species among the Rusetria Complex and its distribution is consistent with placement within the Eastern 2-Plate Identification Group.

Although Young (1969) reported this species in Colorado, we suspect that record represents the superficially similar *T. mulleni* based upon distribution.

####### Torrenticola interiorensis

Taxon classificationAnimaliaTrombidiformesTorrenticolidae

Fisher & Dowling sp. n.

http://zoobank.org/A334B365-1656-4A67-8D6F-82013B6A3F74

######## Material examined.

HOLOTYPE (♂): from USA, Arkansas, Montgomery County, Caddo Gap, access track off Manfred Road, 0.3 km west of Route 8, 29 Jul 2011, by IM Smith, IMS110037.

PARATYPES (7 ♀; 11 ♂): **Arkansas, USA**: 1 ♀ (ALLOTYPE) from Montgomery County, Caddo Gap, access track off Manfred Road, 0.3 km west of Route 8, 29 Jul 2011, by IM Smith, IMS110037 • 2 ♂ from Montgomery County, Caddo Gap, access track off Manfred Road, 0.3 km west of Route 8, 29 Jul 2011, by IM Smith, IMS110037 • 1 ♀ and 1 ♂ from Newton County, Ozark-St. Francis National Forest, Little Buffalo River, 11 Jul 2012, by TD Edwards, TDE 12-0711-004 • **Missouri, USA**: 3 ♀ and 5 ♂ from Crawford County, Huzzah Creek, Red Bluff campground, off Road V, east of Davisville, 23 Jul 2011, by IM Smith, IMS110029 • **Oklahoma, USA**: 2 ♀ and 3 ♂ from Pushmataha County, Walnut Creek, beside Route 271, south of Albion, 1 Jul 1987, by IM Smith, IMS870063A.

######## Type deposition.

Holotype (♂), allotype (♀), and some paratypes (5 ♀; 5 ♂) deposited in the CNC; other paratypes (2 ♀; 5 ♂) deposited in the ACUA.

######## Diagnosis.

*Torrenticola interiorensis* are similar to species with similar dorsal patterning, such as the Rusetria 4-Plate Group (*T. dunni*, *T. glomerabilis*, *T. kittatinniana*, *T. pollani*, *T. rufoalba* and *T. shubini*), Elongata Group (*T. gorti*, *T. elongata*, and *T. reduncarostra*), and *T. bondi*, *T. erectirostra*, *T. robisoni*, *T. irapalpa*, *T. neoanomala*, *T. racupalpa*, *T. skvarlai*, and *T. arktonyx*. They can be differentiated from Rusetria 4-Plates and *T. skvarlai* by having distinct hind coxal margins. *T. interiorensis* can be differentiated from *T. erectirostra*, *T. robisoni* and *T. reduncarostra* by having a straight, anteriorly-directed rostrum (upturned in *T. erectirostra* and *T. reduncarostra*). *T. interiorensis* can be differentiated from *T. arktonyx* by having an unmodified dorsal plate (*T. arktonyx* has distinctive longitudinal dark markings on the anterior portion of the dorsal plate). *T. interiorensis* can be differentiated from *T. racupalpa* by having less elongate pedipalpal tibiae (length/width = 3.76–4.22 in *T. interiorensis*; 4.44–5.50 in *T. racupalpa*) and less elongate rostrum (length/width = 2.63–2.88 in *T. interiorensis*; 3.56–3.88 in *T. racupalpa*). *T. interiorensis* can be differentiated from *T. irapalpa* by having Dgl-4 closer to the dorsal edge (dorsal width/distance between Dgl-4 ♀ = 1.48–1.61 in *T. interiorensis*, 1.81–2.09 in *T. irapalpa*; ♂ = 1.42–1.45 in *T. interiorensis*, 1.58–1.86 in *T. irapalpa*) and more ovoid dorsum (length/width ♀ = 1.29–1.38 in *T. interiorensis*, 1.20–1.28 in *T. irapalpa*; ♂ = 1.34–1.47 in *T. interiorensis*, 1.26–1.30 in *T. irapalpa*). *T. interiorensis* can be differentiated from Elongata Group by being slightly more ovoid (dorsum length/width ♀ = 1.29–1.38 in *T. interiorensis*, 1.45–2.08 in Elongata Group; ♂ = 1.34–1.47 in *T. interiorensis*, 1.51–1.7 in Elongata Group) and having a stockier rostrum (length/width = 2.63–2.88 in *T. interiorensis*, 3.24–4.00 in Elongata Group). *T. interiorensis* can be differentiated from *T. bondi* by having a longer medial suture (♀ = 25–30 in *T. interiorensis*, 10–15 in *T. bondi*; ♂ = 75–83 in *T. interiorensis*, 55–70 in *T. bondi*), anterior venter/genital field width (♀ = 1.31–1.38 in *T. interiorensis*, 1.15–1.25 in *T. bondi*; ♂ = 2.09–2.27 in *T. interiorensis*, 1.95–2.05 in *T. bondi*), and Dgl-4 closer to edge of dorsum (dorsum width/distance between Dgl-4 ♀ = 1.48–1.61 in *T. interiorensis*, 1.63–1.72 in *T. bondi*; ♂ = 1.42–1.45 in *T. interiorensis*, 1.48–1.62 in *T. bondi*). Female *T. interiorensis* can be differentiated from female *T. neoanomala* by having stockier anterio-lateral platelets (length/width = 2.62–2.67 in *T. interiorensis*, 2.86–3.09 in *T. neoanomala*). Male *T. interiorensis* can be differentiated from male *T. neoanomala* by having a shorter anterior venter (220–240 in *T. interiorensis*, 267.5–290 in *T. neoanomala*) and a shorter genital field (132–138 in *T. interiorensis*, 145–160 in *T. neoanomala*).

######## Description.

**Male (Figure 100)** (n = 5) (holotype measurements in parentheses when available) with characters of the genus with following specifications.

**Dorsum** — (510–545 (510) long; 350–405 (360) wide) ovoid with purple coloration separated into anterior and posterior portions and orange medially. Anterio-medial platelets (115–122.5 (115) long; 45–50 (45) wide). Anterio-lateral platelets (150–167.5 (150) long; 50–52.5 (50) wide) free from dorsal plate. Dgl-4 closer to the edge of the dorsum than to the muscle scars (distance between Dgl-4 245–285 (250)). Dorsal plate proportions: dorsum length/width 1.34–1.47 (1.42); dorsal width/distance between Dgl-4 1.42–1.45 (1.44); anterio-medial platelet length/width 2.45–2.72 (2.56); anterio-lateral platelet length/width 3.00–3.19 (3.00); anterio-lateral/anterio-medial length 1.30–1.37 (1.30).

**Gnathosoma — Subcapitulum** (265–282.5 (265) long (ventral); 200–212 (200) long (dorsal); 105–112.5 (105) tall) colorless. Rostrum (112.5–115 (112.5) long; 40–40 (40) wide). Chelicerae (260–282 (260) long) with curved fangs (45–58 (45) long). Subcapitular proportions: ventral length/height 2.51–2.60 (2.52); rostrum length/width 2.81–2.88 (2.81). **Pedipalps** with tuberculate ventral extensions on femora and genua. Palpomeres: trochanter (32.5–37.5 (32.5) long); femur (97.5–105 (97.5) long); genu (55–62.5 (55) long); tibia (77.5–81.25 (77.5) long; 20–21.25 (20) wide); tarsus (17.5–18.75 (17.5) long). Palpomere proportions: femur/genu 1.68–1.78 (1.77); tibia/femur 0.77–0.79 (0.79); tibia length/width 3.76–3.88 (3.88).

**Venter** — (608–660 (640) long; 424–480 (480) wide) mostly colorless with faint purple in areas surrounding coxae. Gnathosomal bay (102.5–137.5 (110) long; 65–85 (75) wide). Cxgl-4 subapical. **Medial suture** (75–82.5 (75) long). **Genital plates** (132.5–137.5 (132.5) long; 100–107.5 (100) wide). Additional measurements: Cx-1 (231–270 (260) long (total); 123.25–134 (130) long (medial)); Cx-3 (300–338 (315) wide); anterior venter (220–240 (220) long). Ventral proportions: gnathosomal bay length/width 1.37–2.12 (1.47); anterior venter/genital field length 1.60–1.80 (1.66); anterior venter length/genital field width 2.09–2.27 (2.20); anterior venter/medial suture 2.73–2.98 (2.93).

**Female (Figure 101)** (n =5) (allotypic measurements in parentheses when available) with characters of the genus with following specifications.

**Dorsum** — (550–620 (550) long; 410–480 (415) wide) ovoid with purple coloration separated into anterior and posterior portions and orange coloration. Anterio-medial platelets (123.75–142.5 (123.75) long; 50–60 (50) wide). Anterio-lateral platelets (160–187.5 (160) long; 60–70 (60) wide) free from dorsal plate. Dgl-4 closer to the edge of the dorsum than to the muscle scars (distance between Dgl-4 255–305 (280)). Dorsal plate proportions: dorsum length/width 1.29–1.38 (1.33); dorsal width/distance between Dgl-4 1.48–1.61 (1.48); anterio-medial platelet length/width 2.36–2.52 (2.48); anterio-lateral platelet length/width 2.62–2.68 (2.67); anterio-lateral/anterio-medial length 1.21–1.33 (1.29).

**Gnathosoma — Subcapitulum** (307.5–325 (310) long (ventral); 220–240 (232.5) long (dorsal); 120–130 (120) tall) colorless. Rostrum (122.5–140 (125) long; 45–50 (45) wide). Chelicerae (300–340 (310) long) with curved fangs (50–65 (55) long). Subcapitular proportions: ventral length/height 2.44–2.58 (2.58); rostrum length/width 2.63–2.83 (2.78). **Pedipalps** with tuberculate ventral extensions on femora and genua. Palpomeres: trochanter (40–45 (40) long); femur (112.5–127.5 (115) long); genu (65–70 (65) long); tibia (85–95 (85) long; 21.25–22.5 (21.25) wide); tarsus (17.5–22.5 (20) long). Palpomere proportions: femur/genu 1.73–1.82 (1.77); tibia/femur 0.74–0.76 (0.74); tibia length/width 3.78–4.22 (4.00).

**Venter** — (680–780 (680) long; 470–580 (510) wide) mostly colorless with purple in areas surrounding coxae. Gnathosomal bay (112.5–152.5 (145) long; 70–100 (90) wide). Cxgl-4 subapical. **Medial suture** (25–30 (30) long). **Genital plates** (155–175 (155) long; 137.5–152.5 (145) wide). Additional measurements: Cx-1 (242–310 (280) long (total); 125–160 (140) long (medial)); Cx-3 (320–390 (350) wide); anterior venter (190–205 (190) long). Ventral proportions: gnathosomal bay length/width 1.22–2.07 (1.61); anterior venter/genital field length 1.17–1.23 (1.23); anterior venter length/genital field width 1.31–1.38 (1.31); anterior venter/medial suture 6.33–8.20 (6.33).

**Immatures** unknown.

######## Etymology.

Specific epithet (*interiorensis*) refers to the Interior Highlands, where this species was found within both major regions (Ozarks and Ouachitas), but not found outside these regions, which suggests it is endemic to the region.

######## Distribution.

**Figure 99.**
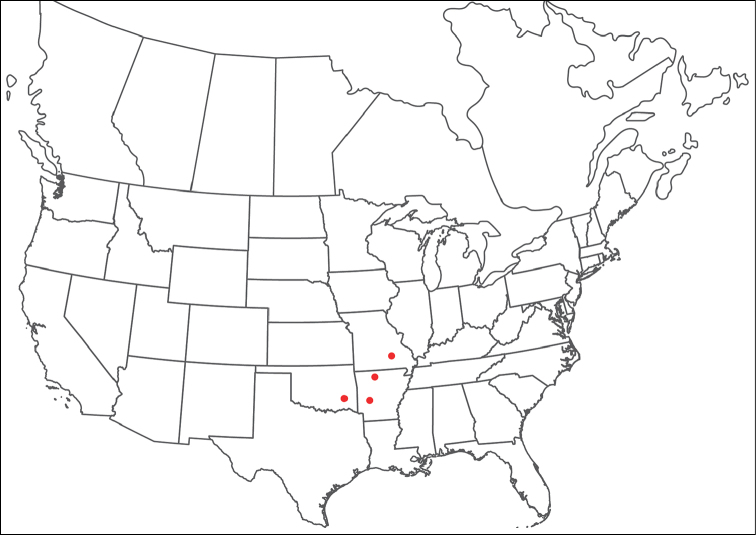
*Torrenticola interiorensis* sp. n. distribution.

Interior Highlands (Ozarks and Ouachitas), likely endemic (Figure 99).

**Figure 100.**
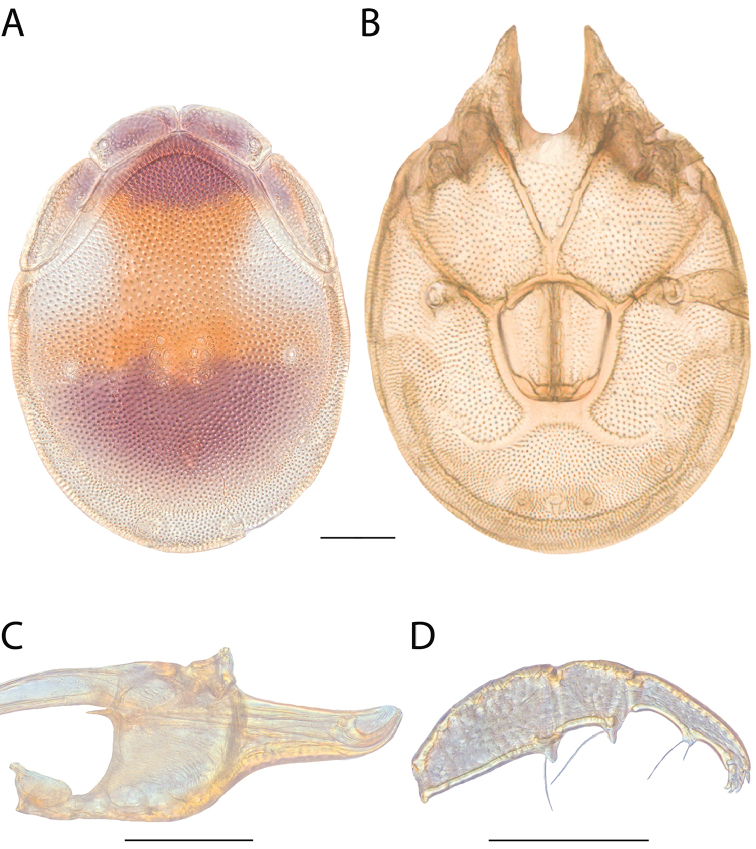
*Torrenticola interiorensis* sp. n. female: **A** dorsal plates **B** venter (legs removed) **C** subcapitulum **D** pedipalp (setae not accurately depicted). Scale = 100 µm.

**Figure 101.**
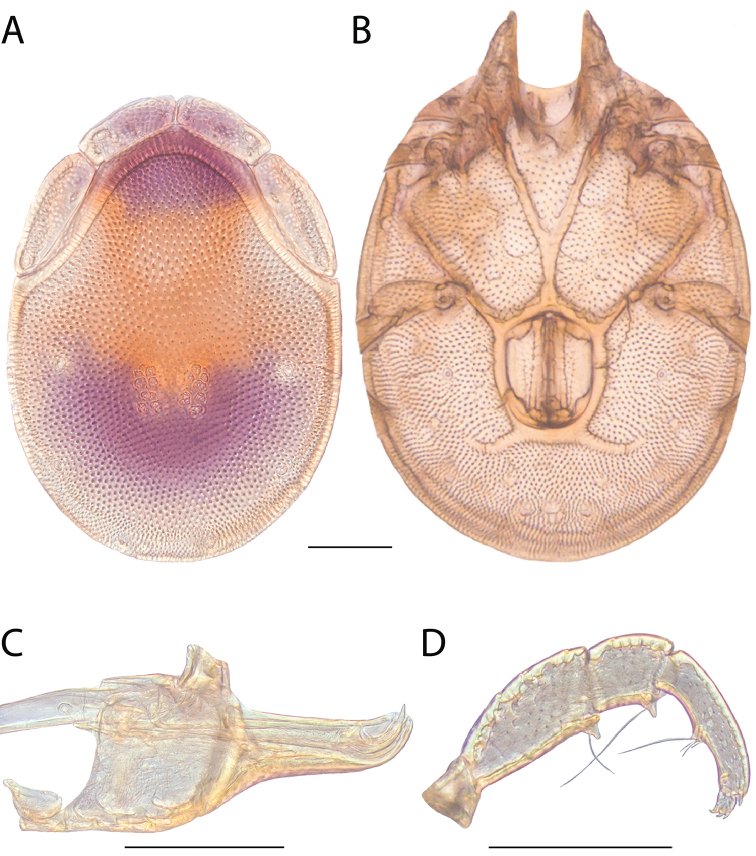
*Torrenticola interiorensis* sp. n. male: **A** dorsal plates **B** venter (legs removed) **C** subcapitulum **D** pedipalp (setae not accurately depicted). Scale = 100 µm.

######## Remarks.

*Torrenticola interiorensis* groups with other members of the Raptor Complex with high support and specimens are less than 2% different in COI sequence from each other. In the combined analysis, *T. interiorensis* groups with the superficially similar *T. neoanomala*, and specimens from these species are greater than 9% different in COI sequence from each other. Based upon this relationship and their similarity, we place these species in the Neoanomala Identification Group. The Neoanomala Group shares a phylogenetic affinity for members of the similar-looking Erectirostra Group.

This species hypothesis is supported by low COI divergence within the species (0–2%) and high divergence between species (3–15%), and by the morphological characters outlined in the diagnosis.

####### Torrenticola irapalpa

Taxon classificationAnimaliaTrombidiformesTorrenticolidae

Fisher & Dowling sp. n.

http://zoobank.org/FEAF955F-6EC2-4A5C-B97F-6168103C5003

######## Material examined.

HOLOTYPE (♀): from New Brunswick, Canada, York County, Stanley, Nashwaak River, Stanley Municipal Park, 19 Jun 2012, by IM Smith, IMS120031, DNA 2956.

PARATYPES (39 ♀; 26 ♂): **Arkansas, USA**: 1 ♀ from Newton County, Buffalo National River, Cecil Creek (36°5'15.72"N, 93°13'23.28"W), 13 Jun 2012, by TD Edwards, TDE 12-0613-010 • 1 ♂ from Newton County, Ozark-St. Francis National Forest, Little Buffalo River, 11 Jun 2012, by TD Edwards, TDE 12-0711-004 • 2 ♀ from Polk County, beside Forest Route 38, north of Shady Lake Recreation Area, East Saline Creek (34°22'23.39"N, 94°1'51.22"W), 30 Jul 2011, by IM Smith, IMS110041 • **Kentucky, USA**: 2 ♀ and 1 ♂ from McCreary County, White Oak Junction, Rock Creek, beside Forest Route 556, 2.3 kilometers south of Route 1363, 8 Jul 1990, by IM Smith, IMS900082A & IMS900082B • **Illinois, USA**: 3 ♀ and 1 ♂ from Kankakee County, Kankakee River State Park, beside Route 102, Rock Creek (41°12'N, 88°0'W), 9 Sep 1991, by IM Smith, IMS910027A • **Indiana, USA**: 1 ♀ from Wayne County, south of intersection of Interstate 70 and Route 1 (39°51'13"N, 85°8'4"W), 31 Jul 2014, by MJ Skvarla, MS 14-0731-001 • **Missouri, USA**: 3 ♀ Wayne County, beside Road 143 near Sam A Baker State Park, 8 Jul 1960, by DR Cook, DRC600010 • **New Brunswick, Canada**: 2 ♀ and 1 ♂ from Charlotte County, Rollingdam, Digdeguash River, beside Highway 770 at covered bridge, 30 Jun 1989, by IM Smith, IMS890053 • 1 ♀ and 1 ♂ from Charlotte County, Digdeguash River, beside Sorrel Ridge Road, west of Whittier Road, 10 Jun 2012, by IM Smith, IMS120015 • 2 ♀ and 1 ♂ from Charlotte County, Rollingham, Digdegaush River, beside Highway 770, 3 Oct 2011, by IM Smith, IMS110118 • 1 ♂ (ALLOTYPE) from York County, Stanley, Nashwaak River, Stanley Municipal Park, 19 Jun 2012, by IM Smith, IMS120031, DNA 2957 • **Ohio, USA**: 1 ♂ from Montgomery County, Engelwood Metro Park (39°52'58"N, 84°17'33"W), 31 Jul 2014, by MJ Skvarla, MS 14-0731-002 • 1 ♀ and 2 ♂ from Pickaway County, beside Scioto-Darby Road, just north of Darby, Big Darby Creek (40°2'N, 83°15'W), 30 Jun 1997, by IM Smith & M MacKenzie, IMS970016 • **Ontario, Canada**: 1 ♀ and 1 ♂ from Grey County, Durham, Saugeen River, beside County Road 27 near Durham Conservation Area, 9 Jun 1989, by IM Smith, IMS890028A • **Pennsylvania, USA**: 1 ♀ from Bedford County, Chaneysville, Sweet Root Picnic Area beside Route 326, 18 Jul 1990, by IM Smith, IMS900105 • 1 ♀ from Huntingdon County, Alan Seeger Natural Area, Stone Creek, beside road from McAlevys Fort to Route 322, 19 Jul 1990, by IM Smith, IMS900107 • 1 ♂ from Tioga County, Straight Run, beside Straight Run Road, 2.1 kilometers north of Route 6 between Ansonia & Wellsboro, 20 Jul1990, by IM Smith, IMS900111 • **Saskatchewan, Canada**: 2 ♀ and 2 ♂ from Torch River beside Highway 106 at access road, 30 kilometers north of Highway 55 at Smeaton, 30 Jul 1988, by IM Smith, IMS880054A • 2 ♀ and 2 ♂ from Torch River at end of forest access road, 10 kilometers north of Highway 55 at Love, 30 Jul 1988, by IM Smith, IMS880056 • **Texas, USA**: 2 ♀ from Bandera County, Vanderpool, beside Route 187, Sabinal River (29°48'10"N, 99°34'30"W), 2 May 2009, by IM Smith, IMS090007 • 1 ♀ and 1 ♂ from Kinney County, Brackettville, beside Route 90, 12.1 kilometers west of Route 131, Pinto Creek (29°20'6"N, 100°32'5"W), 28 Sep 1994, by IM Smith, IMS940025 • 1 ♀ from Uvalde County, Garner State Park, Frio River (29°35'22"N, 99°44'12"W), 28 May 1998, by IM Smith, IMS980027A • 1 ♀ and 1 ♂ from Val Verde County, Bakers Crossing Campground off Route 163, Devils River (29°58'N, 101°9'W), 5 Oct 1999, by IM Smith, IMS990061A • 2 ♀ from Val Verde County, Dolan Falls Preserve, Devils River (29°53'12"N, 100°59'37"W), 24 May 2011, by IM Smith, IMS110013 • 4 ♀ and 4 ♂ from Val Verde County, Dolan Falls Preserve, Snake Spring (29°53'43"N, 100°58'58"W), 25 May 2011, by IM Smith, IMS110015 • **Virginia, USA**: 1 ♀ and 1 ♂ from Bath County, Jackson River, beside Forest Route 1843 (continuation of Route 623), 3.5 kilometers south of Route 220, 16 Jul 1990, by IM Smith, IMS900100 • 2 ♀ and 2 ♂ from Scott County, North Fork Holston River, beside Route 58/421, 0.9 kilometers east of Route 709 at Hiltons, 7 Jul 1990, by IM Smith, IMS900080 • **West Virginia, USA**: 1 ♀ and 1 ♂ from Pendleton County, South Branch of the Potomac River, beside Route 28/55, 20.8 kilometers southwest of Route 42, 17 Jul 1990, by IM Smith, IMS900104.

######## Type deposition.

Holotype (♀), allotype (♂), and most paratypes (34 ♀; 20 ♂) deposited in the CNC; other paratypes (5 ♀; 5 ♂) deposited in ACUA.

######## Diagnosis.

*Torrenticola irapalpa* are similar to other members of the Raptor Group (*T. gnoma*, *T. longitibia*, *T. mjolniri*, *T. elusiva*, *T. racupalpa*, *T. raptor*, *T. danielleae*, *T. daemon*, and *T. ivyae*) in having round bodies; Dgl-4 close to muscles scars; long, thin subcapitular rostra; and long, thin pedipalp tibiae. *T. irapalpa* can be differentiated from *T. longitibia*, *T. mjolniri*, *T. elusiva*, *T. racupalpa*, and *T. ivyae* by having Dgl-4 closer to the dorsal edge (dorsal width/distance between Dgl-4 = 1.81–2.09 in *T. irapalpa*, 2.19–2.77 in others) and a less elongate rostrum (length/width = 2.66–3.39 in *T. irapalpa*; 3.56–4.32 in others). *T. irapalpa* can be differentiated from *T. gnoma* by having Dgl-4 closer to the dorsal edge (dorsal width/distance between Dgl-4 ♀ = 1.81–2.09 in *T. irapalpa*, 2.65–3.29 in *T. gnoma*; ♂ = 1.58–1.86 in *T. irapalpa*, 2.06–2.73 in *T. gnoma*) and by dorsal coloration and pattern. *T. irapalpa* can be differentiated from *T. raptor* by having less elongate tibiae (length/width ♀ = 4.09–5.67 in *T. irapalpa*, 6.00–7.54 in *T. raptor*; ♂ = 4.25–4.75 in *T. irapalpa*, 5.29–5.63 in *T. raptor*); less elongate subcapitulum (ventral length/height = 2.52–2.90 in *T. irapalpa*, 2.98–3.27 in *T. raptor*); and by dorsal pattern. *T. irapalpa* can be differentiated from *T. danielleae* by having Dgl-4 further from the dorsal edge (dorsal width/distance between Dgl-4 = ♀ = 1.81–2.09 in *T. irapalpa*, 1.57–1.70 in *T. danielleae*; ♂ = 1.58–1.86 in *T. irapalpa*, 1.42–1.52 in *T. danielleae*); a less elongate rostrum (length/width = 2.66–3.39 in *T. irapalpa*, 3.43–3.75 in *T. danielleae*); and by dorsal coloration and pattern. Female *T. irapalpa* can be differentiated from female *T. daemon* by having Dgl-4 further from the dorsal edge (dorsal width/distance between Dgl-4 ♀ = 1.81–2.09 in *T. irapalpa*, 1.59–1.67 in *T. daemon)* and a less elongate gnathosomal bay (length/width ♀ = 1.35–1.86 in *T. irapalpa*, 1.95–2.42 in *T. daemon*). Male *T. irapalpa* can be differentiated from male *T. daemon* by dorsal coloration and pattern.

######## Description.

**Female (Figure 103)** (n = 9) (holotype measurements in parentheses when available) with characters of the genus with following specifications.

**Dorsum** — (575–710 (610) long; 465–580 (475) wide) circular with navy blue to bluish-purple coloration separated into anterior and posterior portions with bold orange medially. Anterio-medial platelets (132.5–160 (137.5) long; 52.5–67.5 (62.5) wide). Anterio-lateral platelets (155–197.5 (177.5) long; 67.5–87.5 (77.5) wide) free from dorsal plate. Dgl-4 midway between muscle scars and dorsum edge (distance between Dgl-4 465–580 (475)). Dorsal plate proportions: dorsum length/width 1.20–1.28 (1.28); dorsal width/distance between Dgl-4 1.81–2.09 (1.90); anterio-medial platelet length/width 2.12–2.52 (2.20); anterio-lateral platelet length/width 2.17–2.39 (2.29); anterio-lateral/anterio-medial length 1.17–1.42 (1.29).

**Gnathosoma — Subcapitulum** (300–360 (340) long (ventral); 219–270 (270) long (dorsal); 125–157.5 (145) tall) with faint navy blue to bluish-purple coloration, sometimes colorless. Rostrum (126.25–152.5 (142.5) long; 55–76 (65) wide) elongate. Chelicerae (245–360 (360) long) with curved fangs (55–75 (65) long). Subcapitular proportions: ventral length/height 2.29–2.57 (2.34); rostrum length/width 2.66–3.39 (3.17). **Pedipalps** elongate (especially tibiae) with long tuberculate ventral extensions on femora and genua. Palpomeres: trochanter (45–52.5 (45) long); femur (115–132.5 (132.5) long); genu (65–80 (72.5) long); tibia (100–117.5 (102.5) long; 17.5–20 (20) wide); tarsus (17.5–20 (20) long). Palpomere proportions: femur/genu 1.66–1.89 (1.83); tibia/femur 0.77–0.89 (0.77); tibia length/width 4.09–5.67 (4.82).

**Venter** — (690–870 (770) long; 508–743 (550) wide) with navy blue to bluish purple coloration. Gnathosomal bay (132.5–165 (165) long; 87.5–120 (90) wide). Cxgl-4 far from apex. **Medial suture** (10–22.5 (15) long). **Genital plates** (155–175 (175) long; 140–162.5 (150) wide). Additional measurements: Cx-1 (266–334 (310) long (total); 103–156 (140) long (medial)); Cx-3 (332–487 (375) wide); anterior venter (156–197.5 (182.5) long). Ventral proportions: gnathosomal bay length/width 1.35–1.86 (1.83); anterior venter/genital field length 0.99–1.18 (1.04); anterior venter length/genital field width 1.06–1.27 (1.22); anterior venter/medial suture 8.56–15.63 (12.17).

**Male (Figure 104)** (n = 8) (allotypic measurements in parentheses when available) with characters of the genus with following specifications.

**Dorsum** — (520–620 (560) long; 400–490 (435) wide) circular with navy blue to bluish-purple coloration separated into anterior and posterior portions with bold orange medially. Anterio-medial platelets (115–150 (140) long; 55–67.5 (62.5) wide). Anterio-lateral platelets (160–188.75 (187.5) long; 62.5–77.5 (75) wide) free from dorsal plate. Dgl-4 midway between muscle scars and dorsum edge (distance between Dgl-4 220–310 (250)). Dorsal plate proportions: dorsum length/width 1.26–1.30 (1.29); dorsal width/distance between Dgl-41.58–1.86 (1.74); anterio-medial platelet length/width 2.00–2.48 (2.24); anterio-lateral platelet length/width 2.34–2.60 (2.50); anterio-lateral/anterio-medial length 1.22–1.41 (1.34).

**Gnathosoma — Subcapitulum** (275–340 (310) long (ventral); 207–240 (240) long (dorsal); 105–132.5 (107.5) tall) with faint navy blue to bluish-purple coloration, sometimes colorless. Rostrum (112.5–137.5 (135) long; 40–47.5 (40) wide) elongate. Chelicerae (252–305 (305) long) with curved fangs (51–60 (55) long). Subcapitular proportions: ventral length/height 2.52–2.90 (2.88); rostrum length/width 2.81–3.38 (3.38). **Pedipalps** elongate (especially tibiae) with long tuberculate ventral extensions on femora and genua. Palpomeres: trochanter (35–50 (42.5) long); femur (107.5–123.75 (115) long); genu (60–70 (67.5) long); tibia (87.5–108.75 (95) long; 20–25 (20) wide); tarsus (18.75–20 (20) long). Palpomere proportions: femur/genu 1.70–1.83 (1.70); tibia/femur 0.81–0.91 (0.83); tibia length/width 4.25–4.75 (4.75).

**Venter** — (630–705 (690) long; 490–540 (500) wide) with navy blue to bluish purple coloration. Gnathosomal bay (135–177.5 (146.25) long; 75–95 (75) wide). Cxgl-4 far from apex. **Medial suture** (50–75 (75) long). **Genital plates** (127.5–158.75 (143.75) long; 100–137.5 (122.5) wide). Additional measurements: Cx-1 (260–290 (290) long (total); 137–155 (155) long (medial)); Cx-3 (323–360 (345) wide); anterior venter (215–255 (245) long). Ventral proportions: gnathosomal bay length/width 1.42–2.06 (1.95); anterior venter/genital field length 1.51–1.75 (1.70); anterior venter length/genital field width 1.78–2.20 (2.00); anterior venter/medial suture 3.27–4.40 (3.27).

**Immatures** unknown.

######## Etymology.

Specific epithet (*irapalpa*) refers to the pedipalps of this species, which resemble ferocious weapons of wrath (*ira*, L. fury, rage, wrath; *palpus*, L. hand, feeler).

######## Distribution.

**Figure 102.**
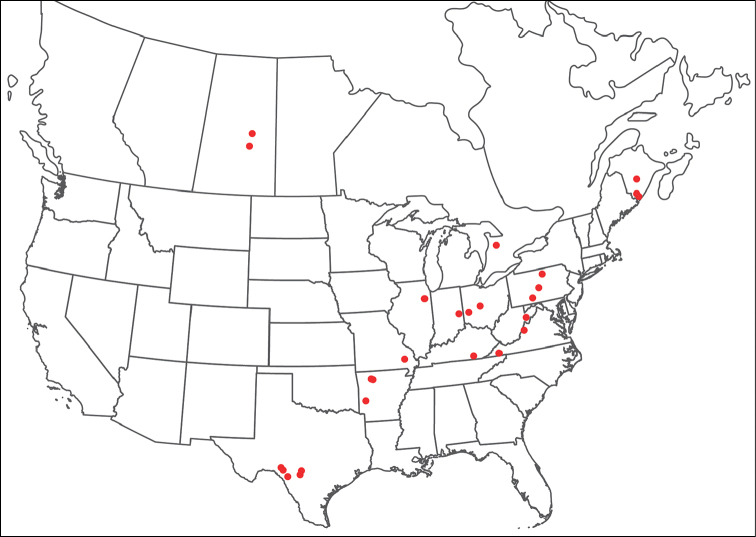
*Torrenticola irapalpa* sp. n. distribution.

Primarily eastern (Figure 102), but note that *T. irapalpa* is one of few *Torrenticola* found in the Great Plains, at least in the south (Texas) and north (Saskatchewan).

**Figure 103.**
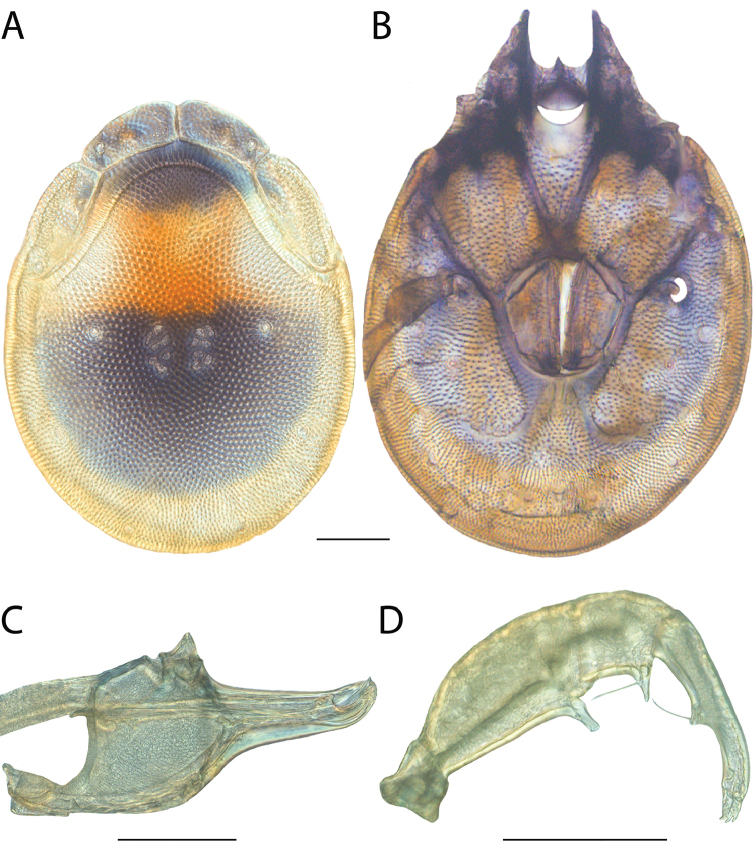
*Torrenticola irapalpa* sp. n. female: **A** dorsal plates **B** venter (legs removed) **C** subcapitulum **D** pedipalp (setae not accurately depicted). Scale = 100 µm.

**Figure 104.**
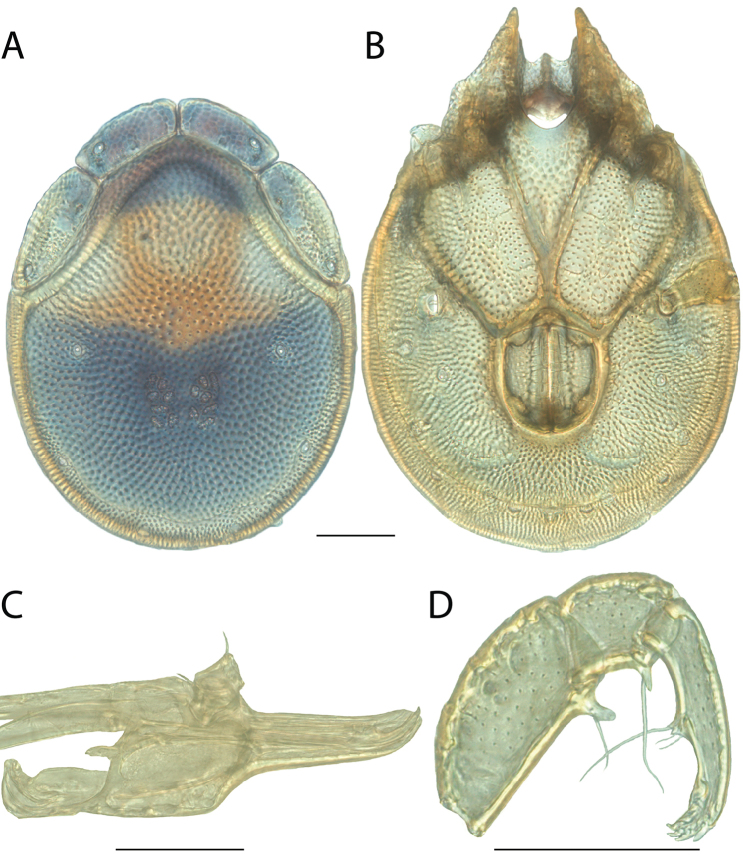
*Torrenticola irapalpa* sp. n. male: **A** dorsal plates **B** venter (legs removed) **C** subcapitulum **D** pedipalp (setae not accurately depicted). Scale = 100 µm.

######## Remarks.

*Torrenticola irapalpa* groups with other members of the Raptor Complex with high support and specimens of this species are less than 2% different in COI sequence from each other. In the combined analysis, *T. irapalpa* groups with *T. gnoma* with high support, but the position of this clade was not recovered. These species are greater than 9% from each other. Based upon overall similarity, distribution, and phylogenetic position, this species is placed within the Raptor Identification Group.

This species hypothesis is supported by low COI divergence within the species (0–2%) and high divergence between species (3–15%), and by the morphological characters outlined in the diagnosis.

####### Torrenticola ivyae

Taxon classificationAnimaliaTrombidiformesTorrenticolidae

Fisher & Dowling sp. n.

http://zoobank.org/74233707-73F0-4ECE-98F3-03FD7E55575A

######## Material examined.

HOLOTYPE (♀): from USA, Florida, Polk County, beside Rt. 471 at junction of Pasco, Polk and Sumter County lines, (28°19'19"N, 82°4'4"W), 24 April 1992, by IM Smith, IMS920003

PARATYPES (5 ♀; 5 ♂): **Florida, USA**: 2 ♀ from Hillsborough County, beside Rt. 41/301 near Hillsborough River State Park, south of Zephyrhills, (28°10'10"N, 82°12'12"W), 24 April 1992, by IM Smith, IMS920001 • 2 ♀ and 3 ♂ from Pasco County, beside Rt. 39 just north of Crystal Springs, (28°11'11"N, 82°10'10"W), 27 April 1992, by IM Smith, IMS920011 • 1 ♂ (ALLOTYPE) from Polk County, beside Rt. 471 at junction of Pasco, Polk and Sumter County lines, (28°19'19"N, 82°4'4"W), 24 April 1992, by IM Smith, IMS920003 • 1 ♀ and 1 ♂ from Polk County, beside Rucks Dairy Road 1.3 km south of Lake Arbuckle Road, (27°42'42"N, 81°26'26"W), 25 April 1992, by IM Smith, IMS920004

######## Type deposition.

Holotype (♀), allotype (♂), and some paratypes (3 ♀; 2 ♂) deposited in the CNC; other paratypes (2 ♀; 2 ♂) deposited in the ACUA.

######## Diagnosis.

*Torrenticola ivyae* are similar to other members of the Raptor Group (*T. gnoma*, *T. irapalpa*, *T. longitibia*, *T. mjolniri*, *T. elusiva*, *T. racupalpa*, *T. raptor*, *T. danielleae*, and *T. daemon*) in having round bodies; Dgl-4 close to muscles scars; long, thin subcapitular rostra; and long, thin pedipalp tibiae. *T. ivyae* can be differentiated from *T. gnoma*, *T. elusiva*, *T. danielleae*, and *T. daemon* by having a more elongate rostrum (length/width ♀ = 4.00–4.15 in *T. ivyae*, 2.74-3.75 in others; ♂ = 3.85–4.08 in *T. ivyae*, 2.56–3.57 in others) and more elongate pedipalpal tibiae (length/width ♀ = 5.07–5.64 in *T. ivyae*, 4.05–5.00 in others, ♂ = 4.75–5.20 in *T. ivyae*, 3.88–4.44 in others). *T. ivyae* can be differentiated from *T. irapalpa*, *T. raptor*, *T. danielleae*, and *T. daemon* by having Dgl-4 closer to the muscle scars (dorsum width/distance between Dgl-4 = 2.20–2.75 in *T. ivyae*, 1.42–2.09 in others). *T. ivyae* can be differentiated from *T. longitibia* (known only from males) by femur/genu (1.83–1.88 in *T. ivyae*, 2.1–2.17 in *T. longitibia*) and having less elongate tibiae (length/width = 4.75–5.20 in *T. ivyae*, 5.50–5.50 in *T. longitibia*). Female *T. ivyae* can be differentiated from female *T. racupalpa* by having a more elongate rostrum (length/width = 4.00–4.15 in *T. ivyae*, 3.56–3.82 in *T. racupalpa*) and more elongate pedipalpal tibiae (length/width = 5.07–5.64 in *T. ivyae*, 4.44–5.00 in *T. racupalpa*). Male *T. ivyae* can be differentiated from male *T. racupalpa* by having a longer anterior venter (♂ 220–230 in *T. ivyae*, 200–205 in *T. racupalpa*) and a shorter genital field (♂ 142–148 in *T. ivyae*, 160–165 in *T. racupalpa*). Female *T. ivyae* can be differentiated from female *T. mjolniri* by having a smaller dorsum (length ♀ = 550–590 in *T. ivyae*, 605–640 in *T. mjolniri*; width ♀ = 460–500 in *T. ivyae*, 510–545 in *T. mjolniri*) and a shorter anterior venter (♀ 155–170 in *T. ivyae*, 180–195 in *T. mjolniri*). Male *T. ivyae* can be differentiated from male *T. mjolniri* by having a less elongate subcapitulum (ventral length/width = 2.57–2.75 in *T. ivyae*, 2.82–3.00 in *T. mjolniri*). Additionally, *T. ivyae* can be differentiated from *T. mjolniri* by being found in Florida (*T. ivyae* is known from the northeast).

######## Description.

**Female (Figure 106)** (n = 5) (holotype measurements in parentheses when available) with characters of the genus with following specifications.

**Dorsum** — (550–590 (580) long; 460–500 (495) wide) circular with reddish-purple coloration anteriorly and posteriorly connected medially. Anterio-medial platelets (120–135 (120) long; 60–62.5 (62.5) wide). Anterio-lateral platelets (165–180 (165) long; 65–75 (75) wide) free from dorsal plate. Dgl-4 much closer to the muscle scars than to the edge of the dorsum (distance between Dgl-4 180–195 (180)). Dorsal plate proportions: dorsum length/width 1.16–1.20 (1.17); dorsal width/distance between Dgl-4 2.42–2.75 (2.75); anterio-medial platelet length/width 1.92–2.25 (1.92); anterio-lateral platelet length/width 2.20–2.62 (2.20); anterio-lateral/anterio-medial length 1.26–1.38 (1.38).

**Gnathosoma — Subcapitulum** (300–325 (325) long (ventral); 232.5–257.5 (247.5) long (dorsal); 115–127.5 (120) tall) colorless. Rostrum (130–145 (145) long; 32.5–35 (35) wide) elongate. Chelicerae (315–340 (335) long) with curved fangs (55–60 (60) long). Subcapitular proportions: ventral length/height 2.55–2.71 (2.71); rostrum length/width 4.00–4.15 (4.14). **Pedipalps** elongate with tuberculate ventral extensions on femora and genua. Palpomeres: trochanter (42.5–46.25 (45) long); femur (117.5–125 (125) long); genu (65–67.5 (65) long); tibia (91.25–100 (95) long; 17.5–18.75 (18.75) wide); tarsus (15–17.5 (17.5) long). Palpomere proportions: femur/genu 1.81–1.92 (1.92); tibia/femur 0.76–0.83 (0.76); tibia length/width 5.07–5.64 (5.07).

**Venter** — (630–755 (750) long; 490–540 (540) wide) with reddish-purple coloration. Gnathosomal bay (150–175 (165) long; 67.5–75 (72.5) wide). Cxgl-4 far from apex. **Medial suture** (25–25 (25) long). **Genital plates** (150–165 (157.5) long; 142.5–150 (150) wide). Additional measurements: Cx-1 (280–300 (300) long (total); 120–145 (145) long (medial)); Cx-3 (320–335 (325) wide); anterior venter (155–170 (170) long). Ventral proportions: gnathosomal bay length/width 2.00–2.44 (2.28); anterior venter/genital field length 0.94–1.13 (1.08); anterior venter length/genital field width 1.03–1.19 (1.13); anterior venter/medial suture 6.20–6.80 (6.80).

**Male (Figure 107)** (n = 4) (allotypic measurements in parentheses when available) with characters of the genus with following specifications.

**Dorsum** — (530–550 (550) long; 390–450 (420) wide) circular with reddish-purple coloration anteriorly and posteriorly connected medially. Anterio-medial platelets (125–135 (130) long; 55–62.5 (57.5) wide). Anterio-lateral platelets (160–190 (160) long; 60–75 (60) wide) free from dorsal plate. Dgl-4 much closer to the muscle scars than to the edge of the dorsum (distance between Dgl-4 165–205 (180)). Dorsal plate proportions: dorsum length/width 1.19–1.36 (1.31); dorsal width/distance between Dgl-4 2.20–2.39 (2.33); anterio-medial platelet length/width 2.00–2.45 (2.26); anterio-lateral platelet length/width 2.27–2.92 (2.67); anterio-lateral/anterio-medial length 1.23–1.46 (1.23).

**Gnathosoma — Subcapitulum** (295–302.5 (300) long (ventral); 220–230 (220) long (dorsal); 110–115 (110) tall) colorless. Rostrum (125–132.5 (130) long; 32.5–32.5 (32.5) wide) elongate. Chelicerae (295–305 (295) long) with curved fangs (45–50 (50) long). Subcapitular proportions: ventral length/height 2.57–2.75 (2.73); rostrum length/width 3.85–4.08 (4.00). **Pedipalps** elongate with tuberculate ventral extensions on femora and genua. Palpomeres: trochanter (40–42.5 (40) long); femur (110–112.5 (110) long); genu (60–60 (60) long); tibia (90–97.5 (90) long; 17.5–20 (17.5) wide); tarsus (15–16.25 (16.25) long). Palpomere proportions: femur/genu 1.83–1.88 (1.83); tibia/femur 0.82–0.87 (0.82); tibia length/width 4.75–5.20 (5.14).

**Venter** — (625–700 (700) long; 470–500 (470) wide) with reddish-purple coloration. Gnathosomal bay (140–150 (142.5) long; 62.5–72.5 (65) wide). Cxgl-4 far from apex. **Medial suture** (60–80 (75) long). **Genital plates** (142.5–147.5 (145) long; 112.5–125 (120) wide). Additional measurements: Cx-1 (280–290 (280) long (total); 125–145 (145) long (medial)); Cx-3 (310–330 (310) wide); anterior venter (220–230 (230) long). Ventral proportions: gnathosomal bay length/width 1.93–2.36 (2.19); anterior venter/genital field length 1.52–1.59 (1.59); anterior venter length/genital field width 1.76–2.00 (1.92); anterior venter/medial suture 2.75–3.67 (3.07).

**Immatures** unknown.

######## Etymology.

Specific epithet (*ivyae*) named in honor of Ivy Fisher, our (JRF and DMF) beautiful daughter, who was born in Florida, the type locality.

######## Distribution.

**Figure 105.**
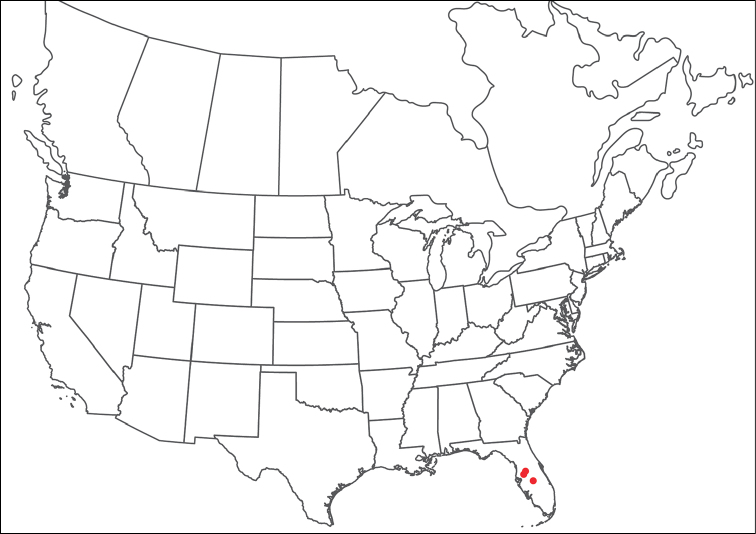
*Torrenticola ivyae* sp. n. distribution.

Florida (Figure 105).

**Figure 106.**
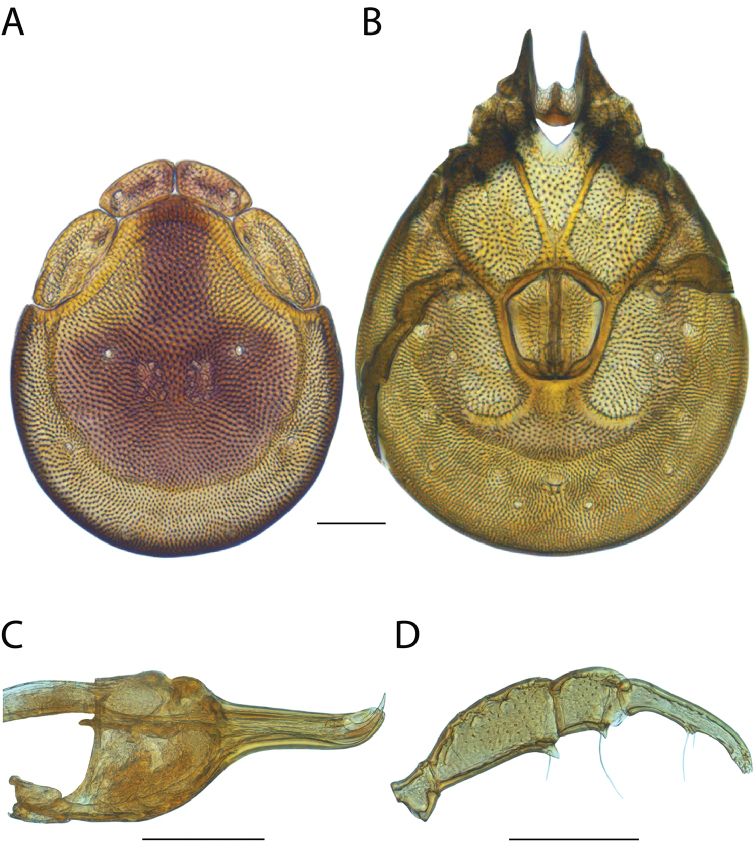
*Torrenticola ivyae* sp. n. female: **A** dorsal plates **B** venter (legs removed) **C** subcapitulum **D** pedipalp (setae not accurately depicted). Scale = 100 µm.

**Figure 107.**
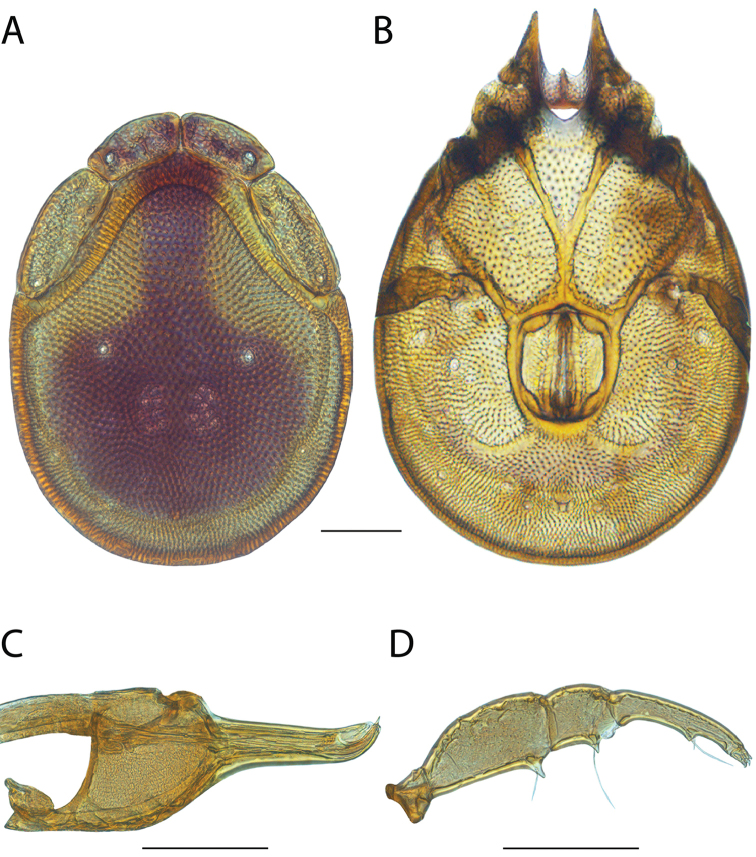
*Torrenticola ivyae* sp. n. male: **A** dorsal plates **B** venter (legs removed) **C** subcapitulum **D** pedipalp (setae not accurately depicted). Scale = 100 µm.

######## Remarks.

Unfortunately, we were unable to acquire fresh material of *Torrenticola ivyae* and therefore this species is not included in our phylogenetic analyses. However, we were able to examine morphology with material preserved in GAW. The overall similarity, elongate subcapitular rostra, elongate pedipalpal tibiae, and Dgl-4 close to the muscle scars, are consistent with placing this species in the Raptor Complex and Raptor Identification Group.

####### Torrenticola karambita

Taxon classificationAnimaliaTrombidiformesTorrenticolidae

Fisher & Dowling sp. n.

http://zoobank.org/240C5D4A-99BD-4BF1-9930-9D02B73479FC

######## Material examined.

HOLOTYPE (♀): from USA, Tennessee, Sevier County, Great Smokey Mountains National Park, Sugarlands Nature Trail (35°40'47"N, 83°31'51"W), 10 Sep 2010, by IM Smith, IMS100125, DNA 1758.

PARATYPES (1 ♀; 3 ♂): 1 ♂ (ALLOTYPE) from Sevier County, Great Smokey Mountains National Park, Sugarlands Nature Trail (35°40'47"N, 83°31'51"W), 10 Sep 2010, by IM Smith, IMS100125, DNA 1846 • 1 ♀ and 2 ♂ from Sevier County, Great Smokey Mountains National Park, Sugarlands Nature Trail (35°40'47"N, 83°31'51"W), 10 Sep 2010, by IM Smith, IMS100125

######## Type deposition.

Holotype (♀), allotype (♂), and some paratypes (1 ♂) deposited in the CNC; other paratypes (1 ♀; 1 ♂) deposited in the ACUA.

######## Diagnosis.

*Torrenticola karambita* is similar to other members of the Erectirostra Group (*T. erectirostra* and *T. robisoni*) in having an upturned rostrum that is wide when viewed ventrally. *T. karambita* can be differentiated from *T. erectirostra* and *T. robisoni* by lacking coloration (*T. erectirostra* has purplish dorsal coloration) and a slightly stockier rostrum (length/width ♀ = 1.57–1.62 in *T. karambita*, 1.72–2.09 in others; ♂ = 1.6–1.95 in *T. karambita*, 2.0–2.2 in *T. erectirostra*).

######## Description.

**Female (Figure 109)** (n = 2) (holotype measurements in parentheses when available) with characters of the genus with following specifications.

**Dorsum** — (690–725 (690) long; 490–500 (490) wide) ovoid and colorless. Anterio-medial platelets (145–162.5 (145) long; 65–75 (65) wide). Anterio-lateral platelets (217.5–220 (217.5) long; 80–82.5 (82.5) wide) free from dorsal plate. Dgl-4 much closer to the edge of the dorsum than to the muscle scars (distance between Dgl-4 340–360 (340)). Dorsal plate proportions: dorsum length/width 1.41–1.45 (1.41); dorsal width/distance between Dgl-4 1.39–1.44 (1.44); anterio-medial platelet length/width 2.17–2.23 (2.23); anterio-lateral platelet length/width 2.64–2.75 (2.64); anterio-lateral/anterio-medial length 1.35–1.50 (1.50).

**Gnathosoma — Subcapitulum** (325–350 (325) long (ventral); 229–254 (229) long (dorsal); 135–135 (135) tall) colorless. Rostrum (110–117.5 (110) long; 70–72.5 (70) wide) wide and unturned with dentation. Chelicerae (320–329 (321) long) with curved fangs (40–52 (41) long). Subcapitular proportions: ventral length/height 2.41–2.59 (2.41); rostrum length/width 1.57–1.62 (1.57). **Pedipalps** short and stocky (especially tibiae) with short tuberculate ventral extensions on femora and genua. Palpomeres: trochanter (52.5–55 (52.5) long); femur (101.25–105 (101.25) long); genu (65–67.5 (67.5) long); tibia (55–57.5 (55) long; 30–30 (30) wide); tarsus (17.5–17.5 (17.5) long). Palpomere proportions: femur/genu 1.50–1.62 (1.50); tibia/femur 0.54–0.55 (0.54); tibia length/width 1.83–1.92 (1.83).

**Venter** — (850–850 (850) long; 595–607 (607) wide) colorless. Gnathosomal bay (170–172.5 (172.5) long; 125–130 (130) wide). Cxgl-4 far from apex. **Medial suture** (15–17.5 (15) long). **Genital plates** (195–197.5 (195) long; 165–175 (165) wide). Additional measurements: Cx-1 (336–348 (336) long (total); 159–175 (174) long (medial)); Cx-3 (415–441 (415) wide); anterior venter (202.5–222.5 (202.5) long). Ventral proportions: gnathosomal bay length/width 1.33–1.36 (1.33); anterior venter/genital field length 1.04–1.13 (1.04); anterior venter length/genital field width 1.23–1.27 (1.23); anterior venter/medial suture 12.71–13.50 (13.50).

**Male (Figure 110)** (n = 3) (allotypic measurements in parentheses when available) with characters of the genus with following specifications.

**Dorsum**— (610–655 (610) long; 410–440 (420) wide) ovoid and colorless. Anterio-medial platelets (133.75–145 (133.75) long; 65–67.5 (65) wide). Anterio-lateral platelets (205–217.5 (207.5) long; 75–77.5 (75) wide) free from dorsal plate. Dgl-4 much closer to the edge of the dorsum than to the muscle scars (distance between Dgl-4 290–330 (290)). Dorsal plate proportions: dorsum length/width 1.44–1.60 (1.45); dorsal width/distance between Dgl-4 1.33–1.45 (1.45); anterio-medial platelet length/width 2.06–2.19 (2.06); anterio-lateral platelet length/width 2.65–2.81 (2.77); anterio-lateral/anterio-medial length 1.44–1.55 (1.55).

**Gnathosoma — Subcapitulum** (285–290 (290) long (ventral); 202–210 (202) long (dorsal); 108.75–115 (112.5) tall) colorless. Rostrum (97.5–102.5 (97.5) long; 52.5–60 (60) wide) wide and unturned with dentation. Chelicerae (268–269 (269) long) with curved fangs (43–52 (47) long). Subcapitular proportions: ventral length/height 2.48–2.67 (2.58); rostrum length/width 1.63–1.95 (1.63). **Pedipalps** short and stocky (especially tibiae) with short tuberculate ventral extensions on femora and genua. Palpomeres: trochanter (47.5–48.75 (47.5) long); femur (87.5–95 (95) long); genu (57.5–62.5 (62.5) long); tibia (55–57.5 (57.5) long; 27.5–27.5 (27.5) wide); tarsus (15–17.5 (17.5) long). Palpomere proportions: femur/genu 1.44–1.52 (1.52); tibia/femur 0.61–0.63 (0.61); tibia length/width 2.00–2.09 (2.09).

**Venter** — (760–785 (760) long; 490–521 (512) wide) colorless. Gnathosomal bay (165–175 (165) long; 105–107.5 (107.5) wide). Cxgl-4 far from apex. **Medial suture** (70–77.5 (77.5) long). **Genital plates** (156.25–167.5 (157.5) long; 120–125 (125) wide). Additional measurements: Cx-1 (284–320 (284) long (total); 115–154 (116) long (medial)); Cx-3 (366–373 (372) wide); anterior venter (247.5–248.75 (248.75) long). Ventral proportions: gnathosomal bay length/width 1.53–1.67 (1.53); anterior venter/genital field length 1.48–1.58 (1.58); anterior venter length/genital field width 1.99–2.06 (1.99); anterior venter/medial suture 3.19–3.54 (3.21).

**Immatures** unknown.

######## Etymology.

Specific epithet (*karambita*) refers to the upturned rostrum that has dentation on both sides in females, which resembles a karambit—small, recurved knives used in the Filipino martial arts practiced by JRF. The karambit is thought to have originated with the Minangkabau people of West Sumatra based upon a similarity to a tiger’s claws.

######## Distribution.

**Figure 108.**
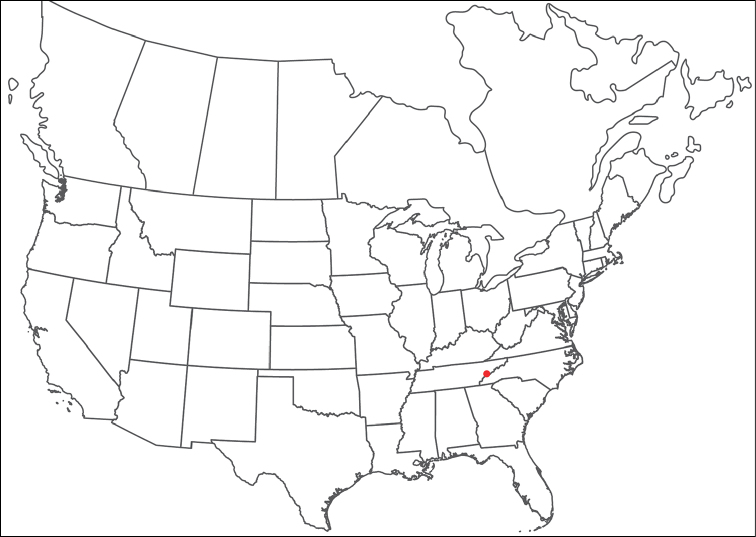
*Torrenticola karambita* sp. n. distribution.

Known only from Great Smokey Mountains National Park, Sevier County, Tennessee (Figure 108).

**Figure 109.**
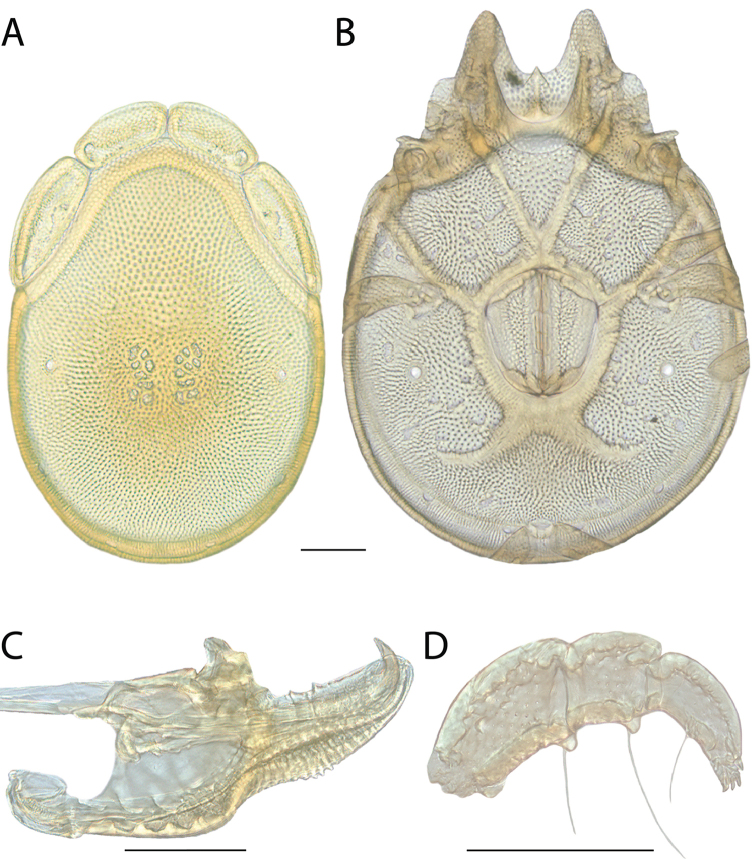
*Torrenticola karambita* sp. n. female: **A** dorsal plates **B** venter (legs removed) **C** subcapitulum **D** pedipalp (setae not accurately depicted). Scale = 100 µm.

**Figure 110.**
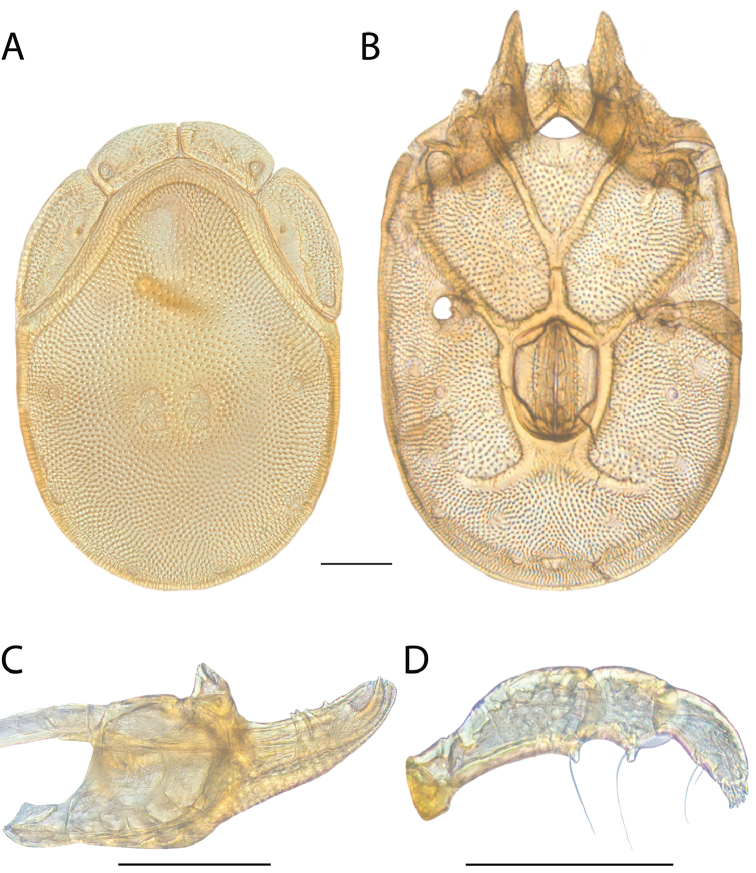
*Torrenticola karambita* sp. n. male: **A** dorsal plates **B** venter (legs removed) **C** subcapitulum **D** pedipalp (setae not accurately depicted). Scale = 100 µm.

######## Remarks.

*Torrenticola karambita* groups with other members of the Raptor Complex with high support in all analyses and specimens are less than 2% different in COI sequence from each other. In the combined analysis, *T. karambita* groups with two other species that share the modified subcapitulum with upturned rostrum: *T. erectirostra* and *T. robisoni*. These species are greater than 9% different from each other. Based upon overall similarity, distribution, and phylogenetic position, these species are placed within the Erectirostra Identification Group.

This species hypothesis is supported by low COI divergence within the species (0–2%), high divergence between species (3–15%), and the morphological characters outlined in the diagnosis.

####### Torrenticola keesdavidsi

Taxon classificationAnimaliaTrombidiformesTorrenticolidae

Cramer, 1992

Torrenticola keesdavidsi Cramer, 1992: 17.

######## Material examined

**(6** ♀; **10** ♂) **. Arizona, USA**: 1 ♀ and 1 ♂ from Cochise County, Chiricahua Mountains; beside Forest Road 42 near junction with Forest Road 42B, (31°55'55"N, 109°15'15"W), 16 July 1987, by IM Smith, IMS870093A • 1 ♀ and 1 ♂ from Cochise County, Chiricahua Mountains; Sycamore Campground east of Sunizona, (31°52'52"N, 109°20'20"W), 15 July 1987, by IM Smith, IMS870091 • 3 ♂ from Cochise County, Chiricahua Mountains; Cave Creek Recreation Area; John Hands Picnic Area off Forest Road 42A west of Portal, (31°53'53"N, 109°13'13"W), 15 July 1987, by IM Smith, IMS870092A • 1 ♀ and 1 ♂ from Coconino County, Oak Creek Canyon; beside Rt. 89A just north of Pine Flat Campground, (35°1'1"N, 111°44'44"W), 21 July 1987, by IM Smith, IMS870099A • 1 ♀ from Coconino County, Oak Creek Canyon; beside Rt. 89A between Bootlegger and Banjo Bill Campgrounds, (34°58'58"N, 111°45'45"W), 21 July 1987, by IM Smith, IMS870100B • 1 ♂ from Coconino County, Oak Creek Canyon; beside Rt. 89A just north of Pine Flat Campground, (35°1'1"N, 111°44'44"W), 21 July 1987, by IM Smith, IMS870099B • **New Mexico, USA**: 2 ♀ from Catron County, Glenwood; Whitewater Picnic Area 8 km east of Rt. 180, (33°22'22"N, 108°50'50"W), 12 July 1987, by IM Smith, IMS870084 • 2 ♂ from Catron County, beside Rt. 15, 65 km north of Rt. 180 (Silver City), (33°12'12"N, 108°13'13"W), 10 July 1987, by IM Smith, IMS870081A • 1 ♂ from Catron County, beside Rt. 15, 65 km north of Rt. 180 (Silver City), (33°12'12"N, 108°13'13"W), 10 July 1987, by IM Smith, IMS870081B.

######## Type deposition.

Holotype (♀) and allotype (♂) deposited in coll. Cristina Cramer, Instituto de Biología, UNAM.

######## Diagnosis.

*Torrenticola keesdavidsi* are similar to other members of the Rala Group (*T. rala*, *T. lamellipalpis*, *T. boettgeri*, *T. kurtvietsi*, *T. dolichodactyla*, and *T. anoplopalpa*) by being colorless, having incomplete hind coxal margins and being distributed in the southwest. *T. keesdavidsi* can be differentiated from all other Rala Group by having a shorter dorsum (length ♀ = 555–605 in *T. keesdavidsi*, 630–800 in others; ♂ = 500–590 in *T. keesdavidsi*, 600–780 in others) and dentate, flanged ventral extensions on the femora (others are lacking extensions, have tuberculate extensions, or flat, wide lamellate extensions). Additionally, *T. keesdavidsi* can be differentiated from all other Rala Group by having more elongate pedipalpal tibiae (length/width = 4.50–5.20 in *T. keesdavidsi*, 1.75–3.38 in others), except *T. lamellipalpis* (4.32–4.94).

######## Re-description.

**Female (Figure 112)** (n = 5) with characters of the genus with following specifications.

**Dorsum** — (555–605 long; 450–495 wide) circular and colorless. Anterio-medial platelets (135–137.5 long; 50–60 wide). Anterio-lateral platelets (180–192.5 long; 70–80 wide) free from dorsal plate. Dgl-4 closer to the edge of the dorsum than to the muscle scars (distance between Dgl-4 315–335). Dorsal plate proportions: dorsum length/width 1.21–1.31; dorsal width/distance between Dgl-4 1.41–1.48; anterio-medial platelet length/width 2.25–2.70; anterio-lateral platelet length/width 2.38–2.75; anterio-lateral/anterio-medial length 1.33–1.43.

**Gnathosoma — Subcapitulum** (312.5–327.5 long (ventral); 222.5–240 long (dorsal); 97.5–105 tall) colorless. Rostrum (120–130 long; 35–37.5 wide). Chelicerae (295–310 long) with curved fangs (45–50 long). Subcapitular proportions: ventral length/height 3.07–3.21; rostrum length/width 3.33–3.57. **Pedipalps** with dentate, flanged ventral extensions on femora and genua. Palpomeres: trochanter (35–37.5 long); femur (105–110 long); genu (55–57.5 long); tibia (90–95 long; 18.75–20 wide); tarsus (15–17.5 long). Palpomere proportions: femur/genu 1.91–2.00; tibia/femur 0.84–0.88; tibia length/width 4.50–4.93.

**Venter** — (695–755 long; 510–560 wide) colorless. Gnathosomal bay (145–150 long; 67.5–80 wide). Cxgl-4 far from apex. **Medial suture** (35–55 long). **Genital plates** (162.5–175 long; 152.5–160 wide). Additional measurements: Cx-1 (285–295 long (total); 140–150 long (medial)); Cx-3 (320–330 wide); anterior venter (192.5–222.5 long). Ventral proportions: gnathosomal bay length/width 1.88–2.19; anterior venter/genital field length 1.18–1.29; anterior venter length/genital field width 1.26–1.44; anterior venter/medial suture 4.05–5.50.

**Male (Figure 113)** (n = 5) with characters of the genus with following specifications.

**Dorsum** — (500–590 long; 410–450 wide) circular and colorless. Anterio-medial platelets (120–130 long; 55–60 wide). Anterio-lateral platelets (165–190 long; 65–75 wide) free from dorsal plate. Dgl-4 closer to the edge of the dorsum than to the muscle scars (distance between Dgl-4 280–320). Dorsal plate proportions: dorsum length/width 1.21–1.33; dorsal width/distance between Dgl-4 1.40–1.48; anterio-medial platelet length/width 2.09–2.27; anterio-lateral platelet length/width 2.33–2.71; anterio-lateral/anterio-medial length 1.32–1.48.

**Gnathosoma — Subcapitulum** (280–310 long (ventral); 202.5–230 long (dorsal); 85–105 tall) colorless. Rostrum (107.5–125 long; 32.5–35 wide). Chelicerae (260–297.5 long) with curved fangs (40–50 long). Subcapitular proportions: ventral length/height 2.81–3.29; rostrum length/width 3.29–3.57. **Pedipalps** with dentate, flanged ventral extensions on femora and genua. Palpomeres: trochanter (32.5–35 long); femur (97.5–110 long); genu (50–56.25 long); tibia (87.5–97.5 long; 17.5–20 wide); tarsus (15–17.5 long). Palpomere proportions: femur/genu 1.84–1.96; tibia/femur 0.86–0.95; tibia length/width 4.81–5.20.

**Venter** — (630–720 long; 460–510 wide) colorless. Gnathosomal bay (130–152.5 long; 60–75 wide). Cxgl-4 far from apex. **Medial suture** (80–95 long). **Genital plates** (140–155 long; 115–125 wide). Additional measurements: Cx-1 (260–280 long (total); 130–150 long (medial)); Cx-3 (280–340 wide); anterior venter (225–260 long). Ventral proportions: gnathosomal bay length/width 1.93–2.17; anterior venter/genital field length 1.55–1.86; anterior venter length/genital field width 1.96–2.17; anterior venter/medial suture 2.61–2.89.

**Immatures** unknown.

######## Etymology.

Cramer (1992) named this species in honor of Kees Davids “as an acknowledgement of his great contributions to acarology and aquatic ecology” (translated from Spanish).

######## Distribution.

**Figure 111.**
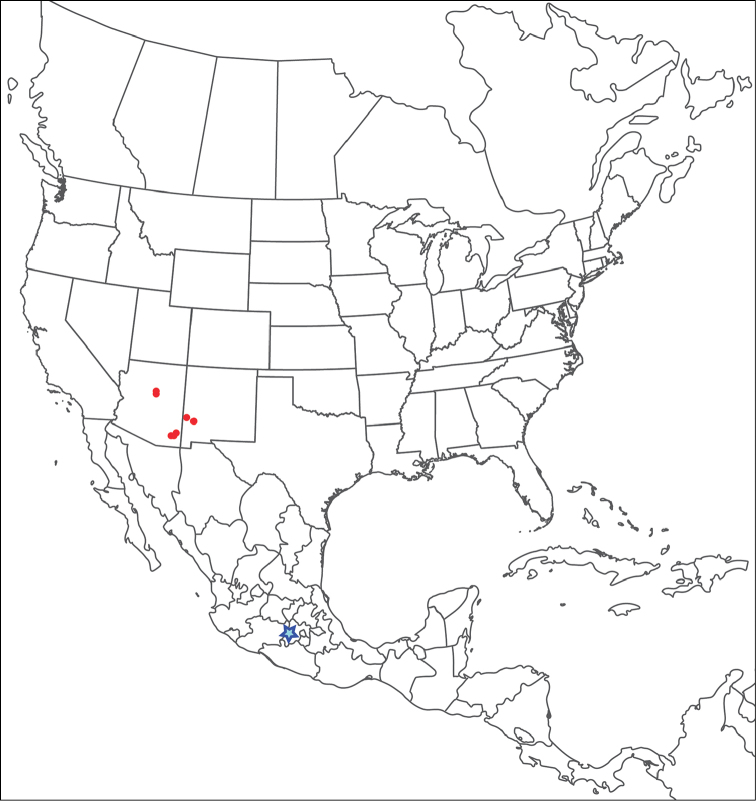
*Torrenticola keesdavidsi* distribution. Blue star represents type locality (Cramer 1992); and red circles represent new records and material examined.

Southwest (Arizona and New Mexico), extending southward into Mexico (Figure 111).

**Figure 112.**
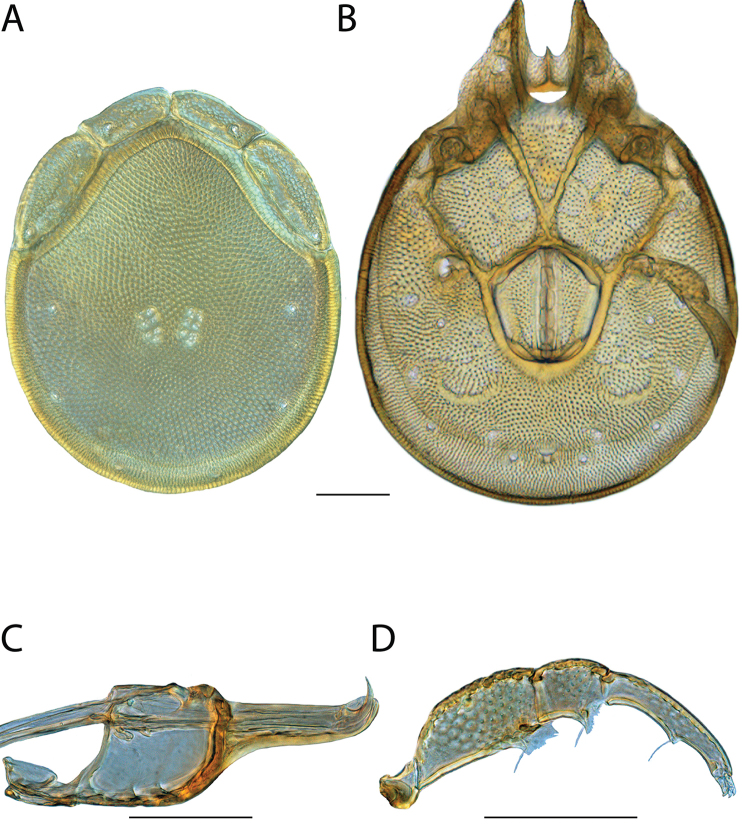
*Torrenticola keesdavidsi* female: **A** dorsal plates **B** venter (legs removed) **C** subcapitulum **D** pedipalp (setae not accurately depicted). Scale = 100 µm.

**Figure 113.**
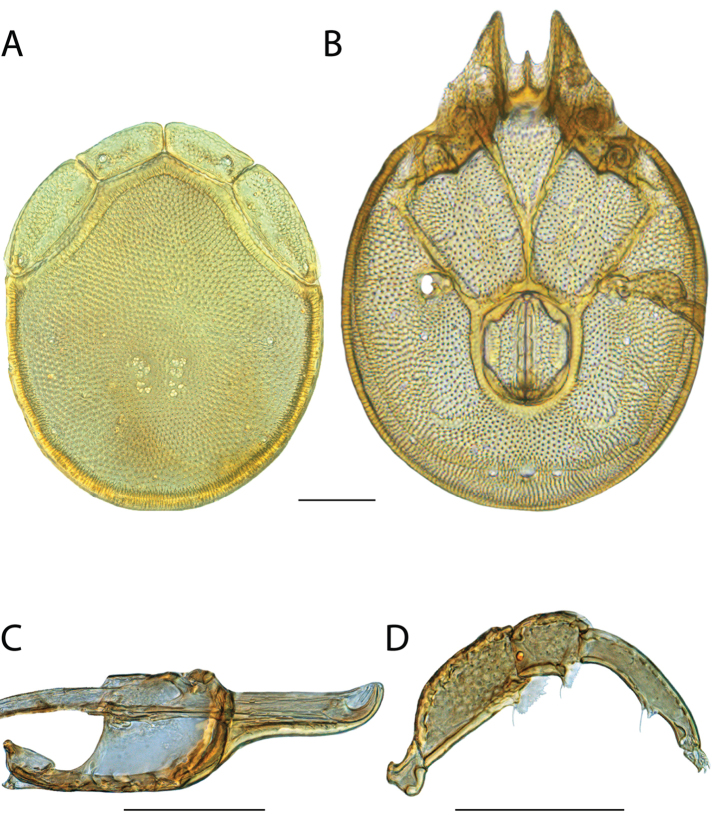
*Torrenticola keesdavidsi* male: **A** dorsal plates **B** venter (legs removed) **C** subcapitulum **D** pedipalp (setae not accurately depicted). Scale = 100 µm.

######## Remarks.

Unfortunately, we were unable to acquire fresh material of *Torrenticola keesdavidsi* and therefore this species is not included in our phylogenetic analyses. We were also unable to examine type material. However, we were able to examine new material from Arizona and New Mexico that fits well within the species hypothesis proposed by Cramer (1992). The overall appearance, incomplete hind coxal margins, distribution, and lack of color place this species in the Rala Group.

####### Torrenticola kittatinniana

Taxon classificationAnimaliaTrombidiformesTorrenticolidae

Habeeb, 1955

Torrenticola kittatinniana Habeeb, 1955: 2.

######## Material examined.

HOLOTYPE (♂): from USA, New Jersey, Sussex County, Little Flatbrook, north of Bevans, 12 Oct 1953, by H Habeeb, HH530110.

PARATYPES (1 ♀ and 0 ♂): **New Jersey, USA**: 1 ♀ (ALLOTYPE) from Morris County, Brook, Brookside, 20 May 1953, by H Habeeb, HH530045.

######## Type deposition.

Holotype (♀) and paratypes (1 ♀) deposited in the CNC.

######## Diagnosis.

*Torrenticola kittatinniana* are similar to other members of the Rusetria “4-Plate” group (*T. dunni*, *T. glomerabilis*, *T. pollani*, *T. rufoalba* and *T. shubini*) and *T. skvarlai* in having anterio-lateral platelets free from the dorsal plate, dorsal coloration separated into anterior and posterior portions, and indistinct hind coxal margins. Female *T. kittatinniana* can be differentiated from female *T. dunni* by having shorter pedipalpal genua (64 in *T. kittatinniana*, 70–75 in *T. dunni*); a shorter subcapitulum (ventral length = 310 in *T. kittatinniana*, 330–355 in *T. dunni*); and stockier anterio-medial platelets (length/width = 2.83 in *T. kittatinniana*, 2.33–2.54 in *T. dunni*). Male *T. kittatinniana* can be differentiated from male *T. dunni* by having a shorter anterior venter (235 in *T. kittatinniana*, 277–285 in *T. dunni*) and thinner dorsum (340 in *T. kittatinniana*, 350–370 in *T. dunni*). *T. kittatinniana* can be differentiated from *T. pollani* by having a stockier rostrum (length/width = 2.71–3.16 in *T. kittatinniana*, 3.27–3.82 in *T. pollani*) and stockier tibiae (length/width ♀ = 3.3 in *T. kittatinniana*, 3.8–4.2 in *T. pollani*; ♂ = 2.80 in *T. kittatinniana*, 3.4–3.8 in *T. pollani*). Female *T. kittatinniana* can be differentiated from female *T. shubini* by having a more elongate rostrum (length/width = 3.16 in *T. kittatinniana*, 2.5–2.7 in *T. shubini*) and a shorter subcapitulum (125 in *T. kittatinniana*, 140–145 in *T. shubini*). Male *T. kittatinniana* can be differentiated from male *T. shubini* by having a longer dorsum (500 in *T. kittatinniana*, 400–465 in *T. shubini*) and a longer genital field (115 in *T. kittatinniana*, 90–108 in *T. shubini*). *T. kittatinniana* can be differentiated from *T. glomerabilis* by having Dgl-4 closer to the dorsal edge (dorsal width/distance between Dgl-4 = 1.2–1.4 in *T. kittatinniana*, 1.5–1.7 in *T. glomerabilis*) and stockier tibiae (length/width ♀ = 3.3 in *T. kittatinniana*, 4.11–4.5 in *T. glomerabilis*; ♂ = 2.8 in *T. kittatinniana*, 3.5–4.4 in *T. glomerabilis*). *T. kittatinniana* can be differentiated from *T. rufoalba* by having a longer dorsum (♀ = 640 in *T. kittatinniana*, 550 in *T. rufoalba*; ♂ = 500 in *T. kittatinniana*, 440 in *T. rufoalba*) and more elongate anterio-medial platelets (length/width = 2.83–2.88 in *T. kittatinniana*, 2.45–2.61 in *T. rufoalba*). Additionally, male *T. kittatinniana* have a longer anterior venter (235 in *T. kittatinniana*, 195 in *T. rufoalba*). *T. kittatinniana* can be differentiated from *T. skvarlai* by having a conical pedipalpal femoral tubercle, whereas *T. skvarlai* has a broad and flat pedipalpal femoral tubercle, and by having a longer anterior venter (♀ = 165 in *T. kittatinniana*, 140–152.5 in *T. skvarlai*; ♂ = 235 in *T. kittatinniana*, 177.5–205 in *T. skvarlai*).

######## Re-description.

**Female (Figure 115)** (n = 1) (allotype only) with characters of the genus with following specifications.

**Dorsum** — (550 long; 400 wide) ovoid with purple coloration separated into anterior and posterior portions bordered with orange. Anterio-medial platelets (107.5 long; 41.25 wide). Anterio-lateral platelets (168.75 long; 55 wide) free from dorsal plate. Dgl-4 much closer to the edge of the dorsum than to the muscle scars (distance between Dgl-4 255). Dorsal plate proportions: dorsum length/width 1.38; dorsal width/distance between Dgl-4 1.57; anterio-medial platelet length/width 2.61; anterio-lateral platelet length/width 3.07; anterio-lateral/anterio-medial length 1.57.

**Gnathosoma— Subcapitulum** (310 long (ventral); 235 long (dorsal); 127.5 tall) colorless. Rostrum (130 long; 42.5 wide). Chelicerae (315 long) with curved fangs (62.5 long). Subcapitular proportions: ventral length/height 2.43; rostrum length/width 3.06. **Pedipalps** with tuberculate ventral extensions on femora and genua. Palpomeres: trochanter (42.5 long); femur (115 long); genu (65 long); tibia (87.5 long; 25 wide); tarsus (17.5 long). Palpomere proportions: femur/genu 1.77; tibia/femur 0.76; tibia length/width 3.50.

**Venter** — (640 long; 450 wide) mostly colorless with faint purple coloration in areas surrounding coxae. Gnathosomal bay (142.5 long; 92.5 wide). Cxgl-4 subapical. **Medial suture** (17.5 long). **Genital plates** (167.5 long; 155 wide). Additional measurements: Cx-1 (125 long (total); 125 long (medial)); Cx-3 (335 wide); anterior venter (155 long). Ventral proportions: gnathosomal bay length/width 1.54; anterior venter/genital field length 0.93; anterior venter length/genital field width 1.00; anterior venter/medial suture 8.86.

**Male (Figure 116)** (n = 1) (holotype only) with characters of the genus with following specifications.

**Dorsum** — (500 long; 340 wide) ovoid with purple coloration separated into anterior and posterior portions bordered with orange. Anterio-medial platelets (90 long; 31.25 wide). Anterio-lateral platelets (140 long; 47.5 wide) free from dorsal plate. Dgl-4 much closer to the edge of the dorsum than to the muscle scars (distance between Dgl-4 280). Dorsal plate proportions: dorsum length/width 1.47; dorsal width/distance between Dgl-4 1.21; anterio-medial platelet length/width 2.88; anterio-lateral platelet length/width 2.95; anterio-lateral/anterio-medial length 1.56.

**Gnathosoma — Subcapitulum** (237.5 long (ventral); 180 long (dorsal); 100 tall) colorless. Rostrum (95 long; 35 wide). Chelicerae (222.5 long) with curved fangs (45 long). Subcapitular proportions: ventral length/height 2.38; rostrum length/width 2.71. **Pedipalps** with tuberculate ventral extensions on femora and genua. Palpomeres: trochanter (37.5 long); femur (87.5 long); genu (57.5 long); tibia (70 long; 25 wide); tarsus (17.5 long). Palpomere proportions: femur/genu 1.52; tibia/femur 0.80; tibia length/width 2.80.

**Venter** — (600 long; 435 wide) mostly colorless with faint purple coloration in areas surrounding coxae. Gnathosomal bay (107.5 long; 72.5) wide). Cxgl-4 subapical. **Medial suture** (102.5 long). **Genital plates** (115 long; 82.5 wide). Additional measurements: Cx-1 (235 long (total); 125 long (medial)); Cx-3 (285 wide); anterior venter (235 long). Ventral proportions: gnathosomal bay length/width 1.48; anterior venter/genital field length 2.04; anterior venter length/genital field width 2.85; anterior venter/medial suture 2.29.

**Immatures** unknown.

######## Etymology.

Habeeb (1955) did not specify an explanation for the specific epithet (*kittatinniana*). However, we suspect it refers to the type locality, which is located in the Great Kittatinny Valley, which is named for Kittatinny Mountain in northwestern New Jersey, a northern extension of the Appalachian Ridge and Valley province.

######## Distribution.

**Figure 114.**
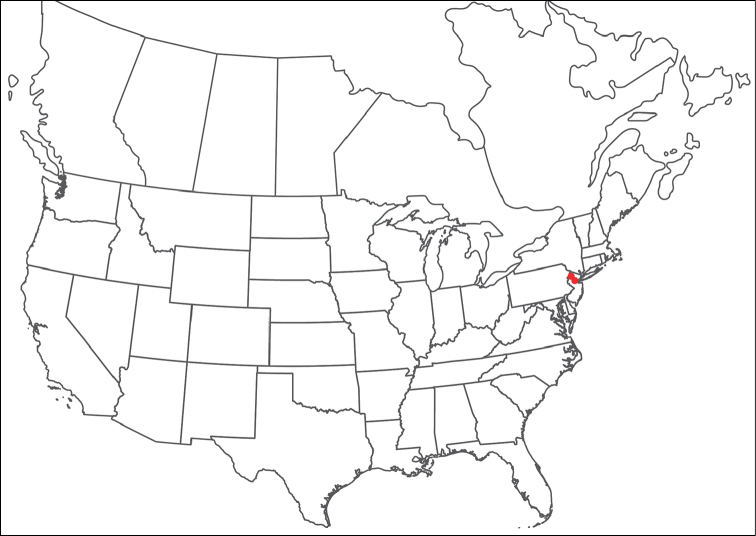
*Torrenticola kittatinniana* distribution.

Northern New Jersey (Figure 114).

**Figure 115.**
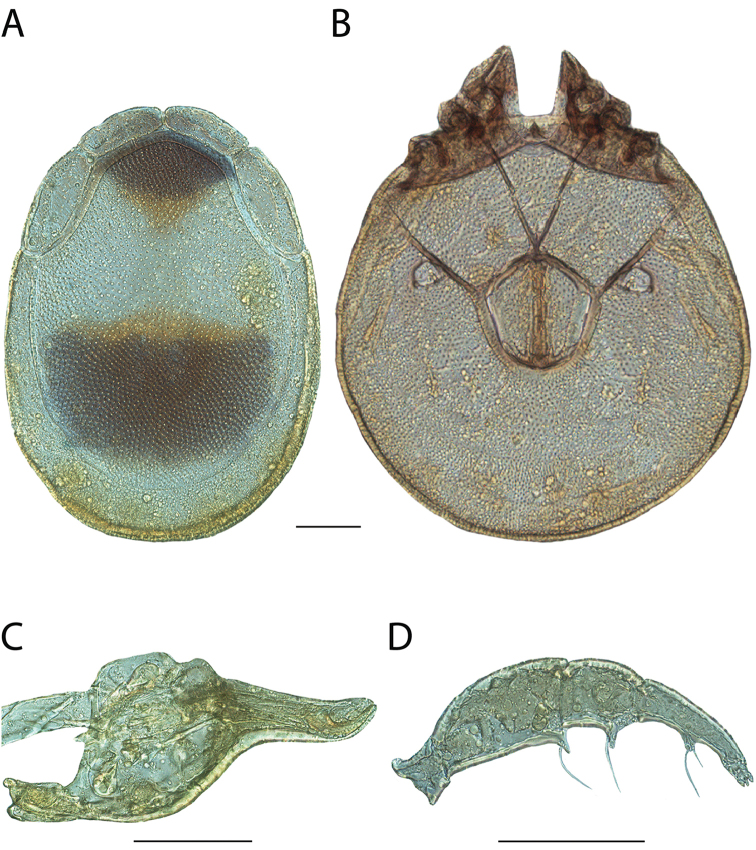
*Torrenticola kittatinniana* female: **A** dorsal plates **B** venter (legs removed) **C** subcapitulum **D** pedipalp (setae not accurately depicted). Scale = 100 µm.

**Figure 116.**
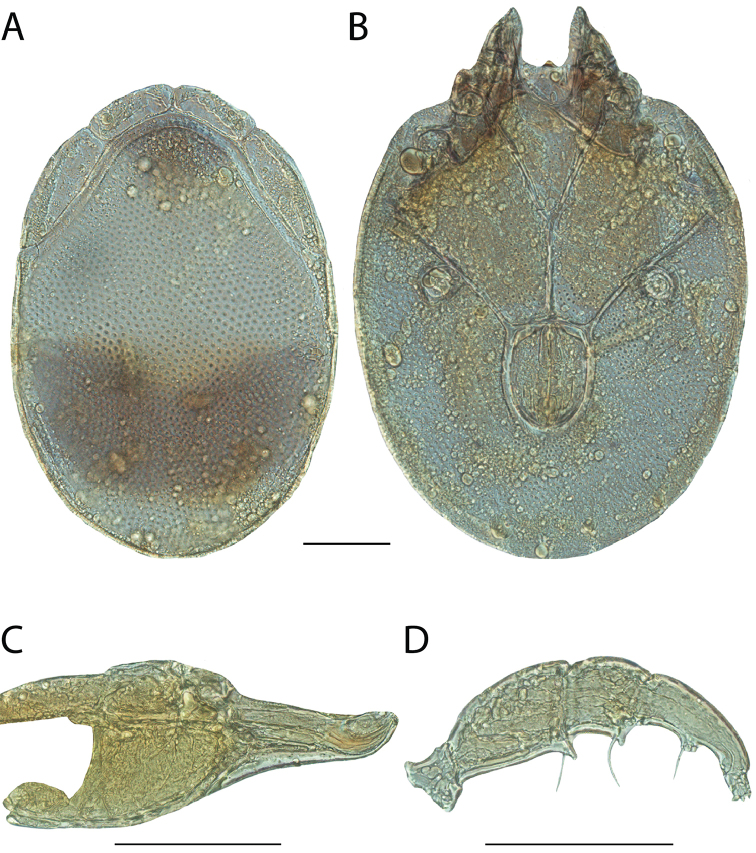
*Torrenticola kittatinniana* male: **A** dorsal plates **B** venter (legs removed) **C** subcapitulum **D** pedipalp (setae not accurately depicted). Scale = 100 µm.

######## Remarks.

Unfortunately, we were unable to acquire more specimens of *Torrenticola kittatinniana* and therefore this species is not included in our phylogenetic analyses. We were able to examine the type specimens. Based upon overall similarity, fusion of the posterio-lateral platelets with the dorsal plate, and distribution, this species clearly groups with other members of the Rusetria Complex and we are able to place it within the Eastern 4-Plate Identification Group.

####### Torrenticola kringi

Taxon classificationAnimaliaTrombidiformesTorrenticolidae

Fisher & Dowling sp. n.

http://zoobank.org/C5724C7A-F8A6-4E6F-9F34-8B08DB3E99DB

######## Material examined.

HOLOTYPE (♀): from USA, Texas, Tyler County, Spurger; beside Farm Road 1013, 8.2 km west of Rt. 92, (30°41'41"N, 94°15'15"W), 30 September 1994, by IM Smith, IMS940027A.

PARATYPES (4 ♀; 5 ♂): **Texas, USA**: 1 ♂ (ALLOTYPE) from Tyler County, Spurger; beside Farm Road 1013, 8.2 km west of Rt. 92, (30°41'41"N, 94°15'15"W), 30 September 1994, by IM Smith, IMS940027A • 4 ♀ and 4 ♂ from Tyler, Spurger; beside Farm Road 1013, 8.2 km west of Rt. 92, (30°41'41"N, 94°15'15"W), 30 September 1994, by IM Smith, IMS940027A.

######## Type deposition.

Holotype (♀), allotype (♂), and some paratypes (2 ♀; 2 ♂) deposited in the CNC; other paratypes (2 ♀; 2 ♂) deposited in the ACUA.

######## Diagnosis.

*Torrenticola kringi* are similar to other members of the Tricolor Complex (*T. bittikoferae*, *T. hoosieri*, *T. larvata*, *T. pearsoni*, *T. olliei*, *T. sierrensis*, *T. tricolor*, *T. trimaculata*, *T. unimaculata*, *T. cardia*, *T. dimorpha*, and *T. mohawk*) in having a short, conical rostrum. *T. kringi* can be differentiated from most *Torrenticola*, including other members of the Tricolor Complex, by having a distinct dorsal pattern of a large anterior dorsal spot. The only other species with this pattern is *T. ululata*, which, like all Rusetria 2-Plates, have anterio-lateral platelets fused to the dorsal plate and *T. unimaculata*, which has a stockier rostrum (length/width = 2.6–3.2 in *T. kringi*, 1.9–2.2 in *T. unimaculata*). *T. kringi* are most similar to other members of the Tricolor Complex that have bold patterning (*T. larvata*, *T. tricolor*, *T. trimaculata*, *T. unimaculata*, *T. cardia*, and *T. mohawk*). *T. kringi* can be further differentiated from all other members of the Tricolor Complex by having a more elongate rostrum (length/width = 2.67–3.13 in *T. kringi*, 1.29–2.59).

######## Description.

**Female (Figure 118)** (n = 5) (holotype measurements in parentheses when available) with characters of the genus with following specifications.

**Dorsum** — (550–580 (580) long; 460–500 (490) wide) circular with coloration restricted to a single dark spot anteriorly (occasionally extending medially), with an orange spot posterior to the dark spot. Anterio-medial platelets (125–145 (145) long; 60–70 (70) wide). Anterio-lateral platelets (160–185 (185) long; 65–75 (75) wide) free to dorsal plate. Dgl-4 much closer to the edge of the dorsum than to the muscle scars (distance between Dgl-4 335–365 (350)). Dorsal plate proportions: dorsum length/width 1.15–1.20 (1.18); dorsal width/distance between Dgl-4 1.26–1.46 (1.40); anterio-medial platelet length/width 2.00–2.25 (2.07); anterio-lateral platelet length/width 2.29–2.69 (2.47); anterio-lateral/anterio-medial length 1.19–1.40 (1.28).

**Gnathosoma — Subcapitulum** (290–302.5 (300) long (ventral); 215–227.5 (227.5) long (dorsal); 117.5–125 (125) tall) colorless. Rostrum (120–130 (125) long; 40–45 (42.5) wide). Chelicerae (275–300 (290) long) with curved fangs (50–55 (50) long). Subcapitular proportions: ventral length/height 2.34–2.47 (2.40); rostrum length/width 2.67–3.13 (2.94). **Pedipalps** with tuberculate ventral extensions on femora and genua. Palpomeres: trochanter (40–42.5 (41.25) long); femur (107.5–112.5 (112.5) long); genu (72.5–77.5 (77.5) long); tibia (91.25–97.5 (96.25) long; 27.5–28.75 (28.75) wide); tarsus (25–26.25 (25) long). Palpomere proportions: femur/genu 1.43–1.48 (1.45); tibia/femur 0.85–0.89 (0.86); tibia length/width 3.32–3.55 (3.35).

**Venter** — (630–690 (680) long; 500–530 (525) wide) colorless. Gnathosomal bay (132.5–152.5 (145) long; 75–82.5 (82.5) wide). Cxgl-4 subapical. **Medial suture** (22.5–30 (30) long). **Genital plates** (165–180 (180) long; 150–160 (160) wide). Additional measurements: Cx-1 (240–270 (270) long (total); 110–130 (130) long (medial)); Cx-3 (320–345 (345) wide); anterior venter (145–170 (170) long). Ventral proportions: gnathosomal bay length/width 1.67–1.94 (1.76); anterior venter/genital field length 0.85–0.97 (0.94); anterior venter length/genital field width 0.92–1.07 (1.06); anterior venter/medial suture 5.17–6.44 (5.67).

**Male (Figure 119)** (n = 5) (allotypic measurements in parentheses when available) with characters of the genus with following specifications.

**Dorsum** — (480–560 (510) long; 395–465 (420) wide) circular with coloration restricted to a single dark spot anteriorly (occasionally extending medially), with an orange spot posterior to the dark spot. Anterio-medial platelets (110–130 (117.5) long; 52.5–65 (57.5) wide). Anterio-lateral platelets (140–170 (150) long; 50–75 (60) wide) free from dorsal plate. Dgl-4 much closer to the edge of the dorsum than to the muscle scars (distance between Dgl-4 285–350 (315)). Dorsal plate proportions: dorsum length/width 1.19–1.30 (1.21); dorsal width/distance between Dgl-4 1.29–1.39 (1.33); anterio-medial platelet length/width 1.96–2.27 (2.04); anterio-lateral platelet length/width 2.27–2.80 (2.50); anterio-lateral/anterio-medial length 1.27–1.31 (1.28).

**Gnathosoma — Subcapitulum** (240–275 (260) long (ventral); 177.5–210 (195) long (dorsal); 90–105 (100) tall) colorless. Rostrum (100–115 (110) long; 35–40 (37.5) wide). Chelicerae (225–270 (260) long) with curved fangs (45–50 (50) long). Subcapitular proportions: ventral length/height 2.55–2.75 (2.60); rostrum length/width 2.81–2.93 (2.93). **Pedipalps** with tuberculate ventral extensions on femora and genua. Palpomeres: trochanter (35–38.75 (37.5) long); femur (85–101.25 (97.5) long); genu (60–71.25 (67.5) long); tibia (70–82.5 (77.5) long; 23.75–27.5 (26.25) wide); tarsus (22.5–25 (23.75) long). Palpomere proportions: femur/genu 1.36–1.44 (1.44); tibia/femur 0.79–0.87 (0.79); tibia length/width 2.95–3.14 (2.95).

**Venter** — (590–695 (640) long; 440–510 (470) colorless. Gnathosomal bay (100–130 (115) long; 62.5–77.5 (75) wide). Cxgl-4 subapical. **Medial suture** (85–100 (85) long). **Genital plates** (115–130 (125) long; 95–105 (100) wide). Additional measurements: Cx-1 (220–270 (250) long (total); 120–150 (145) long (medial)); Cx-3 (275–325 (305) wide); anterior venter (220–255 (240) long). Ventral proportions: gnathosomal bay length/width 1.53–1.79 (1.53); anterior venter/genital field length 1.87–1.96 (1.92); anterior venter length/genital field width 2.32–2.43 (2.40); anterior venter/medial suture 2.44–2.83 (2.82).

**Immatures** unknown.

######## Etymology.

Specific epithet (*kringi*) named in honor of Tim Kring, Department Head of Entomology at Virginia Tech, who was a member of JRF’s Ph.D. committee. His friendship, humor, advice, and student-focused approach has been a great influence to JRF.

######## Distribution.

**Figure 117.**
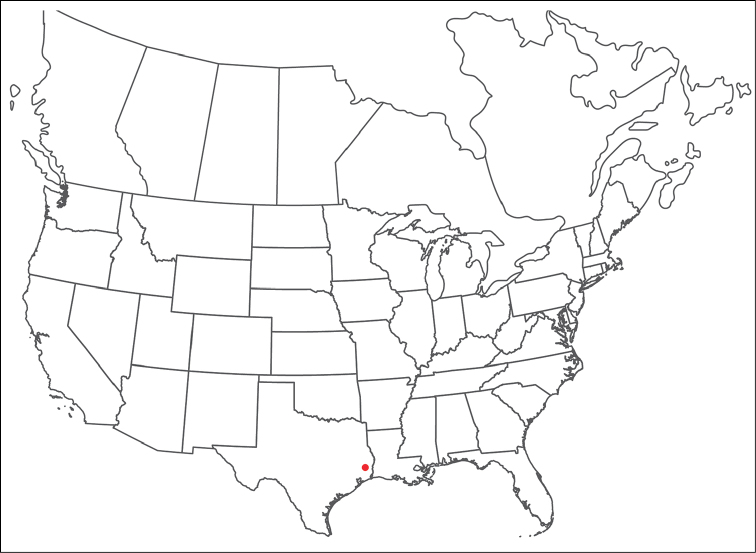
*Torrenticola kringi* sp. n. distribution.

Eastern Texas (Figure 117).

**Figure 118.**
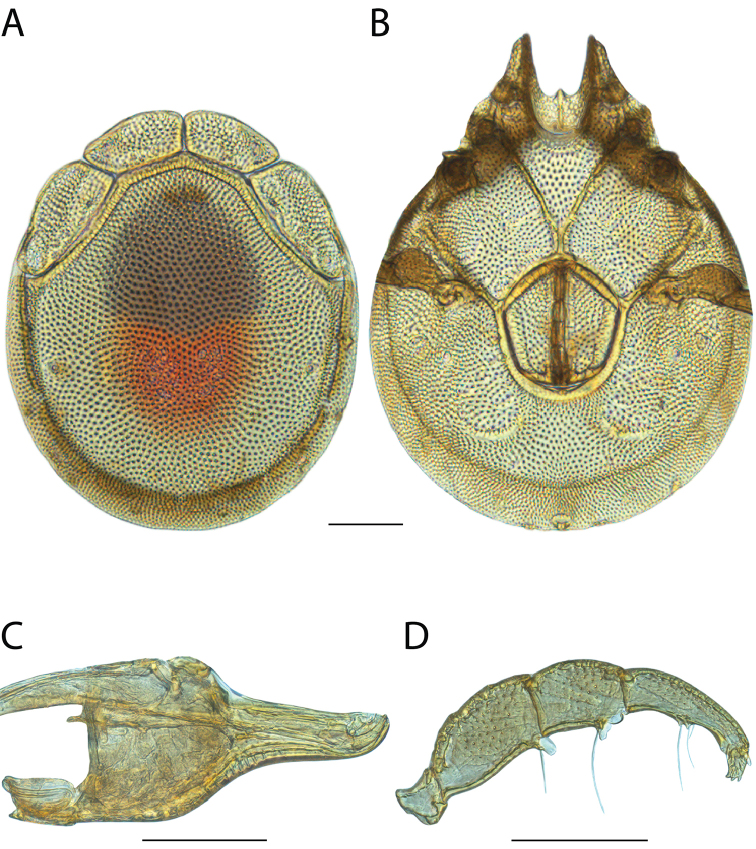
*Torrenticola kringi* sp. n. female: **A** dorsal plates **B** venter (legs removed) **C** subcapitulum **D** pedipalp (setae not accurately depicted). Scale = 100 µm.

**Figure 119.**
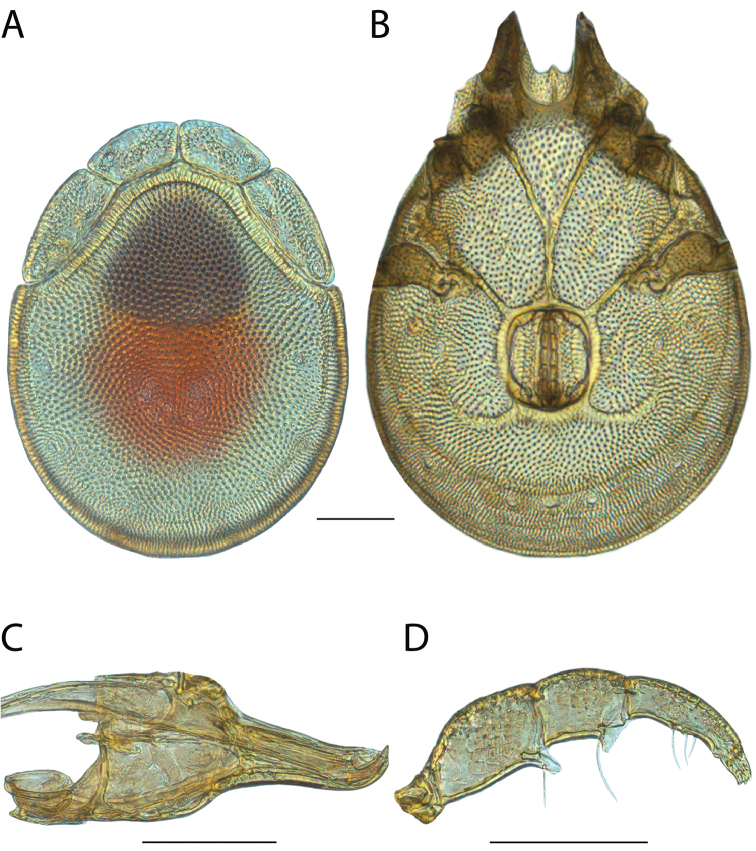
*Torrenticola kringi* sp. n. male: **A** dorsal plates **B** venter (legs removed) **C** subcapitulum **D** pedipalp (setae not accurately depicted). Scale = 100 µm.

######## Remarks.

Unfortunately, we were unable to acquire fresh material of *Torrenticola kringi* and therefore this species is not included in our phylogenetic analyses. However, we were able to examine morphology with material preserved in GAW. The overall similarity, conical rostrum that is downturned in males, and distribution, are consistent with placing this species in the Tricolor Complex and among eastern members of the Tricolor Identification Group.

####### Torrenticola kurtvietsi

Taxon classificationAnimaliaTrombidiformesTorrenticolidae

Cramer, 1992

Torrenticola kurtvietsi Cramer, 1992: 24.

######## Material examined

**(9** ♀; **3** ♂) **. Arizona, USA**: 1 ♀ and 1 ♂ from Cochise County, Chiricahua Mountains; Cave Creek Recreation Area; John Hands Picnic Area off Forest Road 42A west of Portal, (31°53'53"N, 109°13'13"W), 15 July 1987, by IM Smith, IMS870092A • 2 ♀ from Cochise County, Chiricahua Mountains; Cave Creek Recreation Area; John Hands Picnic Area off Forest Road 42A west of Portal, (31°53'53"N, 109°13'13"W), 15 July 1987, by IM Smith, IMS870092B • 1 ♀ and 1 ♂ from Cochise County, Chiricahua Mountains; beside Forest Road 42 near junction with Forest Road 42B, (31°55'55"N, 109°15'15"W), 16 July 1987, by IM Smith, IMS870093A • 2 ♀ and 1 ♂ from Cochise County, Chiricahua Mountains; Cave Creek Recreation Area; Stewart Campground beside Forest Road 42 west of Portal, (31°53'53"N, 109°10'10"W), 16 July 1987, by IM Smith, IMS870094 • 1 ♀ and 1 ♂ from Cochise County, Chiricahua Mountains; Sycamore Campground east of Sunizona, (31°52'52"N, 109°20'20"W), 15 July 1987, by IM Smith, IMS870091 • **Mexico, Mexico**: 1 ♀ from beside Hwy. 134 at Km 29, (19°59'59"N, 103°30'30"W), 7 May 1985, by IM Smith & C Cramer, IMS850138 • 1 ♀ from beside Hwy. 134 at Km 30, (21°0'0"N, 101°51'51"W), 14 May 1985, by IM Smith & C Cramer, IMS850139

######## Type deposition.

Holotype (♀), and allotype (♂), deposited in Cristina Cramer’s collection at the Instituto de Biología, UNAM.

######## Diagnosis.

*Torrenticola kurtvietsi* are similar to other members of the Rala Group (*T. rala*, *T. keesdavidsi*, *T. boettgeri*, *T. lamellipalpis*, *T. dolichodactyla*, and *T. anoplopalpa*) by being colorless, having incomplete hind coxal margins and being distributed in the southwest. *T. kurtvietsi* can be differentiated from all other Rala Group by having a more elongate gnathosomal bay (length/width = 4.47–5.43 in *T. kurtvietsi*, 1.48–2.73 in others), except *T. boettgeri* (3.85–5.00); and stockier pedipalpal tibiae (length/width = 1.75–2.00 in *T. kurtvietsi*, 2.71–5.20 in others), except *T. boettgeri* (1.86–2.14). *T. kurtvietsi* can be differentiated from *T. boettgeri* by having a less elongate dorsum (length/width = 1.29–1.42 in *T. kurtvietsi*, 1.74–1.82 in *T. boettgeri*) and a more elongate subcapitulum (ventral length/width = 2.51–2.70 in *T. kurtvietsi*, 1.96–2.07 in *T. boettgeri*).

######## Re-description.

**Female (Figure 121)** (n = 5) with characters of the genus with following specifications.

**Dorsum** — (680–730 long; 510–560 wide) circular with light pink coloration without a distinct pattern or colorless. Anterio-medial platelets (135–150 long; 60–70 wide). Anterio-lateral platelets (185–215 long; 75–80 wide) free from dorsal plate. Dgl-4 closer to the edge of the dorsum than to the muscle scars (distance between Dgl-4 360–410). Dorsal plate proportions: dorsum length/width 1.29–1.34; dorsal width/distance between Dgl-4 1.34–1.44; anterio-medial platelet length/width 2.07–2.25; anterio-lateral platelet length/width 2.47–2.80; anterio-lateral/anterio-medial length 1.33–1.48.

**Gnathosoma — Subcapitulum** (310–332.5 long (ventral); 205–230 long (dorsal); 115–132.5 tall) colorless. Rostrum (105–112.5 long; 35–35 wide). Chelicerae (325–360 long) with curved fangs (45–55 long). Subcapitular proportions: ventral length/height 2.51–2.70; rostrum length/width 3.00–3.21. **Pedipalps** short and stocky (especially tibiae) without extensions on femora and genua. Palpomeres: trochanter (30–35 long); femur 78.75–87.5 long); genu (57.5–62.5 long); tibia (35–40 long; 20–20 wide); tarsus (12.5–15 long). Palpomere proportions: femur/genu 1.37–1.48; tibia/femur 0.41–0.47; tibia length/width 1.75–2.00.

**Venter** — (840–915 long; 610–650 wide) colorless. Gnathosomal bay (180–205 long; 35–42.5 wide). Cxgl-4 apical. **Medial suture** (40–47.5 long). **Genital plates** (185–195 long; 165–177.5 wide). Additional measurements: Cx-1 (340–360 long (total); 150–180 long (medial)); Cx-3 (370–390 wide); anterior venter (210–225 long). Ventral proportions: gnathosomal bay length/width 4.75–5.43; anterior venter/genital field length 1.08–1.18; anterior venter length/genital field width 1.18–1.33; anterior venter/medial suture 4.53–5.25.

**Male (Figure 122)** (n = 4) with characters of the genus with following specifications.

**Dorsum** — (610–650 long; 450–470 wide) circular with light pink coloration without a distinct pattern or colorless. Anterio-medial platelets (140–150 long; 62.5–65 wide). Anterio-lateral platelets (195–205 long; 70–75 wide) free from dorsal plate. Dgl-4 much closer to the edge of the dorsum than to the muscle scars (distance between Dgl-4 345–360). Dorsal plate proportions: dorsum length/width 1.31–1.42; dorsal width/distance between Dgl-4 1.30–1.35; anterio-medial platelet length/width 2.19–2.31; anterio-lateral platelet length/width 2.67–2.79; anterio-lateral/anterio-medial length 1.33–1.46.

**Gnathosoma — Subcapitulum** (297.5–310 long (ventral); 205–215 long (dorsal); 112.5–115 tall) colorless. Rostrum (100–107.5 long; 35–35 wide). Chelicerae (325–355 long) with curved fangs (50–50 long). Subcapitular proportions: ventral length/height 2.64–2.70; rostrum length/width 2.86–3.07. **Pedipalps** short and stocky (especially tibiae) without extensions on femora and genua. Palpomeres: trochanter (27.5–35 long); femur (75–82.5 long); genu (57.5–60 long); tibia (36.25–38.75 long; 18.75–21.25 wide); tarsus (10–15 long). Palpomere proportions: femur/genu 1.29–1.43; tibia/femur 0.45–0.48; tibia length/width 1.81–2.00.

**Venter** — (785–815 long; 530–540 wide) colorless. Gnathosomal bay (185–195 long; 40–42.5 wide). Cxgl-4 apical. **Medial suture** (100–120 long). **Genital plates** (175–185 long; 135–140 wide). Additional measurements: Cx-1 (340–350 long (total); 150–170 long (medial)); Cx-3 (360–370 wide); anterior venter (287.5–307.5 long). Ventral proportions: gnathosomal bay length/width 4.47–4.81; anterior venter/genital field length 1.58–1.71; anterior venter length/genital field width 135–140; anterior venter/medial suture 2.40–3.00.

**Immatures** unknown.

######## Etymology.

Cramer (1992) named this species in honor of Kurt O. Viets “in recognition of his great contribution to the knowledge of aquatic mites and especially for his invaluable efforts in bringing together the world catalog on this subject” (translated from Spanish).

######## Distribution.

**Figure 120.**
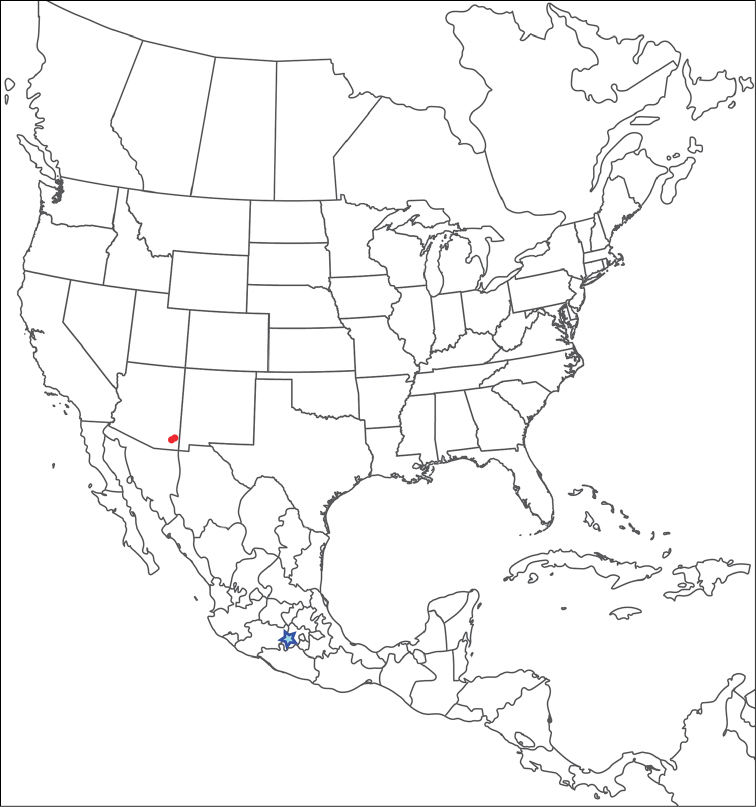
*Torrenticola kurtvietsi* distribution. Blue star represents type locality (Cramer 1992); red circles represent new record and material examined.

Southeastern Arizona (probably also western New Mexico), extending south into Mexico (Figure 120).

**Figure 121.**
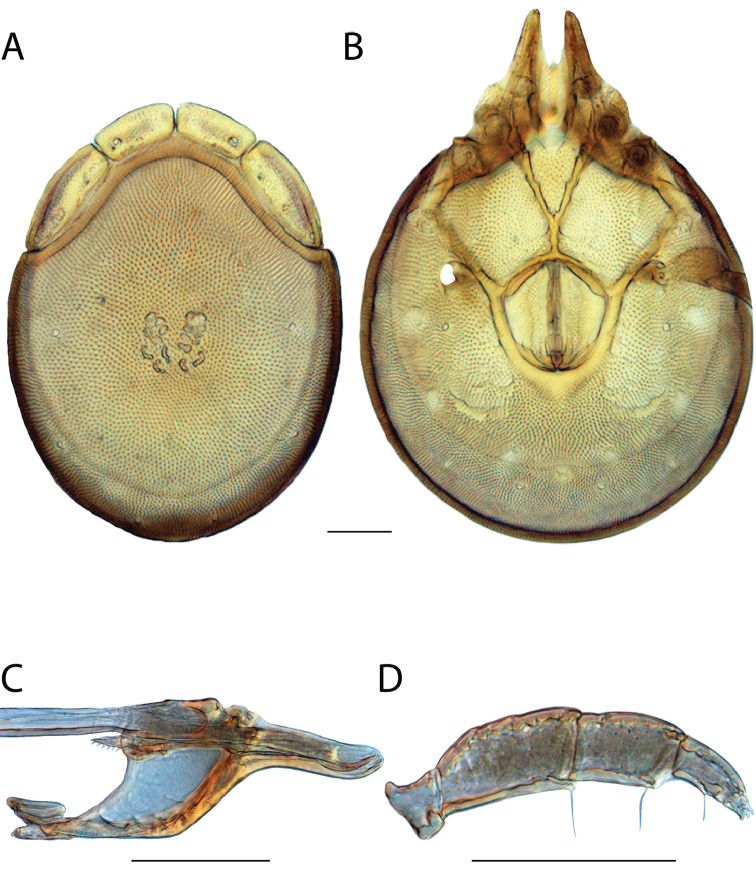
*Torrenticola kurtvietsi* female: **A** dorsal plates **B** venter (legs removed) **C** subcapitulum **D** pedipalp (setae not accurately depicted). Scale = 100 µm.

**Figure 122.**
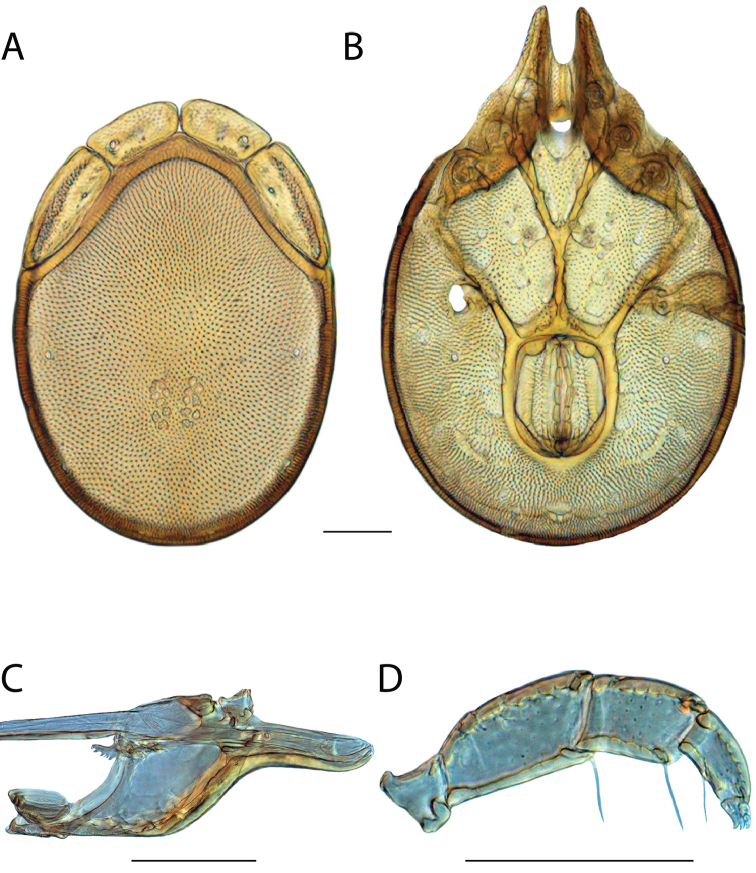
*Torrenticola kurtvietsi* male: **A** dorsal plates **B** venter (legs removed) **C** subcapitulum **D** pedipalp (setae not accurately depicted). Scale = 100 µm.

######## Remarks.

Unfortunately, we were unable to acquire fresh material of *Torrenticola kurtvietsi* and therefore this species is not included in our phylogenetic analyses. We were also unable to examine type material. However, we were able to examine new material from Arizona and Mexico. Based upon overall similarity, incomplete hind coxal margins, distribution, and lack of coloration, this species fits within our concept of the Rala Identification Group.

####### Torrenticola lamellipalpis

Taxon classificationAnimaliaTrombidiformesTorrenticolidae

K.O. Viets, 1977

Torrenticola lamellipalpis K.O. Viets, 1977b: 534.

######## Material examined

**(5** ♀; **4** ♂) **. New Mexico, USA**: 2 ♀ and 2 ♂ from Catron County, beside Rt. 15, 65 km north of Rt. 180 (Silver City), (33°12'12"N, 108°13'13"W), 10 July 1987, by IM Smith, IMS870081A • 2 ♀ from Catron County, beside Rt. 15 just below mouth of Little Creek, (33°12'12"N, 108°13'13"W), 11 July 1987, by IM Smith, IMS870083A • 2 ♂ from Catron County, Glenwood; Whitewater Picnic Area 8 km east of Rt. 180, (33°22'22"N, 108°50'50"W), 12 July 1987, by IM Smith, IMS870084 • 1 ♀ from Grant County, Gila River Recreation Area beside Rt. 15 at Grapevine Recreation Area north of Silver City, (33°11'11"N, 108°12'12"W), 11 July 1987, by IM Smith, IMS870082B.

######## Type deposition.

Holotype (♀) and allotype (♂) deposited in coll. Kurt Otto Viets, Senckenberg Museum Frankfurt, Germany.

######## Diagnosis.

*Torrenticola lamellipalpis* are similar to other members of the Rala Group (*T. rala*, *T. keesdavidsi*, *T. boettgeri*, *T. kurtvietsi*, *T. dolichodactyla*, and *T. anoplopalpa*) by being colorless, having incomplete hind coxal margins and being distributed in the southwest. *T. lamellipalpis* can be differentiated from all other Rala Group by having a wide, prominent lamellate extension on the pedipalpal femora that extend to the genua (others either without extensions or with tuberculate or flanged, dentate extensions).

######## Re-description.

**Female (Figure 124)** (n = 5) with characters of the genus with following specifications.

**Dorsum** — (660–735 long; 425–490 wide) ovoid and colorless. Anterio-medial platelets (162.5–175 long; 55–57.5 wide). Anterio-lateral platelets (205–225 long; 65–75 wide) free from dorsal plate. Dgl-4 closer to the edge of the dorsum than to the muscle scars (distance between Dgl-4 355–370). Dorsal plate proportions: dorsum length/width 1.46–1.60; dorsal width/distance between Dgl-4 1.20–1.32; anterio-medial platelet length/width 2.87–3.05; anterio-lateral platelet length/width 2.80–3.35; anterio-lateral/anterio-medial length 1.17–1.36.

**Gnathosoma — Subcapitulum** (440–465 long (ventral); 330–350 long (dorsal); 125–135 tall) colorless. Rostrum (175–187.5 long; 55–60 wide). Chelicerae (390–420 long) with curved fangs (60–65 long). Subcapitular proportions: ventral length/height 3.38–3.52; rostrum length/width 3.00–3.41. **Pedipalps** with wide, prominent lamellate extensions on femora that extends halfway into the genua. Palpomeres: trochanter (55–60 long); femur (145–152.5 long); genu (75–77.5 long); tibia (97.5–102.5 long; 22.5–23.75 wide); tarsus (17.5–20 long). Palpomere proportions: femur/genu 1.90–1.97; tibia/femur 0.66–0.69; tibia length/width 4.32–4.56.

**Venter** — (815–930 long; 500–560 wide) colorless. Gnathosomal bay (180–195 long; 80–100 wide). Cxgl-4 subapical. **Medial suture** (35–45 long). **Genital plates** (185–195 long; 155–165 wide). Additional measurements: Cx-1 (380–390 long (total); 190–200 long (medial)); Cx-3 (350–365 wide); anterior venter (240–260 long). Ventral proportions: gnathosomal bay length/width 1.90–2.31; anterior venter/genital field length 1.23–1.38; anterior venter length/genital field width 1.48–1.65; anterior venter/medial suture 5.67–6.86.

**Male (Figure 125)** (n = 4) with characters of the genus with following specifications.

**Dorsum** — (620–680 long; 400–460 wide) ovoid and colorless. Anterio-medial platelets (147.5–165 long; 52.5–57.5 wide). Anterio-lateral platelets (195–217.5 long; 70–77.5 wide) free from dorsal plate. Dgl-4 closer to the edge of the dorsum than to the muscle scars (distance between Dgl-4 320–360). Dorsal plate proportions: dorsum length/width 1.48–1.58; dorsal width/distance between Dgl-4 1.25–1.28; anterio-medial platelet length/width 2.77–3.14; anterio-lateral platelet length/width 2.69–3.04; anterio-lateral/anterio-medial length 1.28–1.32.

**Gnathosoma — Subcapitulum** (392.5–427.5 long (ventral); 292.5–325 long (dorsal); 112.5–127.5 tall) colorless. Rostrum (155–170 long; 50–55 wide). Chelicerae (350–390 long) with curved fangs (55–55 long). Subcapitular proportions: ventral length/height 3.29–3.56; rostrum length/width 3.09–3.24. **Pedipalps** with wide, prominent lamellate extensions on femora that extends halfway into the genua. Palpomeres: trochanter (51.25–55 long); femur (130–135 long); genu (70–77.5 long); tibia (97.5–105 long; 20–22.5 wide); tarsus (17.5–20 long). Palpomere proportions: femur/genu 1.74–1.86; tibia/femur 0.74–0.79; tibia length/width 4.67–4.94.

**Venter** — (780–840 long; 450–520 wide) colorless. Gnathosomal bay (170–195 long; 80–90 wide). Cxgl-4 subapical. **Medial suture** (110–130 long). **Genital plates** (160–180 long; 107.5–127.5 wide). Additional measurements: Cx-1 (335–370 long (total); 170–185 long (medial)); Cx-3 (325–375 wide); anterior venter (295–325 long). Ventral proportions: gnathosomal bay length/width 2.00–2.29; anterior venter/genital field length 1.75–1.86; anterior venter length/genital field width 2.47–2.83; anterior venter/medial suture 2.42–2.83.

**Immatures** unknown.

######## Etymology.

Viets (1977b) named the specific epithet (*lamellipalpis*) in reference to the “extensive ventral lamella” on the pedipalp femora (*lāmella*, L. small plate or flake; *palpus*, L. hand, feeler).

######## Distribution.

**Figure 123.**
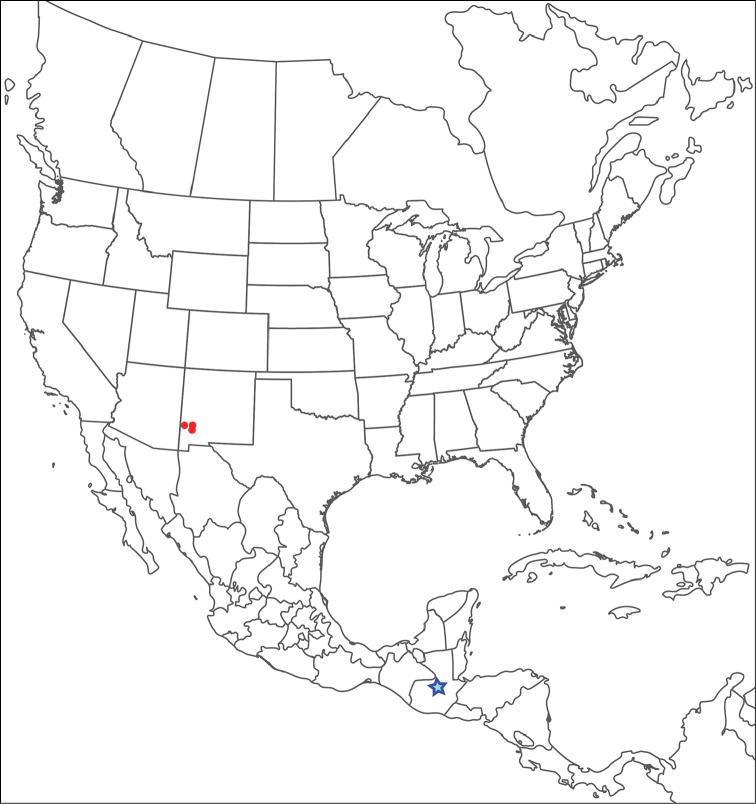
*Torrenticola lamellipalpis* distribution. Blue star represents type locality (K.O. Viets 1977b); and red circles represent new records and material examined.

Southwestern New Mexico (probably also found in southeastern Arizona), extending southward into Guatemala (Figure 123).

**Figure 124.**
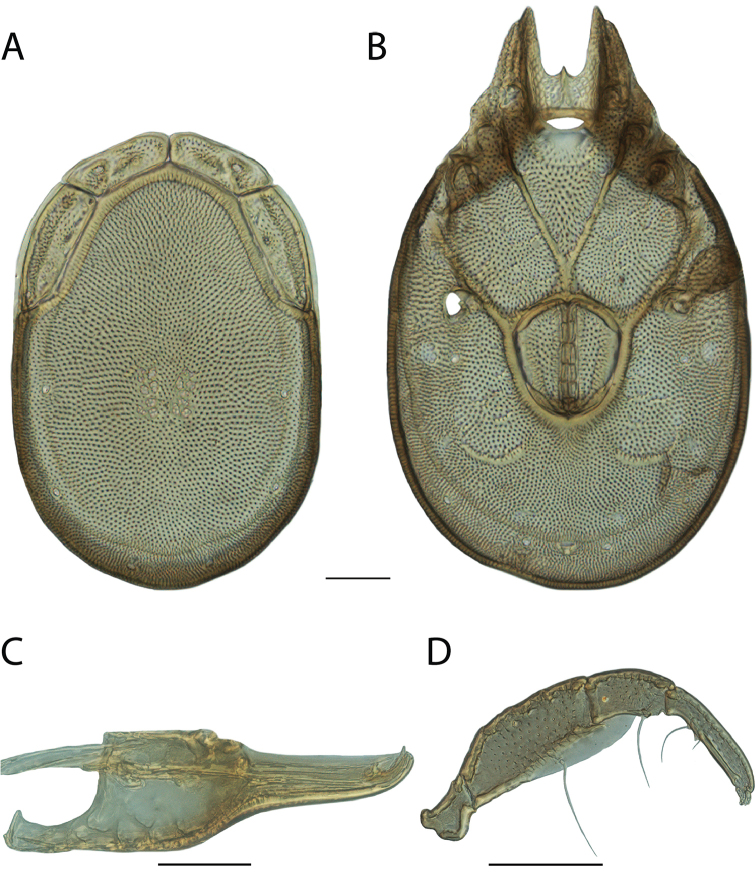
*Torrenticola lamellipalpis* female: **A** dorsal plates **B** venter (legs removed) **C** subcapitulum **D** pedipalp (setae not accurately depicted). Scale = 100 µm.

**Figure 125.**
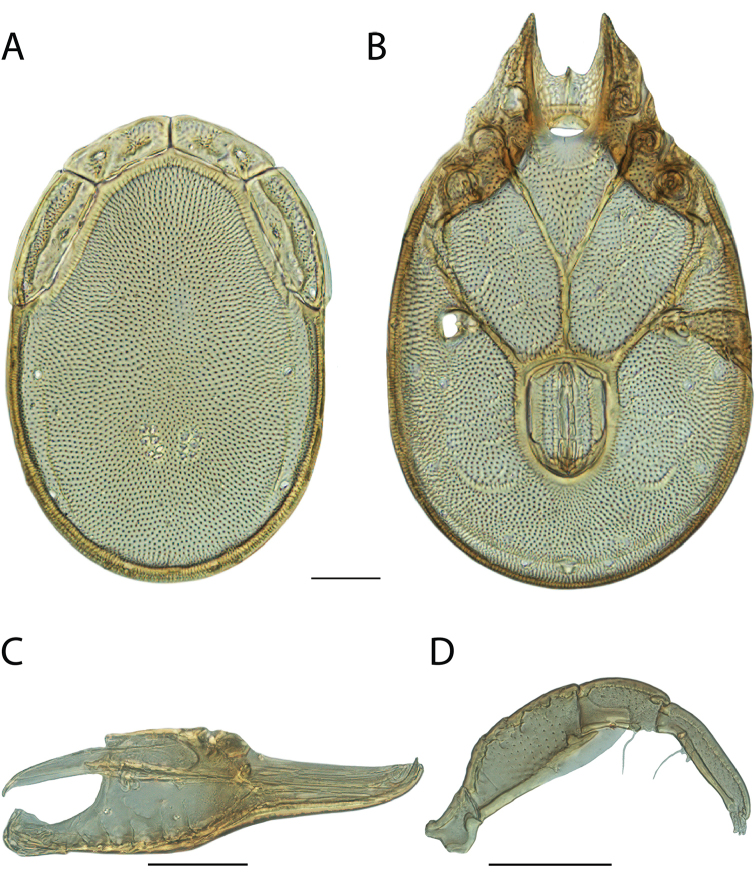
*Torrenticola lamellipalpis* male: **A** dorsal plates **B** venter (legs removed) **C** subcapitulum **D** pedipalp (setae not accurately depicted). Scale = 100 µm.

######## Remarks.

Unfortunately, we were unable to acquire fresh material of *Torrenticola lamellipalpis* and therefore this species is not included in our phylogenetic analyses. We were also unable to examine type material. However, we were able to examine new material from New Mexico. Based upon overall appearance, incomplete hind coxal margins, distribution, and lack of coloration, we were able to place this species within our concept of the Rala Identification Group.

####### Torrenticola larvata

Taxon classificationAnimaliaTrombidiformesTorrenticolidae

Cheri, Fisher, & Dowling, 2016

Torrenticola larvata Cherri et al. (2016): 246.

######## Material examined.

HOLOTYPE (♀): USA, Arkansas, Polk Co., Bard Springs, Ouachita National Forest, Blaylock Creek (34°23'28.3"N, 94°00'31.8"W), 11 Aug 2009, by AJ Radwell and BG Crump, AJR090307B.

PARATYPES (5 ♀; 8 ♂): **Arkansas, USA**: 1 ♂ (ALLOTYPE) from Polk County, Bard Springs, Ouachita National Forest, Blaylock Creek (34°23'28.3"N, 94°00'31.8"W), 11 Aug 2009, by AJ Radwell and BG Crump, AJR090307B • 3 ♀ from Polk County, beside Forest Road 38, North of Shady Lake Rec Area, East Saline Creek (34°22'53.4"N, 94°01'51.2"W), 30 Jul 2011, by IM Smith, IMS110041 • 1 ♀ and 6 ♂ from Montgomery County, Ouachita National Forest, Ouachita River at Mcguire (34°22'53.4"N, 94°1'51.2"W), 27 Aug 2011, by AJ Radwell, AJR110307 • 1 ♀ and 1 ♂ from Garland County, beside Route 7, 3 miles south of Mountain Valley, South Fork of Saline River (34°35'43.3"N, 93°00'45.3"W), 11 May 1977, by DR Cook, DRC770002 • 1 ♂ from Montgomery County, Ouachita National Forest, Ouachita River at Pine Ridge (34°34'53.5"N, 93°53'00.9"W), 5 Oct 2007, by AJ Radwell and HW Robison, AJR070300A.

######## Type deposition.

Holotype (♀), allotype (♂), and most paratypes (3 ♀; 5 ♂) deposited in the CNC; other paratypes (2 ♀; 3 ♂) deposited in ACUA.

######## Diagnosis.

*Torrenticola larvata* are similar to other members of the Tricolor Complex (*T. bittikoferae*, *T. hoosieri*, *T. pearsoni*, *T. olliei*, *T. sierrensis*, *T. tricolor*, *T. trimaculata*, *T. unimaculata*, *T. cardia*, *T. kringi*, *T. dimorpha*, and *T. mohawk*) in having a short, conical rostrum. *T. larvata* can be differentiated from all other *Torrenticola*, including other members of the Tricolor Complex, by having a distinct dorsal pattern. *T. larvata* can be further differentiated from *T. bittikoferae*, *T. sierrensis*, *T. tricolor*, *T. trimaculata*, *T. kringi*, and *T. mohawk* by being more elongate (length/width = 1.41–1.57 in *T. larvata*; 1.17–1.39 in others); and from *T. bittikoferae*, *T. olliei*, *T. sierrensis*, *T. trimaculata*, *T. unimaculata*, *T. dimorpha*, and *T. mohawk* by having a more elongate rostrum (length/width = 2.32–2.53 in *T. larvata*, 1.56–2.27 in others).

######## Re-description

**(amended from Cherri et al. 2016). Female (Figure 127)** (n = 6) (holotype measurements in parentheses when available) with characters of the genus with following specifications.

**Dorsum** — (650–725 (650) long; 450–475 (460) wide) ovoid and elongate with bluish-purple coloration restricted to the anterio-medial platelets and anterior-most portion of anterio-lateral platelets (rarely continuing to anterior border of the dorsal plate) and to the posterior dorsal plate within the area of primary sclerotization (posterior coloration is sometimes absent) with wide strip of orange medially. Anterio-medial platelets (127.5–132.5 (127.5) long; 175–190 (175) wide). Anterio-lateral platelets (175–190 (175) long; 70–77.5 (70) wide) free from dorsal plate. Dgl-4 much closer to the edge of the dorsum than to the muscle scars (distance between Dgl-4 340–375 (340)). Dorsal plate proportions: dorsum length/width 1.41–1.54 (1.41); dorsal width/distance between Dgl-4 1.27–1.35 (1.35); anterio-medial platelet length/width 1.83–2.04 (2.04); anterio-lateral platelet length/width 2.29–2.56 (2.50); anterio-lateral/anterio-medial length 1.37–1.43 (1.37).

**Gnathosoma — Subcapitulum** (275–287.5 (275) long (ventral); 200–211 (200) long (dorsal); 125–140 (125) tall) with bluish-purple coloration. Rostrum (110–117.5 (112.5) long; 47.5–50 (47.5) wide) short and conical. Chelicerae (260–295 (265) long) with curved fangs (53–62 (60) long). Subcapitular proportions: ventral length/height 2.04–2.25 (2.20); rostrum length/width 2.32–2.42 (2.37). **Pedipalps** with tuberculate ventral extensions with dentate tips on femora and genua. Palpomeres: trochanter (40–42.5 (40) long); femur (105–107.5 (107.5) long); genu (67.5–72.5 (67.5) long); tibia (87.5–97.5 (90) long; 25–30 (26.25) wide); tarsus (25–27.5 (25) long). Palpomere proportions: femur/genu 1.48–1.59 (1.59); tibia/femur 0.81–0.91 (0.84); tibia length/width 3.25–3.50 (3.43).

**Venter** — (720–850 (795) long; 525–604 (525) wide) colorless or with bluish-purple coloration, but always with bold bluish-purple on the dorsal coxal area. Gnathosomal bay (117.5–140 (140) long; 77.5–92.5 (77.5) wide). Cxgl-4 subapical. **Medial suture** (25–35 (35) long). **Genital plates** (182.5–187.5 (185) long; 145–152.5 (150) wide). Additional measurements: Cx-1 (260–358.5 (260) long (total); 125–183 (125) long (medial)); Cx-3 (307–375 (325) wide); anterior venter (180–195 (180) long). Ventral proportions: gnathosomal bay length/width 1.31–1.81 (1.81); anterior venter/genital field length 0.97–1.04 (0.97); anterior venter length/genital field width 1.20–1.31 (1.20); anterior venter/medial suture 5.14–7.80 (5.14).

**Male (Figure 128)** (n = 6) (allotypic measurements in parentheses when available) with characters of the genus with following specifications.

**Dorsum** — (550–610 (560) long; 350–400 (360) wide) ovoid and elongate with bluish-purple coloration restricted to the anterio-medial platelets and anterior-most portion of anterio-lateral platelets (rarely continuing to anterior border of the dorsal plate) and to the posterior dorsal plate within the area of primary sclerotization (posterior coloration is sometimes absent) with wide strip of orange medially. Anterio-medial platelets (115–120 (120) long; 52.5–65 (55) wide). Anterio-lateral platelets (147.5–162.5 (155) long; 57.5–70 (70) wide) free from dorsal plate. Dgl-4 much closer to the edge of the dorsum than to the muscle scars (distance between Dgl-4 285–340 (295)). Dorsal plate proportions: dorsum length/width 1.53–1.57 (1.56); dorsal width/distance between Dgl-4 1.18–1.26 (1.22); anterio-medial platelet length/width 1.85–2.24 (2.18); anterio-lateral platelet length/width 2.21–2.83 (2.21); anterio-lateral/anterio-medial length 1.26–1.41 (1.29).

**Gnathosoma — Subcapitulum** (230–247.5 (240) long (ventral); 167–182 (175) long (dorsal); 97.5–102.5 (100) tall) with bluish-purple coloration. Rostrum (91.25–100 (93.75) long; 37.5–40 (40) wide) short and conical. Chelicerae (221–239 (225) long) with curved fangs (45–52 (50) long). Subcapitular proportions: ventral length/height 2.30–2.46 (2.40); rostrum length/width 2.34–2.53 (2.34). **Pedipalps** with tuberculate ventral extensions with dentate tips on femora and genua. Palpomeres: trochanter (32.5–37.5 (35) long); femur (85–95 (90) long); genu (56.25–62.5 (60) long); tibia (75–82.5 (80) long; 23.75–26.25 (25) wide); tarsus (22.5–26.25 (25) long). Palpomere proportions: femur/genu 1.48–1.61 (1.50); tibia/femur 0.82–0.89 (0.89); tibia length/width 3.10–3.20 (3.20).

**Venter** — (660–710 (680) long; 415–443 (420) wide) colorless or with bluish-purple coloration, but always with bold bluish-purple on the dorsal coxal area. Gnathosomal bay (112.5–130 (125) long; 65–70 (67.5) wide). Cxgl-4 subapical. **Medial suture** (102.5–125 (110) long). **Genital plates** (138.75–147.5 (140) long; 97.5–110 (110) wide). Additional measurements: Cx-1 (244–266 (250) long (total); 135–140 (140) long (medial)); Cx-3 (283–305 (295) wide); anterior venter (245–270 (260) long). Ventral proportions: gnathosomal bay length/width 1.67–1.94 (1.85); anterior venter/genital field length 1.77–1.89 (1.86); anterior venter length/genital field width 2.36–2.77 (2.36); anterior venter/medial suture 2.16–2.41 (2.36).

**Immatures** unknown.

######## Etymology.

Cherri et al. (2016) named the specific epithet (*larvata*) for the anterior coloration that gives adults a masked appearance (*larvat*, L. masked).

######## Distribution.

**Figure 126.**
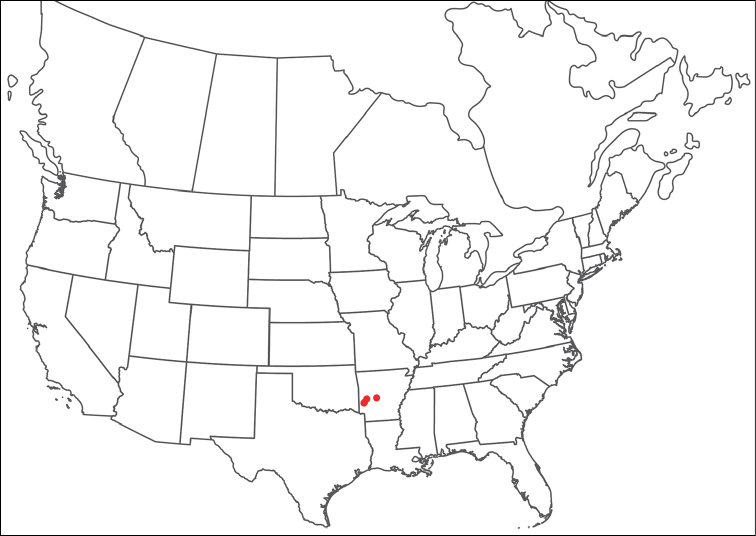
*Torrenticola larvata* distribution.

Ouachita Mountains of Arkansas (Figure 126).

**Figure 127.**
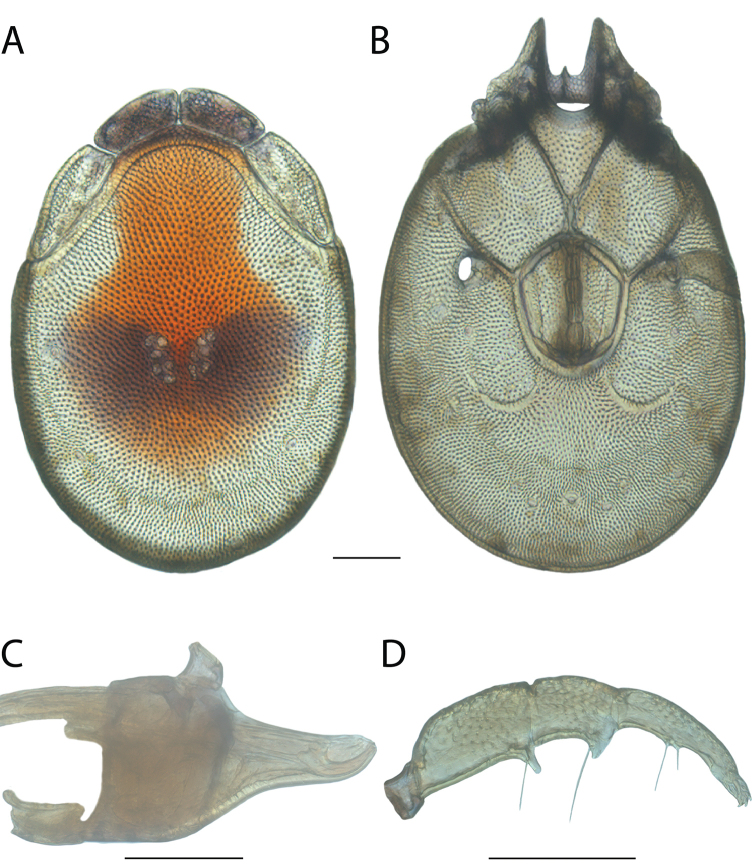
*Torrenticola larvata* female: **A** dorsal plates **B** venter (legs removed) **C** subcapitulum **D** pedipalp (setae not accurately depicted). Scale = 100 µm.

**Figure 128.**
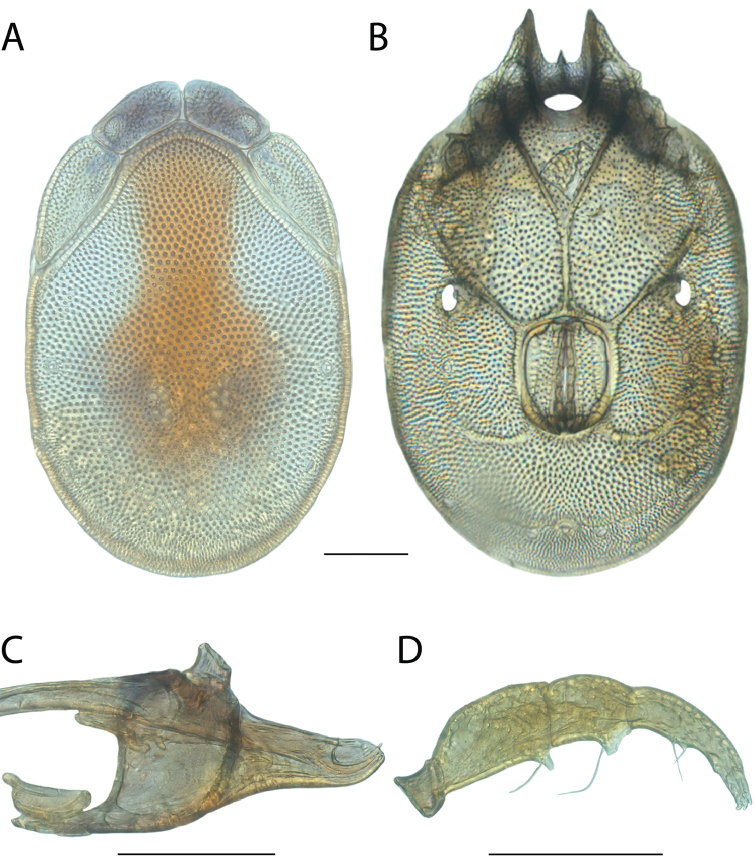
*Torrenticola larvata* male: **A** dorsal plates **B** venter (legs removed) **C** subcapitulum **D** pedipalp (setae not accurately depicted). Scale = 100 µm.

######## Remarks.

*Torrenticola larvata* groups with other members of the Tricolor Complex with high support in all analyses and specimens are less than 1% different in COI sequence from each other. In the combined analysis, *T. larvata* groups with *T. dimorpha* to form a clade sister to all other eastern members of this complex. These species are greater than 11% from each other. Based upon overall similarity, short conical rostrum, long pedipalp tibiae, phylogenetic position, and distribution, we were able to place this species in the Tricolor Identification Group.

This species hypothesis is supported by low COI divergence within the species (0–2%), high divergence between species (3–15%), and the morphological characters outlined in the diagnosis.

####### Torrenticola leviathan

Taxon classificationAnimaliaTrombidiformesTorrenticolidae

Fisher & Dowling sp. n.

http://zoobank.org/2312DACD-A8B7-4759-BB7B-E5F1018BE665

######## Material examined.

HOLOTYPE (♀): from USA, California, Mendocino County, beside Rt. 128 at Paul M. Dimmick Recreation Area, (39°10'10"N, 123°38'38"W), 4 August 1987, by IM Smith, IMS870127A

PARATYPES (3 ♀; 1 ♂): **California, USA**: 1 ♂(ALLOTYPE) from Mendocino, beside Rt. 128 at Paul M. Dimmick Recreation Area, (39°10'10"N, 123°38'38"W), 4 August 1987, by IM Smith, IMS870127A • 3 ♀ from Mendocino County, beside Rt. 128 at Paul M. Dimmick Recreation Area, (39°10'10"N, 123°38'38"W), 4 August 1987, by IM Smith, IMS870127A

######## Type deposition.

Holotype (♀), allotype (♂), and some paratypes (1 ♀) deposited in the CNC; other paratypes (1 ♀) deposited in the ACUA.

######## Diagnosis.

*Torrenticola leviathan* are similar to other members of the Ellipsoidalis Group (*T. multiforma*, *T. occidentalis*, and *T. ellipsoidalis*), in being among the largest *Torrenticola* in the west (dorsum length ♀ = 700–885; ♂ = 665–850), although *T. sierrensis* are also large (dorsum length ♀ = 700–880; ♂ = 590–735) but can easily be distinguished from the Ellipsoidalis Group by being circular instead of ellipsoid or rectangular (dorsum length/width = 1.17–1.28 in *T. sierrensis*, 1.30–1.67 in Ellipsoidalis Group). *T. leviathan* can be differentiated from all other Ellipsoidalis Group by having more elongate pedipalpal tibiae (length/width = 3.43–4.20 in *T. leviathan*, 2.64–3.33 in others).

######## Description.

**Female (Figure 130)** (n = 4) (holotype measurements in parentheses when available) with characters of the genus with following specifications.

**Dorsum** — (845–870 (870) long; 570–610 (580) wide) ellipsoid with faint orange coloration without a distinct pattern or colorless. Anterio-medial platelets (150–155 (155) long; 70–80 (75) wide). Anterio-lateral platelets (240–260 (245) long; 95–105 (95) wide) free from dorsal plate. Dgl-4 closer to the edge of the dorsum than to the muscle scars (distance between Dgl-4 420–460 (425)). Dorsal plate proportions: dorsum length/width 1.40–1.51 (1.50); dorsal width/distance between Dgl-4 1.33–1.36 (1.36); anterio-medial platelet length/width 1.94–2.14 (2.07); anterio-lateral platelet length/width 2.40–2.58 (2.58); anterio-lateral/anterio-medial length 1.55–1.70 (1.58).

**Gnathosoma — Subcapitulum** (330–350 (330) long (ventral); 230–230 (230) long (dorsal); 165–170 (170) tall) colorless. Rostrum (125–130 (125) long; 55–60 (55) wide) short and conical. Chelicerae (335–340 (340) long) with curved fangs (80–85 (80) long). Subcapitular proportions: ventral length/height 2.00–2.06 (2.00); rostrum length/width 2.17–2.27 (2.27). **Pedipalps** with short, tuberculate ventral extensions on femora and genua. Palpomeres: trochanter (50–52.5 (52.5) long); femur (130–130 (130) long); genu (75–77.5 (77.5) long); tibia (105–107.5 (107.5) long; 25–27.5 (27.5) wide); tarsus (20–22.5 (22.5) long). Palpomere proportions: femur/genu 1.68–1.73 (1.68); tibia/femur 0.81–0.83 (0.83); tibia length/width 3.91–4.20 (3.91).

**Venter** — (970–1035 (1035) long; 640–680 (640) wide) colorless. Gnathosomal bay (190–215 (190) long; 85–100 (100) wide). Cxgl-4 subapical. **Medial suture** (25–30 (30) long). **Genital plates** (210–215 (210) long; 180–195 (180) wide). Additional measurements: Cx-1 (390–400 (390) long (total); 180–205 (205) long (medial)); Cx-3 (440–460 (440) wide); anterior venter (220–245 (245) long). Ventral proportions: gnathosomal bay length/width 1.90–2.53 (1.90); anterior venter/genital field length 1.05–1.17 (1.17); anterior venter length/genital field width 1.13–1.36 (1.36); anterior venter/medial suture 7.50–8.80 (8.17).

**Male (Figure 131)** (n = 1) (allotype only) with characters of the genus with following specifications.

**Dorsum** — (770 long; 510 wide) ellipsoid with faint orange coloration without a distinct pattern or colorless. Anterio-medial platelets (140 long; 65 wide). Anterio-lateral platelets (230 long; 85 wide) free from dorsal plate. Dgl-4 closer to the edge of the dorsum than to the muscle scars (distance between Dgl-4 400). Dorsal plate proportions: dorsum length/width 1.51; dorsal width/distance between Dgl-4 1.28; anterio-medial platelet length/width 2.15; anterio-lateral platelet length/width 2.71; anterio-lateral/anterio-medial length 1.64.

**Gnathosoma — Subcapitulum** (285 long (ventral); 200 long (dorsal); 137.5 tall) colorless. Rostrum (95 long; 52.5 wide) short and conical. Chelicerae (275 long) with curved fangs (75 long). Subcapitular proportions: ventral length/height 2.07; rostrum length/width 1.81. **Pedipalps** with short, tuberculate ventral extensions on femora and genua. Palpomeres: trochanter (47.5 long); femur (112.5 long); genu (70 long); tibia (90 long; 26.25 wide); tarsus (20 long). Palpomere proportions: femur/genu 1.61; tibia/femur 0.80; tibia length/width 3.43.

**Venter** — (860 long; 570 wide) colorless. Gnathosomal bay (180 long; 90 wide). Cxgl-4 subapical. **Medial suture** (110 long). **Genital plates** (160 long; 120 wide). Additional measurements: Cx-1 (360 long (total); 185 long (medial)); Cx-3 (420 wide); anterior venter (305 long). Ventral proportions: gnathosomal bay length/width 2.00; anterior venter/genital field length 1.91; anterior venter length/genital field width 2.54; anterior venter/medial suture 2.77.

**Immatures** unknown.

######## Etymology.

Specific epithet (*leviathan*) refers to the large body size of this species, surpassed by few other *Torrenticola* (*liwyātān*, Hebrew, a large aquatic animal; noun in apposition). The Leviathan (modern spelling) is depicted in Jewish mythology (as written in the Tanakh) as one of two huge beasts—the Leviathan in the ocean and the Behemoth on land.

######## Distribution.

**Figure 129.**
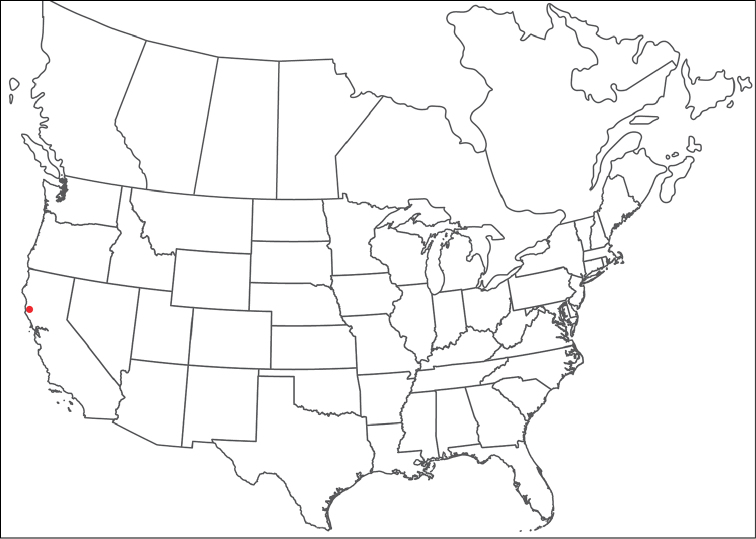
*Torrenticola leviathan* sp. n. distribution.

Northwestern California (Figure 129).

**Figure 130.**
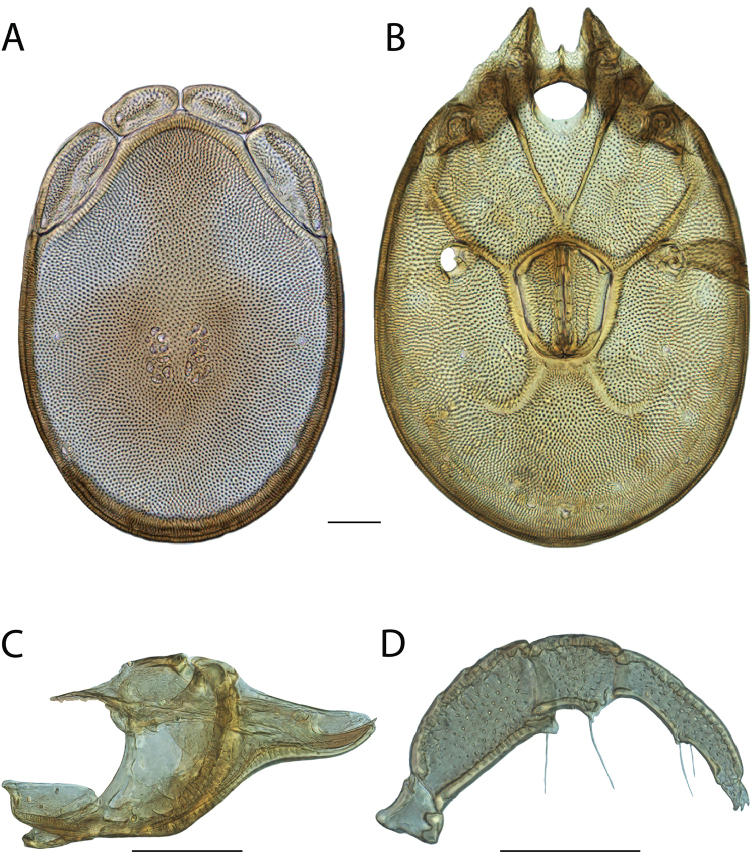
*Torrenticola leviathan* sp. n. female: **A** dorsal plates **B** venter (legs removed) **C** subcapitulum **D** pedipalp (setae not accurately depicted). Scale = 100 µm.

**Figure 131.**
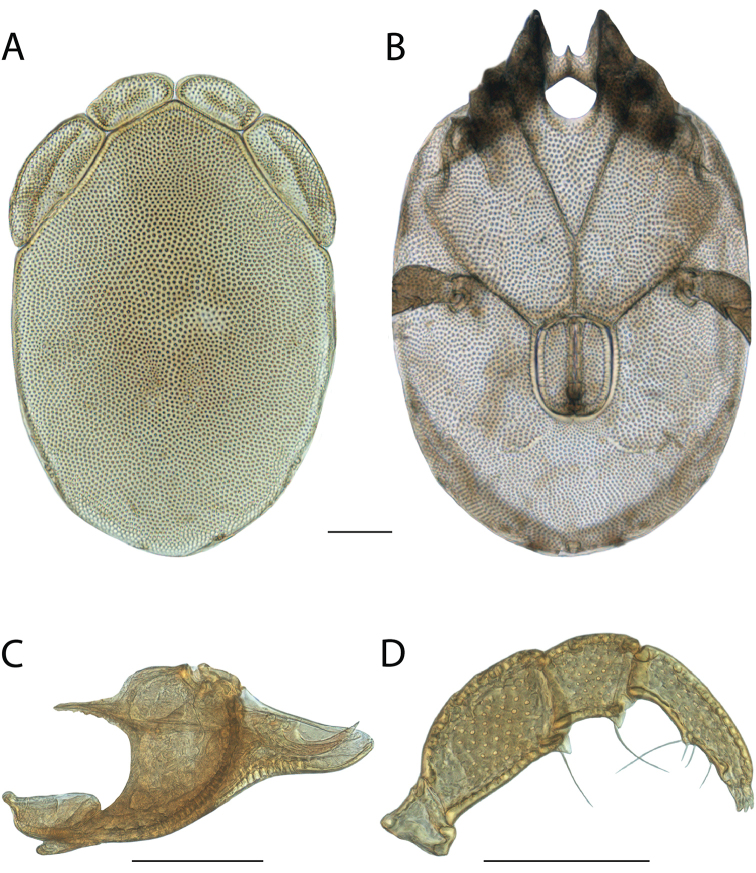
*Torrenticola leviathan* sp. n. male (teneral): **A** dorsal plates **B** venter (legs removed) **C** subcapitulum **D** pedipalp (setae not accurately depicted). Scale = 100 µm.

######## Remarks.

Unfortunately, we were unable to acquire fresh material of *Torrenticola leviathan* and therefore this species is not included in our phylogenetic analyses. However, we were able to examine morphology with material preserved in GAW. The overall appearance, large size, and western distribution, places this species in the Miniforma Complex and the Ellipsoidalis Identification Group.

####### Torrenticola longitibia

Taxon classificationAnimaliaTrombidiformesTorrenticolidae

Fisher & Dowling sp. n.

http://zoobank.org/BFF29739-A62F-498D-9F18-465D46964686

######## Material examined.

HOLOTYPE (♂): from USA, Tennessee, Monroe County, beside Forest Route 35 (35°21'47"N, 84°9'47"W), 12 Sep 2009, by IM Smith, IMS090112, DNA 2979.

PARATYPES (0 ♀; 1 ♂): **Tennessee, USA**: 1 ♂ from USA, Tennessee, Monroe County, beside Forest Route 35 (35°21'47"N, 84°9'47"W), 12 Sep 2009, by IM Smith, IMS090112.

######## Type deposition.

Holotype (♀) deposited in the CNC; paratypes (1 ♂) deposited in the ACUA.

######## Diagnosis.

*Torrenticola longitibia* are similar to other members of the Raptor Group (*T. gnoma*, *T. irapalpa*, *T. mjolniri*, *T. elusiva*, *T. racupalpa*, *T. raptor*, *T. danielleae*, *T. daemon*, and *T. ivyae*) in having round bodies; Dgl-4 close to muscles scars; long, thin subcapitular rostra; and long, thin pedipalp tibiae. *T. longitibia* can be differentiated from all other members of the Raptor Group (both males and females) by having a longer femur with respect to the genu (femur/genu = 2.1–2.17 in *T. longitibia*, 1.66–2.00 in others).

######## Description.

**Female** unknown.

**Male (Figure 133)** (n = 2) (holotype measurements in parentheses when available) with characters of the genus with following specifications.

**Dorsum**— (530–560 (530) long; 420–430 (420) wide) circular with navy blue coloration posteriorly extending in a thin strip anteriorly to the edge of the dorsal plate. Anterio-medial platelets (122.5–132.5 (122.5) long; 62.5–72.5 (62.5) wide). Anterio-lateral platelets (172.5–180 (172.5) long; 78.75–80 (80) wide) free from dorsal plate. Dgl-4 much closer to the muscle scars than to the dorsum edge (distance between Dgl-4 155–175 (155)). Dorsal plate proportions: dorsum length/width 1.26–1.30 (1.26); dorsal width/distance between Dgl-4 2.46–2.71 (2.71); anterio-medial platelet length/width 1.83–1.96 (1.96); anterio-lateral platelet length/width 2.16–2.29 (2.16); anterio-lateral/anterio-medial length 1.36–1.41 (1.41).

**Gnathosoma — Subcapitulum** (300–315 (300) long (ventral); 235–245 (235) long (dorsal); 110–117.5 (110) tall) colorless. Rostrum (135–137.5 (135) long; 32.5–32.5 (32.5) wide) elongate. Chelicerae (325–325 (325) long) with curved fangs (50–50 (50) long). Subcapitular proportions: ventral length/height 2.68–2.73 (2.73); rostrum length/width 4.15–4.23 (4.15). **Pedipalps** elongate (especially tibiae) with tuberculate ventral extensions on femora and genua. Palpomeres: trochanter (42.5–45 (42.5) long); femur (125–131.25 (125) long); genu (57.5–62.5 (57.5) long); tibia (110–110 (110) long; 20–20 (20) wide); tarsus (15–15 (15) long). Palpomere proportions: femur/genu 2.10–2.17 (2.17); tibia/femur 0.84–0.88 (0.88); tibia length/width 5.50–5.50 (5.50).

**Venter** — (660–710 (660) long; 500–550 (500) wide) colorless. Gnathosomal bay (150–155 (150) long; 75–85 (85) wide). Cxgl-4 far from apex. **Medial suture** (60–60 (60) long). **Genital plates** (137.5–150 (137.5) long; 115–122.5 (115) wide). Additional measurements: Cx-1 (270–300 (270) long (total); 130–140 (130) long (medial)); Cx-3 (320–325 (320) wide); anterior venter (205–220 (205) long). Ventral proportions: gnathosomal bay length/width 1.76–2.07 (1.76); anterior venter/genital field length 1.47–1.49 (1.49); anterior venter length/genital field width 1.78–1.80 (1.78); anterior venter/medial suture 3.42–3.67 (3.42).

**Immatures** unknown.

######## Etymology.

Specific epithet (*longitibia*) refers to the long, thin pedipalpal tibiae (*longus*, L. long; *tibia*, L. tibia).

######## Distribution.

**Figure 132.**
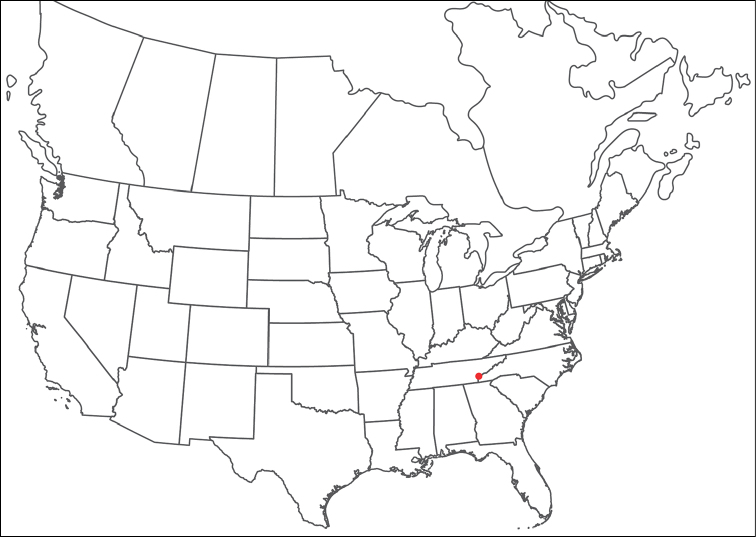
*Torrenticola longitibia* sp. n. distribution.

Known only from Monroe County, Tennessee (Figure 132).

**Figure 133.**
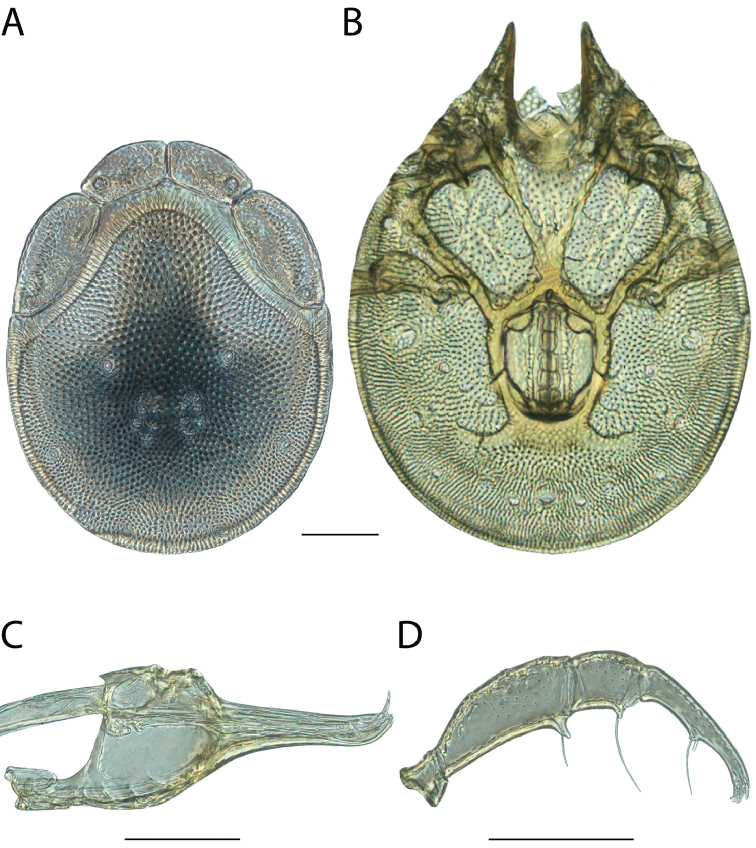
*Torrenticola longitibia* sp. n. male: **A** dorsal plates **B** venter (legs removed) **C** subcapitulum **D** pedipalp (setae not accurately depicted). Scale = 100 µm.

######## Remarks.

*Torrenticola longitibia* is known only from two males, only one of which was available for molecular data and that specimen groups with other members of the Raptor Complex with high support. In all analyses, *T. longitibia* groups with *T. mjolniri*, and these species are more than 4% different in COI sequence from each other. The position of that clade varies with dataset. Based upon overall similarity, phylogenetic position, shape of the pedipalps, and distribution, we were able to place this species within the Raptor Identification Group.

This species hypothesis is supported by high divergence between species (3–15%), and by the morphological characters outlined in the diagnosis.

####### Torrenticola magnexa

Taxon classificationAnimaliaTrombidiformesTorrenticolidae

Habeeb, 1955

Torrenticola amplexa magnexa Habeeb, 1955: 4
Torrenticola amplexa neoconnexa Habeeb, 1957: 1 (initially identified as Torrenticola amplexa connexa (Koenike, 1908) in Habeeb 1955: 4)
Torrenticola neoconnexa Habeeb, 1961: 2.
Torrenticola magnexa Habeeb, 1961: 2.

######## Material examined.

HOLOTYPE (♀): from Canada, New Brunswick, Victoria County, Salmon River, 21 June 1953, by Habeeb, HH530075.

PARATYPES (0 ♀; 1 ♂): **New Brunswick, Canada**: 1 ♂ (ALLOTYPE) from Victoria County, Salmon River, 21 June 1953, by Habeeb, HH530075.

OTHER MATERIAL (29 ♀; 14 ♂): **Maine, USA**: 2 ♀ from Franklin County, Smalls Falls Picnic Area, beside Route 4, Sandy River (44°52'N, 70°31'W), 5 Jul 1989, by IM Smith, IMS890069 • **Missouri, USA**: 1 ♀ from McDonald County, Tiff City, beside Route 43, Buffalo Creek (36°40'17"N, 94°36'17"W), 2 May 1996, by IM Smith, IMS960004 • **Nova Scotia, Canada**: 1 ♀ from Inverness County, Cape Brenton Highland National Park, 10 Sep 2011, by IM Smith, IMS110072 • 2 ♀ from Inverness County, Inervess, Cheticamp River, 10 Sep 2011, by IM Smith, IMS110071 • **New Brunswick, Canada**: 1 ♀ and 1 ♂ from Victoria County, St. Quentin, beside Highway 17, 14 km southwest of Highway 180, 14 Sep 2011, by IM Smith, IMS110059 • 2 ♀ from York County, Napadogan, beside Road J-19, 4 km south of Hwy 107, 6 Oct 2011, by IM Smith, IMS110126 • **Newfoundland, Canada**: 2 ♀ from Crooked Feeder, beside Highway 1, north of Deer Lake (49°10'N, 57°26'W), 25 Jul 1977, by IM Smith, IMS770125B • 1 ♀ and 1 ♂ from Deer Lake, beside Highway 430, 6.2 kilometers north of Highway 1 (49°10'N, 57°26'W), 23 Jul 1977, by IM Smith, IMS770112 • 2 ♀ and 2 ♂ from Molly Chignic Brook, beside Highway 1 (47°51'N, 59°10'W), 7 Oct 1989, by IM Smith, IMS890133 • **Ohio, USA**: 2 ♂ from Montgomery County, Engelwood Metro Park (39°52'58"N, 84°17'33"W), 31 Jul 2014, by MJ Skvarla, MS 14-0731-002 • **Quebec, Canada**: 1 ♀ from Gatineau County, Gatineau Park, Meech Lake (45°32'27"N, 75°54'53"W), 27 Jul 2010, by IM Smith, IMS100105 • 2 ♀ and 2 ♂ from Gatineau County, Gatineau Park, tributary above Blanchet Beach, 6 Apr 2012, by IM Smith, IMS120001 • 1 ♀ and 1 ♂ from Gatineau County, Gatineau Park, beside Wolf Trail above Blanchet (45°32'30"N, 75°55'29"W), 12 Jun 2008, by IM Smith, IMS080009A • 1 ♂ from Matane County, St.-Bernard-des-Lacs, Riviere Ste. Anne (48°55'45"N, 66°7'0"W), 25 Aug 1975, by BP Smith, DW Barr, & N Avruch, BPS750562 • 2 ♀ and 1 ♂ from Matapedia County, beside secondary road, Riviere Matapedia (48°17'40"N, 67°15'20"W), 24 Aug 1975, by BP smith, DW Barr, & N Avruch, BPS750557 • 1 ♀ from Stanstead County, Tompkin Stream, Tomifobia River (45°0'31"N, 72°7'6"W), 20 Aug 1996, by IM Smith & M MacKenzie, IMS960056 • **Tennessee, USA**: 1 ♀ from Blount County, Great Smokey Mountains National Park, Abrams River (35°35'31"N, 83°51'21"W), 17 Sep 2010, by IM Smith, IMS100141 • 1 ♀ from Sevier County, Great Smokey Mountains National Park (35°46'54"N, 83°13'2"W), 16 Sep 2010, by IM Smith, IMS100140 • 1 ♀ from Sevier County, Great Smokey Mountains National Park, middle prong Little Pigeon River (35°42'38"N, 83°22'59"W), 10 Sep 2009, by IM Smith, IMS090106 • **Vermont, USA**: 2 ♀ and 1 ♂ from Addison County, beside road from Lincoln to Ripton, Middlebury River (44°0'N, 73°1'W), 6 Jul 1989, by IM Smith, IMS890075 • 2 ♀ from Addison County, Lincoln, beside US Forest Service Road #54, New Haven River (44°6'N, 72°59'W), 6 Jul 1989, by IM Smith, IMS890074 • 2 ♀ and 2 ♂ from Lamoille County, Stowe, beside Route 108, West Branch of Waterbury River (44°30'N, 72°46'W), 6 Jul 1989, by IM Smith, IMS890072.

######## Type deposition.

Holotype (♀) and allotype (♂) deposited in the CNC.

######## Diagnosis.

*Torrenticola magnexa* are similar to other members of the Partial 2-Plate Group (*T. folkertsae*, *T. pulchra*, and *T. priapus*) in having anterio-lateral platelets partially fused to the dorsal plate and being distributed in the east. *T. magnexa* can be differentiated from other Partial 2-Plate Group by dorsal coloration and pattern. *T. magnexa* can be further differentiated from *T. New 23A* by having a less elongate rostrum (length/width = 2.25–3.00 in *T. magnexa*, 3.17–3.39 in *T. priapus*). *T. magnexa* can be further differentiated from *T. folkertsae* by having less elongate pedipalpal tibiae (length/width = 3.21–4.00 in *T. magnexa*, 4.05–4.83 in *T. folkertsae*). Male *T. magnexa* can be further differentiated from male *T. pulchra* by having a larger genital field (length ♂ = 125–148 in *T. magnexa*, 110–123 in *T. pulchra*; width ♂ = 115–125 in *T. magnexa*, 87–95 in *T. pulchra*) and more elongate pedipalpal tibiae (length/width ♂ = 3.78–4.00 in *T. magnexa*, 3.00–3.35 in *T. pulchra*). Female *T. magnexa* can be differentiated from female *T. pulchra* by having a wider genital field (♀ 170–188 in *T. magnexa*, 147–160 in *T. pulchra*) and longer pedipalpal tibiae (♀ 102–113 in *T. magnexa*, 82–93 in *T. pulchra*).

######## Re-description.

**Female (Figure 135)** (n = 6) (holotype measurements in parentheses when available) with characters of the genus with following specifications.

**Dorsum** — (680–750 (750) long; 500–560 (560) wide) ovoid with bluish-purple or reddish-purple coloration separated into anterior and posterior portions with red medially. Anterio-medial platelets 155–170 (162.5) long; 57.5–65 (60) wide). Anterio-lateral platelets (192.5–210 (210) long; 72.5–85 (85) wide) partially fused to dorsal plate (especially posteriorly). Dgl-4 close to the edge of the dorsum (distance between Dgl-4 390–410 (400)). Dorsal plate proportions: dorsum length/width 1.28–1.42 (1.34); dorsal width/distance between Dgl-4 1.28–1.40 (1.40); anterio-medial platelet length/width 2.42–2.72 (2.71); anterio-lateral platelet length/width 2.47–2.71 (2.47); anterio-lateral/anterio-medial length 1.13–1.33 (1.29).

**Gnathosoma — Subcapitulum** (330–355 long (ventral); 251–277 long (dorsal); 150–170 tall) mostly colorless. Rostrum (137.5–150 long; 47.5–52.5 wide). Chelicerae (335–363 long) with curved fangs (55–78 long). Subcapitular proportions: ventral length/height 2.00–2.37; rostrum length/width 2.75–3.00. **Pedipalps** with tuberculate ventral extensions on femora and genua. Palpomeres: trochanter (47.5–52.5 (50) long); femur (127.5–135 (135) long); genu (75–82.5 (80) long); tibia 102.5–107.5 (102.5) long; 26.25–30 (28.75) wide); tarsus (17.5–25 (22.5) long). Palpomere proportions: femur/genu 1.58–1.71 (1.69); tibia/femur 0.76–0.83 (0.76); tibia length/width 3.57–4.00 (3.57).

**Venter** — (770–900 (830) long; 588–672 (660) wide) colorless. Gnathosomal bay (165–190 (165) long; 95–125 (125) wide). Cxgl-4 subapical. **Medial suture** (0–15 (7.5) long) occasionally absent. **Genital plates** (190–207.5 (207.5) long; 170–187.5 (187.5) wide). Additional measurements: Cx-1 (299–359 (320) long (total); 96–155 (155) long (medial)); Cx-3 (369–455 (455) wide); anterior venter (167.5–175 (175) long). Ventral proportions: gnathosomal bay length/width 1.32–1.87 (1.32); anterior venter/genital field length 0.84–0.91 (0.84); anterior venter length/genital field width 0.93–1.03 (0.93); anterior venter/medial suture (proportion cannot be calculated for specimens without a medial suture) 11.33–23.33 (23.33).

**Male (Figure 136)** (n = 6) (allotypic measurements in parentheses when available) with characters of the genus with following specifications.

**Dorsum** — (540–630 (600) long; 390–450 (430) wide) ovoid with bluish-purple or reddish-purple coloration separated into anterior and posterior portions with red medially. Anterio-medial platelets (125–140 (132.5) long; 47.5–52.5 (50) wide). Anterio-lateral platelets (158.75–192.5 (170) long; 60–70 (60) wide) partially fused to dorsal plate (especially posteriorly). Dgl-4 close to the edge of the dorsum (distance between Dgl-4 310–350 (310)). Dorsal plate proportions: dorsum length/width 1.35–1.43 (1.40); dorsal width/distance between Dgl-4 1.26–1.39 (1.39); anterio-medial platelet length/width 2.48–2.95 (2.65); anterio-lateral platelet length/width 2.44–2.85 (2.83); anterio-lateral/anterio-medial length 1.22–1.43 (1.28).

**Gnathosoma — Subcapitulum** (272.5–300 (285) long (ventral); 200–229 (215) long (dorsal); 113–127.5 (122.5) tall) mostly colorless. Rostrum (110–122.5 (117.5) long; 40–45 (42.5) wide). Chelicerae (260–292 (290) long) with curved fangs (49–60 (55) long). Subcapitular proportions: ventral length/height 2.22–2.42 (2.33); rostrum length/width 2.59–2.84 (2.76). **Pedipalps** with tuberculate ventral extensions on femora and genua. Palpomeres: trochanter (37.5–45 (40) long); femur (105–112.5 (110) long); genu (65–72.5 (67.5) long); tibia (85–97.5 (90) long; 22.5–25 (22.5) wide); tarsus (17.5–20 (20) long). Palpomere proportions: femur/genu 1.55–1.67 (1.63); tibia/femur 0.80–0.87 (0.82); tibia length/width 3.78–4.00 (4.00).

**Venter** — (640–770 (710) long; 465–550 (550) wide) colorless. Gnathosomal bay (127.5–160 (127.5) long; 77.5–95 (77.5) wide). Cxgl-4 subapical. **Medial suture** (62.5–85 (75) long). **Genital plates** (125–147.5 (140) long; 115–125 (115) wide). Additional measurements: Cx-1 (251–285 (270) long (total); 102–140 (140) long (medial)); Cx-3 (335–375 (375) wide); anterior venter (207.5–240 (220) long). Ventral proportions: gnathosomal bay length/width 1.37–1.94 (1.65); anterior venter/genital field length 1.57–1.78 (1.57); anterior venter length/genital field width 1.80–1.98 (1.91); anterior venter/medial suture 2.82–3.68 (2.93).

**Immatures** unknown.

######## Etymology.

Habeeb (1957) did not specify an etymology for the specific epithet (*magnexa*). However, surely this name refers to the similarity of this species to the Palaearctic *T. connexa* (Koenike, 1908) (*néos*, G. new).

######## Distribution.

**Figure 134.**
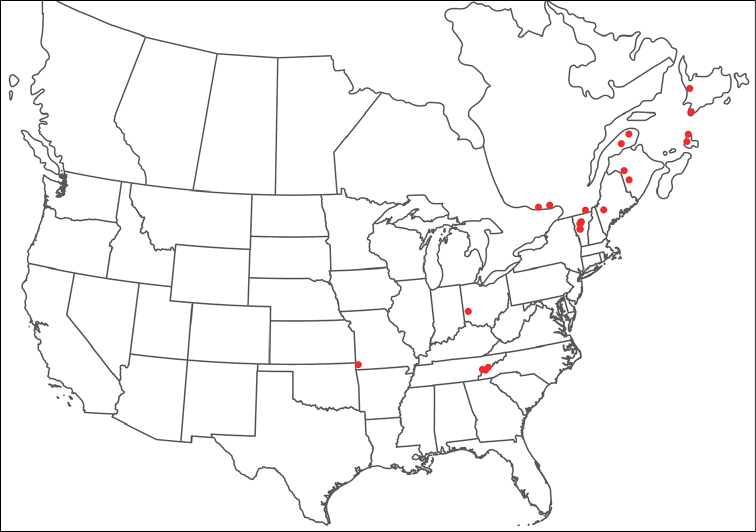
*Torrenticola magnexa* distribution.

Eastern (Figure 134).

**Figure 135.**
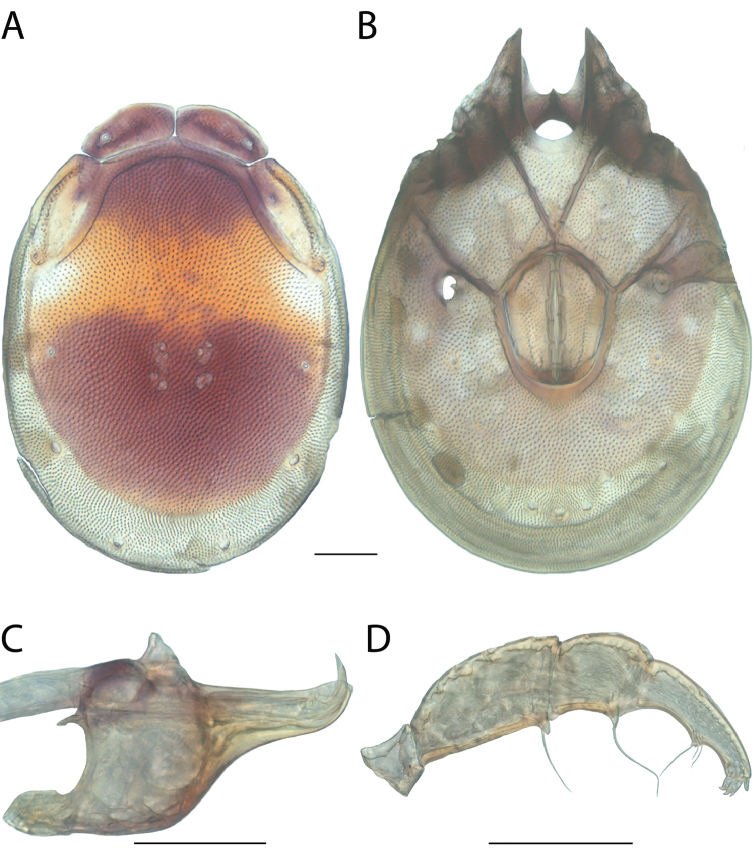
*Torrenticola magnexa* female: **A** dorsal plates **B** venter (legs removed) **C** subcapitulum **D** pedipalp (setae not accurately depicted). Scale = 100 µm.

**Figure 136.**
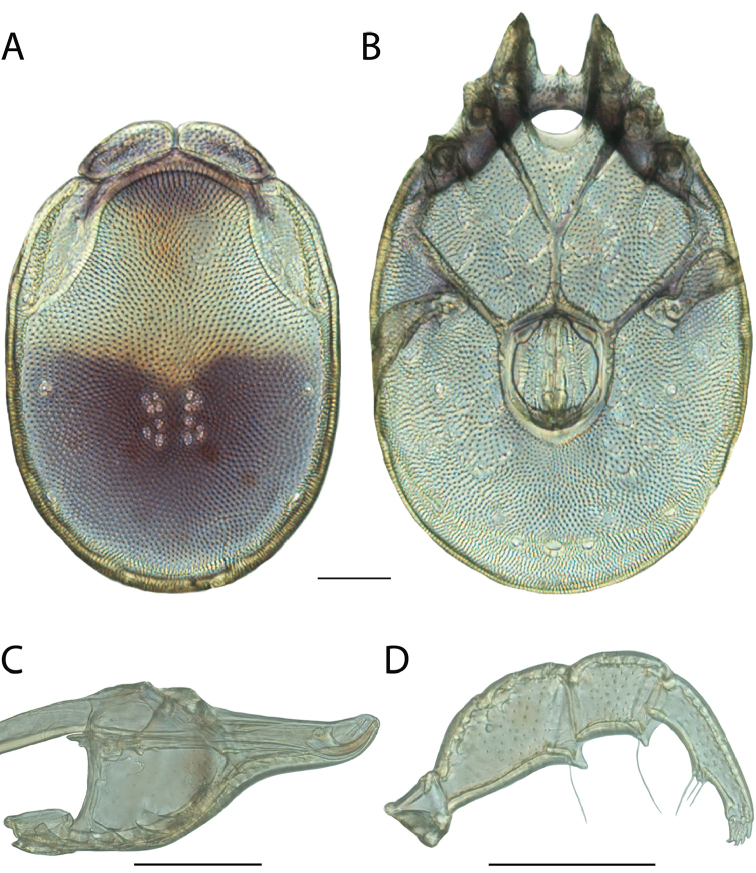
*Torrenticola magnexa* male: **A** dorsal plates **B** venter (legs removed) **C** subcapitulum **D** pedipalp (setae not accurately depicted). Scale = 100 µm.

######## Remarks.

Upon examining the types of *T. magnexa* and *T. neoconnexa* Habeeb, 1957, all characters for both species overlap with members of only one clade in our analyses. Furthermore, the primary character Habeeb (1955, 1957, 1961) used to differentiate *T. magnexa* from *T. neoconnexa* was body size, which is known to be a highly variable character. Therefore, it is apparent that these represent the same species hypothesis and must be synonymized. We therefore synonymize *T. neoconnexa* as the junior synonym of *T. magnexa*.

In all analyses, *Torrenticola magnexa* groups with other members of the Rusetria Complex with high support, all specimens were less than 1% different in COI sequence from each other, and these specimens were 11–13% different from sister species. In the combined analysis, all eastern members of Rusetria Complex are monophyletic, and *T. magnexa* was recovered at the base of that eastern clade. Interestingly, most of the species within the more basal lineages of this eastern clade have lateral platelets that are free from the dorsal plate; whereas western species and most eastern species have lateral platelets fused to the dorsal plate. This is interesting because *T. magnexa*, which have lateral platelets partially fused to the dorsal plate, is recovered in an intermediate position between western species that have fused platelets and eastern species with unfused platelets.

Based upon the partially fused posterio-lateral platelets and the distribution, we place this species within the Partial 2-Plate Identification Group.

This species hypothesis is supported by biogeography, low COI divergence within the species (0–2%) and high divergence between species (3–15%), and the morphological characters outlined in the diagnosis.

####### Torrenticola malarkeyorum

Taxon classificationAnimaliaTrombidiformesTorrenticolidae

Fisher & Dowling sp. n.

http://zoobank.org/A736573D-3D5F-4BFD-8CA2-1C568BB7F8AA

######## Material examined.

HOLOTYPE (♀): from USA, Missouri, Ozark County, Bryant Creek, downstream of Highway 95 bridge, 10 Sep 2011, by AJ Radwell, AJR110105A, DNA 2120.

PARATYPES (9 ♀; 7 ♂): **Maine, USA**: 1 ♂ from Aroostook County, Ashland, beside Route 11 at bridge, Aroostook River (46°38'N, 68°24'W), 4 Jul 1989, by IM Smith, IMS890067 • **Missouri, USA**: 1 ♀ and 1 ♂ from Oregon County, beside Route 19, north of Greer, Eleven Point River (36°48'N, 91°20'W), 28 Jun 1987, by IM Smith, IMS870056 • 1 ♂ (ALLOTYPE) from Ozark County, Bryant Creek, downstream of Highway 95 bridge, 10 Sep 2011, by AJ Radwell, AJR110105A • 1 ♀ from Ozark County, Bryant Creek, downstream of Highway 95 bridge, 10 Sep 2011, by AJ Radwell, AJR110105A • 1 ♀ and 1 ♂ from McDonald County, Tiff City, beside Route 43, Buffalo Creek (36°40'17"N, 94°36'17"W), 2 May 1996, by IM Smith, IMS960004 • **New Brunswick, Canada**: 3 ♀ from Charlotte County, Rollingham, Digdegaush River, beside Highway 770, 3 Oct 2011, by IM Smith, IMS110118 • **New Hampshire, USA**: 1 ♀ from Coos County, picnic area beside Route 110, Upper Ammonoosuc River (44°36'N, 71°24'W), 5 Jul 1989, by IM Smith, IMS890071 • **Tennessee, USA**: 1 ♀ and 1 ♂ from Blount County, Great Smokey Mountains National Park, Cades Cove, Forge Creek (35°35'31"N, 83°51'21"W), 17 Sep 2010, by IM Smith, IMS100141 • 1 ♂ from Sevier Co., Great Smokey Mountains National Park, Middle Prong Little Pigeon River (35°43'33"N, 83°24'1"W), 12 Sep 2010, by IM Smith, IMS100131 • **Virginia, USA**: 1 ♀ and 1 ♂ from Amherst County, Upper Otter Creek Overlook beside Blue Ridge, Otter Creek (37°36'57"N, 79°19'27"W), 7 Sep 2007, by IM Smith, IMS070056A • 1 ♂ from Giles County, Mechanicsburg, beside Dismal Creek Road, Standrock Brook (37°11'38"N, 80°53'26"W), 9 Sep 2005, by IM Smith, IMS050066.

######## Type deposition.

Holotype (♀), allotype (♂), and most paratypes (5 ♀; 4 ♂) deposited in the CNC; other paratypes (4 ♀; 3 ♂) deposited in the ACUA.

######## Diagnosis.

*Torrenticola malarkeyorum* are similar to other members of the Rusetria “Eastern 2-Plates” group (*T. biscutella*, *T. caerulea*, *T. delicatexa*, *T. indistincta*, *T. pendula*, *T. sellersorum*, *T. tysoni*, *T. ululata*, *T. whitneyae*, *T. microbiscutella*, and *T. feminellai*) in having anterio-lateral platelets fused to the dorsal plate, having dorsal coloration separated into anterior and posterior portions (except *T. ululata* and *T. indistincta*), and being distributed in the east. *T. malarkeyorum* can have variable coloration, including light bluish purple and reddish purple. Although several other species are purplish, some *T. malarkeyorum* are easily recognizable because they have bluish-purple coloration similar to *T. tysoni* and *T. biscutella*, albeit much fainter than these species. *T. malarkeyorum* can be differentiated from *T. ululata*, *T. indistincta*, and *T. feminellai* by dorsal coloration and pattern. *T. malarkeyorum* can be differentiated from *T. tysoni* by having a stockier rostrum (length/width = 2.57–2.89 in *T. malarkeyorum*, 3.06–3.5 in *T. tysoni*). Female *T. malarkeyorum* can be differentiated from female *T. biscutella* by having a longer subcapitulum (ventral length = 317.5–335 in *T. malarkeyorum*, 290–315 in *T. biscutella*). Male *T. malarkeyorum* can be differentiated from male *T. biscutella* by having a slightly more ovoid dorsum (length/width = 1.42–1.56 in *T. malarkeyorum*; 1.37–1.42 in *T. biscutella*. Additionally, although *T. malarkeyorum* and *T. biscutella* have the same dorsal coloration and pattern, often the coloration is faint in *T. malarkeyorum* and bold in *T. biscutella*. Female *T. malarkeyorum* can be differentiated from female *T. caerulea* by having a wider genital field (152.5–165 in *T. malarkeyorum*, 120–145 in *T. caerulea*). Additionally, *T. malarkeyorum* can be differentiated from *T. caerulea* by dorsal coloration and pattern. *T. malarkeyorum* can be differentiated from *T. pendula* by having a stockier gnathosomal bay (length/width = 1.62–2.26 in *T. malarkeyorum*, 2.42–2.90 in *T. pendula*); more elongate pedipalp tibiae (length/width = 3.22–3.60 in *T. malarkeyorum*, 2.78–3.05 in *T. pendula*); and by dorsal pattern. *T. malarkeyorum* can be differentiated from *T. whitneyae* by having more elongate pedipalpal tibiae (length/width = 3.22–3.60 in *T. malarkeyorum*, 2.42–2.95 in *T. whitneyae*) and by anterior venter/genital field length (♀ = 0.85–0.89 in *T. malarkeyorum*, 0.59–0.75 in *T. whitneyae*; ♂ = 1.52–1.88 in *T. malarkeyorum*, 1.37–1.43 in *T. whitneyae*). *T. malarkeyorum* can be differentiated from *T. microbiscutella* by having a less elongate dorsum (length/width = 1.33–1.56 in *T. malarkeyorum*, 1.63–1.75 in *T. microbiscutella*). Female *T. malarkeyorum* can be differentiated from female *T. delicatexa* by having a shorter genital field (162.5–170 in *T. malarkeyorum*, 175–198 in *T. delicatexa*). Female *T. malarkeyorum* can be differentiated from female *T. sellersorum* by anterior venter/genital field length (0.85–0.89 in *T. malarkeyorum*, 0.69–0.77 in *T. sellersorum*). Male *T. malarkeyorum* do not have any measurement differences with male *T. delicatexa*, and *T. sellersorum*; however, they can be differentiated by dorsal coloration.

######## Description.

**Female (Figure 138)** (n = 5) (holotype measurements in parentheses when available) with characters of the genus with following specifications.

**Dorsum** — (590–640 (635) long; 425–470 (470) wide) ovoid with highly variable coloration, usually faint (occasionally bold) bluish-purple or reddish-purple separated into anterior and posterior portions, and with faint orange medially. Anterio-medial platelets (121.25–135 (132.5) long; 41.25–47.5 (45) wide). Anterio-lateral platelets (150–170 (170) long; 55–70 (70) wide) fused to dorsal plate. Dgl-4 much closer to the edge of the dorsum than to the muscle scars (distance between Dgl-4 320–335 (330)). Dorsal plate proportions: dorsum length/width 1.33–1.41 (1.35); dorsal width/distance between Dgl-4 1.27–1.44 (1.42); anterio-medial platelet length/width 2.68–3.18 (2.94); anterio-lateral platelet length/width 2.38–2.73 (2.43); anterio-lateral/anterio-medial length 1.11–1.36 (1.28).

**Gnathosoma — Subcapitulum** (317.5–335 (335) long (ventral); 234–250 (250) long (dorsal); 135–155 (155) tall) colorless. Rostrum (123.75–130 (130) long; 45–47.5 (45) wide). Chelicerae (323.75–342.5 (342.5) long) with curved fangs (59–70 (70) long). Subcapitular proportions: ventral length/height 2.13–2.35 (2.16); rostrum length/width 2.61–2.89 (2.89). **Pedipalps** with tuberculate ventral extensions on femora and genua. Palpomeres: trochanter (42.5–50 (47.5) long); femur (115–123.75 (120) long); genu (67.5–72.5 (72.5) long); tibia (90–92.5 (90) long; 25–27.5 (27.5) wide); tarsus (17.5–20 (20) long). Palpomere proportions: femur/genu 1.62–1.71 (1.66); tibia/femur 0.73–0.80 (0.75); tibia length/width 3.27–3.60 (3.27).

**Venter** — (700–770 (760) long; 482–600 (600) wide) colorless (occasionally with faint bluish-purple coloration). Gnathosomal bay (165–182.5 (182.5) long; 97.5–110 (110) wide). Cxgl-4 subapical. **Medial suture** absent. **Genital plates** (162.5–170 (170) long; 152.5–165 (165) wide). Additional measurements: Cx-1 (284–330 (330) long (total); 116–145 (145) long (medial)); Cx-3 (316–385 (385) wide); anterior venter (137.5–150 (150) long). Ventral proportions: gnathosomal bay length/width 1.65–1.82 (1.66); anterior venter/genital field length 0.85–0.89 (0.88); anterior venter length/genital field width 0.88–0.95 (0.91).

**Male (Figure 139)** (n = 5) (allotypic measurements in parentheses when available) with characters of the genus with following specifications.

**Dorsum** — (420–470 (470) long; 285–320 (320) wide) ovoid with highly variable coloration, usually faint (occasionally bold) bluish-purple or reddish-purple separated into anterior and posterior portions, and with faint orange medially. Anterio-medial platelets (92.5–110 (110) long; 32.5–37.5 (37.5) wide). Anterio-lateral platelets (117.5–127.5 (127.5) long; 45–52.5 (52.5) wide) fused to dorsal plate. Dgl-4 much closer to the edge of the dorsum than to the muscle scars (distance between Dgl-4 210–240 (240)). Dorsal plate proportions: dorsum length/width 1.42–1.56 (1.47); dorsal width/distance between Dgl-4 1.29–1.36 (1.33); anterio-medial platelet length/width 2.80–3.15 (2.93); anterio-lateral platelet length/width 2.43–2.61 (2.43); anterio-lateral/anterio-medial length 1.12–1.32 (1.33).

**Gnathosoma — Subcapitulum** (227.5–240 (230) long (ventral); 162–180 (163) long (dorsal); 95–100 (100) tall) colorless. Rostrum (90–95 (92.5) long; 32.5–35 (35) wide). Chelicerae (212.5–230 long) with curved fangs (40–50 long). Subcapitular proportions: ventral length/height 2.28–2.53 (2.30); rostrum length/width 2.57–2.85 (2.64). **Pedipalps** with tuberculate ventral extensions on femora and genua. Palpomeres: trochanter (32.5–40 (35) long); femur (76.25–87.5 (87.5) long); genu (52.5–55 (55) long); tibia (65–75 (75) long; 20–22.5 (22.5) wide); tarsus (15–17.5 (17.5) long). Palpomere proportions: femur/genu 1.39–1.62 (1.59); tibia/femur 0.76–0.92 (0.86); tibia length/width 3.22–3.50 (3.33).

**Venter** — (495–560 (560) long; 320–405 (396) wide) colorless (occasionally with faint bluish-purple coloration). Gnathosomal bay (97.5–130 (115) long; 55–65 (65) wide). Cxgl-4 subapical. **Medial suture** (65–72.5 (72.5) long). **Genital plates** (97.5–115 (110) long; 95–120 (105) wide). Additional measurements: Cx-1 (197.5–230 (230) long (total); 90–115 (107) long (medial)); Cx-3 (250–300 (295) wide); anterior venter (160–200 (200) long). Ventral proportions: gnathosomal bay length/width 1.62–2.26 (1.77); anterior venter/genital field length 1.52–1.88 (1.82); anterior venter length/genital field width 1.53–2.00 (1.90); anterior venter/medial suture 2.37–2.92 (2.76).

**Immatures** unknown.

######## Etymology.

Specific epithet (*malarkeyorum*) named in honor of JRF’s sister, Mayme Malarkey and her family—Andy, Jack, Molly, and Lucy—who are a constant joy to all whom they encounter.

######## Distribution.

**Figure 137.**
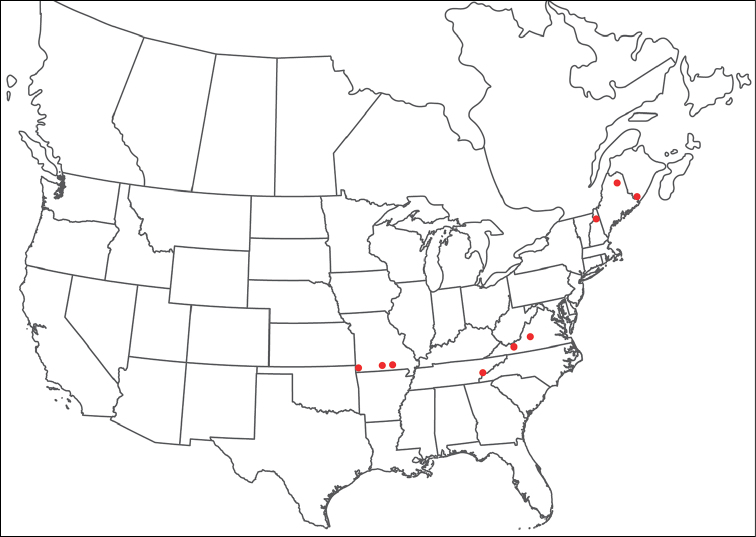
*Torrenticola malarkeyorum* sp. n. distribution.

Eastern (Figure 137), especially highlands (Appalachians and Ozarks).

**Figure 138.**
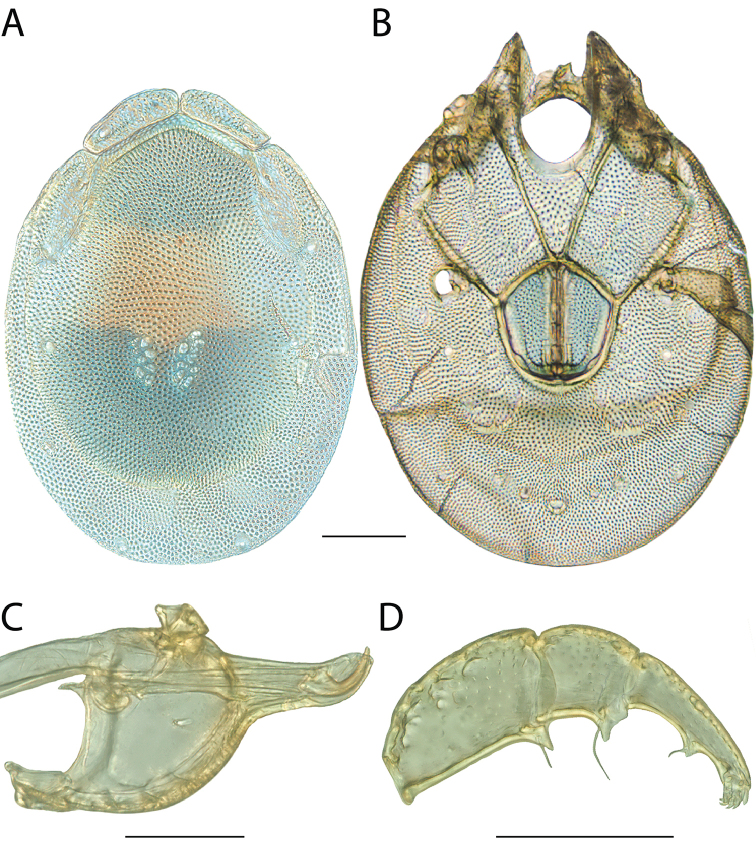
*Torrenticola malarkeyorum* sp. n. female: **A** dorsal plates **B** venter (legs removed) **C** subcapitulum **D** pedipalp (setae not accurately depicted). Scale = 100 µm.

**Figure 139.**
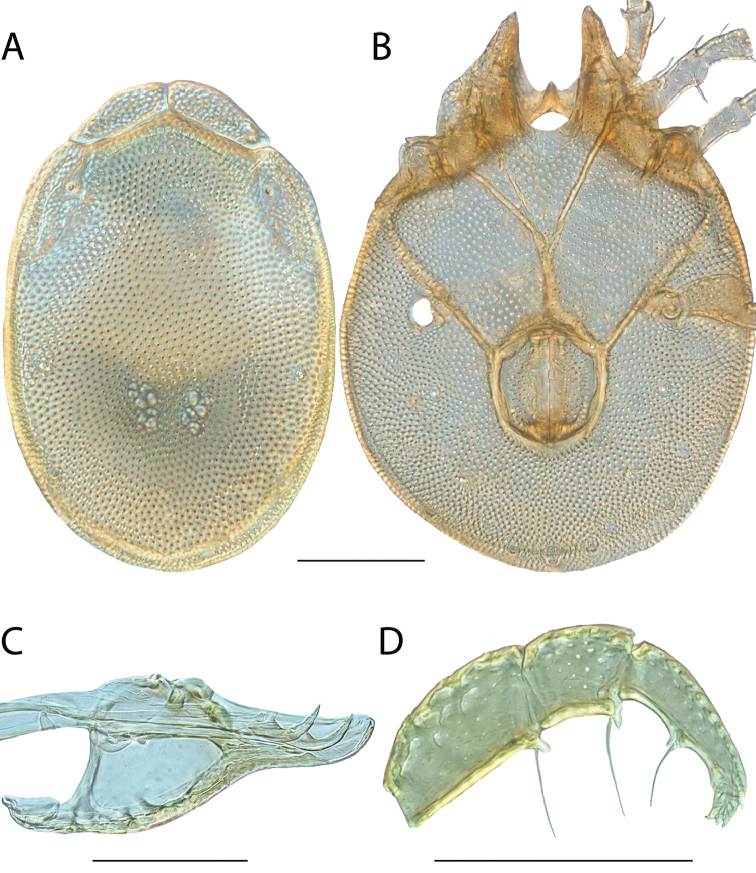
*Torrenticola malarkeyorum* sp. n. male: **A** dorsal plates **B** venter (legs removed) **C** subcapitulum **D** pedipalp (setae not accurately depicted). Scale = 100 µm.

######## Remarks.

*Torrenticola malarkeyorum* groups with other members of the Rusetria Complex with high support and all specimens are less than 2% different in COI sequence from each other. In all analyses, *T. malarkeyorum* groups with two other morphologically similar species: *T. biscutella* and *T. caerulea*. These three species are 3–5% different from each other in COI sequence. The three of these species are morphologically similar to the more distantly-related *T. delicatexa*.

Based upon overall similarity, dorso-lateral platelet fusion, and distribution, we were able to place this species within the Eastern 2-Plate Identification Group.

This species hypothesis is supported by low COI divergence within the species (0–2%) and high divergence between species (3–15%), and by the morphological characters outlined in the diagnosis.

####### Torrenticola manni

Taxon classificationAnimaliaTrombidiformesTorrenticolidae

Fisher & Dowling sp. n.

http://zoobank.org/866CFBA4-9FD6-436E-B0C0-217F0C1C94C1

######## Material examined.

HOLOTYPE (♀): from USA, New Mexico Catron County, Whitewater Creek, Glenwood Whitewater Picnic Area, 5 May 2012, by IM Smith, IMS120005, DNA 2906.

PARATYPES (2 ♀; 4 ♂): **New Mexico, USA**: 1 ♂ (ALLOTYPE) from Catron County, Whitewater Creek, Glenwood Whitewater Picnic Area, 5 May 2012, by IM Smith, IMS120005, DNA 2907 • 1 ♀ and 1 ♂ from Catron County, Whitewater Creek, Glenwood Whitewater Picnic Area, 5 May 2012, by IM Smith, IMS120005 • 1 ♀ and 2 ♂ from Catron County, Whitewater Creek, Glenwood Whitewater Picnic Area, 12 Jul 1987, by IM Smith, IMS870084.

######## Type deposition.

Holotype (♀), allotype (♂), and most paratypes (1 ♀; 2 ♂) deposited in the CNC; other paratypes (1 ♀; 2 ♂) deposited in the ACUA.

######## Diagnosis.

*Torrenticola manni* are similar to members of the Miniforma group (*T. copipalpa*, *T. miniforma*, *T. pacificensis*, *T. rockyensis*, *T. oliveri*, and *T. pinocchio*) in having short, stocky pedipalps (except *T. oliveri* and *T. pinocchio*); similar pedipalpal extensions (unique to members of this group); and being among the smallest *Torrenticola* in the west (dorsum 500–625 long) (except *T. oliveri*). *T. manni* can be differentiated from all other Miniforma group by being from the southwest (all others are from the northwest or Rocky Mountains). *T. manni* are best differentiated from *T. rockyensis* by having more elongate pedipalp tibiae (length/width = 3.13–3.38 in *T. manni*, 2.47–3.11 in *T. rockyensis*). *T. manni* are best differentiated from *T. copipalpa* by having tuberculate pedipalp femoral extensions (broad and flat in *T. copipalpa*). *T. manni* are best differentiated from *T. pacificensis* by having more elongate tibiae (length/width = 3.24–3.38 in *T. manni*, 2.67–3.00 in *T. pacificensis*); having more elongate subcapitular rostra (length/width ♀ = 3.00–3.13 in *T. manni*, 2.59–2.68 in *T. pacificensis*; ♂ = 3.13–3.20 in *T. manni*, 2.76–3.07 in *T. pacificensis*). *T. manni* are best differentiated from *T. miniforma* by being larger (dorsum length ♀ = 565–620 in *T. manni*, 545 in *T. miniforma*; ♂ = 535–550 in *T. manni*, 485 in *T. miniforma*) and having more elongate pedipalp tibiae (length/width = 3.13–3.38 in *T. manni*, 2.38–2.88 in *T. miniforma*). *T. manni* can be differentiated from *T. oliveri* by having a shorter anterior venter (192–230 in *T. manni*, 250–310 in *T. oliveri*) and less elongate pedipalpal tibiae (length/width = 3.13–3.38 in *T. manni*, 3.68–4.13 in *T. oliveri*). *T. manni* can be differentiated from *T. pinocchio* by having a less elongate rostrum (length/width = 3.0–3.2 in *T. manni*, 4.5–4.9 in *T. pinocchio*) and a more ovoid dorsum (length/width = 1.25–1.36 in *T. manni*, 1.53–1.64 in *T. pinocchio*).

######## Description.

**Female (Figure 141)** (n = 3) (holotype measurements in parentheses when available) with characters of the genus with following specifications.

**Dorsum** — (565–620 (620) long; 390–450 (450) wide) ovoid with faint purple coloration restricted posteriorly. Anterio-medial platelets (110–125 (125) long; 52.5–56.25 (56.25) wide). Anterio-lateral platelets (170–182.5 (182.5) long; 62.5–70 (70) wide) free from dorsal plate. Dgl-4 much closer to the edge of dorsum than to the muscle scars (distance between Dgl-4 295–335 (335)). Dorsal plate proportions: dorsum length/width 1.38–1.45 (1.38); dorsal width/distance between Dgl-4 1.32–1.36 (1.34); anterio-medial platelet length/width 2.10–2.22 (2.22); anterio-lateral platelet length/width 2.61–2.81 (2.61); anterio-lateral/anterio-medial length 1.46–1.55 (1.46).

**Gnathosoma — Subcapitulum** (305–337.5 (337.5) long (ventral); 225–255 (255) long (dorsal); 110–121.25 (121.25) tall) colorless. Rostrum (125–140 (140) long; 40–45 (45) wide). Chelicerae (305–332 (332) long) with curved fangs (50–64 (57) long). Subcapitular proportions: ventral length/height 2.77–2.82 (2.78); rostrum length/width 3.00–3.11 (3.11). **Pedipalps** short and stocky (especially tibiae) with broadly tuberculate ventral extensions with dentate tip on femora and dentate, flanged ventral extensions on genua. Palpomeres: trochanter (30–35 (35) long); femur (95–102.5 (102.5) long); genu (65–72.5 (72.5) long); tibia (62.5–70 (68.75) long; 20–21.25 (21.25) wide); tarsus (15–16.25 (16.25) long). Palpomere proportions: femur/genu 1.41–1.46 (1.41); tibia/femur 0.66–0.68 (0.67); tibia length/width 3.13–3.29 (3.24).

**Venter** — (700–780 (780) long; 458–477 (477) wide) colorless. Gnathosomal bay (142.5–150 (150) long; 60–70 (70) wide). Cxgl-4 subapical. **Medial suture** (45–50 (45) long). **Genital plates** (180–192.5 (192.5) long; 160–180 (180) wide). Additional measurements: Cx-1 (255–281 (275) long (total); 120–134 (131) long (medial)); Cx-3 (290–307 (307) wide); anterior venter (192.5–202.5 (202.5) long). Ventral proportions: gnathosomal bay length/width 2.11–2.38 (2.14); anterior venter/genital field length 1.05–1.07 (1.05); anterior venter length/genital field width 1.13–1.23 (1.13); anterior venter/medial suture 3.85–4.50 (4.50).

**Male (Figure 142)** (n = 4) (allotypic measurements in parentheses when available)) with characters of the genus with following specifications.

**Dorsum** — (535–550 (545) long; 375–400 (390) wide) ovoid with faint purple coloration restricted posteriorly. Anterio-medial platelets (105–112.5 (112.5) long; 50–52.5 (52.5) wide). Anterio-lateral platelets (157.5–175 (167.5) long; 62.5–65 (65) wide) free from dorsal plate. Dgl-4 much closer to the edge of dorsum than to the muscle scars (distance between Dgl-4 300–305 (305)). Dorsal plate proportions: dorsum length/width 1.34–1.47 (1.40); dorsal width/distance between Dgl-4 1.25–1.33 (1.28); anterio-medial platelet length/width 2.00–2.15 (2.14); anterio-lateral platelet length/width 2.52–2.80 (2.58); anterio-lateral/anterio-medial length 1.49–1.58 (1.49).

**Gnathosoma — Subcapitulum** (290–305 (297.5) long (ventral); 220–230 (227.5) long (dorsal); 100–110 (107.5) tall) colorless. Rostrum (120–127.5 (127.5) long; 37.5–40 (40) wide). Chelicerae (285–305 (300) long) with curved fangs (45–50 (46) long). Subcapitular proportions: ventral length/height 2.73–2.90 (2.77); rostrum length/width 3.13–3.20 (3.19). **Pedipalps** short and stocky (especially tibiae) with broadly tuberculate ventral extensions with dentate tip on femora and dentate, flanged ventral extensions on genua. Palpomeres: trochanter (27.5–32.5 (32.5) long); femur (87.5–97.5 (92.5) long); genu (60–65 (65) long); tibia (62.5–67.5 (67.5) long; 18.75–20 (20) wide); tarsus (13.75–15 (15) long). Palpomere proportions: femur/genu 1.40–1.50 (1.42); tibia/femur 0.65–0.73 (0.73); tibia length/width 3.19–3.38 (3.38).

**Venter** — (665–695 (695) long; 424.75–490 (424.75) wide) colorless. Gnathosomal bay (115–147.5 (140) long; 55–62.5 (62.5) wide). Cxgl-4 subapical. **Medial suture** (87.5–90 (87.5) long). **Genital plates** (140–150 (150) long; 112.5–116.25 (116.25) wide). Additional measurements: Cx-1 (240–272 (272) long (total); 115–125 (122) long (medial)); Cx-3 (275–315 (278) wide); anterior venter (220–230 (230) long). Ventral proportions: gnathosomal bay length/width 1.92–2.68 (2.24); anterior venter/genital field length 1.52–1.58 (1.53); anterior venter length/genital field width 1.91–1.98 (1.98); anterior venter/medial suture 2.44–2.63 (2.63).

**Immatures** unknown.

######## Etymology.

Specific epithet (*manni*) named in honor of author Charles Mann, whose books about the peopling of North America (e.g., *1491*, *1493*) breach history and venture into human ecology. They are an inspiration to confronting misconceptions and a reminder that even seemingly well-known history, whether archeological or evolutionary, is in fact usually not well-known. His books are a battle cry to never cease learning and to always question.

######## Distribution.

**Figure 140.**
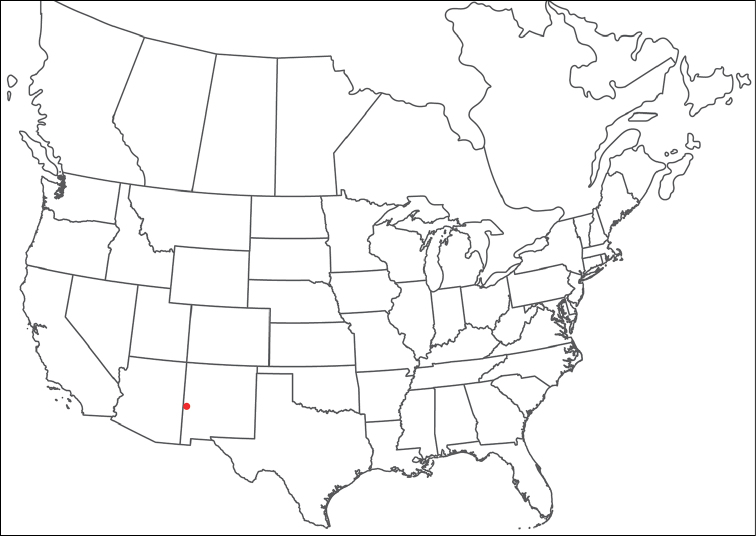
*Torrenticola manni* sp. n. distribution.

Known only from Catron County, New Mexico (Figure 140).

**Figure 141.**
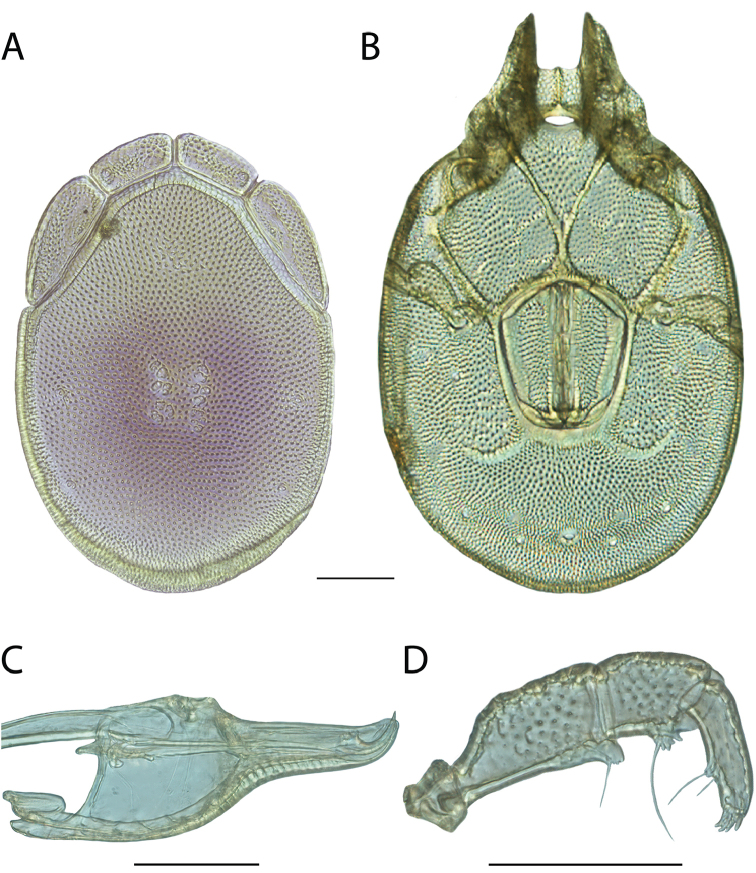
*Torrenticola manni* sp. n. female: **A** dorsal plates **B** venter (legs removed) **C** subcapitulum **D** pedipalp (setae not accurately depicted). Scale = 100 µm.

**Figure 142.**
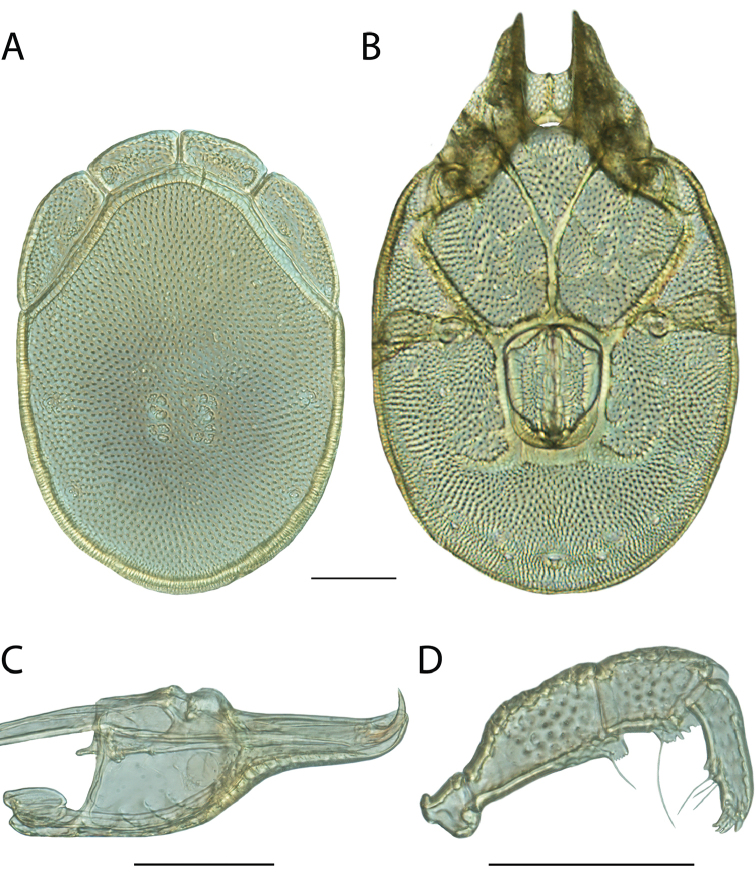
*Torrenticola manni* sp. n. male: **A** dorsal plates **B** venter (legs removed) **C** subcapitulum **D** pedipalp (setae not accurately depicted). Scale = 100 µm.

######## Remarks.

*Torrenticola manni* groups with other members of the Miniforma Complex with high support and specimens of this species are less than 1% different in COI sequence from each other. In all analyses, *T. manni* groups with three other morphologically-similar species with high support: *T. pacificensis*, *T. copipalpa*, and *T. rockyensis*. These species are greater than 4% different from each other in COI sequence. These four species show a higher degree of biogeographic partitioning than most *Torrenticola*.

Based upon overall similarity, the pedipalp genu extensions, and western distribution, we were able to place this species in the Miniforma Identification Group.

This species hypothesis is supported by non-overlapping distribution, low COI divergence within the species (0–2%) and high divergence between species (3–15%), and by the morphological characters outlined in the diagnosis.

####### Torrenticola microbiscutella

Taxon classificationAnimaliaTrombidiformesTorrenticolidae

Fisher & Dowling sp. n.

http://zoobank.org/BA913F73-B71D-4219-9072-71A89B599018

######## Material examined.

HOLOTYPE (♀): from USA, Georgia, White County, Helen; beside Road to Anna Ruby Falls just north of Unicoi State Park, (34°44'44"N, 83°43'43"W), 24 September 1992, by IM Smith, IMS920051.

PARATYPES (5 ♀; 6 ♂): **Georgia, USA**: 1 ♂ (ALLOTYPE) from White County, Helen; beside Road to Anna Ruby Falls just north of Unicoi State Park, (34°44'44"N, 83°43'43"W), 24 September 1992, by IM Smith, IMS920051 • 3 ♀ and 4 ♂ from White County, Helen; beside Road to Anna Ruby Falls just north of Unicoi State Park, (34°44'44"N, 83°43'43"W), 24 September 1992, by IM Smith, IMS920051 • **North Carolina, USA**: • 2 ♀ and 2 ♂ from Macon County, Rainbow Springs; beside Forest Route 67, 2.0 km south of road to Standing Indian Campground, (35°3'3"N, 83°31'31"W), 1 July 1990, by IM Smith, IMS900072.

######## Type deposition.

Holotype (♀), allotype (♂), and some paratypes (3 ♀; 3 ♂) deposited in the CNC; other paratypes (2 ♀; 2 ♂) deposited in the ACUA.

######## Diagnosis.

*Torrenticola microbiscutella* are similar to other members of the Rusetria “Eastern 2-Plates” group (*T. biscutella*, *T. caerulea*, *T. delicatexa*, *T. indistincta*, *T. malarkeyorum*, *T. pendula*, *T. sellersorum*, *T. tysoni*, *T. ululata*, *T. whitneyae*, and *T. feminellai*) in having anterio-lateral platelets fused to the dorsal plate, having dorsal coloration separated into anterior and posterior portions (except *T. ululata* and *T. indistincta*), and being distributed in the east. *T. microbiscutella* can be differentiated from all other Eastern 2-Plates by having an elongate dorsum (length/width = 1.63–1.75 in *T. microbiscutella*, 1.21–1.56 in others). *T. microbiscutella* can be differentiated from most other Eastern 2-Plates by having faint dorsal coloration (most other Eastern 2-Plates have bold coloration).

######## Description.

**Female (Figure 144)** (n = 5) (holotype measurements in parentheses when available) with characters of the genus with following specifications.

**Dorsum** — (490–540 (510) long; 290–325 (300) wide) ovoid and elongate with faint reddish-purple coloration separated into anterior and posterior portions. Anterio-medial platelets (105–112.5 (110) long; 32.5–36.25 (33.75) wide). Anterio-lateral platelets (130–147.5 (135) long; 35–45 (45) wide) fused to dorsal plate. Dgl-4 much closer to the edge of the dorsum than to the muscle scars (distance between Dgl-4 235–250 (235)). Dorsal plate proportions: dorsum length/width 1.65–1.75 (1.70); dorsal width/distance between Dgl-4 1.23–1.30 (1.28); anterio-medial platelet length/width 3.00–3.46 (3.26); anterio-lateral platelet length/width 3.00–3.79 (3.00); anterio-lateral/anterio-medial length 1.20–1.34 (1.23).

**Gnathosoma — Subcapitulum** (260–270 (270) long (ventral); 195–210 (210) long (dorsal); 105–110 (107.5) tall) colorless. Rostrum (102.5–107.5 (107.5) long; 40–42.5 (42.5) wide) conical. Chelicerae (255–265 (262.5) long) with curved fangs (55–55 (55) long). Subcapitular proportions: ventral length/height 2.45–2.57 (2.51); rostrum length/width 2.53–2.63 (2.53). **Pedipalps** with tuberculate ventral extensions on femora and genua. Palpomeres: trochanter (35–37.5 (37.5) long); femur (92.5–97.5 (97.5) long); genu (55–60 (57.5) long); tibia (72.5–80 (75) long; 20–22.5 (21.25) wide); tarsus (15–17.5 (17.5) long). Palpomere proportions: femur/genu 1.57–1.70 (1.70); tibia/femur 0.77–0.84 (0.77); tibia length/width 3.53–3.65 (3.53).

**Venter** — (610–660 (620) long; 335–370 (370) wide) colorless. Gnathosomal bay (120–142.5 (120) long; 65–75 (75) wide). Cxgl-4 subapical. **Medial suture** (42.5–50 (45) long). **Genital plates** (135–150 (145) long; 117.5–125 (120) wide). Additional measurements: Cx-1 (220–240 (230) long (total); 95–105 (105) long (medial)); Cx-3 (235–270 (270) wide); anterior venter (150–160 (157.5) long). Ventral proportions: gnathosomal bay length/width 1.60–2.04 (1.60); anterior venter/genital field length 1.07–1.15 (1.09); anterior venter length/genital field width 1.25–1.33 (1.31); anterior venter/medial suture 3.10–3.76 (3.50).

**Male (Figure 145)** (n = 5) (allotypic measurements in parentheses when available) with characters of the genus with following specifications.

**Dorsum** — (430–455 (440) long; 260–280 (260) wide) ovoid and elongate with faint reddish-purple coloration separated into anterior and posterior portions. Anterio-medial platelets (82.5–101.25 (95) long; 31.25–35 (35) wide). Anterio-lateral platelets (115–127.5 (115) long; 35–40 (37.5) wide) fused to dorsal plate. Dgl-4 much closer to the edge of the dorsum than to the muscle scars (distance between Dgl-4 205–215 (205)). Dorsal plate proportions: dorsum length/width 1.63–1.69 (1.69); dorsal width/distance between Dgl-4 1.21–1.30 (1.27); anterio-medial platelet length/width 2.64–3.12 (2.71); anterio-lateral platelet length/width 3.07–3.64 (3.07); anterio-lateral/anterio-medial length 1.21–1.45 (1.21).

**Gnathosoma — Subcapitulum** (215–235 (232.5) long (ventral); 162.5–177.5 (176.25) long (dorsal); 77.5–90 (87.5) tall) colorless. Rostrum (82.5–95 (92.5) long; 30–36.25 (35) wide) conical. Chelicerae (197.5–232.5 (212.5) long) with curved fangs (45–47.5 (47.5) long). Subcapitular proportions: ventral length/height 2.58–2.77 (2.66); rostrum length/width 2.62–2.85 (2.64). **Pedipalps** with tuberculate ventral extensions on femora and genua. Palpomeres: trochanter (30–32.5 (32.5) long); femur (75–81.25 (76.25) long); genu (50–53.75 (50) long); tibia (66.25–70 (67.5) long; 18.75–20 (20) wide); tarsus (15–17.5 (15) long). Palpomere proportions: femur/genu 1.50–1.60 (1.53); tibia/femur 0.84–0.90 (0.89); tibia length/width 3.31–3.60 (3.38).

**Venter** — (530–575 (540) long; 270–310 (305) colorless. Gnathosomal bay (110–125 (110) long; 55–60 (60) wide). Cxgl-4 subapical. **Medial suture** (75–90 (90) long). **Genital plates** (85–95 (90) long; 85–100 (85) wide). Additional measurements: Cx-1 (200–220 (205) long (total); 95–100 (100) long (medial)); Cx-3 (220–240 (240) wide); anterior venter (185–195 (195) long). Ventral proportions: gnathosomal bay length/width 1.83–2.18 (1.83); anterior venter/genital field length 2.00–2.29 (2.17); anterior venter length/genital field width 1.95–2.29 (2.29); anterior venter/medial suture 2.17–2.47 (2.17).

**Immatures** unknown.

######## Etymology.

Specific epithet (*microbiscutella*) is named because this species is the smallest (at least in females) and most elongate of all members of the Rusetria Complex (*mikrós*, G. small; *bi*, L. two; *scutella*, L. little plate).

######## Distribution.

**Figure 143.**
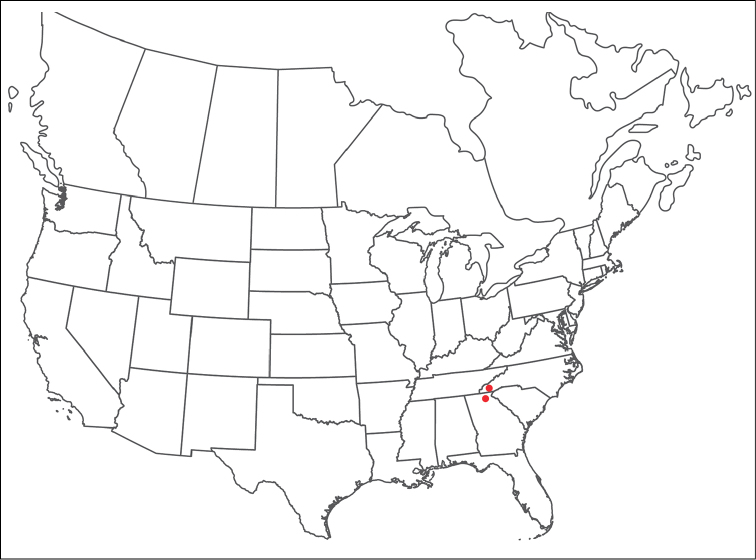
*Torrenticola microbiscutella* sp. n. distribution.

Southern Appalachians (Figure 143).

**Figure 144.**
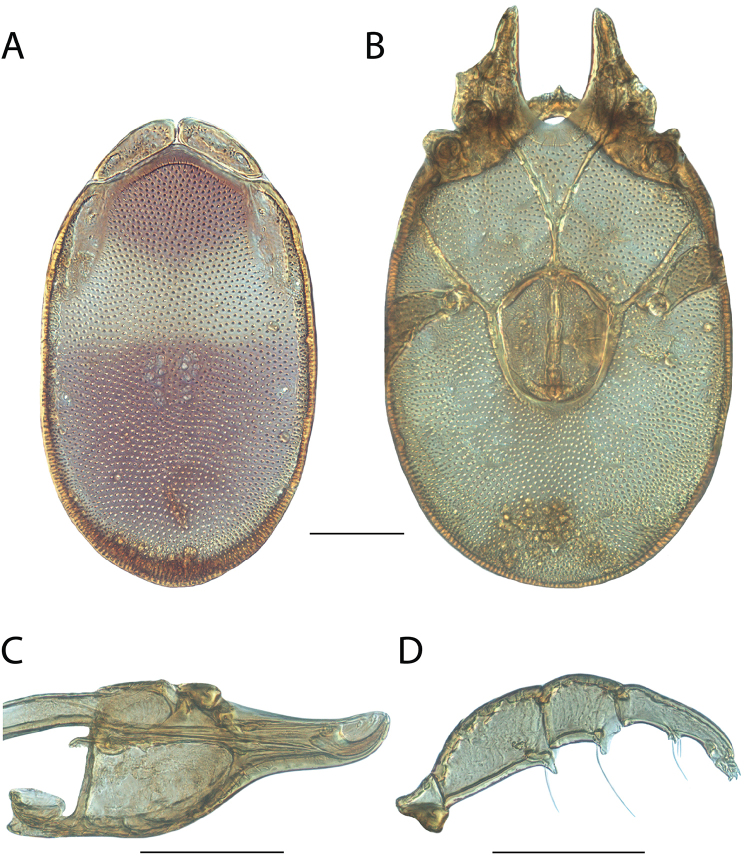
*Torrenticola microbiscutella* sp. n. female: **A** dorsal plates **B** venter (legs removed) **C** subcapitulum **D** pedipalp (setae not accurately depicted). Scale = 100 µm.

**Figure 145.**
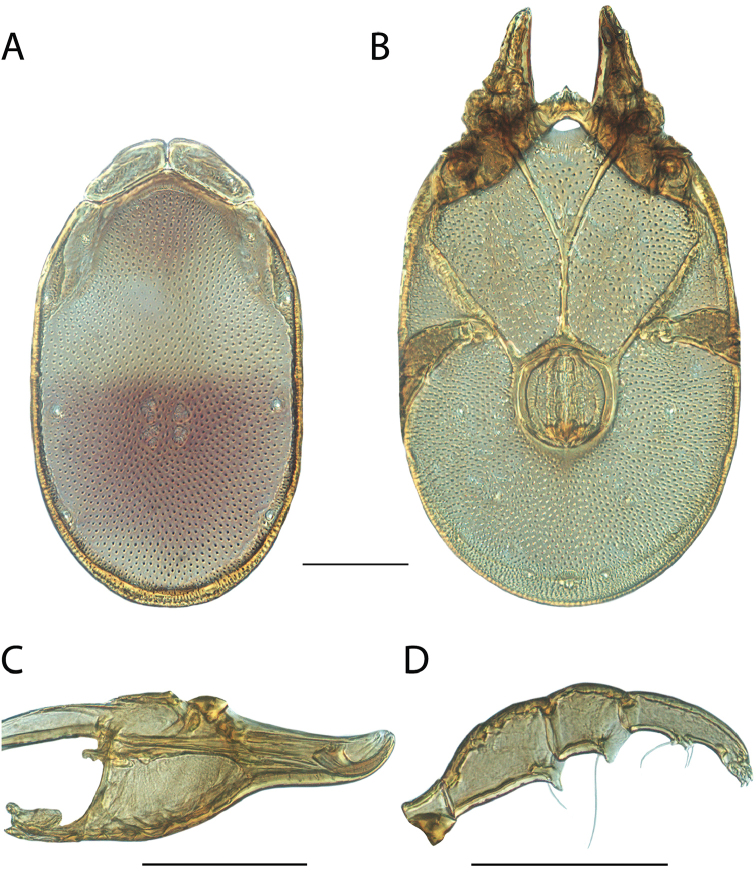
*Torrenticola microbiscutella* sp. n. male: **A** dorsal plates **B** venter (legs removed) **C** subcapitulum **D** pedipalp (setae not accurately depicted). Scale = 100 µm.

######## Remarks.

Unfortunately, we were unable to acquire fresh material of *Torrenticola microbiscutella* and therefore this species is not included in our phylogenetic analyses. However, we were able to examine morphology with material preserved in GAW. The overall similarity, distribution in the east, and fusion of the dorso-lateral platelets to the dorsal plate, are consistent with placing this species in the Rusetria Complex and Eastern 2-Plate Identification Group.

####### Torrenticola miniforma

Taxon classificationAnimaliaTrombidiformesTorrenticolidae

Habeeb, 1974

Torrenticola miniforma Habeeb, 1974: 1.

######## Material examined.

LECTOTYPE (1 ♀): from USA, California, Humboldt County, Prairie Creek Redwoods State Park, Prairie Creek, 12 Jul 1964, by H Habeeb, HH640021.

PARALECTOTYPE (1 ♂): from USA, California, Humboldt County, Prairie Creek Redwoods State Park, Prairie Creek, 12 Jul 1964, by H Habeeb, HH640021.

######## Type deposition.

Types (1 ♀; 1 ♂) deposited in the CNC.

######## Diagnosis.

*Torrenticola miniforma* are similar to members of the Miniforma group (*T. copipalpa*, *T. manni*, *T. pacificensis*, *T. rockyensis*, *T. oliveri*, and *T. pinocchio*) in having short, stocky pedipalps (except *T. oliveri* and *T. pinocchio*); similar pedipalpal extensions (unique to members of this group); and being among the smallest *Torrenticola* in the west (dorsum 500–625 long) (except *T. oliveri*). *T. miniforma* are best differentiated from *T. rockyensis* by being smaller (dorsum length ♀ = 545 in *T. miniforma*, 570–620 in *T. rockyensis*; ♂ = 485 in *T. miniforma*, 525–545 in *T. rockyensis*); having more elongate subcapitular rostra (length/width ♀ = 3.13 in *T. miniforma*, 2.72–2.91 in *T. rockyensis*; ♂ = 3.19 in *T. miniforma*, 2.83–3.00 in *T. rockyensis*); and by being only known from Humboldt County, California, whereas *T. rockyensis* is distributed in the Rocky Mountains (Idaho & Montana). *T. miniforma* are best differentiated from *T. copipalpa* by having tuberculate pedipalp femoral extensions (broad and flat in *T. copipalpa*). *T. miniforma* are best differentiated from *T. pacificensis* by having more elongate anterio-medial platelets (length/width = 2.44–2.65 in *T. miniforma*, 2.00–2.16 in *T. pacificensis*); more elongate subcapitular rostra (length/width = 3.13–3.19 in *T. miniforma*, 2.59–3.07 in *T. pacificensis*); and by being only known from Humboldt County, California, whereas *T. pacificensis* is distributed in the Pacific Coast Ranges of Washington and Oregon. *T. miniforma* are best differentiated from *T. manni* by being smaller (dorsum length ♀ = 545 in *T. miniforma*, 565–620 in *T. manni*; ♂ = 485 in *T. miniforma*, 535–550 in *T. manni*); having stockier pedipalp tibiae (length/width = 2.38–2.88 in *T. miniforma*, 3.13–3.38 in *T. manni*); and by being only known from Humboldt County, California, whereas *T. manni* is only known from Catron County, New Mexico. *T. miniforma* can be differentiated from *T. oliveri* by having a shorter anterior venter (172–200 in *T. miniforma*, 250–310 in *T. oliveri*) and having less elongate pedipalpal tibiae (length/width = 2.3–2.9 in *T. miniforma*, 3.6–4.2 in *T. oliveri*). *T. miniforma* can be differentiated from *T. pinocchio* by having a less elongate rostrum (length/width = 3.1–3.2 in *T. miniforma*, 4.5–4.9 in *T. pinocchio*) and a more ovoid dorsum (length/width = 1.43–1.43 in *T. miniforma*, 1.53–1.64 in *T. pinocchio*).

######## Re-description.

**Female (Figure 147)** (n = 1) (lectotype only) with characters of the genus with following specifications.

**Dorsum** — (545 long; 380 wide) ovoid and colorless. Anterio-medial platelets (112.5 long; 42.5 wide). Anterio-lateral platelets (152.5 long; 50 wide) free from dorsal plate. Dgl-4 much closer to the edge of the dorsum than to the muscle scars (distance between Dgl-4 300). Dorsal plate proportions: dorsum length/width 1.43; dorsal width/distance between Dgl-4 1.27; anterio-medial platelet length/width 2.65; anterio-lateral platelet length/width 3.05; anterio-lateral/anterio-medial length 1.36.

**Gnathosoma — Subcapitulum** (295 long (ventral); 225 long (dorsal); 112.5 tall) colorless. Rostrum (117.5 long; 37.5 wide). Chelicerae (290 long) with curved fangs (52.5 long). Subcapitular proportions: ventral length/height 2.62; rostrum length/width 3.13. **Pedipalps** short and stocky (especially tibiae) with tuberculate ventral extensions on femora and dentate, flanged ventral extensions on genua. Palpomeres: trochanter (32.5 long); femur (90 long); genu (62.5 long); tibia (57.5 long; 20 wide); tarsus (15 long). Palpomere proportions: femur/genu 1.44; tibia/femur 0.64; tibia length/width 2.88.

**Venter** — (680 long; 420 wide) colorless. Gnathosomal bay (145 long; 62.5 wide). Cxgl-4 subapical. **Medial suture** (50 long). **Genital plates** (167.5 long; 165 wide). Additional measurements: Cx-1 (260 long (total); 50 long (medial)); Cx-3 (280 wide); anterior venter (172.5 long). Ventral proportions: gnathosomal bay length/width 2.32; anterior venter/genital field length 1.03; anterior venter length/genital field width 1.05; anterior venter/medial suture 3.45.

**Male (Figure 148)** (n = 1) (paralectotype only) with characters of the genus with following specifications.

**Dorsum** — (485 long; 340 wide) ovoid and colorless. Anterio-medial platelets (97.5 long; 40 wide). Anterio-lateral platelets (145 long; 47.5 wide) free from dorsal plate. Dgl-4 much closer to the edge of the dorsum than to the muscle scars (distance between Dgl-4 270). Dorsal plate proportions: dorsum length/width 1.43; dorsal width/distance between Dgl-4 1.26; anterio-medial platelet length/width 2.44; anterio-lateral platelet length/width 3.05; anterio-lateral/anterio-medial length 1.49.

**Gnathosoma — Subcapitulum** (265 long (ventral); 200 long (dorsal); 95 tall) colorless. Rostrum (107.5 long; 33.75 wide). Chelicerae (265 long) with curved fangs (47.5 long). Subcapitular proportions: ventral length/height 2.79; rostrum length/width 3.19. **Pedipalps** short and stocky (especially tibiae) with tuberculate ventral extensions on femora and dentate, flanged ventral extensions on genua. Palpomeres: trochanter (30 long); femur (77.5 long); genu (52.5 long); tibia (47.5 long; 20 wide); tarsus (15 long). Palpomere proportions: femur/genu 1.48; tibia/femur 0.61; tibia length/width 2.38.

**Venter** — (610 long; 380 wide) colorless. Gnathosomal bay (125 long; 50 wide). Cxgl-4 subapical. **Medial suture** (87.5 long). **Genital plates** (125 long; 50 wide). Additional measurements: Cx-1 (227.5 long (total); 102.5 long (medial)); Cx-3 (250 wide); anterior venter (200 long). Ventral proportions: gnathosomal bay length/width 2.50; anterior venter/genital field length 1.54; anterior venter length/genital field width 1.86; anterior venter/medial suture 2.29.

**Immatures** unknown.

######## Etymology.

Habeeb (1974) did not specify an etymology for the specific epithet (*miniforma*). However, we suspect the specific epithet refers to the small body size of this species, as with others of the Miniforma Group (*minor*, L. small; *forma*, L. form), as Habeeb (1974) notes this species is “the smallest species that has come to the attention of the writer from the pacific area.”

######## Distribution.

**Figure 146.**
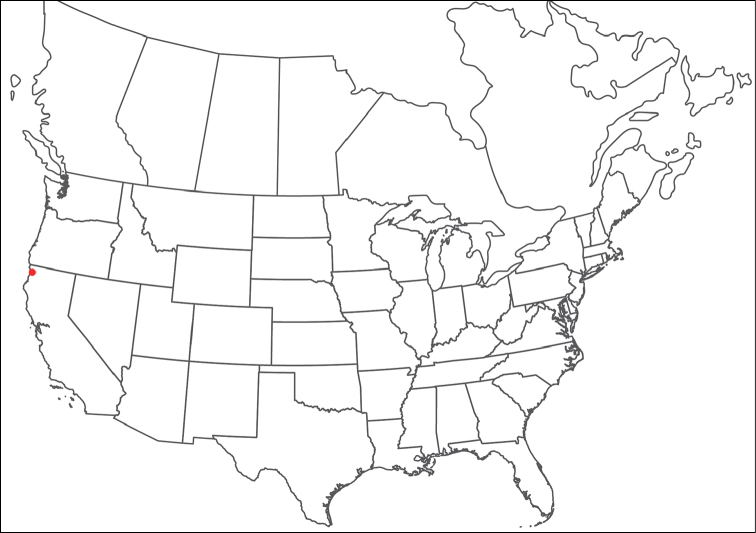
*Torrenticola miniforma* distribution.

Known only from Prairie Creek, Humboldt County, California (Habeeb 1974) (Figure 146).

**Figure 147.**
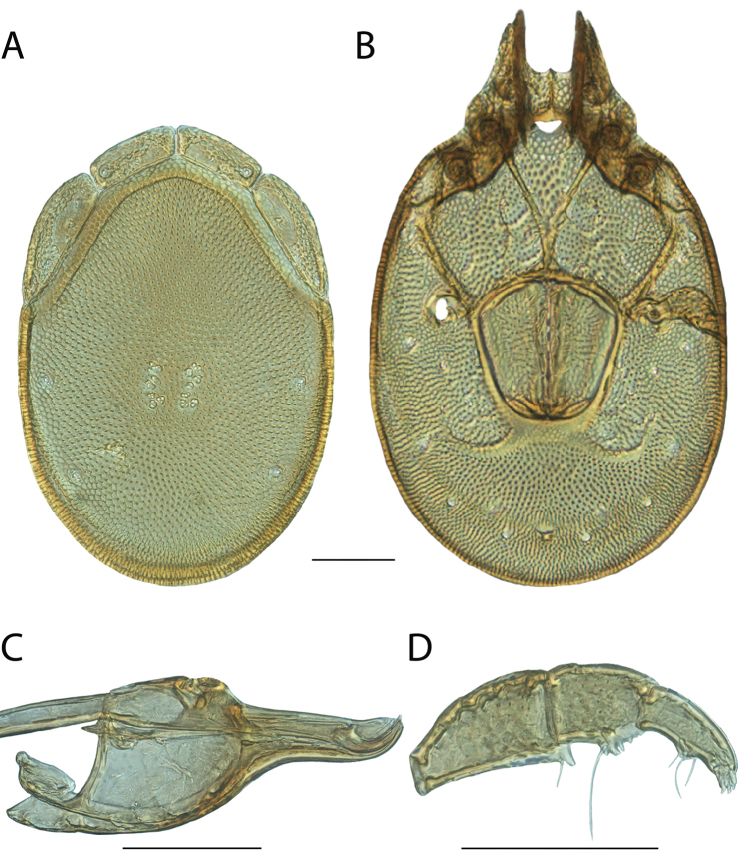
*Torrenticola miniforma* female: **A** dorsal plates **B** venter (legs removed) **C** subcapitulum **D** pedipalp (setae not accurately depicted). Scale = 100 µm.

**Figure 148.**
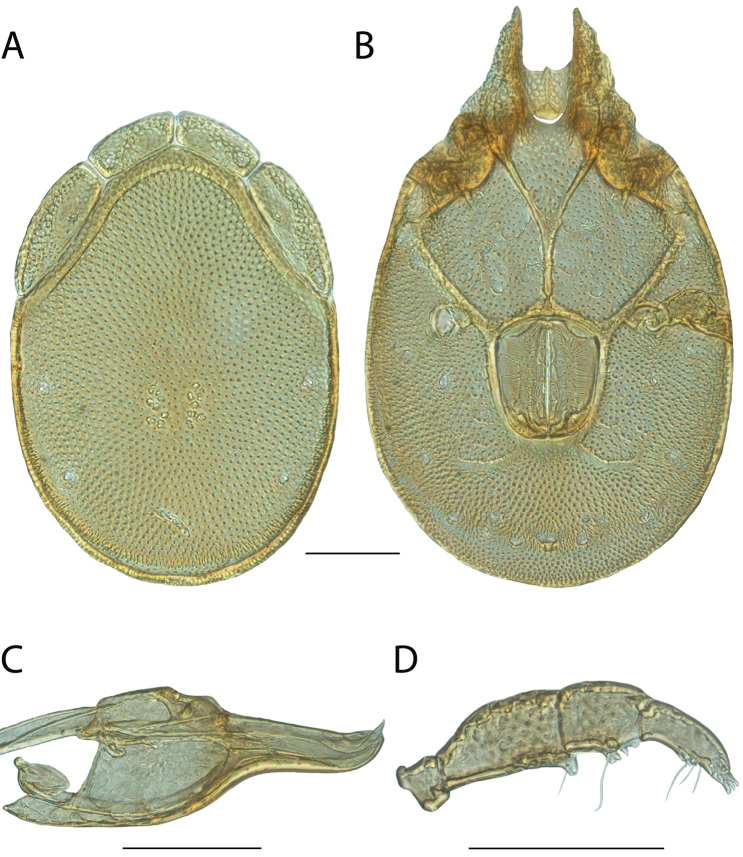
*Torrenticola miniforma* male: **A** dorsal plates **B** venter (legs removed) **C** subcapitulum **D** pedipalp (setae not accurately depicted). Scale = 100 µm.

######## Remarks.

Unfortunately, we were unable to acquire more specimens of *Torrenticola miniforma* and therefore this species is not included in our phylogenetic analyses. However, we were able to examine morphology with the type material. Based upon overall similarity, the pedipalp genu extensions, and western distribution, we were able to place this species in the Miniforma Complex and the Miniforma Identification Group.

####### Torrenticola mjolniri

Taxon classificationAnimaliaTrombidiformesTorrenticolidae

Fisher & Dowling sp. n.

http://zoobank.org/E5E69529-22FE-4357-9FD8-7B96FD917A16

######## Material examined.

HOLOTYPE (♀): from Canada, Ontario, Hastings County, Maple Leaf, Papineau Creek, beside Hwy 62, 18 Aug 2011, by IM Smith, IMS110054, DNA 2860.

PARATYPES (12 ♀; 12 ♂): **Maine, USA**: 1 ♀ and 1 ♂ from Kennebec County, China, beside Causeway along northern shore of China Lake (44°28'45"N, 69°30'43"W), 16 Sep 2003, by IM Smith, IMS030037 • **Ontario, Canada**: 1 ♂ from Frontenac County, Salmon River, beside Highway 7 north of Arden, 26-28 Aug 1982, by IM Smith, CJ Hill, & C Cramer, IMS820005 • 1 ♂ from Hastings County, Duff Corners, Vanderwater Conservation (44°23'14"N, 77°19'1"W), 13 Aug 2009, by IM Smith & ML MacKenzie, IMS090089A • 1 ♂ (ALLOTYPE) from Hastings County, Maple Leaf, Papineau Creek, beside Hwy 62, 18 Aug 2011, by IM Smith, IMS110054, DNA 2857 • 1 ♀ and 1 ♂ from Hastings County, Maple Leaf, Papineau Creek, beside Hwy 62, 18 Aug 2011, by IM Smith, IMS110054 • 6 ♀ and 5 ♂ from Hastings County, Maynooth, Papineau Creek, beside Highway 127, 17 Aug 2011, by IM Smith, IMS110050 & IMS110051 • 1 ♀ and 1 ♂ from Hastings County, Price Conservation Area, Skootamata River, junction of Highways 7 and 37, 20 May 1980, by IM Smith, IMS800009 • 1 ♀ from Muskoka District, Baysville, Echo Lake, bay near Lawsons Cottages, 9-12 Aug 1980, by IM Smith, IMS800015 • 1 ♀ from Muskoka District, Baysville, Echo Lake, 18 Aug 1981, by IM Smith, IMS810023 • **Quebec, Canada**: 1 ♀ and 1 ♂ from Gatineau County, Gatineau Park, Lac Philippe, at campground, 2 Jun 1981, by IM Smith & CJ Hill, IMS810001.

######## Type deposition.

Holotype (♀), allotype (♂), and most paratypes (7 ♀; 7 ♂) deposited in the CNC; other paratypes (5 ♀; 5 ♂) deposited in the ACUA.

######## Diagnosis.

*Torrenticola mjolniri* are similar to other members of the Raptor Group (*T. gnoma*, *T. irapalpa*, *T. longitibia*, *T. elusiva*, *T. racupalpa*, *T. raptor*, *T. danielleae*, *T. daemon*, and *T. ivyae*) in having round bodies; Dgl-4 close to muscles scars; long, thin subcapitular rostra; and long, thin pedipalp tibiae. *T. mjolniri* can be differentiated from *T. elusiva*, *T. gnoma*, and *T. daemon* by having more elongate pedipalp tibiae (length/width = 5.00–6.00 in *T. mjolniri*, 3.88–4.67 in others) and a more elongate rostrum (length/width = 3.81–4.32 in *T. mjolniri*, 2.56–3.65 in others). *T. mjolniri* can be differentiated from *T. racupalpa* by having a longer anterior venter (♀ = 180–195 in *T. mjolniri*, 152.5–165 in *T. racupalpa*; ♂ = 230–255 in *T. mjolniri*, 200–205 in *T. racupalpa*) and by dorsal pattern. *T. mjolniri* can be differentiated from *T. irapalpa* and *T. danielleae* by having Dgl-4 closer to the muscle scars (dorsal width/distance between Dgl-4 = 2.2–2.48 in *T. mjolniri*, 1.42–2.09 in others); having a more elongate rostrum (3.81–4.32 in *T. mjolniri*, 2.66–3.75 in others); and by dorsal pattern. Male *T. mjolniri* can be differentiated from *T. longitibia* (only males) by having Dgl-4 closer to the edge of dorsum (dorsal width/distance between Dgl-4 = 2.2–2.32 in *T. mjolniri*, 2.46–2.71 in *T. longitibia*); stockier pedipalp tibiae (length/width = 5.0–5.33 in *T. mjolniri*, 5.5–5.5 in *T. longitibia*); and a more elongate subcapitulum (ventral length/height = 2.82–3.0 in *T. mjolniri*, 2.68–2.73 in *T. longitibia*). *T. mjolniri* can be differentiated from *T. raptor* by having Dgl-4 closer to the muscle scars (dorsal width/distance between Dgl-4 = 2.2–2.48 in *T. mjolniri*, 1.68–2.02 in *T. raptor*) and a stockier subcapitulum (ventral length/width ♀ = 2.68–2.9 in *T. mjolniri*, 2.98–3.18 in *T. raptor*; ♂ = 2.82–3.0 in *T. mjolniri*, 3.13–3.27 in *T. raptor*). Additionally, female *T. mjolniri* have a shorter anterior venter (180–195 in ♀ *T. mjolniri*, 205–240 in ♀ *T. raptor*). Female *T. mjolniri* can be differentiated from female *T. ivyae* by having a larger dorsum (length ♀ = 605–640 in *T. mjolniri*, 550–590 in *T. ivyae*; width ♀ = 510–545 in *T. mjolniri*, 460–500 in *T. ivyae*) and a longer anterior venter (♀ 180–195 in *T. mjolniri*, 155–170 in *T. ivyae*). Male *T. mjolniri* can be differentiated from male *T. ivyae* by having a more elongate subcapitulum (ventral length/width = 2.82–3.00 in *T. mjolniri*, 2.57–2.75 in *T. ivyae*). Additionally, *T. mjolniri* can be differentiated from *T. ivyae* by being distributed in the northeast (*T. ivyae* is known from Florida).

######## Description.

**Female (Figure 150)** (n = 5) (holotype measurements in parentheses when available) with characters of the genus with following specifications.

**Dorsum** — (605–640 (605) long; 510–545 (520) wide) circular with navy blue to purple coloration posteriorly and a small spot anteriorly often connected medially with a thin strip that is occasionally orange. Anterio-medial platelets (130–150 (135) long; 67.5–70 (67.5) wide). Anterio-lateral platelets (182.5–200 (187.5) long; 67.5–70 (67.5) wide) free from dorsal plate. Dgl-4 closer to the muscle scars than to edge dorsum (distance between Dgl-4 207.5–245 (210)). Dorsal plate proportions: dorsum length/width 1.16–1.24 (1.16); dorsal width/distance between Dgl-4 2.22–2.48 (2.48); anterio-medial platelet length/width 1.86–2.14 (2.00); anterio-lateral platelet length/width 2.11–2.38 (2.27); anterio-lateral/anterio-medial length 1.22–1.46 (1.39).

**Gnathosoma — Subcapitulum** (342.5–355 (342.5) long (ventral); 255–277 (262.5) long (dorsal); 122.5–128.75 (126.25) tall) colorless. Rostrum (142.5–151.25 (151.25) long; 35–38.75 (35) wide). Chelicerae (355–383 (360) long) with curved fangs (52.5–55 (52.5) long) elongate. Subcapitular proportions: ventral length/height 2.68–2.90 (2.71); rostrum length/width 3.81–4.32 (4.32). **Pedipalps** elongate (especially tibiae) with tuberculate ventral extensions on femora and genua. Palpomeres: trochanter (47.5–47.5 (47.5) long); femur (130–135 (130) long); genu (65–70 (65) long); tibia (110–112.5 (112.5) long; 18.75–22.5 (18.75) wide); tarsus (15–17.5 (15) long). Palpomere proportions: femur/genu 1.86–2.00 (2.00); tibia/femur 0.83–0.87 (0.87); tibia length/width 5.00–6.00 (6.00).

**Venter** — (780–825 (790) long; 536–621 (570) wide) often colorless, occasionally areas surrounding coxae are navy blue to purple. Gnathosomal bay (170–175 (175) long; 70–82.5 (70) wide). Cxgl-4 far from apex. **Medial suture** (15–20 (15) long). **Genital plates** (160–173.75 (170) long; 142.5–152.5 (142.5) wide). Additional measurements: Cx-1 (305–330 long (total); 132–164 (162.5) long (medial)); Cx-3 (332–383 wide); anterior venter (180–195 (187.5) long). Ventral proportions: gnathosomal bay length/width 2.06–2.50 (2.50); anterior venter/genital field length 1.07–1.22 (1.10); anterior venter length/genital field width 1.18–1.32 (1.32); anterior venter/medial suture 9.00–12.50 (12.50).

**Male (Figure 151)** (n = 6) (allotypic measurements in parentheses when available) with characters of the genus with following specifications.

**Dorsum** — (520–580 (520) long; 430–450 (430) wide) circular with navy blue to purple coloration posteriorly and a small spot anteriorly often connected medially with a thin strip that is occasionally orange. Anterio-medial platelets (120–135 (122.5) long; 60–67.5 (60) wide). Anterio-lateral platelets (175–200 (185) long; 67.5–77.5 (70) wide) free from dorsal plate. Dgl-4 closer to the muscle scars than to edge dorsum (distance between Dgl-4 185–205 (195)). Dorsal plate proportions: dorsum length/width 1.21–1.29 (1.21); dorsal width/distance between Dgl-4 2.20–2.32 (2.21); anterio-medial platelet length/width 1.85–2.04 (2.04); anterio-lateral platelet length/width 2.34–2.67 (2.64) anterio-lateral/anterio-medial length 1.40–1.51 (1.51).

**Gnathosoma — Subcapitulum** (305–325 (305) long (ventral); 233–241 (233) long (dorsal); 105–112.5 (107.5) tall) colorless. Rostrum (130–140 (130) long; 32.5–32.5 (32.5) wide). Chelicerae (297–322 (297) long) with curved fangs (48–55 (49) long) elongate. Subcapitular proportions: ventral length/height 2.82–3.00 (2.84); rostrum length/width 4.00–4.31 (4.00). **Pedipalps** elongate (especially tibiae) with tuberculate ventral extensions on femora and genua. Palpomeres: trochanter (42.5–42 (42.5) long); femur (115–122.5 (117.5) long); genu (60–65 (65) long); tibia (100–102.5 (102.5) long; 18.75–20 (20) wide); tarsus (17.5–17.5 (17.5) long). Palpomere proportions: femur/genu 1.81–1.96 (1.81); tibia/femur 0.82–0.87 (0.87); tibia length/width 5.00–5.33 (5.13).

**Venter** — (670–740 (670) long; 459–504 (479) wide) often colorless, occasionally areas surrounding coxae are navy blue to purple. Gnathosomal bay (142.5–160 (145) long; 60–67.5 (67.5) wide). Cxgl-4 far from apex. **Medial suture** (62.5–85 (62.5) long). **Genital plates** (137.5–147.5 (137.5) long; 115–142.5 (115) wide). Additional measurements: Cx-1 (275–324 (287) long (total); 139–152 (139) long (medial)); Cx-3 (307–371 (324) wide); anterior venter (230–255 (230) long). Ventral proportions: gnathosomal bay length/width 2.15–2.46 (2.15); anterior venter/genital field length 1.60–1.76 (1.67); anterior venter length/genital field width 1.79–2.00 (2.00); anterior venter/medial suture 3.00–3.68 (3.68).

**Immatures** unknown.

######## Etymology.

Specific epithet (*mjolniri*) named after Mjölnir—the hammer of Thor in Norse mythology—the ancient symbol of which resembles the dorsal patterning this species.

######## Distribution.

**Figure 149.**
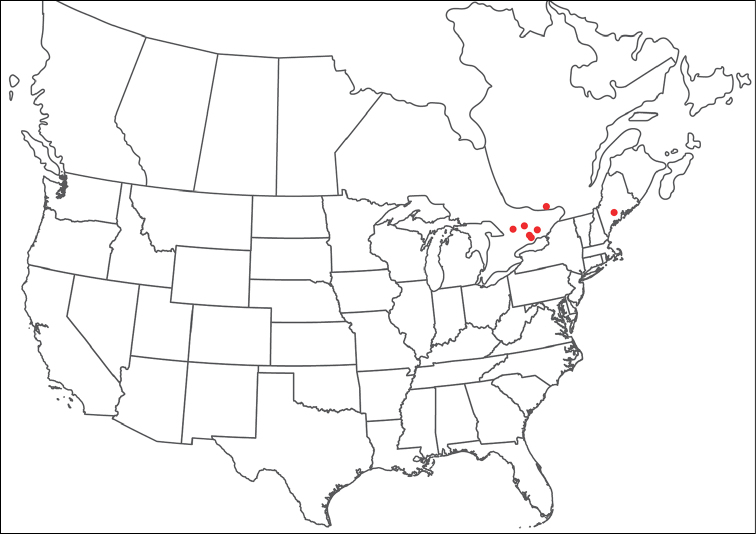
*Torrenticola mjolniri* sp. n. distribution.

Northeastern (Figure 149).

**Figure 150.**
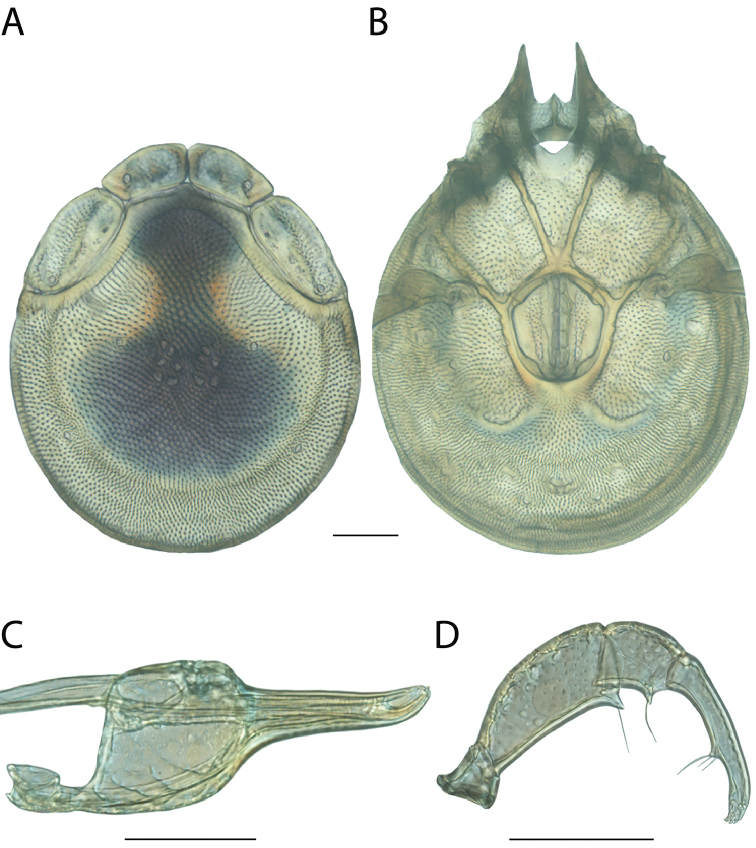
*Torrenticola mjolniri* sp. n. female: **A** dorsal plates **B** venter (legs removed) **C** subcapitulum **D** pedipalp (setae not accurately depicted). Scale = 100 µm.

**Figure 151.**
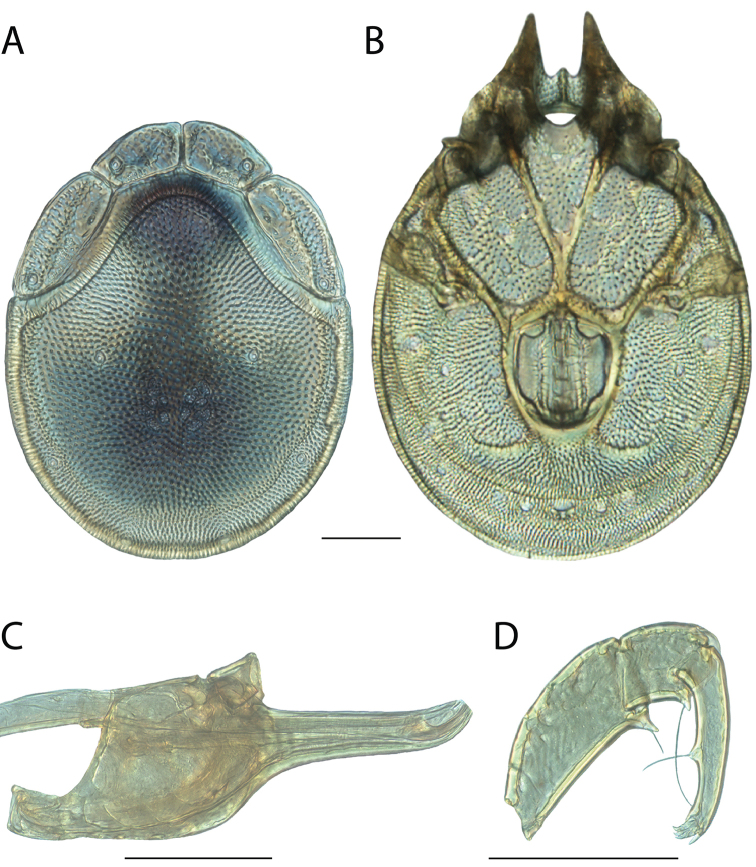
*Torrenticola mjolniri* sp. n. male: **A** dorsal plates **B** venter (legs removed) **C** subcapitulum **D** pedipalp (setae not accurately depicted). Scale = 100 µm.

######## Remarks.

*Torrenticola mjolniri* groups with other members of the Raptor Complex with high support and all specimens are less than 2% different in COI sequence from each other. In all analyses, *T. mjolniri* groups with *T. longitibia*, and these species are more than 4% different in COI sequence from each other. The position of that clade varies with dataset. Based upon overall similarity, phylogenetic position, shape of the pedipalps, and distribution, we were able to place this species within the Raptor Identification Group.

This species hypothesis is supported by low COI divergence within the species (0–2%) and high divergence between species (3–15%), and by the morphological characters outlined in the diagnosis.

####### Torrenticola mohawk

Taxon classificationAnimaliaTrombidiformesTorrenticolidae

Fisher & Dowling sp. n.

http://zoobank.org/387445A2-1757-4867-9771-DA7D6ADF62B6

######## Material examined.

HOLOTYPE (♀): from USA, Maine, Aroostook County, Ashland; beside Rt. 11 at bridge over Aroostook River, (46°38'38"N, 68°24'24"W), 4 July 1989, by IM Smith, IMS890067.

PARATYPES (4 ♀; 5 ♂): 1 ♂ (ALLOTYPE) from Aroostook County, Ashland; beside Rt. 11 at bridge over Aroostook River, (46°38'38"N, 68°24'24"W), 4 July 1989, by IM Smith, IMS890067 • 4 ♀ and 4 ♂ from Aroostook County, Ashland; beside Rt. 11 at bridge over Aroostook River, (46°38'38"N, 68°24'24"W), 4 July 1989, by IM Smith, IMS890067.

######## Type deposition.

Holotype (♀), allotype (♂), and some paratypes (2 ♀; 2 ♂) deposited in the CNC; other paratypes (2 ♀; 2 ♂) deposited in the ACUA.

######## Diagnosis.

*Torrenticola mohawk* are similar to other members of the Tricolor Complex (*T. bittikoferae*, *T. hoosieri*, *T. larvata*, *T. pearsoni*, *T. olliei*, *T. sierrensis*, *T. tricolor*, *T. trimaculata*, *T. unimaculata*, *T. cardia*, *T. kringi*, and *T. dimorpha*) in having a short, conical rostrum. *T. mohawk* are most similar to other members of the Tricolor Complex that have bold patterning (*T. larvata*, *T. tricolor*, *T. trimaculata*, *T. unimaculata*, *T. cardia*, and *T. kringi*). *T. mohawk* can be differentiated from *T. larvata*, *T. tricolor*, *T. cardia*, and *T. kringi* by having a stockier rostrum (length/width ♀ = 1.80–2.00 in *T. mohawk*, 2.14–3.13 in others; ♂ = 2.00–2.13 in *T. mohawk*, 2.27–2.93 in others). *T. mohawk* can be differentiated from *T. unimaculata* and *T. trimaculata* by dorsal pattern.

######## Description.

**Female (Figure 153)** (n = 5) (holotype measurements in parentheses when available) with characters of the genus with following specifications.

**Dorsum** — (680–725 (680) long; 540–560 (560) wide) ellipsoid with faint reddish-purple coloration medially. Anterio-medial platelets (130–140 (132.5) long; 60–77.5 (60) wide). Anterio-lateral platelets (185–197.5 (190) long; 75–82.5 (75) wide) free from dorsal plate. Dgl-4 closer to the edge of the dorsum than to the muscle scars (distance between Dgl-4 365–385 (365)). Dorsal plate proportions: dorsum length/width 1.21–1.32 (1.21); dorsal width/distance between Dgl-4 1.40–1.53 (1.53); anterio-medial platelet length/width 1.81–2.21 (2.21); anterio-lateral platelet length/width 2.31–2.53 (2.53); anterio-lateral/anterio-medial length 1.40–1.43 (1.43).

**Gnathosoma — Subcapitulum** (240–267.5 (260) long (ventral); 170–190 (185) long (dorsal); 115–125 (120) tall) colorless. Rostrum (82.5–95 (90) long; 42.5–52.5 (45) wide) short and conical. Chelicerae (225–255 (240) long) with curved fangs (50–60 (60) long). Subcapitular proportions: ventral length/height 2.08–2.33 (2.17); rostrum length/width 1.80–2.00 (2.00). **Pedipalps** with short, tuberculate ventral extensions on femora and genua. Palpomeres: trochanter (37.5–42.5 (41.25) long); femur (87.5–100 (95) long); genu (60–67.5 (65) long); tibia (77.5–87.5 (82.5) long; 25–27.5 (27.5) wide); tarsus (22.5–23.75 (23.75) long). Palpomere proportions: femur/genu 1.38–1.48 (1.46); tibia/femur 0.87–0.92 (0.87); tibia length/width 3.00–3.18 (3.00).

**Venter** — (800–850 (825) long; 600–630 (605) wide) colorless. Gnathosomal bay (125–145 (125) long; 70–75 (70) wide). Cxgl-4 subapical. **Medial suture** (30–45 (35) long). **Genital plates** (190–205 (195) long; 157.5–170 (160) wide). Additional measurements: Cx-1 (260–285 (260) long (total); 130–140 (130) long (medial)); Cx-3 (355–370 (360) wide); anterior venter (187.5–200 (190) long). Ventral proportions: gnathosomal bay length/width 1.70–2.07 (1.79); anterior venter/genital field length 0.93–0.99 (0.97); anterior venter length/genital field width 1.12–1.21 (1.19); anterior venter/medial suture 4.44–6.33 (5.43).

**Male (Figure 154)** (n = 5) (allotypic measurements in parentheses when available) with characters of the genus with following specifications.

**Dorsum** — (615–660 (655) long; 460–485 (470) wide) ellipsoid with faint reddish-purple coloration medially. Anterio-medial platelets (120–130 (122.5) long; 65–76.25 (67.5) wide). Anterio-lateral platelets (180–192.5 (187.5) long; 75–80 (75) wide) free from dorsal plate Dgl-4 closer to the edge of the dorsum than to the muscle scars (distance between Dgl-4 355–375 (360)). Dorsal plate proportions: dorsum length/width 1.33–1.39 (1.39); dorsal width/distance between Dgl-4 1.27–1.31 (1.31); anterio-medial platelet length/width 1.70–1.85 (1.81); anterio-lateral platelet length/width 2.25–2.50 (2.50); anterio-lateral/anterio-medial length 1.44–1.54 (1.53).

**Gnathosoma — Subcapitulum** (227.5–250 (250) long (ventral); 170–172.5 (172.5) long (dorsal); 105–107.5 (107.5) tall) colorless. Rostrum (80–90 (90) long; 40–45 (45) wide) short and conical. Chelicerae (210–225 (225) long) with curved fangs (50–55 (55) long). Subcapitular proportions: ventral length/height 2.17–2.33 (2.33); rostrum length/width 2.00–2.13 (2.00). **Pedipalps** with short, tuberculate ventral extensions on femora and genua. Palpomeres: trochanter (36.25–37.5 (37.5) long); femur (87.5–92.5 (90) long); genu (60–67.5 (65) long); tibia (75–80 (77.5) long; 26.25–28.75 (27.5) wide); tarsus (20–22.5 (22.5) long). Palpomere proportions: femur/genu 1.37–1.50 (1.38); tibia/femur 0.83–0.89 (0.86); tibia length/width 2.73–2.95 (2.82).

**Venter** — (735–780 (780) long; 510–530 (520) wide) colorless. Gnathosomal bay (107.5–127.5 (120) long; 65–70 (70) wide). Cxgl-4 subapical. **Medial suture** (120–130 (130) long). **Genital plates** (145–155 (152.5) long; 105–107.5 (105) wide). Additional measurements: Cx-1 (250–280 (280) long (total); 145–155 (155) long (medial)); Cx-3 (350–360 (360) wide); anterior venter (290–307.5 (307.5) long). Ventral proportions: gnathosomal bay length/width 1.54–1.82 (1.71); anterior venter/genital field length 1.93–2.03 (2.02); anterior venter length/genital field width 2.74–2.93 (2.93); anterior venter/medial suture 2.27–2.42 (2.37).

**Immatures** unknown.

######## Etymology.

Specific epithet (*mohawk*) named in honor of the Mohawk people (Kanien’kehá:ka), in reference to the dorsal coloration of this species, which resembles the hair-style by the same name (as a noun in apposition). The modern hairstyle does not exactly match traditional Mohawk styles, which resembled that of other tribes of the Iroquois Confederacy. Nevertheless, mohawks the hairstyle, which were originally associated with the Mohawk people in the historically problematic film *Drums Along the Mohawk* (1939), remains a symbol of the Mohawk people.

######## Distribution.

Maine (Figure 152).

**Figure 152.**
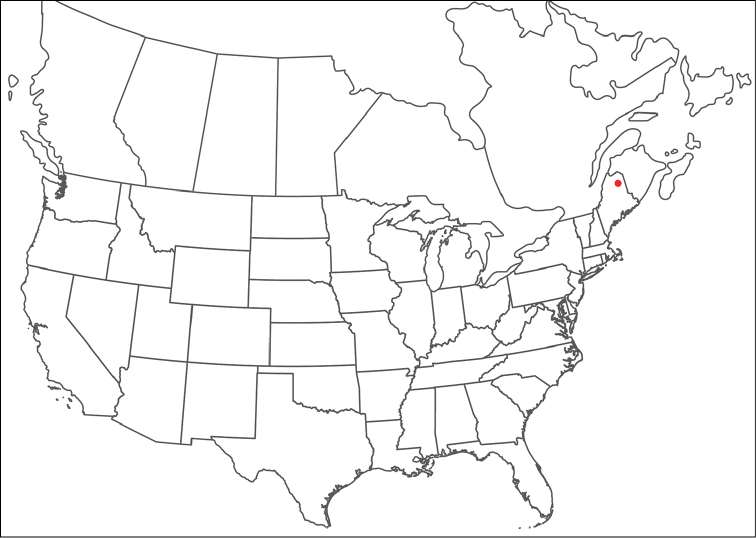
*Torrenticola mohawk* sp. n. distribution.

**Figure 153.**
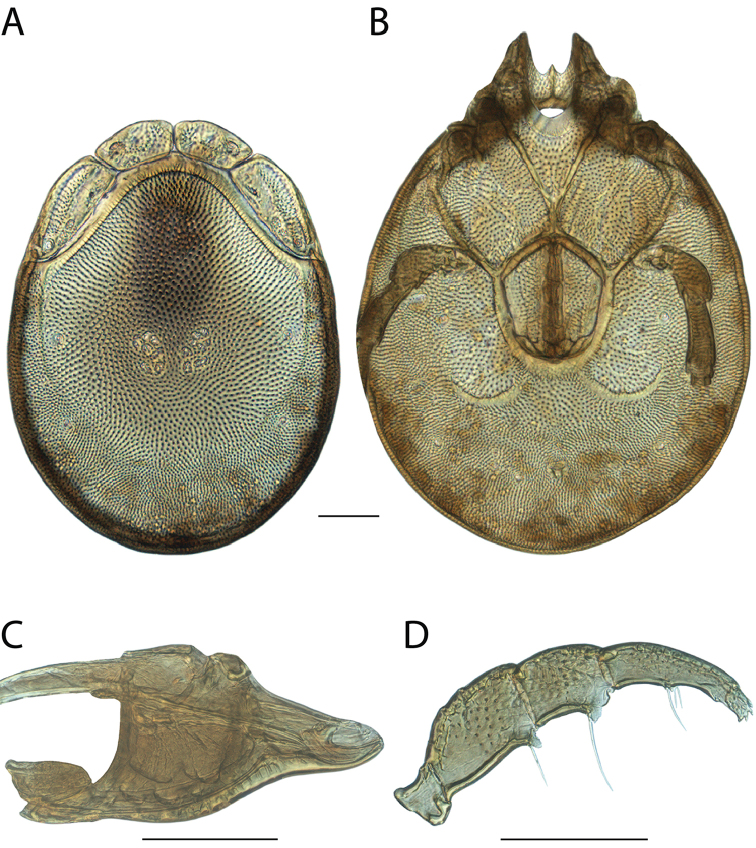
*Torrenticola mohawk* sp. n. female: **A** dorsal plates **B** venter (legs removed) **C** subcapitulum **D** pedipalp (setae not accurately depicted). Scale = 100 µm.

**Figure 154.**
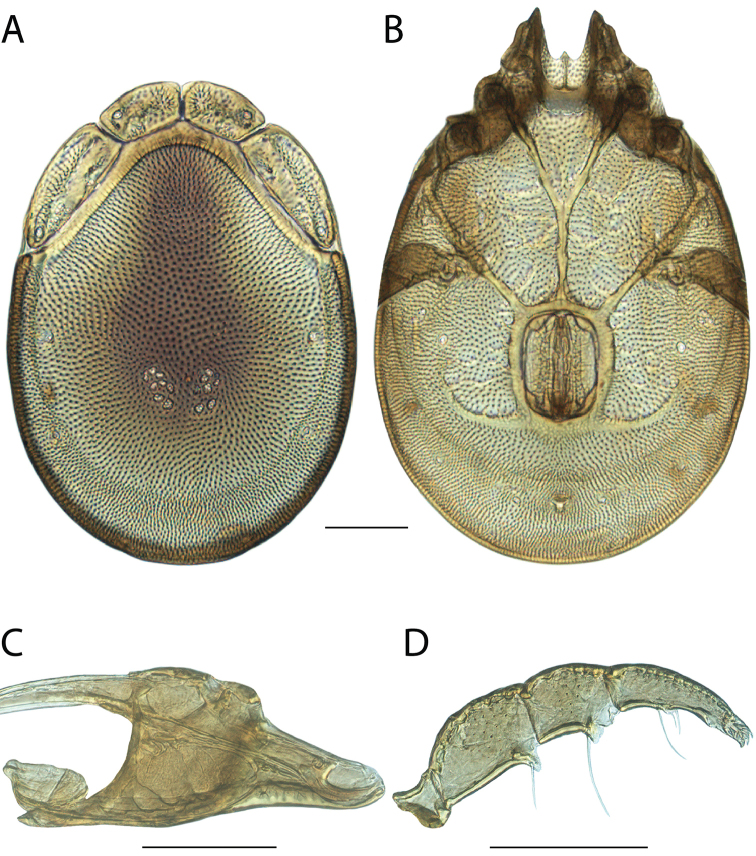
*Torrenticola mohawk* sp. n. male: **A** dorsal plates **B** venter (legs removed) **C** subcapitulum **D** pedipalp (setae not accurately depicted). Scale = 100 µm.

######## Remarks.

Unfortunately, we were unable to acquire fresh material of *Torrenticola mohawk* and therefore this species is not included in our phylogenetic analyses. However, we were able to examine morphology with material preserved in GAW. The overall similarity, distribution in the east, and short conical rostrum that is downturned in males, are consistent with placing this species in the Tricolor Complex and within the eastern clade of the Tricolor Identification Group.

####### Torrenticola mulleni

Taxon classificationAnimaliaTrombidiformesTorrenticolidae

Fisher & Dowling sp. n.

http://zoobank.org/56A3D559-6999-4116-9C5C-6DCC7473A979

######## Material examined.

HOLOTYPE (♀): from USA, Wyoming, Fremont County, Wind River Mountains, Sinks Canyon, Popo Agie River, south of Lander, 1 Aug 2012, by IM Smith, IMS120049, DNA 2928.

PARATYPES (6 ♀; 5 ♂): **Idaho, USA**: 1 ♀ from Blaine County, Little Wood River (43°29'51"N, 114°3'28"W), 27 Jul 2012, by JR Fisher, WA Nelson, & JC O’Neill, ROW 12-0727-001 • 1 ♂ from Custer County, Morgan Creek (44°39'20"N, 114°12'56"W), 31 Jul 2012, by JR Fisher, WA Nelson, & JC O’Neill, ROW 12-0731-004 • **Montana, USA**: 1 ♀ and 1 ♂ from Ravalli County, Bitterroot National Forest, East Fork Bitterroot River (45°51'40"N, 114°1'46"W), 3 Aug 2012, by JR Fisher, WA Nelson, & JC O’Neill, ROW 12-0803-005 • 1 ♀ and 1 ♂ from Missoula County, Lolo National Forest, off 12, Lolo Creek (46°46'7"N, 114°27'53"W), 7 Aug 2012, by JR Fisher, WA Nelson, & JC O’Neill, ROW 12-0807-003 • **Utah, USA**: 1 ♀ from Summit County, Wasatch-Cache National Forest, Slate Creek (40°37'45"N, 111°11'46"W), 23 Jul 2012, by JR Fisher, WA Nelson, & JC O’Neill, ROW 12-0723-006 • 1 ♂ from Wasatch County, Wasatch-Cache National Forest, Provo River (40°35'37"N, 111°5'43"W), 23 Jul 2012, by JR Fisher, WA Nelson, & JC O’Neill, ROW 12-0723-001 • **Wyoming, USA**: 1 ♂ (ALLOTYPE) from Fremont County, Wind River Mountains, Sinks Canyon, Popo Agie River, south of Lander, 1 Aug 2012, by IM Smith, IMS120049, DNA 2929 • 1 ♀ from Fremont County, Wind River Mountains, Sinks Canyon, Popo Agie River, south of Lander, 1 Aug 2012, by IM Smith, IMS120049 • 1 ♀ from Johnson County, Bighorn Mountains, Clear Creek, west of Buffalo Mosier Gulch Picnic Area, 28 Jul 2012, by IM Smith, IMS120041.

######## Type deposition.

Holotype (♀), allotype (♂), and some paratypes (4 ♀; 3 ♂) deposited in the CNC; other paratypes (2 ♀; 2 ♂) deposited in ACUA.

######## Diagnosis.

*Torrenticola mulleni* are similar to other members of the Rusetria “Western 2-Plates” group (*T. nortoni*, *T. walteri*, and *T. welbourni*) in having anterio-lateral platelets fused to the dorsal plate, having faint dorsal coloration, and being distributed in the west. Female *T. mulleni* can be differentiated from other female Western 2-Plates by having a longer medial suture (20–22.5 in *T. mulleni*, 10–12.5 in others). Male *T. mulleni* can be differentiated from other male Western 2-Plates by having a longer genital field (130–140 in *T. mulleni*, 112–125 in others). Additionally, *T. mulleni* can be differentiated from other Western 2-Plates by being distributed in the Rocky Mountains (Idaho, Montana, Utah and Wyoming) instead of California, Oregon and British Columbia.

######## Description.

**Female (Figure 156)** (n = 5) (holotype measurements in parentheses when available) with characters of the genus with following specifications.

**Dorsum** — (595–645 (640) long; 415–470 (440) wide) ovoid with faint purple coloration separated into anterior and posterior portions. Anterio-medial platelets (127.5–145 (145) long; 40–47.5 (45) wide). Anterio-lateral platelets (151.25–165 (160) long; 50–57.5 (57.5) wide) fused to dorsal plate. Dgl-4 much closer to the edge of the dorsum than to the muscle scars (distance between Dgl-4 300–330 (320)). Dorsal plate proportions: dorsum length/width 1.37–1.45 (1.45); dorsal width/distance between Dgl-4 1.34–1.47 (1.38); anterio-medial platelet length/width 2.95–3.24 (3.22); anterio-lateral platelet length/width 2.75–3.15 (2.78); anterio-lateral/anterio-medial length 1.10–1.29 (1.10).

**Gnathosoma — Subcapitulum** (300–327.5 (322.5) long (ventral); 225–245 (245) long (dorsal); 125–150 (145) tall) colorless. Rostrum (120–135 (125) long; 40–45 (45) wide). Chelicerae (300–335 (320) long) with curved fangs (40–65 (65) long). Subcapitular proportions: ventral length/height 2.14–2.40 (2.22); rostrum length/width 2.78–3.18 (2.78). **Pedipalps** with tuberculate ventral extensions on femora and genua. Palpomeres: trochanter (42.5–52.5 (42.5) long); femur (112.5–125 (117.5) long); genu (65–72.5 (67.5) long); tibia (82.5–90 (82.5) long; 26.25–28.75 (26.25) wide); tarsus (17.5–20 (17.5) long). Palpomere proportions: femur/genu 1.70–1.75 (1.74); tibia/femur 0.70–0.78 (0.70); tibia length/width 3.0–3.33 (3.14).

**Venter** — (720–790 (770) long; 460–500 (490) wide) colorless. Gnathosomal bay (160–175 (175) long; 75–90 (85) wide). Cxgl-4 subapical. **Medial suture** (20–22.5 (20) long). **Genital plates** (195–205 (205) long; 170–180 (175) wide). Additional measurements: Cx-1 (285–300 (300) long (total); 115–135 (130) long (medial)); Cx-3 (302.5–335 (320) wide); anterior venter (145–162.5 (155) long). Ventral proportions: gnathosomal bay length/width 1.89–2.16 (2.06); anterior venter/genital field length 0.74–0.80 (0.76); anterior venter length/genital field width 0.85–0.91 (0.89); anterior venter/medial suture 6.89–8.0 (7.75).

**Male (Figure 157)** (n = 4) (allotypic measurements in parentheses when available) with characters of the genus with following specifications.

**Dorsum** — (505–535 (530) long; 350–370 (360) wide) ovoid with faint purple coloration separated into anterior and posterior portions. Anterio-medial platelets (107.5–115 (110) long; 40–41.25 (40) wide). Anterio-lateral platelets (142.5–147.5 (142.5) long; 50–52.5 (52.5) wide) fused to dorsal plate. Dgl-4 much closer to the edge of the dorsum than to the muscle scars (distance between Dgl-4 270–280 (280)). Dorsal plate proportions: dorsum length/width 1.44–1.47 (1.47); dorsal width/distance between Dgl-4 1.29–1.37 (1.29); anterio-medial platelet length/width 2.61–2.88 (2.75); anterio-lateral platelet length/width 2.71–2.95 (2.71); anterio-lateral/anterio-medial length 1.28–1.33 (1.30).

**Gnathosoma — Subcapitulum** (262.5–277.5 (265) long (ventral); 195–210 (202.5) long (dorsal); 105–110 (105) tall) colorless. Rostrum (100–110 (110) long; 37.5–40 (40) wide). Chelicerae (265–270 (270) long) with curved fangs (50–55 (55) long). Subcapitular proportions: ventral length/height 2.39–2.58 (2.52); rostrum length/width 2.63–2.75 (2.75). **Pedipalps** with tuberculate ventral extensions on femora and genua. Palpomeres: trochanter (40–40 (40) long); femur (97.5–102.5 (102.5) long); genu (60–65 (62.5) long); tibia (75–82.5 (82.5) long; 26.25–27.5 (27.5) wide); tarsus (17.5–17.5 (17.5) long). Palpomere proportions: femur/genu 1.58–1.64 (1.64); tibia/femur 0.75–0.80 (0.80); tibia length/width 2.73–3.00 (3.00).

**Venter** — (620–650 (650) long; 390–425 (410) wide) colorless. Gnathosomal bay (130–135 (132.5) long; 70–75 (75) wide). Cxgl-4 subapical. **Medial suture** (100–105 (102.5) long). **Genital plates** (130–140 (140) long; 100–110 (110) wide). Additional measurements: Cx-1 (265–285 (285) long (total); 140–145 (140) long (medial)); Cx-3 (290–310 (310) wide); anterior venter (250–260 (250) long). Ventral proportions: gnathosomal bay length/width 1.77–1.93 (1.77); anterior venter/genital field length 1.79–2.00 (1.79); anterior venter length/genital field width 2.27–2.60 (2.27); anterior venter/medial suture 2.38–2.60 (2.44).

**Immatures** unknown.

######## Etymology.

Specific epithet (*mulleni*) named in honor of Gary Mullen of Auburn University, who boldly devoted the entire second half of his Arachnology course to mites, and thus, in 2001, first introduced JRF to these creatures, sparking a life-long fascination.

######## Distribution.

**Figure 155.**
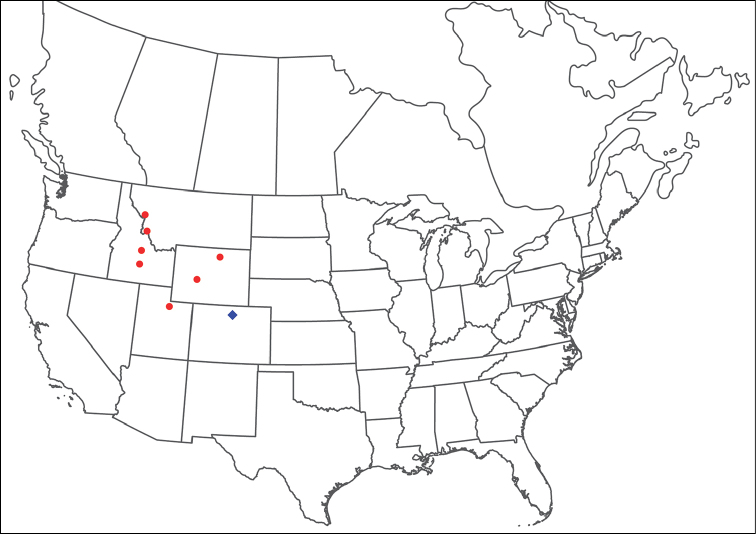
*Torrenticola mulleni* sp. n. distribution. Red dots represent material examined. Blue dot represents previously-published record (Young 1969), reported as *T. indistincta*, but likely representing *T. mulleni*.

Rocky Mountain region (Figure 155).

**Figure 156.**
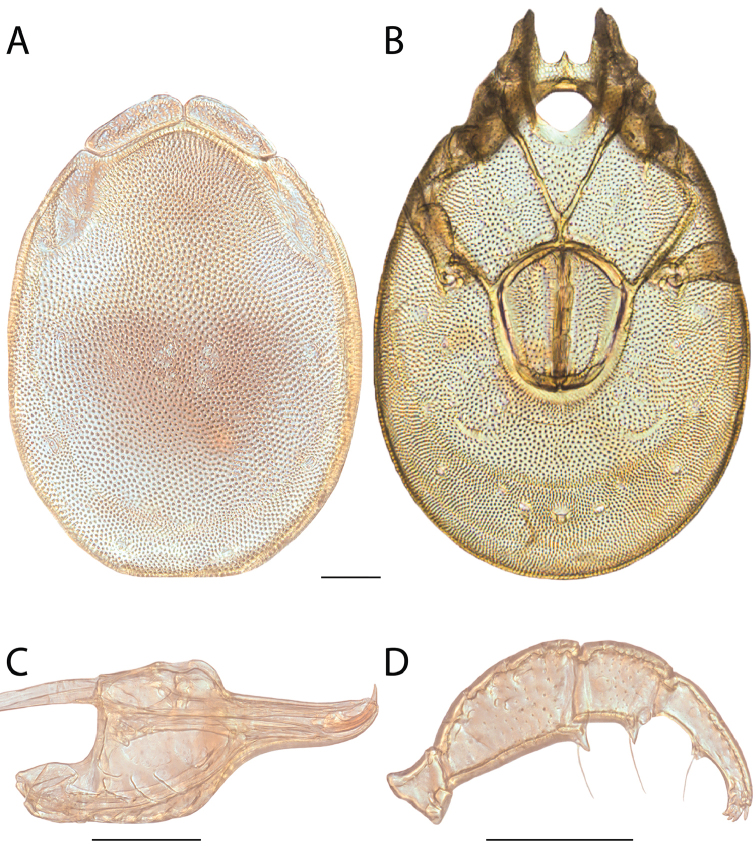
*Torrenticola mulleni* sp. n. male: **A** dorsal plates **B** venter (legs removed) **C** subcapitulum **D** pedipalp (setae not accurately depicted). Scale = 100 µm.

**Figure 157.**
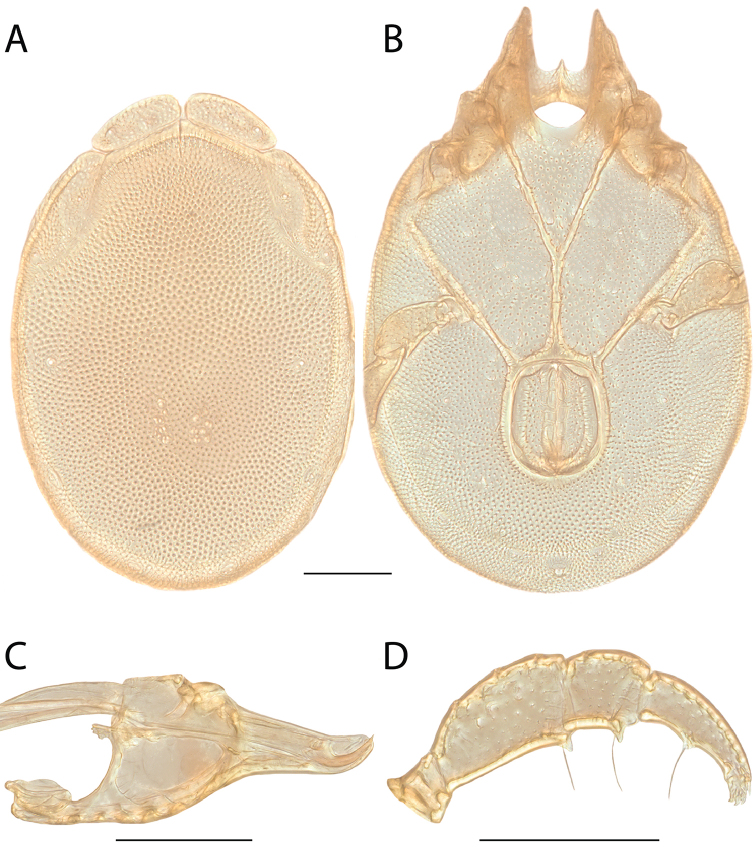
*Torrenticola mulleni* sp. n. female: **A** dorsal plates **B** venter (legs removed) **C** subcapitulum **D** pedipalp (setae not accurately depicted). Scale = 100 µm.

######## Remarks.

*Torrenticola mulleni* groups with other members of the Rusetria Complex with high support and specimens are less than 2% different in COI sequence from each other. In the all analyses, *T. mulleni* groups with the three other members of the Rusetria Complex that are found in western North America: *T. nortoni*, *T. walteri*, and *T. welbourni*. These species are 5–7% different in COI sequence from each other. *T. mulleni* is the only member of the Rusetria Complex that occurs throughout Rocky Mountains. Only *T. walteri* may overlap with this species in the northern Rockies of British Columbia; however, this overlap is speculative as *T. mulleni* was not found in any of our British Columbia samples. Young (1969) reported *T. indistincta* from Colorado, but we suspect this record represents *T. mulleni*.

Based upon overall similarity, the fusion of the posterio-lateral platelets to the dorsal shield, phylogenetic position, and distribution, we place this species within the Western 2-Plate Identification Group.

This species hypothesis is supported by non-overlapping distribution, low COI divergence within the species (0–2%) and high divergence between species (3–15%), and the morphological characters outlined in the diagnosis.

####### Torrenticola multiforma

Taxon classificationAnimaliaTrombidiformesTorrenticolidae

Habeeb, 1974

Torrenticola multiforma Habeeb, 1974: 4.

######## Material examined.

LECTOTYPE (1 ♀): from USA, California, Humboldt County, Prairie Creek Redwoods State Park, Prairie Creek, 12 Jul 1964, by Habeeb, HH640021.

PARALECTOTYPE (1 ♂): from USA, California, Humboldt County, Prairie Creek Redwoods State Park, Prairie Creek, 12 Jul 1964, by Habeeb, HH640021.

OTHER MATERIAL (95 ♀; 84 ♂): **Arizona, USA**: 1 ♀ from Cochise County, Chiricahua Mountains, Turkey Creek at Sycamore campground, east of Sunizona, 15 Jul 1987, by IM Smith, IMS870091 • **British Columbia, Canada**: 1 ♀ and 1 ♂ from Bella Coola Valley, Tweedsmuir Provincial Park, Atnarko River, at campground, 28 Jul 1983, by IM Smith & AB Smith, IMS830054A • 2 ♀ and 2 ♂ from Bella Coola Valley, Tweedsmuir Provincial Park, Atnarko Slough, beside Highway 20, west of Youngs Creek Picnic Area, 4 Aug 1983, by IM Smith, IMS830064 • 3 ♀ and 2 ♂ from Bella Coola Valley, Tweedsmuir Provincial Park, Belarko, Atnarko River, 24-26 Jul 1983, by IM Smith, IMS830049B & IMS830049C • 1 ♀ and 1 ♂ from Bella Coola Valley, Tweedsmuir Provincial Park, Hotnarko River, at end of Atnarko tote road, 27 Jul 1983, by IM Smith, IMS830052 • 5 ♀ and 6 ♂ from Bella Coola Valley, Tweedsmuir Provincial Park, Hotnarko River, at end of Atnarko tote road, 31 Jul 1983, by IM Smith, IMS830059A & IMS830059B • 1 ♀ and 1 ♂ from Bella Coola Valley, Tweedsmuir Provincial Park, Youngs Creek Picnic Area, Atnarko Slough, 24-27 Jul 1983, by IM Smith, IMS830048A • 1 ♀ and 1 ♂ from Bella Coola Valley, Tweedsmuir Provincial Park, Youngs Creek Picnic Area, tributary of Atnarko Slough, 2 Aug 1983, by IM Smith, IMS830062C • 2 ♀ and 1 ♂ from Fernie, Lizard Creek, beside Highway 3, 1.8 km west of Fernie Mountain Provincial Park, 16 Aug 2012, by IM Smith, IMS120073 • 1 ♂ from McClinchy River, beside Highway 20, west of Kleena Kleene, 5 Aug 1983, by IM Smith, IMS830068A • 1 ♀ and 1 ♂ from Tweedsmuir Provincial Park, Youngs Creek, beside Highway 20, between Heckman Pass & Bella Coola Valley, 5 Aug 1983, by IM Smith, IMS830065 • 5 ♀ from Vancouver Island, Caycuse, Nixon Creek, 8 Jul 1976, by IM Smith, IMS760197 & IMS760198 • 3 ♀ and 1 ♂ from Vancouver Island, beside Highway 4, 35.6 kilometers east of Pacific Rim Road, 9 Jul 1976, by IM Smith, IMS760206 • 1 ♀ and 1 ♂ from Vancouver Island, Lake Cowichan, South Shore Road, north of Mesachie Lake, Robertson River, 4 Jul 1976, by IM Smith, IMS760183A • 1 ♂ from Vancouver Island, Lake Cowichan, South Shore Road, north of Mesachie Lake, tributary of Robertson River, 4-10 Jul 1976, by IM Smith, IMS760182 • 1 ♀ and 2 ♂ from Vancouver Island, Lost Shoe Creek, beside Highway 4, 1.3 kilometers east of Pacific Rim Road, 9 Jul 1976, by IM Smith, IMS760202 • 1 ♀ and 1 ♂ from Vancouver Island, Malahat, Goldstream Provincial Park, Goldstream River, 26 Jun 1979, by IM Smith, IMS790028A • 1 ♀ and 2 ♂ from Vancouver Island, North Shore Road, 3.2 kilometers south of Youbou, 4 Jul 1976, by IM Smith, IMS760190 • 3 ♂ from Vancouver Island, Youbou, Shaw Creek, North Shore Road, 4.3 kilometers south of north end of Cowichan Lake, 8 Jul 1976, by IM Smith, IMS760196 • 1 ♀ from Vancouver Island, spring-fed pool beside South Shore Road, 2.3 kilometers south of Caycuse, 26 Jul 1979, by IM Smith, IMS790052 • 1 ♀ from Vancouver Island, spring run beside South Shore Road, 2.3 kilometers north of Lake Cowichan, 6 Jun 1979, by IM Smith, IMS790007 • **California, USA**: 1 ♀ and 2 ♂ from Del Norte County, Six Rivers National Forest, Middle Fork Smith River (41°51'20"N, 123°53'10"W), 15 Aug 2013, by JR Fisher, JRF 13-0815-002 • 1 ♀ and 2 ♂ from Mendocino County, Cottaneva Creek, beside Route 1, 21.8 kilometers southwest of Route 101, 5 Aug 1987, by IM Smith, IMS870129A • 4 ♀ and 4 ♂ from Mendocino County, small stream at beach access road, off Route 1, 2.6 kilometers south of Westport, 5 Aug 1987, by IM Smith, IMS870128A • 1 ♀ and 1 ♂ from Mono County, Humboldt-Toiyabe National Forest, Leavitt Creek (38°18'40"N, 119°34'49"W), 31 Aug 2013, by JR Fisher, JRF 13-0831-004 • 1 ♀ and 1 ♂ from Mono County, Humboldt-Toiyabe National Forest, West Walker River (38°21'59"N, 119°28'55"W), 31 Aug 2013, by JR Fisher, JRF 13-0831-003 • 1 ♀ from Monterey County, Los Padres National Forest, Salmon Creek (35°48'57"N, 121°21'29"W), 6 Sep 2013, by JR Fisher, JRF 13-0906-003 • 1 ♀ and 2 ♂ from Monterey County, Nacimiento River, beside Nacimiento-Ferguson Road at Nacimiento campground, 30 Jul 1987, by IM Smith, IMS870120A • 2 ♀ from Monterey County, Pfeiffer State Park, Big Sur River (36°14'42"N, 121°46'43"W), 4 Sep 2013, by JR Fisher, JRF 13-0904-004 • 2 ♀ and 2 ♂ from Monterey County, Salmon Creek, beside Route 1, south of Gorda, 28 Jul 1987, by IM Smith, IMS870114A • 1 ♀ and 1 ♂ from Nevada County, beside Route 89, north of Hobart Mills, 13 Jun 1976, by IM Smith, IMS760109 • 1 ♀ and 1 ♂ from Nevada County, Tahoe National Forest, Sagehen Creek (39°26'2"N, 120°12'17"W), 26 Aug 2013, by JR Fisher, JRF 13-0826-006 • 1 ♀ from Plumas County, beside Route 89, north of Greenville, 14 Jun 1976, by IM Smith, IMS760113 • 1 ♀ from Siskiyou County, Klamath National Forest, Dead Cow Creek (41°57'16"N, 122°52'23"W), 16 Aug 2013, by JR Fisher, JRF 13-0816-002 • 1 ♀ and 1 ♂ from Tulare County, Stony Creek at Stony Creek Picnic Area, east of Sequoia National Park, 1 Aug 1987, by IM Smith, IMS870124A • 1 ♀ from Ventura County, Ojai, North Fork of Ventura River, beside Route 33, just above Wheeler Gorge, 25-26 Jul 1987, by IM Smith, IMS870109A • **Idaho, USA**: 1 ♀ from Blaine County, Salmon River, beside Route 75 between Obsidian & Galena Summit, 3 Jul 1985, by IM Smith, IMS850067 • 1 ♂ from Blaine County, Sawtooth National Forest, Deer River (43°32'49"N, 114°26'31"W), 29 Jul 2012, by JR Fisher, WA Nelson, & JC O’Neill, ROW 12-0729-001 • 1 ♀ and 1 ♂ from Custer County, Basin Creek campground, Basin Creek, beside Route 75 between Sunbeam & Stanley, 2 Jul 1985, by IM Smith, IMS850066 • 1 ♀ from Custer County, Salmon River at picnic area, beside Route 93 north of Morgan Creek, 2 Jul 1985, by IM Smith, IMS850064 • 1 ♀ and 1 ♂ from Lemhi County, North Fork of Salmon River, beside Route 93, 15 kilometers north of North Fork, 1 Jul 1985, by IM Smith, IMS850062 • 1 ♀ from Lemhi County, Salmon National Forest, Indian Creek (45°24'12"N, 114°10'10"W), 2 Aug 2012, by JR Fisher, WA Nelson, & JC O’Neill, ROW 12-0802-001 • **Nevada, USA**: 2 ♀ and 2 ♂ from Elko County, Lamoille, Lamoille Creek near car park, at end of Lamoille Canyon Road, 12 Aug 1987, by IM Smith, IMS870143A • 2 ♀ and 3 ♂ from Humboldt County, Paradise Valley, Dutch John Creek beside road, 8.3 kilometers north of Hinkey Summit, 11 Aug 1987, by IM Smith, IMS870142A • **New Mexico, USA**: 2 ♀ and 2 ♂ from Catron County, Whitewater Creek, Glenwood Whitewater Picnic Area, 5 May 2012, by IM Smith, IMS120005 • 1 ♀ and 1 ♂ from Catron County, Glenwood Whitewater Creek at Whitewater Creek Picnic Area, 12 Jul 1987, by IM Smith, IMS870084 • 1 ♀ and 1 ♂ from Lincoln County, Eagle Creek, beside Route 532, just above Route 37, 8 Jul 1987, by IM Smith, IMS870077 • 1 ♀ and 1 ♂ from San Miguel County, Pecos, Pecos River, beside Route 63 at Dalton campground, 6 Jul 1987, by IM Smith, IMS870071 • 1 ♀ and 1 ♂ from San Miguel County, stream near Route 63 in Dalton Canyon, 6 Jul 1987, by IM Smith, IMS870073 • 1 ♀ from Sante Fe County, Sante Fe, Tesuque Creek, beside Route 22, south of Tesuque, 5 Jul 1987, by IM Smith, IMS870070 • **Montana, USA**: 1 ♀ from Lake County, stream beside Route 83, 39.5 kilometers north of Condon, 30 Jun 1985, by IM Smith, IMS850059A • 1 ♀ from Ravalli County, Beaverhead National Forest, East Fork Bitterroot River (45°51'40"N, 114°1'46"W), 3 Aug 2012, by JR Fisher, WA Nelson, & JC O’Neill, ROW 12-0803-004 • 1 ♂ from Ravalli County, Bitterroot National Forest, Piquette Creek (45°51'24"N, 114°11'37"W), 6 Aug 2012, by JR Fisher, WA Nelson, & JC O’Neill, ROW 12-0806-002 • 1 ♀ and 2 ♂ from Ravalli County, Medicine Springs, Spring Gulch campground, East Fork of Bitterroot River, beside Route 93, 1 Jul 1985, by IM Smith, IMS850060 • **Oregon, USA**: 1 ♀ from Clackamas County, Rhododendron Pioneer Tollgate campground, Zigzag River, 27 Jun 1976, by IM Smith, IMS760164 • 1 ♀ and 2 ♂ from Coos County, Siskiyou National Forest, Coal Creek, Road 33 between Powers & Agness, 2 Jul 1983, by IM Smith, IMS830015 • 2 ♀ and 2 ♂ from Coos County, Siskiyou National Forest, Road 33 between Powers & Agness, Daphne Grove campground, 2 Jul 1983, by IM Smith, IMS830017 • 1 ♀ and 1 ♂ from Curry County, Port Orford, Butler Bar campground, Elk River, 25 Jun 1976, by IM Smith, IMS760162 • 1 ♀ and 6 ♂ from Curry County, Port Orford, Humbug Mountain State Park Picnic Area, Brush Creek, beside Route 1, 3 Jul 1983, by IM Smith, IMS830020A & IMS830020B • 2 ♀ and 1 ♂ from Curry County, Port Orford, Humbug Mountain State Park Picnic Area, beside Route 1, Brush Creek, 1 Jul 1983, by IM Smith, IMS830012 • 1 ♀ from Curry County, Rogue River National Forest, Elk River (42°42'46"N, 124°18'41"W), 13 Aug 2013, by JR Fisher, JRF 13-0813-003 • 1 ♀ and 1 ♂ from Curry County, Siskiyou National Forest, road 33 between Powers and Agness, North Fork of Foster Creek, 2 Jul 1983, by IM Smith, IMS830019 • 2 ♀ and 1 ♂ from Curry County, Sixes, Sixes River, beside road at mouth of Edson Creek, 4 Jul 1983, by IM Smith, IMS830021A • 1 ♀ and 1 ♂ from Multnomah County, Columbia River Scenic Highway, Horsetail Falls, 27 Jun 1983, by IM Smith, IMS830005 • **Utah, USA**: 2 ♀ and 1 ♂ from Summit County, North Fork of Provo River, beside Route 150, west of Provo River Overlook, 14 Aug 1987, by IM Smith, IMS870148 • 1 ♂ from Utah County, Uinta National Forest, Hobble Creek Road, upstream on Right Fork (40°10'9"N, 111°28'36"W), 22 Jul 2012, by JR Fisher, WA Nelson, & JC O’Neill, ROW 12-0722-001 • 1 ♀ and 1 ♂ from Wasatch County, Provo River, beside Route 150 at Upper Provo River Bridge Picnic Area, 14 Aug 1987, by IM Smith, IMS870149 • 2 ♀ and 1 ♂ from Wasatch County, Wasatch-Cache National Forest, Holden Fork (40°47'19"N, 110°53'2"W), 23 Jul 2012, by JR Fisher, WA Nelson, & JC O’Neill, ROW 12-0723-003 • **Washington, USA**: 2 ♀ from Jefferson County, Rocky Brook (47°43'11"N, 122°56'32"W), 22 Jul 2013, by JC O’Neill & WA Nelson, JNOW 13-0722-002 • 1 ♀ and 1 ♂ from Lewis County, Gifford Pinchot National Forest, Snake Creek (46°38'52"N, 121°43'8"W), 23 Jul 2013, by JC O’Neill, & WA Nelson, JNOW 13-0723-006 • **Wyoming, USA**: 2 ♀ and 1 ♂ from Carbon County, Medicine Bow Mountains, Medicine Bow River, west of Arlington, 30 Jul 2012, by IM Smith, IMS120045 • 1 ♀ and 1 ♂ from Fremont County, Wind River Mountains, Sinks Canyon, Popo Agie River, 1 Aug 2012, by IM Smith, IMS120049.

######## Type deposition.

Types (1 ♀; 1 ♂) deposited in the CNC.

######## Diagnosis.

*Torrenticola multiforma* are similar to other members of the Ellipsoidalis Group (*T. ellipsoidalis*, *T. occidentalis*, and *T. leviathan*), in being among the largest *Torrenticola* in the west (dorsum length ♀ = 700–885; ♂ = 665–850), although *T. sierrensis* are also large (dorsum length ♀ = 700–880; ♂ = 590–735) but can easily be distinguished from the Ellipsoidalis Group by being circular instead of ellipsoid or rectangular (dorsum length/width = 1.17–1.28 in *T. sierrensis*, 1.30–1.67 in Ellipsoidalis Group). *T. multiforma* are best differentiated from other members of the Ellipsoidalis Group by having more elongate subcapitular rostra (length/width = 2.5–2.8 in *T. multiforma*, 1.84–2.27 in other Ellipsoidalis Group).

######## Re-description.

**Female (Figure 159)** (n = 6) with characters of the genus with following specifications.

**Dorsum** — (700–850 (730) long; 500–615 (550) wide) ellipsoid with highly variable coloration, colorless to orange to purple without distinct pattern. Anterio-medial platelets (132.5–150 (145) long; 65–77.5 (65) wide). Anterio-lateral platelets (195–230 (195) long; 77.5–92.5 (82.5) wide) free from dorsal plate. Dgl-4 much closer to the edge of the dorsum than to the muscle scars (distance between Dgl-4 380–465 (400)). Dorsal plate proportions: dorsum length/width 1.31–1.41 (1.33); dorsal width/distance between Dgl-4 1.26–1.38 (1.38); anterio-medial platelet length/width 1.94–2.27 (2.23); anterio-lateral platelet length/width 2.34–2.87 (2.36); anterio-lateral/anterio-medial length 1.34–1.57 (1.34).

**Gnathosoma — Subcapitulum** (340–385 long (ventral); 255–284 long (dorsal); 145–172.5 tall) colorless. Rostrum (132.5–152.5 long; 50–55 wide). Chelicerae (336–395 long) with curved fangs (62–79 long). Subcapitular proportions: ventral length/height 2.19–2.46; rostrum length/width 2.5–2.8. **Pedipalps** with tuberculate ventral extensions on femora and genua. Palpomeres: trochanter (37.5–45 long); femur (115–127.5 long); genu (75–90 long); tibia (87.5–95 long; 27.5–32.5 wide); tarsus (17.5–20 long). Palpomere proportions: femur/genu 1.42–1.53; tibia/femur 0.71–0.78; tibia length/width 2.77–3.18.

**Venter** — (840–1010 long; 588–679 wide) colorless. Gnathosomal bay (165–217.5 long; 82.5–100 wide). Cxgl-4 subapical. **Medial suture** (17.5–32.5 long). **Genital plates** (210–242.5 long; 197.5–222.5 wide). Additional measurements: Cx-1 (295–380 long (total); 123–170 long (medial)); Cx-3 (359–441 wide); anterior venter (175–212.5 long). Ventral proportions: gnathosomal bay length/width 1.89–2.44; anterior venter/genital field length 0.79–0.92; anterior venter length/genital field width 0.86–1.05; anterior venter/medial suture 5.83–11.14.

**Male (Figure 160)** (n = 6) with characters of the genus with following specifications.

**Dorsum** — (665–790 long; 470–580 wide) ellipsoid with highly variable coloration, colorless to orange to purple without distinct pattern. Anterio-medial platelets (125–165 long; 60–77.5 wide). Anterio-lateral platelets (185–222.5 long; 75–87.5 wide) free from dorsal plate. Dgl-4 much closer to the edge of the dorsum than to the muscle scars (distance between Dgl-4 365–450). Dorsal plate proportions: dorsum length/width 1.30–1.41; dorsal width/distance between Dgl-4 1.27–1.38; anterio-medial platelet length/width 1.97–2.33; anterio-lateral platelet length/width 2.46–2.67; anterio-lateral/anterio-medial length 1.35–1.48.

**Gnathosoma — Subcapitulum** (310–380 long (ventral); 236–284) long (dorsal); 137.5–170 tall) colorless. Rostrum (120–150 long; 45–60 wide). Chelicerae (309–382.5 long) with curved fangs (63–75 long). Subcapitular proportions: ventral length/height 2.16–2.34; rostrum length/width 2.50–2.79. **Pedipalps** with tuberculate ventral extensions on femora and genua. Palpomeres: trochanter (40–45 long); femur (102.5–122.5 long); genu (70–87.5 long); tibia (80–95 long; 26.25–32.5 wide); tarsus (17.5–20 long). Palpomere proportions: femur/genu 1.40–1.50; tibia/femur 0.73–0.79; tibia length/width 2.91–3.33.

**Venter** — (805–940 long; 479–653 wide) colorless. Gnathosomal bay (172.5–197.5 long; 75–100 wide). Cxgl-4 subapical. **Medial suture** (62.5–82.5 long). **Genital plates** (175–190 long; 140–155 wide). Additional measurements: Cx-1 (308–375 long (total); 127–180 long (medial)); Cx-3 (351–421 wide); anterior venter (225–270 long). Ventral proportions: gnathosomal bay length/width 1.92–2.30; anterior venter/genital field length 1.29–1.46; anterior venter length/genital field width 1.57–1.81; anterior venter/medial suture 3.27–4.00.

**Immatures** unknown.

######## Etymology.

Habeeb (1974) did not specify an etymology for the specific epithet (*multiforma*). However, he notes that the males “vary a lot in size.” Thus, we suspect the specific epithet refers to the variable body size males of this species (*multus*, L. many; *forma*, L. form).

######## Distribution.

**Figure 158.**
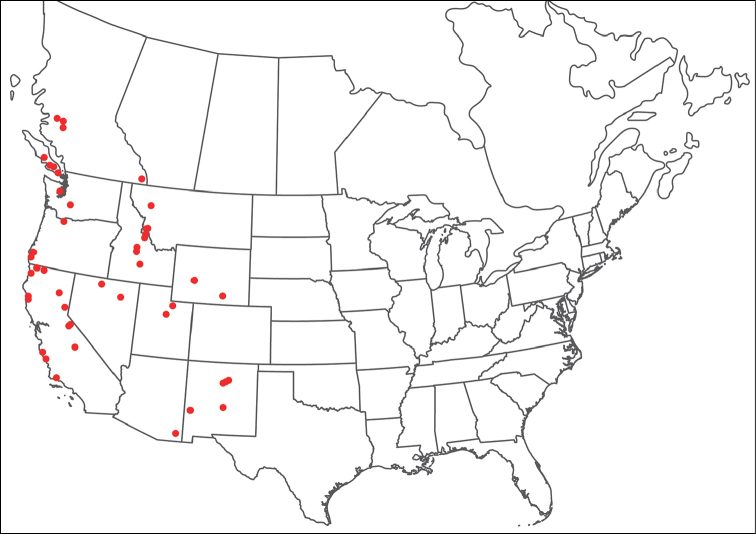
*Torrenticola multiforma* distribution.

Western (Figure 158). *T. multiforma* was previously known only from Prairie Creek, California (Habeeb 1974), but we expand its range to include most of western North America.

**Figure 159.**
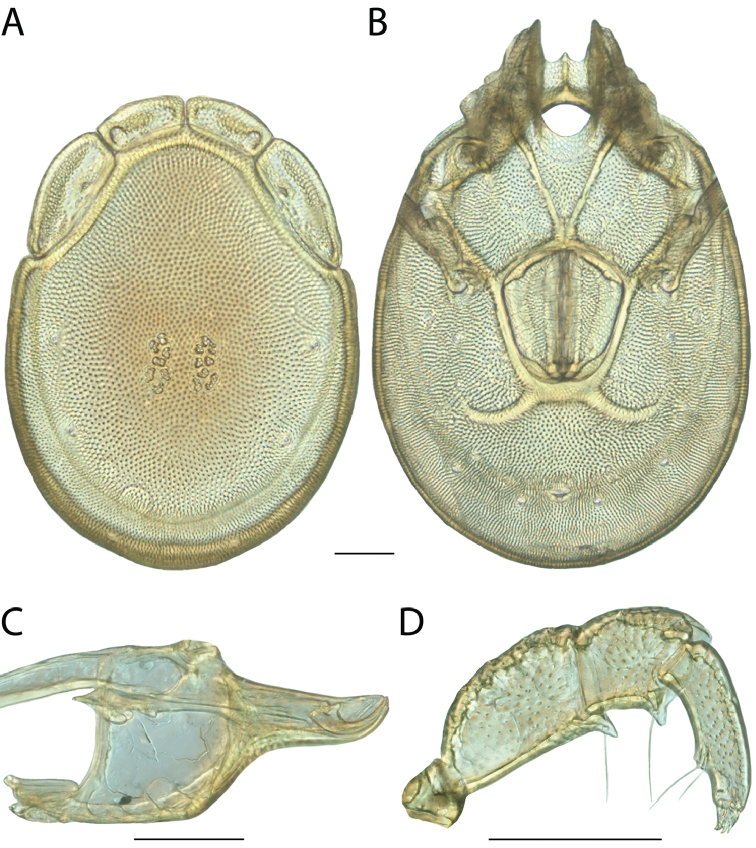
*Torrenticola multiforma* female: **A** dorsal plates **B** venter (legs removed) **C** subcapitulum **D** pedipalp (setae not accurately depicted). Scale = 100 µm.

**Figure 160.**
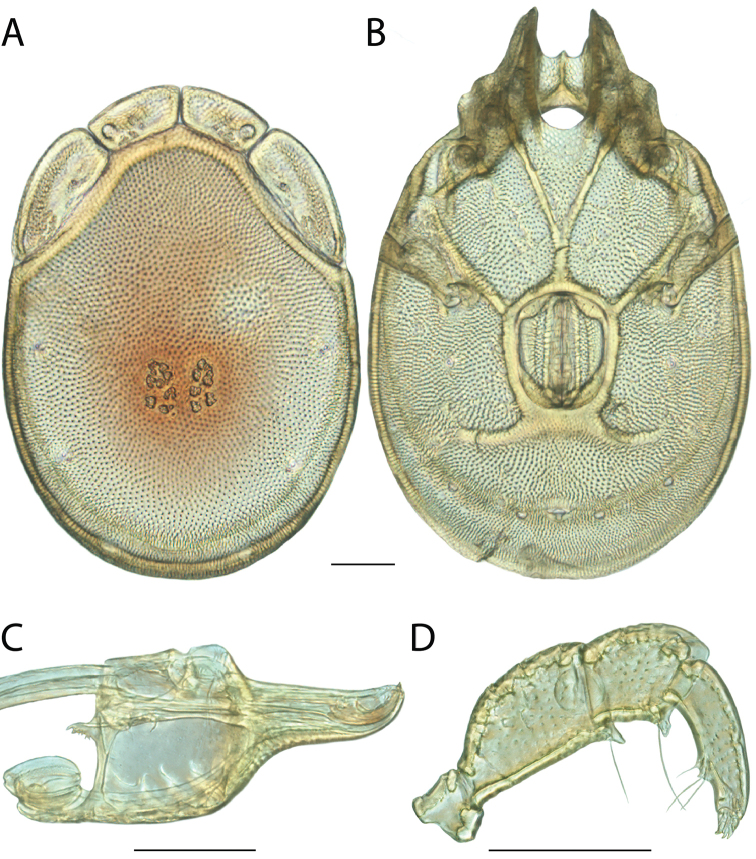
*Torrenticola multiforma* male: **A** dorsal plates **B** venter (legs removed) **C** subcapitulum **D** pedipalp (setae not accurately depicted). Scale = 100 µm.

######## Remarks.

*Torrenticola multiforma* groups other members of the Miniforma Complex with high support and specimens are 0–3% different in COI sequence. This is higher sequence variability than in many species hypotheses presented herein. However, given the topology in the COI tree (Figure 10) and morphological similarity, it seems apparent that the variability represents a continuum across a large distribution, rather than isolated species.

In all analyses, *T. multiforma* groups with *T. ellipsoidalis* and *T. regalis*, which are greater than 10% different from each other. Based upon overall similarity, body size, and distribution, we place this species within the Ellipsoidalis Identification Group.

This species hypothesis is supported by phylogenetic affinity, biogeography, high divergence between species (3–15%), and by the morphological characters outlined in the diagnosis.

####### Torrenticola neoanomala

Taxon classificationAnimaliaTrombidiformesTorrenticolidae

Habeeb, 1957

Torrenticola neoanomala Habeeb, 1957: 2.

######## Material examined.

HOLOTYPE (♂): from Canada, New Brunswick, Victoria County, Salmon River, 21 Jun 1953, by H Habeeb, HH530075.

PARATYPES (1 ♀; 0 ♂): **New Brunswick, Canada**: 1 ♀ from Victoria County, Salmon River, 21 Jun 1953, by H Habeeb, HH530075.

OTHER MATERIAL (27 ♀; 26 ♂; 1 nymph): **Arkansas, USA**: 1 nymph from Montgomery County, Caddo Gap, access track off Manfred Road, 0.3 km west of Route 8, 29 Jul 2011, by IM Smith, IMS110037 • 1 ♀ from Newton County, Buffalo National River, Mill Creek (36°3'42.12"N, 93°8'7.62"W), 30 May 2012, by TD Edwards, TDE 12-0530-004 • **Kentucky, USA**: 1 ♂ from McCreary County, White Oak Junction, Rock Creek, beside Forest Route 556, 2.3 kilometers south of Route 1363, 8 Jul 1990, by IM Smith, IMS900082A • **Maine, USA**: 1 ♀ and 1 ♂ from Aroostook County, Ashland, beside Route 11 at bridge, Aroostook River (46°38'N, 68°24'W), 4 Jul 1989, by IM Smith, IMS890067 • 2 ♀ and 1 ♂ from Piscataquis County, Baxter State Park, Trout Brook, beside road, 25 Jul 1980, by IM Smith, IMS800125A • 1 ♂ from Washington County, Old Stream, off Route 9, 5.5 km west of Route 192 at Wesley, 6 Jun 2012, by IM Smith, IMS120012 • **Missouri, USA**: 1 ♀ from Crawford County, Huzzah Creek, Red Bluff campground, east of Davisville, 23 Jul 2011, by IM Smith, IMS110029 • 1 ♂ from McDonald County, Tiff City, beside Route 43, Buffalo Creek (36°40'17"N, 94°36'17"W), 2 May 1996, by IM Smith, IMS960004 • **New Brunswick, Canada**: 2 ♀ and 6 ♂ from Charlotte County, Rollingham, Digdeguash River, beside Highway 770 at covered bridge, 30 Jun 1989, by IM Smith, IMS890053 • 3 ♀ from Charlotte County, Rollingham, Digdegaush River, beside Highway 770, 3 Oct 2011, by IM Smith, IMS110118 • 1 ♀ and 2 ♂ from York County, Magaguadavic River, beside Highway 3 just east of Thomaston Corners, 1 Jul 1989, by IM Smith, IMS890055A • 1 ♀ and 1 ♂ from York County, Nashwaak River, beside Highway 8, 1.7 kilometers north of Durham Bridge Road, 2 Jul 1989, by IM Smith, IMS890058 • **New York, USA**: 1 ♀ and 2 ♂ from Essex County, Minerva Boreas River, beside Route 28N, 13.8 kilometers northwest of Morse Memorial Parkway, 21 Jun 1990, by IM Smith, IMS900050A • 3 ♀ and 2 ♂ from Schuyler County, beside Town Line Road off Route 228, 0.6 kilometers south of Perry City, 21 July 1990, by IM Smith, IMS900112A • **Nova Scotia, Canada**: 1 ♀ and 1 ♂ from Antigonish County, Antigonish, West River, 1 Jul 1981, IMS810050 • 1 ♂ from Guysborough County, Sherbrooke, St. Mary’s River, 17 Sep 2011, by IM Smith, IMS110087 • 1 ♀ and 1 ♂ from Victoria County, Cape Brenton Island, Baddeck River, beside road to Baddeck Forks, 18 Jul 1981, by IM Smith, IMS810082 • **Ontario, Canada**: 3 ♀ and 3 ♂ from Grey County, Durham, Saugeen River, beside County Road 27 near Durham Conservation Area, 9 Jun 1989, by IM Smith, IMS890028A • **Pennsylvania, USA**: 1 ♀ from Fayette County, Ohiopyle State Park, Laurel Run (39°50'58"N, 79°30'51"W), 10 Aug 2014, by MJ Skvarla, MS 14-0810-005 • **Tennessee, USA**: 1 ♀ from Sevier County, Great Smokey Mountians National Park, Little River (35°40'56"N, 83°39'2"W), 8 Sep 2009, by IM Smith, IMS090103 • 2 ♀ from Monroe County, Tellico River, beside Forest Route 210, 1.8 kilometers east of bridge at Bald River Falls, 5 Jul 1990, by IM Smith, IMS900079 • **Texas, USA**: 1 ♂ from Shackelford County, Albany, beside Route 180, 4.6 kilometers east of Route 283 (32°44'29"N, 99°14'17"W), 3 May 1996, by IM Smith, IMS960007 • **Virginia, USA**: 1 ♀ from Alleghany County, Longdale Furnace, Simpson Creek, beside Forest Road 108, 1.7 kilometers west of Route 850, 14 Jul 1990, by IM Smith, IMS900094 • **West Virginia, USA**: 1 ♀ and 1 ♂ from Pendleton County, North Fork South Branch Potomac River, beside Route 28/55, 20.8 kilometers southwest of Route 42, 17 Jul 1990, by IM Smith, IMS900104.

######## Type deposition.

Holotype (♀) and allotype (♂) deposited in the CNC.

######## Diagnosis.

*Torrenticola neoanomala* are similar to species with similar dorsal patterning, such as the Rusetria “4-Plate” group (*T. dunni*, *T. glomerabilis*, *T. kittatinniana*, *T. pollani*, *T. rufoalba*, and *T. shubini*), Elongata Group (*T. elongata*, *T. gorti*, and *T. reduncarostra*) and *T. interiorensis*, *T. bondi*, *T. erectirostra*, *T. robisoni*, *T. irapalpa*, *T. racupalpa*, *T. skvarlai*, and *T. arktonyx*. They can be differentiated from Rusetria 4-Plates and *T. skvarlai* by having distinct hind coxal margins. *T. neoanomala* can be differentiated from *T. erectirostra* and *T. robisoni* by having a straight, anteriorly-directed rostrum (upturned in *T. erectirostra* and *T. robisoni*). *T. neoanomala* can be differentiated from *T. arktonyx* by having an unmodified dorsal plate (*T. arktonyx* has distinctive longitudinal dark markings on the anterior portion of the dorsal plate that fade posteriorly). *T. neoanomala* can be differentiated from *T. racupalpa* and *T. irapalpa* by having more elongate anterio-lateral platelets (length/width = 2.79–3.23 in *T. neoanomala*, 2.17–2.67 in others) and Dgl-4 closer to the dorsal edge (dorsal width/distance between Dgl-4 = 1.37–1.5 in *T. neoanomala*, 1.58–2.77 in others). *T. neoanomala* can be differentiated from Elongata Group by being slightly more ovoid (dorsum length/width ♀ = 1.35–1.43 in *T. neoanomala*, 1.45–2.08 in Elongata Group; ♂ = 1.43–1.50 in *T. neoanomala*, 1.51–1.7 in Elongata Group) and having a stockier rostrum (length/width = 2.59–2.90 in *T. neoanomala*, 3.24–4.00 in Elongata Group). *T. neoanomala* can be differentiated from *T. bondi* by having a longer medial suture (♀ = 22–40 in *T. neoanomala*, 10–15 in *T. bondi*; ♂ = 95–108 in *T. neoanomala*, 55–70 in *T. bondi*) and by anterior venter/genital field width (♀ = 1.39–1.45 in *T. neoanomala*, 1.15–1.25 in *T. bondi*; ♂ = 2.42–2.66 in *T. neoanomala*, 1.95–2.05 in *T. bondi*). Female *T. neoanomala* can be differentiated from female *T. interiorensis* by having more elongate anterio-lateral platelets (length/width = 2.86–3.09 in *T. neoanomala*, 2.62–2.67 in *T. interiorensis*). Male *T. neoanomala* can be differentiated from male *T. interiorensis* by having a longer anterior venter (267.5–290 in *T. neoanomala*, 220–240 in *T. interiorensis*) and a longer genital field (145–160 in *T. neoanomala*, 132–138 in *T. interiorensis*).

######## Re-description.

**Female (Figure 162)** (n = 6) (allotypic measurements in parentheses when available) with characters of the genus with following specifications.

**Dorsum** — (590–680 (650) long; 420–480 (480) wide) ovoid with bluish-purple to purple coloration separated into anterior and posterior portions with faint orange medially. Anterio-medial platelets (135–155 (140) long; 52.5–65 (60) wide). Anterio-lateral platelets (177.5–205 (205) long; 57.5–70 (67.5) wide) free from dorsal plate. Dgl-4 closer to the edge of the dorsum than to the muscle scars (distance between Dgl-4 280–345 (345)). Dorsal plate proportions: dorsum length/width 1.35–1.43 (1.35); dorsal width/distance between Dgl-4 1.39–1.50 (1.39); anterio-medial platelet length/width 2.23–2.58 (2.33); anterio-lateral platelet length/width 2.86–3.09 (3.04); anterio-lateral/anterio-medial length 1.29–1.46 (1.46).

**Gnathosoma — Subcapitulum** (320–350 long (ventral); 235–259 long (dorsal); 130–152.5 tall) colorless. Rostrum (120–142.5 long; 45–55 wide). Chelicerae (315–358 long) with curved fangs (56–61 long). Subcapitular proportions: ventral length/height 2.30–2.46; rostrum length/width 2.59–2.89. **Pedipalps** with tuberculate ventral extensions on femora and genua. Palpomeres: trochanter (40–47.5 (47.5) long); femur (110–128.75 (123.75) long); genu (67.5–75 (70) long); tibia (77.5–90 (90) long; 21.25–25 (25) wide); tarsus (16.25–20 (20) long). Palpomere proportions: femur/genu 1.63–1.81 (1.77); tibia/femur 0.67–0.74 (0.73); tibia length/width 3.58–3.68 (3.60).

**Venter** — (680–820 (770) long; 480–570 (570) wide) mostly colorless with faint bluish-purple or purple in areas surrounding coxae. Gnathosomal bay (147.5–177.5 (160) long; 87.5–107.5 (107.5) wide). Cxgl-4 subapical. **Medial suture** (22.5–40 (40) long). **Genital plates** (157.5–187.5 (187.5) long; 140–155 (145) wide). Additional measurements: Cx-1 (300–334 (325) long (total); 144–169 (162.5) long (medial)); Cx-3 (336–412.5 (412.5) wide); anterior venter (195–225 (207.5) long). Ventral proportions: gnathosomal bay length/width 1.49–1.78 (1.49); anterior venter/genital field length 1.11–1.27 (1.11); anterior venter length/genital field width 1.39–1.45 (1.43); anterior venter/medial suture 5.19–9.44 (5.19).

**Male (Figure 163)** (n = 5) (holotype measurements in parentheses when available) with characters of the genus with following specifications.

**Dorsum** — (545–635 (555) long; 370–430 (370) wide) ovoid with bluish-purple to purple coloration separated into anterior and posterior portions with faint orange medially. Anterio-medial platelets (120–130 (120) long; 48.75–62.5 (48.75) wide). Anterio-lateral platelets (167.5–197.5 (167.5) long; 55–62.5 (60) wide) free from dorsal plate. Dgl-4 closer to the edge of the dorsum than to the muscle scars (distance between Dgl-4 255–315 (265)). Dorsal plate proportions: dorsum length/width 1.43–1.50 (1.50); dorsal width/distance between Dgl-4 1.37–1.49 (1.40); anterio-medial platelet length/width 2.08–2.46 (2.46); anterio-lateral platelet length/width 2.79–3.23 (2.79); anterio-lateral/anterio-medial length 1.40–1.52 (1.40).

**Gnathosoma — Subcapitulum** (272.5–315 (272.5) long (ventral); 207.5–235 (207.5) long (dorsal); 97.5–122.5 (97.5) tall) colorless. Rostrum (112.5–127.5 (112.5) long; 38.75–45 (38.75) wide). Chelicerae (262–307 (267.5) long) with curved fangs (48–58 (55) long). Subcapitular proportions: ventral length/height 2.57–2.79 (2.79); rostrum length/width 2.67–2.90 (2.90). **Pedipalps** with tuberculate ventral extensions on femora and genua. Palpomeres: trochanter (40–42.5 (40) long); femur (100–115 (100) long); genu (60–70 (60) long); tibia (78.75–87.5 (78.75) long; 20–26.25 (20) wide); tarsus (15–20 (15) long). Palpomere proportions: femur/genu 1.60–1.69 (1.67); tibia/femur 0.74–0.80 (0.79); tibia length/width 3.33–3.94 (3.94).

**Venter** — (675–770 (675) long; 420–515 (470) wide) mostly colorless with faint bluish-purple or purple in areas surrounding coxae. Gnathosomal bay (127.5–152.5 (127.5) long; 68.75–90 (73.75) wide). Cxgl-4 subapical. **Medial suture** (95–107.5 (102.5) long). **Genital plates** (145–160 (150) long; 102.5–120 (102.5) wide). Additional measurements: Cx-1 (270–323 (270) long (total); 142–169 (152.5) long (medial)); Cx-3 (322–384 (340) wide); anterior venter (267.5–290 (272.5) long). Ventral proportions: gnathosomal bay length/width 1.69–2.15 (1.73); anterior venter/genital field length 1.79–1.90 (1.82); anterior venter length/genital field width 2.42–2.66 (2.66); anterior venter/medial suture 2.60–2.83 (2.66).

**Immatures** unknown.

######## Etymology.

Habeeb (1957) did not specify an etymology for the specific epithet (*neoanomala*). In fact, Habeeb (1955) initially identified the specimens as the Palaearctic *T. anomala*, but later (1957) writes, “The mite I reported as *Torrenticola anomala* (Koch) is now seen to be distinct from this European form.” So, this name surely refers to the similarity of this species to the *T. anomala* (Koch, 1837) (*néos*, G. new).

######## Distribution.

**Figure 161.**
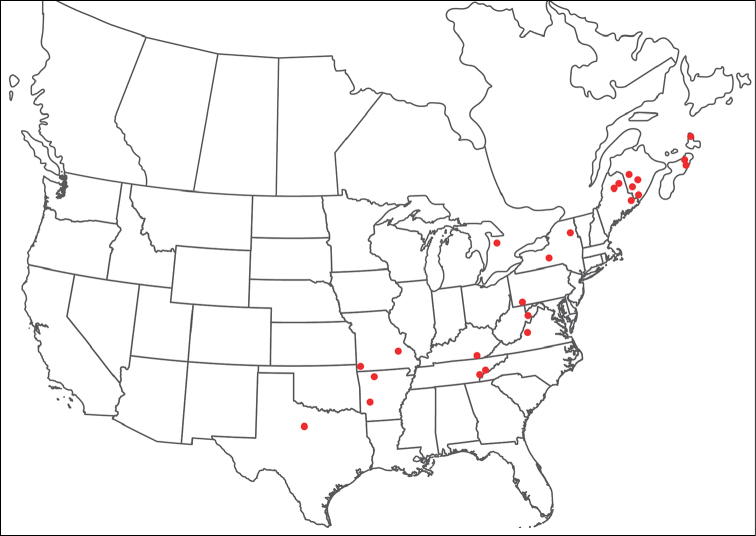
*Torrenticola neoanomala* distribution.

*T. neoanomala* was previously known only from Albert County, New Brunswick, but we extend its range throughout eastern North America (Figure 161). Records from Shackelford County, Texas, make *T. neoanomala* one of the few eastern *Torrenticola* that are found in the Great Plains.

**Figure 162.**
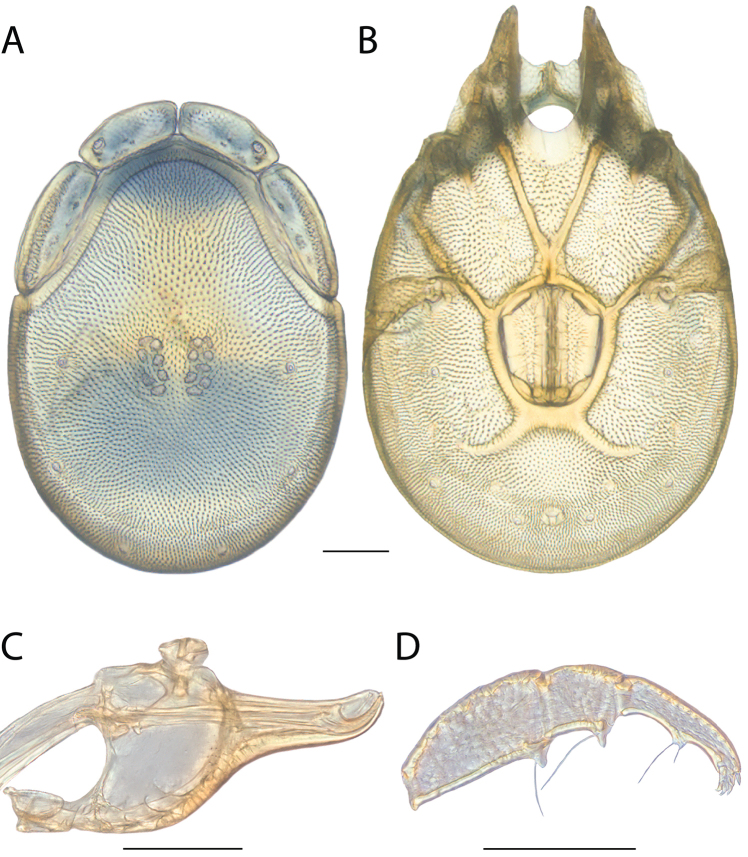
*Torrenticola neoanomala* female: **A** dorsal plates **B** venter (legs removed) **C** subcapitulum **D** pedipalp (setae not accurately depicted). Scale = 100 µm.

**Figure 163.**
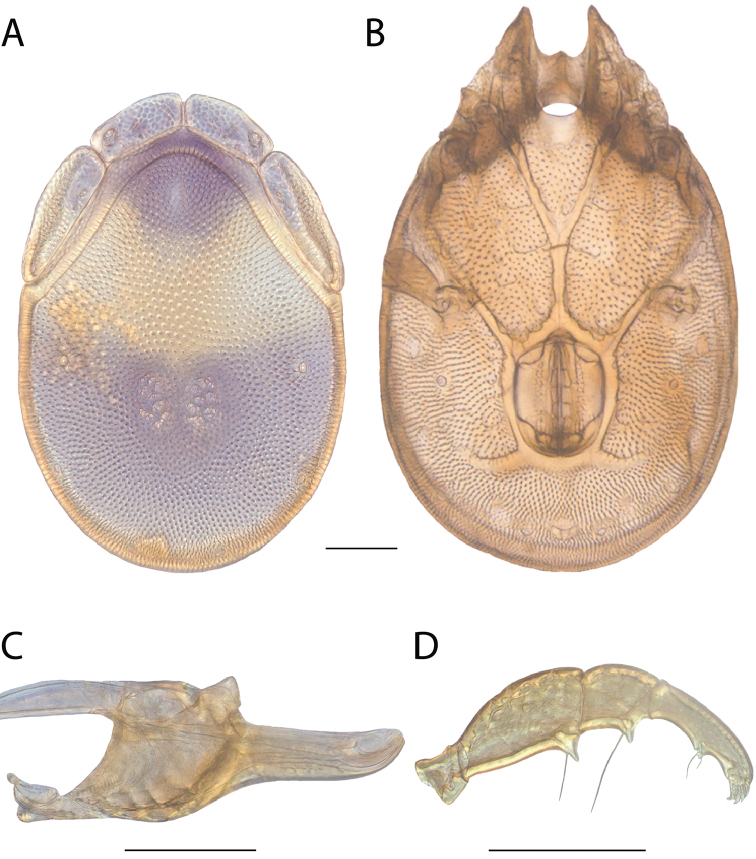
*Torrenticola neoanomala* male: **A** dorsal plates **B** venter (legs removed) **C** subcapitulum **D** pedipalp (setae not accurately depicted). Scale = 100 µm.

######## Remarks.

*Torrenticola neoanomala* groups with other members of the Raptor Complex with high support and specimens are less than 2.5% different in COI sequence from each other. In the combined analysis, *T. neoanomala* groups with the superficially similar *T. interiorensis*, and specimens from these species are greater than 9% different in COI sequence from each other.

Based upon this relationship and their similarity, we place these species in the Neoanomala Identification Group. The Neoanomala Group shares a phylogenetic affinity for members of the similar-looking Erectirostra Group.

This species hypothesis is supported by low COI divergence within the species (0–2%) and high divergence between species (3–15%), and by the morphological characters outlined in the diagnosis.

####### Torrenticola nigroalba

Taxon classificationAnimaliaTrombidiformesTorrenticolidae

Habeeb, 1955

Torrenticola nigroalba Habeeb, 1955: 2.

######## Material examined.

HOLOTYPE (♀): from Canada, New Brunswick, Victoria County, Salmon River, 21 Jun 1953, by H Habeeb, HH530075.

PARATYPES (0 ♀; 1 ♂): **New Brunswick, Canada**: 1 ♂ (ALLOTYPE) from Victoria County, Salmon River, 21 Jun 1953, by H Habeeb, HH530075.

OTHER MATERIAL (83 ♀; 80 ♂): **Alabama, USA**: 2 ♀ and 1 ♂ from Clay County, beside Forest Route 649, 0.8 kilometers northeast of road from Forest Route 600 to Campbell Springs, Talladega Creek, 2 Jul 1990, by IM Smith, IMS900075A • 1 ♂ from Cleburne County, beside Route 431, 3.3 kilometers southeast of Calhoun, Jackson Creek (33°36'N, 85°42'W), 2 Jul 1990, by IM Smith, IMS900074 • 1 ♀ and 1 ♂ from DeKalb County, Desoto State Park, beside Trail Y, West Fork of Little River (34°29'N, 85°32'W), by IM Smith, IMS920053A • **Georgia, USA**: 1 ♂ from Chattooga County, Cloudland, beside Route 48, East Fork Little River (34°31'25"N, 85°30'23"W), 29 Sep 2005, by IM Smith, IMS050110A • 1 ♂ from Floyd County, beside road from Everett Springs to Villanow, 1.8 kilometers south of The Pocket campground, Johns Creek, 4 Jul 1990, by IM Smith, IMS900076 • **Kentucky, USA**: 2 ♀ and 2 ♂ from Bell County, Middlesboro, Sugar Run (36°38'N, 83°39'W), 9 Jul 1990, by IM Smith, IMS900084 • **Maine, USA**: 1 ♀ and 1 ♂ from Aroostook County, Ashland, beside Route 11 at bridge, Aroostook River (46°38'N, 68°24'W), 4 Jul 1989, by IM Smith, IMS890067 • 1 ♂ from Aroostook County, beside Route 2A, 7 miles northeast of Macwahoc, Wytopitlock Stream, 5 Sep 1968, by DR Cook, DRC680063 • 1 ♀ and 1 ♂ from Aroostook County, Monticello, North Branch Meduxnekeag River, 28 Aug 1964, by DR Cook, DRC640042 • 1 ♀ and 1 ♂ from Franklin County, smalls Falls Picnic Area, beside Route 4, Sandy River (44°52'N, 70°31'W), 5 Jul 1989, by IM Smith, IMS890069 • 1 ♀ and 1 ♂ from Piscataquis County, Baxter State Park, Trout Brook, 25 Jul 1980, by IM Smith, IMS800125A • 4 ♀ from Washington County, Old Stream, off Route 9, 5.5 km west of Route 192, 6 Jun 2012, by IM Smith, IMS120012 • **Michigan, USA**: 1 ♀ and 1 ♂ from Cheboygan County, Pigeon River, 23 Jun 1952, by DR Cook, DRC520023 • **New Brunswick, Canada**: 1 ♀ and 2 ♂ from Charlotte County, Rollingham, Digdegaush River, beside Highway 770, 30 Jun 1989, by IM Smith, IMS890053 • 8 ♀ and 2 ♂ from Charlotte County, Rollingham, Digdegaush River, beside Highway 770, 3 Oct 2011, by IM Smith, IMS110118 • 2 ♀ and 2 ♂ from Northumberland County, beside Highway 108, Renous River, 18 Jul 1980, by IM Smith, IMS800111 • 1 ♀ and 1 ♂ from Restigouche County, Mount Carleton Provincial Park, Nictau River, 16 Jul 1980, by IM Smith, IMS800109 • 2 ♂ from Victoria County, near Limestone, small stream, 26 Aug 1964, by DR Cook, DRC640036 • 2 ♀ and 3 ♂ from York County, beside Highway 3, just east of Thomaston Corners, Magaguadavic River, 1 Jul 1989, by IM Smith, IMS890055A • 1 ♀ and 1 ♂ from York County, beside Highway 8, 1.7 kilometers north of Durham Bridge Road, Nashwaak River, 2 Jul 1989, by IM Smith, IMS890058 • 1 ♀ and 1 ♂ from York County, beside Highway 107, 27.3 kilometers west of Road J-19, North Branch of Southwest Miramichi River, 22 Jul 1981, by IM Smith, IMS810092B • 1 ♀ and 1 ♂ from York County, beside Road J-19, 6.3 kilometers north of Nashwaak Experimental Watershed headquarters, Napadogan Brook, 23 Jul 1981, by IM Smith, IMS810095A • 1 ♀ and 1 ♂ from York County, Nashwaak Experimental Watershed, Narrow Mountain Brook, 21 Jul 1980, by IM Smith, IMS800120A • **New Hampshire, USA**: 1 ♀ and 1 ♂ from Coos County, White Mountain National Forest, Zealand Picnic Area, Ammonoosuc River, 29 Jul 1980, by IM Smith, IMS800130 • **New Jersey, USA**: 1 ♀ and 1 ♂ from Sussex County, beside Flatbrook Road, 2.6 kilometers north of Route 206 at Tuttles Corner, Big Flat Brook, 23 Jun 1990, by IM Smith, IMS900053 • **New York, USA**: 3 ♂ from Essex County, beside Route 28N, 13.8 kilometers northwest of Morse Memorial Parkway at Minerva, Boreas River, 21 Jun 1990, by IM Smith, IMS900050A • **North Carolina, USA**: 3 ♀ and 1 ♂ from Haywood County, Great Smokey Mountains National Park, Big Creek (35°45'3.92"N, 83°6'31.67"W), 15 Sep 2009, by AJ Radwell, AJR090008A • 2 ♂ from Swain County, Oconaluftee River (35°32'34.76"N, 83°18'14.13"W), 6 Aug 2008, by AJ Radwell, AJR080019A • 1 ♂ from Yancey County, Lost Cove Picnic Area on Forest Route 472, 2.8 kilometers south of Route 80, South Toe River, 28 Jun 1990, by IM Smith, IMS900065A • **Nova Scotia, Canada**: 1 ♀ and 1 ♂ from Antigonish County, Antigonish, West River, 1 Jul 1981, by IM Smith, IMS810050 • 2 ♀ and 2 ♂ from Cape Brenton Highlands National Park, beside Cabot Trail, south of Neils Harbour, Halfway Brook, 4 Jul 1981, by IM Smith, IMS810058 • 5 ♀ and 3 ♂ from Cape Brenton Highlands National Park, picnic area above Mary Ann Falls, Mar Ann Brook, 5 Jul 1981, by IM Smith, IMS810060 • 1 ♀ and 1 ♂ from Cape Brenton Island, Victoria County, Ingonish, beside Cabot Trail, Dundas Brook, 11 Jul 1981, by IM Smith, IMS810074 • 2 ♀ from Inverness, Cheticamp River, 10 Sep 2011, by IM Smith, IMS110071 • 1 ♂ from Luneburg County, New Germany, LaHave River, beside Highway 10, 23 Sep 2011, by IM Smith, IMS110098 • 1 ♀ from Colchester County, Wentworth, picnic area beside Highway 104, tributary of Wallace River, 19 Jul 1981, by IM Smith, IMS810085 • **Ontario, Canada**: 1 ♂ from Algoma District, Lake Superior Provincial Park, Agawa River, 28 Aug, 1965, by DR Cook, DRC650001 • 2 ♀ and 2 ♂ from Grey County, Durham, beside County Road 27, near Durnham Conservation Area, Saugeen River, 9 Jun 1989, by IM Smith, IMS890028A • 2 ♀ and 1 ♂ from Hastings County, Madawaska, beside Highway 60, Opeongo River, 29 Aug 1981, by IM Smith, IMS810033A • 1 ♀ and 1 ♂ from Hastings County, Maple Leaf, Papineau Creek, east of Davis Road before Highway 62, 18 Aug 2011, by IM Smith, IMS110053 • 2 ♀ and 1 ♂ from Lanark County, beside Lanark Road #12, between Lanark and Fallbrook, Mississippi River, 6 October 1983, by IM Smith & CJ Hill, IMS830094A & IMS830094B • 1 ♀ and 1 ♂ from Muskoka District, Huntsville, East River Xing road to Dyer Memorial, 26 Aug 1981, by IM Smith, IMS810032A • 2 ♀ and 1 ♂ from Nipissing District, Algonquin Provincial Park, at Highway 60, near Lake of Two Rivers, Madawaska River, 15 May 1980, by IM Smith & CJ Hill, IMS800004B & IMS800004C • 1 ♀ and 1 ♂ from Peterborough County, crossing Highway 28 at picnic area just north of Woodview, Eels Creek, 13 Jun 1981, by IM Smith, IMS810018A • 2 ♀ and 2 ♂ from Thunder Bay District, Rossport, Rossport Provincial Campground, off Highway 17, McLeans Creek, 3 Aug 1988, by IM Smith, IMS880065 • **Pennsylvania, USA**: 1 ♀ and 1 ♂ from Fayette County, Dunbar Creek (39°56'16"N, 79°35'3.70"W), 10 Aug 2014, by MJ Skvarla, MS 14-0810-002 • 1 ♀ from Fayette County, off Meadow Run Road, Laurel Run (39°50'58"N, 79°30'51"W), 10 Aug 2014, by MJ Skvarla, MS 14-0810-005 • **Quebec, Canada**: 1 ♂ from Stanstead County, Tompkin Stream, Tomifobia River (45°0'31"N, 72°7'6"W), 20 Aug 1996, by IM Smith & M MacKenzie, IMS960056 • **Tennessee, USA**: 2 ♀ from Blount County, Great Smokey Mountains National Park, Cades Cove, Abrams River (35°35'31"N, 83°51'21"W), 17 Sep 2010, IMS100141 • 2 ♀ and 6 ♂ from Monroe County, Turkey Creek at confluence with Tellico River, 12 Sep 2009, by AJ Radwell, AJR090004 • 2 ♂ from Sevier County, Great Smokey Mountains National Park, middle prong Little Pigeon River (35°43'32"N, 83°24'2"W), 2 Sep 2009, by IM Smith, IMS090093 • **Vermont, USA**: 2 ♀ from Addison County, beside road from Lincoln to Ripton, Middlebury River (44°0'N, 73°1'W), 6 Jul 1989, by IM Smith, IMS890075 • 1 ♀ and 1 ♂ from Addison County, Lincoln, beside US Forest Service Road #54, Haven River (44°6'N, 72°59'W), 6 Jul 1989, by IM Smith, IMS890074 • **Virginia, USA**: 1 ♀ and 1 ♂ from Alleghany County, Covington, beside Route 18, 0.5 kilometers north of Route 657, Potts Creek, 13 Jul 1990, by IM Smith, IMS900091A • 1 ♀ from Amherst County, Upper Otter Creek Overlook beside Blue Ridge, Otter Creek (37°36'57"N, 79°19'27"W), 7 Sep 2007, by IM Smith, IMS070056A • 2 ♀ and 2 ♂ from Bath County, beside Route 287, 2.4 kilometers south of Bacova, Cowardin Run, 15 Jul 1990, by IM Smith, IMS900097 • 1 ♀ and 1 ♂ from Bath County, beside Forest Route 1843, 3.5 kilometers south of Route 220, Jackson River, 16 Jul 1990, IMS900100 • 1 ♀ from Giles County, Mechanicsburg, beside Dismal Creek Road, Standrock Brook (37°11'38"N, 80°53'26"W), 9 Sep 2005, by IM Smith, IMS050066 • 3 ♂ from Highland County, 4.8 kilometers northwest of McDowell, Crab Run, 23 Jul 1964, by DR Cook, DRC640013 • 1 ♀ from Montgomery County, Blacksburg, beside Route 321 at Caldwell, Craig Creek (37°20'N, 80°20'W), 12 Jul 1990, by IM Smith, IMS900089A • 2 ♀ and 2 ♂ from Page County, beside Route 730, 0.2 kilometers west of Route 675, Passage Creek, 25 Jun 1990, by IM Smith, IMS900059 • 2 ♀ and 2 ♂ from Washington County, Damascus, beside Route 58 near boundary of Mount Rogers National Recreation Area, Laurel River, 10 Jul 1990, by IM Smith, IMS900085A • **West Virginia, USA**: 1 ♀ and 1 ♂ from Randolph County, Laurel Fork Campground, off Forest Route 14, south of Wymer, Laurel Fork of Cheat River, 17 Jul 1990, by IM Smith, IMS900102.

######## Diagnosis.

*Torrenticola nigroalba* are similar to other members of the Nigroalba Group (*T. flangipalpa*, *T. solisorta*, *T. dentirostra*) in being small, slightly elongate, having purple dorsal coloration restricted posteriorly, and having distinct yet poorly-defined hind coxal margins. *T. nigroalba* can be differentiated from *T. flangipalpa* in having tuberculate pedipalp femoral extension (flange-like and anteriorly-directed in *T. flangipalpa*); a shorter anterior venter (200–223 in *T. nigroalba*, 235–265 in *T. flangipalpa*); and more elongate pedipalpal tibiae (length/ width ♀ = 5.38–5.83 in *T. nigroalba*, 4.79–5.00 in *T. flangipalpa*; ♂ = 5.08–5.33 in *T. nigroalba*, 4.40–4.86 in *T. flangipalpa*). *T. nigroalba* can be differentiated from *T. dentirostra* by having a smooth rostrum (*T. dentirostra* has a dentate bump midway on the dorsal edge of the rostrum) and by having more elongate pedipalpal tibiae (length/width ♀ = 5.3–5.9 in *T. nigroalba*, 4.5–5.0 in *T. dentirostra*; ♂ = 5.0–5.4 in *T. nigroalba*, 4.5–4.7 in *T. dentirostra*). *T. nigroalba* can be differentiated from *T. solisorta* by lacking orangish coloration immediately anterior to the purple dorsal coloration, although rarely specimens have been found with this coloration. Female *T. nigroalba* are also slightly larger (500–530 in *T. nigroalba*, 475–500 in *T. solisorta*); have a thinner gnathosomal bay (length/width = 1.25–1.55 in *T. nigroalba*, 1.3–1.5 in *T. solisorta*); and have a slightly thicker subcapitulum (3.00–3.14 in *T. nigroalba*, 3.14–3.30 in *T. solisorta*). Male *T. nigroalba* also have a longer medial suture with respect to the anterior venter (anterior venter/medial suture = 2.54–2.77 in *T. nigroalba*, 2.87–3.26 in *T. solisorta*) and a thinner dorsum (290–300 in *T. nigroalba*, 305–320 in *T. solisorta*). Other *Torrenticola* with purple dorsal coloration restricted posteriorly can be confused with *T. nigroalba*, such as *T. tahoei* and *T. oregonensis*. Both of these species are larger (dorsum length ♀ = 500–530 in *T. nigroalba*, 600–810 in others; ♂ = 440–455 in *T. nigroalba*, 560–820 in others) and distributed in the west (*T. nigroalba* is eastern).

######## Type deposition.

Holotype (♀) and allotype (♂) deposited in the CNC.

######## Re-description.

**Female (Figure 165)** (n = 6) (holotype measurements in parentheses when available) with characters of the genus with following specifications.

**Dorsum** — (500–530 (510) long; 330–365 (350) wide) ovoid with bluish-purple to purple coloration restricted posteriorly (rarely with faint orange at the anterior edge of the purple, especially medially). Anterio-medial platelets (105–112.5 (105) long; 40–47.5 (40) wide). Anterio-lateral platelets (145–160 (157.5) long; 46.25–52.5 (50) wide) free from dorsal plate. Dgl-4 closer to the edge of the dorsum than to the muscle scars (distance between Dgl-4 230–260 (245)). Dorsal plate proportions: dorsum length/width 1.42–1.53 (1.46); dorsal width/distance between Dgl-4 1.33–1.46 (1.43); anterio-medial platelet length/width 2.32–2.65 (2.63); anterio-lateral platelet length/width 2.90–3.26 (3.15); anterio-lateral/anterio-medial length 1.29–1.51 (1.50).

**Gnathosoma — Subcapitulum** (280–298 long (ventral); 197.5–220 long (dorsal); 92.5–97.5 tall) elongate and colorless. Rostrum (102.5–112.5 long; 35–40 wide). Chelicerae (273–287 long) with curved fangs (40–53 long). Subcapitular proportions: ventral length/height 3.03–3.14; rostrum length/width 2.81–3.10. **Pedipalps** elongate (especially tibiae) with tuberculate ventral extensions on femora and genua ending broadly and dentate. Palpomeres: trochanter (30–35 (32.5) long); femur (90–95 (95) long); genu (55–57.5 (57.5) long); tibia (85–92.5 (87.5) long; 15–16.25 (13.75) wide); tarsus (12.5–15 (12.5) long). Palpomere proportions: femur/genu 1.64–1.73 (1.65); tibia/femur 0.92–0.97 (0.92); tibia length/width 5.38–5.83 (5.38).

**Venter** — (620–660 (640) long; 340–430 (430) wide) with faint bluish purple or purple coloration. Gnathosomal bay (110–120 (110) long; 72.5–82.5 (82.5) wide). Cxgl-4 far from apex. **Medial suture** (50–55 (55) long). **Genital plates** (135–142.5 (137.5) long; 122.5–132.5 (123.75) wide). Additional measurements: Cx-1 (243–250 (250) long (total); 98–140 (140) long (medial)); Cx-3 (239–285 (285) wide); anterior venter (200–212.5 (203.75) long). Ventral proportions: gnathosomal bay length/width 1.33–1.55 (1.33); anterior venter/genital field length 1.42–1.53 (1.48); anterior venter length/genital field width 1.51–1.68 (1.65); anterior venter/medial suture 3.70–4.25 (3.70).

**Male (Figure 166)** (n = 6) (allotypic measurements in parentheses when available) with characters of the genus with following specifications.

**Dorsum** — (440–455 (455) long; 290–300 (295) wide) ovoid with bluish-purple to purple coloration restricted posteriorly. Anterio-medial platelets (92.5–100 (97.5) long; 35–42.5 (35) wide). Anterio-lateral platelets (137.5–150 (142.5) long; 40–47.5 (47.5) wide) free from dorsal plate. Dgl-4 closer to the edge of the dorsum than to the muscle scars (distance between Dgl-4 205–225 (225)). Dorsal plate proportions: dorsum length/width 1.50–1.55 (1.54); dorsal width/distance between Dgl-4 1.31–1.41 (1.31); anterio-medial platelet length/width 2.26–2.86 (2.79); anterio-lateral platelet length/width 3.00–3.63 (3.00); anterio-lateral/anterio-medial length 1.38–1.58 (1.46).

**Gnathosoma — Subcapitulum** (247.5–260 (250) long (ventral); 181–188.75 (188.75) long (dorsal); 77.5–82.5 (82.5) tall) elongate and colorless. Rostrum (92.5–97.5 (97.5) long; 32.5–35 (32.5) wide). Chelicerae (225–237.5 (237.5) long) with curved fangs (35–40 (40) long). Subcapitular proportions: ventral length/height 3.03–3.23 (3.03); rostrum length/width 2.79–3.00 (3.00). **Pedipalps** elongate (especially tibiae) with tuberculate ventral extensions on femora and genua ending broadly and dentate. Palpomeres: trochanter (25–30 (25) long); femur (77.5–82.5 (78.75) long); genu (50–55 (50) long); tibia (76.25–83.75 (80) long; 15–16.25 (15) wide); tarsus (15–17.5 (17.5) long). Palpomere proportions: femur/genu 1.50–1.65 (1.58); tibia/femur 0.95–1.05 (1.02); tibia length/width 5.08–5.33 (5.33).

**Venter** — (550–570 (550) long; 313–380 (370) wide) with faint bluish purple or purple coloration. Gnathosomal bay (100–107.5 (102.5) long; 60–70 (67.5) wide). Cxgl-4 far from apex. **Medial suture** (77.5–87.5 (80) long). **Genital plates** (105–118.75 (118.75) long; 85–92.5 (90) wide). Additional measurements: Cx-1 (216–237 (235) long (total); 97–135 (127.5) long (medial)); Cx-3 (230–263 (255) wide); anterior venter (212.5–222.5 (215) long). Ventral proportions: gnathosomal bay length/width 1.43–1.75 (1.52); anterior venter/genital field length 1.81–2.12 (1.81); anterior venter length/genital field width 2.32–2.51 (2.39); anterior venter/medial suture 2.54–2.77 (2.69).

**Immatures** unknown.

######## Etymology.

Habeeb (1955) did not specify an etymology for the specific epithet (*nigroalba*). However, surely this name refers to the dorsal coloration being half pigmented (purple) and half unpigmented (yellow), although Habeeb (1955) identified these colors as black and white (*niger*, L. black; *albus*, L. white).

######## Distribution.

**Figure 164.**
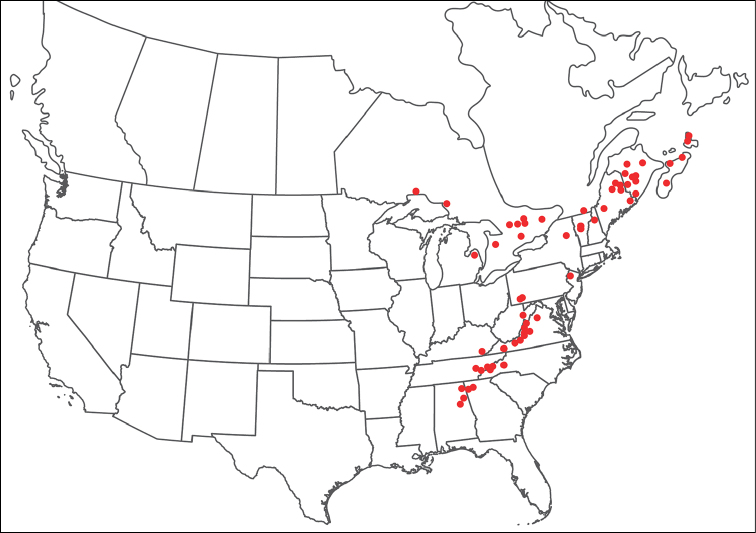
*Torrenticola nigroalba* distribution.

Northeastern and southward throughout the Appalachians (Figure 164). *T. nigroalba* was previously known only from Albert County, New Brunswick; we extend its distribution throughout the Appalachians and into Ottawa.

**Figure 165.**
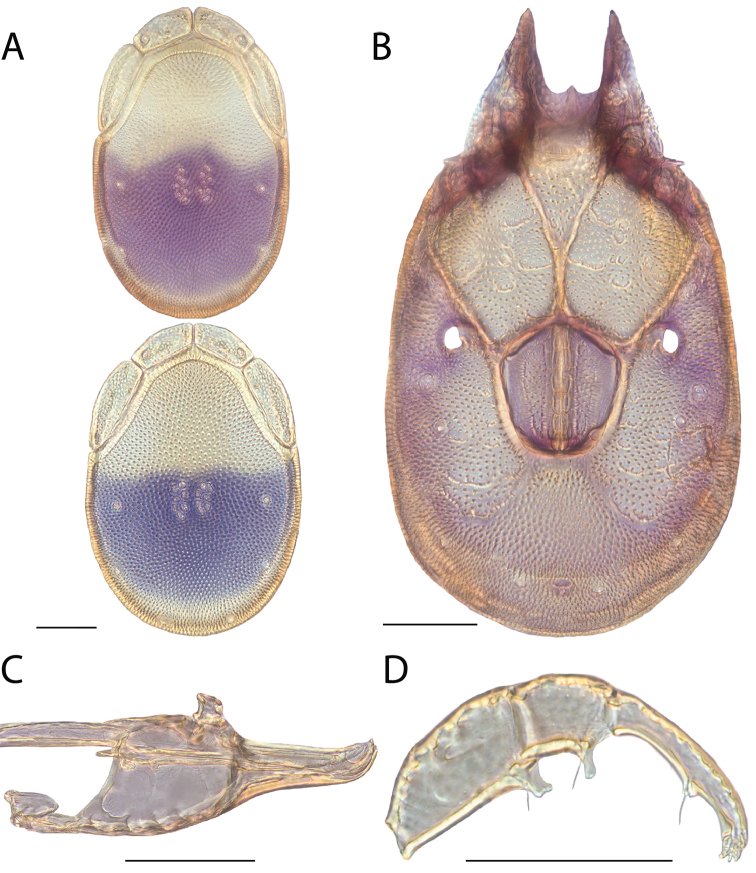
*Torrenticola nigroalba* female: **A** dorsal plates, note variation **B** venter (legs removed) **C** subcapitulum **D** pedipalp (setae not accurately depicted). Scale = 100 µm.

**Figure 166.**
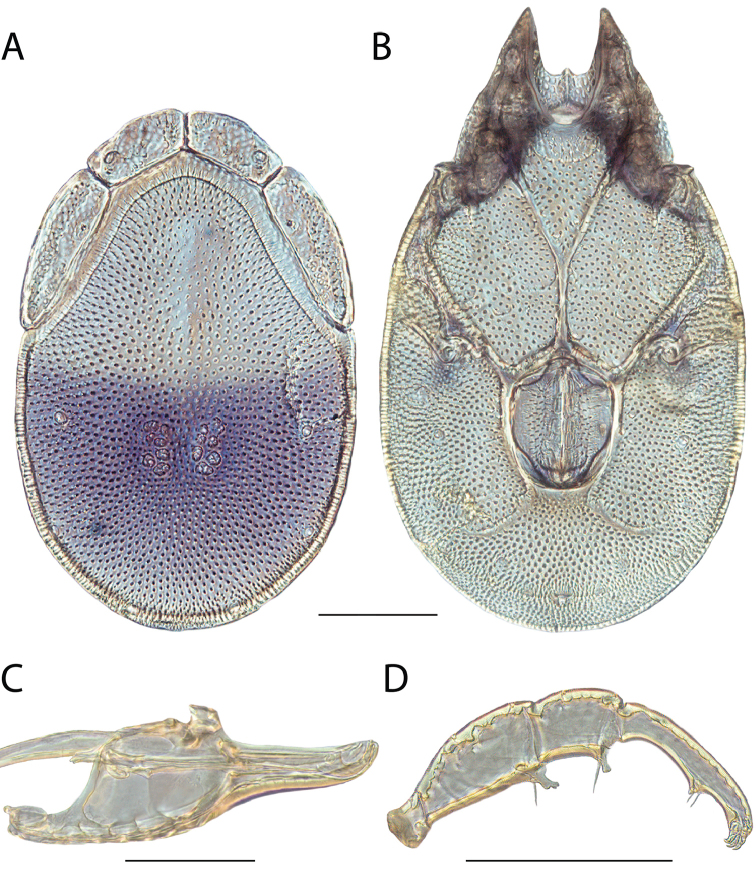
*Torrenticola nigroalba* male: **A** dorsal plates **B** venter (legs removed) **C** subcapitulum **D** pedipalp (setae not accurately depicted). Scale = 100 µm.

######## Remarks.

*Torrenticola nigroalba* groups with other members of the Raptor Complex with high support in all analyses and specimens are less than 1% different in COI sequence from each other. In all analyses, *T. nigroalba* groups with two other morphologically similar species: *T. flangipalpa* and *T. solisorta*. These three species are greater than 12% different from each other in COI sequence. That clade of three species corresponds to an identification group, the Nigroalba Group, the members of which are easily differentiated by their size, coloration, long medial suture in females, and overall appearance.

This species hypothesis is supported by low COI divergence within the species (0–2%) and high divergence between species (3–15%), and by the morphological characters outlined in the diagnosis.

####### Torrenticola nortoni

Taxon classificationAnimaliaTrombidiformesTorrenticolidae

Fisher & Dowling sp. n.

http://zoobank.org/6F164498-113D-4319-8ADB-D85FE40CBC46

######## Material examined.

HOLOTYPE (♀): from USA, California, Trinity County, Shasta-Trinity National Forest, Wilson Creek (40°25'17"N, 123°3'5"W), 20 Aug 2013, by JR Fisher, JRF 13-0820-003, DNA 3000.

PARATYPES (9 ♀; 5 ♂): **California, USA**: 1 ♂ (ALLOTYPE) from USA, California, Trinity County, Shasta-Trinity National Forest, Wilson Creek (40°25'17"N, 123°3'5"W), 20 Aug 2013, by JR Fisher, JRF 13-0820-003, DNA 2996 • 5 ♀ and 2 ♂ from Plumas County, Plumas National Forest, Silver Creek (39°56'60"N, 121°2'17"W), 24 Aug 2013, by JR Fisher, JRF 13-0824-005 • 3 ♀ and 1 ♂ from Trinity County, Shasta-Trinity National Forest, Wilson Creek (40°25'17"N, 123°3'5"W), 20 Aug 2013, by JR Fisher, JRF 13-0820-003 • 1 ♀ and 1 ♂ from Tulare County, Sequoia National Forest, Brush Creek (35°57'57"N, 118°28'43"W), 3 Sep 2013, by JR Fisher, JRF 13-0903-002.

######## Type deposition.

Holotype (♀), allotype (♂), and some paratypes (5 ♀; 2 ♂) deposited in the CNC; other paratypes (4 ♀; 2 ♂) deposited in ACUA.

######## Diagnosis.

*Torrenticola nortoni* are similar to other members of the Rusetria “Western 2-Plates” group (*T. mulleni*, *T. walteri*, and *T. welbourni*) in having anterio-lateral platelets fused to the dorsal plate, having faint dorsal coloration (some are colorless), and being distributed in the west. Female *T. nortoni* can be differentiated from *T. welbourni* (female only known) by having stockier pedipalp tibiae (3.13–3.33 in *A33*, 3.73 in *A30*) and shorter pedipalp femora (112.5–122.5 in *A33*, 137.5 in *A30*). Female *T. nortoni* can be differentiated from female *T. mulleni* by having a shorter medial suture (10–12.5 in *A33*; 20–22.5 in *A31*) and a shorter genital field (177.5–192.5 in *A33*, 195–205 in *A31*). Male *T. nortoni* can be differentiated from male *T. mulleni* by having shorter pedipalp femora (85–93 in *T. nortoni*, 97–103 in *T. mulleni*). Additionally, *T. nortoni* can be differentiated from *T. mulleni* by being distributed in California, instead of in the Rocky Mountains (Idaho, Montana, Utah and Wyoming). Female *T. nortoni* can be differentiated from female *T. walteri* by having slightly longer pedipalp femora with respect to genua (1.69–1.82 in *T. nortoni*, 1.52–1.64 in *T. walteri*) and slightly more elongate anterio-medial platelets (2.74–3.06 in *T. nortoni*, 2.58–2.72 in *T. walteri*). Male *T. nortoni* can be differentiated from male *T. walteri* by having longer pedipalp femora (85–92.5 in *T. nortoni*, 95–100 in *T. walteri*) and slightly more elongate pedipalp tibiae (2.73–3.0 in *T. nortoni*, 3.05–3.10 in *T. walteri*).

######## Description.

**Female (Figure 168)** (n = 5) (holotype measurements in parentheses when available) with characters of the genus with following specifications.

**Dorsum** — (570–645 (580) long; 420–480 (435) wide) ovoid with faint orange coloration or colorless. Anterio-medial platelets (122.5–131.25 (125) long; 40–47.5 (42.5) wide). Anterio-lateral platelets (155–180 (162.5) long; 50–57.5 (57.5) wide) fused to dorsal plate. Dgl-4 closer to the edge of the dorsum than to the muscle scars (distance between Dgl-4 295–320 (310)). Dorsal plate proportions: dorsum length/width 1.31–1.36 (1.33); dorsal width/distance between Dgl-4 1.40–1.50 (1.40); anterio-medial platelet length/width 2.74–3.06 (2.94); anterio-lateral platelet length/width 2.83–3.20 (2.83); anterio-lateral/anterio-medial length 1.22–1.38 (1.30).

**Gnathosoma — Subcapitulum** (310–330 (325) long (ventral); 232.5–243.25 (240) long (dorsal); 132.5–140 (132.5) tall) colorless. Rostrum (125–130 (130) long; 43.75–50 (45) wide). Chelicerae (315–325 (325) long) with curved fangs (60–66 (60) long). Subcapitular proportions: ventral length/height 2.34–2.45 (2.45); rostrum length/width 2.60–2.89 (2.89). **Pedipalps** with tuberculate ventral extensions on femora and genua. Palpomeres: trochanter (45–50 (45) long); femur (112.5–122.5 (120) long); genu (62.5–72.5 (70) long); tibia (80–90 87.5) long; 25–28.75 (26.25) wide); tarsus (17.5–20 (17.5) long). Palpomere proportions: femur/genu 1.69–1.82 (1.71); tibia/femur 0.71–0.73 (0.73); tibia length/width 3.13–3.33 (3.33).

**Venter** — (710–760 (730) long; 470–560 (490) wide) colorless. Gnathosomal bay (152.5–167.5 (167.5) long; 81.25–95 (85) wide). Cxgl-4 subapical. **Medial suture** (10–12.5 (12.5) long). **Genital plates** (177.5–192.5 (182.5) long; 160–182.5 (160) wide). Additional measurements: Cx-1 (270–281 (280) long (total); 110–124 (110) long (medial)); Cx-3 (300–365 (320) wide); anterior venter (157.5–177.5 (157.5) long). Ventral proportions: gnathosomal bay length/width 1.61–1.97 (1.97); anterior venter/genital field length 0.86–0.92 (0.86); anterior venter length/genital field width 0.97–0.98 (0.98); anterior venter/medial suture 12.6–17.75 (12.6).

**Male (Figure 169)** (n = 5) (allotypic measurements in parentheses when available) with characters of the genus with following specifications.

**Dorsum** — (450–500 (455) long; 310–380 (320) wide) ovoid with faint orange coloration or colorless. Anterio-medial platelets (97.5–105 (102.5) long; 35–37.5 (37.5) wide). Anterio-lateral platelets (127.5–145 (137.5) long; 45–53.75 (45) wide) fused to dorsal plate. Dgl-4 closer to the edge of the dorsum than to the muscle scars (distance between Dgl-4 240–285 (245)). Dorsal plate proportions: dorsum length/width 1.32–1.45 (1.42); dorsal width/distance between Dgl-4 1.29–1.37 (1.31); anterio-medial platelet length/width 2.67–2.80 (2.73); anterio-lateral platelet length/width 2.60–3.06 (3.06); anterio-lateral/anterio-medial length 1.28–1.44 (1.34).

**Gnathosoma — Subcapitulum** (250–267.5 (252.5) long (ventral); 180–195 (192.5) long (dorsal); 95–107.5 (97.5) tall) colorless. Rostrum (97.5–105 (105) long; 35–37.5 (37.5) wide). Chelicerae (246–260 (250) long) with curved fangs (45–52 (50) long). Subcapitular proportions: ventral length/height 2.49–2.63 (2.59); rostrum length/width 2.67–2.80 (2.80). **Pedipalps** with tuberculate ventral extensions on femora and genua. Palpomeres: trochanter (35–42.5 (37.5) long); femur (85–92.5 (92.5) long); genu (52.5–60 (56.25) long); tibia (66.25–75 (67.5) long; 22.5–27.5 (22.5) wide); tarsus (15–17.5 (17.5) long). Palpomere proportions: femur/genu 1.54–1.64 (1.64); tibia/femur 0.73–0.81 (0.73); tibia length/width 2.73–3.00 (3.00).

**Venter** — (560–620 (590) long; 350–431 (370) wide) colorless. Gnathosomal bay (106–128 (122.5) long; 65–67.5 (65) wide). Cxgl-4 subapical. **Medial suture** (82.5–110 (102.5) long). **Genital plates** (112.5–125 (115) long; 90–102.5 (90) wide). Additional measurements: Cx-1 (211–230 (230) long (total); 100–110 (110) long (medial)); Cx-3 (260–292 (270) wide); anterior venter (232.5–250 (240) long). Ventral proportions: gnathosomal bay length/width 1.60–1.89 (1.88); anterior venter/genital field length 1.96–2.13 (2.09); anterior venter length/genital field width 2.39–2.67 (2.67); anterior venter/medial suture 2.18–2.82 (2.34).

**Immatures** unknown.

######## Etymology.

Specific epithet (*nortoni*) named in honor of acarologist Roy Norton who taught JRF oribatids during the Soil Mite Course at The Ohio State University Acarology Summer Program; specifically for his talent in weaving biological stories into his lessons.

######## Distribution.

**Figure 167.**
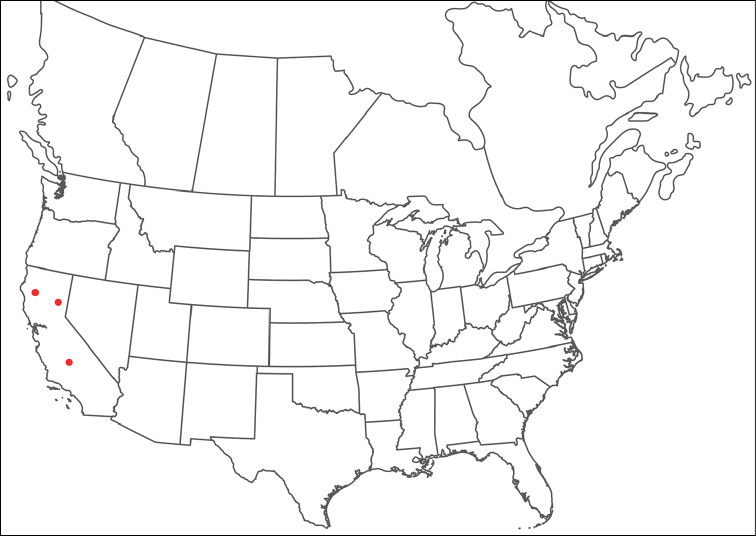
*Torrenticola nortoni* sp. n. distribution.

California (Figure 167).

**Figure 168.**
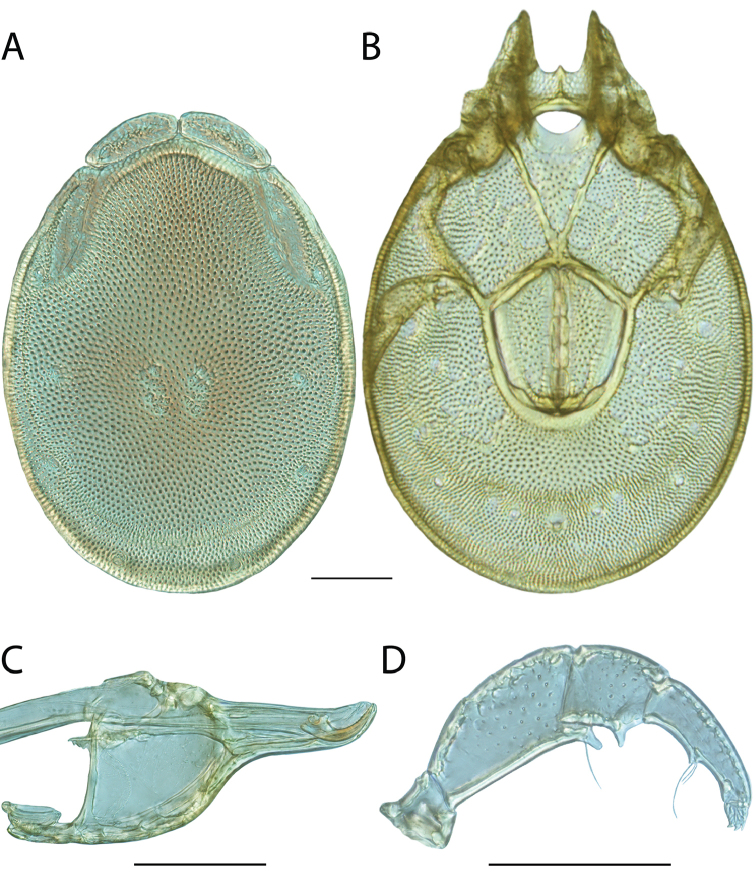
*Torrenticola nortoni* sp. n. female: **A** dorsal plates **B** venter (legs removed) **C** subcapitulum **D** pedipalp (setae not accurately depicted). Scale = 100 µm.

**Figure 169.**
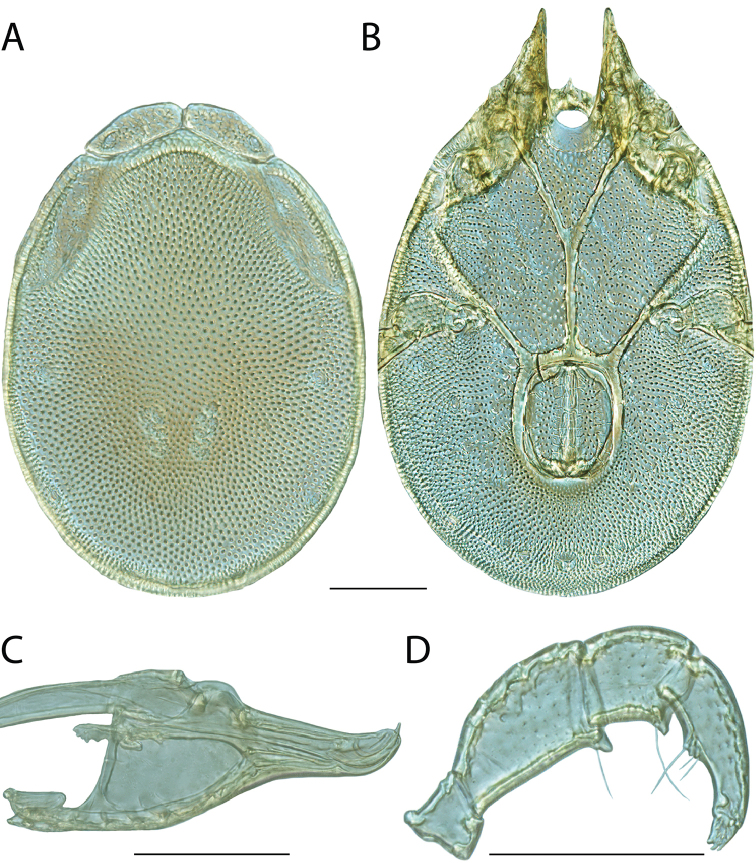
*Torrenticola nortoni* sp. n. male: **A** dorsal plates **B** venter (legs removed) **C** subcapitulum **D** pedipalp (setae not accurately depicted). Scale = 100 µm.

######## Remarks.

*Torrenticola nortoni* groups with other members of the Rusetria Complex with high support and specimens are less than 2% different in COI sequence from each other. In the all analyses, *T. mulleni* groups with the three other members of the Rusetria Complex that are found in western North America: *T. mulleni*, *T. walteri*, and *T. welbourni*. These species are 5–7% different in COI sequence from each other and together make up the Western 2-Plate Identification Group. *Torrenticola nortoni* is one of three of these that occur in California (including *T. walteri* and *T. welbourni*).

This species hypothesis is supported by low COI divergence within the species (0–2%) and high divergence between species (3–15%), and by the morphological characters outlined in the diagnosis.

####### Torrenticola occidentalis

Taxon classificationAnimaliaTrombidiformesTorrenticolidae

(Marshall, 1933)

Atractides occidentalis Marshall, 1933: 40.
Torrenticola occidentalis Mitchell, 1954: 40.

######## Material examined.

HOLOTYPE (♀): from USA, Wyoming, Medicine Bow National Forest, 1928, by JW Scott, RM280072.

######## Type deposition.

Holotype (♀) deposited in the CNC.

######## Diagnosis.

*Torrenticola occidentalis* are similar to other members of the Ellipsoidalis Group (*T. ellipsoidalis*, *T. multiforma*, and *T. leviathan*), and in being among the largest *Torrenticola* in the west (dorsum length ♀ = 700–885; ♂ = 665–850), although *T. sierrensis* are also large (dorsum length ♀ = 700–880; ♂ = 590–735) but can easily be distinguished from the Ellipsoidalis Group by being circular instead of ellipsoid or rectangular (dorsum length/width = 1.17–1.28 in *T. sierrensis*, 1.30–1.67 in Ellipsoidalis Group). *T. occidentalis* can be differentiated from *T. ellipsoidalis* by having a shorter medial suture (20 in *T. occidentalis*, 40–57.5 in *T. ellipsoidalis*) and by having more elongate anterio-lateral platelets (length/width = 2.54 in *T. occidentalis*, 2.00–2.39 in *T. ellipsoidalis*). *T. occidentalis* can be differentiated from *T. multiforma* by having stockier subcapitular rostra (length/width = 2.15 in *T. occidentalis*, 2.5–2.8 in *T. multiforma*). *T. occidentalis* can be differentiated from *T. leviathan* by having less elongate pedipalpal tibiae (length/width = 3.33 in *T. occidentalis*, 3.43–4.20 in *T. leviathan*) and a shorter dorsum (length ♀ = 770 in *T. occidentalis*, 845–870 in *T. leviathan*).

######## Re-description.

**Female (Figure 171)** (n = 1) (holotype only) with characters of the genus with following specifications.

**Dorsum**— (770 long; 590 wide) ellipsoid with pink coloration without a distinct pattern. Anterio-medial platelets (145 long; 76.25 wide). Anterio-lateral platelets (235 long; 92.5 wide) free from dorsal plate. Dgl-4 much closer to the edge of the dorsum than to the muscle scars (distance between Dgl-4 455). Dorsal plate proportions: dorsum length/width 1.31; dorsal width/distance between Dgl-4 1.30; anterio-medial platelet length/width 1.90; anterio-lateral platelet length/width 2.54; anterio-lateral/anterio-medial length 1.62.

**Gnathosoma — Subcapitulum** (370 long (ventral); 282.5 long (dorsal); 170 tall) colorless. Rostrum (145 long; 67.5 wide) short. Chelicerae (370 long) with curved fangs (75 long). Subcapitular proportions: ventral length/height 2.18; rostrum length/width 2.15. **Pedipalps** with tuberculate ventral extensions on femora and genua. Palpomeres: trochanter (50 long); femur (127.5 long); genu (87.5 long); tibia (100 long; 30 wide); tarsus (15 long). Palpomere proportions: femur/genu 1.46; tibia/femur 0.78; tibia length/width 3.33.

**Venter** — (980 long; 660 wide) colorless. Gnathosomal bay (202.5 long; 97.5 wide). Cxgl-4 subapical. **Medial suture** (20 long). **Genital plates** (235 long; 210 wide). Additional measurements: Cx-1 (385 long (total); 180 long (medial)); Cx-3 (430 wide); anterior venter (217.5 long). Ventral proportions: gnathosomal bay length/width 2.08; anterior venter/genital field length 0.93; anterior venter length/genital field width 1.04; anterior venter/medial suture 10.88.

**Male** unknown.

**Immatures** unknown.

######## Etymology.

Marshall (1933) did not specify an etymology for the specific epithet (*occidentalis*). However, surely this name refers to the type locality of this species in western United States (*occidens*, L. direction of the setting sun, west).

######## Distribution.

**Figure 170.**
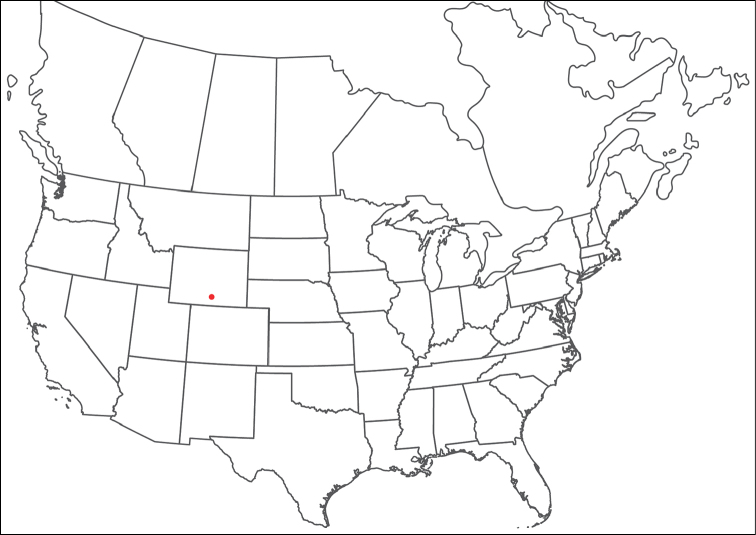
*Torrenticola occidentalis* distribution.

Known only from South-central Wyoming (Medicine Bow National Forest) (Figure 170), from a single female collected from trout stomach contents.

**Figure 171.**
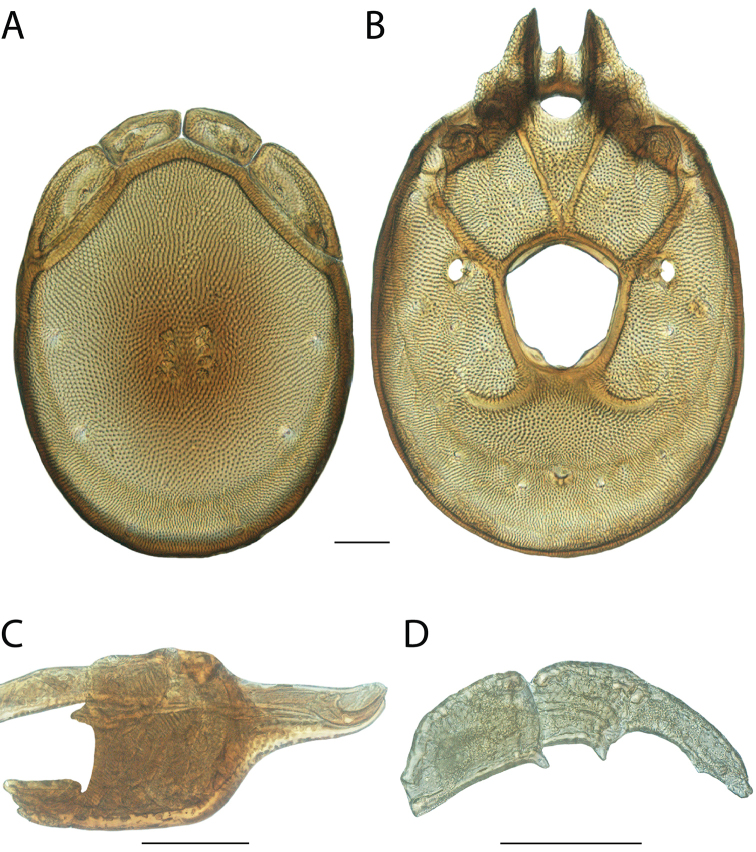
*Torrenticola occidentalis* female: **A** dorsal plates **B** venter (legs removed) **C** subcapitulum **D** pedipalp (setae not accurately depicted). Scale = 100 µm.

######## Remarks.

Unfortunately, we were unable to acquire fresh material of *Torrenticola occidentalis* and therefore this species is not included in our phylogenetic analyses. However, we were able to examine the holotype. Based upon overall similarity and a short, conical rostrum, we place this species in the Miniforma Complex and the Ellipsoidalis Identification Group

####### Torrenticola oliveri

Taxon classificationAnimaliaTrombidiformesTorrenticolidae

Fisher & Dowling sp. n.

http://zoobank.org/B5AF53E5-22E6-4E2B-82CE-36FA1291E914

######## Material examined.

HOLOTYPE (♀): from USA, Oregon, Curry County, Siskiyou National Forest; beside Road #33 between Powers and Agness, (42°39'39"N, 124°4'4"W), 2 July 1983, by IM Smith, IMS830019.

PARATYPES (4 ♀; 4 ♂): **California, United States**: 1 ♀ and 1 ♂ from Mendocino County, beside Rt. 1, 3.6 km southwest of Rt. 101, (39°51'51"N, 123°44'44"W), 5 August 1987, by IM Smith, IMS870130 • 1 ♀ and 1 ♂ from Trinity County, beside Rt. 36, 5.2 km west of Forest Glen Station, (40°23'23"N, 123°21'21"W), 6 August 1987, by IM Smith & JD Smith, IMS870132 • **Oregon, United States**: 1 ♂ (ALLOTYPE) from Curry County, Siskiyou National Forest; beside Road #33 between Powers and Agness, (42°39'39"N, 124°4'4"W), 2 July 1983, by IM Smith, IMS830019 • 1 ♀ from Curry County, Port Orford; Humbug Mountain State Park Picnic Area off Rt. 101, (42°41'41"N, 124°26'26"W), 1 July 1983, by IM Smith, IMS830013 • 1 ♂ from Curry County, Port Orford; Humbug Mountain State Park Picnic Area off Rt. 101, (42°41'41"N, 124°25'25"W), 1 July 1983, by IM Smith, IMS830012 • **Washington, United States**: 1 ♀ from Pierce County, Mt. Rainier National Park; Sunshine Point Campground, (46°44'44"N, 121°54'54"W), 30 June 1976, by IM Smith, IMS760177.

######## Type deposition.

Holotype (♀), allotype (♂), and some paratypes (2 ♀; 2 ♂) deposited in the CNC; other paratypes (2 ♀; 1 ♂) deposited in the ACUA.

######## Diagnosis.

*Torrenticola oliveri* are similar to members of the Miniforma group (*T. copipalpa*, *T. manni*, *T. miniforma*, *T. pacificensis*, *T. rockyensis*, and *T. pinocchio*) in having similar pedipalpal extensions (unique to members of this group). *T. oliveri* can be differentiated from all other members of Miniforma Complex by having a longer anterior venter (250–310 in *T. oliveri*, 173–235 in others) and more elongate pedipalpal tibiae (length/width = 3.68–4.13 in *T. oliveri*, 2.47–3.50 in others).

######## Description.

**Female (Figure 173)** (n = 5) (holotype measurements in parentheses when available) with characters of the genus with following specifications.

**Dorsum** — (615–710 (645) long; 480–545 (490) wide) ovoid with faint pink coloration without a distinct pattern. Anterio-medial platelets (125–157.5 (130) long; 65–75 (65) wide). Anterio-lateral platelets (210–245 (210) long; 75–87.5 (75) wide) free from dorsal plate. Dgl-4 much closer to the edge of the dorsum than to the muscle scars (distance between Dgl-4 370–430 (395)). Dorsal plate proportions: dorsum length/width 1.27–1.34 (1.32); dorsal width/distance between Dgl-4 1.24–1.30 (1.24); anterio-medial platelet length/width 1.92–2.10 (2.00); anterio-lateral platelet length/width 2.63–2.97 (2.80); anterio-lateral/anterio-medial length 1.56–1.68 (1.62).

**Gnathosoma — Subcapitulum** (370–397.5 (397.5) long (ventral); 285–297.5 (297.5) long (dorsal); 112.5–122.5 (117.5) tall) colorless. Rostrum (150–160 (160) long; 47.5–50 (50) wide). Chelicerae (380–415 (415) long) with curved fangs (55–65 (60) long). Subcapitular proportions: ventral length/height 3.08–3.38 (3.38); rostrum length/width 3.00–3.20 (3.20). **Pedipalps** with tuberculate ventral extensions on femora and dentate, tuberculate ventral extensions on genua. Palpomeres: trochanter (33.75–37.5 (37.5) long); femur (110–118.75 (118.75) long); genu (75–83.75 (83.75) long); tibia (80–87.5 (85) long; 20–23.75 (22.5) wide); tarsus (12.5–15 (15) long). Palpomere proportions: femur/genu 1.38–1.53 (1.42); tibia/femur 0.72–0.75 (0.72); tibia length/width 3.68–4.00 (3.78).

**Venter** — (770–835 (805) long; 550–650 (580) wide) with faint pink coloration. Gnathosomal bay (155–167.5 (167.5) long; 70–90 (72.5) wide). Cxgl-4 subapical. **Medial suture** (80–95 (80) long). **Genital plates** (190–205 (205) long; 170–180 (180) wide). Additional measurements: Cx-1 (320–340 (320) long (total); 140–185 (155) long (medial)); Cx-3 (340–400 (360) wide); anterior venter (250–290 (250) long). Ventral proportions: gnathosomal bay length/width 1.75–2.31 (2.31); anterior venter/genital field length 1.22–1.45 (1.22); anterior venter length/genital field width 1.39–1.63 (1.39); anterior venter/medial suture 2.86–3.38 (3.13).

**Male (Figure 174)** (n = 5) (allotypic measurements in parentheses when available) with characters of the genus with following specifications.

**Dorsum** — (575–640 (590) long; 445–470 (470) wide ovoid with faint pink coloration without a distinct pattern. Anterio-medial platelets (125–140 (125) long; 60–75 (60) wide). Anterio-lateral platelets (205–220 (220) long; 65–90 (80) wide) free from dorsal plate. Dgl-4 much closer to the edge of the dorsum than to the muscle scars (distance between Dgl-4 355–375 (375)). Dorsal plate proportions: dorsum length/width 1.26–1.39 (1.26); dorsal width/distance between Dgl-4 1.25–1.28 (1.25); anterio-medial platelet length/width 1.87–2.17 (2.08); anterio-lateral platelet length/width 2.39–3.15 (2.75); anterio-lateral/anterio-medial length 1.54–1.76 (1.76).

**Gnathosoma — Subcapitulum** (325–367.5 (355) long (ventral); 260–285 (262.5) long (dorsal); 105–110 (110) tall) colorless. Rostrum (135–145 (135) long; 45–47.5 (45) wide). Chelicerae (350–410 (365) long) with curved fangs (55–55 (55) long). Subcapitular proportions: ventral length/height 3.10–3.34 (3.23); rostrum length/width 3.00–3.11 (3.00). **Pedipalps** with tuberculate ventral extensions on femora and dentate, tuberculate ventral extensions on genua. Palpomeres: trochanter (32.5–35 (35) long); femur (105–107.5 (107.5) long); genu (75–80 (80) long); tibia (82.5–90 (87.5) long; 20–22.5 (21.25) wide); tarsus (12.5–15 (15) long). Palpomere proportions: femur/genu 1.34–1.40 (1.34); tibia/femur 0.79–0.86 (0.81); tibia length/width 4.00–4.13 (4.12).

**Venter** — (725–795 (725) long; 515–540 (540) wide) with faint pink coloration. Gnathosomal bay (142.5–150 (150) long; 60–70 (70) wide). Cxgl-4 subapical. **Medial suture** (105–130 (105) long). **Genital plates** (155–165 (160) long; 120–130 (130) wide). Additional measurements: Cx-1 (290–310 (295) long (total); 145–165 (150) long (medial)); Cx-3 (330–350 (350) wide); anterior venter (270–310 (270) long). Ventral proportions: gnathosomal bay length/width 2.14–2.40 (2.14); anterior venter/genital field length 1.69–1.88 (1.69); anterior venter length/genital field width 2.08–2.58 (2.08); anterior venter/medial suture 2.27–2.57 (2.57).

**Immatures** unknown.

######## Etymology.

Specific epithet (*oliveri*) named in honor of comedian John Oliver and his team, whose commentary on *Last Week Tonight* breaches the realm of comedy and enters journalism. News and politics, like science, require both challenging misinformation and effectively communicating those challenges—tasks Oliver has humbly mastered.

######## Distribution.

**Figure 172.**
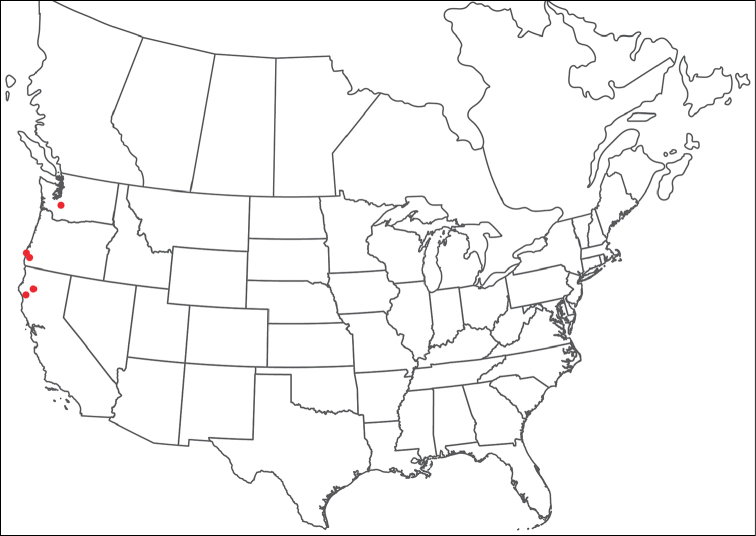
*Torrenticola oliveri* sp. n. distribution.

Pacific Northwest (Figure 172).

**Figure 173.**
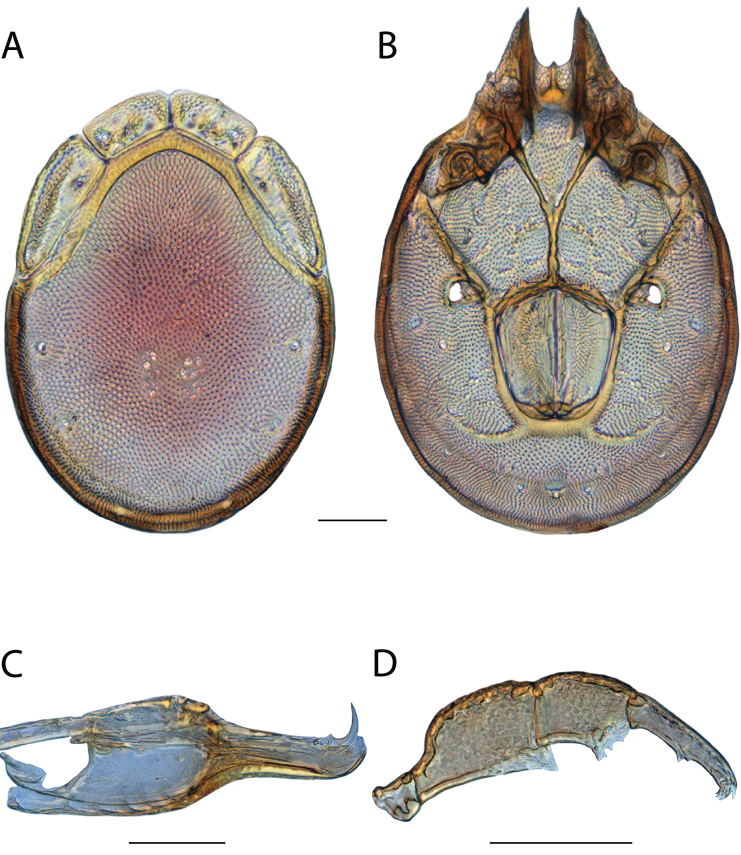
*Torrenticola oliveri* sp. n. female: **A** dorsal plates **B** venter (legs removed) **C** subcapitulum **D** pedipalp (setae not accurately depicted). Scale = 100 µm.

**Figure 174.**
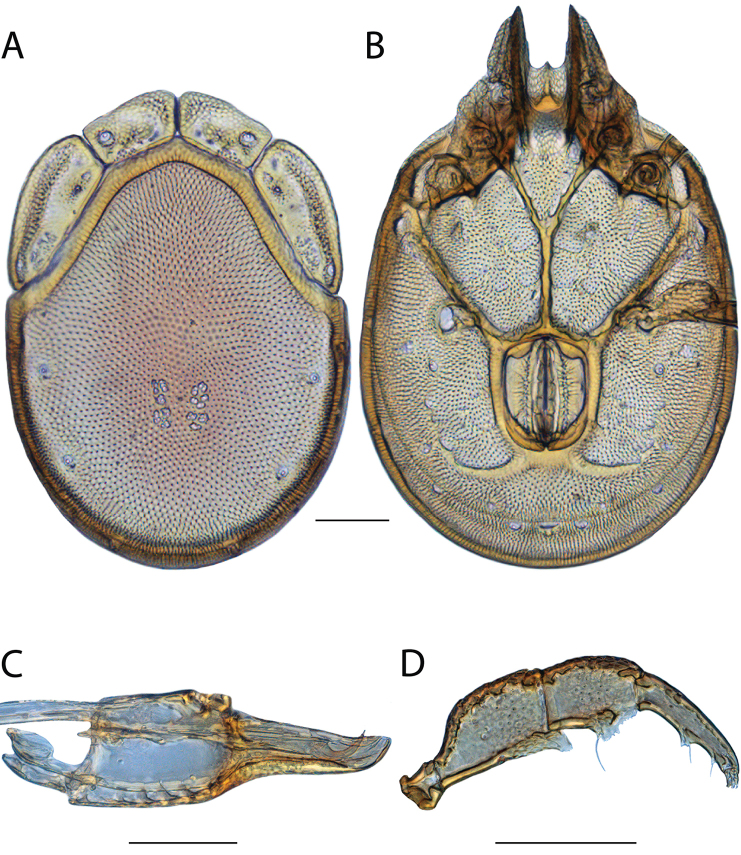
*Torrenticola oliveri* sp. n. male: **A** dorsal plates **B** venter (legs removed) **C** subcapitulum **D** pedipalp (setae not accurately depicted). Scale = 100 µm.

######## Remarks.

Unfortunately, we were unable to acquire fresh material of *Torrenticola oliveri* and therefore this species is not included in our phylogenetic analyses. However, we were able to examine morphology with material preserved in GAW. The overall appearance, small size, distribution, and distinctive flange on pedipalpal genua place this species in the Miniforma Identification Group within the Miniforma Complex.

####### Torrenticola olliei

Taxon classificationAnimaliaTrombidiformesTorrenticolidae

Fisher & Dowling sp. n.

http://zoobank.org/EF825E5C-935A-4BDD-AEDA-A599A4839D7A

######## Material examined.

HOLOTYPE (♀): from USA, Oregon, Douglas County, Umpqua National Forest, Umpqua River (43°17'28"N, 122°37'12"W), 12 Aug 2013, by JC O’Neill, & WA Nelson, JNOW 13-0812-006.

PARATYPES (9 ♀; 12 ♂): **British Columbia, Canada**: 1 ♀ and 1 ♂ from Vancouver Island, Malahat, Goldstream Provincial Park, Goldstream River, 26 Jun 1979, by IM Smith, IMS790028A • **California, USA**: 1 ♀ and 1 ♂ from Humboldt County, Honeydew, Mattole River, beside road to Bull Creek on east side of bridge, 8 Aug 1987, by IM Smith, IMS870135A • 1 ♀ and 1 ♂ from Trinity County, Trinity River, beside Route 299, 8.7 kilometers northwest of Del Loma, 9 Aug 1987, by IM Smith, IMS870137A • 1 ♀ from Trinity County, Weaver Creek, beside Route 299, 4.3 kilometers north of Route 3 West, 9 Aug 1987, by IM Smith, IMS870138A & IMS870138B • **Oregon, USA**: 1 ♂ from Coos County, Siskiyou National Forest, Road 33 between Powers & Agness, Daphne Grove campground, Coquille River, 2 Jul 1983, by IM Smith, IMS830016 • 3 ♀ and 6 ♂ from Curry County, Port Orford, Butler Bar campground, Elk River, 25-26 Jun 1976, by IM Smith, IMS760163 • 1 ♀ from Curry County, Port Orford, small spring run beside road from Humbug Mountain State Park to McGribble campground, 25 Jun 1976, by IM Smith, IMS760161 • 1 ♂ (ALLOTYPE) from Douglas County, Umpqua National Forest, Umpqua River (43°17'28"N, 122°37'12"W), 12 Aug 2013, by JC O’Neill, & WA Nelson, JNOW 13-0812-006 • 1 ♀ and 1 ♂ from Douglas County, Umpqua National Forest, Umpqua River (43°17'28"N, 122°37'12"W), 12 Aug 2013, by JC O’Neill, & WA Nelson, JNOW 13-0812-006.

######## Type deposition.

Holotype (♀), allotype (♂), and most paratypes (5 ♀; 6 ♂) deposited in the CNC; other paratypes (4 ♀; 5 ♂) deposited in the ACUA.

######## Diagnosis.

*Torrenticola olliei* are similar to other members of the Tricolor Complex (*T. bittikoferae*, *T. hoosieri*, *T. larvata*, *T. pearsoni*, *T. sierrensis*, *T. tricolor*, *T. trimaculata*, *T. unimaculata*, *T. cardia*, *T. kringi*, *T. dimorpha*, and *T. mohawk*) in having a short, conical rostrum. *T. olliei* can be differentiated from most *Torrenticola*, including most other members of the Tricolor Complex, by having a very short rostrum (length/width = 1.56–1.81 in *T. olliei*, 1.80–3.13 in others), except *T. bittikoferae* (1.81–1.9) and *T. dimorpha* (1.3–1.6). Additionally, males have a shorter anterior venter than all other Tricolor Complex (♂ = 205–225 in *T. olliei*, 230–335 in others), except *T. kringi* (220–255), and both males and females have a wider genital field than most other Tricolor Complex (♀ = 190–202.5 in *T. olliei*, 145–180 in others; ♂ = 130–137.5 in *T. olliei*, 92.5–120 in others) except for *T. sierrensis* (♀ = 180–212.5; ♂ = 135–175) and female *T. leviathan* (180–195). Finally, *T. olliei* can be differentiated from most Tricolor Complex, except for *T. sierrensis*, by being distributed in the west (all others are eastern).

######## Description.

**Female (Figure 176)** (n=5) (holotype measurements in parentheses when available) with characters of the genus with following specifications.

**Dorsum** — (690–900 (690) long; 500–670 (500) wide) ovoid with orange coloration separated into posterior and anterior portions with faint orange medially. Anterio-medial platelets (130–172.5 (130) long; 60–80 (60) wide). Anterio-lateral platelets (190–235 (190) long; 70–92.5 (70) wide) free from dorsal plate. Dgl-4 much closer to the edge of the dorsum than to the muscle scars (distance between Dgl-4 360–470 (360)). Dorsal plate proportions: dorsum length/width 1.34–1.42 (1.38); dorsal width/distance between Dgl-4 1.31–1.43 (1.39); anterio-medial platelet length/width 1.95–2.23 (2.17); anterio-lateral platelet length/width 2.34–2.71 (2.71); anterio-lateral/anterio-medial length 1.36–1.46 (1.46).

**Gnathosoma — Subcapitulum** (245–287.5 (245) long (ventral); 155–225 (155) long (dorsal); 137.5–152.5 (137.5) tall) colorless and tall. Rostrum (85–112.5 (85) long; 50–67.5 (50) wide) short and conical. Chelicerae (225–305 (225) long) with curved fangs (55–65 (55) long). Subcapitular proportions: ventral length/height 1.78–1.92 (1.78); rostrum length/width 1.62–1.76 (1.70). **Pedipalps** with tuberculate ventral extensions on femora and genua. Palpomeres: trochanter (37.5–50 long); femur (87.5–122.5 (87.55) long); genu (58.75–82.5 (60) long); tibia (75–102.5 (75) long; 25–33.75 (25) wide); tarsus (25–30 (26.25) long). Palpomere proportions: femur/genu 1.46–1.51 (1.46); tibia/femur 0.84–0.90 (0.86); tibia length/width 3.00–3.08 (3.00).

**Venter** — (790–1050 (810) long; 550–765 (550) wide) colorless. Gnathosomal bay (120–170 (150) long; 75–97.5 (75) wide). Cxgl-4 subapical. **Medial suture** (12.5–50 (12.5) long). **Genital plates** (202.5–230 (212.5) long; 190–202.5 (190) wide). Additional measurements: Cx-1 (260–375 (280) long (total); 130–195 (130) long (medial)); Cx-3 (350–465 (350) wide); anterior venter (162.5–255 (162.5) long). Ventral proportions: gnathosomal bay length/width 1.37–2.00 (2.00); anterior venter/genital field length 0.76–1.11 (0.76); anterior venter length/genital field width 0.85–1.28 (0.86); anterior venter/medial suture 5.10–13.00 (13.00).

**Male (Figure 177)** (n = 5) (allotypic measurements in parentheses when available) with characters of the genus with following specifications.

**Dorsum** — (560–640 (570) long; 410–460 (410) wide) ovoid with orange coloration separated into posterior and anterior portions with faint orange medially. Anterio-medial platelets (102.5–125 (107.5) long; 50–70 (56.25) wide). Anterio-lateral platelets (170–187.5 (170) long; 62.5–80 (62.5) wide) free from dorsal plate. Dgl-4 much closer to the edge of the dorsum than to the muscle scars (distance between Dgl-4 320–375 (320)). Dorsal plate proportions: dorsum length/width 1.33–1.44 (1.39); dorsal width/distance between Dgl-4 1.19–1.33 (1.28); anterio-medial platelet length/width 1.71–2.25 (1.91); anterio-lateral platelet length/width 2.34–2.72 (2.72); anterio-lateral/anterio-medial length 1.50–1.68 (1.58).

**Gnathosoma — Subcapitulum** (197.5–220 (212.5) long (ventral); 147.5–160 (159) long (dorsal); 115–125 (115) tall) colorless and tall. Rostrum (70–82.5 (72.5) long; 40–50 (40) wide) short and conical. Chelicerae (195–220 long) with curved fangs (47.5–52.5 long). Subcapitular proportions: ventral length/height 1.65–1.85 (1.85); rostrum length/width 1.56–1.81 (1.81). **Pedipalps** with tuberculate ventral extensions on femora and genua. Palpomeres: trochanter (32.5–35 (32.5) long); femur (75–77.5 (76.25) long); genu (52.5–55 (52.5) long); tibia (67.5–75 (67.5) long; 22.5–25 (22.5) wide); tarsus (20–25 (20) long). Palpomere proportions: femur/genu 1.41–1.48 (1.45); tibia/femur 0.89–0.97 (0.89); tibia length/width 2.70–3.00 (3.00).

**Venter** — (650–710 (650) long; 444–530 (444) wide) colorless. Gnathosomal bay (122.5–137.5 (127.5) long; 62.5–75 (62.5) wide). Cxgl-4 subapical. **Medial suture** (70–80 (75) long). **Genital plates** (165–172.5 (165) long; 130–137.5 (130) wide). Additional measurements: Cx-1 (234–270 (235) long (total); 102–135 (102) long (medial)); Cx-3 (311–355 (312) wide); anterior venter (200–225 (207.5) long). Ventral proportions: gnathosomal bay length/width 1.70–2.04 (2.04); anterior venter/genital field length 1.19–1.30 (1.26); anterior venter length/genital field width 1.54–1.67 (1.60); anterior venter/medial suture 2.67–2.93 (2.77).

**Immatures** unknown.

######## Etymology.

Specific epithet (*olliei*) named in honor of Ollie—pet Boston Terrier of JRF and DMF—whose brachycephaly resembles the short rostrum of this species, the shortest of all *Torrenticola*.

######## Distribution.

**Figure 175.**
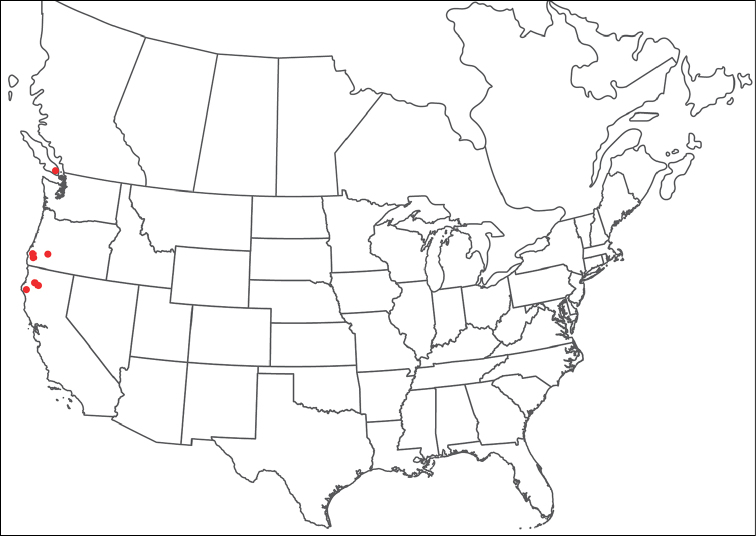
*Torrenticola olliei* sp. n. distribution.

Pacific coastal mountains (Figure 175).

**Figure 176.**
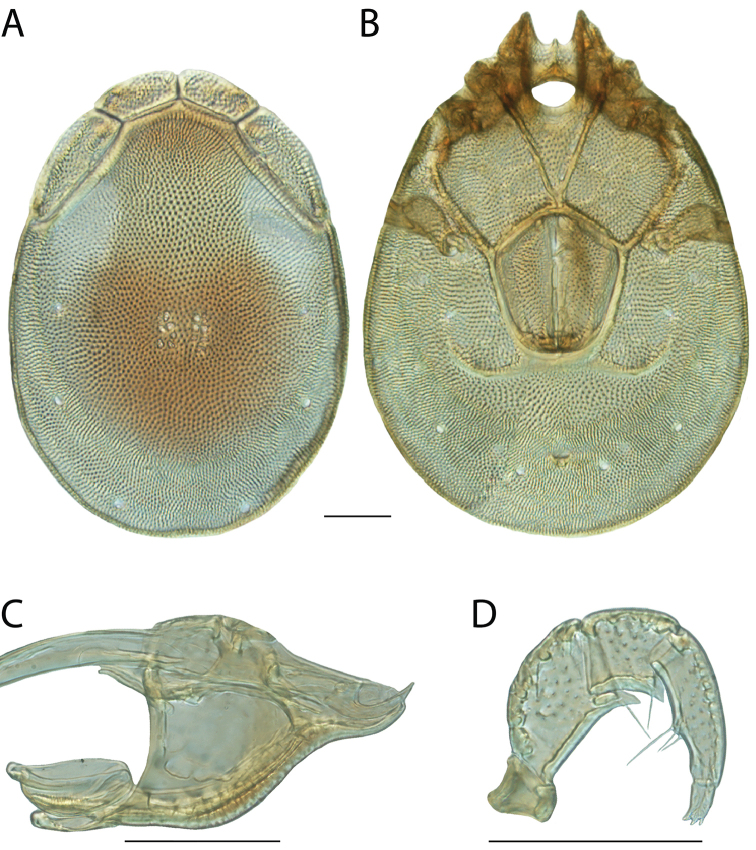
*Torrenticola olliei* sp. n. female: **A** dorsal plates **B** venter (legs removed) **C** subcapitulum **D** pedipalp (setae not accurately depicted). Scale = 100 µm.

**Figure 177.**
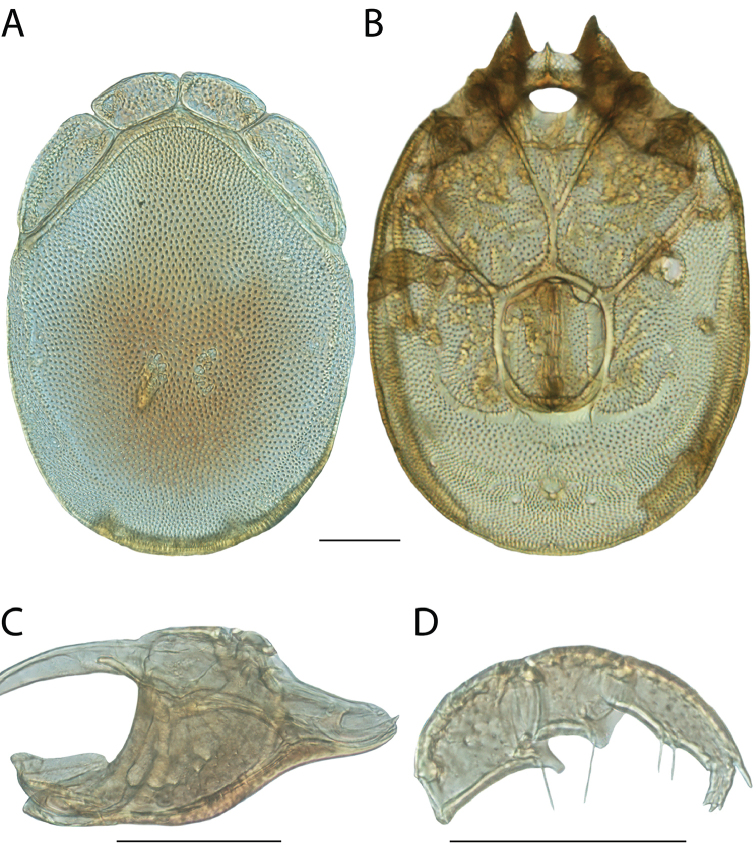
*Torrenticola olliei* sp. n. male: **A** dorsal plates **B** venter (legs removed) **C** subcapitulum **D** pedipalp (setae not accurately depicted). Scale = 100 µm.

######## Remarks.

*Torrenticola olliei* groups with other members of the Tricolor Complex with high support in all analyses and group with *T. sierrensis* to form the western portion of this complex. Only one specimen was analyzed phylogenetically, so intraspecific variation remains unknown, but that specimen is greater than 6% different from all specimens of *T. sierrensis*. This species hypothesis is supported by high divergence between species (3–15%), and by the morphological characters outlined in the diagnosis.

####### Torrenticola oregonensis

Taxon classificationAnimaliaTrombidiformesTorrenticolidae

Fisher & Dowling sp. n.

http://zoobank.org/4DD6B282-08DE-4B42-93F5-AEF20F62105A

######## Material examined.

HOLOTYPE (♀): from USA, Oregon, Grant County, Prairie City; Strawberry Forest Camp, (44°19'19"N, 118°40'40"W), 17 June 1976, by IM Smith, IMS760126.

PARATYPES (3 ♀; 3 ♂): **Oregon, USA**: 1 ♂ (ALLOTYPE) from Grant County, Prairie City; Strawberry Forest Camp, (44°19'19"N, 118°40'40"W), 17 June 1976, by IM Smith, IMS760126 • 1 ♀ from Benton County, Philomath; Marys Peak Campground, (44°30'30"N, 123°33'33"W), 23 June 1976, by IM Smith, IMS760147 • 2 ♂ from Benton County, Philomath; Marys Peak Campground, (44°30'30"N, 123°33'33"W), 27 June 1983, by IM Smith & AB Smith, IMS830006 • 1 ♀ from Benton County, Philomath; Marys Peak Campground, (44°30'30"N, 123°33'33"W), 28 June 1983, by IM Smith, IMS830008 • 1 ♀ from Grant County, Prairie City; beside Rt. 26 just east of Dixie Pass, (44°32'32"N, 118°32'32"W), 17 June 1976, by IM Smith, IMS760125.

######## Type deposition.

Holotype (♀), allotype (♂), and some paratypes (2 ♀; 1 ♂) deposited in the CNC; other paratypes (1 ♀; 1 ♂) deposited in the ACUA.

######## Diagnosis.

*Torrenticola oregonensis* are similar to the other member of Tahoei group, *T. tahoei* by subcapitulum shape, having purple coloration restricted posteriorly and being distributed in the west and to members of the Nigroalba Group (*T. flangipalpa*, *T. nigroalba*, *T. solisorta*, and *T. dentirostra*) in having purple dorsal coloration restricted posteriorly. *T. oregonensis* can be differentiated from the Nigroalba Group by being larger (dorsum length ♀ = 760–840 in *T. oregonensis*, 475–565 in Nigroalba Group; ♂ = 690–820 in *T. oregonensis*, 425–510 in Nigroalba Group) and distributed in Oregon (Nigroalba Group are eastern). *T. oregonensis* can be differentiated from *T. tahoei* by being larger (dorsum length ♀ = 760–840 in *T. oregonensis*, 600–720 in *T. tahoei*; ♂ = 690–820 in *T. oregonensis*, 560–650 in *T. tahoei*; dorsum width ♀ = 560–640 in *T. oregonensis*, 430–515 in *T. tahoei*; ♂ = 520–605 in *T. oregonensis*, 400–460 in *T. tahoei*) and a less elongate subcapitulum (ventral length/width = 2.63–2.74 in *T. oregonensis*, 3.25–4.11 in *T. tahoei*).

######## Description.

**Female (Figure 179)** (n = 4) (holotype measurements in parentheses when available) with characters of the genus with following specifications.

**Dorsum** — (760–840 (760) long; 560–640 (560) wide) ovoid with faint pink coloration medially, occasionally continuing posteriorly. Anterio-medial platelets (145–163.75 (145) long; 72.5–87.5 (72.5) wide). Anterio-lateral platelets (222.5–247.5 (222.5) long; 97.5–102.5 (100) wide) free from dorsal plate. Dgl-4 much closer to the edge of the dorsum than to the muscle scars (distance between Dgl-4 435–495 (435)). Dorsal plate proportions: dorsum length/width 1.30–1.36 (1.36); dorsal width/distance between Dgl-4 1.27–1.29 (1.29); anterio-medial platelet length/width 1.85–2.21 (2.00); anterio-lateral platelet length/width 2.23–2.45 (2.23); anterio-lateral/anterio-medial length 1.48–1.56 (1.53).

**Gnathosoma — Subcapitulum** (380–415 (380) long (ventral); 280–307.5 (280) long (dorsal); 140–157.5 (140) tall) colorless. Rostrum (152.5–170 (152.5) long; 60–65 (60) wide). Chelicerae (380–425 (380) long) with curved fangs (70–75 (70) long). Subcapitular proportions: ventral length/height 2.63–2.71 (2.71); rostrum length/width 2.48–2.62 (2.54). **Pedipalps** with tuberculate ventral extensions on femora and tuberculate extensions on genua that are slightly dentate. Palpomeres: trochanter (42.5–46.25 (42.5) long); femur (115–133.75 (115) long); genu (83.75–95 (83.75) long); tibia (100–110 (100) long; 25–27.5 (25) wide); tarsus (16.25–17.5 (17.5) long). Palpomere proportions: femur/genu 1.37–1.43 (1.37); tibia/femur 0.78–0.87 (0.87); tibia length/width 3.81–4.00 (4.00).

**Venter** — (900–990 (900) long; 660–750 (660) wide) colorless. Gnathosomal bay (170–185 (170) long; 75–100 (75) wide). Cxgl-4 subapical. **Medial suture** (60–80 (60) long). **Genital plates** (210–230 (210) long; 185–205 (185) wide). Additional measurements: Cx-1 (330–370 (330) long (total); 162.5–190 (170) long (medial)); Cx-3 (410–470 (410) wide); anterior venter (230–270 (230) long). Ventral proportions: gnathosomal bay length/width 1.80–2.27 (2.27); anterior venter/genital field length 1.04–1.22 (1.10); anterior venter length/genital field width 1.24–1.32 (1.24); anterior venter/medial suture 3.00–3.83 (3.83).

**Male (Figure 180)** (n = 3) (allotypic measurements in parentheses when available) with characters of the genus with following specifications.

**Dorsum** — (690–820 (820) long; 520–605 (605) wide) ovoid with faint pink coloration medially, occasionally continuing posteriorly. Anterio-medial platelets (140–155 (150) long; 67.5–82.5 (82.5) wide). Anterio-lateral platelets (215–247.5 (247.5) long; 87.5–102.5 (102.5) wide) free from dorsal plate. Dgl-4 much closer to the edge of the dorsum than to the muscle scars (distance between Dgl-4 420–480 (480)). Dorsal plate proportions: dorsum length/width 1.33–1.36 (1.36); dorsal width/distance between Dgl-4 1.24–1.26 (1.26); anterio-medial platelet length/width 1.82–2.30 (1.82); anterio-lateral platelet length/width 2.25–2.46 (2.41); anterio-lateral/anterio-medial length 1.45–1.65 (1.65).

**Gnathosoma — Subcapitulum** (370–380 (380) long (ventral); 275–290 (290) long (dorsal); 135–140 (140) tall) colorless. Rostrum (145–150 (150) long; 52.5–60 (60) wide). Chelicerae (375–395 (395) long) with curved fangs (65–70 (70) long). Subcapitular proportions: ventral length/height 2.64–2.74 (2.71); rostrum length/width 2.50–2.76 (2.50). **Pedipalps** with tuberculate ventral extensions on femora and tuberculate extensions on genua that are slightly dentate. Palpomeres: trochanter (40–43.75 (43.75) long); femur (115–127.5 (127.5) long); genu (82.5–87.5 (87.5) long); tibia (97.5–102.5 (102.5) long; 25–26.25 (26.25) wide); tarsus (15–16.25 (15) long). Palpomere proportions: femur/genu 1.31–1.46 (1.46); tibia/femur 0.80–0.85 (0.80); tibia length/width 3.90–3.90 (3.90).

**Venter** — (840–930 (930) long; 610–710 (710) wide) colorless. Gnathosomal bay (157.5–172.5 (157.5) long; 70–100 (100) wide). Cxgl-4 subapical. **Medial suture** (100–120 (120) long). **Genital plates** (165–175 (175) long; 130–140 (140) wide). Additional measurements: Cx-1 (330–340 (340) long (total); 160–180 (180) long (medial)); Cx-3 (390–455 (455) wide); anterior venter (275–325 (325) long). Ventral proportions: gnathosomal bay length/width 1.58–2.46 (1.58); anterior venter/genital field length 1.65–1.86 (1.86); anterior venter length/genital field width 2.11–2.32 (2.32); anterior venter/medial suture 2.71–2.75 (2.71).

**Immatures** unknown.

######## Etymology.

Specific epithet (*oregonensis*) refers to the distribution of this species, which was paradoxically found in several ecoregions across Oregon, but not in surrounding areas.

######## Distribution.

**Figure 178.**
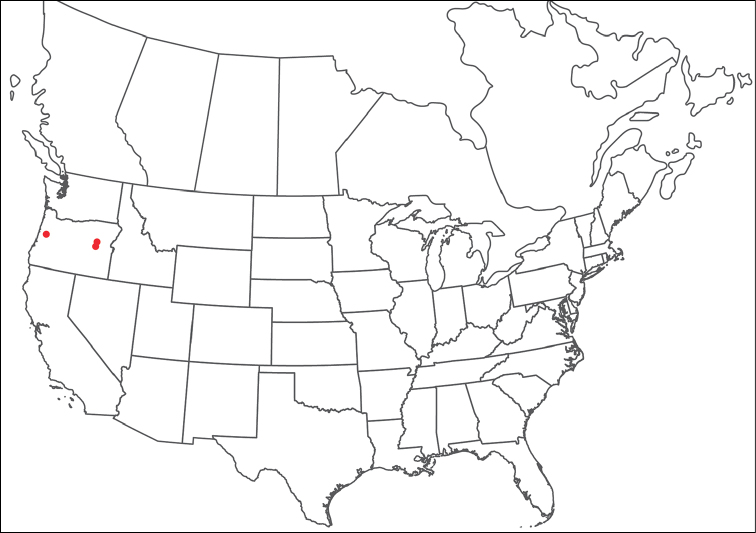
*Torrenticola oregonensis* sp. n. distribution.

Oregon (Figure 178).

**Figure 179.**
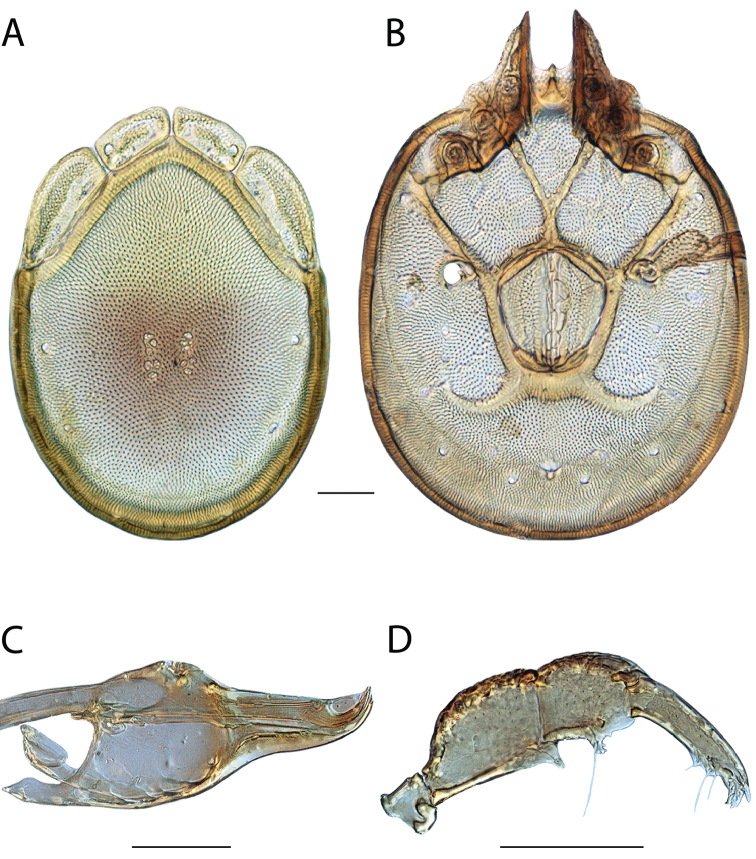
*Torrenticola oregonensis* sp. n. female: **A** dorsal plates **B** venter (legs removed) **C** subcapitulum **D** pedipalp (setae not accurately depicted). Scale = 100 µm.

**Figure 180.**
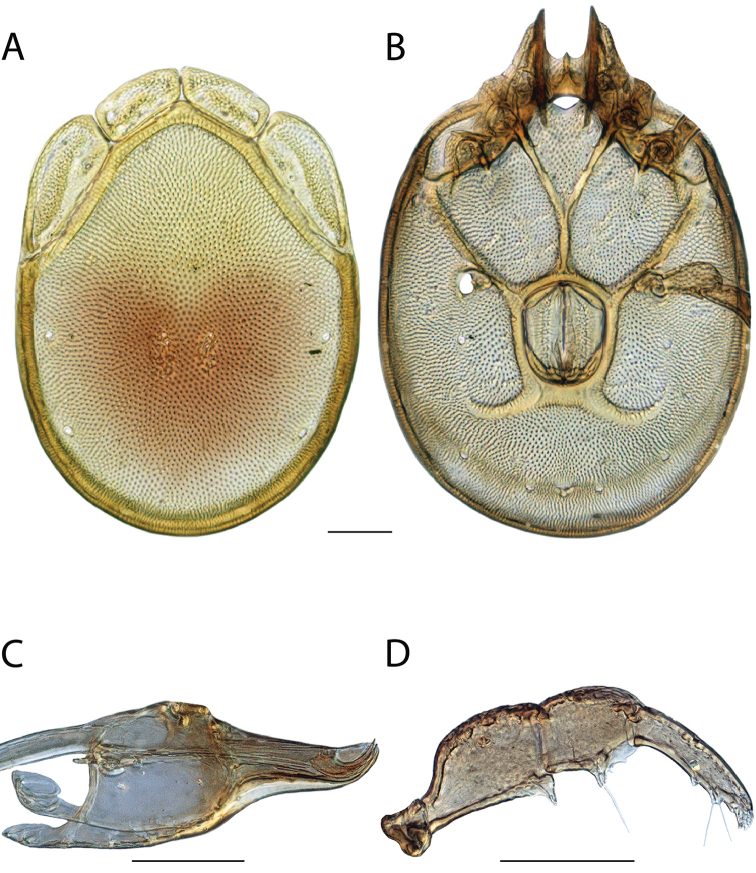
*Torrenticola oregonensis* sp. n. male: **A** dorsal plates **B** venter (legs removed) **C** subcapitulum **D** pedipalp (setae not accurately depicted). Scale = 100 µm.

######## Remarks.

Unfortunately, we were unable to acquire fresh material of *Torrenticola oregonensis* and therefore this species is not included in our phylogenetic analyses. However, we were able to examine morphology with material preserved in GAW. Due to its unique set of characteristics, we are unable to place this species into a species group or complex.

####### Torrenticola pacificensis

Taxon classificationAnimaliaTrombidiformesTorrenticolidae

Fisher & Dowling sp. n.

http://zoobank.org/CEB459C5-08DD-4A1B-A84F-0B2113AAEC2D

######## Material examined.

HOLOTYPE (♀): from USA, Washington, Clallam County, Green Creek (48°10'45"N, 124°12'21"W), 24 Jul 2013, by JC O’Neill, & WA Nelson, JNOW 13-0724-005.

PARATYPES (34 ♀; 31 ♂): **British Columbia, Canada**: 2 ♀ and 1 ♂ from Vancouver Island, Caycuse, Nixon Creek, 8 Jul 1976, by IM Smith, IMS760197 & IMS760198 • 1 ♀ and 1 ♂ from Vancouver Island, beside Highway 4, 35.6 kilometers east of Pacific Rim Road, 9 Jul 1976, by IM Smith, IMS760206 • 1 ♂ from Vancouver Island, beside Highway 4, 1.3 kilometers east of Pacific Rim Road, Lost Shoe Creek, 9 Jul 1976, by IM Smith, IMS760202 • 2 ♀ and 2 ♂ from Vancouver Island, Lake Cowichan, Skutz Falls, Skutz Creek, near Cowichan River, 9 Jul 1979, by IM Smith, IMS790036A & IMS790036B • 1 ♀ from Vancouver Island, Lake Cowichan, Cowichan River, above Skutz Falls, 9 Jul 1979, by IM Smith, IMS790035 • 1 ♀ and 1 ♂ from Vancouver Island, Lake Cowichan, South Shore Road, north of Mesachie Lake, Robertson River, 4 Jul 1976, by IM Smith, IMS760183A • 1 ♀ and 1 ♂ from Vancouver Island, Lake Cowichan, South Shore Road, north of Mesachie Lake, tributary of Robertson River, 10 Jul 1976, by IM Smith, IMS760183B • 1 ♀ and 1 ♂ from Vancouver Island, Lake Cowichan, South Shore Road, north of Mesachie Lake, tributary of Robertson River, 4-10 Jul 1976, by IM Smith, IMS760182 • 1 ♀ and 1 ♂ from Vancouver Island, Malahat, Goldstream Provincial Park, Goldstream River, 26 Jun 1979, by IM Smith, IMS790028A • 1 ♀ and 1 ♂ from Vancouver Island, Malahat, Goldstream Provincial Park, near Mt. Finlayson Trail, 26 Jun 1979, by IM Smith, IMS790027A • 1 ♂ from Vancouver Island, beside South Shore Road, 2.3 kilometers south of Caycuse, 26 Jul 1979, by IM Smith, IMS790052 • 1 ♂ from Vancouver Island, beside North Shore Road, 1.7 kilometers north of Lake Cowichan, 7 Jun 1979, by IM Smith, IMS790008C • 1 ♀ and 1 ♂ from Vancouver Island, North Shore Road, 3.2 kilometers south of Youbou, 4 Jul 1976, by IM Smith, IMS760190 • 1 ♂ from Vancouver Island, Youbou, North Shore Road, Shaw Creek, 4.3 kilometers south of north end of Cowichan Lake, by IM Smith, IMS760196 • **Oregon, USA**: 1 ♀ and 1 ♂ from Benton County, Marys Peak near Philomath, Parker Creek, 27-28 Jun 1983, by IM Smith & AB Smith, IMS830006 • 1 ♀ from Benton County, Marys Peak near Philomath, Parker Creek, 28 Jun 1983, by IM Smith, IMS830007 • 1 ♂ from Benton County, Philomath, Marys Peak campground, Park Creek, 23 Jun 1976, by IM Smith, IMS760146 • 1 ♀ from Coos County, Siskiyou National Forest, road 33 between Powers and Agness, Coal Creek, 2 Jul 1983, by IM Smith, IMS830015 • 1 ♀ from Coos County, Siskiyou National Forest, road 33 between Powers and Agness, 0.5 kilometers east of Daphne Grove campground, 2 Jul 1983, by IM Smith, IMS830017 • 3 ♀ and 2 ♂ from Curry County, Port Orford, small spring run beside road from Humbug Mountain State Park to McGribble campground, 25 Jun 1976, IMS760161 • 1 ♂ from Curry County, Port Orford, Humbug Mountain State Park Picnic Area, Brush Creek, beside Route 1, 3 Jul 1983, by IM Smith, IMS830020A • 1 ♀ and 1 ♂ from Curry County, Siskiyou National Forest, road 33 between Powers and Agness, North Fork of Foster Creek, 2 Jul 1983, by IM Smith, IMS830019 • 1 ♀ from Lane County, Gate Creek (44°8'48"N, 122°34'20"W), 11 Aug 2013, by JC O’Neill, & WA Nelson, JNOW 13-0811-001 • 1 ♀ and 1 ♂ from Lincoln County, Blackberry campground near Tidewater, Alsea River, 28 Jun 1983, by IM Smith & AB Smith, IMS830009 • 4 ♀ and 2 ♂ from Marion County, Marion Forks Riverside campground, North Fork of Santiam River, 22 Jun 1976, by IM Smith, IMS760145 • 2 ♀ and 2 ♂ from Multnomah County, Columbia River Scenic Highway, stream below Horsetail Falls, 27 Jun 1983, by IM Smith, IMS830005 • 1 ♀ from Tillamook County, Siuslaw National Forest, Alder Creek (45°9'27"N, 123°47'60"W), 6 Aug 2013, by JC O’Neill, & WA Nelson, JNOW 13-0806-002 • **Washington, USA**: 1 ♂ (ALLOTYPE) from Clallam County, Green Creek (48°10'45"N, 124°12'21"W), 24 Jul 2013, by JC O’Neill, & WA Nelson, JNOW 13-0724-005 • 2 ♀ and 1 ♂ from Clallam County, Green Creek (48°10'45"N, 124°12'21"W), 24 Jul 2013, by JC O’Neill, & WA Nelson, JNOW 13-0724-005 • 2 ♀ and 1 ♂ from Lewis County, Gifford Pinchot National Forest, Snake Creek (46°38'52"N, 121°43'8"W), 23 Jul 2013, by JC O’Neill, & WA Nelson, JNOW 13-0723-006 • 1 ♀ and 3 ♂ from Snohomish County, Mount Baker National Forest, Marten River (48°4'19"N, 121°36'24"W), 28 Jul 2013, by JC O’Neill, & WA Nelson, JNOW 13-0728-002.

######## Type deposition.

Holotype (♀), allotype (♂), and most paratypes (29 ♀; 325♂) deposited in the CNC; other paratypes (5 ♀; 5 ♂) deposited in ACUA.

######## Diagnosis.

*Torrenticola pacificensis* are similar to members of the Miniforma group (*T. copipalpa*, *T. manni*, *T. miniforma*, *T. rockyensis*, *T. oliveri*, and *T. pinocchio*) in having short, stocky pedipalps (except *T. oliveri* and *T. pinocchio*); similar pedipalpal extensions (unique to members of this group); and being among the smallest *Torrenticola* in the west (dorsum 500–625 long) (except *T. oliveri*). *T. pacificensis* are best differentiated from *T. rockyensis* by females having a stockier rostrum (length/width ♀ = 2.59–2.68 in *T. pacificensis*, 2.72–2.91 in *T. rockyensis*); and by being distributed in the Pacific Coast Ranges of Washington and Oregon instead of the Rocky Mountains (Idaho & Montana). *T. pacificensis* are best differentiated from *T. copipalpa* by having tuberculate pedipalp femoral extensions (broad and flat in *T. copipalpa*) and by being distributed in the Pacific Coast Ranges of Washington and Oregon, whereas although *T. pacificensis* overlap, they are more southern (southwest Oregon & California). *T. pacificensis* are best differentiated from *T. manni* by having stockier tibiae (length/width = 2.67–3.00 in *pacificensis*, 3.13–3.38 in *T. manni*); having a stockier rostrum (length/width ♀ = 2.59–2.68 in *T. pacificensis*, 3.00–3.13 in *T. manni*; ♂ = 2.76–3.07 in *T. pacificensis*, 3.13–3.20 in *T. manni*); and by being distributed in the Pacific Coast Ranges of Washington and Oregon, whereas *T. manni* is only known from Catron County, New Mexico. *T. pacificensis* are best differentiated from *T. miniforma* by having stockier anterio-medial platelets (length/width = 2.00–2.16 in *T. pacificensis*, 2.44–2.65 in *T. miniforma*); stockier subcapitular rostra (length/width = 2.59–3.07 in *T. pacificensis*; 3.13–3.19 in *T. miniforma*); and by being distributed in the Pacific Coast Ranges of Washington and Oregon, whereas *T. miniforma* is only known from Humboldt County, California. *T. pacificensis* can be differentiated from *T. oliveri* by having a shorter anterior venter (177–235 in *T. pacificensis*, 250–310 in *T. oliveri*) and less elongate pedipalpal tibiae (length/width = 2.6–3.0 in *T. pacificensis*, 3.6–4.2 in *T. oliveri*). *T. pacificensis* can be differentiated from *T. pinocchio* by having a less elongate rostrum (length/width = 2.5–3.1 in *T. pacificensis*, 4.5–4.9 in *T. pinocchio*) and less elongate pedipalpal tibiae (length/width = 2.67–3.00 in *T. pacificensis*, 3.13–3.50 in *T. pinocchio*).

######## Description.

**Female (Figure 182)** (n = 6) (holotype measurements in parentheses when available) with characters of the genus with following specifications.

**Dorsum** — (530–625 (580) long; 335–430 (400) wide) ovoid with purple coloration restricted posteriorly. Anterio-medial platelets (100–117.5 (110) long; 47.5–55 (52.5) wide). Anterio-lateral platelets (145–170 (162.5) long; 52.5–63.75 (57.5) wide) free from dorsal plate. Dgl-4 much closer to the edge of dorsum than to the muscle scars (distance between Dgl-4 270–345 (300)). Dorsal plate proportions: dorsum length/width 1.42–1.58 (1.45); dorsal width/distance between Dgl-4 1.24–1.36 (1.33); anterio-medial platelet length/width 2.05–2.14 (2.10); anterio-lateral platelet length/width 2.63–2.95 (2.83); anterio-lateral/anterio-medial length 1.43–1.48 (1.48).

**Gnathosoma — Subcapitulum** (287.5–325 (300) long (ventral); 210–236 (225) long (dorsal); 110–125 (115) tall) colorless. Rostrum (110–127.5 (120) long; 41.25–47.5 (45) wide). Chelicerae (265–331 (305) long) with curved fangs (50–62 (52.5) long). Subcapitular proportions: ventral length/height 2.52–2.65 (2.61); rostrum length/width 2.59–2.68 (2.67). **Pedipalps** short and stocky (especially tibiae) with nearly tuberculate, dentate ventral extensions on femora and dentate, flanged ventral extensions on genua. Palpomeres: trochanter (30–35 (32.5) long); femur (85–95 (95) long); genu (57.5–67.5 (65) long); tibia (55–67.5 (62.5) long; 20–23.75 (22.5) wide); tarsus (12.5–17.5 (15) long). Palpomere proportions: femur/genu 1.40–1.48 (1.46); tibia/femur 0.65–0.71 (0.66); tibia length/width 2.71–3.00 (2.78).

**Venter** — (650–770 (720) long; 434–521 (440) wide) colorless or with faint purple coloration. Gnathosomal bay (122.5–142.5 (140) long; 66.25–87.5 (67.5) wide). Cxgl-4 subapical. **Medial suture** (47.5–55 (50) long). **Genital plates** (162.5–185 (180) long; 143.75–172.5 (160) wide). Additional measurements: Cx-1 (230–289 (250) long (total); 110–142 (120) long (medial)); Cx-3 (280–331 (280) wide); anterior venter (177.5–205 (182.5) long). Ventral proportions: gnathosomal bay length/width 1.49–2.07 (2.07); anterior venter/genital field length 1.01–1.11 (1.01); anterior venter length/genital field width 1.07–1.23 (1.14); anterior venter/medial suture 3.38–3.89 (3.65).

**Male (Figure 183)** (n = 5) (allotypic measurements in parentheses when available) with characters of the genus with following specifications.

**Dorsum** — (525–590 (550) long; 355–375 (370) wide) ovoid with purple coloration restricted posteriorly. Anterio-medial platelets (97.5–115 (102.5) long; 47.5–55 (47.5) wide). Anterio-lateral platelets (147.5–162.5 (147.5) long; 52.5–57.5 52.5) wide) free from dorsal plate. Dgl-4 much closer to the edge of dorsum than to the muscle scars (distance between Dgl-4 280–290 (290)). Dorsal plate proportions: dorsum length/width 1.48–1.57 (1.49); dorsal width/distance between Dgl-4 1.28–1.30 (1.28); anterio-medial platelet length/width 2.00–2.16 (2.16); anterio-lateral platelet length/width 2.73–2.83 (2.81); anterio-lateral/anterio-medial length 1.41–1.54 (1.44).

**Gnathosoma — Subcapitulum** (275–287.5 (285) long (ventral); 195–220 (212) long (dorsal); 103.75–115 (107.5) tall) colorless. Rostrum (107.5–117.5 (113.75) long; 35–42.5 (38.75) wide). Chelicerae (251–281 (273) long) with curved fangs (46–57 (47) long). Subcapitular proportions: ventral length/height 2.50–2.65 (2.65); rostrum length/width 2.76–3.07 (2.94). **Pedipalps** short and stocky (especially tibiae) with nearly tuberculate, dentate ventral extensions on femora and dentate, flanged ventral extensions on genua. Palpomeres: trochanter (30–32.5 (32.5) long); femur (82.5–90 (85) long); genu (57.5–65 (62.5) long); tibia (55–60 (57.5) long; 20–22.5 (21.25) wide); tarsus (15–16.25 (16.25) long). Palpomere proportions: femur/genu 1.36–1.50 (1.36); tibia/femur 0.65–0.68 (0.68); tibia length/width 2.67–2.94 (2.71).

**Venter** — (645–740 (740) long; 388–460 (431) wide) colorless or with faint purple coloration. Gnathosomal bay (120–140 (135) long; 60–75 (60) wide). Cxgl-4 subapical. **Medial suture** (82.5–87.5 (82.5) long). **Genital plates** (140–147.5 (140) long; 110–120 (113.75) wide). Additional measurements: Cx-1 (243–277 (277) long (total); 120–146 (125) long (medial)); Cx-3 (245–310 (300) wide); anterior venter (216.25–235 (220) long). Ventral proportions: gnathosomal bay length/width 1.60–2.25 (2.25); anterior venter/genital field length 1.50–1.59 (1.57); anterior venter length/genital field width 1.89–1.98 (1.93); anterior venter/medial suture 2.49–2.69 (2.67).

**Immatures** unknown.

######## Etymology.

Specific epithet (*pacificensis*) refers to the distribution of this species in the Pacific Ranges of the Pacific Northwest. This location-based name reflects that locality is the best way to differentiate this species from others in the Miniforma Group, particularly *T. rockyensis*.

######## Distribution.

**Figure 181.**
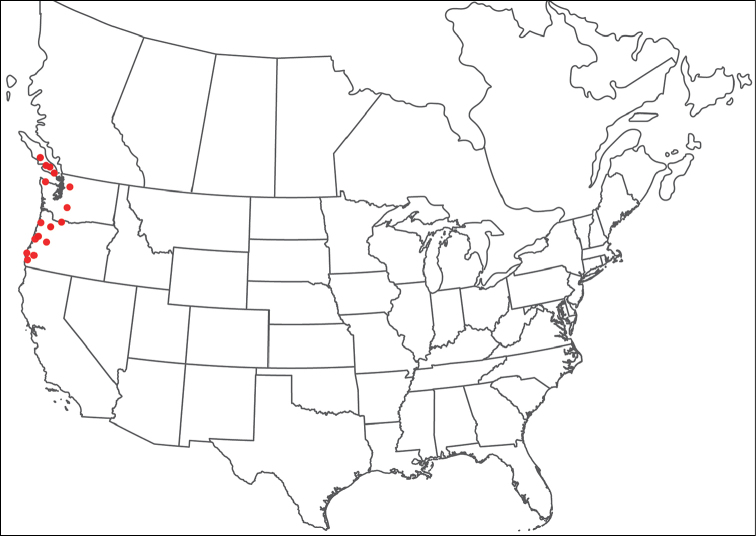
*Torrenticola pacificensis* sp. n. distribution.

Western Oregon and western Washington (Figure 181). It seems reasonable to suspect *T. pacificensis* also occurs in coastal British Columbia and northwestern California.

**Figure 182.**
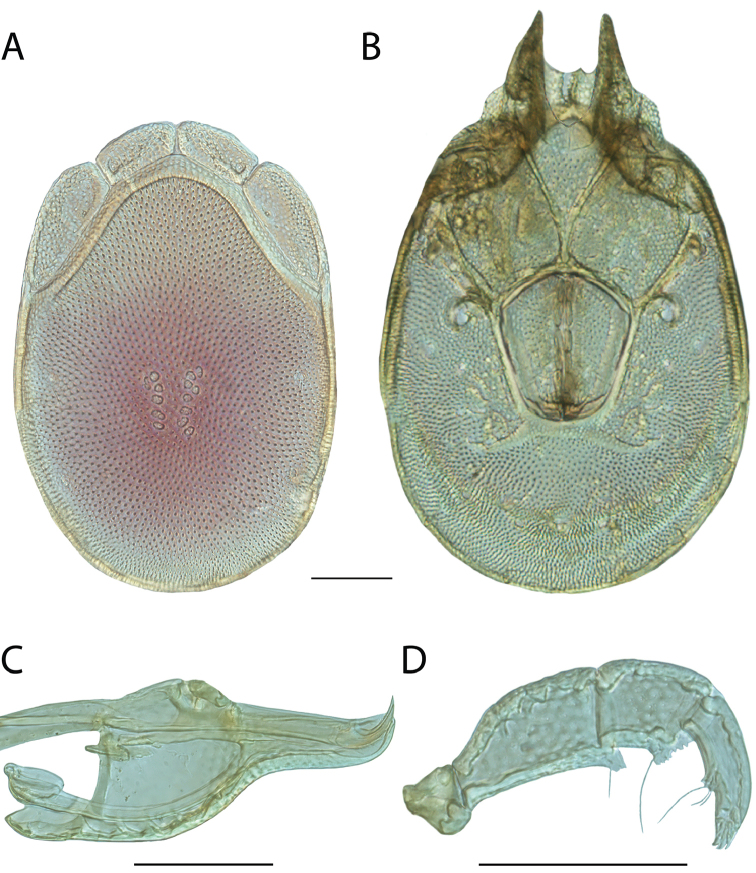
*Torrenticola pacificensis* sp. n. female: **A** dorsal plates **B** venter (legs removed) **C** subcapitulum **D** pedipalp (setae not accurately depicted). Scale = 100 µm.

**Figure 183.**
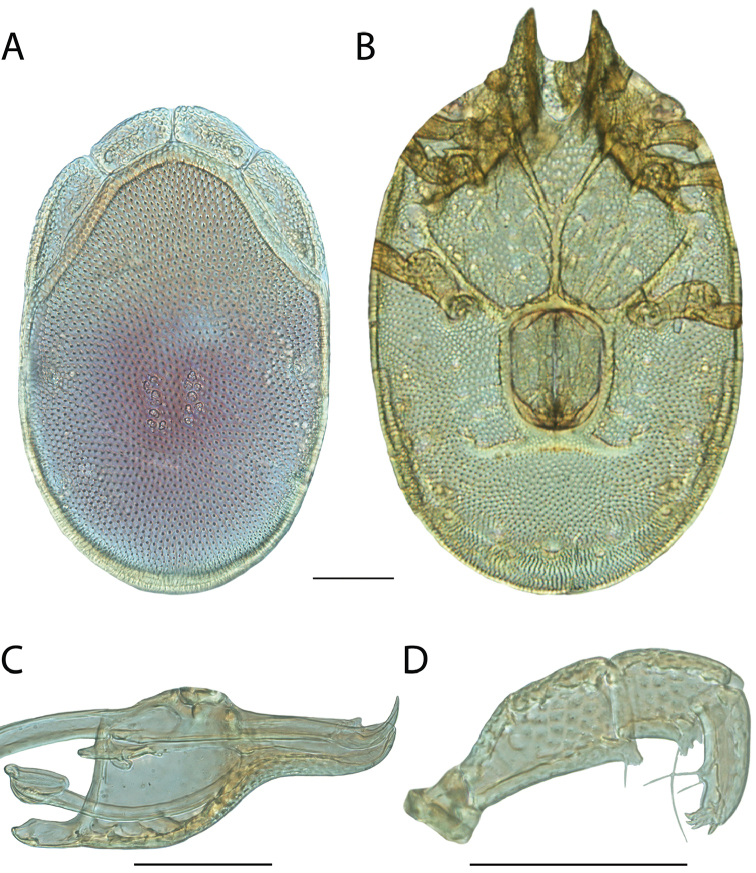
*Torrenticola pacificensis* sp. n. male: **A** dorsal plates **B** venter (legs removed) **C** subcapitulum **D** pedipalp (setae not accurately depicted). Scale = 100 µm.

######## Remarks.

*Torrenticola pacificensis* groups with other members of the Miniforma Complex with high support and specimens of this species are less than 1% different in COI sequence from each other. In all analyses, *T. pacificensis* groups with three other morphologically similar species: *T. rockyensis*, *T. manni*, and *T. copipalpa*. These three species are greater than 4% different from each other in COI sequence. This species overlaps with *T. copipalpa* in west-central Oregon, but is the only member of the Miniforma group that occurs north of that point of overlap.

Based upon overall similarity, the pedipalp genu extensions, and western distribution, we were able to place this species in the Miniforma Identification Group.

This species hypothesis is supported by biogeography, low COI divergence within the species (0–2%) and high divergence between species (3–15%), and by the morphological characters outlined in the diagnosis.

####### Torrenticola pearsoni

Taxon classificationAnimaliaTrombidiformesTorrenticolidae

Fisher & Dowling sp. n.

http://zoobank.org/F77CFD97-1A19-4B8F-B315-0786EE5F3892

######## Material examined.

HOLOTYPE (♀): from USA, Arkansas, Montgomery County, Ouachita National Forest, South Fork Ouachita River, 29 Jul 2011, by AJ Radwell, & B Crump, AJR110302.

PARATYPES (7 ♀; 10 ♂): **Arkansas, USA**: 2 ♀ and 3 ♂ from Montgomery County, Ouachita National Forest, South Fork Ouachita River, 29 Jul 2011, by AJ Radwell, & B Crump, AJR110302 • 1 ♀ from Newton County, Buffalo National River, Mill Creek (36°3'42.12"N, 93°8'7.62"W), 30 May 2012, by TD Edwards, TDE 12-0530-004 • 1 ♀ from Newton County, Buffalo National River, Mill Creek (36°3'42.12"N, 93°8'7.62"W), 20 Jun 2012, by TD Edwards, TDE 12-0620-004 • 1 ♀ from Newton County, Ozark-St Francis National Forest, Little Buffalo River, 11 Jul 2012, by TD Edwards, TDE 12-0711-004 • **Missouri, USA**: 1 ♂ (ALLOTYPE) from Crawford County, Huzzah Creek, Red Bluff campground, east of Davisville, 23 Jul 2011, by IM Smith, IMS110029 • 1 ♂ from Crawford County, Huzzah Creek, Red Bluff campground, east of Davisville, 23 Jul 2011, by IM Smith, IMS110029 • 2 ♂ from McDonald County, Tiff City, beside Route 43, Buffalo Creek (36°40'17"N, 94°36'17"W), 2 May 1996, by IM Smith, IMS960004 • 1 ♀ and 1 ♂ from Oregon County, beside Route 19, north of Greer, Eleven Point (36°48'N, 91°20'W), 28 Jun 1987, by IM Smith, IMS870056 • **Oklahoma, USA**: 1 ♀ and 2 ♂ from Pushmataha County, beside Route 271, south of Albion, Walnut Creek (34°39'N, 95°7'W), 1 Jul 1987, by IM Smith, IMS870063A.

######## Type deposition.

Holotype (♀), allotype (♂), and some paratypes (4 ♀; 5 ♂) deposited in the CNC; other paratypes (3 ♀; 4 ♂) deposited in ACUA.

######## Diagnosis.

*Torrenticola pearsoni* are similar to other members of the Tricolor Complex (*T. bittikoferae*, *T. hoosieri*, *T. larvata*, *T. olliei*, *T. sierrensis*, *T. tricolor*, *T. trimaculata*, *T. unimaculata*, *T. cardia*, *T. kringi*, *T. dimorpha*, and *T. mohawk*) in having a short, conical rostrum. *T. pearsoni* can be differentiated from most other Tricolor Complex (except *T. bittikoferae*, *T. hoosieri* and *T. dimorpha*) by having diffuse pink dorsal coloration, whereas most other members have bold patterning. *T. pearsoni* can be differentiated from *T. hoosieri* by having ventral extensions on the pedipalp femora and genua (lacking in *T. hoosieri*) and having stockier pedipalp tibiae (length/width = 3.0–3.3 in *T. pearsoni*, 3.6–4.4 in *T. hoosieri*). Male *T. pearsoni* can be differentiated from *T. bittikoferae* (males only) by having Dgl-4 closer to the dorsal edge (dorsal width/distance between Dgl-4 = 1.2–1.3 in *T. pearsoni*, 1.6–1.7 in *T. bittikoferae*); more elongate pedipalp tibiae (length/width = 3.1–3.3 in *T. pearsoni*, 2.7–2.8 in *T. bittikoferae*); and a more elongate rostrum (length/width = 2.1–2.4 in *T. pearsoni*, 1.8–1.9 in *T. bittikoferae*). *T. pearsoni* can be differentiated from *T. dimorpha* by having an unmodified dorsal plate (*T. dimorpha* has a dorsal plate medial extension covering nearly half the length of the anterio-medial platelets) and by males having unmodified pedipalps (male *T. dimorpha* have large, highly modified pedipalps which are expanded vertically and laterally).

######## Description.

**Female (Figure 185)** (n = 5) (holotype measurements in parentheses when available) with characters of the genus with following specifications.

**Dorsum** — (620–730 (620) long; 455–500 (455) wide) ellipsoid with faint pink coloration without a distinct pattern. Anterio-medial platelets (123.75–142.5 (123.75) long; 62.5–70 (62.5) wide). Anterio-lateral platelets (167.5–195 (167.5) long; 70–80 (72.5) wide) free from dorsal plate. Dgl-4 much closer to the edge of the dorsum than to the muscle scars (distance between Dgl-4 365–395 (365)). Dorsal plate proportions: dorsum length/width 1.36–1.47 (1.36); dorsal width/distance between Dgl-4 1.21–1.30 (1.25); anterio-medial platelet length/width 1.93–2.04 (1.98); anterio-lateral platelet length/width 2.31–2.64 (2.31); anterio-lateral/anterio-medial length 1.34–1.42 (1.35).

**Gnathosoma — Subcapitulum** (247.5–290 (247.5) long (ventral); 170–210 (171) long (dorsal); 120–142.5 (120) tall) colorless. Rostrum (92.5–112.5 (92.5) long; 45–51.25 (45) wide) short and conical. Chelicerae (220–274 (221) long) with curved fangs (53–75 (64) long). Subcapitular proportions: ventral length/height 2.04–2.12 (2.06); rostrum length/width 2.06–2.25 (2.06). **Pedipalps** with tuberculate ventral extensions on femora and flat, flanged extensions on genua. Palpomeres: trochanter (37.5–50 (37.5) long); femur (96.25–120 (96.25) long); genu (57.5–75 (57.5) long); tibia (75–90 (75) long; 23.75–27.5 (23.75) wide); tarsus (20–25 (20) long). Palpomere proportions: femur/genu 1.60–1.69 (1.67); tibia/femur 0.70–0.80 (0.78); tibia length/width 3.00–3.27 (3.16).

**Venter** — (742–802.75 (743) long; 505–610 (506) wide) colorless. Gnathosomal bay (117.5–145 (132.5) long; 80–100 (80) wide). Cxgl-4 subapical. **Medial suture** (27.5–42.5 (30) long). **Genital plates** (180–187.5 (182.5) long; 147.5–155 (147.5) wide). Additional measurements: Cx-1 (235–282 (260) long (total); 130–138 (137) long (medial)); Cx-3 (335–420 (336) wide); anterior venter (180–200 (187.5) long). Ventral proportions: gnathosomal bay length/width 1.26–1.69 (1.66); anterior venter/genital field length 1.00–1.07 (1.03); anterior venter length/genital field width 1.18–1.33 (1.27); anterior venter/medial suture 4.53–6.55 (6.25).

**Male (Figure 186)** (n = 5) (allotypic measurements in parentheses when available) with characters of the genus with following specifications.

**Dorsum** — (620–675 (620) long; 430–495 (430) wide) ellipsoid with faint pink coloration without a distinct pattern. Anterio-medial platelets (117.5–127.5 (117.5) long; 62.5–75 (62.5) wide). Anterio-lateral platelets (172.5–195 (188.75) long; 67.5–82.5 (70) wide) free from dorsal plate. Dgl-4 much closer to the edge of the dorsum than to the muscle scars (distance between Dgl-4 355–410 (360)). Dorsal plate proportions: dorsum length/width 1.34–1.44 (1.44); dorsal width/distance between Dgl-4 1.19–1.24 (1.19); anterio-medial platelet length/width 1.70–2.04 (1.88); anterio-lateral platelet length/width 2.36–2.70 (2.70); anterio-lateral/anterio-medial length 1.35–1.61 (1.61).

**Gnathosoma — Subcapitulum** (250–260 (260) long (ventral); 170–187 (186) long (dorsal); 112.5–117.5 (112.5) tall) colorless. Rostrum (92.5–102.5 (102.5) long; 42.5–46.25 (42.5) wide) short and conical. Chelicerae (225–244 (239) long) with curved fangs (55–63 (63) long). Subcapitular proportions: ventral length/height 2.20–2.31 (2.31); rostrum length/width 2.11–2.41 (2.41). **Pedipalps** with tuberculate ventral extensions on femora and flat, flanged extensions on genua. Palpomeres: trochanter (40–42.5 (42.5) long); femur (100–106.25 (106.25) long); genu (60–67.5 (67.5) long); tibia (75–85 (75) long; 23.75–26.25 (23.75) wide); tarsus (22.5–22.5 (22.5) long). Palpomere proportions: femur/genu 1.54–1.67 (1.57); tibia/femur 0.71–0.83 (0.71); tibia length/width 3.10–3.24 (3.16).

**Venter** — (720–800 (720) long; 461–588 (461) wide) colorless. Gnathosomal bay (110–130 (125) long; 70–87.5 (70) wide). Cxgl-4 subapical. **Medial suture** (107.5–135 (107.5) long). **Genital plates** (140–155 (152.5) long; 95–105 (95) wide). Additional measurements: Cx-1 (276–302 (283) long (total); 140–167 (140) long (medial)); Cx-3 (334–414 (334) wide); anterior venter (285–335 (285) long). Ventral proportions: gnathosomal bay length/width 1.26–1.79 (1.79); anterior venter/genital field length 1.87–2.18 (1.87); anterior venter length/genital field width 2.93–3.19 (3.00); anterior venter/medial suture 2.30–2.85 (2.65).

**Immatures** unknown.

######## Etymology.

Specific epithet (*pearsoni*) named in honor of Pearson Dowling, son of APGD, for his unquenchable curiosity for all things big and small.

######## Distribution.

**Figure 184.**
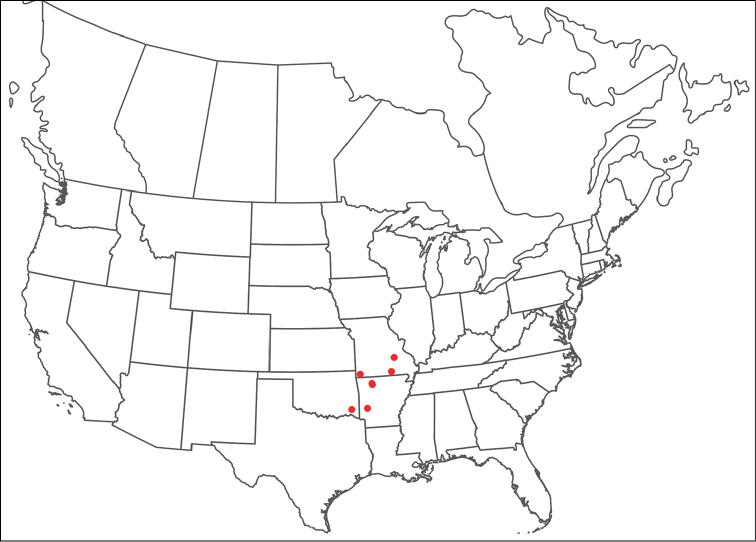
*Torrenticola pearsoni* sp. n. distribution.

Interior Highlands of Missouri and Arkansas (Figure 184).

**Figure 185.**
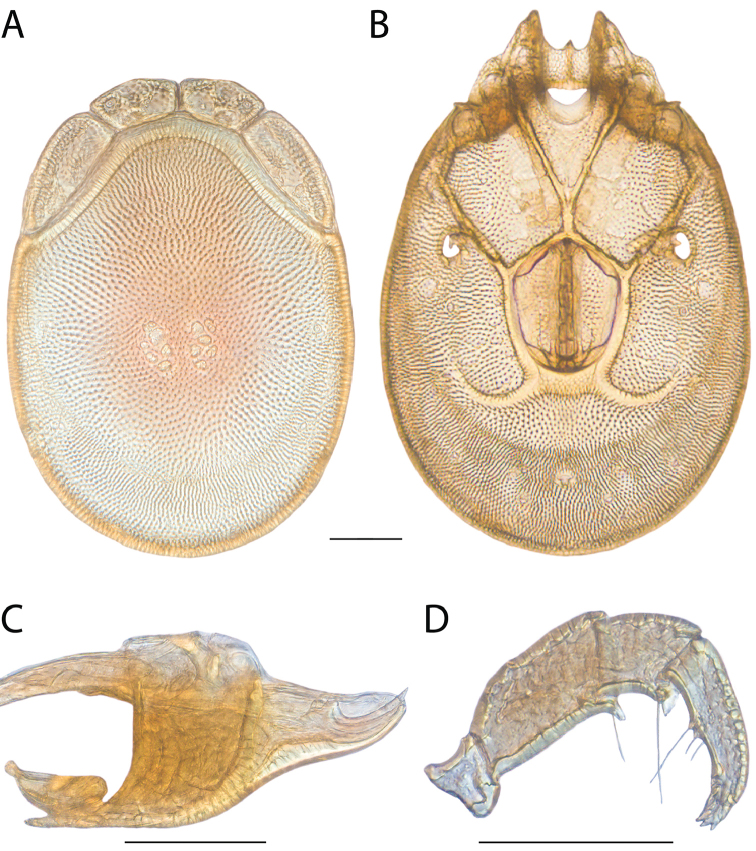
*Torrenticola pearsoni* sp. n. female: **A** dorsal plates **B** venter (legs removed) **C** subcapitulum **D** pedipalp (setae not accurately depicted). Scale = 100 µm.

**Figure 186.**
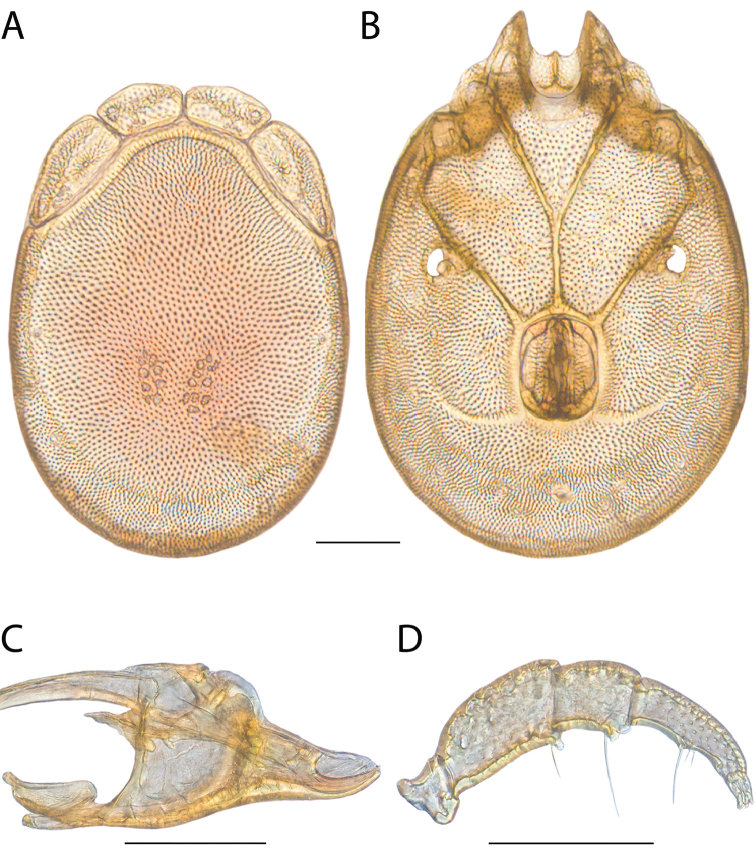
*Torrenticola pearsoni* sp. n. male: **A** dorsal plates **B** venter (legs removed) **C** subcapitulum **D** pedipalp (setae not accurately depicted). Scale = 100 µm.

######## Remarks.

*Torrenticola pearsoni* groups with other members of the Tricolor Complex in all analyses with high support and specimens are less than 2% different in COI sequence from each other. In the combined analysis, *T. pearsoni* groups with two other species (*T. hoosieri* and *T. projector*) with high support and these species are greater than 11% different from each other. Whereas most eastern members of the Tricolor Complex have distinctive patterns, this clade of three species contains members that lack dark patterns.

This species hypothesis is supported by low COI divergence within the species (0–2%), high divergence between species (3–15%), and the morphological characters outlined in the diagnosis.

####### Torrenticola pendula

Taxon classificationAnimaliaTrombidiformesTorrenticolidae

Fisher & Dowling sp. n.

http://zoobank.org/C4DF2AEE-F6DC-4DD7-84A1-53A392532968

######## Material examined.

HOLOTYPE (♀): from USA, Maine, Washington County, Old Stream, off Route 9, 5.5 km west of Route 192 at Wesley, 6 June 2012, by IM Smith, IMS120012.

PARATYPES (1 ♀; 1 ♂): **Maine, USA**: 1 ♂ (ALLOTYPE) from Washington County, Old Stream, off Route 9, 5.5 km west of Route 192 at Wesley, 6 June 2012, by IM Smith, IMS120012 • 1 ♀ from Washington County, Old Stream, off Route 9, 5.5 km west of Route 192 at Wesley, 6 June 2012, by IM Smith, IMS120012.

######## Type deposition.

Holotype (♀), allotype (♂) deposited in the CNC; paratype (1 ♀) deposited in the ACUA.

######## Diagnosis.

*Torrenticola pendula* are similar to other members of the Rusetria “Eastern 2-Plates” group (*T. biscutella*, *T. caerulea*, *T. delicatexa*, *T. indistincta*, *T. malarkeyorum*, *T. sellersorum*, *T. tysoni*, *T. ululata*, *T. whitneyae*, *T. microbiscutella*, *T. feminellai*) in having anterio-lateral platelets fused to the dorsal plate, having dorsal coloration separated into anterior and posterior portions (except *T. ululata* and *T. indistincta*), and being distributed in the east. It is one of only four Eastern 2-Plates that have dark, bold, bluish-purple coloration (also *T. tysoni*, *T. biscutella*, and *T. sellersorum*). *T. pendula* can be differentiated from other Eastern 2-Plates (except *T. whitneyae* and *T. feminellai*) by having a more elongate gnathosomal bay (2.42–2.9 in *T. pendula*, 1.4–2.24 in others) and often by having a dorsal pattern of dark bluish-purple separated into anterior and posterior portions connected medially. In the Rusetria Complex only *T. whitneyae* and *T. magnexa* (rarely) has a similar pattern. *T. pendula* can be differentiated from *T. magnexa* by having anterio-medial platelets fully fused to the dorsal plate (anterio-lateral platelets partially fused to the dorsal plate in *T. magnexa*). *T. pendula* can be differentiated from *T. whitneyae* by having a more elongate rostrum (length/width = 2.87–3.06 in *T. pendula*, 2.41–2.69 in *T. whitneyae*). *T. pendula* can be differentiated from *T. feminellai* by having less elongate pedipalpal tibiae (length/width = 2.78-3.05 in *T. pendula*; 3.63–4.11 in *T. feminellai*).

######## Description.

**Female (Figure 188)** (n = 2) (holotype measurements in parentheses when available) with characters of the genus with following specifications.

**Dorsum** — (630–650 (630) long; 490–490 (490) wide) ovoid with bold purple coloration both anteriorly and posteriorly connected medially. Anterio-medial platelets (145–145 (145) long; 45–47.5 (47.5) wide). Anterio-lateral platelets (170–177.5 (170) long; 62.5–75 (62.5) wide) fused to dorsal plate. Dgl-4 much closer to the edge of the dorsum than to the muscle scars (distance between Dgl-4 350–350 (350)). Dorsal plate proportions: dorsum length/width 1.29–1.33 (1.29); dorsal width/distance between Dgl-4 1.40–1.40 (1.40); anterio-medial platelet length/width 3.05–3.22 (3.05); anterio-lateral platelet length/width 2.37–2.72 (2.72); anterio-lateral/anterio-medial length 1.17–1.22 (1.17).

**Gnathosoma — Subcapitulum** (320–335 (335) long (ventral); 242–252 (252) long (dorsal); 147.5–147.5 (147.5) tall) colorless. Rostrum (127.5–137.5 (137.5) long; 42.5–45 (45) wide). Chelicerae (336–347 (347) long) with curved fangs (53–62 (62) long). Subcapitular proportions: ventral length/height 2.17–2.27 (2.27); rostrum length/width 3.00–3.06 (3.06). **Pedipalps** with tuberculate ventral extensions on femora and genua. Palpomeres: trochanter (45–47.5 (47.5) long); femur (102.5–107.5 (102.5) long); genu (67.5–71.25 (67.5) long); tibia (72.5–75 (75) long; 23.75–25 (25) wide); tarsus (17.5–17.5 (17.5) long). Palpomere proportions: femur/genu 1.51–1.52 (1.52); tibia/femur 0.67–0.73 (0.73); tibia length/width 3.00–3.05 (3.00).

**Venter** — (770–800 (770) long; 532–557 (557) wide) with bold purple coloration. Gnathosomal bay (187.5–195 (195) long; 75–77.5 (75) wide). Cxgl-4 subapical. **Medial suture** (10–12.5 (12.5) long). **Genital plates** (180–186.25 (186.25) long; 168.75–172.5 (168.75) wide). Additional measurements: Cx-1 (290–301 (290) long (total); 110–115 (115) long (medial)); Cx-3 (345–350 (346) wide); anterior venter (145–157.5 (145) long). Ventral proportions: gnathosomal bay length/width 2.42–2.60 (2.60); anterior venter/genital field length 0.78–0.88 (0.78); anterior venter length/genital field width 0.86–0.91 (0.86); anterior venter/medial suture 11.60–15.75 (11.60).

**Male (Figure 189)** (n = 1) (allotype only) with characters of the genus with following specifications.

**Dorsum** — (500 long; 380 wide) ovoid with bold purple coloration separated into anterior and posterior portions. Anterio-medial platelets (130 long; 41.25 wide). Anterio-lateral platelets (155 long; 62.5 wide) fused to dorsal plate. Dgl-4 much closer to the edge of the dorsum than to the muscle scars (distance between Dgl-4 275). Dorsal plate proportions: dorsum length/width 1.32; dorsal width/distance between Dgl-4 1.38; anterio-medial platelet length/width 3.15; anterio-lateral platelet length/width 2.48; anterio-lateral/anterio-medial length 1.19.

**Gnathosoma — Subcapitulum** (272.5 long (ventral); 197.5 long (dorsal); 110 tall) colorless. Rostrum (107.5 long; 37.5 wide). Chelicerae (280 long) with curved fangs 50 long). Subcapitular proportions: ventral length/height 2.48; rostrum length/width 2.87. **Pedipalps** with tuberculate ventral extensions on femora and genua. Palpomeres: trochanter (38.75 long); femur (85 long); genu (58.75 long); tibia (62.5 long; 22.5 wide); tarsus (15 long). Palpomere proportions: femur/genu 1.45; tibia/femur 0.74; tibia length/width 2.78.

**Venter** — (620 long; 430 wide) with faint purple coloration. Gnathosomal bay (152.5 long; 52.5 wide). **Medial suture** (72.5 long). Cxgl-4 subapical. **Genital plates** (126.25 long; 120 wide). Additional measurements: Cx-1 (250 long (total); 100 long (medial)); Cx-3 (300 wide); anterior venter (207.5 long). Ventral proportions: gnathosomal bay length/width 2.90; anterior venter/genital field length 1.64; anterior venter length/genital field width 1.73; anterior venter/medial suture 2.86.

**Immatures** unknown.

######## Etymology.

Specific epithet (*pendula*) named for the swinging torture axe described in “The Pit and the Pendulum” by Edgar Allan Poe, most artistic depictions of which resemble the dorsal patterning on this species.

######## Distribution.

**Figure 187.**
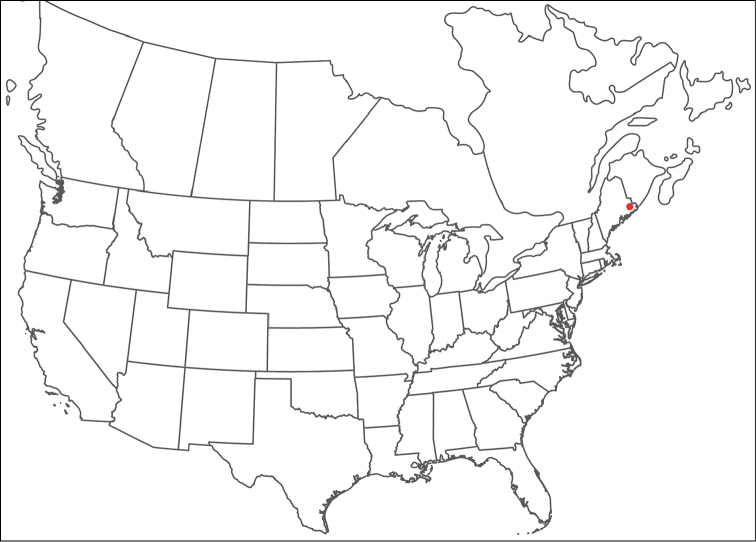
*Torrenticola pendula* sp. n. distribution.

Known only from Washington County, Maine (Figure 187).

**Figure 188.**
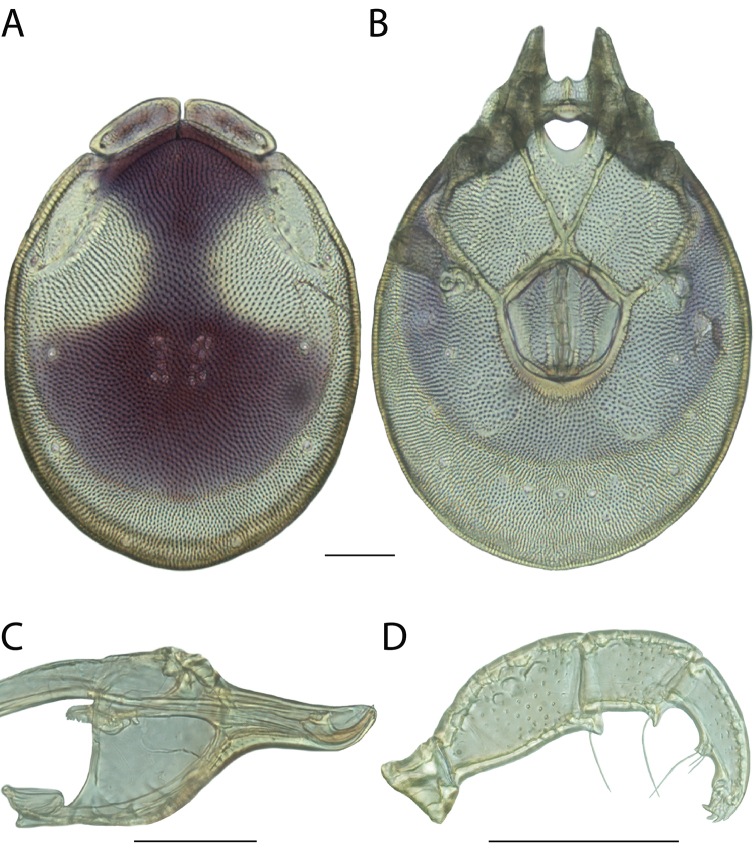
*Torrenticola pendula* sp. n. female: **A** dorsal plates **B** venter (legs removed) **C** subcapitulum **D** pedipalp (setae not accurately depicted). Scale = 100 µm.

**Figure 189.**
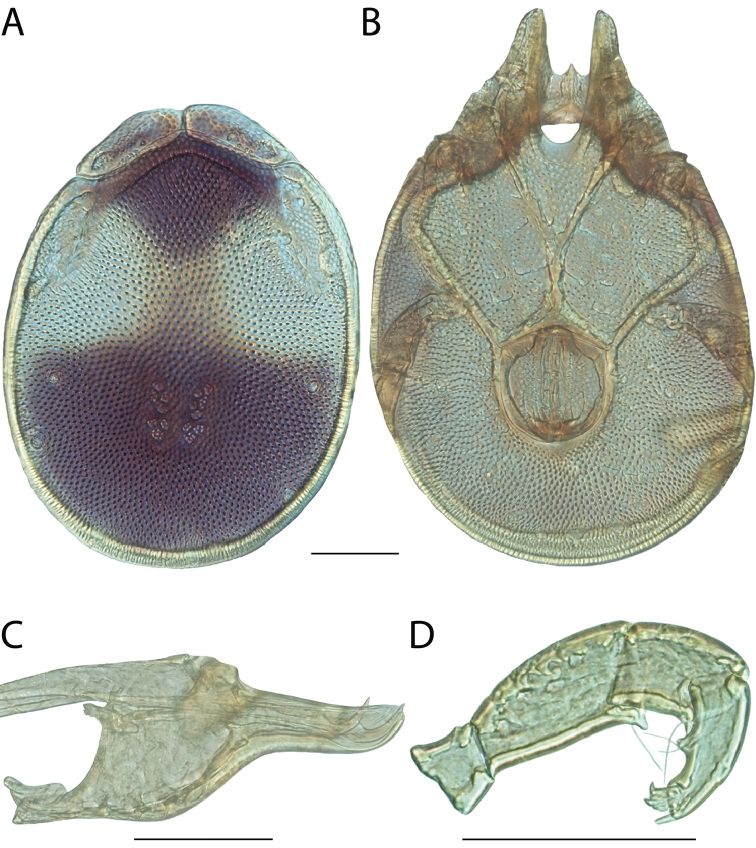
*Torrenticola pendula* sp. n. male: **A** dorsal plates **B** venter (legs removed) **C** subcapitulum **D** pedipalp (setae not accurately depicted). Scale = 100 µm.

######## Remarks.

*Torrenticola pendula* groups with other members of the Rusetria Complex with high support. The two specimens (one male and one female) are 6.7% different in COI sequence from each other, suggesting each represents a separate species. However, in addition to similar overall appearance and occurring in the same sample, they also are indistinguishable in characters that are not usually sexually dimorphic (e.g., pedipalp proportions). Because of this, we consider these two specimens as the same species, despite the high COI variability.

In all analyses, *T. pendula* groups with other eastern members of the Rusetria Complex that have posterio-lateral platelets fused with the dorsal shield, but in both analyses the position of this species within that clade was not well-supported. This species was 17–18% different in COI sequence from sister species.

Based upon overall similarity, fusion of the lateral platelets with the dorsal shield, phylogenetic position, and distribution, we were able to place this species in the Eastern 2-Plate Identification Group.

This species hypothesis is supported by phylogenetic affinity, high divergence between species (17–18%), and by the morphological characters outlined in the diagnosis.

####### Torrenticola pinocchio

Taxon classificationAnimaliaTrombidiformesTorrenticolidae

Fisher & Dowling sp. n.

http://zoobank.org/D8162FB2-4C67-4E20-9FA4-B8991E87B21F

######## Material examined.

HOLOTYPE (♀): from USA, California, Mendocino County, beside Rt. 128, 7.3 km south of Boonville, (38°57'57"N, 123°19'19"W), 4 August 1987, by IM Smith, IMS870126A

PARATYPES (3 ♀; 2 ♂): **California, USA**: 1 ♂ (ALLOTYPE) from Mendocino County, beside Rt. 128, 7.3 km south of Boonville, (38°57'57"N, 123°19'19"W), 4 August 1987, by IM Smith, IMS870126A • 1 ♀ from Trinity County, beside Rt. 36 at Forest Glen Station Campground, (40°23'23"N, 123°20'20"W), 6 August 1987, by IM Smith & JD Smith, IMS870131 • **Oregon, USA**: 1 ♀ and 1 ♂ from Curry County, Port Orford; Butler Bar Campground off Elk River Road, (42°43'43"N, 124°16'16"W), 25 June 1976, by IM Smith, IMS760162 • 1 ♀ from Curry County, Sixes; Edson Creek Campground beside Sixes River Road, (42°48'48"N, 124°24'24"W), 4 July 1983, by IM Smith, IMS830021A

######## Type deposition.

Holotype (♀), allotype (♂), and some paratypes (2 ♀) deposited in the CNC; other paratypes (1 ♀; 1 ♂) deposited in the ACUA.

######## Diagnosis.

*Torrenticola pinocchio* are similar to members of the Miniforma group (*T. copipalpa*, *T. manni*, *T. miniforma*, *T. pacificensis*, *T. rockyensis*, and *T. oliveri*) in having similar pedipalpal extensions (unique to members of this group); and being among the smallest *Torrenticola* in the west (dorsum 500–625 long) (except *T. oliveri*). *T. pinocchio* can be differentiated from all other *Torrenticola* by having a more elongate rostrum (length/width = 4.58–3.91 in *T. pinocchio*, 1.29–4.40 in others).

######## Description.

**Female (Figure 191)** (n = 4) (holotype measurements in parentheses when available) with characters of the genus with following specifications.

**Dorsum** — (580–610 (580) long; 360–400 (370) wide) ellipsoid and colorless. Anterio-medial platelets (95–115 (102.5) long; 47.5–50 (47.5) wide). Anterio-lateral platelets (150–165 (150) long; 55–65 (60) wide) free from dorsal plate. Dgl-4 much closer to the edge of the dorsum than to the muscle scars (distance between Dgl-4 280–310 (295)). Dorsal plate proportions: dorsum length/width 1.53–1.64 (1.57); dorsal width/distance between Dgl-4 1.25–1.30 (1.25); anterio-medial platelet length/width 1.90–2.30 (2.16); anterio-lateral platelet length/width 2.50–2.82 (2.50); anterio-lateral/anterio-medial length 1.43–1.63 (1.46).

**Gnathosoma — Subcapitulum** (300–310 (305) long (ventral); 230–240 (230) long (dorsal); 100–107.5 (107.5) tall) colorless. Rostrum (137.5–145 (140) long; 30–30 (30) wide). Chelicerae (290–305 (300) long) with curved fangs (40–45 (45) long). Subcapitular proportions: ventral length/height 2.84–3.05 (2.84); rostrum length/width 4.58–4.83 (4.67). **Pedipalps** with broad, dentate, and anteriorly-directed ventral extensions on femora and dentate, flanged ventral extensions on genua. Palpomeres: trochanter (30–30 (30) long); femur (97.5–102.5 (97.5) long); genu (67.5–71.25 (67.5) long); tibia (65–70 (70) long; 20–20 (20) wide); tarsus (15–17.5 (17.5) long). Palpomere proportions: femur/genu 1.39–1.45 (1.44); tibia/femur 0.65–0.72 (0.72); tibia length/width 3.25–3.50 (3.50).

**Venter — Subcapitulum** (720–755 (730) long; 400–450 (410) wide) colorless. Gnathosomal bay (140–145 (145) long; 55–60 (60) wide). Cxgl-4 subapical. **Medial suture** (52.5–55 (55) long). **Genital plates** (155–167.5 (160) long; 135–150 (140) wide). Additional measurements: Cx-1 (260–270 (270) long (total); 120–130 (130) long (medial)); Cx-3 (52.5–55 (55) wide); anterior venter (180–195 (192.5) long). Ventral proportions: gnathosomal bay length/width 2.42–2.59 (2.42); anterior venter/genital field length 1.16–1.20 (1.20); anterior venter length/genital field width 1.30–1.41 (1.38); anterior venter/medial suture 3.43–3.71 (3.50).

**Male (Figure 192)** (n = 2) (allotypic measurements in parentheses when available) with characters of the genus with following specifications.

**Dorsum** — (520–540 (520) long; 340–350 (340) wide) ellipsoid and colorless. Anterio-medial platelets (95–102.5 (95) long; 45–47.5 (45) wide). Anterio-lateral platelets (145–155 (145) long; 50–57.5 (50) wide) free from dorsal plate. Dgl-4 much closer to the edge of the dorsum than to the muscle scars (distance between Dgl-4 260–270 (260)). Dorsal plate proportions: dorsum length/width 1.53–1.54 (1.53); dorsal width/distance between Dgl-4 1.30–1.31 (1.31); anterio-medial platelet length/width 2.11–2.16 (2.11); anterio-lateral platelet length/width 2.70–2.90 (2.90); anterio-lateral/anterio-medial length 1.51–1.53 (1.53).

**Gnathosoma — Subcapitulum** (275–290 (275) long (ventral); 215–225 (215) long (dorsal); 90–95 (95) tall) colorless. Rostrum (130–135 (130) long; 27.5–27.5 (27.5) wide). Chelicerae ((265) long) with curved fangs ((45) long). Subcapitular proportions: ventral length/height 2.89–3.22 (2.89); rostrum length/width 4.73–4.91 (4.73). **Pedipalps** with broad, dentate, and anteriorly-directed ventral extensions on femora and dentate, flanged ventral extensions on genua. Palpomeres: trochanter (30–32.5 (30) long); femur (90–96.25 (90) long); genu (67.5–67.5 (67.5) long); tibia (62.5–65 (65) long; 18.75–20 (18.75) wide); tarsus (15–15 (15) long). Palpomere proportions: femur/genu 1.33–1.43 (1.33); tibia/femur 0.65–0.72 (0.72); tibia length/width 3.13–3.47 (3.47).

**Venter** — (650–670 (650) long; 385–410 (385) wide) colorless. Gnathosomal bay (120–125 (110) long; 50–52.5 (50) wide). Cxgl-4 subapical. **Medial suture** (80–85 (80) long). **Genital plates** (117.5–125 (117.5) long; 97.5–100 (97.5) wide). Additional measurements: Cx-1 (240–240 (240) long (total); 110–125 (110) long (medial)); Cx-3 (260–280 (260) wide); anterior venter (210–225 (210) long). Ventral proportions: gnathosomal bay length/width 2.29–2.50 (2.50); anterior venter/genital field length 1.79–1.80 (1.79); anterior venter length/genital field width 2.15–2.25 (2.15); anterior venter/medial suture 2.63–2.65 (2.63).

**Immatures** unknown.

######## Etymology.

Specific epithet (*pinocchio*) refers to the elongate rostrum of this species, which resembles the ever-growing nose of Pinocchio (noun in apposition), a fictional character from the popular Italian children’s novel, *The Adventures of Pinocchio* by Carlo Collodi (1883). Pinocchio is well-known for his nose, which grows every time he tells a lie.

######## Distribution.

**Figure 190.**
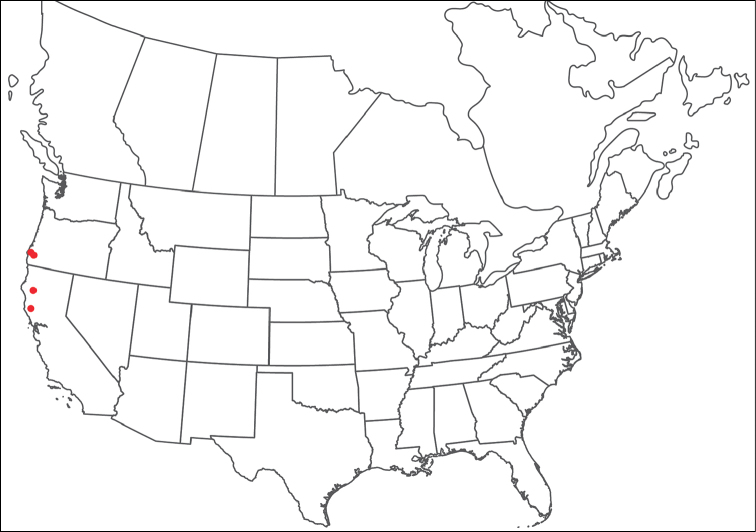
*Torrenticola pinocchio* sp. n. distribution.

California and Oregon (Figure 190).

**Figure 191.**
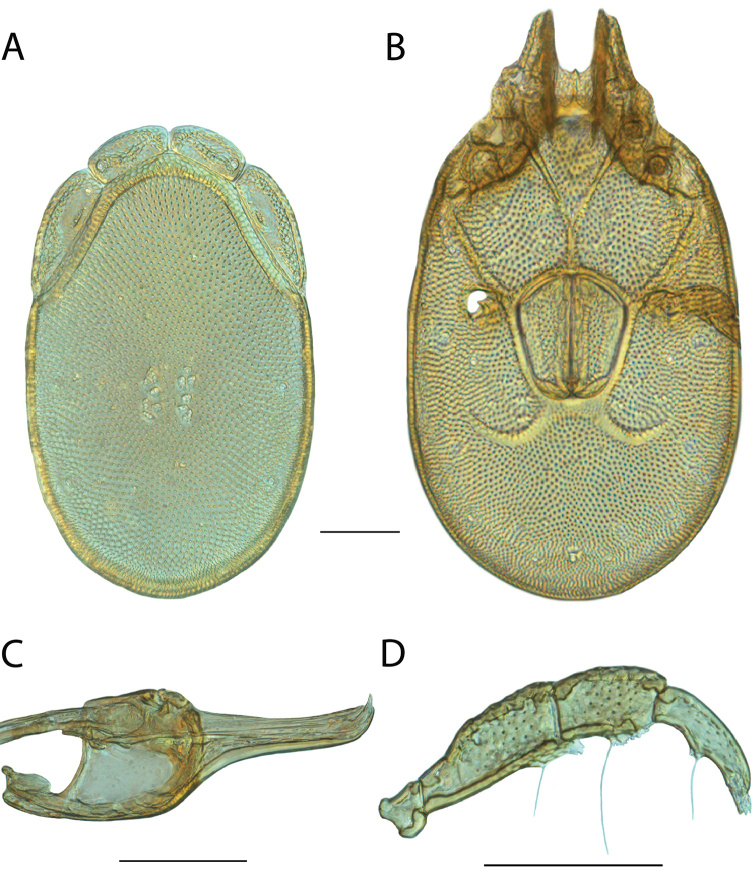
*Torrenticola pinocchio* sp. n. female: **A** dorsal plates **B** venter (legs removed) **C** subcapitulum **D** pedipalp (setae not accurately depicted). Scale = 100 µm.

**Figure 192.**
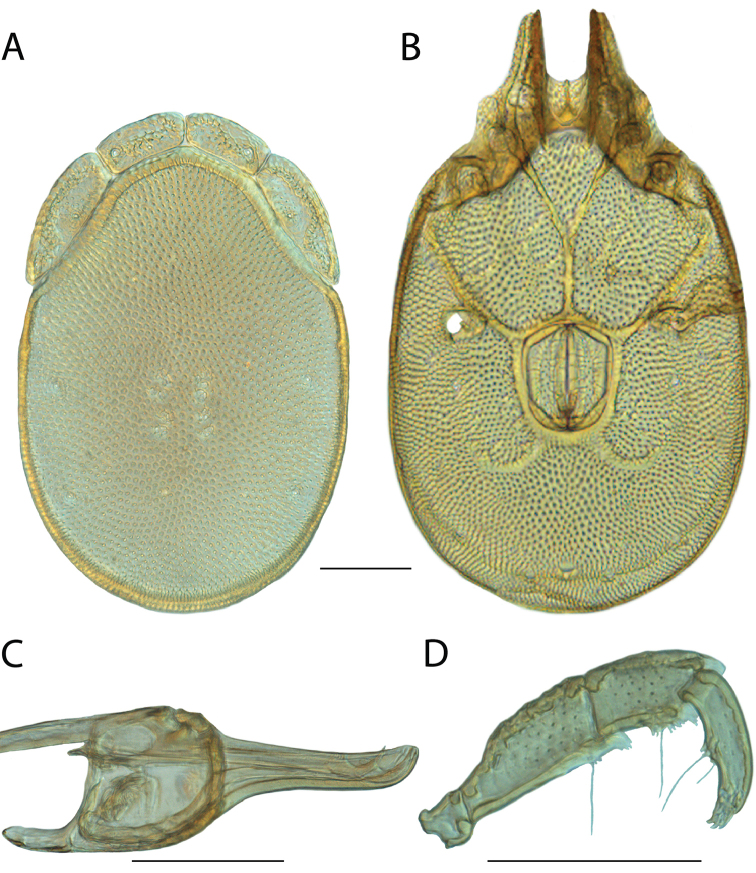
*Torrenticola pinocchio* sp. n. male: **A** dorsal plates **B** venter (legs removed) **C** subcapitulum **D** pedipalp (setae not accurately depicted). Scale = 100 µm.

######## Remarks.

Unfortunately, we were unable to acquire fresh material of *Torrenticola pinocchio* and therefore this species is not included in our phylogenetic analyses. However, we were able to examine morphology with material preserved in GAW. The overall appearance, small size, dentate flange on the pedipalpal genua, and distribution, are consistent with placing this species in the Miniforma Complex and Miniforma Identification Group.

####### Torrenticola pollani

Taxon classificationAnimaliaTrombidiformesTorrenticolidae

Fisher & Dowling sp. n.

http://zoobank.org/4B2CC127-07E7-48DC-921E-2EE6BB6AAE8B

######## Material examined.

HOLOTYPE (♀): from USA, Alabama, Lauderdale County, off Natchez Parkway, 7 km south of Tennessee state line (34°56'31"N, 87°49'41"W), 27 Sep 2010, by IM Smith, IMS100162, DNA 1288.

PARATYPES (4 ♀; 11 ♂): **Alabama, USA**: 2 ♂ from Clay County, beside Forest Route 649, 0.8 kilometers northeast of road from Forest Route 600 to Campbell Springs, Talladega Creek, 2 Jul 1990, by IM Smith, IMS900075A • 1 ♂ from Cleburne County, beside Route 431, 3.3 kilometers southeast of Calhoun, Jackson Creek (33°36'N, 85°42'W), 2 Jul 1990, by IM Smith, IMS900074 • 1 ♂ (ALLOTYPE) from Lauderdale County, off Natchez Parkway, 7 km south of Tennessee state line (34°56'31"N, 87°49'41"W), 27 Sep 2010, by IM Smith, IMS100162 • 1 ♀ and 1 ♂ from Lauderdale County, off Natchez Parkway, 7 km south of Tennessee state line (34°56'31"N, 87°49'41"W), 24 Sep 2009, by IM Smith, IMS090121 • 3 ♀ and 2 ♂ from Lauderdale County, off Natchez Parkway, 7 km south of Tennessee state line (34°56'31"N, 87°49'41"W), 27 Sep 2010, by IM Smith, IMS100162 • **Georgia, USA**: 1 ♂ from Floyd County, beside road from Everett Springs to Villanow, 1.8 kilometers south of The Pocket Campground, Johns Creek, 4 Jul 1990, by IM Smith, IMS900076 • 1 ♂ from Floyd County, beside road from Everett Springs to Villanow, 1.4 kilometers south of The Pocket Campground, tributary of Johns Creek, 4 Jul 1990, by IM Smith, IMS900076 • 2 ♂ from Floyd County, The Pocket Campground, between Everett Springs and Villanow, tributary of Johns Creek, 4 Jul 1990, by IM Smith, IMS900073A • 1 ♂ from White County, Helen, beside Road to Anna Ruby Falls, Smith Creek (34°44'N, 83°43'W), 24 Sep 1992, by IM Smith, IMS920051.

######## Type deposition.

Holotype (♀), allotype (♂), and other paratypes (2 ♀; 5 ♂) deposited in the CNC; other paratypes (2 ♀; 5 ♂) deposited in ACUA.

######## Diagnosis.

*Torrenticola pollani* are similar to other members of the Rusetria “4-Plates” group (*T. dunni*, *T. glomerabilis*, *T. kittatinniana*, *T. rufoalba*, and *T. shubini*) and *T. skvarlai* in having anterio-lateral platelets free from the dorsal plate, dorsal coloration separated into anterior and posterior portions, and indistinct hind coxal margins. *T. pollani* can be differentiated from *T. dunni* by having a smaller dorsum (length ♀ = 535–560 in *T. pollani*, 605–680 in *T. dunni*; ♂ = 440–490 in *T. pollani*, 500–540 in *T. dunni*; width ♀ , 410–420 in *T. pollani*, 440–490 in *T. dunni*; ♂ = 350–370 in *A38*, 310–340 in *T. pollani*); and a more elongate rostrum (length/width = 3.3–3.8 in *T. pollani*, 2.8–3.1 in *T. dunni*). *T. pollani* can be differentiated from *T. shubini* by having more elongate tibiae (length/width ♀ = 4.00–4.18 in *T. pollani*, 3.35–3.60 in *T. shubini*; ♂ = 3.44–3.75 in *T. pollani*, 3.11–3.22 in *T. shubini*) and a more elongate rostrum (length/width = 3.27–3.82 in *T. pollani*, 2.24–2.85 in *T. shubini*). *T. pollani* can be differentiated from *T. glomerabilis* by having more elongate anterio-medial platelets (length/width ♀ = 2.5–3.0 in *T. pollani*, 1.9–2.3 in *T. glomerabilis*; ♂ = 2.3–2.5 in *T. pollani*, 1.9–2.2 in *T. glomerabilis*) and thinner dorsum (♀ = 410–420 in *T. pollani*, 460–490 in *T. glomerabilis*; ♂ = 310–340 in *T. pollani*, 395–430 in *T. glomerabilis*). *T. pollani* can be differentiated from *T. kittatinniana* by having a more elongate rostrum (length/width = 3.27–3.82 in *T. pollani*, 2.71–3.16 in *T. kittatinniana*) and more elongate tibiae (length/width ♀ = 3.8–4.2 in *T. pollani*, 3.3 in *T. kittatinniana*; ♂ = 3.44–3.75 in *T. pollani*, 2.80 in *T. kittatinniana*). *T. pollani* can be differentiated from *T. rufoalba* by having a more elongate rostrum (length/width = 3.27–3.82 in *T. pollani*, 2.96–3.06 in *T. rufoalba*). Female *T. pollani* can be differentiated from female *T. rufoalba* by having more elongate tibiae (length/width = 3.8–4.2 in *T. pollani*, 3.5 in *T. rufoalba*). Male *T. pollani* can be differentiated from male *T. rufoalba* by having a longer anterior venter (235–250 in *T. pollani*, 195 in *T. rufoalba*). *T. pollani* can be differentiated from *T. skvarlai* by having a conical pedipalpal femoral tubercle, whereas *T. skvarlai* has a broad and flat pedipalpal femoral tubercle, and by having a longer anterior venter (♀ = 155–163 in *T. pollani*, 140–153 in *T. skvarlai*; ♂ = 235–250 in *T. pollani*, 177.5–205 in *T. skvarlai*).

######## Description.

**Female (Figure 194)** (n = 5) (holotype measurements in parentheses when available) with characters of the genus with following specifications.

**Dorsum** — (535–560 (550) long; 410–420 (415) wide) ovoid with purple to bluish-purple coloration separated into anterior and posterior portions, and occasionally with faint strip of orange medially. Anterio-medial platelets (105–125 (105) long; 40–47.5 (40) wide). Anterio-lateral platelets (152.5–170 (152.5) long; 52.5–62.5 (52.5) wide) free from dorsal plate. Dgl-4 much closer to the edge of the dorsum than to the muscle scars (distance between Dgl-4 270–290 (270)). Dorsal plate proportions: dorsum length/width 1.29–1.37 (1.33); dorsal width/distance between Dgl-4 1.45–1.54 (1.54); anterio-medial platelet length/width 2.50–3.03 (2.63); anterio-lateral platelet length/width 2.64–2.93 (2.90); anterio-lateral/anterio-medial length 1.34–1.47 (1.45).

**Gnathosoma — Subcapitulum** (310–332.5 (310) long (ventral); 236–257.5 (237) long (dorsal); 122.5–132.5 (122.5) tall) colorless. Rostrum (133.75–150 (133.75) long; 35–41.25 (35) wide). Chelicerae (310–340 (315) long) with curved fangs (53–60 (56) long). Subcapitular proportions: ventral length/height 2.43–2.61 (2.53); rostrum length/width 3.27–3.82 (3.82). **Pedipalps** with tuberculate ventral extensions on femora and genua. Palpomeres: trochanter (37.5–42.5 (40) long); femur (115–127.5 (117.5) long); genu (65–70 (65) long); tibia (80–92.5 (80) long; 20–22.5 (20) wide); tarsus (17.5–20 (17.5) long). Palpomere proportions: femur/genu 1.70–1.92 (1.81); tibia/femur 0.68–0.78 (0.68); tibia length/width 3.89–4.18 (4.00).

**Venter** — (610–675 (675) long; 461–490 (489) wide) colorless. Gnathosomal bay (142.5–170 (142.5) long; 77.5–97.5 (87.5) wide). Cxgl-4 subapical. **Medial suture** (12.5–20 (17.5) long). **Genital plates** (157.5–175 (157.5) long; 137.5–152.5 (137.5) wide). Additional measurements: Cx-1 (258–290 (259) long (total); 89–121 (96) long (medial)); Cx-3 (304–364 (310) wide); anterior venter (155–162.5 (157.5) long). Ventral proportions: gnathosomal bay length/width 1.63–2.13 (1.63); anterior venter/genital field length 0.89–1.02 (1.00); anterior venter length/genital field width 1.02–1.15 (1.15); anterior venter/medial suture 7.75–12.70 (9.00).

**Male (Figure 195)** (n = 5) (allotypic measurements in parentheses when available) with characters of the genus with following specifications.

**Dorsum** — (440–490 (450) long; 310–340 (315) wide) ovoid with purple to bluish-purple coloration separated into anterior and posterior portions, and occasionally with faint strip of orange medially. Anterio-medial platelets (92.5–102.5 (100) long; 40–42.5 (42.5) wide). Anterio-lateral platelets (140–155 (142.5) long; 42.5–50 (45) wide) free from dorsal plate. Dgl-4 much closer to the edge of the dorsum than to the muscle scars (distance between Dgl-4 210–250 (215)). Dorsal plate proportions: dorsum length/width 1.33–1.44 (1.43); dorsal width/distance between Dgl-4 1.32–1.48 (1.47); anterio-medial platelet length/width 2.31–2.44 (2.35); anterio-lateral platelet length/width 3.10–3.29 (3.17); anterio-lateral/anterio-medial length 1.43–1.59 (1.43).

**Gnathosoma — Subcapitulum** (265–285 (265) long (ventral); 202–208 (203) long (dorsal); 87.5–100 (97.5) tall) colorless. Rostrum (111.25–122.5 (111.25) long; 32.5–35 (32.5) wide). Chelicerae (257–280 (263) long) with curved fangs (45–50 (47) long). Subcapitular proportions: ventral length/height 2.72–3.06 (2.72); rostrum length/width 3.41–3.54 (3.42). **Pedipalps** with tuberculate ventral extensions on femora and genua. Palpomeres: trochanter (35–40 (35) long); femur (100–103.75 (101.25) long); genu (60–62.5 (60) long); tibia (72.5–80 (75) long; 20–22.5 (20) wide); tarsus (15–17.5 (17.5) long). Palpomere proportions: femur/genu 1.64–1.73 (1.69); tibia/femur 0.70–0.78 (0.74); tibia length/width 3.41–3.75 (3.75).

**Venter** — (540–600 (555) long; 358–408 (359) wide) colorless. Gnathosomal bay (95–127.5 (116.25) long; 65–77.5 (70) wide). Cxgl-4 subapical. **Medial suture** (92.5–110 (93.75) long). **Genital plates** (105–120 (110) long; 80–90 (83.75) wide). Additional measurements: Cx-1 (210–250 (246) long (total); 84–125 (111) long (medial)); Cx-3 (266–300 (266) wide); anterior venter (235–250 (237.5) long). Ventral proportions: gnathosomal bay length/width 1.46–1.89 (1.66); anterior venter/genital field length 2.08–2.29 (2.16); anterior venter length/genital field width 2.78–3.03 (2.84); anterior venter/medial suture 2.27–2.54 (2.53).

**Immatures** unknown.

######## Etymology.

Specific epithet (*pollani*) named in honor of author Michael Pollan, whose influential books breach mere accounts on food culture and enter insightful discussions of human ecology.

######## Distribution.

**Figure 193.**
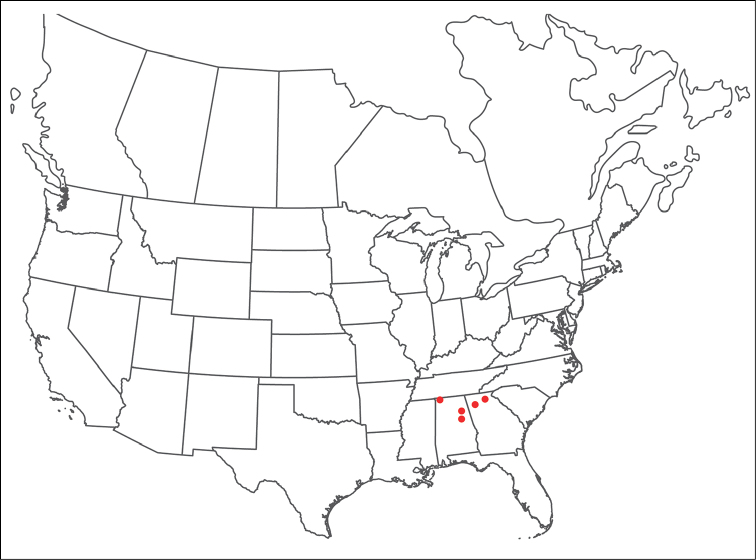
*Torrenticola pollani* sp. n. distribution.

Southeastern (northern Alabama and Georgia) (Figure 193).

**Figure 194.**
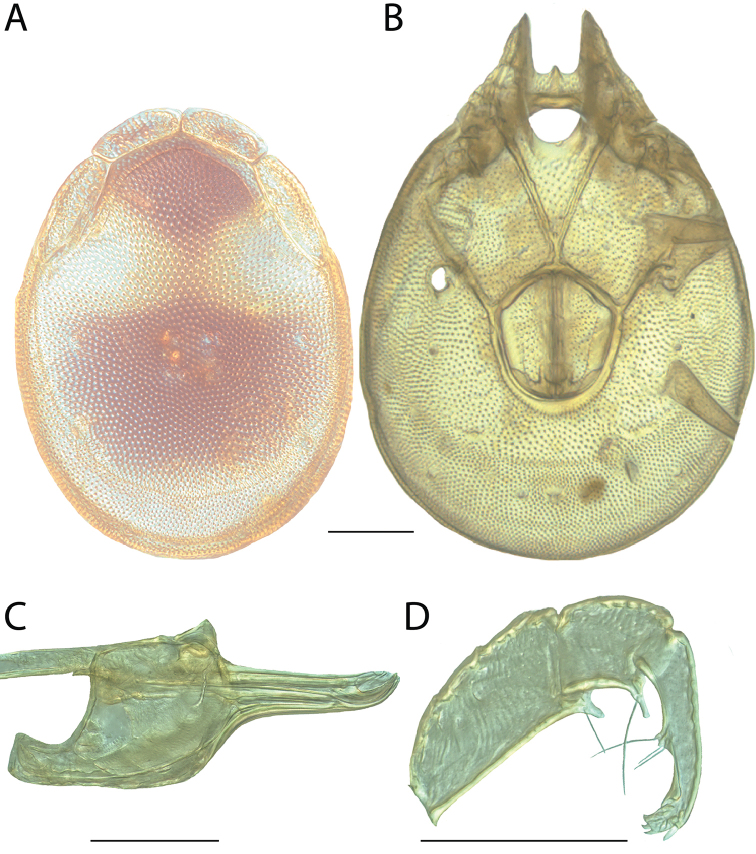
*Torrenticola pollani* sp. n. female: **A** dorsal plates **B** venter (legs removed) **C** subcapitulum **D** pedipalp (setae not accurately depicted). Scale = 100 µm.

**Figure 195.**
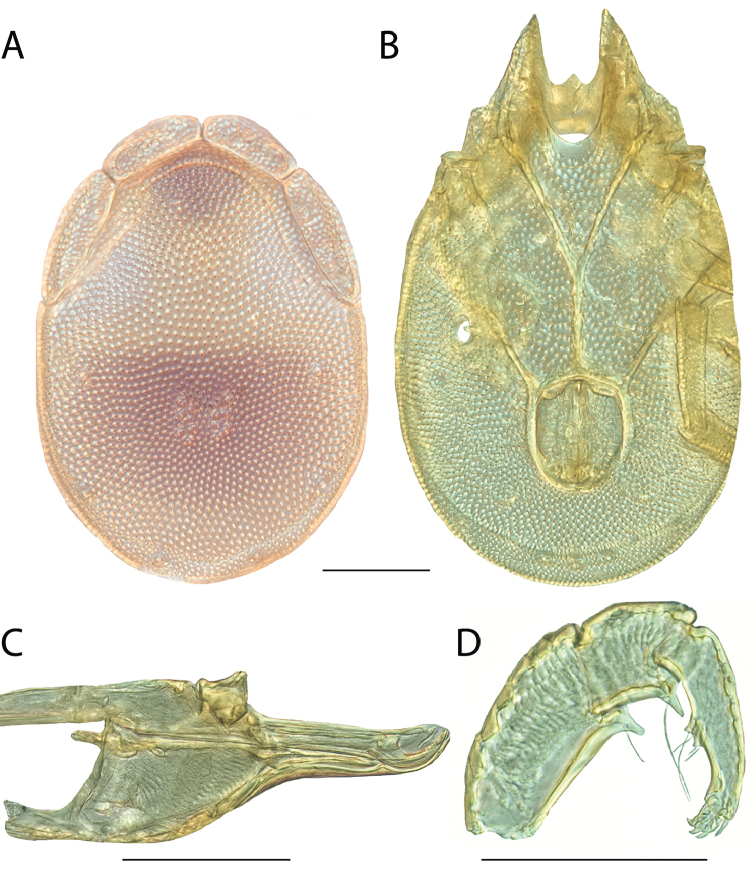
*Torrenticola pollani* sp. n. male: **A** dorsal plates **B** venter (legs removed) **C** subcapitulum **D** pedipalp (setae not accurately depicted). Scale = 100 µm.

######## Remarks.

In all analyses, *Torrenticola pollani* groups with other members of the Rusetria Complex with high support and specimens of this species are less than 1% different in COI sequence from each other. In all analyses, *Torrenticola pollani* groups with two other species with high support: *T. dunni* and *T. shubini*. These species are greater than 5–10% different from each other in COI sequence. Given our collection efforts in the southern Appalachians, it is reasonable to speculate that *T. pollani* does not overlap in range with either *T. dunni* or *T. shubini*. However, our collections are sparse in the coastal plains and we expect future collecting to expand the distribution southward.

Based upon overall similarity, dorso-lateral platelet fusion, and distribution, we were able to place this species within the Eastern 2-Plate Identification Group

This species hypothesis is supported by biogeography, low COI divergence within the species (0–2%) and high divergence between species (3–15%), and by the morphological characters outlined in the diagnosis.

####### Torrenticola priapus

Taxon classificationAnimaliaTrombidiformesTorrenticolidae

Fisher & Dowling sp. n.

http://zoobank.org/262CE779-DB0C-4533-9AE3-CC430B47801A

######## Material examined.

HOLOTYPE (♀): from USA, Texas, Tyler County, Spurger; beside Farm Road 1013, 8.2 km west of Rt. 92, (30°41'41"N, 94°15'15"W), 30 September 1994, by IM Smith, IMS940027A

PARATYPES (5 ♀; 5 ♂): **New Hampshire, USA**: 4 ♀ and 1 ♂ from Woodstock County, beside Rt. 118, 3.2 km south of Rt. 112, (44°0'0"N, 71°45'45"W), 11 September 1992, by IM Smith, IMS920036 • **Texas, USA**: 1 ♂ (ALLOTYPE) from Tyler County, Spurger; beside Farm Road 1013, 8.2 km west of Rt. 92, (30°41'41"N, 94°15'15"W), 30 September 1994, by IM Smith, IMS940027A • 1 ♀ and 3 ♂ from Tyler County, Spurger; beside Farm Road 1013, 8.2 km west of Rt. 92, (30°41'41"N, 94°15'15"W), 30 September 1994, by IM Smith, IMS940027A

######## Type deposition.

Holotype (♀), allotype (♂), and some paratypes (3 ♀; 2 ♂) deposited in the CNC; other paratypes (2 ♀; 2 ♂) deposited in the ACUA.

######## Diagnosis.

*Torrenticola priapus* are similar to other members of the Partial 2-Plate Group (*T. folkertsae*, *T. magnexa*, and *T. pulchra*) in having anterio-lateral platelets partially fused to the dorsal plate and being distributed in the east. *T. priapus* can be differentiated from *T. magnexa* and *T. pulchra* by dorsal coloration and pattern. *T. priapus* can be further differentiated from *T. magnexa* by having a more elongate rostrum (length/width = 3.17–3.39 in *T. priapus*, 2.25–3.00 in *T. magnexa*). *T. priapus* can be further differentiated from *T. pulchra* by having a round dorsum (length/width = 1.18–1.22 in *T. priapus*, 1.4–1.61 in *T. pulchra*) and more elongate pedipalpal tibiae (length/width ♀ = 3.9–4.22 in *T. priapus*, 3.3–3.70 in *T. pulchra*, ♂ = 3.5–3.78 in *T. priapus*, 3.00–3.35 in *T. pulchra*). *T. priapus* can be differentiated from *T. folkertsae* by having less elongate pedipalpal tibiae (length/width ♀ = 3.9–4.22 in *T. priapus*, 4.5–4.83 in *T. folkertsae*, ♂ = 3.5–3.78 in *T. priapus*, 4.05–4.33 in *T. folkertsae*) and a more elongate rostrum (length/width = 3.17–3.39 in *T. priapus*, 2.55–3.00 in *T. folkertsae*).

######## Description.

**Female (Figure 197)** (n = 5) (holotype measurements in parentheses when available) with characters of the genus with following specifications.

**Dorsum** — (610–690 (650) long; 510–580 (540) wide) circular with purple coloration posteriorly and anteriorly connected medially but not extending to platelets. Anterio-medial platelets (150–170 (162.5) long; 62.5–70 (65) wide). Anterio-lateral platelets (175–190 (185) long; 75–95 (95) wide) partially fused to dorsal plate. Dgl-4 nearly halfway between the edge of the dorsum and the muscle scars (distance between Dgl-4 305–370 (345)). Dorsal plate proportions: dorsum length/width 1.19–1.21 (1.20); dorsal width/distance between Dgl-4 1.56–1.67 (1.57); anterio-medial platelet length/width 2.37–2.50 (2.50); anterio-lateral platelet length/width 1.95–2.33 (1.95); anterio-lateral/anterio-medial length 1.12–1.23 (1.14).

**Gnathosoma — Subcapitulum** (325–355 (355) long (ventral); 245–270 (270) long (dorsal); 137.5–155 (150) tall) colorless. Rostrum (142.5–152.5 (152.5) long; 45–45 (45) wide). Chelicerae (330–370 (360) long) with curved fangs (55–65 (65) long). Subcapitular proportions: ventral length/height 2.28–2.37 (2.37); rostrum length/width 3.17–3.39 (3.39). **Pedipalps** with tuberculate ventral extensions on femora and genua. Palpomeres: trochanter (42.5–50 (48.75) long); femur (118.75–135 (135) long); genu (70–80 (76.25) long); tibia (92.5–105 (97.5) long; 22.5–25 (25) wide); tarsus (17.5–22.5 (22.5) long). Palpomere proportions: femur/genu 1.69–1.77 (1.77); tibia/femur 0.72–0.84 (0.72); tibia length/width 3.90–4.22 (3.90).

**Venter** — (720–850 (770) long; 590–645 (605) wide) colorless. Gnathosomal bay (160–192.5 (192.5) long; 77.5–90 (80) wide). Cxgl-4 subapical. **Medial suture** (15–15 (15) long). **Genital plates** (180–190 (190) long; 167.5–185 (185) wide). Additional measurements: Cx-1 (280–320 (315) long (total); 115–135 (130) long (medial)); Cx-3 (355–390 (390) wide); anterior venter (150–165 (160) long). Ventral proportions: gnathosomal bay length/width 2.00–2.41 (2.41); anterior venter/genital field length 0.81–0.89 (0.84); anterior venter length/genital field width 0.86–0.97 (0.86); anterior venter/medial suture 10.00–11.00 (10.67).

**Male (Figure 198)** (n = 4) (allotypic measurements in parentheses when available) with characters of the genus with following specifications.

**Dorsum** — (530–585 (530) long; 435–495 (435) wide) circular with purple coloration posteriorly and anteriorly connected medially but not extending to platelets. Anterio-medial platelets (131.25–145 (131.25) long; 52.5–62.5 (55) wide). Anterio-lateral platelets (165–182.5 (170) long; 70–77.5 (70) wide) partially fused to dorsal plate. Dgl-4 nearly halfway between the edge of the dorsum and the muscle scars (distance between Dgl-4 265–330 (265)). Dorsal plate proportions: dorsum length/width 1.18–1.22 (1.22); dorsal width/distance between Dgl-4 1.50–1.70 (1.64); anterio-medial platelet length/width 2.32–2.76 (2.39); anterio-lateral platelet length/width 2.13–2.43 (2.43); anterio-lateral/anterio-medial length 1.14–1.30 (1.30).

**Gnathosoma — Subcapitulum** (305–320 (305) long (ventral); 230–245 (235) long (dorsal); 125–130 (125) tall) colorless. Rostrum (127.5–140 (127.5) long; 40–42.5 (40) wide). Chelicerae (305–320 (305) long) with curved fangs (57.5–60 (60) long). Subcapitular proportions: ventral length/height 2.44–2.48 (2.44); rostrum length/width 3.19–3.29 (3.19). **Pedipalps** with tuberculate ventral extensions on femora and genua. Palpomeres: trochanter (42.5–45 (42.5) long); femur (110–117.5 (110) long); genu (67.5–72.5 (67.5) long); tibia (85–93.75 (85) long; 22.5–25 (22.5) wide); tarsus (20–22.5 (20) long). Palpomere proportions: femur/genu 1.62–1.70 (1.63); tibia/femur 0.76–0.80 (0.77); tibia length/width 3.50–3.78 (3.78).

**Venter** — (625–695 (625) long; 510–560 (540) wide) colorless. Gnathosomal bay (145–157.5 (145) long; 70–77.5 (70) wide). Cxgl-4 subapical. **Medial suture** (75–80 (75) long). **Genital plates** (165–185 (165) long; 145–160 (150) wide). Additional measurements: Cx-1 (270–305 (285) long (total); 120–140 (140) long (medial)); Cx-3 (340–360 (350) wide); anterior venter (220–250 (230) long). Ventral proportions: gnathosomal bay length/width 2.00–2.25 (2.07); anterior venter/genital field length 1.33–1.42 (1.39); anterior venter length/genital field width 1.47–1.61 (1.53); anterior venter/medial suture 2.93–3.13 (3.07).

**Immatures** unknown.

######## Etymology.

Specific epithet (*priapus*) is named for Priapus, the Greek fertility god who was marked by his oversized, permanent erection, which refers to the genital opening in the male—the largest of all North American *Torrenticola*.

######## Distribution.

**Figure 196.**
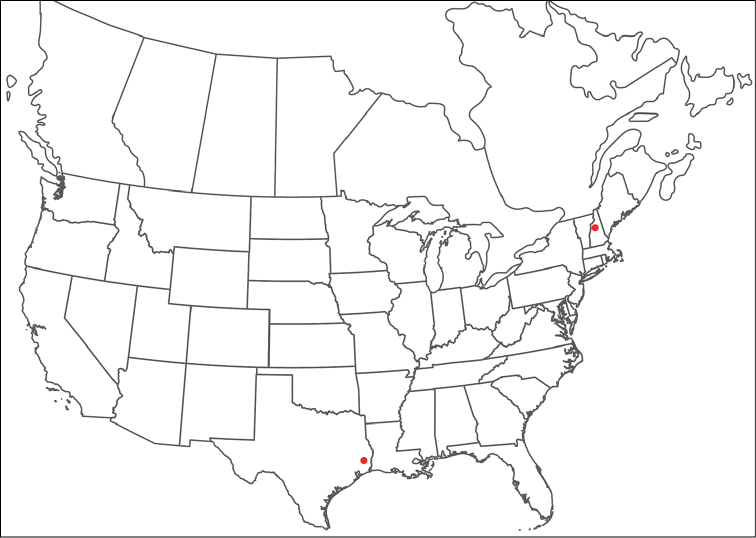
*Torrenticola priapus* sp. n. distribution.

Eastern (Figure 196). Distribution seemingly disjunct (Texas and New Hampshire).

**Figure 197.**
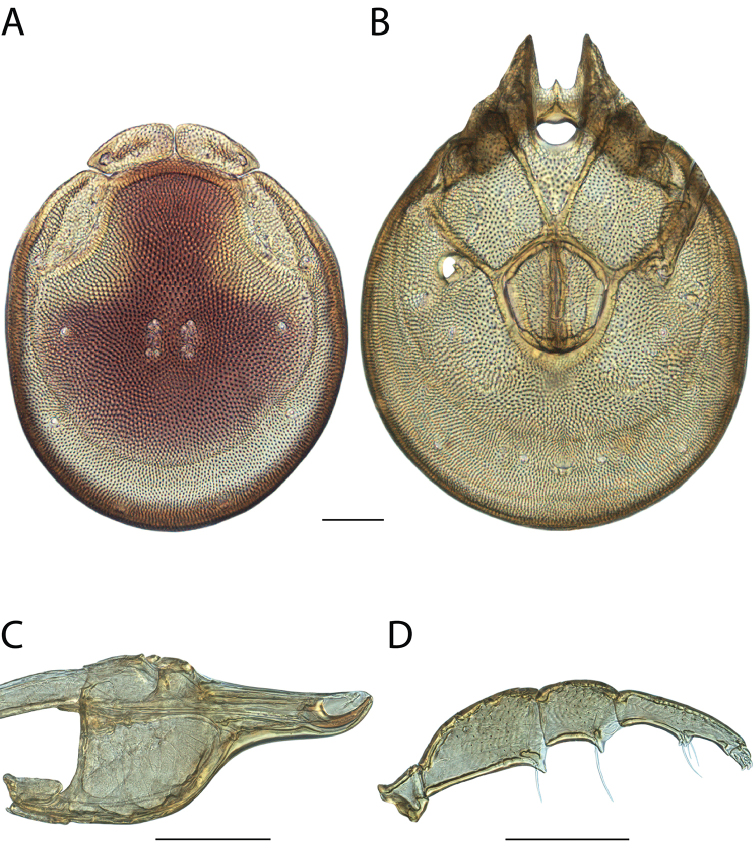
*Torrenticola priapus* sp. n. female: **A** dorsal plates **B** venter (legs removed) **C** subcapitulum **D** pedipalp (setae not accurately depicted). Scale = 100 µm.

**Figure 198.**
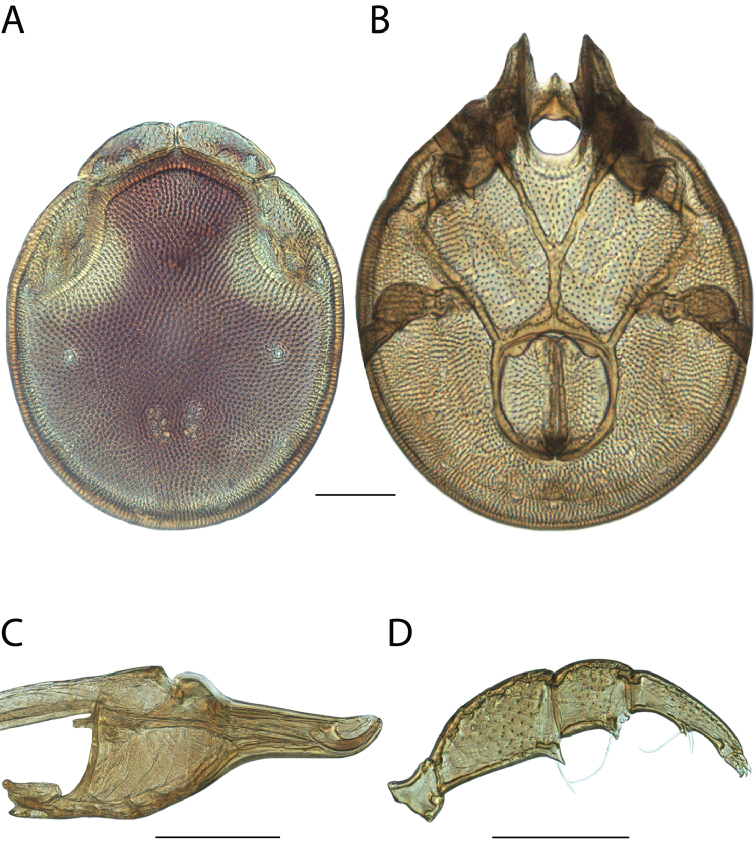
*Torrenticola priapus* sp. n. male: **A** dorsal plates **B** venter (legs removed) **C** subcapitulum **D** pedipalp (setae not accurately depicted). Scale = 100 µm.

######## Remarks.

Unfortunately, we were unable to acquire fresh material of *Torrenticola priapus* and therefore this species is not included in our phylogenetic analyses. However, we were able to examine morphology with material preserved in GAW. The overall similarity, distribution, and partial fusion of the dorso-lateral platelets to the dorsal plate, are consistent with placing this species in the Rusetria Complex and within the Partial 2-Plate Identification Group.

####### Torrenticola projector

Taxon classificationAnimaliaTrombidiformesTorrenticolidae

Habeeb, 1961

Torrenticola projector Habeeb, 1961: 1.

######## Material examined.

LECTOTYPE (1 ♀): from USA, New York, Cayuga County, Moravia, brook, 22 May 1960, by H Habeeb, HH600011.

PARALECTOTYPE (1 ♂): from USA, New York, Cayuga County, Moravia, brook, 22 May 1960, by H Habeeb, HH600011.

OTHER MATERIAL (25 ♀; 27 ♂): **Alabama, USA**: 4 ♀ and 4 ♂ from Clay County, Talladega Creek, beside Forest Route 649, 0.8 kilometers northeast of road from Forest Route 600 to Campbell Springs, 2 Jul 1990, by IM Smith, IMS900075A • 1 ♂ from Cleburne County, beside Route 431, 3.3 kilometers southeast of Calhoun, Jackson Creek (33°36'N, 85°42'W), 2 Jul 1990, by IM Smith, IMS900074 • 2 ♀ and 1 ♂ from Lauderdale County, off Natchez Trace Parkway (34°56'31"N, 87°49'41"W), 24 Sep 2009, by IM Smith, IMS090121 • 1 ♂ from Lauderdale County, off Natchez Trace Parkway (34°56'31"N, 87°49'41"W), 27 Sep 2010, by IM Smith, IMS100162 • **Georgia, USA**: 1 ♂ from Chattooga County, beside road from Everett Springs to Villanow, 1.4 kilometers south of Pocket Recreation Area, 4 Jul 1990, by IM Smith, IMS900077 • 1 ♀ and 2 ♂ from Floyd County, tributary of Johns Creek, beside road from Everett Springs to Villanow, 1.4 kilometers south of The Pocket Campground, 4 Jul 1990, by IM Smith, IMS900077 • **New York, USA**: 3 ♀ and 2 ♂ from Cayuga County, Dutch Hollow Brook, beside Route 38A at Niles, 22 Jul 1990, by IM Smith, IMS900113A • 2 ♀ and 1 ♂ from Schuyler County, beside Town Line Road off Route 228, 0.6 kilometers south of Perry City, 21 Jul 1990, by IM Smith, IMS900112A • **North Carolina, USA**: 3 ♀ and 5 ♂ from Yancey County, South Toe River, Lost Cove Picnic Area on Forest Route 472, 2.8 kilometers south of Route 80, 28 Jun 1990, by IM Smith, IMS900065A • 2 ♂ from Yancey County, South Toe River (35°45'10"N, 82°12'43"W), 28 Jun 1990, by IM Smith, IMS900065A • **Pennsylvania, USA**: 1 ♀ from Fayette County, Dunbar Creek (39°57'50"N, 79835'3.70"W), 10 Aug 2014, by MJ Skvarla, MS 14-0810-001• 1 ♂ from Fayette County, Dunbar Creek (39°56'16.10"N, 79°35'3.70"W), 10 Aug 2014, by MJ Skvarla, MS 14-0810-002 • **Tennessee, USA**: 2 ♂ from Monroe County, Turkey Creek , beside Forest Route 35, 2.0 kilometers northeast of road from Route 165 to Miller Chapel Church, 5 Jul 1990, by IM Smith, IMS900078 • 2 ♀ from Sevier County, Great Smoky Mountains National Park, Middle Prong Pigeon River (35°43'32"N, 83°24'2"W), 2 Sep 2009, by IM Smith, IMS090093 • 3 ♀ and 1 ♂ from Sevier County, Great Smoky Mountains National Park, Sugarlands Nature Trail (35°40'47"N, 83°31'51"W), 10 Sep 2010, by IM Smith, IMS100125 • **Virginia, USA**: 1 ♂ from Amherst County, Upper Otter Creek Overlook beside Blue Ridge, Otter Creek (37°36'57"N, 79°19'27"W), 7 Sep 2007, by IM Smith, IMS070056A • 4 ♀ and 2 ♂ from Washington County, Damascus, Laurel River, beside Route 58 near boundary of Mount Rogers National Recreation Area, 10 Jul 1990, by IM Smith, IMS900085A.

######## Type deposition.

Types (1 ♀; 1 ♂) deposited in the CNC.

######## Diagnosis.

*Torrenticola projector* are unlike nearly all other *Torrenticola* in having such elongate bodies and subcapitula. *T. elongata* are also elongate, but have different dorsal coloration and do not have an elongate subcapitulum. *T. tahoei* also have elongate subcapitula, but have rounder bodies with different dorsal coloration. *T. anoplopalpa* also have elongate subcapitula, but have rounder bodies and incomplete hind coxae.

######## Re-description.

**Female (Figure 200)** (n = 5) with characters of the genus with following specifications.

**Dorsum** — (625–745 long; 400–470 wide) ovoid and elongate with pink coloration without distinct pattern. Anterio-medial platelets (95–115 long; 55–62.5 wide). Anterio-lateral platelets (185–217.5 long; 50–57.5 wide) free from dorsal plate. Dgl-4 much closer to the edge of the dorsum than to the muscle scars (distance between Dgl-4 320–370). Dorsal plate proportions: dorsum length/width 1.52–1.68; dorsal width/distance between Dgl-4 1.24–1.28; anterio-medial platelet length/width 1.73–2.05; anterio-lateral platelet length/width 3.36–3.90; anterio-lateral/anterio-medial length 1.76–1.95.

**Gnathosoma — Subcapitulum** (382.5–445 long (ventral); 300–333 long (dorsal); 87.5–97.5 tall) colorless and elongate. Rostrum (142.5–172.5 long; 37.5–42.5 wide) elongate. Chelicerae (360–428 long) with curved fangs (51–62 long). Subcapitular proportions: ventral length/height 4.03–4.78; rostrum length/width 3.80–4.27. **Pedipalps** short and stocky (especially tibiae) without extensions on femora and genua. Palpomeres: trochanter (47.5–55 long); femur (108.75–125 long); genu (60–70 long); tibia (21.25–25 long; 21.25–25 wide); tarsus (20–22.5 long). Palpomere proportions: femur/genu 1.74–1.81; tibia/femur 0.55–0.60; tibia length/width 2.82–3.11.

**Venter** — (830–931 long; 480–544 wide) colorless. Gnathosomal bay (92.5–110 long; 67.5–80 wide). Cxgl-4 far from apex. **Medial suture** (50–77.5 long). **Genital plates** 183.75–188.75 long; 147.5–160 wide). Additional measurements: Cx-1 (296.75–357 long (total); 215–250 long (medial)); Cx-3 (310–374 wide); anterior venter (285–340 long). Ventral proportions: gnathosomal bay length/width 1.22–1.63; anterior venter/genital field length 1.55–1.84; anterior venter length/genital field width 1.78–2.16; anterior venter/medial suture 4.39–6.05.

**Male (Figure 201)** (n = 6) with characters of the genus with following specifications.

**Dorsum** — (540–630 (600) long; 335–400 wide) ovoid and elongate with pink coloration without distinct pattern. Anterio-medial platelets (90–112.5 long; 47.5–57.5 wide). Anterio-lateral platelets (170–205 long; 47.5–57.5 wide) free from dorsal plate. Dgl-4 much closer to the edge of the dorsum than to the muscle scars (distance between Dgl-4 265–335). Dorsal plate proportions: dorsum length/width 1.54–1.61); dorsal width/distance between Dgl-4 1.16–1.26; anterio-medial platelet length/width 1.70–2.20; anterio-lateral platelet length/width 3.23–3.68; anterio-lateral/anterio-medial length 1.61–2.00.

**Gnathosoma — Subcapitulum** (325–420 long (ventral); 245–334 long (dorsal); 78.75–90 tall) colorless and elongate. Rostrum (127.5–158.75 long; 31.25–35 wide) elongate. Chelicerae (297–382 long) with curved fangs (41–61 long). Subcapitular proportions: ventral length/height 3.94–4.94; rostrum length/width 3.64–4.54. **Pedipalps** short and stocky (especially tibiae) without extensions on femora and genua. Palpomeres: trochanter (45–50 long); femur (100–113.75 long); genu (62.5–67.5 long); tibia (62.5–67.5 long; 22.5–22.5 wide); tarsus (17.5–20 long). Palpomere proportions: femur/genu 1.58–1.75; tibia/femur 0.59–0.64; tibia length/width 2.78–3.00.

**Venter** — (680–860 long; 405–492 wide) colorless. Gnathosomal bay (82.5–132.5 long; 60–75 wide). Cxgl-4 far from apex. **Medial suture** (127.5–142.5 long). **Genital plates** (137.5–157.5 long; 100–111.25 wide). Additional measurements: Cx-1 (252–392 long (total); 175–267 long (medial)); Cx-3 (282–355 wide); anterior venter (320–410 long). Ventral proportions: gnathosomal bay length/width 1.38–2.04; anterior venter/genital field length 2.27–2.64; anterior venter length/genital field width 3.20–3.71; anterior venter/medial suture 2.39–3.15.

**Immatures** unknown.

######## Etymology.

Habeeb (1961) did not specify an etymology for the specific epithet (*projector*). However, surely this name reflects the elongated, projectable gnathosoma (*proiectus*, L. stick out)

######## Distribution.

**Figure 199.**
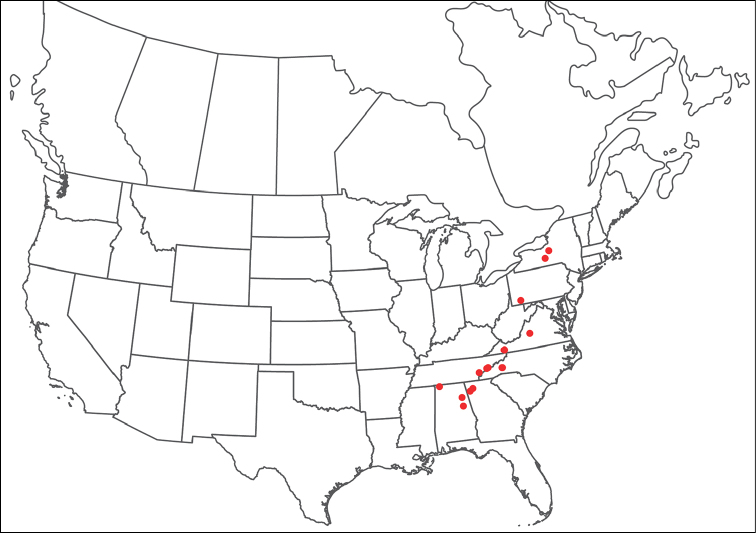
*Torrenticola projector* distribution.

Appalachians (Figure 199). *T. projector* was previously known only from New York; we extend its range throughout the Appalachians.

**Figure 200.**
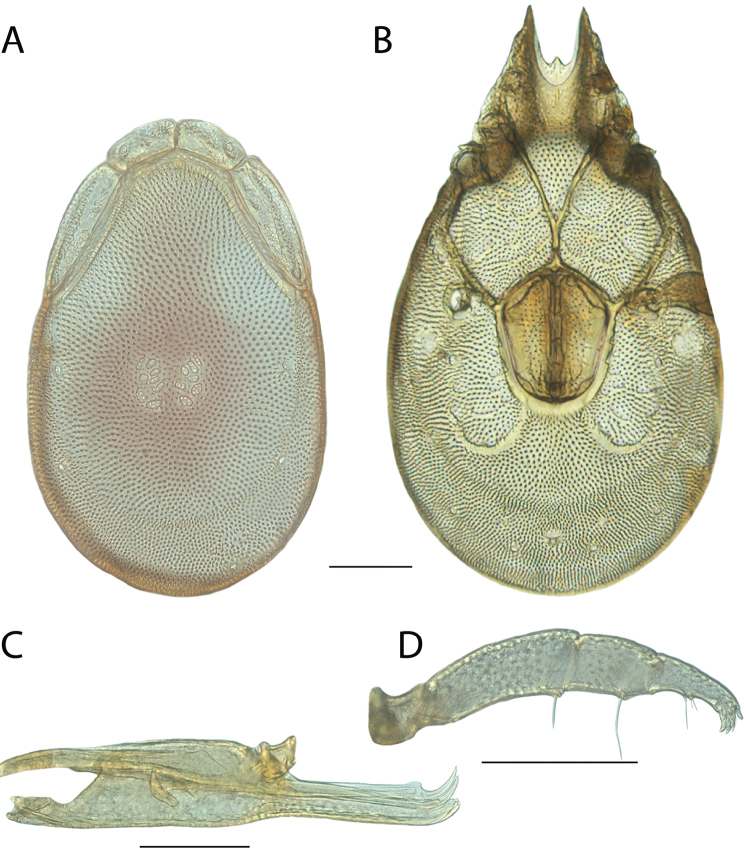
*Torrenticola projector* female: **A** dorsal plates **B** venter (legs removed) **C** subcapitulum **D** pedipalp (setae not accurately depicted). Scale = 100 µm.

**Figure 201.**
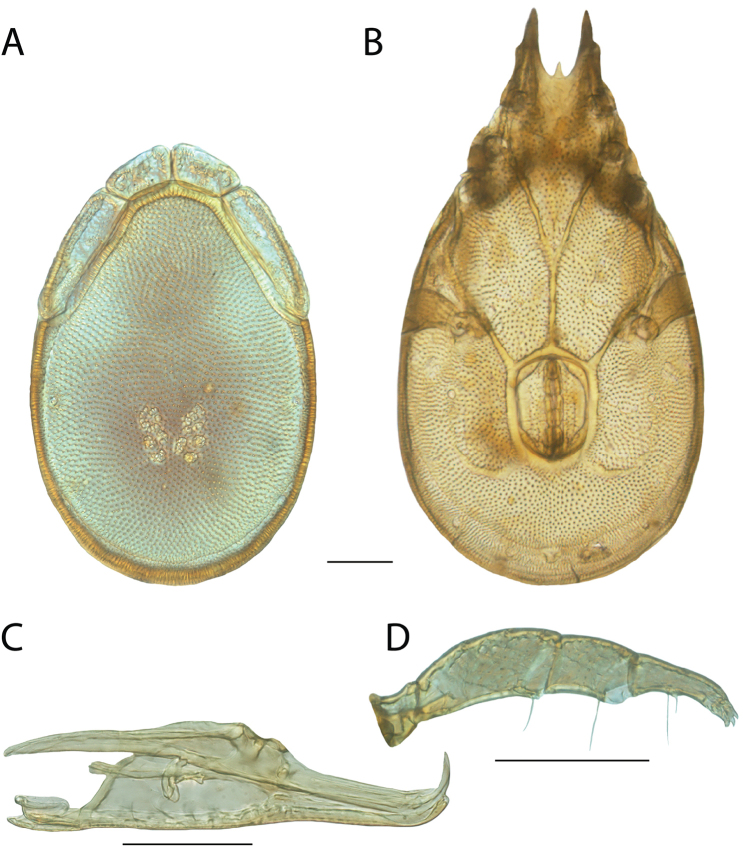
*Torrenticola projector* male: **A** dorsal plates **B** venter (legs removed) **C** subcapitulum **D**pedipalp (setae not accurately depicted). Scale = 100 µm.

######## Remarks.

*Torrenticola projector* groups with other members of the Tricolor Complex in all analyses with high support and specimens are less than 1% different in COI sequence from each other. In the combined analysis, *T. projector* groups with two other species (*T. hoosieri* and *T. pearsoni*) with high support and these species are greater than 4% different from each other. Whereas most eastern members of the Tricolor Complex have distinctive patterns, this clade of three species contains members that lack dark patterns. *T. projector* is among the most distinctive of all *Torrenticola* because of the highly elongated gnathosoma and body. Because of this distinctive morphology, we do not place this species into an identification group.

This species hypothesis is supported by low COI divergence within the species (0–2%), high divergence between species (3–15%), and the morphological characters outlined in the diagnosis.

####### Torrenticola pulchra

Taxon classificationAnimaliaTrombidiformesTorrenticolidae

Fisher & Dowling sp. n.

http://zoobank.org/6DFF72DC-131D-4C43-AE70-B25E8888FD41

######## Material examined.

HOLOTYPE (♀): from, USA, Illinois, Pope County, Eddyville; Bell Smith Springs Recreation Area; just below low water bridge at Hunting Branch Picnic Area, (37°31'31"N, 88°39'39"W), 1 September 2006, by IM Smith, S Yi, IMS060057.

PARATYPES (4 ♀; 5 ♂): **Illinois, USA**: 1 ♂ (ALLOTYPE) from Pope County, Eddyville; Bell Smith Springs Recreation Area; just below low water bridge at Hunting Branch Picnic Area, (37°31'31"N, 88°39'39"W), 1 September 2006, by IM Smith, S Yi, IMS060057 • 3 ♀ and 3 ♂ from Pope County, Eddyville; Bell Smith Springs Recreation Area; just below low water bridge at Hunting Branch Picnic Area, (37°31'31"N, 88°39'39"W), 1 September 2006, by IM Smith, S Yi, IMS060057 • 1 ♀ and 1 ♂ from Pope County, Eddyville; Bell Smith Springs Recreation Area; Hunting Branch Picnic Area, (37°31'31"N, 88°40'40"W), 9 September 1994, by IM Smith, IMS940004

######## Type deposition.

Holotype (♀), allotype (♂), and some paratypes (2 ♀; 2 ♂) deposited in the CNC; other paratypes (2 ♀; 2 ♂) deposited in the ACUA.

######## Diagnosis.

*Torrenticola pulchra* are similar to other members of the Partial 2-Plate Group (*T. folkertsae*, *T. magnexa*, and *T. priapus*) in having anterio-lateral platelets partially fused to the dorsal plate and being distributed in the east. *T. pulchra* can be differentiated from other Partial 2-Plate Group by dorsal coloration and pattern. *T. pulchra* can be further differentiated from *T. priapus* and *T. folkertsae* by having a more ovoid dorsum (length/width = 1.4–1.61 in *T. pulchra*, 1.11–1.29 in others) and less elongate pedipalpal tibiae (length/width ♀ = 3.3–3.70 in *T. pulchra*, 3.9–4.83 in others, ♂ = 3.00–3.35 in *T. pulchra*, 3.5–4.33 in others). Male *T. pulchra* can be further differentiated from male *T. magnexa* by having a smaller genital field (length ♂ = 110–123 in *T. pulchra*, 125–148 in *T. magnexa*; width ♂ = 87–95 in *T. pulchra*, 115–125 in *T. magnexa*,) and less elongate pedipalpal tibiae (length/width ♂ = 3.00–3.35 in *T. pulchra*, 3.78–4.00 in *T. magnexa*,). Female *T. pulchra* can be differentiated from female *T. magnexa* by having a thinner genital field (♀ 147–160 in *T. pulchra*, 170–188 in *T. magnexa*) and shorter pedipalpal tibiae (♀ 82–93 in *T. pulchra*, 102–113 in *T. magnexa*).

######## Description.

**Female (Figure 203)** (n = 5) (holotype measurements in parentheses when available) with characters of the genus with following specifications.

**Dorsum** — (640–710 (710) long; 450–490 (480) wide) ovoid with bold blue coloration separated into anterior and posterior portions, posterior portion not meeting posterior limit of dorsal plate, and bold red medially. Anterio-medial platelets (130–145 (135) long; 50–55 (55) wide). Anterio-lateral platelets (177.5–200 (190) long; 60–70 (60) wide) nearly fused to dorsal plate posteriorly. Dgl-4 closer to the edge of the dorsum than to the muscle scars (distance between Dgl-4 320–375 (355)). Dorsal plate proportions: dorsum length/width 1.40–1.48 (1.48); dorsal width/distance between Dgl-4 1.30–1.45 (1.35); anterio-medial platelet length/width 2.45–2.90 (2.45); anterio-lateral platelet length/width 2.77–3.17 (3.17); anterio-lateral/anterio-medial length 1.33–1.41 (1.41).

**Gnathosoma — Subcapitulum** (295–345 (345) long (ventral); 215–252.5 (252.5) long (dorsal); 130–155 (155) tall) faint blue coloration. Rostrum (117.5–135 (135) long; 40–45 (45) wide). Chelicerae (295–350 (350) long) with curved fangs (60–70 (70) long). Subcapitular proportions: ventral length/height 2.03–2.33 (2.23); rostrum length/width 2.94–3.06 (3.00). **Pedipalps** with tuberculate ventral extensions on femora and genua. Palpomeres: trochanter (47.5–52.5 (52.5) long); femur (118.75–132.5 (130) long); genu (67.5–75 (72.5) long); tibia (82.5–92.5 (90) long; 25–26.25 (25) wide); tarsus (17.5–20 (20) long). Palpomere proportions: femur/genu 1.73–1.80 (1.79); tibia/femur 0.69–0.72 (0.69); tibia length/width 3.30–3.70 (3.60).

**Venter** — (740–840 (840) long; 530–560 (560) wide) with blue coloration. Gnathosomal bay (155–190 (190) long; 75–90 (75) wide). Cxgl-4 subapical. **Medial suture** (10–20 (10) long). **Genital plates** (180–200 (192.5) long; 147.5–160 (155) wide). Additional measurements: Cx-1 (280–350 (350) long (total); 130–162.5 (162.5) long (medial)); Cx-3 (325–365 (360) wide); anterior venter (160–175 (175) long). Ventral proportions: gnathosomal bay length/width 1.94–2.53 (2.53); anterior venter/genital field length 0.80–0.96 (0.91); anterior venter length/genital field width 1.00–1.13 (1.13); anterior venter/medial suture 8.00–17.50 (17.50).

**Male (Figure 204)** (n = 5) (allotypic measurements in parentheses when available) with characters of the genus with following specifications.

**Dorsum** — (490–520 (500) long; 310–330 (315) wide ovoid with bold blue coloration separated into anterior and posterior portions, posterior portion not meeting posterior limit of dorsal plate, and bold red medially. Anterio-medial platelets (95–110 (100) long; 40–47.5 (42.5) wide). Anterio-lateral platelets (140–160 (145) long; 45–55 (50) wide) nearly fused to dorsal plate posteriorly. Dgl-4 closer to the edge of the dorsum than to the muscle scars (distance between Dgl-4 235–265 (245)). Dorsal plate proportions: dorsum length/width 1.53–1.61 (1.59); dorsal width/distance between Dgl-4 1.23–1.36 (1.29); anterio-medial platelet length/width 2.24–2.50 (2.35); anterio-lateral platelet length/width 2.80–3.56 (2.90); anterio-lateral/anterio-medial length 1.45–1.55 (1.45).

**Gnathosoma — Subcapitulum** (255–262.5 (255) long (ventral); 182.5–195 (182.5) long (dorsal); 90–95 (90) tall) faint blue coloration. Rostrum (97.5–105 (97.5) long; 30–32.5 (30) wide). Chelicerae (240–255 (245) long) with curved fangs (45–50 (45) long). Subcapitular proportions: ventral length/height 2.68–2.83 (2.83); rostrum length/width 3.23–3.50 (3.25). **Pedipalps** with tuberculate ventral extensions on femora and genua. Palpomeres: trochanter (37.5–42.5 (37.5) long); femur (97.5–100 (100) long); genu (55–57.5 (57.5) long); tibia (67.5–72.5 (70) long; 21.25–22.5 (22.5) wide); tarsus (15–17.5 (15) long). Palpomere proportions: femur/genu 1.70–1.82 (1.74); tibia/femur 0.69–0.73 (0.70); tibia length/width 3.00–3.35 (3.11).

**Venter** — (600–625 (600) long; 355–390 (355) wide) with blue coloration. Gnathosomal bay (120–130 (120) long; 60–67.5 (67.5) wide). Cxgl-4 subapical. **Medial suture** (105–130 (125) long). **Genital plates** (110–122.5 (115) long; 87.5–95 (92.5) wide). Additional measurements: Cx-1 (240–255 (240) long (total); 122.5–130 (125) long (medial)); Cx-3 (275–295 (280) wide); anterior venter (235–270 (255) long). Ventral proportions: gnathosomal bay length/width 1.78–2.08 (1.78); anterior venter/genital field length 2.00–2.32 (2.22); anterior venter length/genital field width 2.61–2.91 (2.76); anterior venter/medial suture 2.04–2.24 (2.04).

**Immatures** unknown.

######## Etymology.

Specific epithet (*pulchra*) refers to the bright and distinctive coloration of this species (*pulchra*, L. beautiful).

######## Distribution.

**Figure 202.**
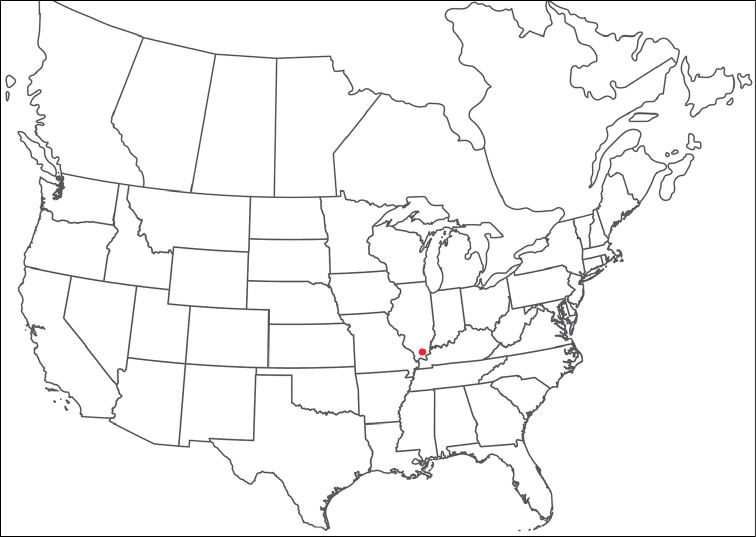
*Torrenticola pulchra* sp. n. distribution.

Southern Illinois (Figure 202).

**Figure 203.**
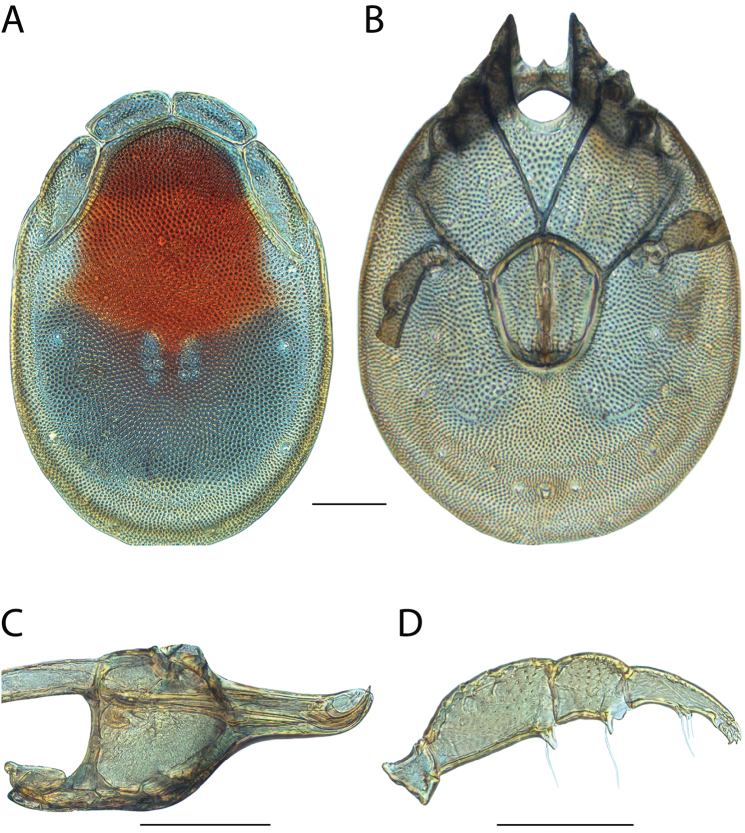
*Torrenticola pulchra* sp. n. female: **A** dorsal plates **B** venter (legs removed) **C** subcapitulum **D** pedipalp (setae not accurately depicted). Scale = 100 µm.

**Figure 204.**
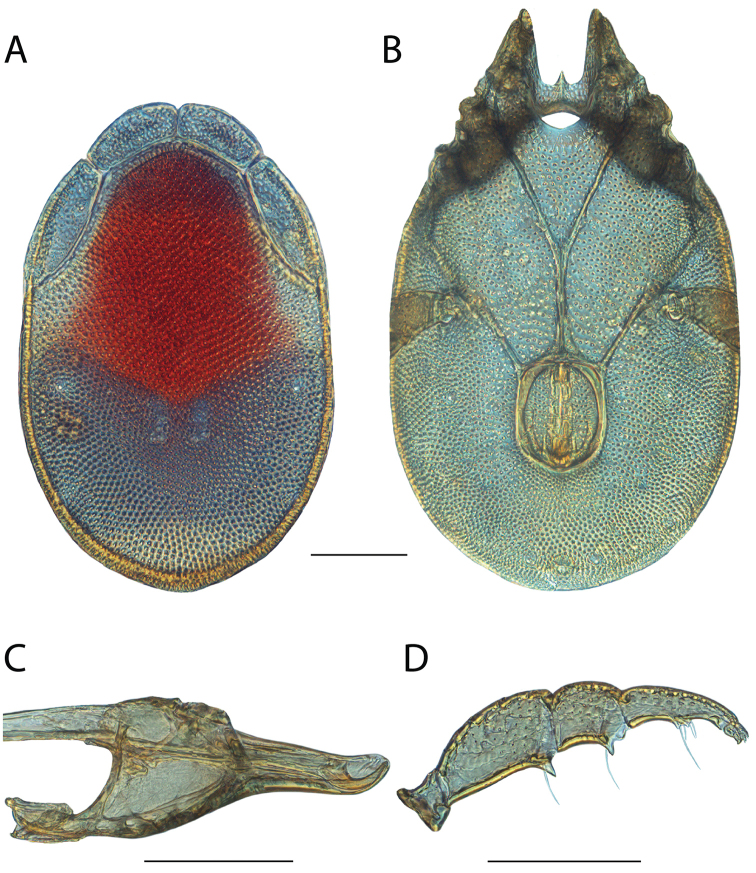
*Torrenticola pulchra* sp. n. male: **A** dorsal plates **B** venter (legs removed) **C** subcapitulum **D** pedipalp (setae not accurately depicted). Scale = 100 µm.

######## Remarks.

Unfortunately, we were unable to acquire fresh material of *Torrenticola pulchra* and therefore this species is not included in our phylogenetic analyses. However, we were able to examine morphology with material preserved in GAW. The overall similarity, distribution, and partial fusion of the dorso-lateral platelets to the dorsal plate, are consistent with placing this species in the Rusetria Complex and within the Partial 2-Plate Identification Group.

####### Torrenticola racupalpa

Taxon classificationAnimaliaTrombidiformesTorrenticolidae

Fisher & Dowling sp. n.

http://zoobank.org/A920D498-57BC-4F1D-9A01-6EF24095B853

######## Material examined.

HOLOTYPE (♀): from USA, Tennessee, Wayne County, Glenrock Branch Creek (35°15'50"N, 87°37'34"W), 24 Sep 2009, by IM Smith, IMS090124, DNA 1867.

PARATYPES (4 ♀; 2 ♂): **Virginia, USA**: 4 ♀ and 2 ♂ (ALLOTYPE) from Smyth County, Mount Rogers National Recreation Area, Little Laurel Creek, beside Route 600, 2.2 kilometers north of Route 603, 10 Jul 1990, by IM Smith, IMS900086.

######## Type deposition.

Holotype (♀), allotype (♂), and most paratypes (3 ♀) deposited in the CNC; other paratypes (1 ♀; 1 ♂) deposited in the ACUA.

######## Diagnosis.

*Torrenticola racupalpa* are similar to other members of the Raptor Group (*T. gnoma*, *T. irapalpa*, *T. longitibia*, *T. mjolniri*, *T. elusiva*, *T. raptor*, *T. danielleae*, *T. daemon*, and *T. ivyae*) in having round bodies; Dgl-4 close to muscles scars; long, thin subcapitular rostra; and long, thin pedipalp tibiae. Female *T. racupalpa* can be differentiated from *T. elusiva* by having a more elongate subcapitulum (ventral length/height = 2.48–2.73 in *T. racupalpa*, 2.39 in *T. elusiva*); and by dorsal pattern. *T. racupalpa* can be differentiated from *T. irapalpa* and *T. daemon* by having Dgl-4 closer to the muscle scars (dorsal width/distance between Dgl-4 = 2.19–2.77 in *T. racupalpa*, 1.45–2.09 in others) and a more elongate rostrum (length/width = 3.56–3.88 in *T. racupalpa*, 2.66–3.39 in others). *T. racupalpa* can be differentiated from *T. gnoma* by having a more elongate rostrum (length/width = 3.56–3.88 in *T. racupalpa*, 2.56–3.13 in *T. gnoma*) and dorsal coloration and pattern. *T. racupalpa* can be differentiated from *T. mjolniri* by having a shorter anterior venter (♀ =152.5–165 in *T. racupalpa*, 180–195 in *T. mjolniri*; ♂ = 200–205 in *T. racupalpa*, 230–255 in *T. mjolniri*); and by dorsal pattern. Male *T. racupalpa* can be differentiated from *T. longitibia* (males only) by having a shorter femur with respect to the genu (femur/genu = 1.92–1.92 in *T. racupalpa*, 2.10–2.17 in *T. longitibia*); a stockier rostrum (length/width = 3.88–3.88 in *T. racupalpa*, 4.15–4.23 in *T. longitibia*); and dorsal pattern. *T. racupalpa* can be differentiated from *T. raptor* by having Dgl-4 closer to the muscle scars (dorsal width/distance between Dgl-4 = 2.19–2.77 in *T. racupalpa*, 1.68–2.02 in *T. raptor*); and shorter anterior venter (♀ = 152.5–165 in *T. racupalpa*, 205–240 in *T. raptor*, ♂ = 200–205 in *T. racupalpa*, 245–305 in *T. raptor*). Female *T. racupalpa* can be differentiated from female *T. raptor* by having stockier pedipalp tibiae (length/width = 4.44–5.00 in *T. racupalpa*, 6–7.54 in *T. raptor*). *T. racupalpa* can be differentiated from *T. danielleae* by having Dgl-4 closer to the muscle scars (dorsal width/distance between Dgl-4 = 2.19–2.77 in *T. racupalpa*, 1.42–1.70 in *T. danielleae*) and by dorsal coloration and pattern. Female *T. racupalpa* can be differentiated from female *T. ivyae* by having a less elongate rostrum (length/width = 3.56–3.82 in *T. racupalpa*, 4.00–4.15 in *T. ivyae*) and less elongate pedipalpal tibiae (length/width = 4.44–5.00 in *T. racupalpa*, 5.07–5.64 in *T. ivyae*). Male *T. racupalpa* can be differentiated from male *T. ivyae* by having a shorter anterior venter (♂ 200–205 in *T. racupalpa*, 220–230 in *T. ivyae*) and a longer genital field (♂ 160–165 in *T. racupalpa*, 142–148 in *T. ivyae*).

######## Type deposition.

Holotype (♀) deposited in the CNC.

######## Description.

**Female (Figure 206)** (n = 3) (holotype measurements in parentheses when available) with characters of the genus with following specifications.

**Dorsum** — (570–630 (570) long; 450–540 (450) wide) ovoid with bluish-purple coloration separated into anterior and posterior portions with bold orange medially. Anterio-medial platelets (130–145 (130) long; 52.5–65 (52.5) wide). Anterio-lateral platelets (157.5–200 (157.5) long; 71.25–75 (71.25) wide) free from dorsal plate. Dgl-4 much closer to the muscle scars than to dorsum edge (distance between Dgl-4 170–205 (170)). Dorsal plate proportions: dorsum length/width 1.17–1.27 (1.27); dorsal width/distance between Dgl-4 2.59–2.77 (2.65); anterio-medial platelet length/width 2.19–2.48 (2.48); anterio-lateral platelet length/width 2.21–2.67 (2.21); anterio-lateral/anterio-medial length 1.21–1.40 (1.21).

**Gnathosoma — Subcapitulum** (330–375 (330) long (ventral); 251–297.5 (252) long (dorsal); 130–145 (130) tall) faint bluish-purple coloration. Rostrum (142.5–170 (142.5) long; 40–45 (40) wide) elongate. Chelicerae (319–375 (320) long) with curved fangs (50–60 (50) long). Subcapitular proportions: ventral length/height 2.48–2.73 (2.54); rostrum length/width 3.56–3.82 (3.56). **Pedipalps** elongate (especially tibia) with long tuberculate ventral extensions on femora and genua. Palpomeres: trochanter (45–45 (45) long); femur (125–145 (125) long); genu (67.5–75 (67.5) long); tibia (100–125 (100) long; 22.5–25 (22.5) wide); tarsus (17.5–20 (17.5) long). Palpomere proportions: femur/genu 1.85–1.93 (1.85); tibia/femur 0.80–0.86 (0.80); tibia length/width 4.44–5.00 (4.44).

**Venter** — (700–800 (700) long; 490–610 (491) wide) with bold bluish-purple coloration. Gnathosomal bay (152.5–185 (152.5) long; 80–87.5 (87.5) wide). Cxgl-4 subapical. **Medial suture** (17.5–20 (17.5) long). **Genital plates** (152.5–185 (152.5) long; 140–170 (140) wide). Additional measurements: Cx-1 (285–320 (286) long (total); 114–140 (115) long (medial)); Cx-3 (332–380 (332) wide); anterior venter (152.5–165 (152.5) long). Ventral proportions: gnathosomal bay length/width 1.74–2.31 (1.74); anterior venter/genital field length 0.89–1.00 (1.00); anterior venter length/genital field width 0.97–1.09 (1.09); anterior venter/medial suture 8.00–8.71 (8.71).

**Male (Figure 207)** (n = 2) (allotypic measurements in parentheses when available) with characters of the genus with following specifications.

**Dorsum** — (555–570 (570) long; 460–475 (475) wide) ovoid with bluish-purple coloration separated into anterior and posterior portions with bold orange medially. Anterio-medial platelets (130–135 (135) long; 57.5–65 (65) wide). Anterio-lateral platelets (182.5–190 (190) long; 70–80 (80) wide) free from dorsal plate. Dgl-4 much closer to the muscle scars than to dorsum edge (distance between Dgl-4 200–210 (200)). Dorsal plate proportions: dorsum length/width 1.20–1.21 (1.20); dorsal width/distance between Dgl-4 2.19–2.38 (2.38); anterio-medial platelet length/width 2.08–2.26 (2.08); anterio-lateral platelet length/width 2.38–2.61 (2.38); anterio-lateral/anterio-medial length 1.40–1.41 (1.41).

**Gnathosoma — Subcapitulum** (315–317.5 (317.5) long (ventral); 250–250 (250) long (dorsal); 110–115 (115) tall) faint bluish-purple coloration. Rostrum (155–155 (155) long; 40–40 (40) wide) elongate. Chelicerae (295–310 (310) long) with curved fangs (50–55 (50) long). Subcapitular proportions: ventral length/height 2.76–2.86 (2.76); rostrum length/width 3.88–3.88 (3.88). **Pedipalps** elongate (especially tibia) with long tuberculate ventral extensions on femora and genua. Palpomeres: trochanter (40–40 (40) long); femur (125–125 (125) long); genu (65–65 (65) long); tibia (107.5–110 (107.5) long; 20–20 (20) wide); tarsus (15–15 (15) long). Palpomere proportions: femur/genu 1.92–1.92 (1.92); tibia/femur 0.86–0.88 (0.86); tibia length/width 5.38–5.50 (5.38).

**Venter** — (695–710 (710) long; 500–505 (500) wide) with bold bluish-purple coloration. Gnathosomal bay (157.5–160 (157.5) long; 70–70 (70) wide). Cxgl-4 subapical. **Medial suture** (45–45 (45) long). **Genital plates** (160–165 (165) long; 125–125 (125) wide). Additional measurements: Cx-1 (300–305 (300) long (total); 145–145 (145) long (medial)); Cx-3 (340–345 (340) wide); anterior venter (200–205 (205) long). Ventral proportions: gnathosomal bay length/width 2.25–2.29 (2.25); anterior venter/genital field length 1.24–1.25 (1.24); anterior venter length/genital field width 1.60–1.64 (1.64); anterior venter/medial suture 4.44–4.56 (4.56).

**Immatures** unknown.

######## Etymology.

Specific epithet (*racupalpa*) refers to the spined, rake-like pedipalps which have long, thin tibiae and elongate tubercles (*racu*, Old English feminine, rake; *palpus*, L. hand, feeler).

######## Distribution.

**Figure 205.**
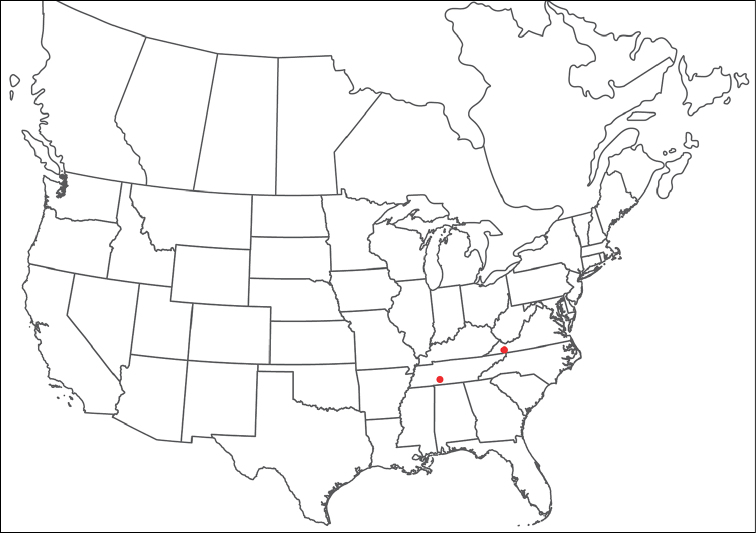
*Torrenticola racupalpa* sp. n. distribution.

Southeastern, Tennessee and Virginia (Figure 205).

**Figure 206.**
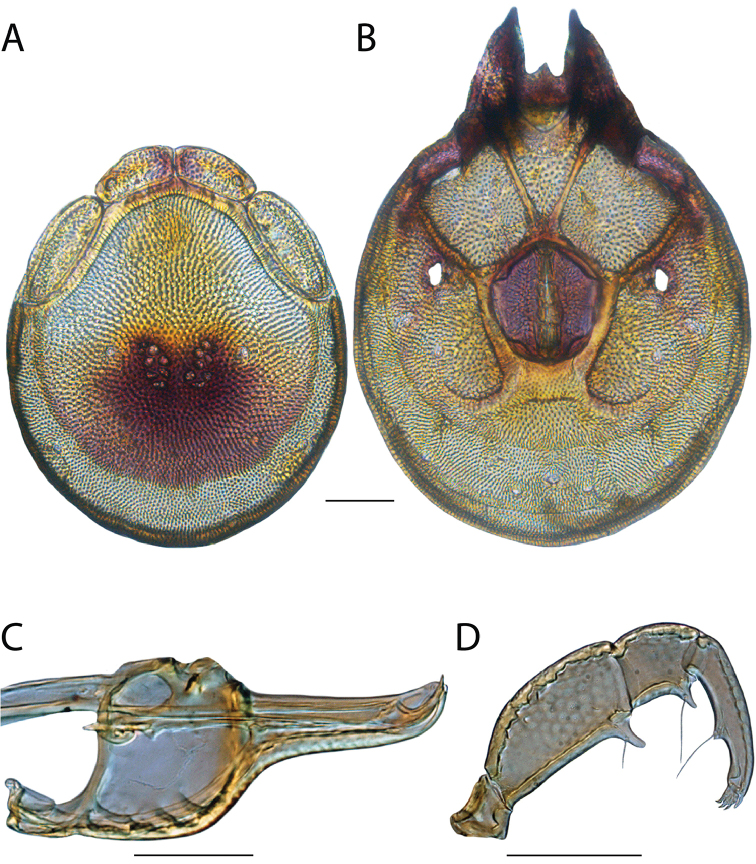
*Torrenticola racupalpa* sp. n. female: **A** dorsal plates **B** venter (legs removed) **C** subcapitulum **D** pedipalp (setae not accurately depicted). Scale = 100 µm.

**Figure 207.**
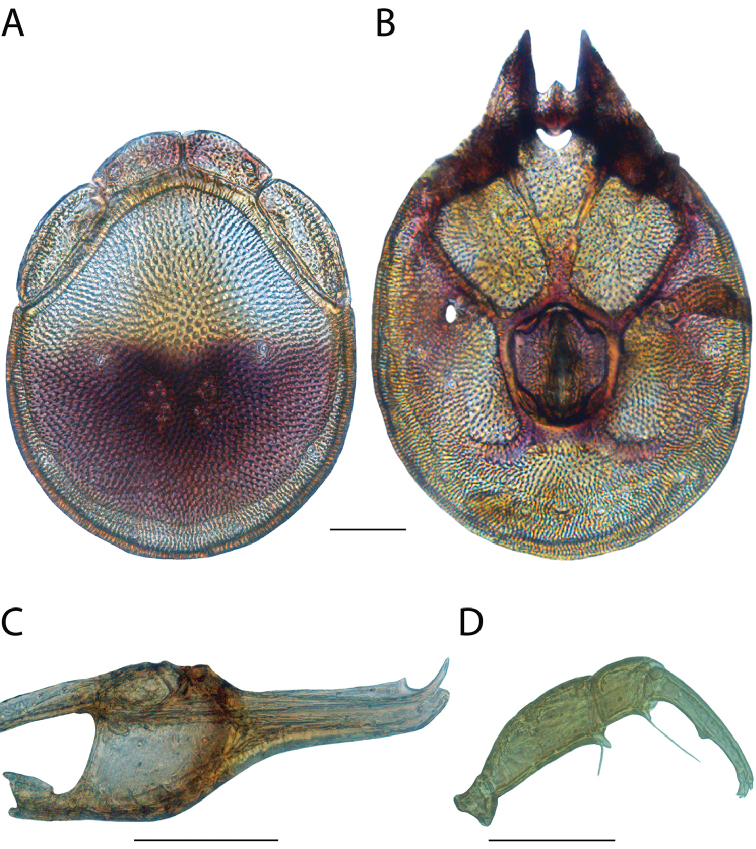
*Torrenticola racupalpa* sp. n. male: **A** dorsal plates **B** venter (legs removed) **C** subcapitulum **D** pedipalp (setae not accurately depicted). Scale = 100 µm.

######## Remarks.

In the COI analysis, *Torrenticola racupalpa* groups with other members of the Raptor Complex with high support. We were only able to acquire molecular data from one specimen, and we were unable to amplify 28S, which disabled us from examining the placement of this species in our combined analysis. In the COI analysis, *T. racupalpa* groups with *T. elusiva* with high confidence, and these species are greater than 4% different in COI sequence from each other. Based upon this information as well as morphology, we place this species in Raptor Identification Group.

This species hypothesis is supported by high divergence between species (3–15%), and by the morphological characters outlined in the diagnosis.

####### Torrenticola rala

Taxon classificationAnimaliaTrombidiformesTorrenticolidae

Cook, 1980

Torrenticola rala Cook, 1980: 394.

######## Material examined

**(11** ♀; **15** ♂) **. Arizona, USA**: 3 ♂ from Coconino County, Oak Creek Canyon, Oak Creek, beside Route 89A, between Banjo Bill & Bootlegger campgrounds, 21 Jul 1987, by IM Smith, IMS870100A • 1 ♂ and 1 ♂ from Cochise County, Chiricahua Mountains, Cave Creek Recreation Area, Cave Creek at John Hand Picnic Area, 15 Jul 1987, by IM Smith, IMS870092B • 1 ♀ from Cochise County, Chiricahua Mountains, Cave Creek Recreation Area, Cave Creek at Stewart campground, 16 Jul 1987, by IM Smith, IMS870094 • 1 ♀ from Cochise County, Chiricahua Mountains, Cave Creek, Herb Martyr Campground, Forest Road 42A 10 May 2012, by IM Smith, IMS120009 • 3 ♀ and 1 ♂ from Gila County, East Verde River, beside Route 87, north of Payson, 19 Jul 1987, by IM Smith, IMS870097 • 2 ♂ from Yavapai County, West Clear Creek at Clear Creek campground, off Forest Road 9, east of Camp Verde, 19-22 Jul 1987, by IM Smith, IMS870098 • **New Mexico, USA**: • 1 ♂ from Catron County, Glenwood Whitewater Creek at Whitewater Creek Picnic Area, 12 Jul 1987, by IM Smith, IMS870084 • 2 ♀ and 4 ♂ from Catron County, Little Creek, beside Route 15, 65 kilometers north of Route 180 in Silver City, 10 Jul 1987, by IM Smith, IMS870081A • **Texas, USA**: 1 ♀ and 1 ♂ from Bandera County, Lost Maples State Natural Area, Sabinal River (29°49'N, 99°34'W), 27 Sep 1995, by IM Smith, IMS950052 • 1 ♀ from Bandera County, Vanderpool, beside Route 187, Sabinal River (29°48'10"N, 99°34'30"W), 2 May 2009, by IM Smith, IMS090007 • 1 ♀ and 1 ♂ from Val Verde County, Bakers Crossing Campground, off Route 163, Devils River (29°58'N, 101°9'W), 5 Oct 1999, by IM Smith, IMS990061A • 1 ♀ from Uvalde County, Garner State Park, Frio River (29°35'22"N, 99°44'12"W), 28 May 1998, by IM Smith, IMS980027A.

######## Type deposition.

Holotype (♂) deposited in prep. no. DC 12–72 FMC. Allotype (♀) deposited in prep. no. DC 43–72 FMC.

######## Diagnosis.

*Torrenticola rala* are similar to other members of the Rala Group (*T. boettgeri*, *T. keesdavidsi*, *T. kurtvietsi*, *T. lamellipalpis*, *T. dolichodactyla*, and *T. anoplopalpa*) by being colorless, having incomplete hind coxal margins and being distributed in the southwest. *T. rala* can be differentiated from all other Rala Group by having a stockier rostrum (length/width = 1.63–1.83 in *T. rala*, 2.19–3.57 in others). Additionally, *T. rala* can be differentiated from all other Rala Group by having a shorter anterior venter (♀ = 170–185 in *T. rala* 192–260 in others ♂ = 200–225 in *T. rala*, 235–308 in others), except male *T. keesdavidsi* (♂ 225–260).

######## Re-description.

**Female (Figure 209)** (n = 5) (holotype measurements in parentheses when available) with characters of the genus with following specifications.

**Dorsum** — (630–700 long; 465–505 wide) ellipsoid and colorless. Anterio-medial platelets (150–162.5 long; 62.5–72.5 wide). Anterio-lateral platelets (172.5–195 long; 70–80 wide) free from dorsal plate. Dgl-4 much closer to the edge of the dorsum than to the muscle scars (distance between Dgl-4 370–420). Dorsal plate proportions: dorsum length/width 1.30–1.46; dorsal width/distance between Dgl-4 1.17–1.26; anterio-medial platelet length/width 2.14–2.41; anterio-lateral platelet length/width 2.38–2.79; anterio-lateral/anterio-medial length 1.15–1.26.

**Gnathosoma — Subcapitulum** (247.5–260 long (ventral); 160–170 long (dorsal); 115–120 tall) tall and colorless. Rostrum (79–89 long; 45–50 wide) short and conical. Chelicerae (217.5–247.5 long) with curved fangs (52.5–60 long). Subcapitular proportions: ventral length/height 2.06–2.17; rostrum length/width 1.70–1.83. **Pedipalps** short and stocky (especially tibiae) with tuberculate ventral extensions on femora and ventral extensions on genua absent. Palpomeres: trochanter (35–37.5 long); femur (70–77.5 long); genu (50–57.5 long); tibia (55–62.5 long; 20–22.5 wide); tarsus (15–17.5 long). Palpomere proportions: femur/genu 1.35–1.43; tibia/femur 0.79–0.81; tibia length/width 2.75–2.88.

**Venter** — (760–840 long; 527–585 wide) colorless. Gnathosomal bay (150–162.5 long; 55–62.5 wide). Cxgl-4 apical. **Medial suture** (25–35 long). **Genital plates** (175–190 long; 155–170 wide). Additional measurements: Cx-1 (280–300 long (total); 130–145 long (medial)); Cx-3 (345–380 wide); anterior venter (170–185 long). Ventral proportions: gnathosomal bay length/width 2.58–2.73; anterior venter/genital field length 0.92–1.01; anterior venter length/genital field width 1.00–1.19; anterior venter/medial suture 5.00–6.80.

**Male** (**Figure 210**) (n = 5) (allotypic measurements in parentheses when available) with characters of the genus with following specifications.

**Dorsum** — (600–655 long; 435–480 wide) ellipsoid and colorless. Anterio-medial platelets (135–165 long; 55–67.5 wide). Anterio-lateral platelets (177.5–190 long; 64–82.5 wide) free from dorsal plate. Dgl-4 much closer to the edge of the dorsum than to the muscle scars (distance between Dgl-4 375–400). Dorsal plate proportions: dorsum length/width 1.35–1.42; dorsal width/distance between Dgl-4 1.16–1.20; anterio-medial platelet length/width 2.40–2.48; anterio-lateral platelet length/width 2.30–2.78; anterio-lateral/anterio-medial length 1.15–1.31.

**Gnathosoma — Subcapitulum** (230–249 long (ventral); 152.5–162.5 long (dorsal); 107.5–112.5 tall) tall and colorless. Rostrum (77.5–81 long; 45–47.5 wide) short and conical. Chelicerae (215–232.5 long) with curved fangs (42.5–50 long). Subcapitular proportions: ventral length/height 2.14–2.21; rostrum length/width 1.63–1.72. **Pedipalps** short and stocky (especially tibiae) with tuberculate ventral extensions on femora and ventral extensions on genua absent. Palpomeres: trochanter (32.5–37.5 long); femur (65–72.5 long); genu (49–52.5 long); tibia (55–62.5 long; 20–21 wide); tarsus (15–17.5 long). Palpomere proportions: femur/genu 1.33–1.45; tibia/femur 0.79–0.89; tibia length/width 2.71–2.94.

**Venter** — (710–765 long; 500–540 wide) colorless. Gnathosomal bay (135–145 long; 55–62.5 wide). Cxgl-4 apical. **Medial suture** (65–80 long). **Genital plates** (160–185 long; 127.5–135 wide). Additional measurements: Cx-1 (270–295 long (total); 125–145 long (medial)); Cx-3 (337.5–375 wide); anterior venter (200–225 long). Ventral proportions: gnathosomal bay length/width 2.20–2.45; anterior venter/genital field length 1.14–1.33; anterior venter length/genital field width 1.57–1.67; anterior venter/medial suture 2.66–3.31.

**Immatures** unknown.

######## Etymology.

Cook (1980) did not specify an etymology for this specific epithet (*rala*) and we are unable to offer helpful speculation.

######## Distribution.

**Figure 208.**
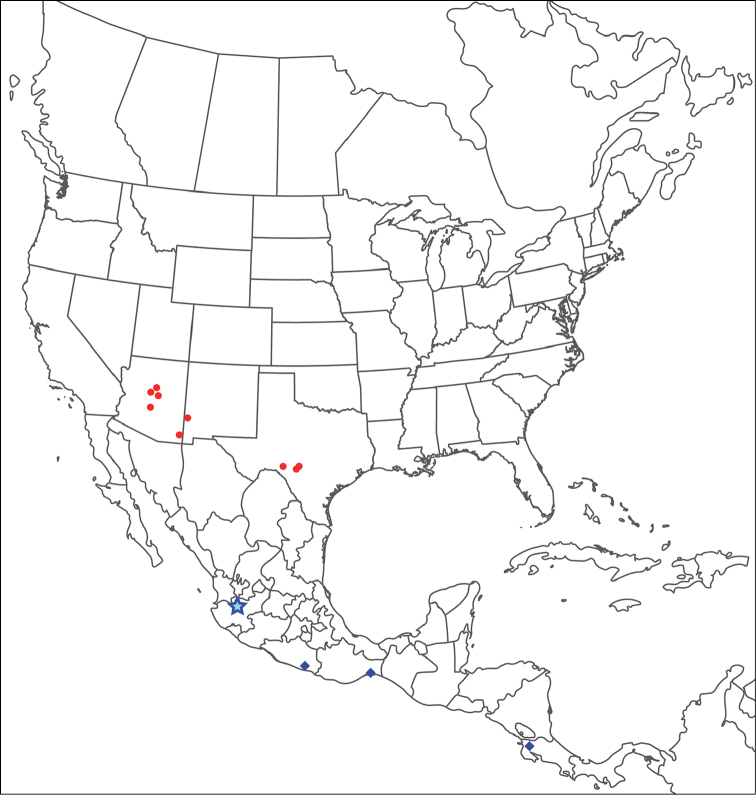
*Torrenticola rala* distribution. Red dots represent material examined; blue diamonds represent previous records (Cook 1980); blue star represents holotype locality (Cook 1980).

Southwestern U.S. (Arizona, New Mexico, Texas) and southward through Central America (Figure 208).

**Figure 209.**
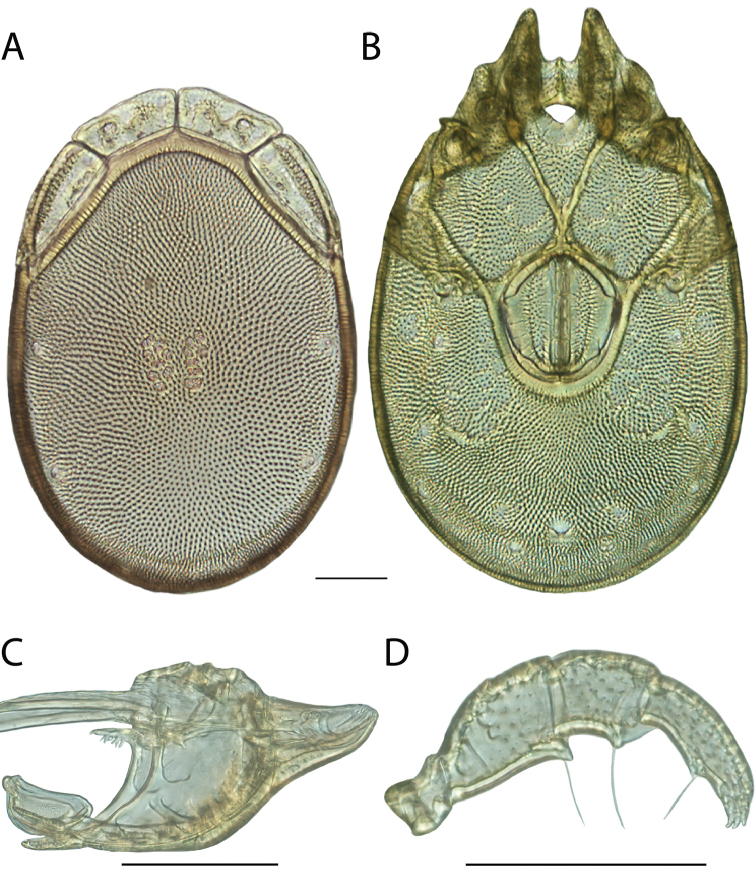
*Torrenticola rala* female: **A** dorsal plates **B** venter (legs removed) **C** subcapitulum **D** pedipalp (setae not accurately depicted). Scale = 100 µm.

**Figure 210.**
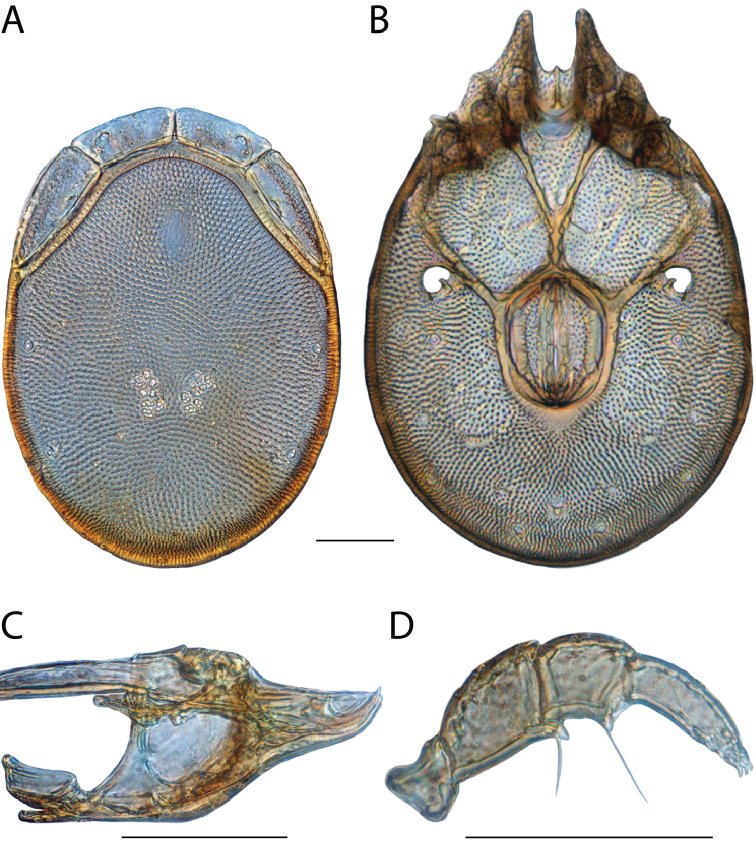
*Torrenticola rala* male: **A** dorsal plates **B** venter (legs removed) **C** subcapitulum **D** pedipalp (setae not accurately depicted). Scale = 100 µm.

######## Remarks.

Our analyses were unable to confidently place *Torrenticola rala* phylogenetically. The COI analyses recovers this species at the base of the Raptor Complex, but this relationship was not recovered in the combined analysis. Because of this ambiguity, we refrain from placing this species in a species complex. However, based upon the morphological similarity that this species shares with six other species, we place this species within the Rala Identification Group.

*T. rala* is greater than 13% different in COI sequence from sister species. This species hypothesis is supported by high divergence between species (3–15%) and by the morphological characters outlined in the diagnosis.

####### Torrenticola raptor

Taxon classificationAnimaliaTrombidiformesTorrenticolidae

Fisher & Dowling sp. n.

http://zoobank.org/29F4761F-CC8C-404C-9679-A85A0EB59EF0

######## Material examined.

HOLOTYPE (♀): from USA, Ontario, Hastings County, Maple Leaf, Papineau Creek, beside Highway 62, 18 Aug 2011, by IM Smith, IMS110054, DNA 2864.

PARATYPES (58 ♀; 40 ♂): **Alabama, USA**: 3 ♀ and 3 ♂ from Clay County, Talladega Creek, beside Forest Route 649, 0.8 kilometers northeast of road from Forest Route 600 to Campbell Springs, 2 Jul 1990, by IM Smith, IMS900075A • **Maine, USA**: 1 ♀ from Aroostook County, Ashland, beside Route 11 at bridge, Aroostook River (46°38'N, 68°24'W), 4 Jul 1989, by IM Smith, IMS890067 • 1 ♀ from Franklin County, Smalls Falls Picnic Area, beside Route 4, Sandy River (44°52'N, 70°31'W), 5 Jul 1989, by IM Smith, IMS890069 • 2 ♀ from Washington County, Old Stream, off Route 9, 5.5 km west of Route 192, 6 Jun 2012, by IM Smith, IMS120012 • **New Brunswick, Canada**: 2 ♀ and 1 ♂ from Charlotte County, Rollingham, Digdeguash River, beside Highway 770 at covered bridge, 30 Jun 1989, by IM Smith, IMS890053 • 2 ♀ and 1 ♂ from Charlotte County, Rollingham, Digdegaush River, beside Highway 770, 3 Oct 2011, by IM Smith, IMS110118 • 1 ♂ from Northumberland County, Renous River, beside Highway 108, 18 Jul 1980, by IM Smith, IMS800111 • 1 ♀ and 1 ♂ from Restigouche County, Mt. Carleton Provincial Park, Nictau River, 16 Jul 1980, by IM Smith, IMS800109 • 1 ♀ and 1 ♂ from York County, Magaguadavic River, beside Highway 3, just east of Thomaston Corners, 1 Jul 1989, by IM Smith, IMS890055A • 1 ♂ from York County, Napadogan Brook, beside Road J-19, 6.3 kilometers north of Nashwaak Exper. Watershed headquarters, 23 Jul 1981, by IM Smith, IMS810095B • 1 ♀ from York County, Nashwaak Exper. Watershed, Nashwaak River, at trunk road, 20 Jul 1980, by IM Smith, IMS800118A • **New Hampshire, USA**: 1 ♀ from Coos County, picnic area beside Route 110, Ammonoosuc River (44°36'N, 71°24'W), 5 Jul 1989, by IM Smith, IMS890071 • **New Jersey, USA**: 1 ♀ and 1 ♂ from Sussex County, Big Flat Brook beside Flatbrook Road, 2.6 kilometers north of Route 206 at Tuttles Corner, 23 Jun 1990, by IM Smith, IMS900053 • **New York, USA**: 2 ♀ from Essex County, Minerva, Boreas River, beside Route 28N, 13.8 kilometers northwest of Morse Memorial Parkway, 21 Jun 1990, by IM Smith, IMS900050A • 1 ♀ and 1 ♂ from Greene County, Schoharie Creek, beside Route 23A, 9.6 kilometers west of Route 296 in Hunter, 22 Jun 1990, by IM Smith, IMS900052 • 1 ♀ from Ulster County, beside Route 28, 1.6 kilometers south of Mt. Tremper, 17 Aug 1964, by DR Cook, DRC640023 • 2 ♀ and 1 ♂ from Warren County, East Branch of Sacandaga River, beside Route 8, 14.5 kilometers east of Route 30, 21 Jun 1990, by IM Smith, IMS900051A • **North Carolina, USA**: 2 ♀ and 2 ♂ from Yancey County, Lost Cove Picnic Area, South Toe River, on Forest Route 472, 2.8 kilometers south of Route 80, 28 Jun 1990, by IM Smith, IMS900065A • **Nova Scotia, Canada**: 1 ♀ from Inervess County, Cape Brenton Highlands National Park, Cheticamp entrance, pond near Salmon Pools Trailhead, 10 Sep 2011, by IM Smith, IMS110072 • 1 ♀ from Inervess County, Inervess, Cheticamp River, 10 Sep 2011, by IM Smith, IMS110071 • 1 ♀ and 1 ♂ from Luneburg County, New Germany, LaHave River, beside Highway 10, 23 Sep 2011, by IM Smith, IMS110098 • 2 ♀ and 1 ♂ from Victoria County, Cape Brenton Island, Baddeck River, beside road to Baddeck Forks, 18 Jul 1981, by IM Smith, IMS810082 • **Ontario, Canada**: 1 ♂ from Cochrane County, Hearst, Pitopiko River, beside Highway 11, 24 Jul 1975, by IM Smith & D Spaner, IMS750120 • 3 ♀ and 3 ♂ from Hastings County, Madawaska, Opeongo River, beside Highway 60, 29 Aug 1981, by IM Smith, IMS810033A • 2 ♀ and 2 ♂ from Hastings County, Maple Leaf, Papineau Creek, 10-11 Jun 1981, by IM Smith, IMS810014A • 1 ♂ (ALLOTYPE) from Hastings County, Maple Leaf, Papineau Creek, east of Davis Road before Highway 62, 18 Aug 2011, by IM Smith, IMS110053, DNA 1257 • 2 ♀ and 1 ♂ from Hastings County, Maple Leaf, Papineau Creek, east of Davis Road before Highway 62, 18 Aug 2011, by IM Smith, IMS110053 • 1 ♀ from Hastings County, Maple Leaf, Papineau Creek, beside Highway 62, 18 Aug 2011, by IM Smith, IMS110054 • 1 ♀ from Kenora County, Ignace, beside Highway 599, 4.2 kilometers north of Highway 17, 31 Jul 1975, by IM Smith, IMS750216 • 2 ♀ and 2 ♂ from Kenora County, Revell River, beside Highway 17, 40.2 kilometers east of Highway 72, 3 Jun 1980, by IM Smith, IMS800052 • 2 ♀ and 2 ♂ from Muskoka County, Huntsville, East River, Xing road to Dyer Memorial, 26 Aug 1981, by IM Smith, IMS810032A • 1 ♀ and 1 ♂ from Lanark County, Mississippi River, beside Lanark Road #12, between Lanark & Fallbrook, 6 Oct 1983, by IM Smith & CJ Hill, IMS830094A • 1 ♂ from Nipissing County, Algonquin Provincial Park, Madawaska River, at Highway 60, near Lake of Two Rivers, 15 May 1980, by IM Smith & CJ Hill, IMS800004C • 1 ♀ and 1 ♂ from Nipissing County, Aumond Creek, beside Highway 17, east of Mattawa, 30 Aug 1983, by IM Smith & CJ Hill, IMS830079A • 1 ♀ and 1 ♂ from Nipissing County, Aumond Creek, beside Highway 17, east of Mattawa, 30 Aug 1983, by IM Smith & CJ Hill, IMS830079B • 1 ♀ from Nipissing County, Bastien Creek, at picnic area, beside Highway 17, east of Mattawa, 30 Aug 1983, by IM Smith & CJ Hill, IMS830076A • 1 ♀ and 1 ♂ from Peterborough County, Apsley, Eels Creek, crossing Highway 28, just south of Eels Lake, 13 Jun 1981, by IM Smith, IMS810017A • 1 ♀ and 1 ♂ from Peterborough County, Eels Creek, crossing Highway 28 at picnic area, just north of Woodview, 13 Jun 1981, by IM Smith, IMS810018A • 1 ♂ from Thunder Bay County, west of Geraldton, Creelman Creek, beside Highway 11, 26 Jul 1975, by IM Smith & D Spaner, IMS750147 • **Quebec, Canada**: 1 ♀ from Gatineau County, Gatineau Park stream crossing, Gatineau Parkway at Meech Lake Road, 15 Sep 1981, by IM Smith & C Cramer, IMS810034A • 1 ♀ from Gatineau County, beside Gatineau Parkway, at Meech Lake Road, 13 Aug 1982, by IM Smith & CJ Hill, IMS820001A • 1 ♂ from Gatineau County, beside Gatineau Parkway, at Meech Lake Road, 13 Aug 1982, by IM Smith & CJ Hill, IMS820001B • 1 ♀ and 1 ♂ from Pontiac County, beside road at east end of Thorne Lake (45°32'N, 76°0'W), 1 May 1986, by IM Smith & CJ Hill, IMS860002A • **South Carolina, USA**: 1 ♀ from Greenville County, Matthews Creek, 24 Apr 2014, by D Eargle, JRF 14-0424-001 • **Tennessee, USA**: 1 ♀ and 1 ♂ from Monroe County, Tellico River, beside Forest Route 210, 1.8 kilometers east of bridge at Bald River Falls, 5 Jul 1990, by IM Smith, IMS900079 • 1 ♀ from Monroe County, Tellico River (35°20'27"N, 84°11'31"W), 12 Sep 2009, by IM Smith, IMS090111 • 1 ♀ from Sevier County, Great Smokey Mountians National Park, Little River (35°40'56"N, 83°39'2"W), 8 Sep 2009, by IM Smith, IMS090103 • 2 ♀ from Sevier County, Great Smokey Mountianss National Park, Laurel Creek (35°39'7"N, 83°42'32"W), 17 Sep 2010, by IM Smith, IMS100145 • **West Virginia, USA**: 1 ♀ and 1 ♂ from Pocahontas County, Island Campground, East Fork of Greenbrier River, beside Route 28, northeast of Thornwood, 16 Jul 1990, by IM Smith, IMS900101A • 1 ♀ and 1 ♂ from Randolph County, Laurel Fork Campground, Laurel Fork of Cheat River, off Forest Route 14, south of Wymer, 17 Jul 1990, by IM Smith, IMS900102.

######## Type deposition.

Holotype (♀), allotype (♂), and most paratypes (53 ♀; 34 ♂) deposited in the CNC; other paratypes (5 ♀; 5 ♂) deposited in ACUA.

######## Diagnosis.

*Torrenticola raptor* are similar to other members of the Raptor Group (*T. gnoma*, *T. irapalpa*, *T. longitibia*, *T. mjolniri*, *T. elusiva*, *T. racupalpa*, *T. danielleae*, *T. daemon*, and *T. ivyae*) in having round bodies; Dgl-4 close to muscles scars; long, thin subcapitular rostra; and long, thin pedipalp tibiae. *T. raptor* can be differentiated from all other members of the Raptor Group by having a more elongate subcapitulum (ventral length/height ♀ = 2.98–3.18 in *T. raptor*, 2.26–2.90 in others; ♂ = 3.13–3.27 in *T. raptor*, 2.29–3.00 in others). *T. raptor* can be further differentiated from *T. elusiva*, *T. irapalpa*, *T. gnoma*, *T. danielleae*, *T. daemon*, and *T. ivyae* by having more elongate pedipalp tibiae (length/width ♀ = 6.00–7.54 in *T. raptor*, 4.09–5.67 in others; ♂ = 5.29–5.63 in *T. raptor*, 3.88–5.20 in others). *T. raptor* can be further differentiated from *T. mjolniri*, *T. longitibia*, *T. gnoma*, *T. elusiva*, *T. racupalpa*, and *T. ivyae* by having Dgl-4 closer to the dorsal edge (dorsal width/distance between Dgl-4 = 1.66–2.02 in *T. raptor*, 2.06–3.29 in others).

######## Description.

**Female (Figure 212)** (n = 13) (holotype measurements in parentheses when available) with characters of the genus with following specifications.

**Dorsum** — (570–660 (625) long; 465–550 (505) wide) circular with coloration posteriorly extending in a strip anteriorly to the edge of the dorsal plate (rarely without anterior extension), coloration variable from navy blue to purple to reddish purple. Anterio-medial platelets (122.5–150 (135) long; 60–80 (75) wide). Anterio-lateral platelets (182.5–210 (195) long; 75–90 (80) wide) free from dorsal plate. Dgl-4 closer to the muscle scars than to the dorsum edge (distance between Dgl-4 230–295 (275)). Dorsal plate proportions: dorsum length/width 1.19–1.31 (1.24); dorsal width/distance between Dgl-4 1.80–2.02 (1.84); anterio-medial platelet length/width 1.75–2.07 (1.80); anterio-lateral platelet length/width 2.28–2.49 (2.44); anterio-lateral/anterio-medial length 1.39–1.57 (1.44).

**Gnathosoma — Subcapitulum** (335–380 (365) long (ventral); 249–283 (270) long (dorsal); 107.5–125 (120) tall) colorless. Rostrum (150–167.5 (160) long; 37.5–45 (37.5) wide) elongate. Chelicerae (316–368 (355) long) with curved fangs (43–60 (60) long). Subcapitular proportions: ventral length/height 2.98–3.18 (3.04); rostrum length/width 3.44–4.40 (4.27). **Pedipalps** elongate (especially tibiae) with long tuberculate ventral extensions on femora and genua. Palpomeres: trochanter (37.5–42.5 (42.5) long); femur (122.5–143.75 (137.5) long); genu (65–77.5 (70) long); tibia (112.5–142.5 (122.5) long; 16.25–20 (18.75) wide); tarsus (17.5–22.5 (22.5) long). Palpomere proportions: femur/genu 1.68–1.96 (1.96); tibia/femur 0.82–1.03 (0.89); tibia length/width 6.00–7.54 (6.53).

**Venter** — (720–830 (800) long; 535–609 (550) wide) with faint navy blue to purple coloration or colorless. Gnathosomal bay (135–192.5 (172.5) long; 62.5–85 (75) wide). Cxgl-4 far from apex. **Medial suture** (35–45 (45) long). **Genital plates** (157.5–182.5 (170) long; 135–153.75 (145) wide). Additional measurements: Cx-1 (271–330 (330) long (total); 92–160 (160) long (medial)); Cx-3 (336–373 (340) wide); anterior venter (205–240 (220) long). Ventral proportions: gnathosomal bay length/width 1.74–2.48 (2.30); anterior venter/genital field length 1.23–1.47 (1.29); anterior venter length/genital field width 1.41–1.65 (1.52); anterior venter/medial suture 4.56–6.29 (4.89).

**Male (Figure 213)** (n = 5) (allotypic measurements in parentheses when available) with characters of the genus with following specifications.

**Dorsum** — (540–610 (540) long; 415–450 (440) wide) circular with coloration posteriorly extending in a strip anteriorly to the edge of the dorsal plate (rarely without anterior extension), coloration variable from navy blue to purple to reddish purple. Anterio-medial platelets (126.25–132.5 (127.5) long; 57.5–65 (57.5) wide). Anterio-lateral platelets (177.5–190 (177.5) long; 68–80 (75) wide) free from dorsal plate. Dgl-4 closer to the muscle scars than to the dorsum edge (distance between Dgl-4 230–265 (230)). Dorsal plate proportions: dorsum length/width 1.23–1.36 (1.23); dorsal width/distance between Dgl-4 1.66–1.91 (1.91); anterio-medial platelet length/width 1.94–2.22 (2.22); anterio-lateral platelet length/width 2.34–2.58 (2.37); anterio-lateral/anterio-medial length 1.39–1.47 (1.39).

**Gnathosoma — Subcapitulum** (297.5–327.5 (297.5) long (ventral); 220–247 (221) long (dorsal); 95–102.5 (95) tall) colorless. Rostrum (131.25–147.5 (131.25) long; 30–35 (30) wide) elongate. Chelicerae (274–306 (274) long) with curved fangs (44–54 (45) long). Subcapitular proportions: ventral length/height 3.13–3.27 (3.13); rostrum length/width 3.89–4.38 (4.38). **Pedipalps** elongate (especially tibiae) with long tuberculate ventral extensions on femora and genua. Palpomeres: trochanter (37.5–40 (40) long); femur (111.25–122.5 (111.25) long); genu (62.5–65 (62.5) long); tibia (102.5–112.5 (102.5) long; 18.75–21.25 (18.75) wide); tarsus (17.5–20 (20) long). Palpomere proportions: femur/genu 1.73–1.88 (1.78); tibia/femur 0.89–0.99 (0.92); tibia length/width 5.29–5.63 (5.47).

**Venter** — (680–790 (680) long; 457–523 (458) wide) with faint navy blue to purple coloration or colorless. Gnathosomal bay (132.5–145 (132.5) long; 52.5–77.5 (52.5) wide). Cxgl-4 far from apex. **Medial suture** (80–107.5 (80) long). **Genital plates** (137.5–152.5 (141.25) long; 110–125 (120) wide). Additional measurements: Cx-1 (268–322 (269) long (total); 112–160 (113) long (medial)); Cx-3 (332–347 (335) wide); anterior venter (245–305 (245) long). Ventral proportions: gnathosomal bay length/width 1.81–2.52 (2.52); anterior venter/genital field length 1.73–2.11 (1.73); anterior venter length/genital field width 2.04–2.60 (2.04); anterior venter/medial suture 2.84–3.06 (3.06).

**Immatures** unknown.

######## Etymology.

Specific epithet (*raptor*) refers to the long, thin pedipalps (especially tibiae) of this species which, combined with pronounced tubercles, appear especially capable of grasping slippery prey (*rapio L.* to seize; known in apposition).

######## Distribution.

**Figure 211.**
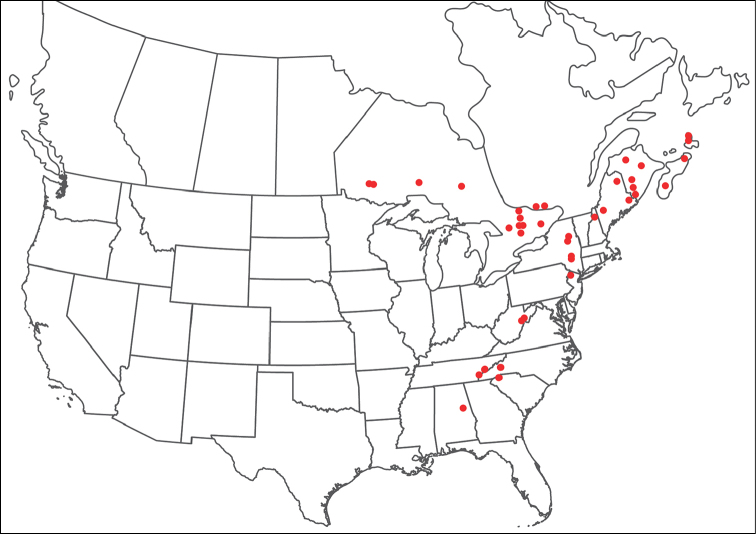
*Torrenticola raptor* sp. n. distribution.

Northeastern, but extending southward in the Appalachians (Figure 211).

**Figure 212.**
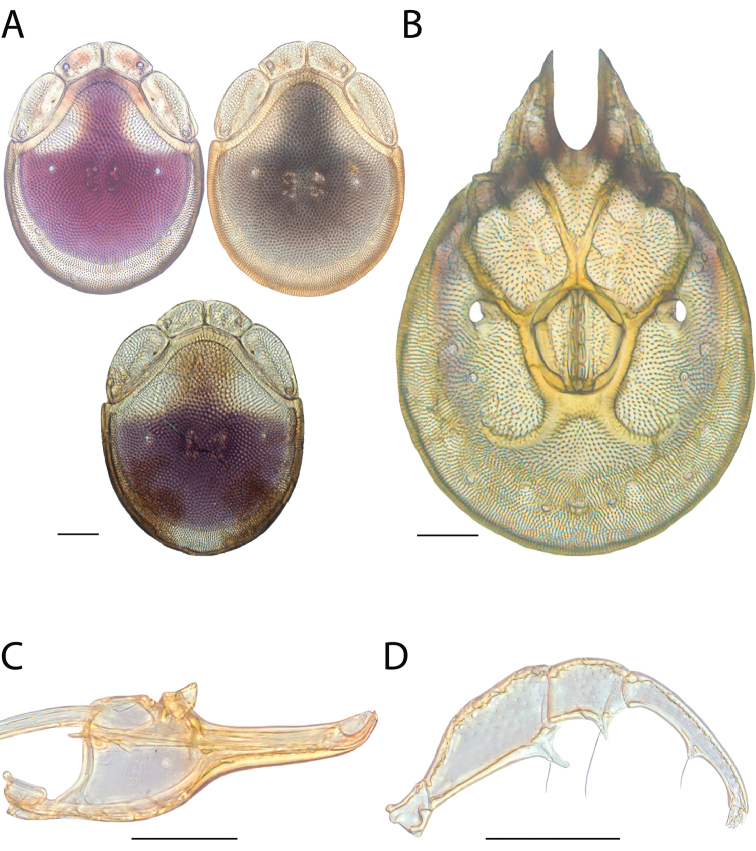
*Torrenticola raptor* sp. n. female: **A** dorsal plates, note color variation **B** venter (legs removed) **C** subcapitulum **D** pedipalp (setae not accurately depicted). Scale = 100 µm.

**Figure 213.**
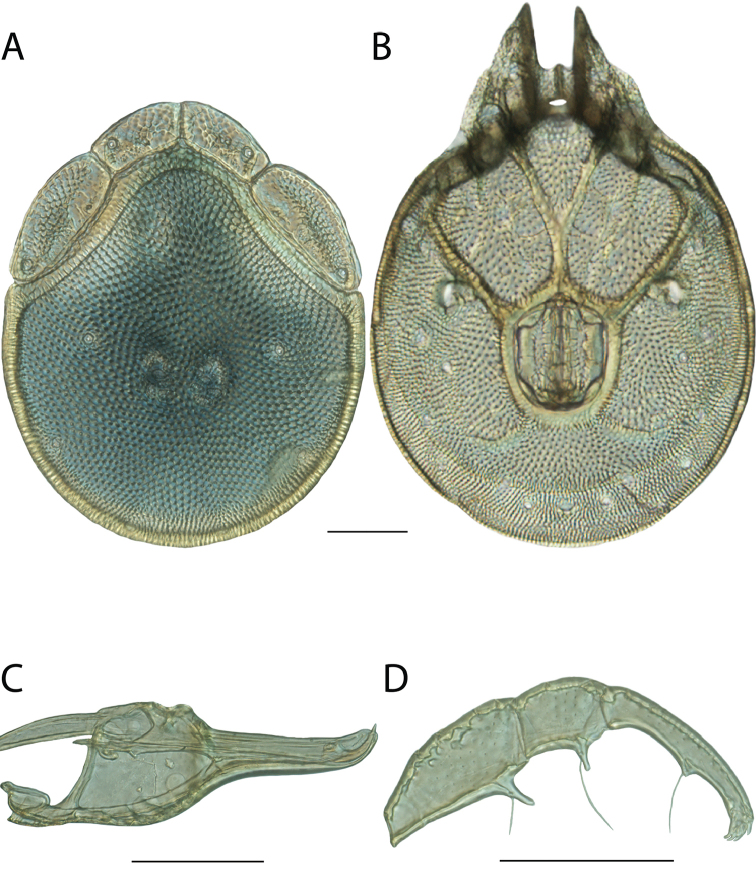
*Torrenticola raptor* sp. n. male: **A** dorsal plates **B** venter (legs removed) **C** subcapitulum **D** pedipalp (setae not accurately depicted). Scale = 100 µm.

######## Remarks.

*Torrenticola raptor* groups with other members of the Raptor Complex with high support and specimens of this species are less than 4% different in COI sequence from each other. This is higher sequence variability than in many species hypotheses presented herein. However, given the topology in the COI tree (Figure 7) and morphological similarity, it seems apparent that the variability represents a continuum across a large distribution rather than isolated species, so we consider these specimens to be within the same species hypothesis.

The position of *T. raptor* varied with analysis and was not well-supported in the combined analysis, so we are unable to comment on its affinities. However, morphological similarity is consistent with placing this species in the Raptor Identification Group.

This species hypothesis is supported by high divergence between species (3–15%), and by the morphological characters outlined in the diagnosis.

####### Torrenticola raptoroides

Taxon classificationAnimaliaTrombidiformesTorrenticolidae

Fisher & Dowling sp. n.

http://zoobank.org/92C6BF5F-BC02-4D5E-ABFD-140F2247D59F

######## Material examined.

HOLOTYPE (♀): from USA, New Mexico, Catron County, Little Creek, Gila Hot Springs, Little Creek Recreation Area off Route 15, 6 May 2012, by IM Smith, IMS120006, DNA 2895.

PARATYPES (8 ♀; 8 ♂): **Arizona, USA**: 2 ♀ and 2 ♂ from Coconino County, Oak Creek Canyon, Oak Creek, beside Route 89A, between Banjo Bill & Bootlegger campgrounds, 21 Jul 1987, by IM Smith, IMS870100B • 1 ♀ from Coconino County, Oak Creek Canyon, Oak Creek, beside Route 89A, just north of Pine Flat campground, 21 Jul 1987, by IM Smith, IMS870099B • 1 ♀ and 1 ♂ from Yavapai County, West Clear Creek at Clear creek campground, off Forest Road 9, east of Camp Verde, 22 Jul 1987, by IM Smith, IMS870102 • **New Mexico, USA**: 1 ♂ (ALLOTYPE) from Grant County, East Fork Gila River, Grapevine Recreation Area off Route 15, north of Silver City, 5 May 2012, by IM Smith, IMS120007, DNA 2900 • 1 ♂ from Catron County, Gila River, beside Route 15, just below mouth of Little Creek, 11 Jul 1987, by IM Smith, IMS870083A • 2 ♀ and 1 ♂ from Catron County, Little Creek, Gila Hot Springs, Little Creek Recreation Area off Route 15, 6 May 2012, by IM Smith, IMS120006 • 2 ♀ and 2 ♂ from Catron County, Little Creek, beside Route 15, 65 kilometers north of Route 180 in Silver City, 10 Jul 1987, by IM Smith, IMS870081A.

######## Type deposition.

Holotype (♀), allotype (♂), and most paratypes (5 ♀; 5 ♂) deposited in the CNC; other paratypes (3 ♀; 3 ♂) deposited in ACUA.

######## Diagnosis.

*Torrenticola raptoroides* are unlike all other western species in having round bodies with dorsal coloration restricted posteriorly and long, thin pedipalp tibiae. Additionally, they are only known from Catron & Grant Counties, New Mexico.

######## Description.

**Female (Figure 215)** (n = 5) (holotype measurements in parentheses when available) with characters of the genus with following specifications.

**Dorsum** — (610–690 (650) long; 485–530 (520) wide) ovoid with navy blue coloration separated into anterior and posterior portions with faint orange medially. Anterio-medial platelets (140–167.5 (157.5) long; 56–67.5 (61.25) wide). Anterio-lateral platelets (165–200 (195) long; 72.5–82.5 (75) wide) free from dorsal plate. Dgl-4 much closer to the edge of the dorsum than to the muscle scars (distance between Dgl-4 295–380 (380)). Dorsal plate proportions: dorsum length/width 1.19–1.30 (1.25); dorsal width/distance between Dgl-4 1.37–1.64 (1.37); anterio-medial platelet length/width 2.36–2.60 (2.57); anterio-lateral platelet length/width 2.27–2.60 (2.60); anterio-lateral/anterio-medial length 1.15–1.24 (1.24).

**Gnathosoma — Subcapitulum** (315–335 (330) long (ventral); 240–250 (249) long (dorsal); 135–140 (137.5) tall) colorless. Rostrum (135–142.5 (142.5) long; 40–45 (42.5) wide). Chelicerae (335–360 (345) long) with curved fangs (62–69 (63) long). Subcapitular proportions: ventral length/height 2.25–2.48 (2.40); rostrum length/width 3.17–3.38 (3.35). **Pedipalps** elongate (especially tibiae) with tuberculate ventral extensions on femora and genua. Palpomeres: trochanter (40–47.5 (45) long); femur (120–131.25 (131.25) long); genu (63.75–75 (72.5) long); tibia (100–112.5 (112.5) long; 21.25–22.5 (22.5) wide); tarsus (15–17.5 (17.5) long). Palpomere proportions: femur/genu 1.60–1.88 (1.81); tibia/femur 0.79–0.94 (0.86); tibia length/width 4.44–5.00 (5.00).

**Venter** — (710–805 (805) long; 562–600 (562) wide) colorless. Gnathosomal bay (167.5–182.5 (180) long; 72.5–100 (82.5) wide). Cxgl-4 far from apex. **Medial suture** (10–17.5 (12.5) long). **Genital plates** (172.5–182.5 (172.5) long; 167.5–177.5 (167.5) wide). Additional measurements: Cx-1 (300–311 (311) long (total); 111–140 (112) long (medial)); Cx-3 (350–410 (373) wide); anterior venter (150–187.5 (170) long). Ventral proportions: gnathosomal bay length/width 1.83–2.41 (2.18); anterior venter/genital field length 0.86–1.04 (0.99); anterior venter length/genital field width 0.88–1.07 (1.01); anterior venter/medial suture 10.71–16.75 (13.60).

**Male (Figure 216)** (n = 5) (allotypic measurements in parentheses when available) with characters of the genus with following specifications.

**Dorsum** — (530–605 (590) long; 415–500 (455) wide) ovoid with navy blue coloration separated into anterior and posterior portions with faint orange medially. Anterio-medial platelets (130–147.5 (140) long; 50–62.5 (62.5) wide). Anterio-lateral platelets (170–192.5 (177.5) long; 60–77.5 (75) wide) free from dorsal plate. Dgl-4 much closer to the edge of the dorsum than to the muscle scars (distance between Dgl-4 290–365 (320)). Dorsal plate proportions: dorsum length/width 1.21–1.30 (1.30); dorsal width/distance between Dgl-4 1.34–1.43 (1.42); anterio-medial platelet length/width 2.24–2.60 (2.24); anterio-lateral platelet length/width 2.33–2.83 (2.37); anterio-lateral/anterio-medial length 1.25–1.33 (1.27).

**Gnathosoma — Subcapitulum** (260–300 (290) long (ventral); 197.5–225 (215) long (dorsal); 105–122.5 (112.5) tall) colorless. Rostrum (112.5–127.5 (122.5) long; 37.5–42.5 (40) wide). Chelicerae (255–295 (282) long) with curved fangs (45–64 (64) long). Subcapitular proportions: ventral length/height 2.40–2.58 (2.58); rostrum length/width 2.94–3.06 (3.06). **Pedipalps** elongate (especially tibiae) with tuberculate ventral extensions on femora and genua. Palpomeres: trochanter (35–42.5 (40) long); femur (85–113.75 (113.75) long); genu (55–67.5 (67.5) long); tibia (85–107.5 (102.5) long; 20–22.5 (22.5) wide); tarsus (15–17.5 (17.5) long). Palpomere proportions: femur/genu 1.55–1.69 (1.69); tibia/femur 0.90–1.00 (0.90); tibia length/width 4.25–4.78 (4.56).

**Venter** — (630–710 (710) long; 480–580 (498) wide) colorless. Gnathosomal bay (135–162.5 (157.5) long; 60–72.5 (70) wide). Cxgl-4 far from apex. **Medial suture** (60–70 (62.5) long). **Genital plates** (135–155 (147.5) long; 120–130 (127.5) wide). Additional measurements: Cx-1 (260–300 (280) long (total); 130–155 (131) long (medial)); Cx-3 (330–375 (340) wide); anterior venter (195–235 (227.5) long). Ventral proportions: gnathosomal bay length/width 1.90–2.32 (2.25); anterior venter/genital field length 1.37–1.66 (1.54); anterior venter length/genital field width 1.56–1.86 (1.78); anterior venter/medial suture 3.25–3.64 (3.64).

**Immatures** unknown.

######## Etymology.

Specific epithet (*raptoroides*) refers to the long, thin pedipalp tibia and long genual/femoral tubercles, which are similar to members of the Raptor Group (*rapio*, L. to seize; -*oides*, G. resembling).

######## Distribution.

**Figure 214.**
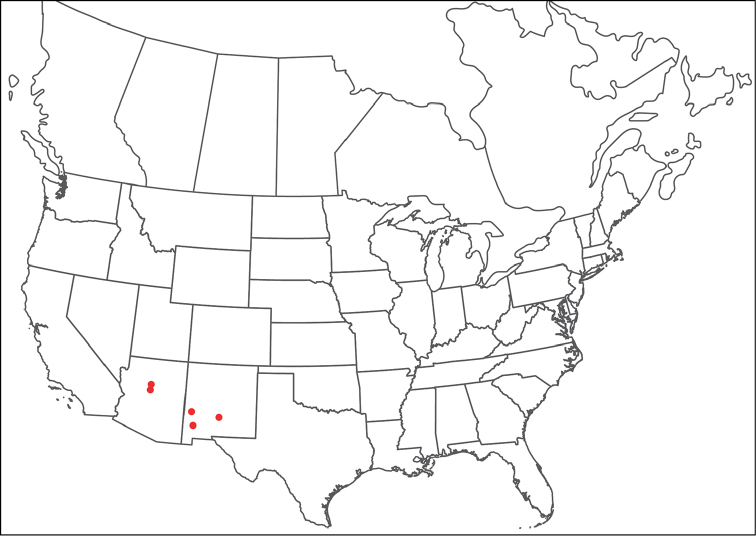
*Torrenticola raptoroides* sp. n. distribution.

Southeastern, Arizona and New Mexico (Figure 214).

**Figure 215.**
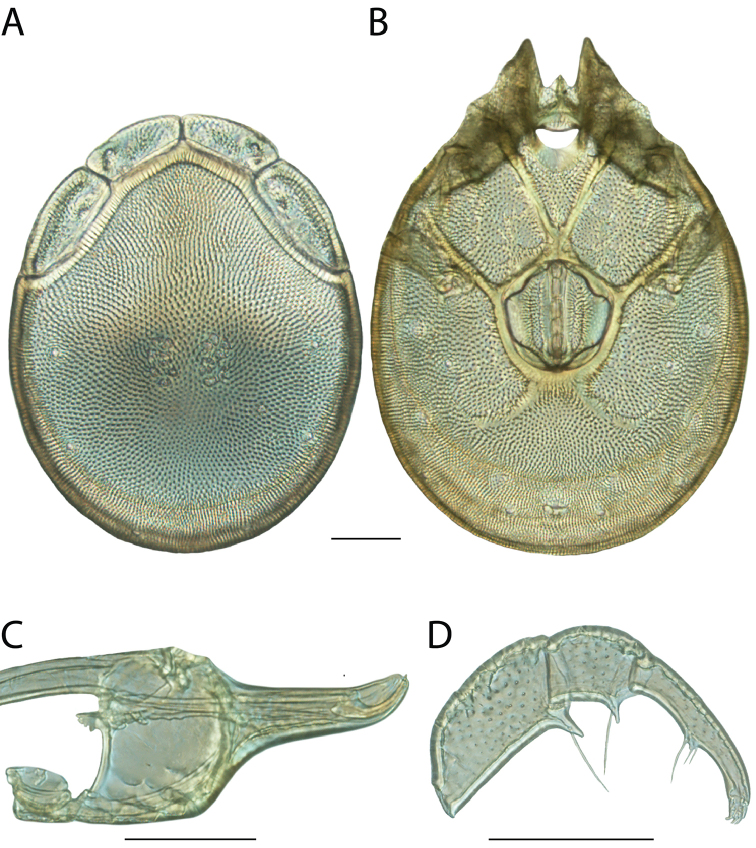
*Torrenticola raptoroides* sp. n. female: **A** dorsal plates **B** venter (legs removed) **C** subcapitulum **D** pedipalp (setae not accurately depicted). Scale = 100 µm.

**Figure 216.**
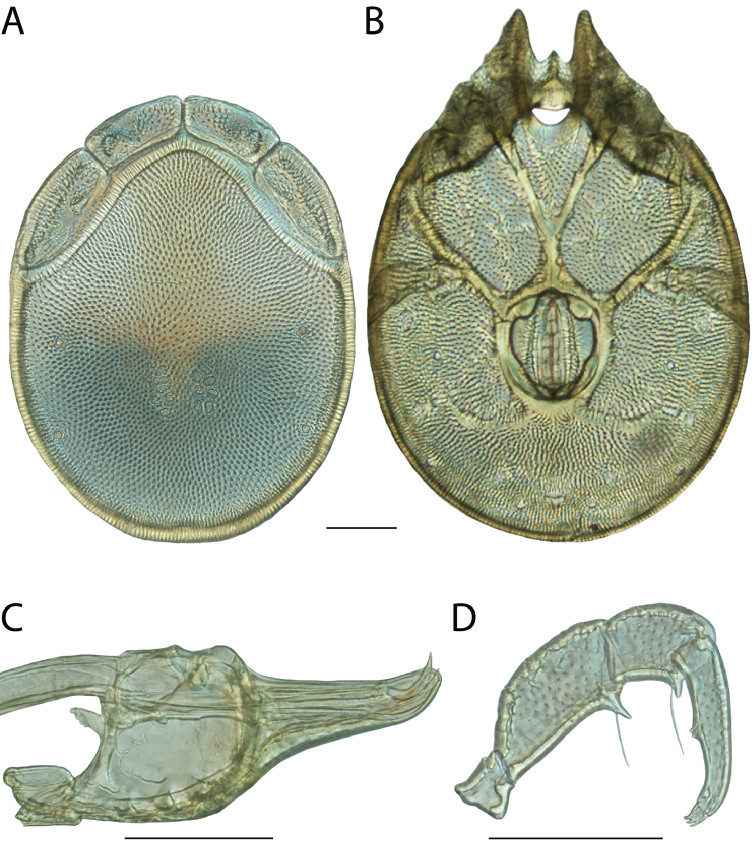
*Torrenticola raptoroides* sp. n. male: **A** dorsal plates **B** venter (legs removed) **C** subcapitulum **D** pedipalp (setae not accurately depicted). Scale = 100 µm.

######## Remarks.

Our analyses were unable to confidently place *Torrenticola raptoroides* phylogenetically. Both analyses place this species at the base of the Raptor Complex, but this relationship was not well-supported. Because of this ambiguity, we refrain from placing this species in a species complex. Furthermore, because of the unique morphology, we are also unable to place this species within an identification group.

All specimens are less than 1% different in COI sequence and are greater than 15% different from sister species. This species hypothesis is supported by biogeography, low COI divergence within the species (0–2%) and high divergence between species (3–15%), and by the morphological characters outlined in the diagnosis.

####### Torrenticola reduncarostra

Taxon classificationAnimaliaTrombidiformesTorrenticolidae

Fisher & Dowling sp. n.

http://zoobank.org/F3BC5217-1524-4F40-9D2C-549F20E161E9

######## Material examined.

HOLOTYPE (♀): from USA, Virginia, Washington County, Damascus; beside Rt. 58 just inside boundary of Mount Rogers National Recreation Area, (36°38'38"N, 81°45'45"W), 10 July 1990, by IM Smith, IMS900085A.

PARATYPES (8 ♀; 7 ♂): **Alabama, USA**: 1 ♂ from Clay County, beside Forest Route 649, 0.8 km northeast of road from Campbell Springs to Forest Route 600, (33°22'22"N, 85°52'52"W), 3 July 1990, by IM Smith, IMS900075A • **Maine, USA**: 1 ♀ and 1 ♂ from Aroostook County, Ashland; beside Rt. 11 at bridge over Aroostook River, (46°38'38"N, 68°24'24"W), 4 July 1989, by IM Smith, IMS890067 • **New Brunswick, Canada**: 2 ♀ and 1 ♂ from York County, beside Hwy. 8, 1.7 km north of road to Durham Bridge, (46°7'7"N, 66°36'36"W), 2 July 1989, by IM Smith, IMS890058 • **New York, USA**: 1♀ and 1 ♂ from Schuyler County, beside Town Line Road off Route 228, 0.6 km south of Perry City, (42°29'29"N, 76°42'42"W), 21 July 1990, by IM Smith, IMS900112A • **Tennessee, USA**: 1 ♀ from Monroe County, beside Forest Route 35, 2.0 km northeast of road from Rt. 165 to Miller Chapel Baptist Church, (35°21'21"N, 84°9'9"W), 5 July 1990, by IM Smith, IMS900078 • 2 ♀ and 2 ♂ from Monroe County, beside Forest Route 210, 1.8 km east of bridge at Bald River Falls, (35°19'19"N, 84°10'10"W), 5 July 1990, by IM Smith, IMS900079 • **Virginia, USA**: 1 ♂ (ALLOTYPE) from Washington County, Damascus; beside Rt. 58 just inside boundary of Mount Rogers National Recreation Area, (36°38'38"N, 81°45'45"W), 10 July 1990, by IM Smith, IMS900085A • 1 ♀ from Washington County, Damascus; beside Rt. 58 just inside boundary of Mount Rogers National Recreation Area, (36°38'38"N, 81°45'45"W), 10 July 1990, by IM Smith, IMS900085A

######## Type deposition.

Holotype (♀), allotype (♂), and some paratypes (5 ♀; 3 ♂) deposited in the CNC; other paratypes (3 ♀; 3 ♂) deposited in the ACUA.

######## Diagnosis.

*Torrenticola reduncarostra* are similar to species with similar dorsal patterning, such as the Rusetria “4-Plate” group (*T. dunni*, *T. glomerabilis*, *T. kittatinniana*, *T. pollani*, *T. rufoalba* and *T. shubini*), Neoanomala Group (*T. interiorensis* and *T. neoanomala*), and *T. bondi*, *T. gorti*, *T. elongata*, *T. erectirostra*, *T. robisoni*, *T. irapalpa*, *T. racupalpa*, *T. skvarlai*, and *T. arktonyx*. *T. reduncarostra* can be differentiated from all *Torrenticola*, except Erectirostra Group, by having an upturned rostrum. *T. reduncarostra* can be differentiated from *T. erectirostra*, *T. karambita*, and *T. robisoni* by lacking dentation on the rostrum (others have strong dentation on the lateral edge of the rostrum).

######## Description.

**Female (Figure 218)** (n = 5) (holotype measurements in parentheses when available) with characters of the genus with following specifications.

**Dorsum** — (610–665 (625) long; 420–450 (420) wide) ovoid with faint purple coloration separated into anterior and posterior portions, occasionally colorless. Anterio-medial platelets (120–130 (120) long; 55–57.5 (55) wide). Anterio-lateral platelets (175–205 (185) long; 57.5–67.5 (60) wide) free from dorsal plate. Dgl-4 approximately half-way between the edge of the dorsum and the muscle scars (distance between Dgl-4 280–315 (290)). Dorsal plate proportions: dorsum length/width 1.45–1.5 (1.49); dorsal width/distance between Dgl-4 1.40–1.50 (1.45); anterio-medial platelet length/width 2.18–2.27 (2.18); anterio-lateral platelet length/width 2.59–3.42 (3.08); anterio-lateral/anterio-medial length 1.46–1.58 (1.54).

**Gnathosoma — Subcapitulum** (330–340 (330) long (ventral); 240–255 (240) long (dorsal); 110–120 (120) tall) colorless. Rostrum (130–140 (132.5) long; 35–37.5 (35) wide) elongate and upturned. Chelicerae (345–360 (350) long) with curved fangs (55–60 (55) long). Subcapitular proportions: ventral length/height 2.75–3.09 (2.75); rostrum length/width 3.71–4.00 (3.79). **Pedipalps** with tuberculate ventral extensions on femora and genua. Palpomeres: trochanter (45–50 (47.5) long); femur (115–120 (115) long); genu (62.5–65 (62.5) long); tibia (67.5–72.5 (70) long; 23.75–26.25 (26.25) wide); tarsus (16.25–20 (17.5) long). Palpomere proportions: femur/genu 1.81–1.85 (1.84); tibia/femur 0.58–0.62 (0.61); tibia length/width 2.67–2.85 (2.67).

**Venter** — (740–800 (740) long; 470–515 (500) wide) colorless. Gnathosomal bay (145–165 (155) long; 65–70 (67.5) wide). Cxgl-4 subapical. **Medial suture** (25–35 (25) long). **Genital plates** (170–192.5 (170) long; 150–157.5 (155) wide). Additional measurements: Cx-1 (290–320 (305) long (total); 150–165 (155) long (medial)); Cx-3 (325–350 (330) wide); anterior venter (190–210 (190) long). Ventral proportions: gnathosomal bay length/width 2.07–2.46 (2.30); anterior venter/genital field length 1.07–1.24 (1.12); anterior venter length/genital field width 1.23–1.40 (1.23); anterior venter/medial suture 6.00–8.10 (7.60).

**Male (Figure 219)** (n = 5) (allotypic measurements in parentheses when available) with characters of the genus with following specifications.

**Dorsum** — (480–580 (525) long; 295–360 (335) wide) ovoid with faint purple coloration separated into anterior and posterior portions, occasionally colorless. Anterio-medial platelets (95–115 (115) long; 42.5–52.5 (50) wide). Anterio-lateral platelets (165–195 (170) long; 52.5–55 (52.5) wide) free from dorsal plate. Dgl-4 approximately halfway between the edge of the dorsum and the muscle scars (distance between Dgl-4 215–255 (220)). Dorsal plate proportions: dorsum length/width 1.51–1.68 (1.57); dorsal width/distance between Dgl-4 1.37–1.52 (1.52); anterio-medial platelet length/width 2.19–2.30 (2.30); anterio-lateral platelet length/width 3.14–3.71 (3.24); anterio-lateral/anterio-medial length 1.48–1.79 (1.48).

**Gnathosoma — Subcapitulum** (255–300 (290) long (ventral); 195–220 (207.5) long (dorsal); 87.5–95 (95) tall) colorless. Rostrum (102.5–120 (110) long; 27.5–32.5 (30) wide) elongate and upturned. Chelicerae (250–300 (300) long) with curved fangs (45–55 (55) long). Subcapitular proportions: ventral length/height 2.91–3.16 (3.05); rostrum length/width 3.54–3.73 (3.67). **Pedipalps** with tuberculate ventral extensions on femora and genua. Palpomeres: trochanter (40–42.5 (42.5) long); femur (90–105 (102.5) long); genu (52.5–60 (58.75) long); tibia (62.5–72.5 (67.5) long; 22.5–25 (25) wide); tarsus (15–17.5 (17.5) long). Palpomere proportions: femur/genu 1.69–1.83 (1.74); tibia/femur 0.66–0.72 (0.66); tibia length/width 2.70–3.11 (2.70).

**Venter** — (595–710 (650) long; 345–410 (370) wide) colorless. Gnathosomal bay (122.5–145 (137.5) long; 57.5–67.5 (57.5) wide). Cxgl-4 subapical. **Medial suture** (75–105 (85) long). **Genital plates** (135–150 (140) long; 100–105 (102.5) wide). Additional measurements: Cx-1 (250–280 (270) long (total); 130–140 (135) long (medial)); Cx-3 (270–300 (295) wide); anterior venter (222.5–257.5 (245) long). Ventral proportions: gnathosomal bay length/width 1.96–2.39 (2.39); anterior venter/genital field length 1.53–1.81 (1.75); anterior venter length/genital field width 2.23–2.45 (2.39); anterior venter/medial suture 2.45–3.19 (2.88).

**Immatures** unknown.

######## Etymology.

Specific epithet (*reduncarostra*) refers to the rostrum, which is curved upwards anteriorly, a rare condition outside of the Erectirostra Group (*reduncus*, L. bent backward; *rostrum*, L. snout).

######## Distribution.

**Figure 217.**
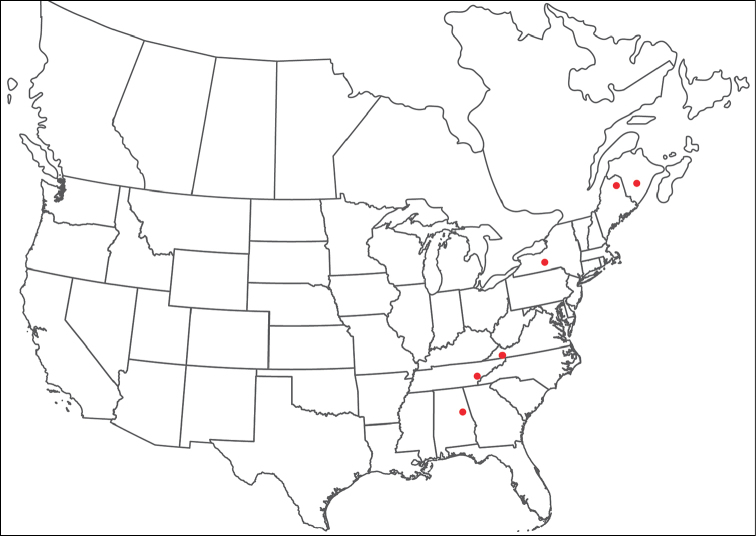
*Torrenticola reduncarostra* sp. n. distribution.

Appalachians (Figure 217).

**Figure 218.**
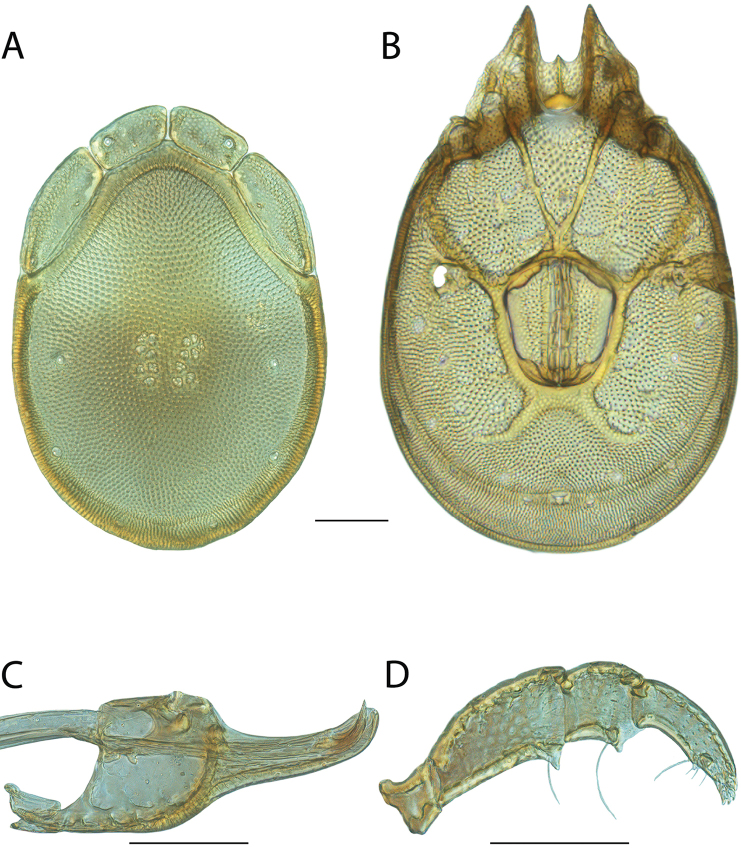
*Torrenticola reduncarostra* sp. n. female: **A** dorsal plates **B** venter (legs removed) **C** subcapitulum **D** pedipalp (setae not accurately depicted). Scale = 100 µm.

**Figure 219.**
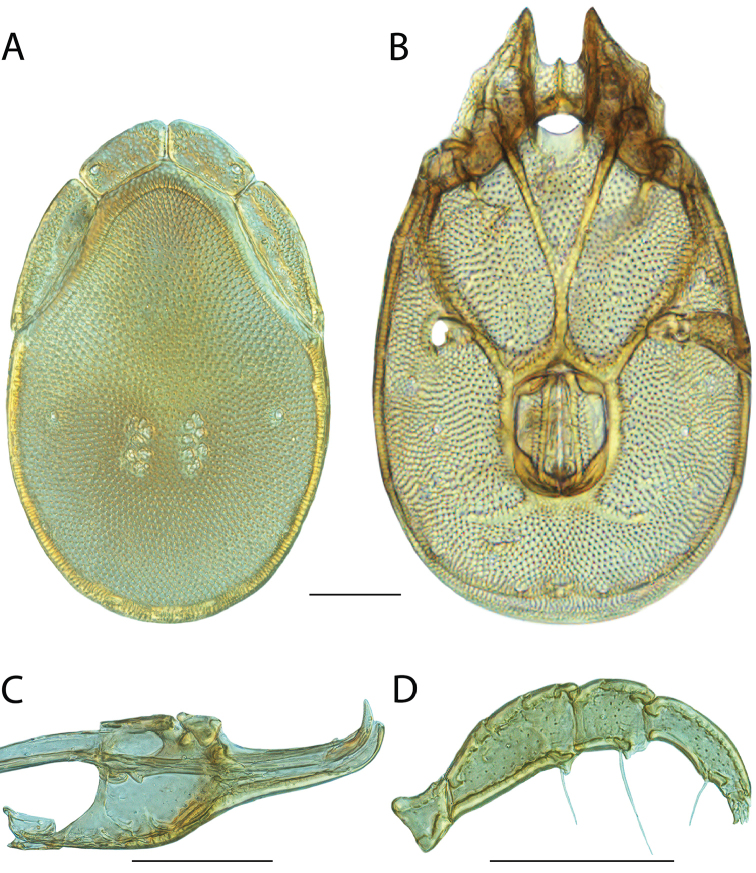
*Torrenticola reduncarostra* sp. n. male: **A** dorsal plates **B** venter (legs removed) **C** subcapitulum **D** pedipalp (setae not accurately depicted). Scale = 100 µm.

######## Remarks.

Unfortunately, we were unable to acquire fresh material of *Torrenticola reduncarostra* and therefore this species is not included in our phylogenetic analyses. However, we were able to examine morphology with material preserved in GAW. The overall appearance, small size, and elongate, ovoid body shape are consistent with placing this species in the Raptor Complex and within the Elongata Identification Group.

####### Torrenticola regalis

Taxon classificationAnimaliaTrombidiformesTorrenticolidae

Fisher & Dowling sp. n.

http://zoobank.org/DC4A22B6-7EBA-42FC-9DF1-D2AF617CCEC5

######## Material examined.

HOLOTYPE (♂): from USA, Oregon, Curry County, Port Orford, beside Elk River Road, 9 km east of Elk River Fish Hatchery (42°42'22"N, 124°20'28"W), 18 Jun 2010, by IM Smith, IMS100075, DNA 1442.

PARATYPES (2 ♀; 6 ♂): **Oregon, USA**: 1 ♀ (ALLOTYPE) from Curry County, Port Orford; beside Elk River Road 9.0 km east of Elk River Fish Hatchery, (42°42'42"N, 124°20'20"W), 18 June 2010, by IM Smith, IMS100075 • 1 ♀ and 6 ♂ from Curry County, Port Orford; beside Elk River Road 9.0 km east of Elk River Fish Hatchery, (42°42'42"N, 124°20'20"W), 18 June 2010, by IM Smith, IMS100075

######## Type deposition.

Holotype (♀), allotype (♂), and most paratypes (1 ♀; 3 ♂) deposited in the CNC; other paratypes (1 ♀; 2 ♂) deposited in ACUA.

######## Diagnosis.

*Torrenticola regalis* can be differentiated from all other western *Torrenticola* by having purple coloration covering the entire dorsal plate and by being the only member of the Miniforma Complex with indistinct or partial hind coxal margins. Occasionally its coloration can look similar to *T. tahoei*, but *T. regalis* can be differentiated from *T. tahoei* by having a shorter anterior venter (200–265 in *T. regalis*, 285–325 in *T. tahoei*), a stockier subcapitulum (ventral length/height = 2.41–2.68 in *T. regalis*, 3.25–4.11 in *T. tahoei*), and by having indistinct hind coxal margins (distinct in *T. tahoei*).

######## Description.

**Female (Figure 221)** (n = 2) (allotypic measurements in parentheses when available) with characters of the genus with following specifications.

**Dorsum** — (680–695 (695) long; 540–540 (540) wide) circular with purple coloration, excepting platelets. Anterio-medial platelets (152.5–160 (160) long; 65–65 (65) wide). Anterio-lateral platelets (207.5–222.5 (222.5) long; 80–83.75 (83.75) wide) free from dorsal plate. Dgl-4 much closer to the edge of dorsum than to the muscle scars (distance between Dgl-4 405–420 (420)). Dorsal plate proportions: dorsum length/width 1.26–1.29 (1.29); dorsal width/distance between Dgl-4 1.29–1.33 (1.29); anterio-medial platelet length/width 2.35–2.46 (2.46); anterio-lateral platelet length/width 2.59–2.66 (2.66); anterio-lateral/anterio-medial length 1.36–1.39 (1.39).

**Gnathosoma — Subcapitulum** (400–410 (410) long (ventral); 295–307.5 (307.5) long (dorsal); 160–170 (170) tall) colorless. Rostrum (155–165 (165) long; 52.5–55 (55) wide). Chelicerae (410–420 (420) long) with curved fangs (70–75 (70) long). Subcapitular proportions: ventral length/height 2.41–2.50 (2.41); rostrum length/width 2.95–3.00 (3.00). **Pedipalps** with tuberculate ventral extensions on femora and tuberculate ventral extensions with dentate tip on genua. Palpomeres: trochanter (45–47.5 (45) long); femur (132.5–132.5 (132.5) long); genu (80–85 (85) long); tibia (90–93.75 (93.75) long; 30–30 (30) wide); tarsus (17.5–17.5 (17.5) long). Palpomere proportions: femur/genu 1.56–1.66 (1.56); tibia/femur 0.68–0.71 (0.71); tibia length/width 3.00–3.13 (3.13).

**Venter** — (860–860 (860) long; 630–630 (630) wide) colorless. Gnathosomal bay (197.5–205 (205) long; 75–80 (75) wide). Cxgl-4 subapical. **Medial suture** (30–40 (30) long). **Genital plates** (225–232.5 (232.5) long; 205–205 (205) wide). Additional measurements: Cx-1 (340–350 (350) long (total); 140–145 (145) long (medial)); Cx-3 (395–405 (405) wide); anterior venter 200–200 (200) long). Ventral proportions: gnathosomal bay length/width 2.47–2.73 (2.73); anterior venter/genital field length 0.86–0.89 (0.86); anterior venter length/genital field width 0.98–0.98 (0.98); anterior venter/medial suture 5.00–6.67 (6.67).

**Male (Figure 222)** (n = 5) (holotype measurements in parentheses when available) with characters of the genus with following specifications.

**Dorsum** — (580–640 (630) long; 465–520 (490) wide) circular with purple coloration, excepting platelets. Anterio-medial platelets (127.5–145 (140) long; 55–67.5 (67.5) wide). Anterio-lateral platelets (177.5–217.5 (203.75) long; 72.5–85 (82.5) wide) free from dorsal plate. Dgl-4 much closer to the edge of dorsum than to the muscle scars (distance between Dgl-4 375–420 (400)). Dorsal plate proportions: dorsum length/width 1.23–1.29 (1.29); dorsal width/distance between Dgl-4 1.23–1.27 (1.23); anterio-medial platelet length/width 2.07–2.32 (2.07); anterio-lateral platelet length/width 2.44–2.64 (2.47); anterio-lateral/anterio-medial length 1.39–1.50 (1.46).

**Gnathosoma — Subcapitulum** (355–375 (365) long (ventral); 255–280 (270) long (dorsal); 132.5–150 (145) tall) colorless. Rostrum (140–150 (145) long; 45–55 (55) wide). Chelicerae (350–375 (365) long) with curved fangs (65–70 (67.5) long). Subcapitular proportions: ventral length/height 2.50–2.68 (2.52); rostrum length/width 2.64–3.16 (2.64). **Pedipalps** with tuberculate ventral extensions on femora and tuberculate ventral extensions with dentate tip on genua. Palpomeres: trochanter (41.25–50 (45) long); femur (113.75–125 (125) long); genu (72.5–82.5 (80) long); tibia (82.5–92.5 (92.5) long; 23.75–27.5 (27.5) wide); tarsus (17.5–17.5 (17.5) long). Palpomere proportions: femur/genu 1.48–1.57 (1.56); tibia/femur 0.71–0.74 (0.74); tibia length/width 3.36–3.50 (3.36).

**Venter** — (730–780 (770) long; 535–640 (640) wide) colorless. Gnathosomal bay (160–185 (160) long; 72.5–92.5 (92.5) wide). Cxgl-4 subapical. **Medial suture** (75–90 (90) long). **Genital plates** (185–190 (185) long; 145–155 (152.5) wide). Additional measurements: Cx-1 (305–340 (320) long (total); 135–160 (160) long (medial)); Cx-3 (350–410 (410) wide); anterior venter 237.5–265 (265) long). Ventral proportions: gnathosomal bay length/width 1.73–2.55 (1.73); anterior venter/genital field length 1.28–1.43 (1.43); anterior venter length/genital field width 1.62–1.74 (1.74); anterior venter/medial suture 2.64–3.47 (2.94).

**Immatures** unknown.

######## Etymology.

Specific epithet (*regalis*) refers to the coloration of this species, which is nearly colorless except the purple dorsum, giving the appearance of a mite wearing a purple cape, such as those often depicted as worn by royalty (*rēgālis*, L. royal).

######## Distribution.

**Figure 220.**
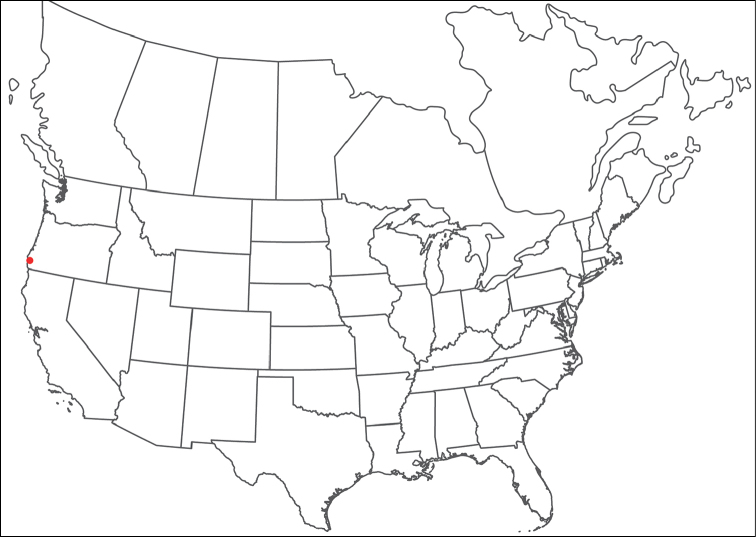
*Torrenticola regalis* sp. n. distribution.

Known only from Curry County, Oregon (Figure 220).

**Figure 221.**
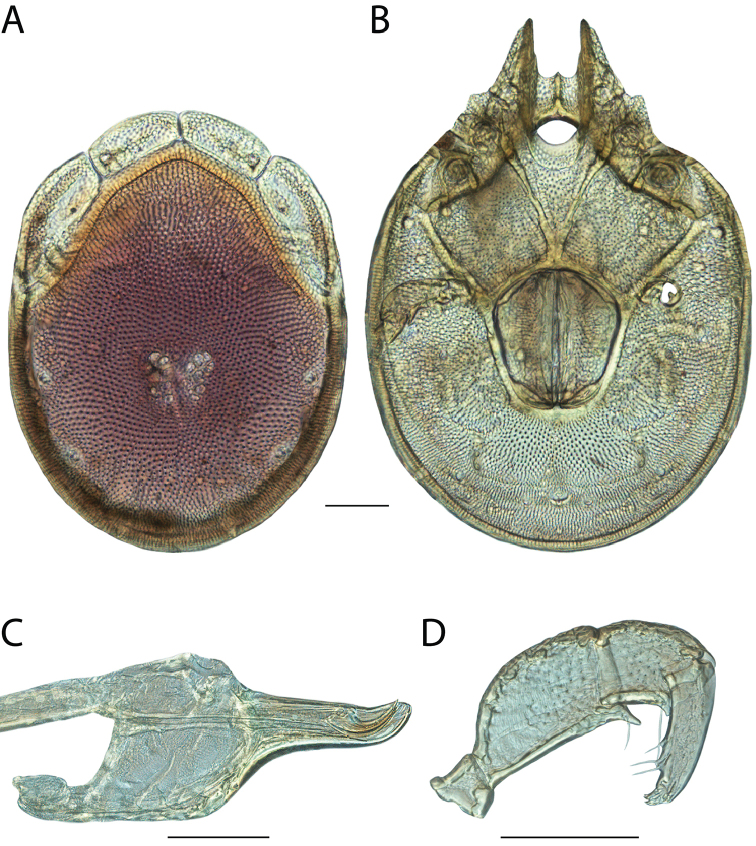
*Torrenticola regalis* sp. n. female: **A** dorsal plates **B** venter (legs removed) **C** subcapitulum **D** pedipalp (setae not accurately depicted). Scale = 100 µm.

**Figure 222.**
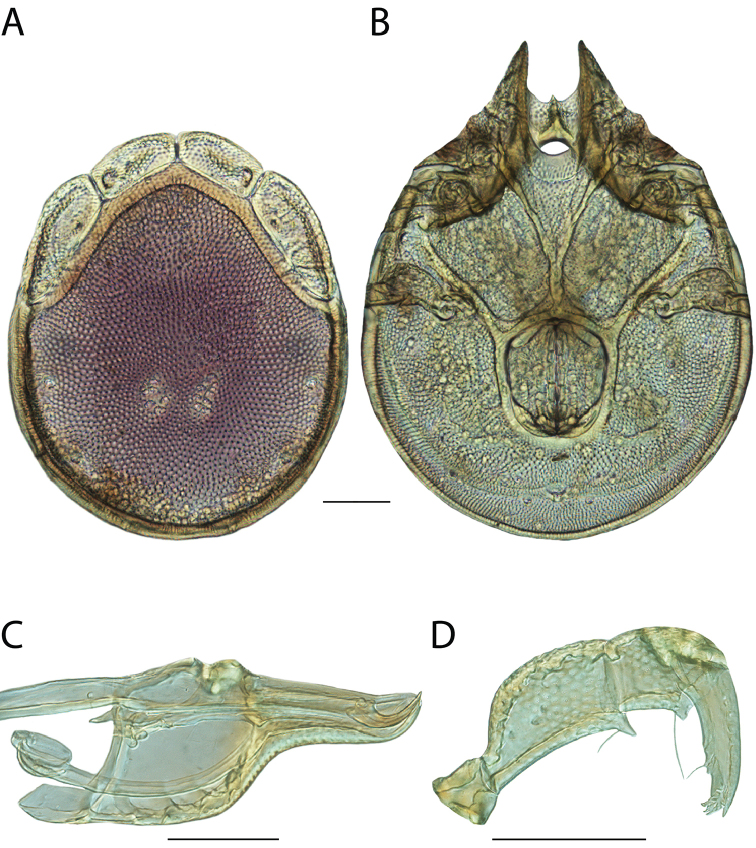
*Torrenticola regalis* sp. n. male: **A** dorsal plates **B** venter (legs removed) **C** subcapitulum **D** pedipalp (setae not accurately depicted). Scale = 100 µm.

######## Remarks.

*Torrenticola regalis* groups with other members of the Miniforma Complex with high support and is greater than 17% different from other species in the Ellipsoidalis Identification Group. This species is represented by a single specimen that does not resemble any other species group. This species hypothesis is supported by phylogenetic affinity, high divergence between species (3–15%), and by the morphological characters outlined in the diagnosis.

####### Torrenticola robisoni

Taxon classificationAnimaliaTrombidiformesTorrenticolidae

Fisher & Dowling sp. n.

http://zoobank.org/F5FEE1EC-3580-4DF8-A138-499D8AABD9E4

######## Material examined.

HOLOTYPE (♀): from USA, Arkansas, Polk County, East Saline Creek, 30 Jul 2011, by IM Smith, IMS110041

PARATYPES (1 ♀; 0 ♂): **Oklahoma, USA**: Pushmataha County, beside Route 271, Walnut Creek (34°39'N 95°7'W), 1 Jul 1987, by IM Smith, IMS870063A

######## Type deposition.

Holotype (♀) deposited in the CNC; paratype (1 ♀) deposited in the ACUA.

######## Diagnosis.

*Torrenticola robisoni* are similar to other members of the Erectirostra Group (*T. karambita* and *T. erectirostra*). They are similar to other species with similar dorsal patterning, but can be differentiated from these by having a dentate, upturned rostrum that is wide when viewed ventrally. *T. robisoni* can be differentiated from *T. erectirostra* by having more elongate anterio-lateral platelets (length/width ♀ = 2.96–3.00 in *T. robisoni*, 2.52–2.69 in *T. erectirostra*). *T. robisoni* can be differentiated from *T. karambita* by having dorsal coloration (*T. karambita* is colorless) and having a more elongate rostrum (length/width ♀ = 2.09–2.09 in *T. robisoni*, 1.57–1.62 in *T. karambita*). *T. robisoni* can be further differentiated from *T. erectirostra* and *T. karambita* by being distributed in the Interior Highlands, while the others are in the Appalachians.

######## Description.

**Female (Figure 224)** (n = 2) (holotype measurements in parentheses when available) with characters of the genus with following specifications.

**Dorsum**— (650–670 (650) long; 455–500 (455) wide) ovoid with reddish purple coloration separated into anterior and posterior portions with orange medially. Anterio-medial platelets (147.5–150 (150) long; 60–62.5 (60) wide). Anterio-lateral platelets (200–210 (200) long; 67.5–70 (67.5) wide) free from dorsal plate. Dgl-4 closer to the edge of the dorsum than to the muscle scars (distance between Dgl-4 305–340 (305)). Dorsal plate proportions: dorsum length/width 1.34–1.43 (1.34); dorsal width/distance between Dgl-4 1.47–1.49 (1.49); anterio-medial platelet length/width 2.36–2.50 (2.50); anterio-lateral platelet length/width 2.96–3.00 (2.96); anterio-lateral/anterio-medial length 1.33–1.42 (1.33).

**Gnathosoma — Subcapitulum** (325–335 (325) long (ventral); 218–250 (218) long (dorsal); 125–132.5 (132.5) tall) colorless. Rostrum (115–120 (115) long; 55–57.5 (55) wide) wide and upturned with dentation. Chelicerae (315–330 (315) long) with curved fangs (46–50 (46) long). Subcapitular proportions: ventral length/height 2.45–2.68 (2.45); rostrum length/width 2.09–2.09 (2.09). **Pedipalps** short and stocky (especially tibiae) with tuberculate ventral extensions on femora and genua. Palpomeres: trochanter (52.5–52.5 (52.5) long); femur (95–107.5 (95) long); genu (60–62.5 (60) long); tibia (47.5–58.75 (47.5) long; 25–27.5 (25) wide); tarsus (15–17.5 (15) long). Palpomere proportions: femur/genu 1.58–1.72 (1.58); tibia/femur 0.50–0.55 (0.50); tibia length/width 1.90–2.14 (1.90).

**Venter** — (779–825 (779) long; 543–550 (543) wide) colorless. Gnathosomal bay (165–190 (165) long; 115–120 (115) wide). Cxgl-4 far from apex. **Medial suture** (12.5–20 (12.5) long). **Genital plates** (168.75–202.5 (202.5) long; 157.5–180 (180) wide). Additional measurements: Cx-1 (308–350 (308) long (total); 142–155 (142) long (medial)); Cx-3 (367–375 (367) wide); anterior venter (182.5–200 (182.5) long). Ventral proportions: gnathosomal bay length/width 1.43–1.58 (1.43); anterior venter/genital field length 1.05–1.08 (1.08); anterior venter length/genital field width 1.16–1.25 (1.16); anterior venter/medial suture 10.00–14.60 (14.60).

**Male** unknown.

**Immatures** unknown.

######## Etymology.

Specific epithet (*robisoni*) named in honor of Henry W. Robison for his efforts in communicating the importance of the Interior Highlands (Ozarks and Ouachitas)—the type locality—which have a high proportion of endemic species, yet are understudied with respect to other areas of increased endemism (e.g., California floristic province, coastal plains, southern Appalachians, Pacific Northwest). His contributions instill passion for the region to even the casual reader, and have inspired many, including JRF, to pursue biodiversity research in the area.

######## Distribution.

**Figure 223.**
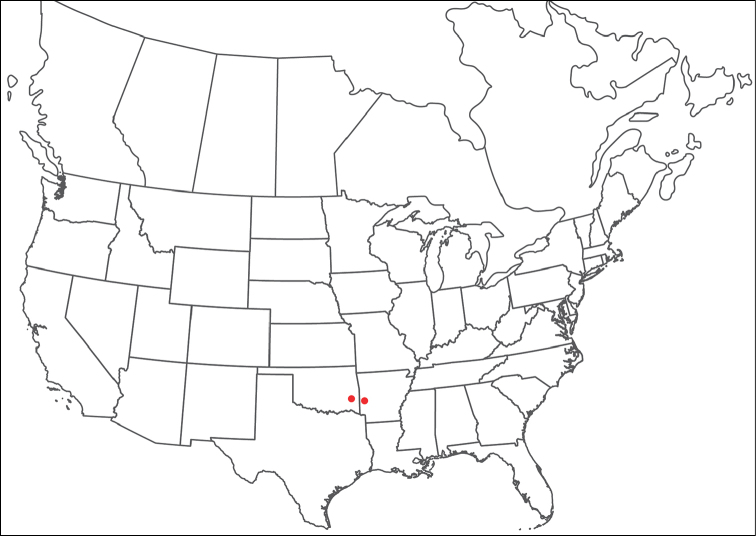
*Torrenticola robisoni* sp. n. distribution.

Known only from the Ouachita Mountains, possibly endemic (Figure 223).

**Figure 224.**
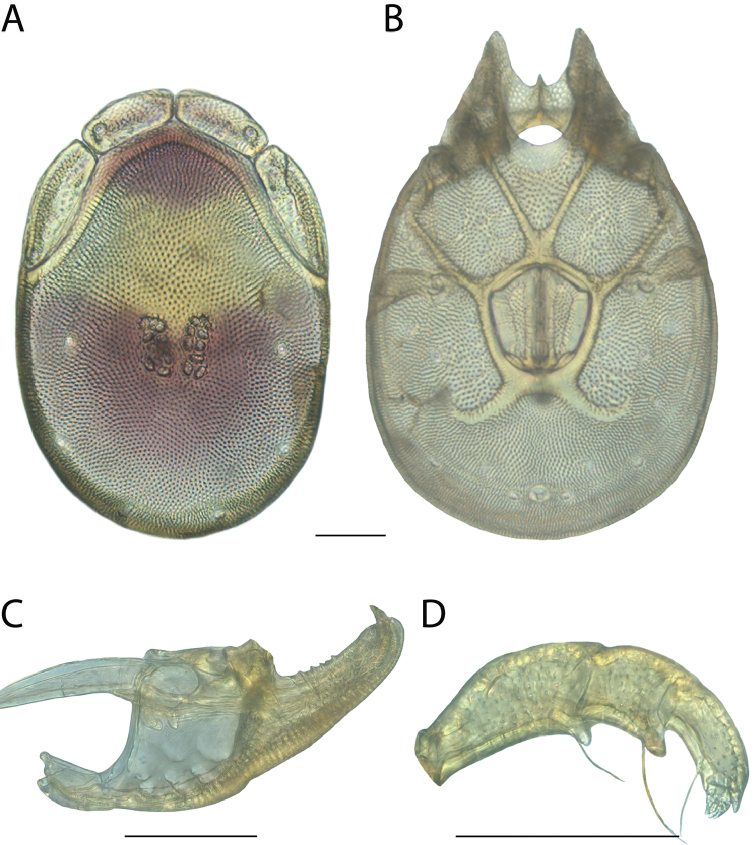
*Torrenticola robisoni* sp. n. female: **A** dorsal plates **B** venter (legs removed) **C** subcapitulum **D** pedipalp (setae not accurately depicted). Scale = 100 µm.

######## Remarks.

*Torrenticola robisoni* groups with other members of the Raptor Complex with high support. The single *T. robisoni* specimen that we were able to include in our phylogenetic analysis is 8–9% different in COI sequence from *T. erectirostra* and *T. karambita*. Based upon the distinctive morphology of the gnathosoma of these species, we place them in the Erectirostra Identification group.

This species hypothesis is supported by biogeography, high divergence between species, and by morphological characters outlined in the diagnosis.

####### Torrenticola rockyensis

Taxon classificationAnimaliaTrombidiformesTorrenticolidae

Fisher & Dowling sp. n.

http://zoobank.org/F1B21665-2736-40B5-B1FC-C74B58457661

######## Material examined.

HOLOTYPE (♀): from USA, Idaho, Blaine County, Sawtooth National Forest, Salmon River (43°53'7"N, 114°46'15"W), 29 Jul 2012, by JR Fisher, WA Nelson, & JC O’Neill, ROW 12-0729-002, DNA 2623.

PARATYPES (4 ♀; 6 ♂): Idaho, USA: 2 ♀ and 2 ♂ from Blaine County, Salmon River, beside Route 75 between Obsidian & Galena Summit, 3 Jul 1985, by IM Smith, IMS850067 • 1 ♂ from Blaine County, Sawtooth National Forest, Salmon River (43°53'7"N, 114°46'15"W), 29 Jul 2012, by JR Fisher, WA Nelson, & JC O’Neill, ROW 12-0729-002 • 2 ♀ and 3 ♂ from Custer County, Basin Creek campground, beside Route 75 between Sunbeam & Stanley Basin Creek, 2 Jul 1985, by IM Smith, IMS850066 • 1 ♀ and 1 ♂ from Custer County, Challis National Forest, Stanley Creek (44°15'12"N, 115°0'19"W), 30 Jul 2012, by JR Fisher, WA Nelson, & JC O’Neill, ROW 12-0730-005 • 1 ♀ and 1 ♂ from Custer County, Salmon River (44°12'31"N, 114°55'51"W), 29 Jul 2012, by JR Fisher, WA Nelson, & JC O’Neill, ROW 12-0729-003 • 1 ♂ from Custer County, Stanley, Little Redfish Lake, 3 Jul 1985, by IM Smith, IMS850069 • 2 ♀ from Lemhi County, North Fork of Salmon River, beside Route 93, 15 kilometers north of North Fork, 1 Jul 1985, by IM Smith, IMS850062 • 1 ♂ (ALLOTYPE) from Lemhi County, Salmon National Forest, Niapas Creek (45°8'15"N, 114°13'4"W), 2 Aug 2012, by JR Fisher, WA Nelson, & JC O’Neill, ROW 12-0802-003, DNA 2626 • 1 ♀ from Lemhi County, Salmon National Forest, Niapas Creek (45°8'15"N, 114°13'4"W), 2 Aug 2012, by JR Fisher, WA Nelson, & JC O’Neill, ROW 12-0802-003 • Montana, USA 1 ♂ from Missoula County, Lolo National Forest, Lolo Creek (46°41'51"N, 114°32'34"W), 7 Aug 2012, by JR Fisher, WA Nelson & JC O’Neill, ROW 12-0807-002 • 1 ♀ from Missoula County, Lolo National Forest, Lolo Creek (46°46'7"N, 114°27'53"W), 7 Aug 2012, by JR Fisher, WA Nelson & JC O’Neill, ROW 12-0807-003 • 1 ♂ from Ravalli County, Bitterroot National Forest, Soda Spring Creek (45°47'12"N, 114°21'2"W), 6 Aug 2012, by JR Fisher, WA Nelson & JC O’Neill, ROW 12-0806-001 • 1 ♀ and 1 ♂ from Ravalli County, Medicine Springs, Spring Gulch campground, East Fork of Bitterroot River, beside Route 93, 1 Jul 1985, by IM Smith, IMS850060.

######## Type deposition.

Holotype (♀), allotype (♂), and other paratypes (2 ♀; 3 ♂) deposited in the CNC; other paratypes (2 ♀; 2 ♂) deposited in ACUA.

######## Diagnosis.

*Torrenticola rockyensis* are similar to members of the Miniforma group (*T. copipalpa*, *T. manni*, *T. miniforma*, *T. pacificensis*, *T. oliveri*, and *T. pinocchio*) in having short, stocky pedipalps (except *T. oliveri* and *T. pinocchio*); similar pedipalpal extensions (unique to members of this group); and being among the smallest *Torrenticola* in the west (dorsum 500–625 long) (except *T. oliveri*). *T. rockyensis* can be differentiated from all other Miniforma group by being distributed in the Rocky Mountains. *T. rockyensis* are best differentiated from *T. copipalpa* by having tuberculate pedipalp femoral extensions (broad and flat in *T. copipalpa*). *T. rockyensis* are best differentiated from *T. pacificensis* by females having more elongate subcapitular rostra (length/width ♀ = 2.72–2.91 in *T. rockyensis*, 2.59–2.68 in *T. pacificensis*). *T. rockyensis* are best differentiated from *T. manni* by having stockier pedipalp tibiae (length/width = 2.47–3.11 in *T. rockyensis*, 3.13–3.38 in *T. manni*). *T. rockyensis* are best differentiated from *T. miniforma* by being larger (dorsum length ♀ = 570–620 in *T. rockyensis*, 545 in *T. miniforma*; ♂ = 525–545 in *T. rockyensis*, 485 in *T. miniforma*) and having stockier subcapitular rostra (length/width ♀ = 2.72–2.91 in *T. rockyensis*, 3.13 in *T. miniforma*; ♂ = 2.83–3.00 in *T. rockyensis*, 3.19 in *T. miniforma*). *T. rockyensis* can be differentiated by having a shorter anterior venter (175–223 in *T. rockyensis*, 250–310 in *T. oliveri*) and less elongate pedipalpal tibiae (length/width = 2.4–3.1 in *T. rockyensis*, 3.6–4.2 in *T. oliveri*). *T. rockyensis* can be differentiated from *T. pinocchio* by having a less elongate rostrum (length/width = 2.7–3.0 in *T. rockyensis*, 4.5–4.9 in *T. pinocchio*).

######## Description.

**Female (Figure 226)** (n = 5) (holotype measurements in parentheses when available) with characters of the genus with following specifications.

**Dorsum** — (570–620 (620) long; 400–420 (400) wide) ovoid with purple coloration often restricted posteriorly, occasionally encroaching anteriorly nearly to the platelets (one specimen with purple on the platelets). Anterio-medial platelets (110–120 (120) long; 43.75–52.5 (52.5) wide). Anterio-lateral platelets (160–172.5 (172.5) long; 52.5–62.5 (62.5) wide) free from dorsal plate. Dgl-4 much closer to the edge of dorsum than to the muscle scars (distance between Dgl-4 290–310 (300)). Dorsal plate proportions: dorsum length/width 1.43–1.55 (1.55); dorsal width/distance between Dgl-4 1.33–1.38 (1.33); anterio-medial platelet length/width 2.19–2.51 (2.29); anterio-lateral platelet length/width 2.76–3.05 (2.76); anterio-lateral/anterio-medial length 1.43–1.55 (1.44).

**Gnathosoma — Subcapitulum** (300–315 (302.5) long (ventral); 222.5–235 (231) long (dorsal); 115–122.5 (117.5) tall) colorless. Rostrum (117.5–130 (123.75) long; 42.5–45 (42.5) wide). Chelicerae (283–310 (283) long) with curved fangs (52.5–68 (67) long). Subcapitular proportions: ventral length/height 2.49–2.68 (2.57); rostrum length/width 2.72–2.91 (2.91). **Pedipalps** short and stocky (especially tibiae) with tuberculate (occasionally broadly tuberculate), dentate ventral extensions on femora and dentate, flanged ventral extensions on genua. Palpomeres: trochanter 35–42.5 (42.5) long); femur (90–95 (95) long); genu (63.75–67.5 (67.5) long); tibia (62.5–70 (70) long; 21.25–23.75 (22.5) wide); tarsus (12.5–17.5 (15) long). Palpomere proportions: femur/genu 1.38–1.46 (1.41); tibia/femur 0.68–0.74 (1.74); tibia length/width 2.78–3.11 (3.11).

**Venter** — (615–770 (760) long; 339–470 (455) wide) colorless. Gnathosomal bay (120–147.5 (145) long; 65–80 (67.5) wide). Cxgl-4 subapical. **Medial suture** (42.5–60 (52.5) long). **Genital plates** (172.5–190 (185) long; 157.5–173.75 (170) wide). Additional measurements: Cx-1 (239–279 (279) long (total); 112–133 (121) long (medial)); Cx-3 (247–310 (296) wide); anterior venter (175–202.5 (190) long). Ventral proportions: gnathosomal bay length/width 1.71–2.27 (2.15); anterior venter/genital field length 0.99–1.07 (1.03); anterior venter length/genital field width 1.08–1.19 (1.12); anterior venter/medial suture 3.13–4.29 (3.62).

**Male (Figure 227)** (n = 5) (allotypic measurements in parentheses when available) with characters of the genus with following specifications.

**Dorsum** — (525–545 (535) long; 335–350 (350) wide) ovoid with purple coloration often restricted posteriorly, occasionally encroaching anteriorly nearly to the platelets. Anterio-medial platelets (102.5–106.25 (105) long; 45–47.5 (45) wide). Anterio-lateral platelets (145–155 (152.5) long; 52.5–55 (52.5) wide) free from dorsal plate. Dgl-4 much closer to the edge of dorsum than to the muscle scars (distance between Dgl-4 255–285 (280)). Dorsal plate proportions: dorsum length/width 1.53–1.57 (1.53); dorsal width/distance between Dgl-4 1.23–1.31 (1.25); anterio-medial platelet length/width 2.21–2.36 (2.33); anterio-lateral platelet length/width 2.68–2.90 (2.90); anterio-lateral/anterio-medial length 1.36–1.51 (1.45).

**Gnathosoma — Subcapitulum** (270–285 (285) long (ventral); 197.5–208 (207) long (dorsal); 102.5–107.5 (107.5) tall) colorless. Rostrum (106.25–112.5 (112.5) long; 36.25–38.75 (37.5) wide). Chelicerae (269–284 (282) long) with curved fangs (41–52 (46) long). Subcapitular proportions: ventral length/height 2.62–2.67 (2.65); rostrum length/width 2.83–3.00 (3.00). **Pedipalps** short and stocky (especially tibiae) with tuberculate (occasionally broadly tuberculate), dentate ventral extensions on femora and dentate, flanged ventral extensions on genua. Palpomeres: trochanter (30–40 (31.25) long); femur (81.25–86.25 (86.25) long); genu (57.5–60 (60) long); tibia (52.5–62.5 (62.5) long; 21.25–22.5 (22.5) wide); tarsus (12.5–15 (15) long). Palpomere proportions: femur/genu 1.41–1.44 (1.44); tibia/femur 0.62–0.74 (0.72); tibia length/width 2.47–2.82 (2.78).

**Venter** — (580–690 (690) long; 352–420 (385) wide) colorless. Gnathosomal bay (130–140 (140) long; 57.5–62.5 (62.5) wide). Cxgl-4 subapical. **Medial suture** (75–92.5 (92.5) long). **Genital plates** (137.5–147.5 (143.75) long; 105–113.75 (110) wide). Additional measurements: Cx-1 (234–266 (266) long (total); 98.25–137 (136) long (medial)); Cx-3 (245–270 (260) wide); anterior venter (197.5–222.5 (215) long). Ventral proportions: gnathosomal bay length/width 2.12–2.26 (2.24); anterior venter/genital field length 1.42–1.51 (1.50); anterior venter length/genital field width 1.82–1.98 (1.95); anterior venter/medial suture 2.32–2.73 (2.32).

**Immatures** unknown.

######## Etymology.

Specific epithet (*rockyensis*) refers to the distribution of this species in the Rocky Mountains. This location-based naming reflects that locality is the easiest way to differentiate this species from others in the Miniforma Group, particularly *T. pacificensis*.

######## Distribution.

**Figure 225.**
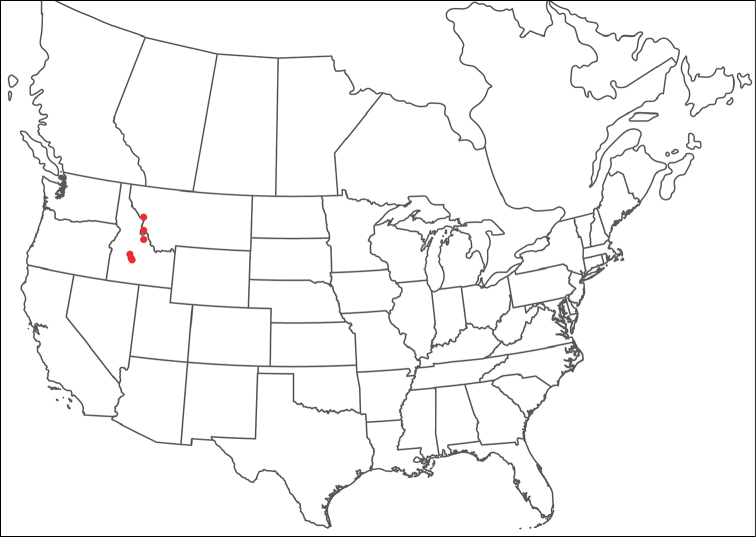
*Torrenticola rockyensis* sp. n. distribution.

Rocky Mountains of Idaho and Montana (Figure 225).

**Figure 226.**
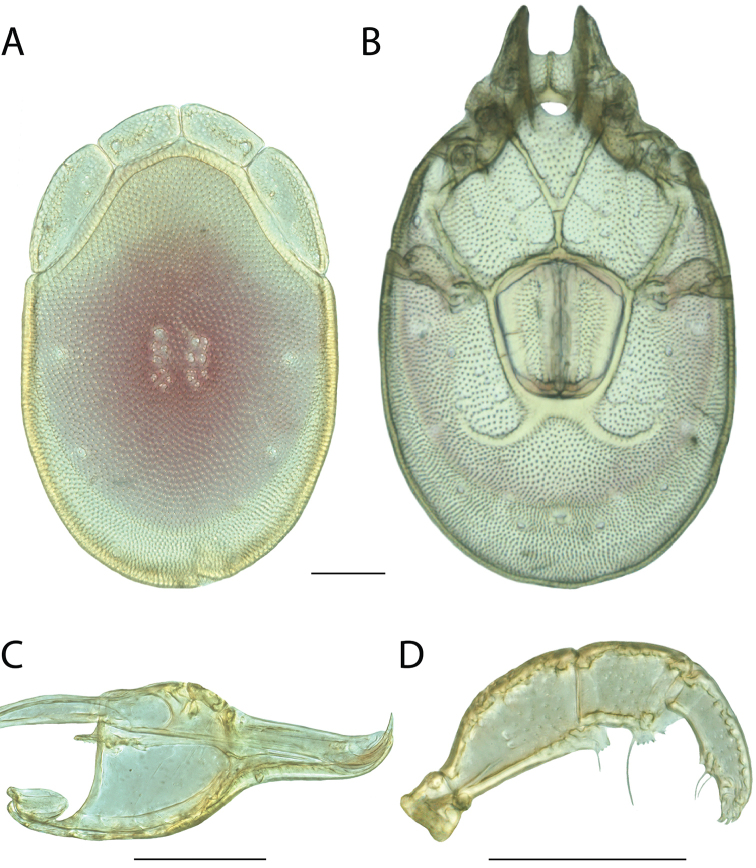
*Torrenticola rockyensis* sp. n. female: **A** dorsal plates **B** venter (legs removed) **C** subcapitulum **D** pedipalp (setae not accurately depicted). Scale = 100 µm.

**Figure 227.**
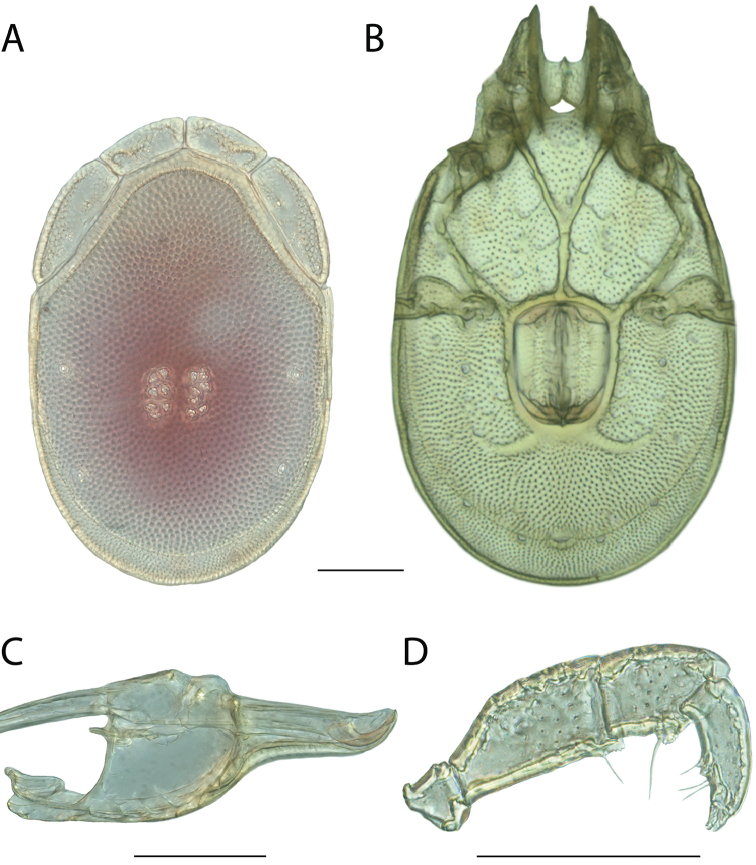
*Torrenticola rockyensis* sp. n. male: **A** dorsal plates **B** venter (legs removed) **C** subcapitulum **D** pedipalp (setae not accurately depicted). Scale = 100 µm.

######## Remarks.

*Torrenticola rockyensis* groups with other members of the Miniforma group in all analyses with high support. All specimens are less than 1% different in COI sequence from each other and are greater than 5% different from other members of the group. This species is the only member of the Miniforma group that occurs in the Rocky Mountains.

This species hypothesis is supported by non-overlapping distributions, low COI divergence within the species, and high divergence between species (3–15%), and by the morphological characters outlined in the diagnosis.

*Torrenticola rockyensis* groups with other members of the Miniforma Complex with high support and specimens of this species are less than 1% different in COI sequence from each other. In all analyses, *T. rockyensis* groups with three other morphologically similar species: *T. pacificensis*, *T. manni*, and *T. copipalpa*. *Torrenticola rockyensis* is greater than 5% different from the other three species in COI sequence and is the only one of the four to occur in the Rocky Mountains.

Based upon overall similarity, the pedipalp genu extensions, and western distribution, we were able to place this species in the Miniforma Identification Group.

This species hypothesis is supported by biogeography, low COI divergence within the species (0–2%) and high divergence between species (3–15%), and by the morphological characters outlined in the diagnosis.

####### Torrenticola rufoalba

Taxon classificationAnimaliaTrombidiformesTorrenticolidae

Habeeb, 1955

Torrenticola anomala rufoalba Habeeb, 1955: 2.
Torrenticola rufoalba Habeeb, 1957: 5.

######## Material examined.

HOLOTYPE (♂): from USA, New Jersey, Morris County, Brook, Brookside, 20 May 1953, by H Habeeb.

PARATYPES (1 ♀ and 0 ♂): **New Jersey, USA**: 1 ♀ (ALLOTYPE) from Sussex County, Little Flatbrook, north of Bevans, 12 Oct 1953, by H Habeeb.

######## Type deposition.

Holotype (♂) and allotype (♀) deposited in the CNC.

######## Diagnosis.

*Torrenticola rufoalba* are similar to other members of the Rusetria “4-Plates” group (*T. dunni*, *T. glomerabilis*, *T. kittatinniana*, *T. pollani*, and *T. shubini*) and *T. skvarlai* in having anterio-lateral platelets free from the dorsal plate, dorsal coloration separated into anterior and posterior portions, and indistinct hind coxal margins. Male *T. rufoalba* can be differentiated from other male Rusetria four plates by having a shorter anterior venter (195 in *T. rufoalba*, 215–285 in others). *T. rufoalba* can be differentiated from *T. dunni* by having a shorter dorsum (♀ = 550 in *T. rufoalba*, 605–680 in *T. dunni*; ♂ = 440 in *T. rufoalba*, 500–540 in *T. dunni*) and a thinner dorsum (♀ = 400 in *T. rufoalba*, 440–490 in *T. dunni*; ♂ = 320 in *T. rufoalba*, 350–370 in *T. dunni*). *T. rufoalba* can be differentiated from *T. pollani* by having a stockier rostrum (length/width = 2.96–3.06 in *T. rufoalba*, 3.27–3.82 in *T. pollani*). Female *T. rufoalba* can be differentiated from female *T. pollani* by having stockier tibiae (length/width = 3.5 in *T. rufoalba*, 3.8–4.2 in *T. pollani*). *T. rufoalba* can be differentiated from *T. shubini* by having a more elongate rostrum (length/width = 2.96–3.06 in *T. rufoalba*, 2.24–2.92 in *T. shubini*). Female *T. rufoalba* can be differentiated from female *T. shubini* by having a wider genital field (157.5 in *T. rufoalba*, 137–145 in *T. shubini*). *T. rufoalba* can be differentiated from *T. glomerabilis* and by having more elongate anterio-medial platelets (length/width = 2.45–2.61 in *T. rufoalba*, 1.9–2.3 in *T. glomerabilis*) and thinner dorsum (♀ = 400 in *T. rufoalba*, 460–490 in *T. glomerabilis*; ♂ = 320 in *T. rufoalba*, 395–430 in *T. glomerabilis*). *T. rufoalba* can be differentiated from *T. kittatinniana* by having a shorter dorsum (♀ = 550 in *T. rufoalba*, 640 in *T. kittatinniana*; ♂ = 440 in *T. rufoalba*, 500 in *T. kittatinniana*) and stockier anterio-medial platelets (length/width = 2.45–2.61 in *T. rufoalba*, 2.83–2.88 in *T. kittatinniana*). *T. rufoalba* can be differentiated from *T. skvarlai* by having a conical pedipalpal femoral tubercle, whereas *T. skvarlai* has a broad and flat pedipalpal femoral tubercle.

######## Re-description.

**Female (Figure 229)** (n = 1) (allotype only) with characters of the genus with following specifications.

**Dorsum** — (550 long; 400 wide) ovoid with reddish-purple coloration separated into anterior and posterior portions with a strip of orange medially. Anterio-medial platelets (107.5 long; 41.25 wide). Anterio-lateral platelets (168.75 long; 55 wide) free from dorsal plate. Dgl-4 closer to the edge of the dorsum than to the muscle scars (distance between Dgl-4 255). Dorsal plate proportions: dorsum length/width 1.38; dorsal width/distance between Dgl-4 1.57; anterio-medial platelet length/width 2.61; anterio-lateral platelet length/width 3.07; anterio-lateral/anterio-medial length 1.57.

**Gnathosoma — Subcapitulum** (310 long (ventral); 235 long (dorsal); 127.5 tall) colorless. Rostrum (130 long; 42.5 wide). Chelicerae (315 long) with curved fangs (62.5 long). Subcapitular proportions: ventral length/height 2.43; rostrum length/width 3.06. **Pedipalps** with tuberculate ventral extensions on femora and genua. Palpomeres: trochanter (42.5 long); femur (115 long); genu (65 long); tibia (87.5 long; 25 wide); tarsus (17.5 long). Palpomere proportions: femur/genu 1.77; tibia/femur 0.76; tibia length/width 3.50.

**Venter** — (640 long; 450 wide) mostly colorless with reddish-purple coloration in areas surrounding coxae. Gnathosomal bay (142.5 long; 92.5 wide). Cxgl-4 subapical. **Medial suture** (17.5 long). **Genital plates** (167.5 long; 155 wide). Additional measurements: Cx-1 (125 long (total); 125 long (medial)); Cx-3 (335 wide); anterior venter (155 long). Ventral proportions: gnathosomal bay length/width 1.54; anterior venter/genital field length 0.93; anterior venter length/genital field width 1.00; anterior venter/medial suture 8.86.

**Male (Figure 230)** (n = 1) (holotype only) with characters of the genus with following specifications.

**Dorsum** — (440 long; 320 wide) ovoid with reddish-purple coloration separated into anterior and posterior portions with a strip of orange medially. Anterio-medial platelets (95 long; 38.75 wide). Anterio-lateral platelets (142.5 long; 47.5 wide) free from dorsal plate. Dgl-4 closer to the edge of the dorsum than to the muscle scars (distance between Dgl-4 237.5). Dorsal plate proportions: dorsum length/width 1.38; dorsal width/distance between Dgl-4 1.35; anterio-medial platelet length/width 2.45; anterio-lateral platelet length/width 3.00; anterio-lateral/anterio-medial length 1.50.

**Gnathosoma — Subcapitulum** (257.5 long (ventral); 190 long (dorsal); 95 tall) colorless. Rostrum (103.75 long; 35 wide). Chelicerae (237.5 long) with curved fangs (50 long). Subcapitular proportions: ventral length/height 2.71; rostrum length/width 2.96. **Pedipalps** with tuberculate ventral extensions on femora and genua. Palpomeres: trochanter (35 long); femur (92.5 long); genu (55 long); tibia (76.25 long; 21.25 wide); tarsus (17.5 long). Palpomere proportions: femur/genu 1.68; tibia/femur 0.82; tibia length/width 3.59.

**Venter** — (530 long; 400 wide) mostly colorless with reddish-purple coloration in areas surrounding coxae. Gnathosomal bay (120 long; 67.5 wide). Cxgl-4 subapical. **Medial suture** (72.5 long). **Genital plates** (120 long; 97.5 wide). Additional measurements: Cx-1 (235 long (total); 112.5 long (medial)); Cx-3 (280 wide); anterior venter (195 long). Ventral proportions: gnathosomal bay length/width 1.78; anterior venter/genital field length 1.63; anterior venter length/genital field width 2.00; anterior venter/medial suture 2.69.

**Immatures** unknown.

######## Etymology.

Habeeb (1955) did not specify an etymology for this specific epithet (*rufoalba*). However, we speculate that it refers to the dorsal coloration as Habeeb (1955) notes, “color red and white” (*rufus*, L. red; *albus*, L. white).

######## Distribution.

**Figure 228.**
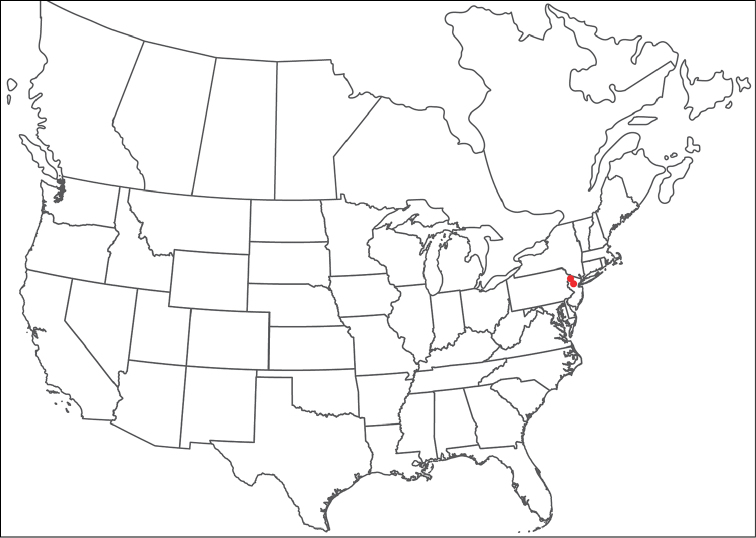
*Torrenticola rufoalba* distribution.

Northern New Jersey (Figure 228).

**Figure 229.**
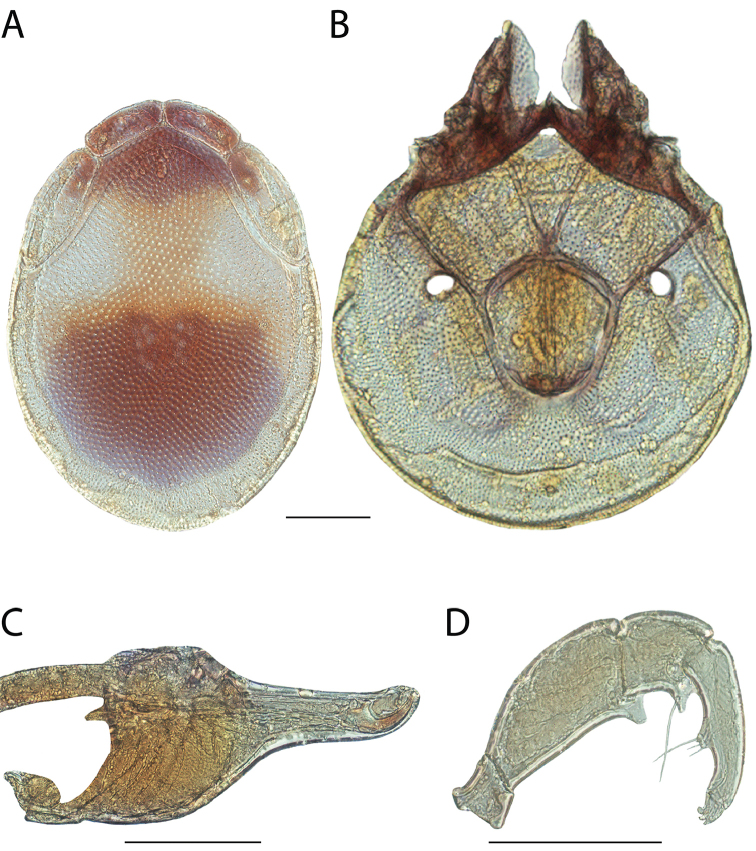
*Torrenticola rufoalba* female: **A** dorsal plates **B** venter (legs removed) **C** subcapitulum **D** pedipalp (setae not accurately depicted). Scale = 100 µm.

**Figure 230.**
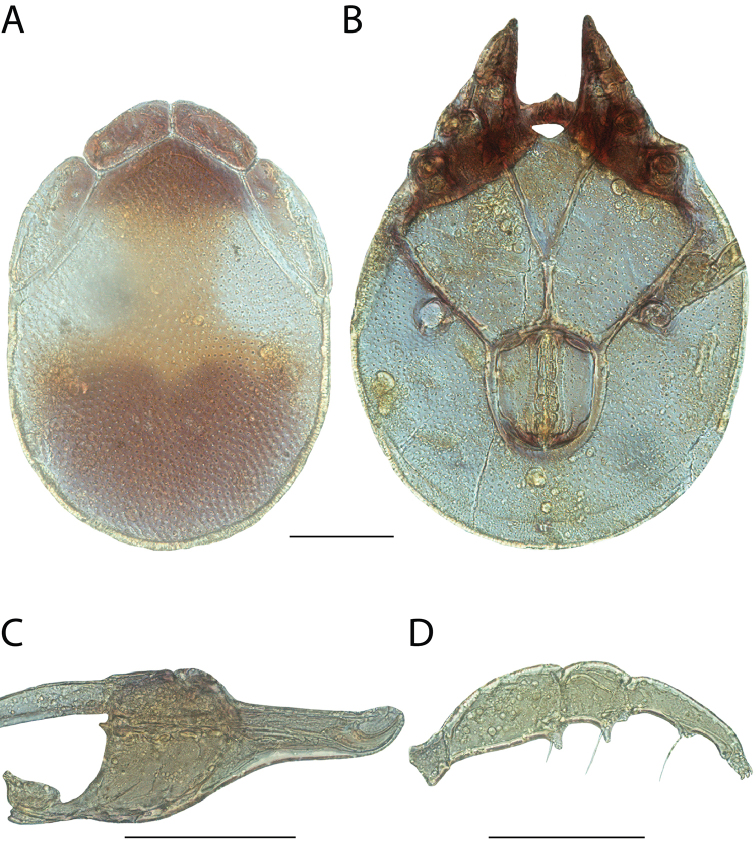
*Torrenticola rufoalba* male: **A** dorsal plates **B** venter (legs removed) **C** subcapitulum **D** pedipalp (setae not accurately depicted). Scale = 100 µm.

######## Remarks.

Unfortunately, we were unable to acquire more specimens of *Torrenticola rufoalba* and therefore this species is not included in our phylogenetic analyses. However, we were able to examine the holotype. Based upon overall similarity, the fusion of the posterio-lateral platelets, and distribution, this species clearly groups with the Rusetria Complex and can be placed into the Eastern 2-Plate Identification Group.

####### Torrenticola sellersorum

Taxon classificationAnimaliaTrombidiformesTorrenticolidae

Fisher & Dowling sp. n.

http://zoobank.org/E42D30E2-9FF8-4BFD-8431-7F20BB2AD9E0

######## Material examined.

HOLOTYPE (♀): from USA, Pennsylvania, Fayette County, Ohiopyle State Park, Laurel Run (39°50'58"N, 79°30'51"W), 10 Aug 2014, by MJ Skvarla, MS 14-0810-005, DNA 2831.

PARATYPES (18 ♀; 8 ♂): **Manitoba, Canada**: 1 ♀ and 1 ♂ from Eating Point Creek, 0.3 kilometers east of Highway 6 (53°15'7"N, 99°18'54"W), 4 Jul 2009, by IM Smith, IMS090030A • 1 ♀ and 1 ♂ from Ochre River, beside Highway 5 (51°3'N, 99°46'W), 6 Sep 1970, by DW Barr & H Frania, ROM700638 • **New Mexico, USA**: 2 ♀ from Catron County, Whitewater Creek, Glenwood Whitewater Picnic Area, 5 May 2012, by IM Smith, IMS120005 • 1 ♀ from Grant County, east fork of Gila River, Grapevine Recreation Area off Route 15 north of Silver City, 5 May 2012, by IM Smith, IMS120007 • **Ohio, USA**: 2 ♀ and 1 ♂ from Hocking County, beside road near Ash Cave, East Branch of Queer Creek (39°24'N, 82°33'W), 5 May 1993, by IM Smith & DR Cook, IMS930001A • **Pennsylvania, USA**: 1 ♂ (ALLOTYPE) from Fayette County, Ohiopyle State Park, Laurel Run (39°50'58"N, 79°30'51"W), 10 Aug 2014, by MJ Skvarla, MS 14-0810-005, DNA 2835 • 1 ♂ from Fayette County, Ohiopyle State Park, Laurel Run (39°50'58"N, 79°30'51"W), 10 Aug 2014, by MJ Skvarla, MS 14-0810-005• 2 ♀ from Westmoreland County, Irwin Park (40°19'38"N, 79°42'30"W), 4 Aug 2014, by MJ Skvarla, MS 14-0804-004 • 4 ♀ from Somerset County, Laurel Hill State Park Laurel Hill Creek (40°1'6"N, 79°14'4"W), 8 Aug 2014, by MJ Skvarla, MS 14-0808-001 • **Saskatchewan, Canada**: 2 ♀ and 1 ♂ from Smeaton Torch River, beside Highway 106, 26.2 km north of Highway 55, 20 Aug 2012, by IM Smith, IMS120079 • **South Dakota, USA**: 2 ♀ and 2 ♂ from Pennington County, Willow Creek Trail Head off Route 244, Willow Creek (43°54'N, 103°32'W), 10 Sep 1999, by IM Smith, IMS990033.

######## Type deposition.

Holotype (♀), allotype (♂), and other paratypes (13 ♀; 4 ♂) deposited in the CNC; other paratypes (5 ♀; 3 ♂) deposited in ACUA.

######## Diagnosis.

*Torrenticola sellersorum* are similar to other members of the Rusetria “Eastern 2-Plates” group (*T. biscutella*, *T. caerulea*, *T. delicatexa*, *T. indistincta*, *T. malarkeyorum*, *T. pendula*, *T. tysoni*, *T. ululata*, *T. whitneyae*, *T. microbiscutella*, and *T. feminellai*) in having anterio-lateral platelets fused to the dorsal plate, having dorsal coloration separated into anterior and posterior portions (except *T. ululata* and *T. indistincta*), and being distributed in the east. It is one of only four Eastern 2-Plates that have dark, bold, bluish-purple coloration (also *T. tysoni*, *T. biscutella*, and *T. pendula*). *T. sellersorum* can be differentiated from *T. ululata*, *T. indistincta*, and *T. feminellai* by dorsal coloration and pattern. *T. sellersorum* can be differentiated from *T. tysoni* by having a stockier rostrum (length/width ♀ = 2.44–2.68 in *T. sellersorum*, 3.06–3.31 in *T. tysoni*; ♂ = 2.71–3.05 in *T. sellersorum*, 3.14–3.50 in *T. tysoni*). Female *T. sellersorum* can be differentiated from female *T. biscutella* by anterior venter/genital field length (0.69–0.77 in *T. sellersorum*, 0.82–0.88 in *T. biscutella*). Male *T. sellersorum* can be differentiated from male *T. biscutella* by having slightly more elongate anterio-lateral platelets (length/width = 2.76–3.00 in *T. sellersorum*, 2.58–2.74 in *T. biscutella*). *T. sellersorum* can be differentiated from *T. pendula* by having a stockier gnathosomal bay (length/width = 1.56–2.08 in *T. sellersorum*, 2.42–2.90 in *T. pendula*) and more elongate tibiae (length/width = 3.13–3.8 in *T. sellersorum*, 2.78–3.05 in *T. pendula*). *T. sellersorum* can be differentiated from *T. whitneyae* by having more elongate pedipalpal tibiae (length/width = 3.13–3.80 in *T. sellersorum*, 2.42–2.95 in *T. whitneyae*). *T. sellersorum* can be differentiated from *T. microbiscutella* by having a less elongate dorsum (length/width = 1.23–1.54 in *T. sellersorum*, 1.63–1.75 in *T. microbiscutella*). Female *T. sellersorum* can be differentiated from female *T. malarkeyorum* by anterior venter/genital field length (0.69–0.77 in *T. sellersorum*, 0.85–0.89 in *T. malarkeyorum*). Female *T. sellersorum* can be differentiated from female *T. caerulea* by having a wider genital field (150–182 in *T. sellersorum*, 120–145 in *T. caerulea*). Female *T. sellersorum* can be differentiated from female *T. delicatexa* by having a rounder dorsum (length/width = 1.23–1.37 in *T. sellersorum*, 1.38–1.44 in *T. delicatexa*). Male *T. sellersorum* do not have any measurement differences with male *T. malarkeyorum*, *T. caerulea*, and *T. delicatexa*; however, they can be differentiated by dorsal coloration.

######## Description.

**Female (Figure 232)** (n = 5) (holotype measurements in parentheses when available) with characters of the genus with following specifications.

**Dorsum** — (540–650 (540) long; 400–520 (400) wide) ovoid with bold (occasionally faint) bluish-purple coloration separated into anterior and posterior portions with a thin or thick strip of red medially. Anterio-medial platelets (112.5–135 (117.5) long; 35–50 (35) wide). Anterio-lateral platelets (147.5–172.5 (147.5) long; 47.5–75 (47.5) wide) fused to dorsal plate. Dgl-4 much closer to the edge of the dorsum than to the muscle scars (distance between Dgl-4 290–370 (295)). Dorsal plate proportions: dorsum length/width 1.23–1.37 (1.35); dorsal width/distance between Dgl-4 1.35–1.42 (1.36); anterio-medial platelet length/width 2.65–3.36 (3.36); anterio-lateral platelet length/width 2.23–3.11 (3.11); anterio-lateral/anterio-medial length 1.26–1.36 (1.26).

**Gnathosoma — Subcapitulum** (282.5–330 (285) long (ventral); 210–245 (219) long (dorsal); 130–157.5 (135) tall) colorless. Rostrum (110–125 (113.75) long; 42.5–47.5 (42.5) wide). Chelicerae 280–325 (280) long) with curved fangs (51–66 (62) long). Subcapitular proportions: ventral length/height 2.05–2.17 (2.11); rostrum length/width 2.44–2.68 (2.68). **Pedipalps** with tuberculate ventral extensions on femora and genua. Palpomeres: trochanter (40–50 (42.5) long); femur (107.5–122.5 (110) long); genu (62.5–72.5 (65) long); tibia (82.5–95 (85) long; 22.5–27.5 (22.5) wide); tarsus (17.5–20 (20) long). Palpomere proportions: femur/genu 1.69–1.85 (1.69); tibia/femur 0.75–0.79 (0.77); tibia length/width 3.14–3.80 (3.78).

**Venter** — (640–800 (645) long; 449–529 (470) wide) colorless. Gnathosomal bay (132.5–187.5 (157.5) long; 82.5–97.5 (82.5) wide). Cxgl-4 subapical. **Medial suture** (0–10 (0) long) often absent. **Genital plates** (160–195 (165) long; 150–181.25 (157.5) wide). Additional measurements: Cx-1 (230–322 (270) long (total); 83–126 (106) long (medial)); Cx-3 (310–365 (319) wide); anterior venter (120–142.5 (120) long). Ventral proportions: gnathosomal bay length/width 1.56–2.08 (1.91); anterior venter/genital field length 0.69–0.77 (0.73); anterior venter length/genital field width 0.74–0.80 (0.76); anterior venter/medial suture (proportion cannot be calculated for specimens without a medial suture) 12.25–17.00.

**Male (Figure 233)** (n = 5) (allotypic measurements in parentheses when available) with characters of the genus with following specifications.

**Dorsum** — (390–470 (400) long; 255–330 (280) wide) ovoid with bold (occasionally faint) bluish-purple coloration separated into anterior and posterior portions with a thin or thick strip of red medially. Anterio-medial platelets (82.5–97.5 (82.5) long; 30–40 (30) wide). Anterio-lateral platelets (112.5–145 (120) long; 37.5–52.5 (42.5) wide) fused to dorsal plate. Dgl-4 much closer to the edge of the dorsum than to the muscle scars (distance between Dgl-4 190–250 (220)). Dorsal plate proportions: dorsum length/width 1.42–1.54 (1.43); dorsal width/distance between Dgl-4 1.27–1.34 (1.27); anterio-medial platelet length/width 2.44–2.83 (2.75); anterio-lateral platelet length/width 2.76–3.00 (2.82); anterio-lateral/anterio-medial length 1.32–1.49 (1.45).

**Gnathosoma — Subcapitulum** (205–245 (215) long (ventral); 151–179 (166) long (dorsal); 80–107.5 (91.25) tall) colorless. Rostrum 80–95 (85) long; 27.5–35 (30) wide). Chelicerae (195–235 (208) long) with curved fangs (40–50 (46) long). Subcapitular proportions: ventral length/height 2.24–2.59 (2.36); rostrum length/width 2.71–3.05 (2.83). **Pedipalps** with tuberculate ventral extensions on femora and genua. Palpomeres: trochanter (30–38.75 (30) long); femur (75–87.5 (80) long); genu (46.25–52.5 (50) long); tibia (62.5–70 (67.5) long; 18.75–21.25 (20) wide); tarsus (15–17.5 (15) long). Palpomere proportions: femur/genu 1.60–1.67 (1.60); tibia/femur 0.79–0.87 (0.84); tibia length/width 3.13–3.47 (3.38).

**Venter** — (465–570 (490) long; 290–367 (325) wide) colorless. Gnathosomal bay (105–135 (117.5) long; 57.5–66.25 (65) wide). Cxgl-4 subapical. **Medial suture** (57.5–72.5 (57.5) long). **Genital plates** (102.5–122.5 (107.5) long; 91.25–115 (96.25) wide). Additional measurements: Cx-1 (189–237 (199) long (total); 79–96 (85) long (medial)); Cx-3 (230–290 (232) wide); anterior venter (150–185 (150) long). Ventral proportions: gnathosomal bay length/width 1.75–2.08 (1.81); anterior venter/genital field length 1.39–1.59 (1.40); anterior venter length/genital field width 1.55–1.78 (1.56); anterior venter/medial suture 2.32–2.61 (2.61).

**Immatures** unknown.

######## Etymology.

Specific epithet (*sellersorum*) named in honor of the grandparents of DMF, who always supported and encouraged her love of nature and discovery.

######## Distribution.

**Figure 231.**
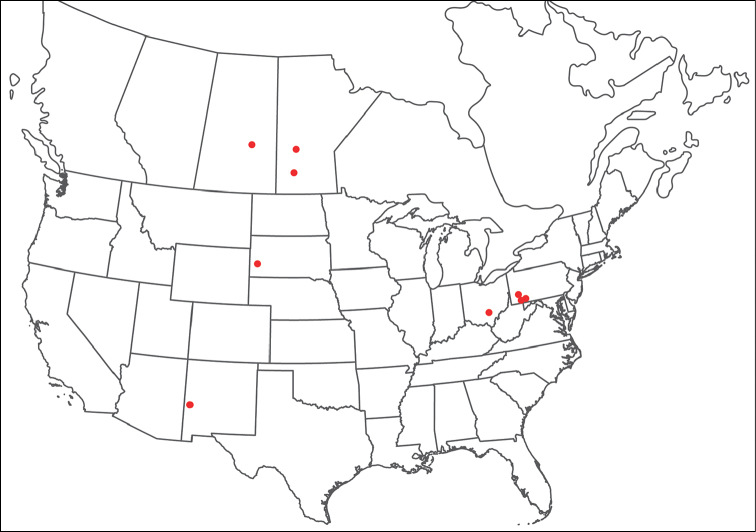
*Torrenticola sellersorum* sp. n. distribution.

Widespread (Figure 231). *T. sellersorum* are unlike all other *Torrenticola* in our collections in that they span either side of the Great Plains, which act as a barrier for most other species. *T. sellersorum* are likely absent from cool, highland streams of the Appalachians, Interior Highlands, and Rocky Mountains, but are likely widely distributed throughout the Interior. Explaining the peculiar distribution of *T. sellersorum* will depend on knowledge of dispersal capabilities, including host preference by the larvae and the dispersal capabilities of the host.

**Figure 232.**
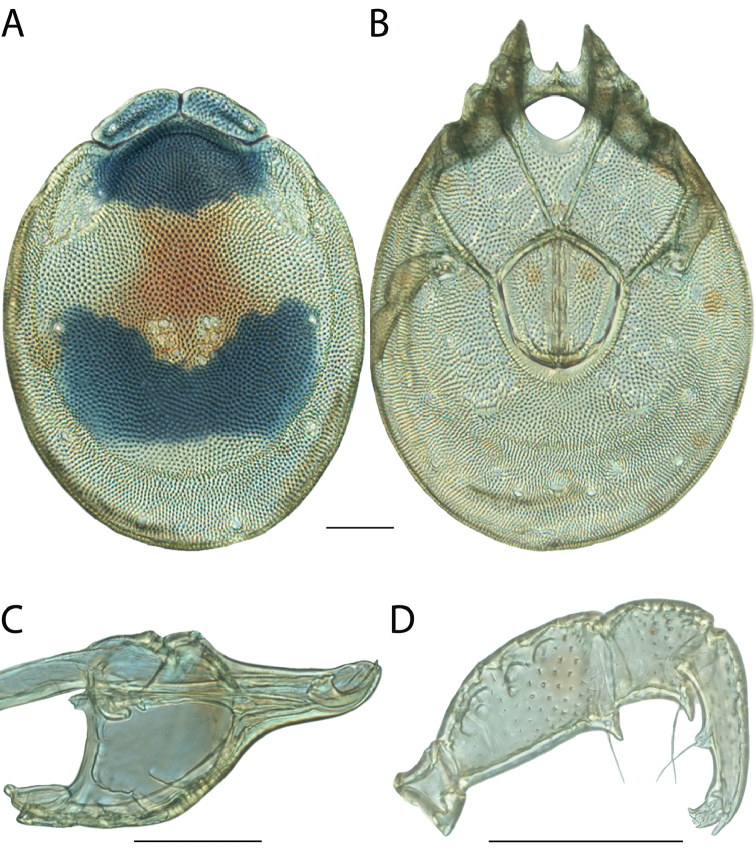
*Torrenticola sellersorum* sp. n. female: **A** dorsal plates **B** venter (legs removed) **C** subcapitulum **D** pedipalp (setae not accurately depicted). Scale = 100 µm.

**Figure 233.**
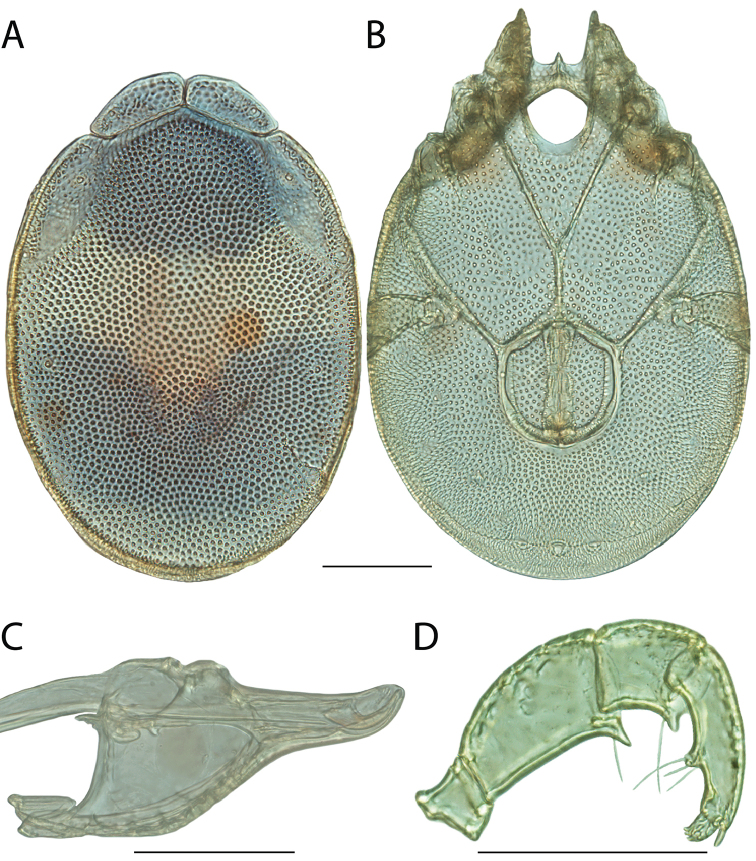
*Torrenticola sellersorum* sp. n. male: **A** dorsal plates **B** venter (legs removed) **C** subcapitulum **D** pedipalp (setae not accurately depicted). Scale = 100 µm.

######## Remarks.

In all analyses, *Torrenticola sellersorum* groups with other members of the Rusetria Complex with high support and specimens of this species are less than 2.3% different in COI sequence from each other. In all analysis, *T. sellersorum* groups with other eastern members of the Rusetria Complex that have fused lateral platelets. Because of this, we are able to place this species in the Eastern 2-Plate Identification Group.

The slightly increased COI variation of this species (2.3%) is expected for specimens separated by great geographic distance. For example, the specimens from Pennsylvania are 0–1.3% different from each other and the specimens from Saskatchewan are 0.3% different from each other, but Pennsylvania specimens are 1.3–2.0% different from the Saskatchewan specimens. However, even specimens from New Mexico are only 2.0–2.3% different from other localities, despite being separated by great geographic distance and ecoregions that act as barriers for other *Torrenticola* (i.e., the Great Plains). This variation in COI in the New Mexico samples supports two hypotheses: 1) given the relatively low divergence of approximately 2%, New Mexico specimens represent the same species as specimens further east; and conversely, 2) given the relatively high divergence of greater than 1%, New Mexico specimens represent a long-standing population, not a recent introduction from the east.

This species hypothesis is supported by low COI divergence within the species (0–2.3%) (but see above discussion) and high divergence between species (3–15%), and by the morphological characters outlined in the diagnosis.

####### Torrenticola sharkeyi

Taxon classificationAnimaliaTrombidiformesTorrenticolidae

Fisher & Dowling sp. n.

http://zoobank.org/4785F071-6FDF-4732-A1D2-67B6CDD0CB86

######## Material examined.

HOLOTYPE (♀): from USA, New Mexico, Catron County, Gila Hot Springs, Little Creek Recreational Area, Little Creek, off Route 15, 6 May 2012, by IM Smith, IMS120006, DNA 2891.

PARATYPES (13 ♀; 11 ♂): **Arizona, USA**: 2 ♀ and 2 ♂ from Coconino County, Oak Creek Canyon, Oak Creek, beside Route 89A, between Banjo Bill & Bootlegger campgrounds, 21 Jul 1987, by IM Smith, IMS870100A & IMS870100B • 1 ♀ and 1 ♂ from Yavapai County, West Clear Creek at Clear Creek campground, off Forest Road 9, east of Camp Verde, 19-22 Jul 1987, by IM Smith, IMS870098 & IMS870102 • **New Mexico, USA**: 1 ♀ and 1 ♂ from Catron County, Gila Cliff Dwellers National Monument, West Fork of Gila River, 10 Jul 1987, by IM Smith, IMS870080A • 1 ♂ (ALLOTYPE) from Catron County, Gila Hot Springs, Little Creek, Little Creek Recreational Area off Route 15, 6 May 2012, by IM Smith, IMS120006, DNA 2892 • 1 ♀ and 1 ♂ from Catron County, Gila River, beside Route 15, just below mouth of Little Creek, 11 Jul 1987, by IM Smith, IMS870083A • 2 ♀ and 1 ♂ from Catron County, Gila Hot Springs, Little Creek, Little Creek Recreational Area off Route 15, 6 May 2012, by IM Smith, IMS120006 • 2 ♀ and 3 ♂ from Catron County, Little Creek, beside Route 15, 65 kilometers north of Route 180 in Silver City, 10 Jul 1987, by IM Smith, IMS870081A • 3 ♀ and 2 ♂ from Grant County, Gila River Recreation Area, East Fork of Gila River, beside Route 15, 11 Jul 1987, by IM Smith, IMS870082A • 1 ♀ and 1 ♂ from Grant County, Grapevine Recreational Area, East Fork of Gila River, off Route 15 north of Silver City, 5 May 2012, by IM Smith, IMS120007.

######## Type deposition.

Holotype (♀), allotype (♂), and most paratypes (8 ♀; 5 ♂) deposited in the CNC; other paratypes (5 ♀; 5 ♂) deposited in ACUA.

######## Diagnosis.

*Torrenticola sharkeyi* is unlike all other western species by having the following combination of characters: dorsal coloration, although faint, restricted posteriorly; indistinct hind coxal margins; and ellipsoid body. It is only known from Catron & Grant Counties, New Mexico. It is most similar to members of the Rala Group (*T. rala*, *T. keesdavidsi*, *T. kurtvietsi*, *T. boettgeri*, *T. lamellipalpis*, *T. dolichodactyla*, and *T. anoplopalpa*); however, members of this group are colorless. *T. sharkeyi* can be differentiated from *T. boettgeri*, *T. kurtvietsi*, *T. lamellipalpis*, *T. keesdavidsi*, and *T. anoplopalpa* by having tuberculate ventral extensions on the pedipalpal femora (others are lacking extensions, have dentate flanged extensions or wide lamellate extensions). *T. sharkeyi* can be differentiated from *T. rala*, *T. kurtvietsi*, *T. boettgeri*, *T. dolichodactyla* and *T. anoplopalpa* by having a more elongate pedipalpal tibiae (length/width = 3.81–4.10 in *T. sharkeyi*, 1.75–3.38 in others). *T. sharkeyi* can be differentiated from *T. kurtvietsi*, *T. keesdavidsi*, *T. lamellipalpis*, and *T. anoplopalpa* by having a stockier subcapitulum (ventral length/width = 2.03–2.40 in *T. sharkeyi*, 2.51–4.16 in others).

######## Description.

**Female (Figure 235)** (n = 5) (holotype measurements in parentheses when available) with characters of the genus with following specifications.

**Dorsum** — (650–750 (715) long; 450–530 (490) wide) ovoid with faint bluish-purple coloration restricted posteriorly or colorless. Anterio-medial platelets (130–155 (137.5) long; 50–57.5 (52.5) wide). Anterio-lateral platelets (187.5–225 (202.5) long; 62.5–75 (67.5) wide) free from dorsal plate. Dgl-4 closer to the edge of the dorsum than to the muscle scars (distance between Dgl-4 305–360 (335)). Dorsal plate proportions: dorsum length/width 1.41–1.46 (1.46); dorsal width/distance between Dgl-4 1.38–1.52 (1.46); anterio-medial platelet length/width 2.60–2.77 (2.62); anterio-lateral platelet length/width 2.91–3.08 (3.00); anterio-lateral/anterio-medial length 1.39–1.47 (1.47).

**Gnathosoma — Subcapitulum** (300–335 (310) long (ventral); 215–245 (227) long (dorsal); 135–157.5 (152.5) tall) colorless. Rostrum (117.5–137.5 (125) long; 45–55 (46.25) wide). Chelicerae (290–340 (314) long) with curved fangs (62.5–70 (68) long). Subcapitular proportions: ventral length/height 2.03–2.25 (2.03); rostrum length/width 2.45–2.89 (2.70). **Pedipalps** with short tuberculate ventral extensions on femora and genua. Palpomeres: trochanter (42.5–50 (50) long); femur (112.5–132.5 (125) long); genu (66.25–75 (68.75) long); tibia (100–107.5 (105) long; 25–27.5 (26.25) wide); tarsus (23.75–27.5 (27.5) long). Palpomere proportions: femur/genu 1.67–1.83 (1.82); tibia/femur 0.81–0.91 (0.84); tibia length/width 3.91–4.10 (4.00).

**Venter** — (730–880 (870) long; 513–610 (543) wide) colorless. Gnathosomal bay (145–186.25 (181.25) long; 92.5–105 (105) wide). Cxgl-4 apical. **Medial suture** (20–25 (20) long). **Genital plates** (177.5–200 (200) long; 157.5–180 (167.5) wide). Additional measurements: Cx-1 (280–350 (323) long (total); 125–160 (140) long (medial)); Cx-3 (365–405 (365) wide); anterior venter (167.5–186.25 (181.25) long). Ventral proportions: gnathosomal bay length/width 1.57–1.84 (1.73); anterior venter/genital field length 0.91–0.97 (0.91); anterior venter length/genital field width 1.03–1.08 (1.08); anterior venter/medial suture 7.30–9.25 (9.06).

**Male (Figure 236)** (n = 5) (allotypic measurements in parentheses when available) with characters of the genus with following specifications.

**Dorsum** — (580–650 (650) long; 415–470 (470) wide) ovoid with faint bluish-purple coloration restricted posteriorly or colorless. Anterio-medial platelets (125–137.5 (137.5) long; 40–60 (60) wide). Anterio-lateral platelets (180–197.5 (197.5) long; 62.5–82.5 (82.5) wide) free from dorsal plate. Dgl-4 closer to the edge of the dorsum than to the muscle scars (distance between Dgl-4 280–320 (320)). Dorsal plate proportions: dorsum length/width 1.36–1.44 (1.38); dorsal width/distance between Dgl-4 1.46–1.49 (1.47); anterio-medial platelet length/width 2.29–3.13 (2.29); anterio-lateral platelet length/width 2.39–2.96 (2.39); anterio-lateral/anterio-medial length 1.37–1.51 (1.44).

**Gnathosoma — Subcapitulum** (250–300 (300) long (ventral); 195–216 (215.75) long (dorsal); 105–127.5 (127.5) tall) colorless. Rostrum (100–119 (118.75) long; 37.5–50 (42.5) wide). Chelicerae (270–303 (303) long) with curved fangs (46–50 (47) long). Subcapitular proportions: ventral length/height 2.31–2.40 (2.35); rostrum length/width 2.30–2.91 (2.79). **Pedipalps** with short tuberculate ventral extensions on femora and genua. Palpomeres: trochanter (45–47.5 (47.5) long); femur (102.5–115 (110) long); genu (57.5–67.5 (67.5) long); tibia (88–104 (103.75) long; 22.5–27 (26.25) wide); tarsus (22.5–27.5 (22.5) long). Palpomere proportions: femur/genu 1.63–1.80 (1.63); tibia/femur 0.83–0.94 (0.94); tibia length/width 3.80–3.95 (3.95).

**Venter** — (670–771 (770) long; 460–520 (496) wide) colorless. Gnathosomal bay (127.5–170 (170) long; 80–90 (90) wide). Cxgl-4 apical. **Medial suture** (85–105 (100) long). **Genital plates** (177.5–202 (201.25) long; 135–147.5 (147.5) wide). Additional measurements: Cx-1 (270–315 (305) long (total); 140–157.5 (157.5) long (medial)); Cx-3 (325–370 (360) wide); anterior venter (237.5–270 (270) long). Ventral proportions: gnathosomal bay length/width 1.46–1.94 (1.89); anterior venter/genital field length 1.28–1.41 (1.34); anterior venter length/genital field width 1.70–1.93 (1.83); anterior venter/medial suture 2.43–2.79 (2.70).

**Immatures** unknown.

######## Etymology.

Specific epithet (*sharkeyi*) named in honor of braconid systematist, Michael Sharkey, who advised JRF during his master’s degree and solidified his life-long passion for taxonomy.

######## Distribution.

**Figure 234.**
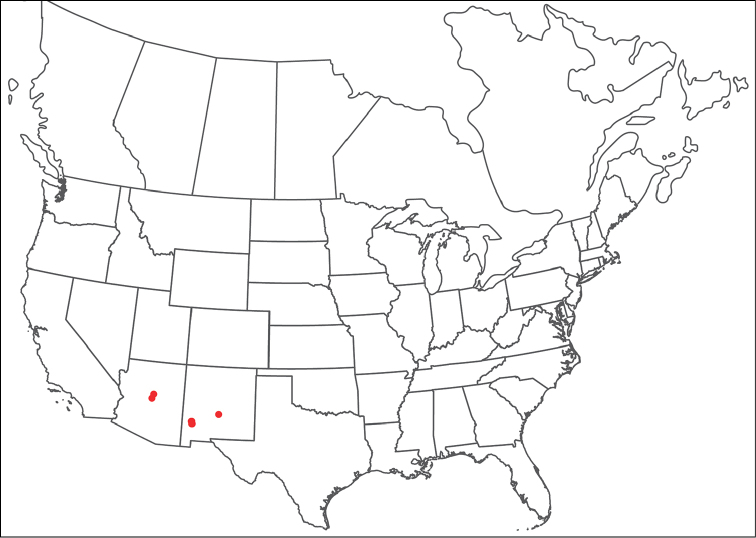
*Torrenticola sharkeyi* sp. n. distribution.

Southwestern, New Mexico and Arizona (Figure 234).

**Figure 235.**
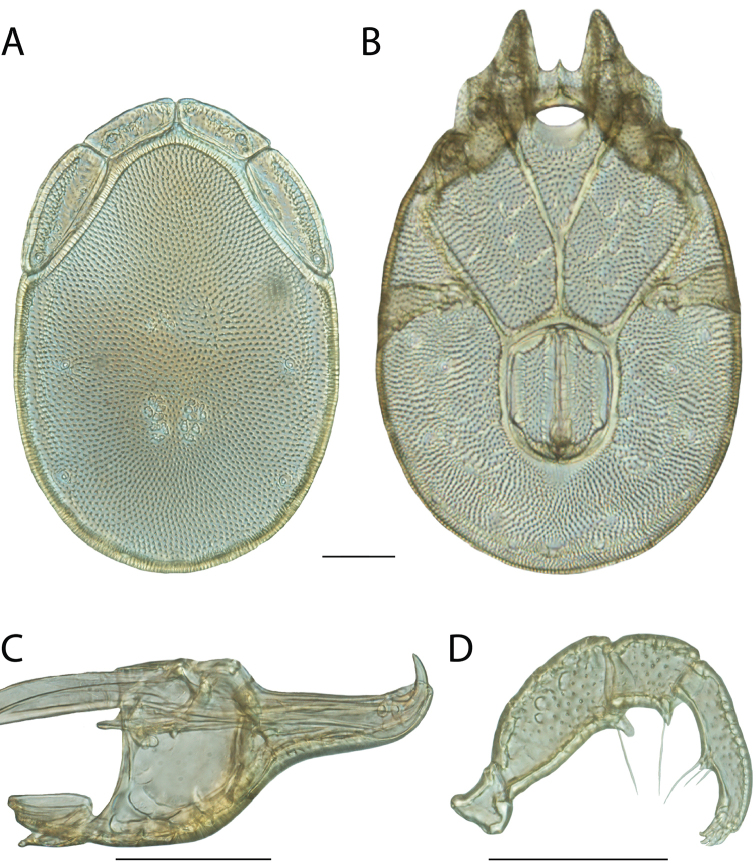
*Torrenticola sharkeyi* sp. n. female: **A** dorsal plates **B** venter (legs removed) **C** subcapitulum **D** pedipalp (setae not accurately depicted). Scale = 100 µm.

**Figure 236.**
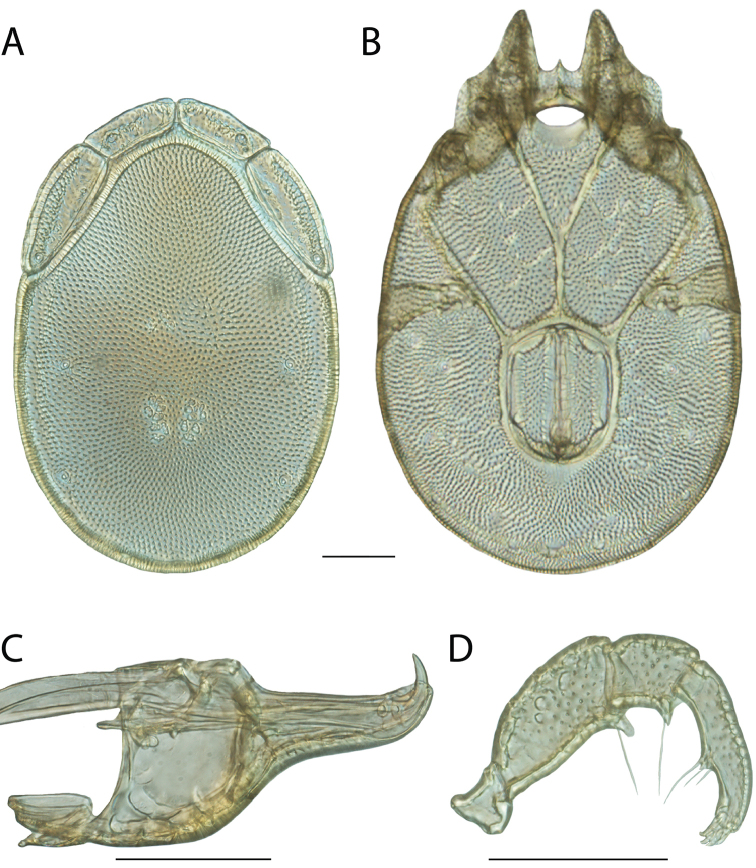
*Torrenticola sharkeyi* sp. n. male: **A** dorsal plates **B** venter (legs removed) **C** subcapitulum **D** pedipalp (setae not accurately depicted). Scale = 100 µm.

######## Remarks.

Our analyses were unable to confidently place *Torrenticola sharkeyi* phylogenetically. The COI analyses recovers this species at the base of the Raptor Complex, but this relationship was not recovered in the combined analysis. Because of this ambiguity, we refrain from placing this species in a species complex. Furthermore, because of the unique morphology of this species, we refrain from placing this species in an identification group.

All specimens are less than 2% different in COI sequence from each other and greater than 16% different from sister species.

This species hypothesis is supported by low COI divergence within the species (0–2%) and high divergence between species (greater than 3%), and by the morphological characters outlined in the diagnosis.

####### Torrenticola shubini

Taxon classificationAnimaliaTrombidiformesTorrenticolidae

isher & Dowling sp. n.

http://zoobank.org/160D26D4-7B82-4C2E-A02D-B7CFD114839B

######## Material examined.

HOLOTYPE (♀): USA, Tennessee, Sevier County, Great Smokey Mountains National Park, middle prong Little Pigeon River (35°44'12"N, 83°24'51"W), 12 Sep 2010, by IM Smith, IMS100132.

PARATYPES (5 ♀; 5 ♂): **Pennsylvania, USA**: 1 ♂ (ALLOTYPE) from Somerset County, Laurel Hill State Park, Laurel Hill Creek (40°1'6"N, 79°14'4"W), 8 Aug 2014, by MJ Skvarla, MS 14-0808-001, DNA 2845 • **Tennessee, USA**: 1 ♀ from Blount County, Great Smokey Mountains National Park, Abrams River (35°35'31"N, 83°51'21"W), 17 Sep 2010, by IM Smith, IMS100141 • 2 ♀ from Monroe County, Turkey Creek (35°21'47"N, 84°9'47"W), 12 Sep 2009, by IM Smith, IMS090112 • 1 ♂ from Sevier County, Great Smokey Mountains National Park, middle prong Little Pigeon River (35°43'33"N, 83°24'1"W), 12 Sep 2010, by IM Smith, IMS100131 • 1 ♂ from Sevier County, Great Smokey Mountains National Park, middle prong Little Pigeon River (35°44'12"N, 83°24'51"W), 12 Sep 2010, by IM Smith, IMS100132 • **Vermont, USA**: 2 ♀ from Addison County, Lincoln, beside Forest Service Road #54, New Haven River (44°6'N, 72°59'W), 6 Jul 1989, by IM Smith, IMS890074 • **Virginia, USA**: 2 ♂ from Scott County, beside Route 58/421, 0.9 kilometers east of Route 709, North Fork of Holston River (36°39'N, 82°28'W), by IM Smith, IMS900080.

######## Type deposition.

Holotype (♀), allotype (♂), and most paratypes (3 ♀; 2 ♂) deposited in the CNC; other paratypes (2 ♀; 2 ♂) deposited in ACUA.

######## Diagnosis.

*Torrenticola shubini* are similar to other members of the Rusetria “4-Plates” group (*T. dunni*, *T. glomerabilis*, *T. kittatinniana*, *T. pollani*, and *T. rufoalba*) and *T. skvarlai* in having anterio-lateral platelets free from the dorsal plate, dorsal coloration separated into anterior and posterior portions, and indistinct hind coxal margins. Female *T. shubini* can be differentiated from female *T. dunni* by having a thinner rostrum (length/width = 2.5–2.7 in *T. shubini*, 2.8–3.0 in *T. dunni*). Male *T. dunni* can be differentiated from male *T. shubini* by having a longer anterior venter (215–238 in *T. shubini*, 277–285 in *T. dunni*). *T. shubini* can be differentiated from *T. pollani* by having stockier tibiae (length/width ♀ = 3.30–3.60 in *T. shubini*, 3.89–4.18 in *T. pollani*; ♂ = 3.11–3.22 in *T. shubini*, 3.41–3.75 in *T. pollani*) and a stockier rostrum (length/width = 2.24–2.92 in *T. shubini*, 3.27–3.82 in *T. pollani*). *T. shubini* can be differentiated from *T. glomerabilis* by having the Dgl-4 closer to the dorsal edge (dorsal width/length between Dgl-4 = 1.2–1.4 in *T. shubini*; 1.5–1.7 in *T. glomerabilis*); and stockier tibiae (length/width ♀ = 3.3–3.6 in *T. shubini*, 4.1–4.5 in *T. glomerabilis*; ♂ = 3.1–3.2 in *T. shubini*, 3.5–4.4 in *T. glomerabilis*). Female *T. shubini* can be differentiated from female *T. kittatinniana* by having a stockier rostrum (length/width = 2.5–2.7 in *T. shubini*, 3.16 in *T. kittatinniana*) and a taller subcapitulum (140–145 in *T. shubini*, 125 in *T. kittatinniana*). Male *T. shubini* can be differentiated from male *T. kittatinniana* by having a shorter dorsum (400–465 in *T. shubini*, 500 in *T. kittatinniana*) and a shorter genital field (90–108 in *T. shubini*, 115 in *T. kittatinniana*). *T. shubini* can be differentiated from *T. rufoalba* by having a stockier rostrum (length/width = 2.24–2.92 in *T. shubini*, 2.96–3.06 in *T. rufoalba*). Female *T. shubini* can be differentiated from female *T. rufoalba* by having a thinner genital field (137–145 in *T. shubini*, 157.5 in *T. rufoalba*). Male *T. shubini* can be differentiated from male *T. rufoalba* by having a longer anterior venter (215–238 in *T. pollani*, 195 in *T. rufoalba*). *T. shubini* can be differentiated from *T. skvarlai* by having a conical pedipalpal femoral tubercle, whereas *T. skvarlai* has a broad and flat pedipalpal femoral tubercle, and by having a longer anterior venter (♀ = 156–170 in *T. shubini*, 140–153 in *T. skvarlai*; ♂ = 215–238 in *T. shubini*, 177–205 in *T. skvarlai*).

######## Description.

**Female (Figure 238)** (n = 5) (holotype measurements in parentheses when available) with characters of the genus with following specifications.

**Dorsum** — (550–640 (590) long; 415–455 (430) wide) ovoid with faint bluish-purple coloration separated into anterior and posterior portions, and with faint orange medially. Anterio-medial platelets (115–125 (122.5) long; 40–47.5 (46.25) wide). Anterio-lateral platelets (160–170 (165) long; 55–60 (56.25) wide) free from dorsal plate. Dgl-4 much closer to the edge of the dorsum than to the muscle scars (distance between Dgl-4 310–335 (325)). Dorsal plate proportions: dorsum length/width 1.33–1.45 (1.37); dorsal width/distance between Dgl-4 1.32–1.42 (1.32); anterio-medial platelet length/width 2.63–2.88 (2.65); anterio-lateral platelet length/width 2.75–2.96 (2.93); anterio-lateral/anterio-medial length 1.28–1.43 (1.35).

**Gnathosoma — Subcapitulum** (300–320 (310) long (ventral); 223–255 (232) long (dorsal); 140–145 (140) tall) colorless. Rostrum (117.5–122.5 (120) long; 45–48.75 (45) wide). Chelicerae (288–320 (316) long) with curved fangs (54–63 (57) long). Subcapitular proportions: ventral length/height 2.14–2.21 (2.21); rostrum length/width 2.51–2.67 (2.67). **Pedipalps** with tuberculate ventral extensions on femora and genua. Palpomeres: trochanter (42.5–47.5 (47.5) long); femur (112.5–121.25 (117.5) long); genu (63.75–70 (66.25) long); tibia (82.5–90 (83.75) long; 23.75–25 (25) wide); tarsus (17.5–17.5 (17.5) long). Palpomere proportions: femur/genu 1.71–1.83 (1.77); tibia/femur 0.68–0.75 (0.71); tibia length/width 3.30–3.60 (3.35).

**Venter** — (640–780 (740) long; 446–521 (447) wide) colorless. Gnathosomal bay (127.5–165 (165) long; 85–96.25 (96.25) wide). Cxgl-4 subapical. **Medial suture** (7.5–22.5 (7.5) long). **Genital plates** (156.25–175 (167.5) long; 137.5–145 wide). Additional measurements: Cx-1 (238–308 (291) long (total); 98–138 (138) long (medial)); Cx-3 (307–356 (307) wide); anterior venter (157.5–170 (157.5) long). Ventral proportions: gnathosomal bay length/width 1.50–1.94 (1.71); anterior venter/genital field length 0.94–1.05 (0.94); anterior venter length/genital field width 1.14–1.24; anterior venter/medial suture 7.50–21.00 (7.50).

**Male (Figure 239)** (n = 5) (allotypic measurements in parentheses when available) with characters of the genus with following specifications.

**Dorsum** — (400–465 (460) long; 260–305 (300) wide) ovoid with faint bluish-purple coloration separated into anterior and posterior portions, and with faint orange medially. Anterio-medial platelets (82.5–100 (92.5) long; 30–40 (35) wide). Anterio-lateral platelets (115–135 (127.5) long; 35–47.5 (42.5) wide) free from dorsal plate. Dgl-4 much closer to the edge of the dorsum than to the muscle scars (distance between Dgl-4 210–250 (250)). Dorsal plate proportions: dorsum length/width 1.52–1.57 (1.53); dorsal width/distance between Dgl-4 1.20–1.27 (1.20); anterio-medial platelet length/width 2.31–2.92 (2.64); anterio-lateral platelet length/width 2.84–3.29 (3.00); anterio-lateral/anterio-medial length 1.29–1.41 (1.38).

**Gnathosoma — Subcapitulum** (210–245 (240) long (ventral); 162.5–180 (171) long (dorsal); 78.75–87.5 (85) tall) colorless. Rostrum (87.5–95 (92.5) long; 30–42.5 (32.5) wide). Chelicerae (210–234 (229) long) with curved fangs (38–42.5 (40) long). Subcapitular proportions: ventral length/height 2.67–2.82 (2.82); rostrum length/width 2.24–2.92 (2.85). **Pedipalps** with tuberculate ventral extensions on femora and genua. Palpomeres: trochanter (32.5–37.5 (37.5) long); femur (82.5–91.25 (87.5) long); genu (50–55 (52.5) long); tibia (62.5–72.5 (70) long; 20–22.5 (22.5) wide); tarsus (15–16.25 (15) long). Palpomere proportions: femur/genu 1.62–1.75 (1.67); tibia/femur 0.76–0.83 (0.80); tibia length/width 3.11–3.22 (3.11).

**Venter** — (490–571 (571) long; 310–390 (342) wide) colorless. Gnathosomal bay (92.5–115 (115) long; 55–65 (65) wide). Cxgl-4 subapical. **Medial suture** (95–105 (95) long). **Genital plates** (90–107.5 (107.5) long; 75–80 (80) wide). Additional measurements: Cx-1 (200–235 (235) long (total); 96–121 (98) long (medial)); Cx-3 (235–293 (260) wide); anterior venter (215–237.5 (232.5) long). Ventral proportions: gnathosomal bay length/width 1.54–1.82 (1.77); anterior venter/genital field length 2.16–2.39 (2.16); anterior venter length/genital field width 2.84–3.07 (2.91); anterior venter/medial suture 2.05–2.45 (2.45).

**Immatures** unknown.

######## Etymology.

Specific epithet (*shubini*) named in honor of author and palaeontologist Neil Shubin for his efforts to popularize stories of human evolution with his book (2009) and TV series (2014), *Your Inner Fish*. As with many of the species that Shubin studies (e.g., *Tiktaalik roseae* Daeschler, Shubin & Jenkins, 2006), *Torrenticola shubini* may represent a key evolutionary transition in the Rusetria Complex, between species that do and do not have their lateral platelets fused to the dorsal shield.

######## Distribution.

**Figure 237.**
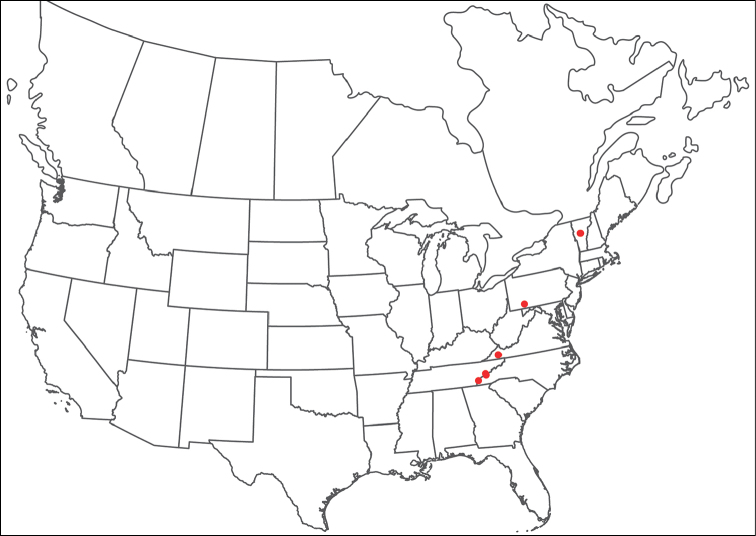
*Torrenticola shubini* sp. n. distribution.

Appalachians (Figure 237).

**Figure 238.**
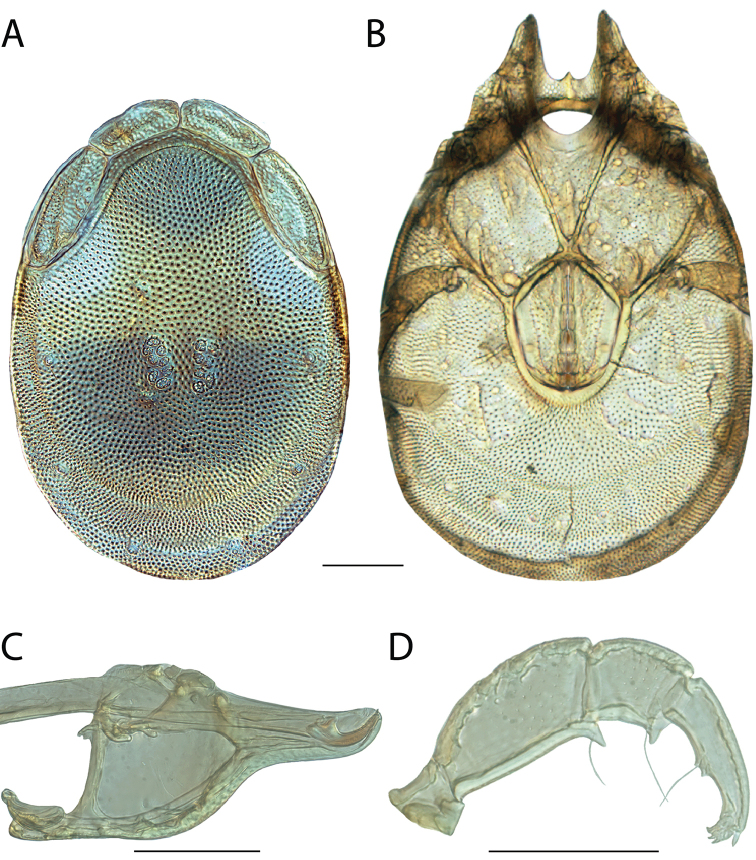
*Torrenticola shubini* sp. n. female: **A** dorsal plates **B** venter (legs removed) **C** subcapitulum **D** pedipalp (setae not accurately depicted). Scale = 100 µm.

**Figure 239.**
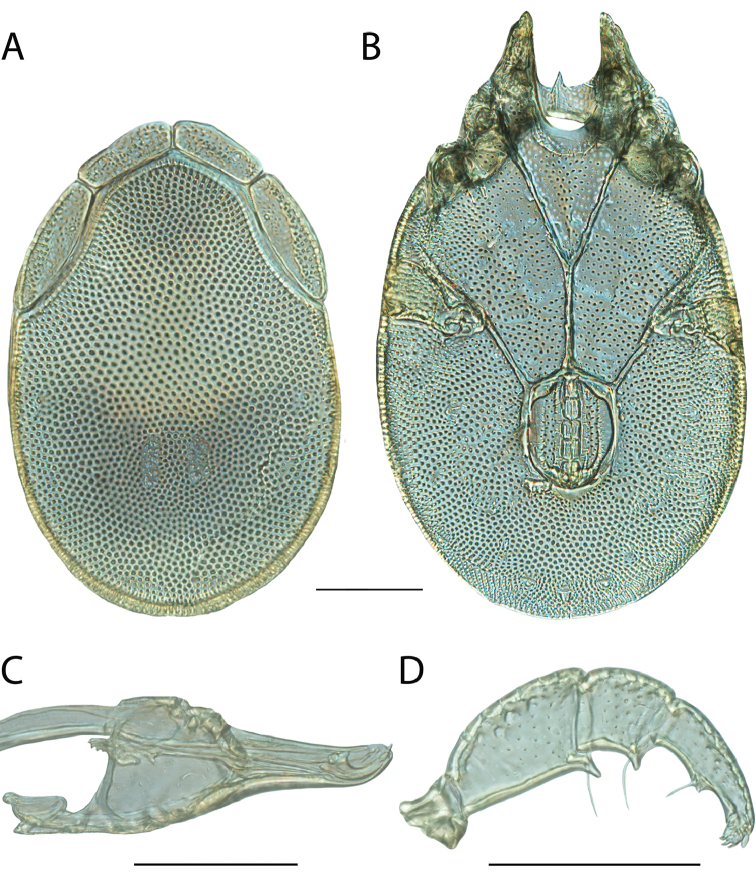
*Torrenticola shubini* sp. n. male: **A** dorsal plates **B** venter (legs removed) **C** subcapitulum **D** pedipalp (setae not accurately depicted). Scale = 100 µm.

######## Remarks.

*Torrenticola shubini* groups with other members of the Rusetria Complex with high support and specimens of this species are less than 2% different in COI sequence from each other. In all analyses, *T. shubini* groups with two other species with high support: *T. dunni* and *T. pollani*. *Torrenticola shubini* specimens are 5–12% different in COI sequence from these other species. The range of *T. shubini* overlaps with *T. dunni* in the southern Appalachians, but the ranges of these species do not overlap with *T. pollani*.

Based upon overall similarity, dorso-lateral platelet fusion, and distribution, we were able to place this species within the Eastern 4-Plate Identification Group

This species hypothesis is supported by low COI divergence within the species (0–2%) and high divergence between species (3–15%), and by the morphological characters outlined in the diagnosis.

####### Torrenticola sierrensis

Taxon classificationAnimaliaTrombidiformesTorrenticolidae

(Marshall, 1943)

Atractides sierrensis Marshall, 1943: 307.
Atractides mercedensis Marshall, 1943: 310.
Torrenticola sierrensis Mitchell, 1954: 40.

######## Material examined.

LECTOTYPE (1 ♀): USA, California, Santa Cruz County, Waddell Creek, 25-26 Aug 1932, by PR Needham, RM320007

PARALECTOTYPE (1 ♂): California, Santa Cruz County, Waddell Creek, 1 Dec 1932, by PR Needham, RM320008.

TOPOTYPES (1 ♀; 1 ♂): from USA, California, Santa Cruz County, Waddell Creek, 30-31 Aug 1933, by PR Needham, RM330016.

OTHER MATERIAL (54 ♀; 34 ♂): **British Columbia, Canada**: 3 ♀ and 1 ♂ from Ryan Rest Area off Highway 3, east of Yahk Moyie River, 15 Aug 2012, by IM Smith, IMS120071 • 1 ♀ from Vancouver Island, Lake Cowichan, Cowichan River, above Skutz Falls, 9 Jul 1979, by IM Smith, IMS790035 • **California, USA**: 1 ♀ from Del Norte County, Six Rivers National Forest, Middle Fork Smith River (41°51'20"N, 123°53'10"W), 15 Aug 2013, by JR Fisher, JRF 13-0815-002 • 1 ♀ from El Dorado County, Upper Truckee River (38°50'56"N, 120°1'39"W), 29 Aug 2013, by JR Fisher, JRF 13-0829-003 • 1 ♀ and 1 ♂ from El Dorado County, Upper Truckee River (38°50'56"N, 120°1'39"W), 29 Aug 2013, by JR Fisher, JRF 13-0829-004 • 2 ♀ and 2 ♂ from Mariposa County, El Portal, Indian Flat campground, Merced River, 9-10 Jun 1976, by IM Smith, IMS760087 • 2 ♀ from Mariposa County, Yosemite Valley, East Fork of Merced River, 22 Aug 1933, by PR Needham, RM330012 • 1 ♀ from Mendocino County, Jackson Demonstration State Park, North Fork of Big River (39°20'46"N, 123°30'35"W), 22 Aug 2013, by JR Fisher, JRF 13-0822-002 • 1 ♀ from Mendocino County, Paul M. Dimmick Recreation Area, North Fork of Navarro River, beside Route 128, 4 Aug 1987, by IM Smith, IMS870127A • 1 ♂ from Mendocino County, Rancheria Creek, beside Route 128, 7.3 kilometers south of Boonville, 4 Aug 1987, by IM Smith, IMS870126A • 1 ♀ and 1 ♂ from Monterey County, Big Sur River, beside Route 1 near Pfeiffer-Big Sur State Park, 28-29 Jul 1987, by IM Smith, IMS870116A • 3 ♀ from Monterey County, Big Sur, Pfeiffer-Big Sure State Park, Big Sur Creek, 7 Jun 1976, by IM Smith, IMS760074 • 1 ♀ from Monterey County, Los Padres National Forest, Salmon Creek (35°48'57"N, 121°21'29"W), 6 Sep 2013, by JR Fisher, JRF 13-0906-003 • 1 ♀ from Monterey County, Pfeiffer State Park, Big Sur River (36°14'42"N, 121°46'43"W), 4 Sep 2013, by JR Fisher, JRF 13-0904-004 • 1 ♀ and 1 ♂ from Monterey County, Salmon Creek, beside Route 1, south of Gorda, 28 Jul 1987, by IM Smith, IMS870114A • 1 ♀ from Nevada County, beside Route 89, north of Hobart Mills, 13 Jun 1976, by IM Smith, IMS760109 • 1 ♀ from Nevada County, Tahoe National Forest, Sagehen Creek (39°26'2"N, 120°12'17"W), 26 Aug 2013, by JR Fisher, JRF 13-0826-006 • 2 ♀ from Trinity County, Shasta-Trinity National Forest, Wilson Creek (40°25'17"N, 123°3'5"W), 20 Aug 2013, by JR Fisher, JRF 13-0820-003 • 1 ♂ from Shasta County, Battle Creek, beside Route 44, 5.6 kilometers west of Viola, 10 Aug 1987, by IM Smith, IMS870139A • 1 ♀ from Trinity County, South Fork of Trinity River, beside Route 36 at Forest Glen campground, 6 Aug 1987, by IM Smith, IMS870131 • 1 ♀ and 1 ♂ from Tulare County, Brush Creek, Brush Creek Flat, beside SM99 between Roads End Station & Johnsondale, 31 Jul 1987, by IM Smith, IMS870122 • 1 ♀ and 3 ♂ from Tulare County, Kern River, Brush Creek Flat, beside SM99 between Roads End Station & Johnsondale, 31 Jul 1987, by IM Smith, IMS870121 • 1 ♂ from Tulare County, Stony Creek, Stony Creek Picnic Area, east of Sequoia National Park, 1 Aug 1987, by IM Smith, IMS870124A • **Idaho, USA**: 1 ♀ and 1 ♂ from Custer County, Challis National Forest, Stanley Creek (44°15'12"N, 115°0'19"W), 30 Jul 2012, by JR Fisher, WA Nelson, & JC O’Neill, ROW 12-0730-005 • 1 ♀ and 1 ♂ from Custer County, Salmon River at picnic area, beside Route 93, north of Morgan Creek, 2 Jul 1985, by IM Smith, IMS850064 • 1 ♀ from Fremont County, Targhee National Forest, Rock Creek (44°6'44"N, 111°15'4"W), by JR Fisher, WA Nelson, & JC O’Neill, ROW 12-0725-001• **Montana, USA**: 1 ♀ from Ravalli County, Bitterroot National Forest, West Fork Bitterroot River (45°54'38"N, 114°9'43"W), 6 Aug 2012, by JR Fisher, WA Nelson, & JC O’Neill, ROW 12-0806-003 • 1 ♀ from Ravalli County, Medicine Springs, Spring Gulch campground, East Fork of Bitterroot River, beside Route 93, 1 Jul 1985, by IM Smith, IMS850060 • **Nevada, USA**: 1 ♀ and 1 ♂ from Humboldt County, Paradise Valley, Cabin Creek beside road, 10.7 kilometers north of Hinkey Summit, 11 Aug 1987, by IM Smith, IMS870141A • 1 ♀ and 2 ♂ from Humboldt County, Paradise Valley, Dutch John Creek beside road, 8.3 kilometers north of Hinkey Summit, 11 Aug 1987, by IM Smith, IMS870142A • **Oregon, USA**: 1 ♀ and 2 ♂ from Coos County, Siskiyou National Forest, Road 33 between Powers & Agness, 2 Jul 1983, by IM Smith, IMS830015 • 2 ♀ from Curry County, Port Orford, Butler Bar campground, Elk River, 25 Jun 1976, by IM Smith, IMS760162 • 1 ♀ and 1 ♂ from Curry County, Port Orford, Humbug Mountain State Park Picnic Area, beside Route 1, Brush Creek, 1 Jul 1983, by IM Smith, IMS830012 • 1 ♀ and 2 ♂ from Curry County, Port Orford, Humbug Mountain State Park Picnic Area, Brush Creek, beside Route 1, 3 Jul 1983, by IM Smith, IMS830020B • 1 ♀ from Curry County, Quosatana Creek (42°29'21"N, 124°14'2"W), 14 Aug 2013, by JR Fisher, JRF 13-0814-003 • 1 ♀ from Curry County, Rogue River National Forest, Elk River (42°42'46"N, 124°18'41"W), 13 Aug 2013, by JR Fisher, JRF 13-0813-003 • 3 ♀ from Lane County, Gate Creek (44°8'48"N, 122°34'20"W), 11 Aug 2013, by JC O’Neill, & WA Nelson, JNOW 13-0811-001 • 1 ♀ and 1 ♂ from Curry County, Sixes, Sixes River, beside road at mouth of Edson Creek, 4 Jul 1983, by IM Smith, IMS830021B • 1 ♀ and 1 ♂ from Lincoln County, Blackberry campground near Tidewater, Alsea River, 28 Jun 1983, by IM Smith, IMS830009 • 2 ♀ and 2 ♂ from Lincoln County, Blackberry campground near Tidewater, Alsea River, 29 Jun 1983, by IM Smith, IMS830010 • 2 ♀ and 2 ♂ from Lincoln County, Siuslaw National Forest, Five Rivers Creek (44°19'53"N, 123°50'59"W), 8 Aug 2013, by JC O’Neill, & WA Nelson, JNOW 13-0808-001 • **Washington, USA**: 2 ♀ and 2 ♂ from Cowlitz County (46°22'24"N, 122°34'45"W), 16 Jul 2013, by JC O’Neill, & WA Nelson, JNOW 13-0716-001 • 1 ♀ and 1 ♂ from Grays Harbor County, Stewarts Creek (47°15'49"N, 123°55'12"W), 25 Jul 2013, by JC O’Neill, & WA Nelson, JNOW 13-0725-001 • **Wyoming, USA**: 1 ♀ and 2 ♂ from Sublette County, Boulder Creek, beside Route 191, just north of Boulder, 15 Aug 1987, by IM Smith, IMS870151A & IMS870151B.

######## Type deposition.

Types (1 ♀; 1 ♂) deposited in the CNC.

######## Diagnosis.

*Torrenticola sierrensis* are similar to other members of the Tricolor Complex (*T. bittikoferae*, *T. hoosieri*, *T. larvata*, *T. pearsoni*, *T. olliei*, *T. tricolor*, *T. trimaculata*, *T. unimaculata*, *T. cardia*, *T. kringi*, *T. dimorpha*, and *T. mohawk*) in having a short, conical rostrum. *T. sierrensis* can be differentiated from most Tricolor Complex by being distributed in the west and having a wider genital field (♀ = 180–213 in *T. sierrensis*, 145–180 in others; ♂ = 135–175 in *T. sierrensis*, 92–120 in others), except for *T. olliei* (♀ = 190–203, ♂ = 130–138). *T. sierrensis* can be differentiated from *T. olliei* by having a more elongate rostrum (length/width = 1.95–2.14 in *T. sierrensis*, 1.56–1.81 in *T. olliei*).

######## Re-description.

**Female (Figure 241)** (n = 9) (holotype measurements in parentheses when available) with characters of the genus with following specifications.

**Dorsum** — (700–880 (865) long; 550–740 (710) wide) circular with orange or reddish purple coloration separated into anterior and posterior portions with faint orange medially (occasionally colorless). Anterio-medial platelets (162.5–197.5 (187.5) long; 65–82.5 (81.25) wide). Anterio-lateral platelets (192.5–250 (225) long; 82.5–100 (92.5) wide) free from dorsal plate. Dgl-4 much closer to the edge of the dorsum than to the muscle scars (distance between Dgl-4 435–520 (500)). Dorsal plate proportions: dorsum length/width 1.19–1.27 (1.22); dorsal width/distance between Dgl-4 1.22–1.43 (1.42); anterio-medial platelet length/width 2.10–2.50 (2.31); anterio-lateral platelet length/width 2.33–2.50 (2.43); anterio-lateral/anterio-medial length 1.18–1.27 (1.20).

**Gnathosoma — Subcapitulum** 320–365 (365) long (ventral); 227–265 (260) long (dorsal); 140–165 (152.5) tall) colorless. Rostrum (120–140 (140) long; 60–70 (70) wide) short and conical. Chelicerae (320–355 long) with curved fangs (61–77.5 long). Subcapitular proportions: ventral length/height 2.19–2.39 (2.39); rostrum length/width 1.96–2.08 (2.00). **Pedipalps** with tuberculate ventral extensions with dentate tips on femora and genua. Palpomeres: trochanter (45–55 (55) long); femur (115–135 (135) long); genu (80–92.5 (92.5) long); tibia (107.5–117.5 (117.5) long; 32.5–35 (35) wide); tarsus (21.25–35 32.5) long). Palpomere proportions: femur/genu 1.35–1.55 (1.46); tibia/femur 0.86–0.95 (0.87); tibia length/width 3.07–3.48 (3.36).

**Venter** — (775–1040 (1015) long; 619–820 (770) wide) colorless. Gnathosomal bay (155–205 (195) long; 95–112.5 (112.5) wide). Cxgl-4 subapical. **Medial suture** (17.5–37.5 (22.5) long). **Genital plates** (210–235 (222.5) long; 180–212.5 (212.5) wide). Additional measurements: Cx-1 (192.5–370 (370) long (total); 136–185 (172.5) long (medial)); Cx-3 (250–475 (475) wide); anterior venter (192.5–237.5 (212.5) long). Ventral proportions: gnathosomal bay length/width 1.50–1.91 (1.73); anterior venter/genital field length 0.90–1.06 (0.96); anterior venter length/genital field width 1.00–1.13 (1.00); anterior venter/medial suture 6.33–12.14 (9.44).

**Male (Figure 242)** (n = 7) (allotypic measurements in parentheses when available) with characters of the genus with following specifications.

**Dorsum** — (590–735 (715) long; 460–590 (580) wide) circular with orange or reddish purple coloration separated into anterior and posterior portions with faint orange medially (occasionally colorless). Anterio-medial platelets (137.5–172.5 (172.5) long; 60–75 (75) wide). Anterio-lateral platelets (185–222.5 (215) long; 72.5–95 (92.5) wide) free from dorsal plate. Dgl-4 much closer to the edge of the dorsum than to the muscle scars (distance between Dgl-4 355–460 (460)). Dorsal plate proportions: dorsum length/width 1.17–1.28 (1.23); dorsal width/distance between Dgl-4 1.26–1.33 (1.26); anterio-medial platelet length/width 2.24–2.48 (2.30); anterio-lateral platelet length/width 2.12–2.55 (2.32); anterio-lateral/anterio-medial length 1.22–1.38 (1.25).

**Gnathosoma — Subcapitulum** (270–330 (325) long (ventral); 200–235 (235) long (dorsal); 118.75–140 (135) tall) colorless. Rostrum (100–122.5 (122.5) long; 50–60 (60) wide) short and conical. Chelicerae (262–317.5 (315) long) with curved fangs (50–67.5 (60) long). Subcapitular proportions: ventral length/height 2.23–2.46 (2.41); rostrum length/width 1.95–2.14 (2.04). **Pedipalps** with tuberculate ventral extensions with dentate tips on femora and genua. Palpomeres: trochanter (40–50 (50) long); femur (92.5–120 (120) long); genu (65–80 (78.75) long); tibia (82.5–107.5 (102.5) long; 27.5–32.5 (32.5) wide); tarsus (25–32.5 (32.5) long). Palpomere proportions: femur/genu 1.42–1.52 (1.52); tibia/femur 0.85–0.95 (0.85); tibia length/width 3.00–3.31 (3.15).

**Venter** — (690–895 (875) long; 538–667 (665) wide) colorless. Gnathosomal bay (112.5–180 (175) long; 80–100 (92.5) wide). Cxgl-4 subapical. **Medial suture** (72.5–92.5 (90) long). **Genital plates** (172.5–220 (217.5) long; 135–175 (175) wide). Additional measurements: Cx-1 (269–360 (360) long (total); 137–182.5 (182.5) long (medial)); Cx-3 (364–455 (455) wide); anterior venter (255–295 (290) long). Ventral proportions: gnathosomal bay length/width 1.41–1.89 (1.89); anterior venter/genital field length 1.32–1.51 (1.33); anterior venter length/genital field width 1.66–1.89 (1.66); anterior venter/medial suture 3.03–3.59 (3.22).

**Immatures** unknown.

######## Etymology.

Although Marshall (1943) did not specify an etymology for the specific epithet (*sierrensis*), surely it refers to the type locality of this species within the Sierra Nevada in California.

######## Distribution.

**Figure 240.**
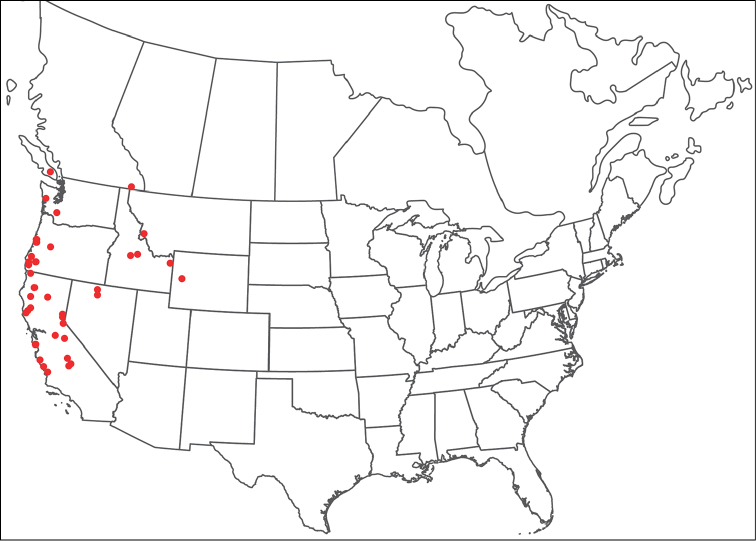
*Torrenticola sierrensis* distribution.

Western (Figure 240), but likely not found in the southwest. *T. sierrensis* was previously known only from a few localities in California; we extend the range in western North America, but not in the southwest.

**Figure 241.**
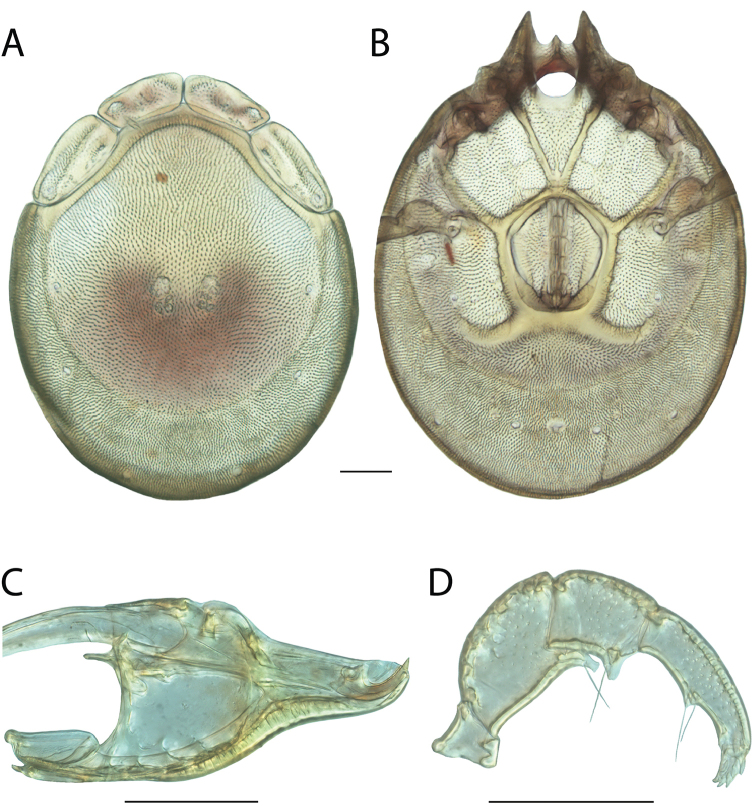
*Torrenticola sierrensis* female: **A** dorsal plates **B** venter (legs removed) **C** subcapitulum **D** pedipalp (setae not accurately depicted). Scale = 100 µm.

**Figure 242.**
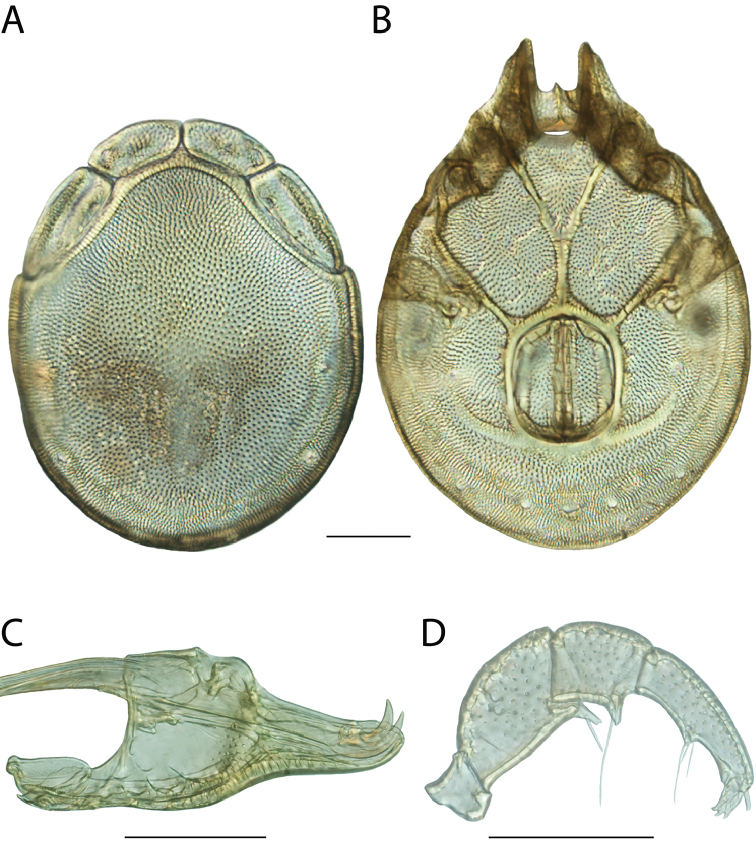
*Torrenticola sierrensis* male: **A** dorsal plates **B** venter (legs removed) **C** subcapitulum **D** pedipalp (setae not accurately depicted). Scale = 100 µm.

######## Remarks.

*Torrenticola sierrensis* group with other members of the Tricolor Complex with high support in all analyses and group with *T. olliei* to form the western portion of this complex. Specimens within this complex are 0–4.5% different in COI sequence from each other and greater than 6% different from *T. olliei*. This is higher sequence variability in COI than in most species hypotheses presented herein. However, given the topology in the COI tree (Figure 10) and morphological similarity, it seems apparent that the variability represents a continuum across a large distribution, rather than isolated species. This species hypothesis is supported by phylogenetic affinity and by the morphological characters outlined in the diagnosis.

Upon examining the types of *T. sierrensis* and *T. mercedensis* (Marshall, 1943), all characters for both species overlap with members of only one clade in our analyses. Therefore, it is apparent that these represent the same species hypothesis and must be synonymized. As First Revisers (ICZN Article 24.2), we select “*sierrensis*” as the senior synonym over “*mercedensis*” due to its broader range implication (“*mercedensis*” refers to the Merced River), even though *T. sierrensis* is distributed more widely than just the Sierra Nevada ranges.

####### Torrenticola skvarlai

Taxon classificationAnimaliaTrombidiformesTorrenticolidae

Fisher & Dowling sp. n.

http://zoobank.org/FBB57CBC-6128-4319-8891-2920FDD53171

######## Material examined.

HOLOTYPE (♀): from USA, Pennsylvania, Somerset County, Laurel Hill State Park, Laurel Hill Creek (40°1'6"N, 79°14'4"W), 8 Aug 2014, by MJ Skvarla, MS 14-0808-001.

PARATYPES (17 ♀; 24 ♂): **New Hampshire, USA**: 1 ♀ and 1 ♂ from Coos County, Randolph, Moose River, beside Route 2, 30 Jul 1981, by IM Smith, IMS810105 • 2 ♀ and 2 ♂ from Coos County, White Mountain National Forest, Peabody River, Dolly Copp Campground, south of Gorham, 27 Jul 1981, IMS810099A & IMS810099B • **New York, USA**: 1 ♀ and 1 ♂ from Essex County, Minerva, Boreas River, beside Route 28N, 13.8 kilometers northwest of Morse Memorial Parkway, 21 Jun 1990, by IM Smith, IMS900050A • 2 ♀ and 1 ♂ from Greene County, Schoharie Creek, beside Route 23A, 9.6 kilometers west of Route 296 in Hunter, 22 Jun 1990, by IM Smith, IMS900052 • **North Carolina, USA**: 1 ♀ and 1 ♂ from Macon County, Cullasaja River, Highlands beside Route 64/28, 4.1 kilometers northwest of Cliffside Lake Road, 30 Jun 1990, by IM Smith, IMS900070A • 2 ♂ from Macon County, Rainbow Springs, Nantahala River, beside Forest Route 67, south of Standing Indian Campground, 1 Jul 1990, IMS900072 • 2 ♀ and 2 ♂ from Yancey County, South Toe River, Lost Cove Picnic Area on Forest Route 472, 2.8 kilometers south of Route 80, 28 Jun 1990, by IM Smith, IMS900065A • **Pennsylvania, USA**: 1 ♂ from Bedford County, Chaneysville, Sweet Root Picnic Area beside Route 326, 18 Jul 1990, by IM Smith, IMS900105 • 1 ♀ and 3 ♂ from Fayette County, Dunbar Creek (39°57'50"N, 79°35'8.70"W), 10 Aug 2014, by MJ Skvarla, MS 14-0810-001 • 2 ♀ and 1 ♂ from Huntingdon County, Alan Seeger Natural Area, Stone Creek, beside road from McAlevys Fort to Route 322, 19 Jul 1990, by IM Smith, IMS900107 • 1 ♂ (ALLOTYPE) from Somerset County, Laurel Hill State Park, Laurel Hill Creek (40°1'6"N, 79°14'4"W), 8 Aug 2014, by MJ Skvarla, MS 14-0808-001 • 2 ♀ from Somerset County, Laurel Hill State Park, Laurel Hill Creek (40°1'6"N, 79°14'4"W), 8 Aug 2014, by MJ Skvarla, MS 14-0808-001 • **Tennessee, USA**: 1 ♀ and 1 ♂ from Monroe County, Tellico River, beside Forest Route 210, 1.8 kilometers east of bridge at Bald River Falls, 5 Jul 1990, by IM Smith, IMS900079 • 1 ♂ from Monroe County, Turkey Creek , beside Forest Route 35, 2.0 kilometers northeast of road from Route 165 to Miller Chapel Church, 5 Jul 1990, by IM Smith, IMS900078 • **Virginia, USA**: 1 ♀ and 4 ♂ from Washington County, Damascus, Laurel River, beside Route 58 near boundary of Mount Rogers National Recreation Area, 10 Jul 1990, by IM Smith, IMS900085A • **West Virginia, USA**: 1 ♂ from Pendleton County, Spruce Knob, beside Forest Route 112, 10.2 kilometers west of Route 33, 17 Jul 1990, by IM Smith, IMS900103 • 1 ♀ and 1 ♂ from Randolph County, Laurel Fork of Cheat River, Laurel Fork Campground off Forest Route 14, south of Wymer, 17 Jul 1990, by IM Smith, IMS900102.

######## Type deposition.

Holotype (♀), allotype (♂), and other paratypes (12 ♀; 18 ♂) deposited in the CNC; other paratypes (5 ♀; 5 ♂) deposited in ACUA.

######## Diagnosis.

*Torrenticola skvarlai* are similar to species with similar dorsal patterning, such as the Rusetria “4-Plate” group (*T. dunni*, *T. glomerabilis*, *T. kittatinniana*, *T. pollani*, *T. rufoalba*, and *T. shubini*), Elongata Group (*T. elongata*, *T. gorti*, and *T. reduncarostra*), Neoanomala Group (*T. interiorensis* and *T. neoanomala*), *T. bondi*, *T. racupalpa*, *T. irapalpa*, *T. erectirostra*, and *T. arktonyx*. They can be differentiated from all of these except Rusetria 4-Plates by having indistinct hind coxal margins. *T. skvarlai* can be differentiated from all Rusetria 4-Plates by having broadly tuberculate, dentate pedipalp femoral extensions (all Rusetria Complex have conical tuberculate pedipalp femoral extensions, usually without dentation) and by having a shorter anterior venter (♀ = 140–152.5 in *T. skvarlai*, 155–213 in Rusetria 4-Plates; ♂ = 177–205 in *T. skvarlai*, 215–285 in Rusetria 4-Plates).

######## Description.

**Female (Figure 244)** (n = 5) (holotype measurements in parentheses when available) with characters of the genus with following specifications.

**Dorsum** — (495–560 (495) long; 370–420 (370) wide) ovoid with reddish-purple or bluish-purple coloration separated into anterior and posterior portions, occasionally with orange medially. Anterio-medial platelets (107.5–117.5 (107.5) long; 42.5–47.5 (42.5) wide). Anterio-lateral platelets (155–167.5 (155) long; 52.5–62.5 (52.5) wide) free from dorsal plate. Dgl-4 closer to the edge of the dorsum than to the muscle scars (distance between Dgl-4 245–285 (255)). Dorsal plate proportions: dorsum length/width 1.33–1.39 (1.34); dorsal width/distance between Dgl-4 1.45–1.58 (1.45); anterio-medial platelet length/width 2.37–2.53 (2.53); anterio-lateral platelet length/width 2.68–2.95 (2.95); anterio-lateral/anterio-medial length 1.36–1.44 (1.44).

**Gnathosoma — Subcapitulum** (290–315 (290) long (ventral); 218–240 (219) long (dorsal); 120–135 (120) tall) colorless. Rostrum (115–130 (115) long; 40–47.5 (40) wide). Chelicerae (286.75–321 (299) long) with curved fangs (49–65 (54) long). Subcapitular proportions: ventral length/height 2.33–2.48 (2.42); rostrum length/width 2.74–2.94 (2.88). **Pedipalps** with broadly tuberculate, dentate ventral extensions on femora and tuberculate ventral extensions on genua. Palpomeres: trochanter (37.5–45 (40) long); femur (105–119 (105) long); genu (62.5–67.5 (62.5) long); tibia (82.5–90 (82.5) long; 22.5–25 (22.5) wide); tarsus (17.5–20 (17.5) long). Palpomere proportions: femur/genu 1.67–1.76 (1.68); tibia/femur 0.76–0.79 (0.79); tibia length/width 3.50–3.78 (3.67).

**Venter** — (600–690 (600) long; 435–480 (435) wide) with faint reddish-purple or bluish-purple coloration. Gnathosomal bay (125–165 (135) long; 85–105 (85) wide). Cxgl-4 subapical. **Medial suture** (12.5–15 (12.5) long). **Genital plates** (165–172.5 (166.25) long; 150–152.5 (150) wide). Additional measurements: Cx-1 (243–285 (257) long (total); 109–120 (109) long (medial)); Cx-3 (295–345 (300) wide); anterior venter (140–152.5 (145) long). Ventral proportions: gnathosomal bay length/width 1.47–1.71 (1.59); anterior venter/genital field length 0.84–0.92 (0.87); anterior venter length/genital field width 0.93–1.02 (0.97); anterior venter/medial suture 10.17–12.00 (11.60).

**Male (Figure 245)** (n = 5) (allotypic measurements in parentheses when available) with characters of the genus with following specifications.

**Dorsum** — (425–510 (450) long; 310–360 (325) wide) ovoid with reddish-purple or bluish-purple coloration separated into anterior and posterior portions, occasionally with orange medially. Anterio-medial platelets (95–102.5 (100) long; 38.75–47.5 (40) wide). Anterio-lateral platelets (142.5–177.5 (147.5) long; 45–57.5 (47.5) wide) free from dorsal plate. Dgl-4 closer to the edge of the dorsum than to the muscle scars (distance between Dgl-4 220–240 (240)). Dorsal plate proportions: dorsum length/width 1.32–1.42 (1.38); dorsal width/distance between Dgl-4 1.35–1.50 (1.35); anterio-medial platelet length/width 2.16–2.50 (2.50); anterio-lateral platelet length/width 3.09–3.28 (3.11); anterio-lateral/anterio-medial length 1.48–1.73 (1.48).

**Gnathosoma — Subcapitulum** (245–290 (255) long (ventral); 177.5–220 (193) long (dorsal); 92.5–105 (97.5) tall) colorless. Rostrum (97.5–125 (102.5) long; 32.5–45 (35) wide). Chelicerae (235–280 (245) long) with curved fangs (47.5–55 (54) long). Subcapitular proportions: ventral length/height 2.62–2.76 (2.62); rostrum length/width 2.78–3.00 (2.93). **Pedipalps** with broadly tuberculate, dentate ventral extensions on femora and tuberculate ventral extensions on genua. Palpomeres: trochanter (30–40 (36.25) long); femur (87.5–105 (87.5) long); genu (50–60 (52.5) long); tibia (75–82.5 (78.75) long; 20–22.5 (21.25) wide); tarsus (15–18.75 (17.5) long). Palpomere proportions: femur/genu 1.67–1.80 (1.67); tibia/femur 0.79–0.90 (0.90); tibia length/width 3.65–3.88 (3.71).

**Venter** — (530–620 (550) long; 358–415 (359) wide) with faint reddish-purple or bluish-purple coloration. Gnathosomal bay (130–145 (130) long; 62.5–75 (73.75) wide). Cxgl-4 subapical. **Medial suture** (55–75 (75) long). **Genital plates** (120–135 (120) long; 100–110 (100) wide). Additional measurements: Cx-1 (225–280 (242) long (total); 100–135 (102) long (medial)); Cx-3 (270–320 (272) wide); anterior venter (177.5–205 (192.5) long). Ventral proportions: gnathosomal bay length/width 1.76–2.16 (1.76); anterior venter/genital field length 1.48–1.63 (1.60); anterior venter length/genital field width 1.78–1.95 (1.93); anterior venter/medial suture 2.57–3.23 (2.57).

**Immatures** unknown.

######## Etymology.

Specific epithet (*skvarlai*) named in honor of Michael Skvarla, who collected specimens of the species and is a dear friend and colleague of JRF.

######## Distribution.

**Figure 243.**
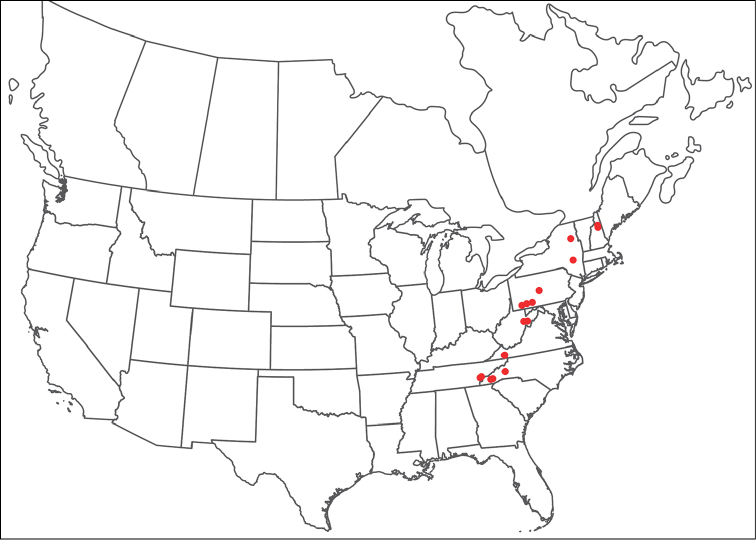
*Torrenticola skvarlai* sp. n. distribution.

Appalachians (Figure 243).

**Figure 244.**
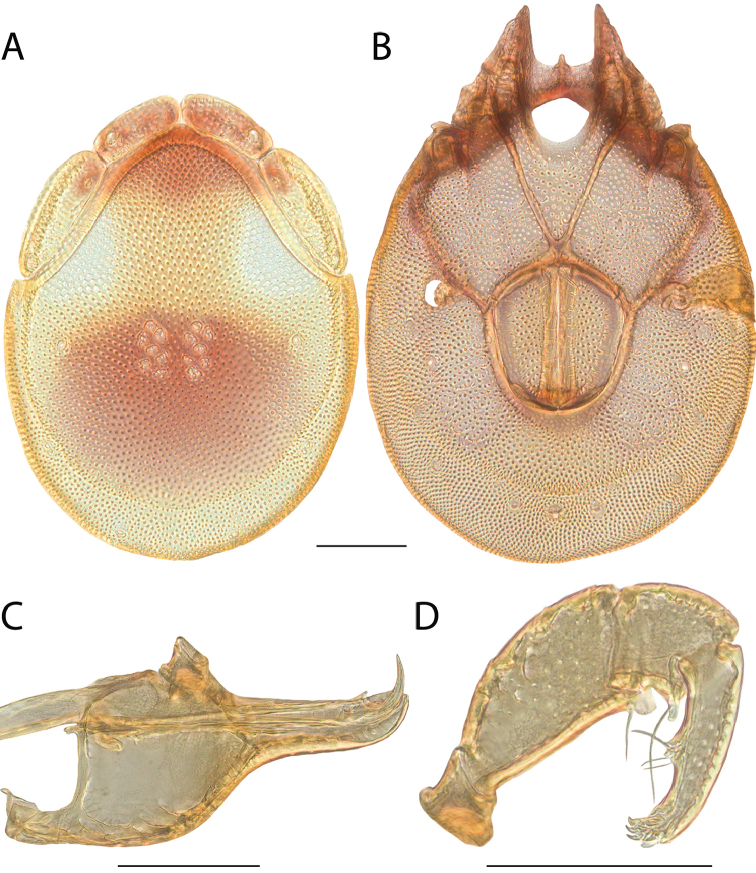
*Torrenticola skvarlai* sp. n. female: **A** dorsal plates **B** venter (legs removed) **C** subcapitulum **D** pedipalp (setae not accurately depicted). Scale = 100 µm.

**Figure 245.**
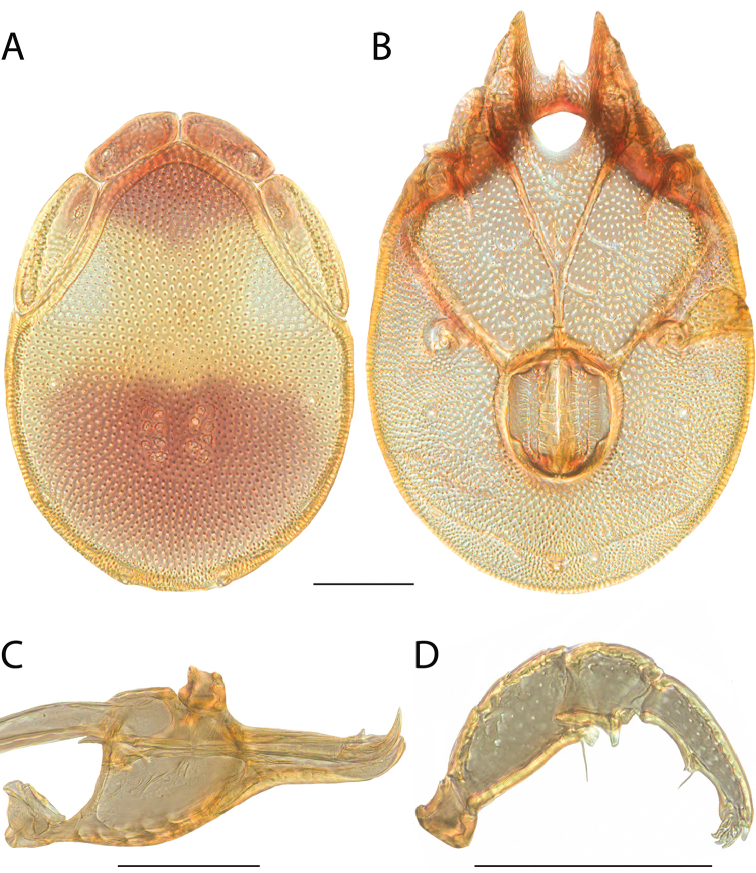
*Torrenticola skvarlai* sp. n. male: **A** dorsal plates **B** venter (legs removed) **C** subcapitulum **D** pedipalp (setae not accurately depicted). Scale = 100 µm.

######## Remarks.

*Torrenticola skvarlai* groups with other members of the Raptor Complex with high support and specimens are less than 2% different in COI sequence from each other and greater than 9% from sister species. Placement of this species varied with analysis and was never well-supported. Furthermore, this species is not readily identifiable without keying, which does not warrant placement within an identification group.

This species hypothesis is supported by low COI divergence within the species (0–2%) and high divergence between species (3–15%), and by the morphological characters outlined in the diagnosis.

####### Torrenticola solisorta

Taxon classificationAnimaliaTrombidiformesTorrenticolidae

Fisher & Dowling sp. n.

http://zoobank.org/FDDDB599-3669-430E-B96E-09567B324EA4

######## Material examined.

HOLOTYPE (♀): from USA, Arkansas, Polk County, East Saline Creek, beside Forest Road 38, north of Shady Lake Recreation Area, 30 Jul 2011, by IM Smith, IMS 110041, DNA 1300.

PARATYPES (4 ♀; 6 ♂): **Arkansas, USA**: 2 ♂ from Montgomery County, Gaston, South Fork Ouachita River, 29 Jul 2011, by IM Smith, IMS110040 • 1 ♂ (ALLOTYPE) from Montgomery County, Ouachita National Forest, South Fork Ouachita River, 29 Jul 2011, by AJ Radwell, & B Crump, AJR110302 • 4 ♀ and 3 ♂ from Polk County, East Saline Creek, beside Forest Road 38, north of Shady Lake Recreation Area, 30 Jul 2011, by IM Smith, IMS 110041.

######## Type deposition.

Holotype (♀), allotype (♂), and most paratypes (2 ♀; 4 ♂) deposited in the CNC; other paratypes (2 ♀; 2 ♂) deposited in ACUA.

######## Diagnosis.

*Torrenticola solisorta* are similar to other members of the Nigroalba Group (*T. flangipalpa*, *T. nigroalba*, and *T. dentirostra*) in being small, slightly elongate, having purple dorsal coloration restricted posteriorly, and having distinct yet poorly-defined hind coxal margins. *T. solisorta* can be differentiated from *T. flangipalpa* by having tuberculate pedipalp femoral extension (flange-like and anteriorly-directed in *T. flangipalpa*); a shorter anterior venter (192–225 in *T. solisorta*, 235–265 in *T. flangipalpa*); and more elongate pedipalp tibia (length/ width ♀ = 5.67–5.82 in *T. solisorta*, 4.79–5.00 in *T. flangipalpa*; ♂ = 5.08–5.17 in *T. solisorta*, 4.40–4.86 in *T. flangipalpa*). *T. solisorta* can be differentiated from *T. dentirostra* by having a smooth rostrum (*T. dentirostra* has a dentate bump midway on the dorsal edge of the rostrum) and more elongate pedipalpal tibiae (length/width ♀ = 5.6–5.9 in *T. solisorta*, 4.5–5.0 in *T. dentirostra*; ♂ = 5.0–5.2 in *T. solisorta*, 4.5–4.7 in *T. dentirostra*). *T. solisorta* can be differentiated from *T. nigroalba* by having orangish coloration immediately anterior to the purple dorsal coloration. Additionally, female *T. solisorta* can be differentiated from female *T. nigroalba* by having a slightly shorter dorsum (475–500 in *T. solisorta*, 500–530 in *T. nigroalba*); a thicker gnathosomal bay (length/width = 1.3–1.5 in *T. solisorta*, 1.25–1.55 in *T. nigroalba*); and a slightly thinner subcapitulum (3.14–3.30 in *T. solisorta*, 3.00–3.14 in *T. nigroalba*). Male *T. solisorta* also can be differentiated from *T. nigroalba* by anterior venter/medial suture (2.87–3.26 in *T. solisorta*, 2.54–2.77 in *T. nigroalba*) and having a wider dorsum (305–320 in *T. solisorta*, 290–300 in *T. nigroalba*). Other *Torrenticola* with purple dorsal coloration restricted posteriorly can be confused with *T. solisorta*, such as *T. tahoei* and *T. oregonensis*. Both of these species are larger (dorsum length ♀ = 475–500 in *T. solisorta*, 600–840 in others; ♂ = 425–460 in *T. solisorta*, 560–820 in others) and distributed in the west (*T. solisorta* is known only from the Ouachita Mountains in Arkansas).

######## Description.

**Female (Figure 247)** (n = 5) (holotype measurements in parentheses when available) with characters of the genus with following specifications.

**Dorsum** — (475–500 (500) long; 325–360 (350) wide) ovoid with bluish-purple to purple coloration restricted posteriorly with orange immediately anterior and fading anteriorly. Anterio-medial platelets (102.5–112.5 (112.5) long; 42.5–46.25 (45) wide). Anterio-lateral platelets (138.75–150 (147.5) long; 47.5–50 (47.5) wide) free from dorsal plate. Dgl-4 closer to the edge of the dorsum than to the muscle scars (distance between Dgl-4 220–240 (240)). Dorsal plate proportions: dorsum length/width 1.38–1.46 (1.43); dorsal width/distance between Dgl-4 1.45–1.50 (1.46); anterio-medial platelet length/width 2.22–2.53 (2.50); anterio-lateral platelet length/width 2.78–3.16 (3.11); anterio-lateral/anterio-medial length 1.31–1.43 (1.31).

**Gnathosoma — Subcapitulum** (280–300 (300) long (ventral); 199–217 (217) long (dorsal); 85–92.5 (92.5) tall) elongate and colorless. Rostrum (105–110 (110) long; 35–37.5 (36.25) wide). Chelicerae (256–281 (278) long) with curved fangs (34–44 (43) long). Subcapitular proportions: ventral length/height 3.14–3.29 (3.24); rostrum length/width 2.87–3.03 (3.03). **Pedipalps** elongate (especially tibiae) with tuberculate ventral extensions on femora and genua ending broadly and dentate. Palpomeres: trochanter (27.5–30 (30) long); femur (86.25–93.75 (93.75) long); genu (52.5–55 (55) long); tibia (80–85 (85) long; 13.75–15 (15) wide); tarsus (12.5–15 (12.5) long). Palpomere proportions: femur/genu 1.64–1.70 (1.70); tibia/femur 0.91–0.99 (0.91); tibia length/width 5.67–5.82 (5.67).

**Venter** — (580–640 (630) long; 356–439 (380) wide) with bluish-purple or purple coloration. Gnathosomal bay (97.5–110 (110) long; 70–77.5 (77.5) wide). Cxgl-4 far from apex. **Medial suture** (47.5–50 (47.5) long). **Genital plates** (132.5–140 (140) long; 117.5–122.5 (122.5) wide). Additional measurements: Cx-1 (228–249 (249) long (total); 88–121 (121) long (medial)); Cx-3 (236.5–259.75 (252) wide); anterior venter (192.5–207.5 (205) long). Ventral proportions: gnathosomal bay length/width 1.30–1.50 (1.42); anterior venter/genital field length 1.45–1.54 (1.46); anterior venter length/genital field width 1.60–1.77 (1.67); anterior venter/medial suture 3.85–4.37 (4.32).

**Male (Figure 248)** (n = 6) (allotypic measurements in parentheses when available) with characters of the genus with following specifications.

**Dorsum** — (425–460 (435) long; 305–320 (305) wide) ovoid with bluish-purple to purple coloration restricted posteriorly with orange immediately anterior and fading anteriorly. Anterio-medial platelets (97.5–105 (100) long; 40–45 (42.5) wide). Anterio-lateral platelets (130–145 (145) long; 45–50 (45) wide) free from dorsal plate. Dgl-4 closer to the edge of the dorsum than to the muscle scars (distance between Dgl-4 205–220 (215)). Dorsal plate proportions: dorsum length/width 1.37–1.48 (1.43); dorsal width/distance between Dgl-4 1.42–1.51 (1.42); anterio-medial platelet length/width 2.33–2.44 (2.35); anterio-lateral platelet length/width 2.85–3.22 (3.22); anterio-lateral/anterio-medial length 1.32–1.45 (1.45).

**Gnathosoma — Subcapitulum** (252.5–265 (255) long (ventral); 181–196 (190) long (dorsal); 80–87.5 (80) tall) elongate and colorless. Rostrum (92.5–100 (100) long; 32.5–35 (35) wide). Chelicerae (240–246 (240) long) with curved fangs (33–45 (45) long). Subcapitular proportions: ventral length/height 2.97–3.19 (3.19); rostrum length/width 2.81–2.92 (2.86). **Pedipalps** elongate (especially tibiae) with tuberculate ventral extensions on femora and genua ending broadly and dentate. Palpomeres: trochanter (27.5–30 (27.5) long); femur (80–82.5 (80) long); genu (48.75–51.25 (48.75) long); tibia (77.5–82.5 (77.5) long; 15–16.25 (15) wide); tarsus (12.5–15 (12.5) long). Palpomere proportions: femur/genu 1.59–1.65 (1.64); tibia/femur 0.94–1.00 (0.97); tibia length/width 5.08–5.17 (5.17).

**Venter** — (520–570 (545) long; 335–382 (335) wide) with bluish-purple or purple coloration. Gnathosomal bay (85–107.5 (102.5) long; 60–72.5 (67.5) wide). Cxgl-4 far from apex. **Medial suture** (65–77.5 (75) long). **Genital plates** (105–115 (105) long; 90–95 (92.5) wide). Additional measurements: Cx-1 (208–231 (230) long (total); 91–130 (130) long (medial)); Cx-3 (240–281 (240) wide); anterior venter (210–225 (215) long). Ventral proportions: gnathosomal bay length/width 1.17–1.52 (1.52); anterior venter/genital field length 1.87–2.10 (2.05); anterior venter length/genital field width 2.27–2.50 (2.32); anterior venter/medial suture 2.87–3.26 (2.87).

**Immatures** unknown.

######## Etymology.

Specific epithet (*solisorta*) refers to the dorsal coloration of this species, which is the easiest way to differentiate it from its sister species (*T. nigroalba*). If one imagines the posterior purple color as the landscape and horizon, then the orangish coloration resembles the sun beginning to rise (*solis ortus*, L. sunrise).

######## Distribution.

**Figure 246.**
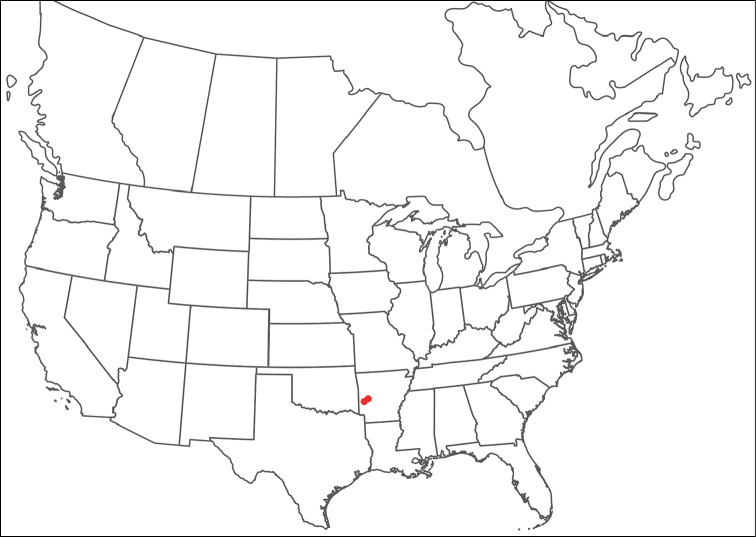
*Torrenticola solisorta* sp. n. distribution.

Ouachita Mountains (Figure 246).

**Figure 247.**
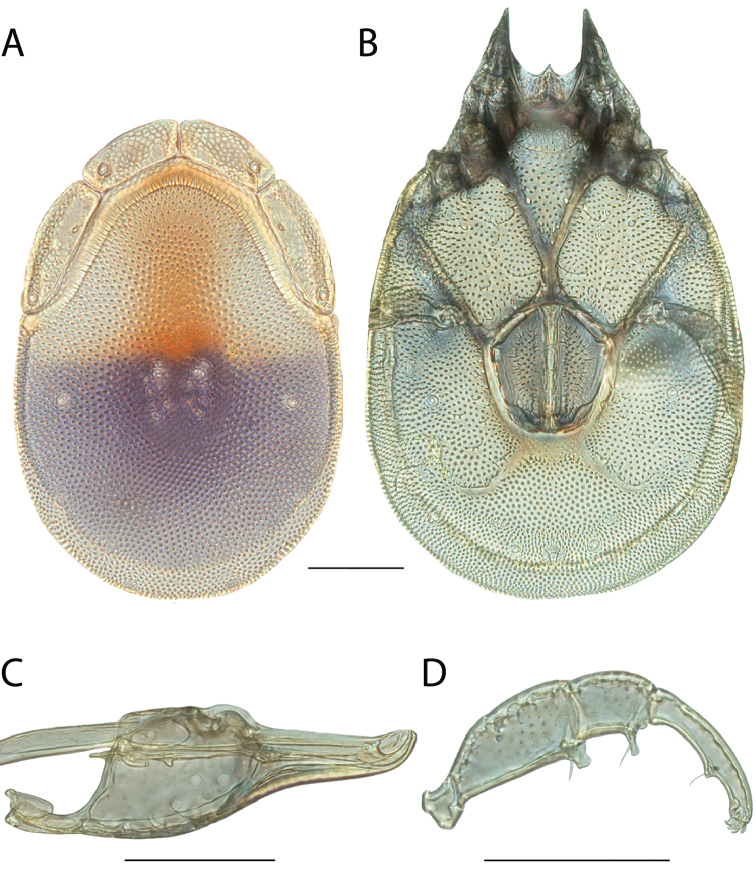
*Torrenticola solisorta* sp. n. female: **A** dorsal plates **B** venter (legs removed) **C** subcapitulum **D** pedipalp (setae not accurately depicted). Scale = 100 µm.

**Figure 248.**
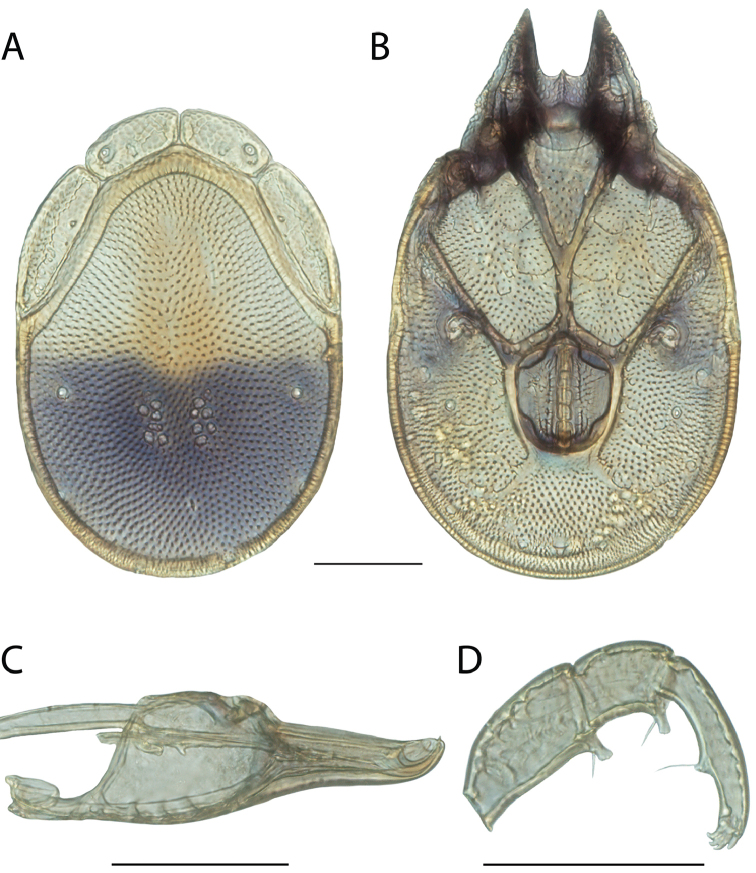
*Torrenticola solisorta* sp. n. male: **A** dorsal plates, note the faint orange coloration, which is a rare character state (most specimens are much brighter), not a male character **B** venter (legs removed) **C** subcapitulum **D** pedipalp (setae not accurately depicted). Scale = 100 µm.

######## Remarks.

*Torrenticola solisorta* groups with other members of the Raptor Complex with high support in all analyses and specimens are less than 1% different in COI sequence from each other. In all analyses, *T. solisorta* groups with two other morphologically similar species: *T. flangipalpa* and *T. nigroalba*. *Torrenticola solisorta* is greater than 4% different in COI from its sister species (*T. nigroalba*). That clade of three species corresponds to an identification group, the Nigroalba Group, the members of which are easily differentiated by their size, coloration, long medial suture in females, and overall appearance.

This species hypothesis is supported by low COI divergence within the species (0–2%) and high divergence between species (3–15%), and by the morphological characters outlined in the diagnosis.

####### Torrenticola tahoei

Taxon classificationAnimaliaTrombidiformesTorrenticolidae

(Marshall, 1943)

Atractides tahoei Marshall, 1943: 308.
Torrenticola tahoei Mitchell, 1954: 40.

######## Material examined.

HOLOTYPE (♀): from USA, California, Santa Cruz County, Waddell Creek, 28 April 1933, PR Needham, RM330007.

PARATYPES (1 ♀; 0 ♂): from USA, California, El Dorado County, South Lake Tahoe, Taylor Creek, 1 Sep 1932, by PR Needham, RM320005.

OTHER MATERIAL (49 ♀; 59 ♂): **British Columbia, Canada**: 2 ♀ and 1 ♂ from Ryan Rest Area off Highway 3, east of Yahk Moyie River, 15 Aug 2012, by IM Smith, IMS120071 • 1 ♀ and 1 ♂ from Vancouver Island, beside Highway 4, 35.6 kilometers east of Pacific Rim Road, 9 Jul 1976, by IM Smith, IMS760206 • 2 ♀ and 1 ♂ from Vancouver Island, Caycuse, Nixon Creek, 8 Jul 1976, by IM Smith, IMS760197& IMS760198 • 1 ♀ from Vancouver Island, Lake Cowichan, Cowichan River, above Skutz Falls, 9 Jul 1979, by IM Smith, IMS790035 • 1 ♀ and 2 ♂ from Vancouver Island, Lake Cowichan, Robertson River, South Shore Road, north of Mesachie Lake, 10 Jul 1976, by IM Smith, IMS760183A • 1 ♂ from Vancouver Island, Lake Cowichan, tributary of Robertson River, South Shore Road, north of Mesachie Lake, 10 Jul 1976, by IM Smith, IMS760183B • 2 ♀ and 2 ♂ from Vancouver Island, Lake Cowichan, Skutz Falls, Skutz Creek, near Cowichan River, 9 Jul 1979, by IM Smith, IMS790036A & IMS790036B • 1 ♀ and 1 ♂ from Vancouver Island, Lost Shoe Creek, beside Highway 4, 1.3 kilometers east of Pacific Rim Road, 9 Jul 1976, by IM Smith, IMS760203 • 1 ♀ from Vancouver Island, spring run beside South Shore Road, 2.3 kilometers north of Lake Cowichan, 6 Jun 1979, by IM Smith, IMS790007 • 1 ♀ and 1 ♂ from Vancouver Island, Ucluelet, beside Highway 4, 16.6 kilometers east of Pacific Rim Road, 18-19 Jul 1979, by IM Smith, IMS790047 • 1 ♀ from Vancouver Island, Youbou, Shaw Creek, North Shore Road, 4.3 kilometers south of north end of Cowichan Lake, 8 Jul 1976, by IM Smith, IMS760196 • **California, USA**: 1 ♂ from Calaveras County, Stanislaus National Forest, North Fork Stanislaus River (38°25'20"N, 120°2'47"W), 30 Aug 2013, by JR Fisher, JRF 13-0830-005 • 1 ♀ and 1 ♂ from El Dorado County, El Dorado National Forest, Taylor Creek (38°55'59"N, 120°3'21"W), 27 Aug 2013, by JR Fisher, JRF 13-0827-003 • 1 ♀ from Mendocino County, Jackson Demonstration State Park, North Fork of Big River (39°20'46"N, 123°30'35"W), 22 Aug 2013, by JR Fisher, JRF 13-0822-002 • 1 ♀ and 1 ♂ from Mendocino County, Cottaneva Creek, beside Route 1, 21.8 kilometers southwest of Route 101, 5 Aug 1987, by IM Smith, IMS870129A • 1 ♀ and 1 ♂ from Mendocino County, small stream at beach access road, off Route 1, 2.6 kilometers south of Westport, 5 Aug 1987, by IM Smith, IMS870128A • 2 ♀ from Monterey County, Nacimiento River, beside Nacimiento-Ferguson Road at Nacimiento campground, 30 Jul 1987, by IM Smith, IMS870120A • 1 ♀ and 1 ♂ from Monterey County, Salmon Creek, beside Route 1, south of Gorda, 28 Jul 1987, by IM Smith, IMS870114A • 1 ♀ and 1 ♂ from Monterey County, spring run on north side of Salmon Creek, beside Route 1, south Gorda, 29 Jul 1987, by IM Smith, IMS870117 • 1 ♂ from Nevada County, Tahoe National Forest, Sagehen Creek (39°26'2"N, 120°12'17"W), 26 Aug 2013, by JR Fisher, JRF 13-0826-006 • 1 ♀ and 1 ♂ from Plumas County, beside Route 89, north of Greenville, 14 Jun 1976, by IM Smith, IMS760113 • 3 ♀ and 3 ♂ from Shasta County, Battle Creek, beside Route 44, 5.6 kilometers west of Viola, 10 Aug 1987, by IM Smith, IMS870139A • 1 ♂ from Trinity County, small cascading trickle beside Route 36, 5.2 kilometers west of Forest Glen Station, 6 Aug 1987, by IM Smith, IMS870132 • 1 ♀ and 1 ♂ from Tulare County, Johnsondale, Double Bunk Creek, beside SM50, 2.2 kilometers east of Double Bunk Meadow, 31 Jul 1987, by IM Smith, IMS870123 • 1 ♂ from Tulare County, Kern River, Brush Creek Flat, beside SM99 between Roads End Station & Johnsondale, 31 Jul 1987, by IM Smith, IMS870121 • 1 ♂ from Tulare County, Stony Creek, Stony Creek Picnic Area, east of Sequoia National Park, 1 Aug 1987, by IM Smith, IMS870124A • 1 ♀ and 2 ♂ from Ventura County, Ojai, North Fork of Ventura River, beside Route 33, just above Wheeler Gorge, 25-26 Jul 1987, by IM Smith, IMS870109A & IMS870109B • 1 ♀ and 1 ♂ from Ventura County, Ojai, North Fork of Ventura River just below Wheeler Gorge campground, beside Route 33, 27 Jul 1987, by IM Smith, IMS870112• **Idaho, USA**: 1 ♀ from Custer County, Challis National Forest, Squaw Creek (44°19'35"N, 114°28'15"W), 30 Jul 2012, by JR Fisher, WA Nelson, & JC O’Neill, ROW 12-0730-002 • 1 ♂ from Lemhi County, Salmon National Forest, Niapas Creek (45°8'15"N, 114°13'4"W), 2 Aug 2012, by JR Fisher, WA Nelson, & JC O’Neill, ROW 12-0802-003 • **Montana, USA**: 1 ♀ and 1 ♂ from Missoula County, Lolo National Forest, Lolo Creek (46°46'7"N, 114°27'53"W), 7 Aug 2012, by JR Fisher, WA Nelson, & JC O’Neill, ROW 12-0807-003 • 1 ♀ and 1 ♂ from Ravalli County, Bitterroot National Forest, West Fork Bitterroot River (45°54'38"N, 114°9'43"W), 6 Aug 2012, by JR Fisher, WA Nelson, & JC O’Neill, ROW 12-0806-003 • 1 ♀ from Ravalli County, Medicine Springs, Spring Gulch campground, East Fork of Bitterroot River, beside Route 93, 1 Jul 1985, by IM Smith, IMS850060 • **Oregon, USA**: 2 ♀ and 3 ♂ from Coos County, Siskiyou National Forest, Road 33 between Powers & Agness, Coal Creek, 2 Jul 1983, by IM Smith, IMS830015 • 1 ♂ from Curry County, Port Orford, Butler Bar campground, Elk River, 25 Jun 1976, by IM Smith, IMS760162 • 1 ♂ from Curry County, Port Orford, Butler Bar campground, Elk River, 25-26 Jun 1976, by IM Smith, IMS760163 • 1 ♀ and 2 ♂ from Curry County, Port Orford, Humbug Mountain State Park Picnic Area, beside Route 1, Brush Creek, 1 Jul 1983, by IM Smith, IMS830012 • 2 ♀ and 6 ♂ from Curry County, Port Orford, Humbug Mountain State Park Picnic Area, Brush Creek, beside Route 1, 3 Jul 1983, by IM Smith, IMS830020A & IMS830020B • 1 ♀ from Curry County, Port Orford, Humbug Mountain State Park Picnic Area, beside Route 1, 1 Jul 1983, by IM Smith, IMS830013 • 3 ♀ and 5 ♂ from Curry County, Sixes, Sixes River, beside road at mouth of Edson Creek, 4 Jul 1983, by IM Smith, IMS830021A & IMS830021B • 2 ♂ from Lane County, Gate Creek (44°8'48"N, 122°34'20"W), 11 Aug 2013, by JC O’Neill, & WA Nelson, JNOW 13-0811-001• 2 ♀ and 1 ♂ from Lincoln County, Blackberry campground, near Tidewater, Alsea River, 28 Jun 1983, by IM Smith & AB Smith, IMS830009 • 2 ♀ and 3 ♂ from Multnomah County, Columbia River Scenic Highway, Horsetail Falls, 27 Jun 1983, by IM Smith, IMS830005 • **Washington, USA**: 2 ♀ from Clallam County, Green Creek (48°10'45"N, 124°12'21"W), 24 Jul 2013, by JC O’Neill, & WA Nelson, JNOW 13-0724-005 • 1 ♂ from Clallam County, Olympic National Forest, Jimmy Come Lately Creek (47°59'5"N, 123°0'5"W), 23 Jul 2013, by JC O’Neill, & WA Nelson, JNOW 13-0724-001 • 1 ♀ from Clallam County, Whiskey Creek (48°8'23"N, 123°47'7"W), 24 Jul 2013, by JC O’Neill, & WA Nelson, JNOW 13-0724-004 • 1 ♀ and 1 ♂ from Lewis County, Gifford Pinchot National Forest, Snake Creek (46°38'52"N, 121°43'8"W), 23 Jul 2013, by JC O’Neill, & WA Nelson, JNOW 13-0723-006 • 2 ♂ from Snohomish County, Mount Baker National Forest, Marten River (48°4'19"N, 121°36'24"W), 28 Jul 2013, by JC O’Neill, & WA Nelson, JNOW 13-0728-002.

######## Type deposition.

Holotype (♀) and allotype (♂) deposited in the CNC.

######## Diagnosis.

*Torrenticola tahoei* are similar to the other member of Tahoei group, *T. oregonensis* by subcapitulum shape, having purple coloration restricted posteriorly and being distributed in the west and to members of the Nigroalba Group (*T. flangipalpa*, *T. nigroalba*, *T. solisorta*, and *T. dentirostra*) in having purple dorsal coloration restricted posteriorly. *T. tahoei* can be differentiated from the Nigroalba Group by being larger (dorsum length ♀ = 600–720 in *T. tahoei*, 475–565 in Nigroalba Group; ♂ = 560–650 in *T. tahoei*, 425–510 in Nigroalba Group) and distributed in the west (Nigroalba Group are eastern). *T. tahoei* can be differentiated from *T. oregonensis* by being smaller (dorsum length ♀ = 600–720 in *T. tahoei*, 760–840 in *T. oregonensis*; ♂ = 560–650 in *T. tahoei*, 690–820 in *T. oregonensis*; dorsum width ♀ = 430–515 in *T. tahoei*, 560–640 in *T. oregonensis*; ♂ = 400–460 in *T. tahoei*, 520–605 in *T. oregonensis*) and a more elongate subcapitulum (ventral length/width = 3.25–4.11 in *T. tahoei*, 2.63–2.74 in *T. oregonensis*).

######## Re-description.

**Female (Figure 250)** (n = 7) (holotype measurements in parentheses when available) with characters of the genus with following specifications.

**Dorsum** — (600–720 (640) long; 430–515 (515) wide) ovoid with purple coloration restricted posteriorly. Anterio-medial platelets (115–140 (132.5) long; 62.5–77.5 (72.5) wide). Anterio-lateral platelets (177.5–202.5 (192.5) long; 65–85 (75) wide) free from dorsal plate. Dgl-4 much closer to the edge of the dorsum than to the muscle scars (distance between Dgl-4 335–400 (380)). Dorsal plate proportions: dorsum length/width 1.24–1.44 (1.24); dorsal width/distance between Dgl-4 1.25–1.43 (1.36); anterio-medial platelet length/width 1.68–1.96 (1.83); anterio-lateral platelet length/width 2.31–2.76 (2.57); anterio-lateral/anterio-medial length 1.40–1.57 (1.45).

**Gnathosoma — Subcapitulum** (345–390 (390) long (ventral); 266–315 (315) long (dorsal); 95–110 (95) tall) elongate and colorless. Rostrum (142.5–157.5 (157.5) long; 40–47.5 (45) wide) elongate. Chelicerae (358–395 (395) long) with curved fangs (50–56 (52.5) long). Subcapitular proportions: ventral length/height 3.41–4.11 (4.11); rostrum length/width 3.11–3.81 (3.50). **Pedipalps** elongate (especially tibiae) with broad, flat, dentate, and anteriorly-directed ventral extensions on femora and broadly tuberculate, dentate ventral extensions on genua. Palpomeres: trochanter (32.5–40 long); femur (102.5–117.5 long); genu (72.5–85 long); tibia (87.5–97.5 long; 21.25–25 wide); tarsus (15–17.5 long). Palpomere proportions: femur/genu 1.38–1.52; tibia/femur 0.82–0.86; tibia length/width 3.89–4.22.

**Venter** — (770–850 (810) long; 466–565 (565) wide) mostly colorless with areas of purple coloration. Gnathosomal bay (95–112.5 (100) long; 65–80 (70) wide). Cxgl-4 far from apex. **Medial suture** (95–130 (122.5) long). **Genital plates** (160–172.5 (166.25) long; 145–160 (160) wide). Additional measurements: Cx-1 (266–301 (290) long (total); 159–195 (190) long (medial)); Cx-3 (297–340 (335) wide); anterior venter (285–322.5 (312.5) long) elongate. Ventral proportions: gnathosomal bay length/width 1.19–1.59 (1.43); anterior venter/genital field length 1.70–1.88 (1.88); anterior venter length/genital field width 1.84–2.08 (1.95); anterior venter/medial suture 2.37–3.11 (2.55).

**Male (Figure 251)** (n = 5) with characters of the genus with following specifications.

**Dorsum** — (560–650 long; 400–460 wide) ovoid with purple coloration restricted posteriorly. Anterio-medial platelets (112.5–125 long; 57.5–67.5 wide). Anterio-lateral platelets (163.75–200 long; 65–80 wide) free from dorsal plate. Dgl-4 much closer to the edge of the dorsum than to the muscle scars (distance between Dgl-4 300–370). Dorsal plate proportions: dorsum length/width 1.30–1.41; dorsal width/distance between Dgl-4 1.24–1.33; anterio-medial platelet length/width 1.70–1.96; anterio-lateral platelet length/width 2.38–2.71; anterio-lateral/anterio-medial length 1.44–1.65.

**Gnathosoma — Subcapitulum** (325–347.5 long (ventral); 244–268 long (dorsal); 92.5–106.25 tall) elongate and colorless. Rostrum (130–140 long; 40–45 wide) elongate. Chelicerae (320–353 long) with curved fangs (41–56 long). Subcapitular proportions: ventral length/height 3.25–3.57; rostrum length/width 3.06–3.31. **Pedipalps** elongate (especially tibia) with broad, flat, dentate, and anteriorly-directed ventral extensions on femora and broadly tuberculate, dentate ventral extensions on genua. Palpomeres: trochanter (35–37.5 long); femur (100–107.5 long); genu (67.5–75 long); tibia (82.5–92.5 long; 20–23.75 wide); tarsus (12.5–17.5 long). Palpomere proportions: femur/genu 1.43–1.48; tibia/femur 0.80–0.88; tibia length/width 3.67–4.13.

**Venter** — (720–790 long; 450–517 wide) mostly colorless with areas of purple coloration. Gnathosomal bay (95–105 long; 65–77.5 wide). Cxgl-4 far from apex. **Medial suture** (120–147.5 long). **Genital plates** (132.5–142.5 long; 110–122.5 wide). Additional measurements: Cx-1 (232–264 long (total); 138–183 long (medial)); Cx-3 (285–315 wide); anterior venter (305–325 long) elongate. Ventral proportions: gnathosomal bay length/width 1.27–1.54; anterior venter/genital field length 2.21–2.34; anterior venter length/genital field width 2.57–2.82; anterior venter/medial suture 2.18–2.54.

**Immatures** unknown.

######## Etymology.

Marshall (1943) did not specify an etymology for this specific epithet (*tahoei*).It surely refers to the collection locality of the paratype, Taylor’s Creek near Lake Tahoe, California, although the holotype was collected from Waddell Creek near the coast.

######## Distribution.

**Figure 249.**
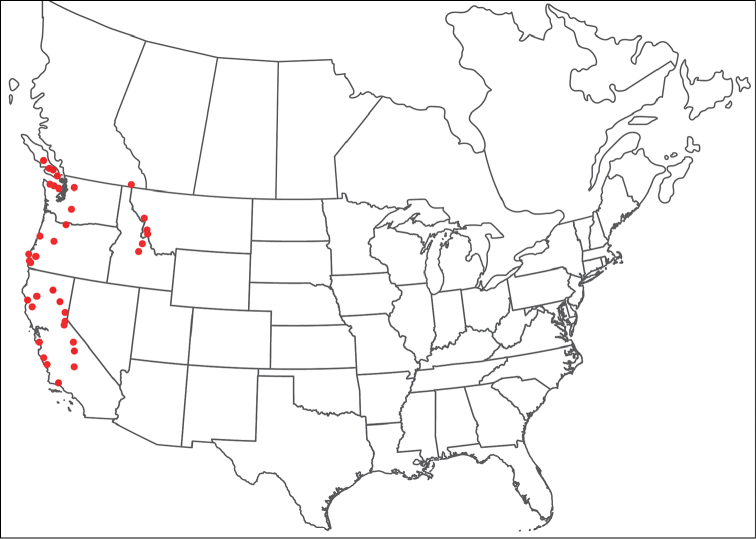
*Torrenticola tahoei* distribution.

Western, but not known from the southwest (Figure 249). *T. tahoei* was originally reported only from a few localities in California; we expand its known range into Oregon and Washington, and the Rocky Mountains.

**Figure 250.**
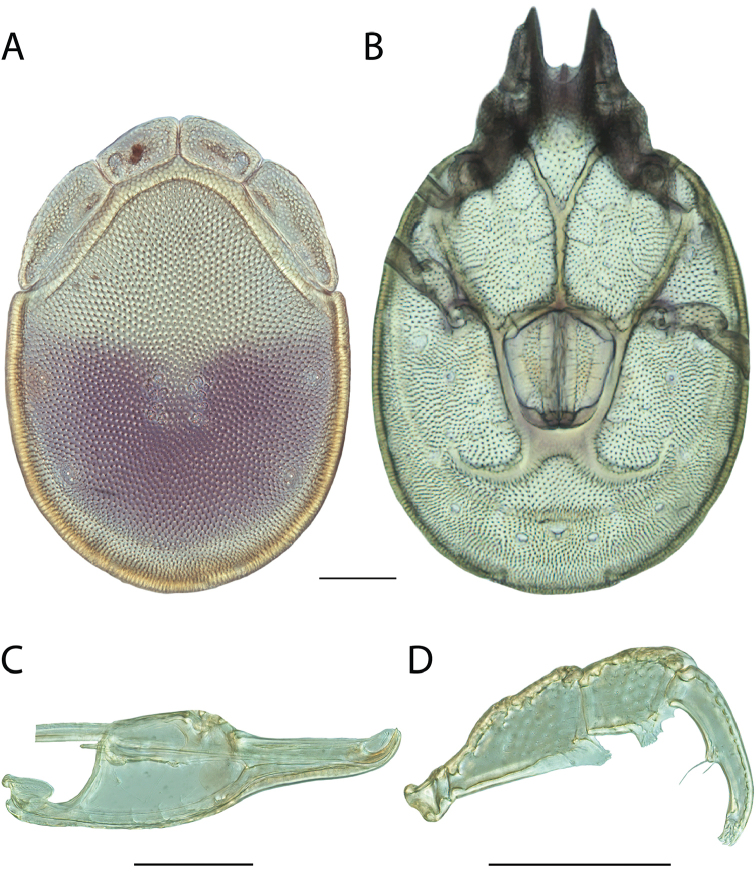
*Torrenticola tahoei* female: **A** dorsal plates **B** venter (legs removed) **C** subcapitulum **D** pedipalp (setae not accurately depicted). Scale = 100 µm.

**Figure 251.**
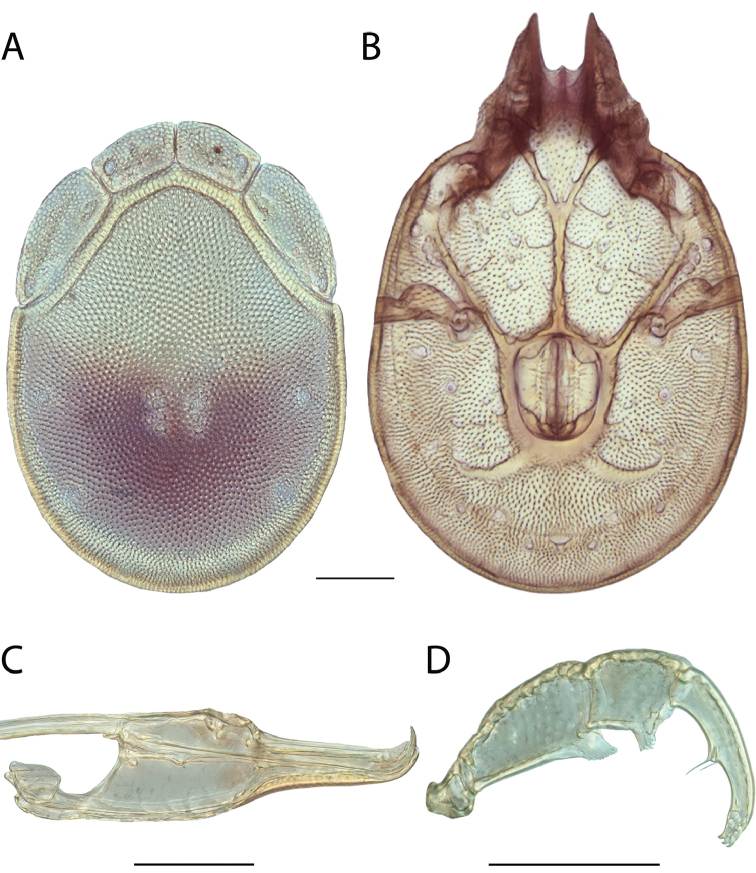
*Torrenticola tahoei* male: **A** dorsal plates **B** venter (legs removed) **C** subcapitulum **D** pedipalp (setae not accurately depicted). Scale = 100 µm.

######## Remarks.

*Torrenticola tahoei* groups with members of the Miniforma Complex with high support in all analyses. There is considerable genetic variability within this species (0–3.8%), particularly between specimens from California and those from elsewhere. However, we find no morphological characters to support California specimens being recognized as a separate species, so we consider them all one widely distributed species.

This species is so distinct and recognizable from all other species that we do not place it within an identification group. It is greater than 11.6% different in COI sequence from other members of the Miniforma group.

This species hypothesis is supported by high divergence between species (3–15%) and by the morphological characters outlined in the diagnosis.

####### Torrenticola tricolor

Taxon classificationAnimaliaTrombidiformesTorrenticolidae

Habeeb, 1957

Torrenticola tricolor Habeeb, 1957: 1.

######## Material examined.

HOLOTYPE (♂): from USA, New Jersey, Sussex County, Little Flatbrook, north of Bevans, 12 Oct 1953, by H Habeeb, HH530110.

PARATYPES (1 ♀; 0 ♂): **New Jersey, USA**: 1 ♀ (ALLOTYPE) from Sussex County, Little Flatbrook, north of Bevans, 12 Oct 1953, by H Habeeb, HH530110.

OTHER MATERIAL (15 ♀; 8 ♂): **Georgia, USA**: 1 ♀ and 2 ♂ from Chattooga County, East Fork of Little River, Cloudland (34°31'25"N, 85°30'23"W), 28 Sep 1992, by IM Smith, IMS920056A • **New Hampshire, USA**: 1 ♀ from Coos County, picnic area beside Route 110, Upper Ammonoosuc River (44°36'N, 71°24'W), 5 Jul 1989, by IM Smith, IMS890071 • **New Jersey, USA**: 1 ♀ and 1 ♂ from Warren County, Pequest River, 16 kilometers west of Hackettstown, 15 Aug 1964, by DR Cook, DRC640020 • **New York, USA**: 1 ♀ from Hamilton County, beside Route 8, 4.8 kilometers from Warren County line, 19 Aug 1964, by DR Cook, DRC640026 • **Nova Scotia, Canada**: 2 ♀ and 1 ♂ from Guysborough County, Sherbrooke, Sherbrooke Picnic Park beside Highway 7, 17 Sep 2011, by IM Smith, IMS110087 • **Ontario, Canada**: 1 ♀ from Hastings County, Moira River, Vanderwater Conservation Area, off Highway 37 south of Tweed, 20 Sep 1983, by IM Smith & CJ Hill, IMS830086C • 1 ♀ from Muskoka District, Baysville, shallow bay on south side of Echo Lake, 18 Aug 1981, by IM Smith, IMS810023 • **South Carolina, USA**: 1 ♀ from Greenville County, Matthews Creek, 24 Apr 2014, by D Eargle, JRF 14-0424-001 • **Tennessee, USA**: 1 ♀ from Blount County, Great Smoky National Park, Abrams River (35°35'30"N, 83°51'20"W), 17 Sep 2010, by IM Smith, IMS100142 • 1 ♀ from Monroe County, Tellico River (35°19'N, 84°10'W), 5 Jul 1990, by IM Smith, IMS900079 • 1 ♂ from Monroe County, Tellico River (35°20'27"N, 84°11'31"W), 12 Sep 2009, by IM Smith, IMS090111 • 3 ♀ and 1 ♂ from Sevier County, Great Smoky Mountains National Park, Middle Prong Little Pigeon River (35°43'33"N, 83°24'1"W), 12 Sep 2010, by IM Smith, IMS100131 • 1 ♂ from Sevier County, Great Smoky Mountains National Park, Middle Prong Little Pigeon River (35°44'12"N, 83°24'51"W), 12 Sep 2010, by IM Smith, IMS100132 • **Virginia, USA**: 1 ♂ from Alleghany County, Covington, Potts Creek, beside Route 18, 0.5 kilometers north of Route 657, 13 Jul 1990, by IM Smith, IMS900091B • 1 ♀ from Rappahannock County, Sperrysville, North Fork of Thornton River, beside Route 612, 0.5 miles from Route 211, 8 Sep 1968, by DR Cook, DRC680066.

######## Type deposition.

Holotype (♀) and allotype (♂) deposited in the CNC.

######## Diagnosis.

*Torrenticola tricolor* are similar to other members of the Tricolor Complex (*T. bittikoferae*, *T. hoosieri*, *T. larvata*, *T. pearsoni*, *T. olliei*, *T. sierrensis*, *T. trimaculata*, *T. unimaculata*, *T. cardia*, *T. kringi*, *T. dimorpha*, and *T. mohawk*) in having a short, conical rostrum. *T. tricolor* can be differentiated from all *Torrenticola*, including other members of the Tricolor Complex, by having a distinct dorsal pattern. Although the spots always have this basic construction, there is great variability between specimens. *T. tricolor* are most similar to other members of the Tricolor Complex that have bold patterning (*T. larvata*, *T. unimaculata*, *T. trimaculata*, *cardia*, *kringi*, and *T. mohawk*). *T. tricolor* can be further differentiated from *T. larvata* by being rounder (dorsum length/width = 1.2–1.38 in *T. tricolor*, 1.41–1.57 in *T. larvata*) and stockier pedipalp tibiae (length/width ♀ = 3.0–3.11 in *T. tricolor*, 3.25–3.5 in *T. larvata*; ♂ = 2.7–2.8 in *T. tricolor*, 3.1–3.2 in *T. larvata*). *T. tricolor* can be further differentiated from *T. trimaculata* by having longer genital field (♀ = 187.5–210 in *T. tricolor*, 157.5–185 in *T. trimaculata*; ♂ = 145–170 in *T. tricolor*, 120–140 in *T. trimaculata*) and a slightly more elongate rostrum (length/width ♀ = 2.14–2.39 in *T. tricolor*, 1.91–2.1 in *T. trimaculata*; ♂ = 2.37–2.5 in *T. tricolor*, 2.05–2.22 in *T. trimaculata*). *T. tricolor* can be further differentiated from *T. unimaculata* by having stockier pedipalp tibiae (♀ = 3.0–3.11 in *T. tricolor*, 3.2–3.4 in *T. unimaculata*; ♂ = 2.69–2.8 in *T. tricolor*, 2.9–3.11 in *T. unimaculata*) and shorter medial suture in females (15–22.5 in *T. tricolor*, 40–47.5 in *T. unimaculata*). *T. tricolor* can be further differentiated from *T. cardia* by having a rounder dorsum (length/width = 1.2–1.38 in *T. tricolor*, 1.39–1.54 in *T. cardia*) and longer pedipalpal genua (75–83 in *T. tricolor*, 60–70 in *T. cardia*). *T. tricolor* can be further differentiated from *T. kringi* by having a stockier rostrum (length/width = 2.14–2.50 in *T. tricolor*, 2.67–3.13 in *T. kringi*). *T. tricolor* can be differentiated from *T. mohawk* by having a more elongate rostrum (length/width ♀ = 2.14–2.39 in *T. tricolor*, 1.80–2.00 in *T. mohawk*; ♂ = 2.37–2.50 in *T. tricolor*, 2.00–2.13 in *T. mohawk*).

######## Re-description.

**Male (Figure 253)** (n = 6) (holotype measurements in parentheses when available) with characters of the genus with following specifications.

**Dorsum** — (580–660 (640) long; 435–520 (520) wide) circular to ellipsoid with reddish-purple or bluish-purple to navy blue coloration in two large posterior spots and a smaller anterior spot all merged together, often with orange between the posterior spots. Anterio-medial platelets (120–137.5 (137.5) long; 62.5–70 (70) wide). Anterio-lateral platelets (175–207.5 (192.5) long; 72.5–95 (95) wide) free from dorsal plate. Dgl-4 usually closer to the edge of the dorsum than to the muscle scars, occasionally halfway between the dorsum edge and muscle scars (distance between Dgl-4 295–375 (325)). Dorsal plate proportions: dorsum length/width 1.23–1.38 (1.23); dorsal width/distance between Dgl-4 1.33–1.60 (1.60); anterio-medial platelet length/width 1.92–2.00 (1.96); anterio-lateral platelet length/width 2.03–2.55 (2.03); anterio-lateral/anterio-medial length 1.40–1.58 (1.40).

**Gnathosoma — Subcapitulum** (247.5–295 (247.5) long (ventral); 197.5–226 (197.5) long (dorsal); 105–122.5 (112.5) tall) colorless. Rostrum (107.5–125 (107.5) long; 45–52.5 (45) wide) short and conical. Chelicerae (254–290 (260) long) with curved fangs (42–56 (52.5) long). Subcapitular proportions: ventral length/height 2.20–2.62 (2.20); rostrum length/width 2.37–2.50 (2.39). **Pedipalps** with tuberculate ventral extensions on femora and genua. Palpomeres: trochanter (37.5–45 (37.5) long); femur (95–110 (95) long); genu (70–80 (75) long); tibia (76.25–90 (76.25) long; 28.75–32.5 (28.75) wide); tarsus (22.5–27.5 (22.5) long). Palpomere proportions: femur/genu 1.27–1.45 (1.27); tibia/femur 0.80–0.86 (0.80); tibia length/width 2.65–2.80 (2.65).

**Venter** — (720–804 (790) long; 448–660 (660) wide) colorless. Gnathosomal bay (110–145 (126.25) long; 75–97.5 (85) wide). Cxgl-4 subapical. **Medial suture** (105–137.5 (105) long). **Genital plates** (145–170 (157.5) long; 107.5–115 (107.5) wide). Additional measurements: Cx-1 (271–290.5 (280) long (total); 139–163 (150) long (medial)); Cx-3 (316–401 (382.5) wide); anterior venter (255–297.5 (262.5) long). Ventral proportions: gnathosomal bay length/width 1.33–1.61 (1.49); anterior venter/genital field length 1.67–1.92 (1.67); anterior venter length/genital field width 2.37–2.77 (2.44); anterior venter/medial suture 2.16–2.50 (2.50).

**Female (Figure 254)** (n = 6) (allotypic measurements in parentheses when available) with characters of the genus with following specifications.

**Dorsum** — (600–755 (610) long; 470–575 (510) wide) circular to ellipsoid with reddish-purple or bluish-purple to navy blue coloration in two large posterior spots and a smaller anterior spot all merged together, often with orange between the posterior spots. Anterio-medial platelets (135–148.75 (137.5) long; 66.25–75 (70) wide). Anterio-lateral platelets (195–207.5 (195) long; 82.5–100 (100) wide) free from dorsal plate. Dgl-4 usually closer to the edge of the dorsum than to the muscle scars, occasionally halfway between the dorsum edge and muscle scars (distance between Dgl-4 320–410 (320)). Dorsal plate proportions: dorsum length/width 1.20–1.35 (1.20); dorsal width/distance between Dgl-4 1.36–1.59 (1.59); anterio-medial platelet length/width 1.93–2.20 (1.96); anterio-lateral platelet length/width 1.95–2.52 (1.95); anterio-lateral/anterio-medial length 1.33–1.46 (1.42).

**Gnathosoma — Subcapitulum** (285–330 (285) long (ventral); 210–251 (210) long (dorsal); 132.5–142.5 (132.5) tall) colorless. Rostrum (112.5–137.5 (112.5) long; 52.5–57.5 (52.5) wide) short and conical. Chelicerae (300–332 (300) long) with curved fangs (49–63 (52.5) long). Subcapitular proportions: ventral length/height 2.15–2.42 (2.15); rostrum length/width 2.14–2.39 (2.14). **Pedipalps** with tuberculate ventral extensions on femora and genua. Palpomeres: trochanter (42.5–47.5 (42.5) long); femur (107.5–120 (107.5) long); genu (75–82.5 (75) long); tibia (90–105 (90) long; 30–33.75 (30) wide); tarsus (22.5–27.5 (22.5) long). Palpomere proportions: femur/genu 1.41–1.48 (1.43); tibia/femur 0.84–0.89 (0.84); tibia length/width 3.00–3.11 (3.00).

**Venter** — (690–890 (740) long; 561–665 (665) wide) colorless. Gnathosomal bay (145–165 (145) long; 91.25–102.5 (91.25) wide). Cxgl-4 subapical. **Medial suture** (15–22.5 (20) long). **Genital plates** (187.5–210 (187.5) long; 155–171.25 (167.5) wide). Additional measurements: Cx-1 (265–311 (265) long (total); 125–151.75 (125) long (medial)); Cx-3 (375–433 (375) wide); anterior venter (152.5–192.5 (152.5) long). Ventral proportions: gnathosomal bay length/width 1.53–1.70 (1.59); anterior venter/genital field length 0.81–0.95 (0.81); anterior venter length/genital field width 0.91–1.18 (0.91); anterior venter/medial suture 7.63–11.83 (7.63).

**Immatures** unknown.

######## Etymology.

Although Habeeb (1957) did not specify an etymology for the specific epithet (*tricolor*), it surely refers to his comment on the dorsal coloration, “color black with some red and white” (*tres*, L. three; *color*, L. color).

######## Distribution.

**Figure 252.**
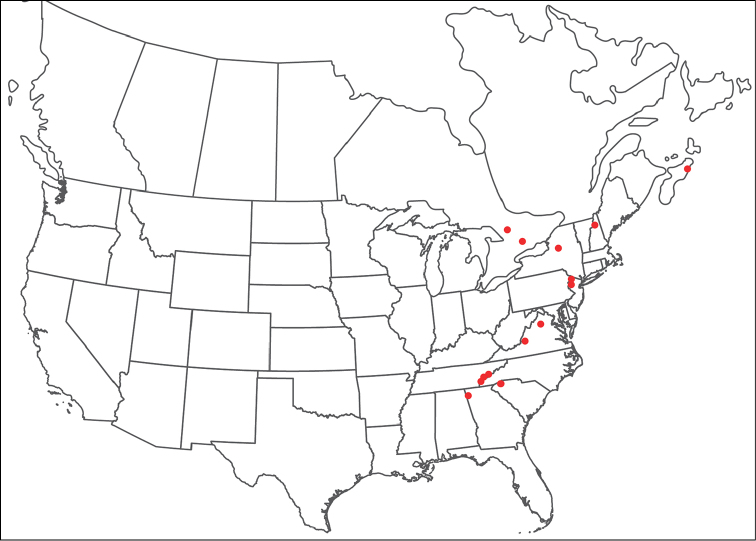
*Torrenticola tricolor* distribution.

Northeastern and southward throughout the Appalachians (Figure 252). *T. tricolor* was previously known only from northern New Jersey.

**Figure 253.**
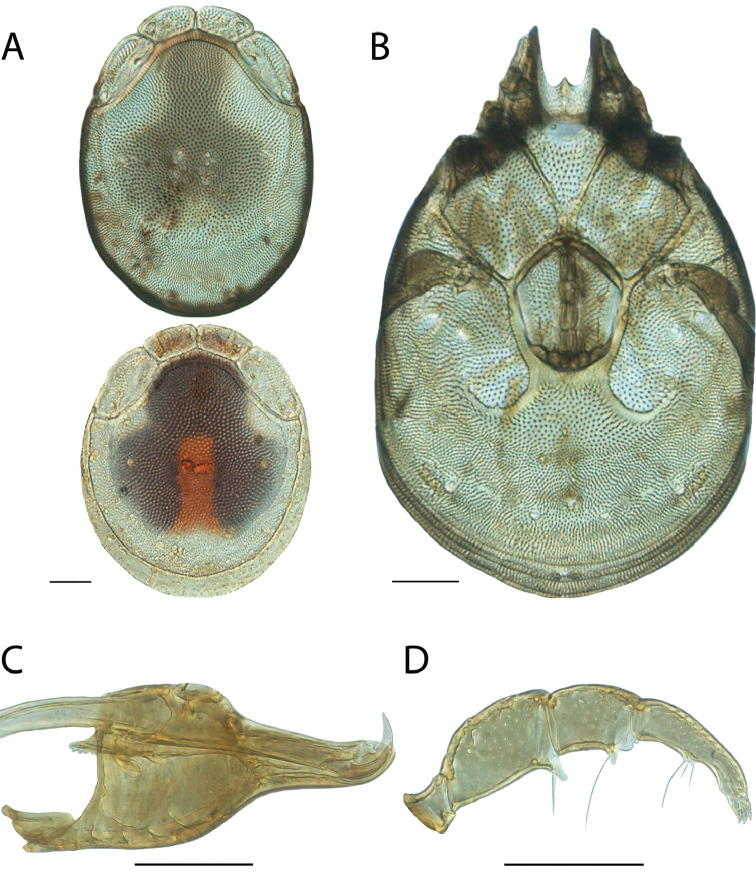
*Torrenticola tricolor* female: **A** dorsal plates, note color variation **B** venter (legs removed) **C** subcapitulum **D** pedipalp (setae not accurately depicted). Scale = 100 µm.

**Figure 254.**
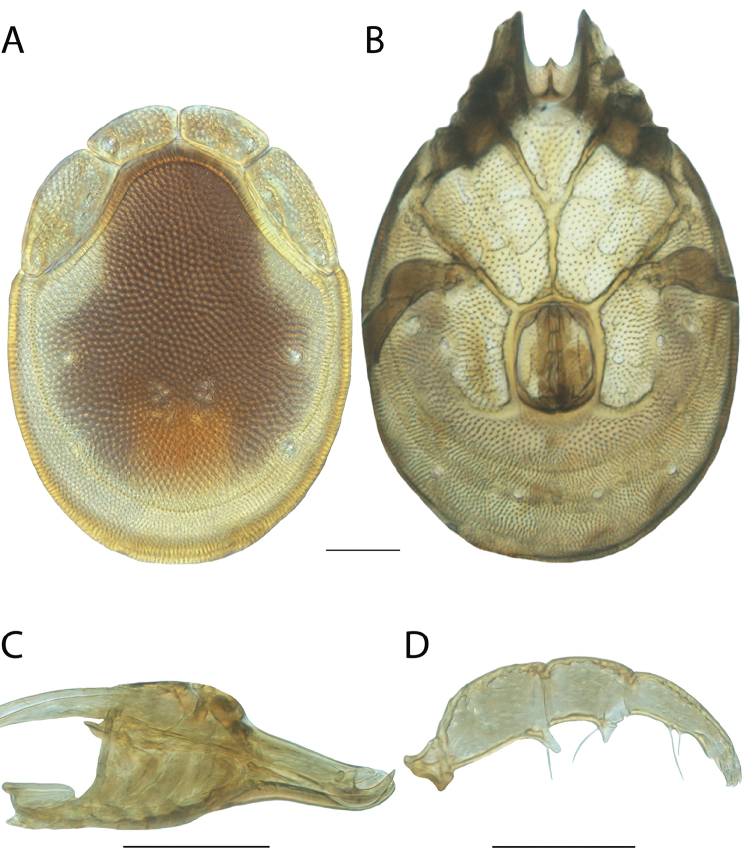
*Torrenticola tricolor* male: **A** dorsal plates **B** venter (legs removed) **C** subcapitulum **D** pedipalp (setae not accurately depicted). Scale = 100 µm.

######## Remarks.

*Torrenticola tricolor* group with other members of the Tricolor Complex with high support and specimens of this species are less than 2.4% different in COI sequence from each other. This within-species sequence variability is higher than in many species hypotheses presented herein. However, given the topology in the COI tree (Figure 14) and morphological similarity, it seems apparent that the variability represents a continuum across a large distribution, rather than isolated species. In all analyses, *T. tricolor* group with two other species which also have dorsal spots: *T. trimaculata* and *T. unimaculata*. This clade represents some of the most distinctive of all *Torrenticola*. This species is greater than 7% different in COI from sister species.

This species hypothesis is supported by high divergence between species (3–15%), and by the morphological characters outlined in the diagnosis.

####### Torrenticola trimaculata

Taxon classificationAnimaliaTrombidiformesTorrenticolidae

Fisher, 2015

Torrenticola trimaculata Fisher et al. (2015): 71, 89.

######## Material examined.

HOLOTYPE (♀): USA, Arkansas, Madison County, Withrow Springs State Park, War Eagle Creek (36°8'59.3"N, 93°44'26.94"W), 27 Jul 2011, by IM Smith, IMS110034.

PARATYPES (49 ♀; 37 ♂): **Arkansas, USA**: 1 ♂ (ALLOTYPE) from Madison County, Withrow Springs State Park, War Eagle Creek (36°8'59.3"N, 93°44'26.94"W), 27 Jul 2011, by IM Smith, IMS110034 • 2 ♀ and 3 ♂ from Madison County, Withrow Springs State Park, War Eagle Creek (36°8'59.3"N, 93°44'26.94"W), 27 Jul 2011, by IM Smith, IMS110034 • 1 ♀ from Marion County, Crooked Creek ex. Northern hogsucker (*Hypentelium nigricans*) (36°15'9.9"N, 94°26'25.8"W), 22 Jul 2014, by CT McAllister • 3 ♀ and 2 ♂ from Montgomery County, Ouachita National Forest, Ouachita River (34°34'53.20"N, 93°53'0.16"W), 5 Oct 2007, by AJ Radwell, & HW Robison, AJR070300A • 8 ♀ and 5 ♂ from Montgomery County, Ouachita National Forest, South Fork of Ouachita River, 29 Jul 2011, by AJ Radwell, & B Crump, AJR110302 • 2♀ and 1 ♂ from Montgomery County, Ouachita National Forest, Ouachita River, 27 Aug 2011, by AJ Radwell, AJR110307 • 4 ♀ and 4 ♂ from Montgomery County, Ouachita National Forest, South Fork of Ouachita River, 29 Jul 2011, by IM Smith, IMS110040 • 1 ♀ from Montgomery County, Caddo River, 29 Jul 2011, by IM Smith, IMS110037 • 1 ♂ from Newton County, Ozark National Forest, Mill Creek (36°3'42.12"N, 93°8'7.62"W), 20 Jun 2012, by TD Edwards, TDE 12-0620-010 • 2 ♀ and 2 ♂ from Newton County, Ozark National Forest, Little Buffalo River, 2 Sep 2012, by TD Edwards, TDE 12-0902-003 • 1 ♂ from Newton County, Buffalo National River, Whiteley Creek (35°59'28.14"N, 93°23'57.24"W), 23 May 2012, by TD Edwards, TDE 12-0523-002 • **Illinois, USA**: 2 ♀ and 1 ♂ from Union County, Clear Creek (37°33'N, 89°23'W), 13 Sep 1991, by IM Smith, IMS910036A • **Indiana, USA**: 1 ♀ from Wayne County (39°51'13"N, 85°8'4"W), 24 Jul 2014, by MJ Skvarla, MS 14-0731-001 • **Georgia, USA**: 1 ♀ from Chattooga County, Johns Creek (34°34'N, 80°5'W), 4 Jul 1990, by IM Smith, IMS900076 • **Kentucky, USA**: 1 ♀ and 2 ♂ from McCreary County, Rock Creek (36°42'N, 84°36'W), 8 Jul 1990, by IM Smith, IMS900082B • **Michigan, USA**: 2 ♀ and 2 ♂ from Barry County, Thornapple River (42°39'N, 85°17'W), 29 Jul 1959, by DR Cook, DRC590034 • **Missouri, USA**: 2 ♀ and 1 ♂ from Crawford County, Huzzah Creek, 23 Jul 2011, by IM Smith, IMS110029 • **New York, USA**: 3 ♀ and 1 ♂ from St. Lawrence County, Canton (44°35'N, 75°10'W), 15 May 1986, by BP Smith, BPS860508 • 1 ♀ from USA, New York, Delaware Co., Roscoe (41°55'N, 74°54'W), 11 June 1988, by PW Schefter and R MacCulloch, IMS880110 • **Nova Scotia, Canada**: 1 ♀ from Victoria County, Baddeck River (44°52'N, 61°5'W), 18 Jul 1981, by IM Smith, IMS810082 • **Ontario, Canada**: 4 ♀ and 2 ♂ from Grey County, Saugeen River (44°10'N, 80°49'W), 9 Jun 1989, by IM Smith, IMS890028A • 1 ♀ from Madoc (44°30'N, 77°28'W), 4 May 1980, by IM Smith, IMS800003A • 1 ♂ from Renfrew County, Madawaska River (45°21'N, 76°40'W), 25 May 1980, by IM Smith, IMS800012 • 1 ♀ and 1 ♂ from Lanark County, Mississippi River (45°3'N, 76°23'W), 6 Oct 1983, by IM Smith and CJ Hill, IMS830093A • **Texas, USA**: 1 ♀ from Gillespie County, Fredericksburg, Pedernales River (30°14'42"N, 98°54'50"W), 30 May 1998, by IM Smith, IMS980029 • **Virginia, USA**: 1 ♀ and 1 ♂ from Scott County, North Fork of Holston River (36°39'N, 82°28'W), 7 Jul 1990, by IM Smith, IMS0900080 • 2 ♀ and 4 ♂ from Alleghany County, Potts Creek (37°44'N, 80°2'W), 13 Jul 1990, by IM Smith, IMS900091B • 1 ♀ and 1 ♂ from Bath County, Jackson River (38°8'N, 79°46'W), 16 Jul 1990, by IM Smith, IMS900100 • **West Virginia, USA**: 2 ♀ from Pendleton County, North Fork of South Branch of Potomac River (39°0'N, 79°22'W), 17 Jul 1990, by IM Smith, IMS900104.

######## Type deposition.

Holotype (♀), allotype (♂), and other paratypes (44 ♀; 32 ♂) deposited in the CNC; other paratypes (4 ♀; 4 ♂) deposited in ACUA.

######## Diagnosis.

*Torrenticola trimaculata* are similar to other members of the Tricolor Complex (*T. bittikoferae*, *T. hoosieri*, *T. larvata*, *T. pearsoni*, *T. olliei*, *T. sierrensis*, *T. tricolor*, *T. unimaculata*, *T. cardia*, *T. kringi*, *T. dimorpha*, and *T. mohawk*) in having a short, conical rostrum. *T. trimaculata* can be differentiated from all *Torrenticola*, including other members of the Tricolor Complex, by having a distinct dorsal pattern. Individuals are reported in two distinct morphs primarily based upon ventral coloration. *T. trimaculata* are most similar to other members of the Tricolor Complex that have bold patterning (*T. larvata*, *T. unimaculata*, *T. tricolor*, *T. cardia*, *T. kringi*, and *T. mohawk*). *T. trimaculata* can be further differentiated from *T. larvata* by being rounder (dorsum length/width = 1.2–1.37 in *T. trimaculata*, 1.41–1.57 in *T. larvata*); Dgl-4 closer to the muscle scars (dorsal width/distance between Dgl-4 = 1.49–1.69 in *T. trimaculata*, 1.18–1.35 in *T. larvata*); and a stockier rostrum (length/width = 1.91–2.22 in *T. trimaculata*, 2.32–2.53 in *T. larvata*). *T. trimaculata* can be further differentiated from *T tricolor* by having shorter genital field (♀ = 157.5–185 in *T. trimaculata*, 187.5–210 in *T. tricolor*; ♂ = 120–140 in *T. trimaculata*, 145–170 in *T. tricolor*) and a stockier rostrum (length/width ♀ = 1.91–2.1 in *T. trimaculata*, 2.14–2.39 in *T. tricolor*; ♂ = 2.05–2.22 in *T. trimaculata*, 2.37–2.5 in *T. tricolor*). *T. trimaculata* can be further differentiated from *T. unimaculata* by Dgl-4 closer to the muscles scars (dorsal width/distance between Dgl-4 = 1.49–1.69 in *T. trimaculata*, 1.23–1.41 in *T. unimaculata*) and females with shorter medial suture (♀ = 17.5–27.5 in *T. trimaculata*, 40–47.5 in *T. unimaculata*). *T. trimaculata* can be further differentiated from *T. cardia* by having Dgl-4 further from the edge of the dorsum (dorsum width/distance between Dgl-4 = 1.5–1.7 in *T. trimaculata*, 1.15–1.4 in *T. cardia*) and having a stockier rostrum (length/width ♀ = 1.191–2.10 in *T. trimaculata*, 2.24–2.50 in *T. cardia*; ♂ = 2.05–2.22 in *T. trimaculata*, 2.27–2.47 in *T. cardia*). *T. trimaculata* can be differentiated from *T. kringi* by having a stockier rostrum (length/width = 1.9–2.3 in *T. trimaculata*, 2.6–3.2 in *T. kringi*). *T. trimaculata* can be differentiated from *T. mohawk* by having a shorter anterior venter (♀ = 157–180 in *T. trimaculata*, 187–200 in *T. mohawk*, ♂ = 230–260 in *T. trimaculata*, 290–308 in *T. mohawk*). Additionally, female *T. trimaculata* can be differentiated from female *T. mohawk* by having longer pedipalpal tibiae (♀ = 90–97 in *T. trimaculata*, 77–88 in *T. mohawk*).

######## Re-description

**(amended from Fisher et al. 2015). Female (Figure 256)** (n = 6) (holotype measurements in parentheses when available) with characters of the genus with following specifications.

**Dorsum** — (570–725 (725) long; 455–550 (550) wide) circular to ellipsoid with bluish-purple to navy blue coloration in three distinct spots, one anteriorly and two posteriorly, and orange medially. Anterio-medial platelets (125–145 (145) long; 60–72.5 (67.5) wide). Anterio-lateral platelets (162.5–180 (180) long; 80–85 (82.5) wide) free from dorsal plate. Dgl-4 approaching midway between muscle scars and the dorsum edge (distance between Dgl-4 295–335 (325)). Dorsal plate proportions: dorsum length/width 1.20–1.32 (1.32); dorsal width/distance between Dgl-4 1.49–1.69 1.69); anterio-medial platelet length/width 1.93–2.19 (2.15); anterio-lateral platelet length/width 2.00–2.19 (2.18); anterio-lateral/anterio-medial length 1.23–1.30 (1.24).

**Gnathosoma — Subcapitulum** (250–280 (280) long (ventral); 202–252 (210) long (dorsal); 115–135 (130) tall) colorless or with bluish-purple to navy blue coloration. Rostrum (97.5–110 (107.5) long; 47.5–55 (52.5) wide) short and conical. Chelicerae (261–326 (270) long) with curved fangs (48–62 (55) long). Subcapitular proportions: ventral length/height 2.04–2.17 (2.15); rostrum length/width 1.91–2.10 (2.05). **Pedipalps** with tuberculate ventral extensions on femora and genua. Palpomeres: trochanter (37.5–42.5 (42.5) long); femur (97.5–107.5 (103.75) long); genu (65–77.5 (70) long); tibia (90–96.25 (92.5) long; 27.5–30 (27.5) wide); tarsus (22.5–25 (22.5) long). Palpomere proportions: femur/genu 1.39–1.50 (1.48); tibia/femur 0.88–0.92 (0.89); tibia length/width 3.17–3.36 (3.36).

**Venter** — (615–840 (840) long; 533–700 (700) wide) colorless or with variable amount of bluish-purple to navy blue coloration. Gnathosomal bay (130–145 (130) long; 80–90 (80) wide). Cxgl-4 subapical. **Medial suture** (17.5–27.5 (20) long). **Genital plates** (157.5–185 (180) long; 152.5–185 (180) wide). Additional measurements: Cx-1 (258–306.75 (260) long (total); 109–154 (145) long (medial)); Cx-3 (328–390 (390) wide); anterior venter (157.5–180 (180) long). Ventral proportions: gnathosomal bay length/width 1.49–1.75 (1.63); anterior venter/genital field length 0.90–1.03 (1.00); anterior venter length/genital field width 1.00–1.14 (1.14); anterior venter/medial suture 6.09–9.29 (9.00).

**Male (Figure 257)** (n = 5) (allotypic measurements in parentheses when available) with characters of the genus with following specifications.

**Dorsum** — (530–595 (595) long; 390–440 (435) wide) circular to ellipsoid with bluish-purple to navy blue coloration in three distinct spots, one anteriorly and two posteriorly, and orange medially. Anterio-medial platelets (107.5–130 (120) long; 55–67.5 (62.5) wide). Anterio-lateral platelets (70–87.5 (75) long; 70–87.5 (75) wide) free from dorsal plate. Dgl-4 approaching midway between muscle scars and the dorsum edge (distance between Dgl-4 250–285 (270)). Dorsal plate proportions: dorsum length/width 1.30–1.37 (1.37); dorsal width/distance between Dgl-4 1.51–1.61 (1.61); anterio-medial platelet length/width 1.92–2.00 (1.92); anterio-lateral platelet length/width 1.91–2.23 (2.23); anterio-lateral/anterio-medial length 1.29–1.46 (1.40).

**Gnathosoma — Subcapitulum** (207.5–242.5 (230) long (ventral); 175–215 (194.75) long (dorsal); 92.5–112.5 (102.5) tall) colorless or with bluish-purple to navy blue coloration. Rostrum (87.5–100 (93.75) long; 40–47.5 (42.5) wide) short and conical. Chelicerae (232–286 (251) long) with curved fangs (40–53 (49) long). Subcapitular proportions: ventral length/height 2.16–2.43 (2.24); rostrum length/width 2.05–2.22 (2.21). **Pedipalps** with tuberculate ventral extensions on femora and genua. Palpomeres: trochanter (32.5–40 (32.5) long); femur (85–95 (93.75) long); genu (60–70 (65) long); tibia (75–82.5 (75) long; 25–27.5 (27.5) wide); tarsus (20–25 (22.5) long). Palpomere proportions: femur/genu 1.34–1.44 (1.44); tibia/femur 0.80–0.88 (0.80); tibia length/width 2.73–3.00 (2.73).

**Venter** — (588–719 (715) long; 431–571 (568) wide) colorless or with variable amount of bluish-purple to navy blue coloration. Gnathosomal bay (82.5–112.5 (102.5) long; 62.5–77.5 (77.5) wide). Cxgl-4 subapical. **Medial suture** (87.5–105 (105) long). **Genital plates** (120–140 (127.5) long; 92.5–105 (95) wide). Additional measurements: Cx-1 (216–297 (261) long (total); 130–168 (152) long (medial)); Cx-3 (287–372 (349) wide); anterior venter (230–260 (255) long). Ventral proportions: gnathosomal bay length/width 1.32–1.55 (1.32); anterior venter/genital field length 1.79–2.04 (2.00); anterior venter length/genital field width 92.5–105 (95); anterior venter/medial suture 2.43–2.63 (2.43).

**Immatures** unknown.

######## Etymology.

Fisher et al. (2015) named the specific epithet (*trimaculata*) in reference to the dorsal coloration of three dark spots (*tres*, L. three; *macula*, L. spot).

######## Distribution.

**Figure 255.**
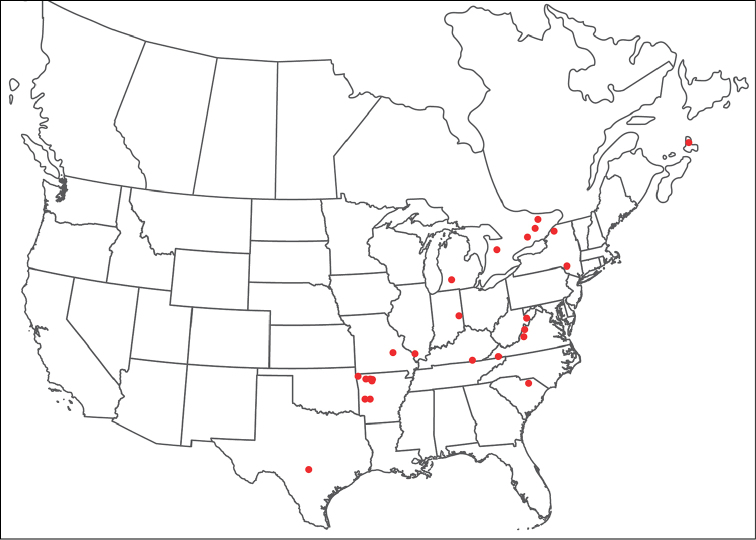
*Torrenticola trimaculata* distribution.

Eastern North America (Figure 255).

**Figure 256.**
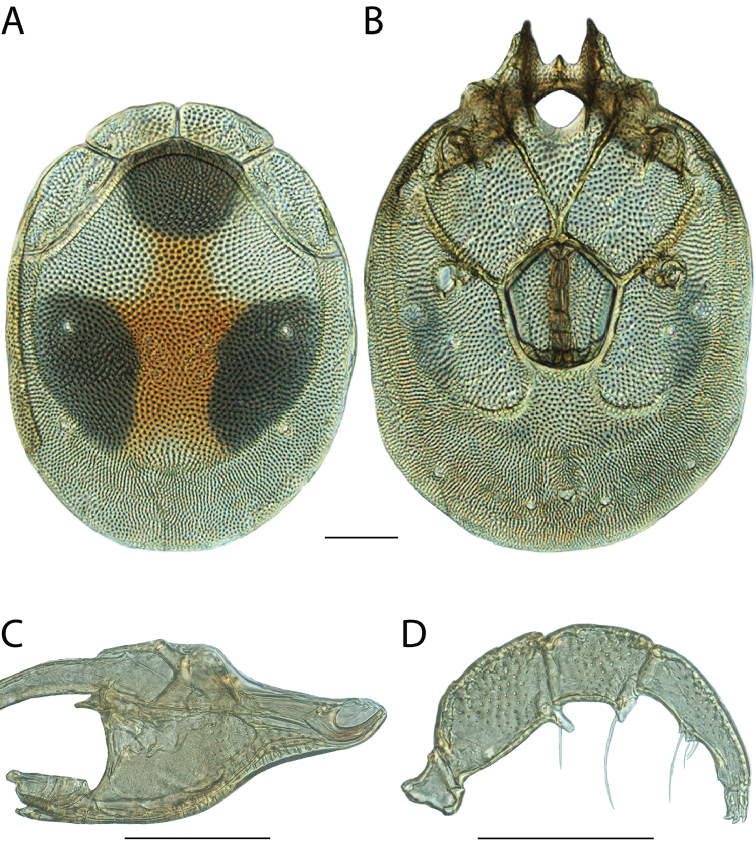
*Torrenticola trimaculata* female: **A** dorsal plates **B** venter (legs removed) **C** subcapitulum **D** pedipalp (setae not accurately depicted). Scale = 100 µm.

**Figure 257.**
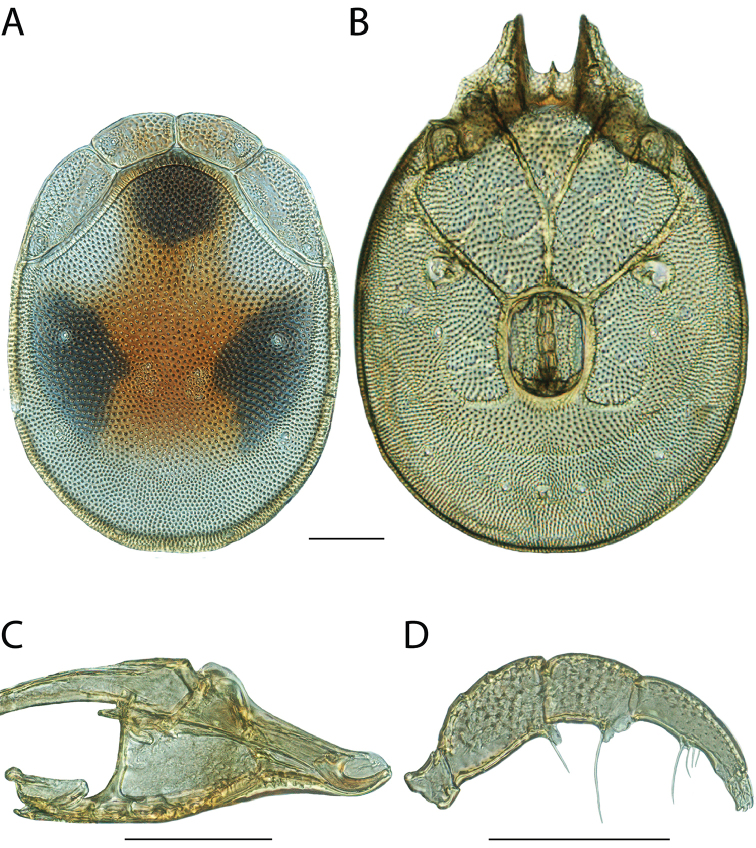
*Torrenticola trimaculata* male: **A** dorsal plates **B** venter (legs removed) **C** subcapitulum **D** pedipalp (setae not accurately depicted). Scale = 100 µm.

######## Remarks.

*Torrenticola trimaculata* group with other members of the Tricolor Complex with high support in all analyses. All specimens of this species are less than 2% different in COI sequence from each other and are greater than 7% from sister species. In all analyses, *T. trimaculata* groups with two other species which also have dorsal spots: *T. tricolor* and *T. unimaculata*. This clade represents some of the most distinctive of all *Torrenticola*.

This species hypothesis is supported by low COI divergence within the species (0–2%), high divergence between species (3–15%), and the morphological characters outlined in the diagnosis.

Fisher et al. (2015) reported two color morphs based primarily upon presence of ventral coloration. Morph I, with a dark venter, was only known from the Interior Highlands and all eastern specimens were reported as Morph II, with a colorless venter. However, we examined additional specimens from across eastern North America and found colored individuals mixed within samples of uncolored individuals. We remain unsure what controls color in *Torrenticola*, but with several other species exhibiting great color variation (e.g., *T. tricolor*, *T. gorti*), we do not consider it useful to continue with the “morph” concept in this species.

####### Torrenticola tysoni

Taxon classificationAnimaliaTrombidiformesTorrenticolidae

Fisher & Dowling sp. n.

http://zoobank.org/3F9D2DE0-5A5E-4055-8EE6-99F6070AA127

######## Material examined.

HOLOTYPE (♀): from USA, Alabama, Lauderdale County, off Natchez Trace Parkway, 7 km south of Tennessee state line (34°56'31"N, 87°49'41"W), 24 Sep 2009, by IM Smith, IMS090121, DNA 2871.

PARATYPES (6 ♀; 5 ♂): **Alabama, USA**: 1 ♂ (ALLOTYPE) from Lauderdale County, off Natchez Trace Parkway, 7 km south of Tennessee state line (34°56'31"N, 87°49'41"W), 24 Sep 2009, by IM Smith, IMS090121, DNA 2870 • 1 ♀ from Lauderdale County, off Natchez Trace Parkway, 7 km south of Tennessee state line (34°56'31"N, 87°49'41"W), 24 Sep 2009, by IM Smith, IMS090121 • 2 ♀ and 2 ♂ from Lauderdale County, off Natchez Trace Parkway, 7 km south of Tennessee state line (34°56'32"N, 87°49'43"W), 27 Sep 2010, by IM Smith, IMS100163 • 1 ♂ from Lauderdale County, off Natchez Trace Parkway, 7 km south of Tennessee state line (34°56'31"N, 87°49'41"W), 27 Sep 2010, by IM Smith, IMS100162 • **Tennessee, USA**: 3 ♀ and 1 ♂ from Wayne County, Glenrock Branch Creek (35°15'50"N, 87°37'34"W), 24 Sep 2009, by IM Smith, IMS090124.

######## Type deposition.

Holotype (♀), allotype (♂), and most paratypes (4 ♀; 2 ♂) deposited in the CNC; other paratypes (2 ♀; 2 ♂) deposited in ACUA.

######## Diagnosis.

*Torrenticola tysoni* are similar to other members of the Rusetria “Eastern 2-Plates” group (*T. biscutella*, *T. caerulea*, *T. delicatexa*, *T. indistincta*, *T. malarkeyorum*, *T. pendula*, *T. sellersorum*, *T. ululata*, *T. whitneyae*, *T. microbiscutella*, and *T. feminellai*) in having anterio-lateral platelets fused to the dorsal plate, having dorsal coloration separated into anterior and posterior portions (except *T. ululata* and *T. indistincta*), and being distributed in the east. It is one of only four Eastern 2-Plates that have dark, bold, bluish-purple coloration (also *T. biscutella*, *T. sellersorum*, and *T. pendula*). *T. tysoni* can be further differentiated from other Eastern 2-Plates by having a more elongate rostrum (length/width = ♀ = 3.06–3.31 in *T. tysoni*, 2.33–3.0 in others; ♂ = 3.14–3.50 in *T. tysoni*, 2.50–3.05 in others), except *T. feminellai* (3.05–3.38) and female *T. pendula* (3.0–3.06). *T. tysoni* can be differentiated from *T. feminellai* and *T. pendula* by dorsal coloration and pattern.

######## Description.

**Female (Figure 259)** (n = 5) (holotype measurements in parentheses when available) with characters of the genus with following specifications.

**Dorsum** — (610–670 (670) long; 450–475 (475) wide) ovoid with bold bluish-purple coloration separated into anterior and posterior portions, and with faint orange medially. Anterio-medial platelets (125–137.5 (135) long; 42.5–52.5 (52.5) wide). Anterio-lateral platelets (162.5–175 (172.5) long; 55–67.5 (67.5) wide) fused to dorsal plate. Dgl-4 much closer to the edge of the dorsum than to the muscle scars (distance between Dgl-4 320–330 (330)). Dorsal plate proportions: dorsum length/width 1.35–1.41 (1.41); dorsal width/distance between Dgl-4 1.38–1.44 (1.44); anterio-medial platelet length/width 2.50–3.18 (2.57); anterio-lateral platelet length/width 2.56–3.18 (2.56); anterio-lateral/anterio-medial length 1.18–1.40 (1.28).

**Gnathosoma — Subcapitulum** (310–327.5 (327.5) long (ventral); 230–245 (245) long (dorsal); 130–135 (135) tall) mostly colorless. Rostrum (125–132.5 (130) long; 40–42.5 (42.5) wide). Chelicerae (315–380 (350) long) with curved fangs (57.5–60 (60) long). Subcapitular proportions: ventral length/height 2.30–2.46 (2.43); rostrum length/width 3.06–3.31 (3.06). **Pedipalps** with tuberculate ventral extensions on femora and genua. Palpomeres: trochanter (37.5–47.5 (45) long); femur (112.5–120 (120) long); genu (62.5–70 (70) long); tibia (80–90 (87.5) long; 22.5–25 (25) wide); tarsus (17.5–22.5 (20) long). Palpomere proportions: femur/genu 1.61–1.80 (1.71); tibia/femur 0.70–0.80 (0.73); tibia length/width 3.50–3.67 (3.50).

**Venter** — (715–790 (790) long; 505–560 (540) wide) with bold bluish-purple coloration. Gnathosomal bay (162.5–172.5 (172.5) long; 72.5–100 (85) wide). Cxgl-4 subapical. **Medial suture** (13.75–22.5 (22.5) long). **Genital plates** (175–185 (180) long; 152.5–155 (155) wide). Additional measurements: Cx-1 (280–315 (310) long (total); 115–145 (140) long (medial)); Cx-3 (310–350 (335) wide); anterior venter (150–172.5 (172.5) long). Ventral proportions: gnathosomal bay length/width 1.65–2.24 (2.03); anterior venter/genital field length 0.81–0.99 (0.96); anterior venter length/genital field width 0.97–1.11 (1.11); anterior venter/medial suture 7.67–11.27 (7.67).

**Male (Figure 260)** (n = 5) (allotypic measurements in parentheses when available) with characters of the genus with following specifications.

**Dorsum** — (430–485 (460) long; 310–340 (320) wide) ovoid with bold bluish-purple coloration separated into anterior and posterior portions, and with faint orange medially. Anterio-medial platelets (97.5–127.5 (127.5) long; 35–40 (40) wide). Anterio-lateral platelets (127.5–137.5 (127.5) long; 42.5–50 (47.5) wide) fused to dorsal plate. Dgl-4 much closer to the edge of the dorsum than to the muscle scars (distance between Dgl-4 235–245 (240)). Dorsal plate proportions: dorsum length/width 1.39–1.48 (1.44); dorsal width/distance between Dgl-4 1.32–1.39 (1.33); anterio-medial platelet length/width 2.60–3.19 (3.19); anterio-lateral platelet length/width 2.65–3.00 (2.68); anterio-lateral/anterio-medial length 1.00–1.31 (1.00).

**Gnathosoma — Subcapitulum** (250–265 (250) long (ventral); 192.5–200 (195) long (dorsal); 77.5–95 (90) tall) mostly colorless. Rostrum (100–110 (100) long; 30–35 (30) wide). Chelicerae (237.5–260 (250) long) with curved fangs (45–47.5 (45) long). Subcapitular proportions: ventral length/height 2.76–3.23 (2.78); rostrum length/width 3.14–3.50 (3.33). **Pedipalps** with tuberculate ventral extensions on femora and genua. Palpomeres: trochanter (33.75–48.75 (37.5) long); femur (87.5–97.5 (92.5) long); genu (52.5–62.5 (57.5) long); tibia (67.5–75 (75) long; 20–21.25 (21.25) wide); tarsus (15–17.5 (15) long). Palpomere proportions: femur/genu 1.56–1.68 (1.61); tibia/femur 0.72–0.81 (0.81); tibia length/width 3.18–3.63 (3.53).

**Venter**— (540–585 (560) long; 350–410 (360) wide) with bold bluish-purple coloration. Gnathosomal bay (110–122.5 (112.5) long; 62.5–67.5 (62.5) wide). Cxgl-4 subapical. **Medial suture** (85–115 (85) long). **Genital plates** (110–117.5 (110) long; 87.5–92.5 (92.5) wide). Additional measurements: Cx-1 (240–250 (240) long (total); 115–127.5 (122.5) long (medial)); Cx-3 (250–290 (265) wide); anterior venter (227.5–250 (232.5) long). Ventral proportions: gnathosomal bay length/width 1.69–1.85 (1.80); anterior venter/genital field length 1.98–2.13 (2.11); anterior venter length/genital field width 2.51–2.86 (2.51); anterior venter/medial suture 2.17–2.74 (2.74).

**Immatures** unknown.

######## Etymology.

Specific epithet (*tysoni*) named in honor of Neil Degrasse Tyson for his efforts in popularizing cosmology and science in general with *Cosmos: A Spacetime Odyssey* (2014), which was a worthy update to Carl Sagan’s *Cosmos: A Personal Voyage* (1980).

######## Distribution.

**Figure 258.**
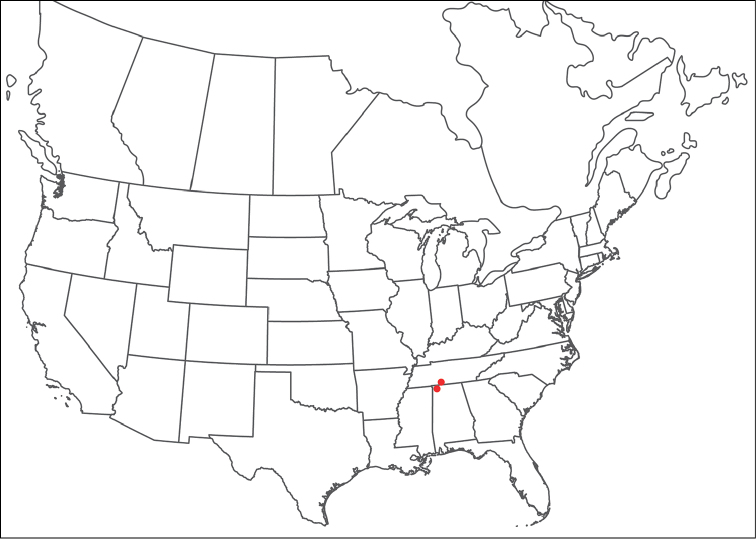
*Torrenticola tysoni* sp. n. distribution.

Known only from several localities along Natchez Trace Parkway (Figure 258).

**Figure 259.**
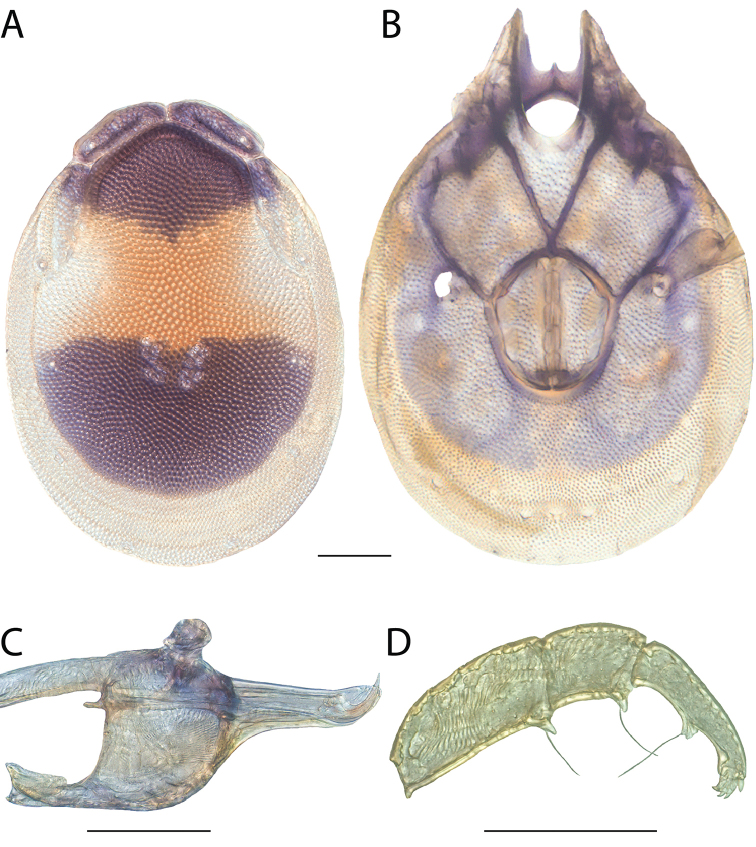
*Torrenticola tysoni* sp. n. female: **A** dorsal plates **B** venter (legs removed) **C** subcapitulum **D** pedipalp (setae not accurately depicted). Scale = 100 µm.

**Figure 260.**
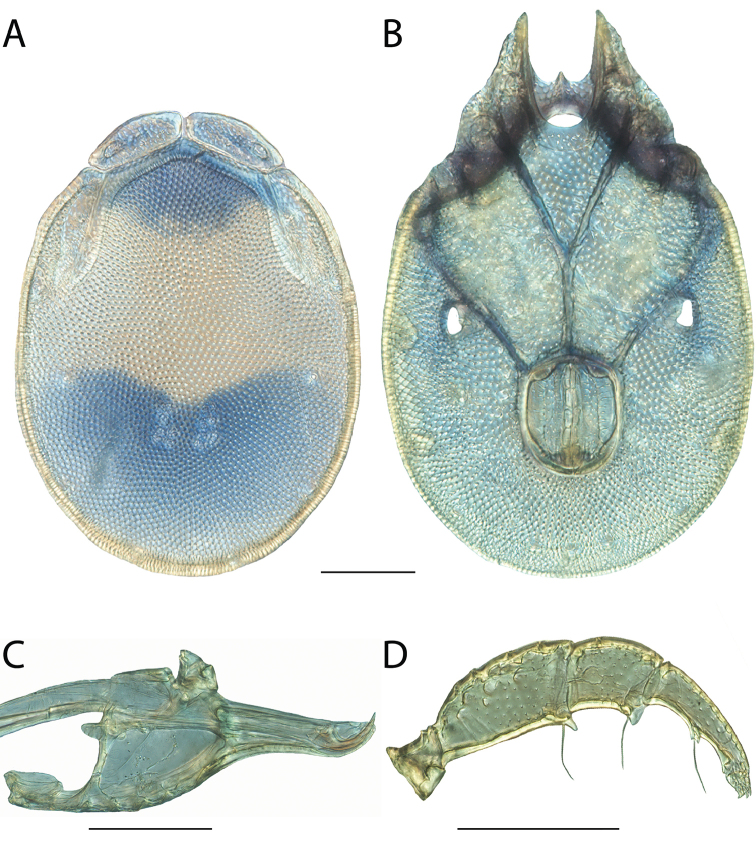
*Torrenticola tysoni* sp. n. male: **A** dorsal plates **B** venter (legs removed) **C** subcapitulum **D** pedipalp (setae not accurately depicted). Scale = 100 µm.

######## Remarks.

*Torrenticola tysoni* groups with other members of the Rusetria Complex with high support. Specimens of this species from Tennessee and Alabama are less than 1% different in COI sequence from each other and the specimen from Arkansas is less than 2% different from those. This species is greater than 10% different from sister species.

In all analyses, *T. tysoni* groups with two other species (*T. sellersorum* and *T. pendula*) as a paraphyletic grade at the base of the Eastern 2-Plate Identification Group. Based upon overall similarity, lateral platelets fused with the dorsal shield, and distribution, we were able to place this species within the Eastern 2-Plate Identification Group.

This species hypothesis is supported by low COI divergence within the species (0–2%) and high divergence between species (3–15%), and by the morphological characters outlined in the diagnosis.

####### Torrenticola ululata

Taxon classificationAnimaliaTrombidiformesTorrenticolidae

Fisher & Dowling sp. n.

http://zoobank.org/36729057-C4D7-4662-BF9B-B2854286F6CE

######## Material examined.

HOLOTYPE (♀): from USA, Mississippi, Attala County, Hurricane Creek (33°4'N, 89°32'W), 13 Oct 1999, by IM Smith, IMS990071.

PARATYPES (2 ♀; 4 ♂): **Alabama, USA**: 1 ♀ from Lauderdale County, off Natchez Trace parkway, 7 km south of Tennessee state line (34°56'32"N, 87°49'43"W), 24 Sep 2009, by IM Smith, IMS090122 • 2 ♂ from Lauderdale County, off Natchez Trace parkway, 7 km south of Tennessee state line (34°56'32"N, 87°49'43"W), 27 Sep 2010, by IM Smith, IMS100163 • **Mississippi, USA**: 1 ♂ (ALLOTYPE) from Attala County, Hurricane Creek (33°4'58"N, 89°31'31"W), 30 Sep 2010, by IM Smith, IMS100168 • 1 ♀ and 1 ♂ from Attala County, Hurricane Creek (33°4'58"N, 89°31'31"W), 30 Sep 2010, by IM Smith, IMS100168.

######## Type deposition.

Holotype (♀), allotype (♂), and most paratypes (1 ♀; 2 ♂) deposited in the CNC; other paratypes (1 ♀; 1 ♂) deposited in ACUA.

######## Diagnosis.

*Torrenticola ululata* are similar to other members of the Rusetria “Eastern 2-Plates” group (*T. biscutella*, *T. caerulea*, *T. delicatexa*, *T. indistincta*, *T. malarkeyorum*, *T. pendula*, *T. sellersorum*, *T. tysoni*, *T. whitneyae*, *T. microbiscutella*, and *T. feminellai*) in having anterio-lateral platelets fused to the dorsal plate, and being distributed in the east. *T. ululata* can be differentiated from most *Torrenticola*, including other Eastern 2-Plates, by having a distinct dorsal pattern with a single dark spot posteriorly and an orange spot posterior to the dark spot. The only other species with this pattern is *T. unimaculata* and *T. kringi*, which both have anterio-lateral platelets free from the dorsal plate.

######## Description.

**Female (Figure 262)** (n = 3) (holotype measurements in parentheses when available) with characters of the genus with following specifications.

**Dorsum** — (540–580 (570) long; 400–450 (450) wide) circular or occasionally ovoid with coloration restricted to a single dark spot anteriorly, often with an orange spot posterior to the dark spot. Anterio-medial platelets (130–137.5 (130) long; 50–55 (55) wide). Anterio-lateral platelets (150–180 (150) long; 72.5–77.5 (72.5) wide) fused to dorsal plate. Dgl-4 approaching midway between muscle scars and dorsum edge (distance between Dgl-4 265–295 (265)). Dorsal plate proportions: dorsum length/width 1.27–1.41 (1.27); dorsal width/distance between Dgl-4 1.36–1.70 (1.70); anterio-medial platelet length/width 2.36–2.70 (2.36); anterio-lateral platelet length/width 2.07–2.32 (2.07); anterio-lateral/anterio-medial length 1.15–1.31 (1.15).

**Gnathosoma — Subcapitulum** (290–305 (290) long (ventral); 216–235 (216) long (dorsal); 130–140 (130) tall) colorless. Rostrum (125–125 (125) long; 42.5–45 (42.5) wide). Chelicerae (289–317 (289) long) with curved fangs (59–64 (64) long). Subcapitular proportions: ventral length/height 2.18–2.23 (2.23); rostrum length/width 2.78–2.94 (2.94). **Pedipalps** with tuberculate ventral extensions on femora and genua. Palpomeres: trochanter (42.5–45 (45) long); femur (117.5–122.5 (117.5) long); genu (67.5–75 (67.5) long); tibia (97.5–105 (97.5) long; 21.25–25 (21.25) wide); tarsus (17.5–20 (17.5) long). Palpomere proportions: femur/genu 1.63–1.74 (1.74); tibia/femur 0.83–0.86 (0.83); tibia length/width 4.20–4.59 (4.59).

**Venter** — (595–670 (670) long; 517–540 (518) wide) colorless. Gnathosomal bay (152.5–160 (152.5) long; 85–102.5 (85) wide). Cxgl-4 subapical. **Medial suture** (15–15 (15) long). **Genital plates** (155–170 (155) long; 147.5–157.5 (147.5) wide). Additional measurements: Cx-1 (276–282 (281) long (total); 115–126 (120) long (medial)); Cx-3 (357–382 (358) wide); anterior venter 155–160 (160) long). Ventral proportions: gnathosomal bay length/width 1.56–1.79 (1.79); anterior venter/genital field length 0.93–1.03 (1.03); anterior venter length/genital field width 0.98–1.08 (1.08); anterior venter/medial suture 10.33–10.67 (10.67).

**Male (Figure 263)** (n = 4) (allotypic measurements in parentheses when available) with characters of the genus with following specifications.

**Dorsum** — (460–510 (500) long; 355–380 (380) wide) circular or occasionally ovoid with coloration restricted to a single dark spot anteriorly, often with an orange spot posterior to the dark spot. Anterio-medial platelets (107.5–117.5 (110) long; 42.5–50 (42.5) wide). Anterio-lateral platelets (145–155 (155) long; 55–60 (60) wide) fused to dorsal plate. Dgl-4 approaching midway between muscle scars and dorsum edge (distance between Dgl-4 235–250 (250)). Dorsal plate proportions: dorsum length/width 1.30–1.38 (1.32); dorsal width/distance between Dgl-4 1.45–1.57 (1.52); anterio-medial platelet length/width 2.26–2.59 (2.59); anterio-lateral platelet length/width 2.52–2.77 (2.58); anterio-lateral/anterio-medial length 1.32–1.41 (1.41).

**Gnathosoma — Subcapitulum** (227.5–255 (247.5) long (ventral); 165–190 (176) long (dorsal); 90–100 (100) tall) colorless. Rostrum (90–102.5 (100) long; 32.5–38.75 (37.5) wide). Chelicerae (225–254 (225) long) with curved fangs (34–49 (46) long). Subcapitular proportions: ventral length/height 2.48–2.55 (2.48); rostrum length/width 2.61–2.77 (2.67). **Pedipalps** with tuberculate ventral extensions on femora and genua. Palpomeres: trochanter (37.5–40 long); femur (92.5–100 (100) long); genu (57.5–65 (60) long); tibia (77.5–92.5 (85) long; 20–22.5 (20) wide); tarsus (15–17.5 (15) long). Palpomere proportions: femur/genu 1.54–1.67 (1.67); tibia/femur 0.84–0.93 (0.85); tibia length/width 3.88–4.25 (4.25).

**Venter** — (580–620 (580) long; 425–487 (426) wide) colorless. Gnathosomal bay (110–125 (110) long; 67.5–75 (67.5) wide). Cxgl-4 subapical. **Medial suture** (80–100 (100) long). **Genital plates** (117.5–122.5 (120) long; 102.5–110 (107.5) wide). Additional measurements: Cx-1 (246–257 (257) long (total); 124–129 (125) long (medial)); Cx-3 (283–326 (290) wide); anterior venter (220–257.5 (252.5) long). Ventral proportions: gnathosomal bay length/width 1.63–1.67 (1.63); anterior venter/genital field length 1.87–2.17 (2.10); anterior venter length/genital field width 2.05–2.49 (2.35); anterior venter/medial suture 2.53–3.19 (2.53).

**Immatures** unknown.

######## Etymology.

Specific epithet (*ululata*) refers to the dorsal coloration resembling a wailing mouth, where the dark anterior spot is the oral cavity and the posterior red spot is the tongue (*ululatus*, L. shriek, wail).

######## Distribution.

**Figure 261.**
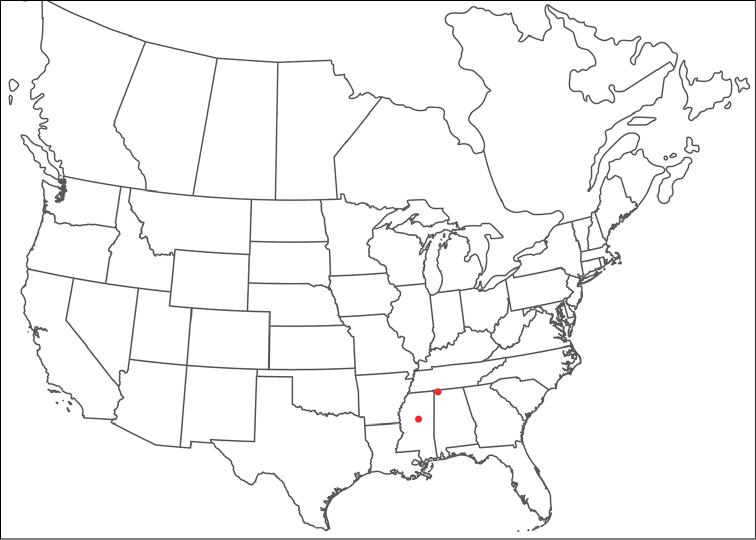
*Torrenticola ululata* sp. n. distribution.

Southeastern, Mississippi and Alabama (Figure 261).

**Figure 262.**
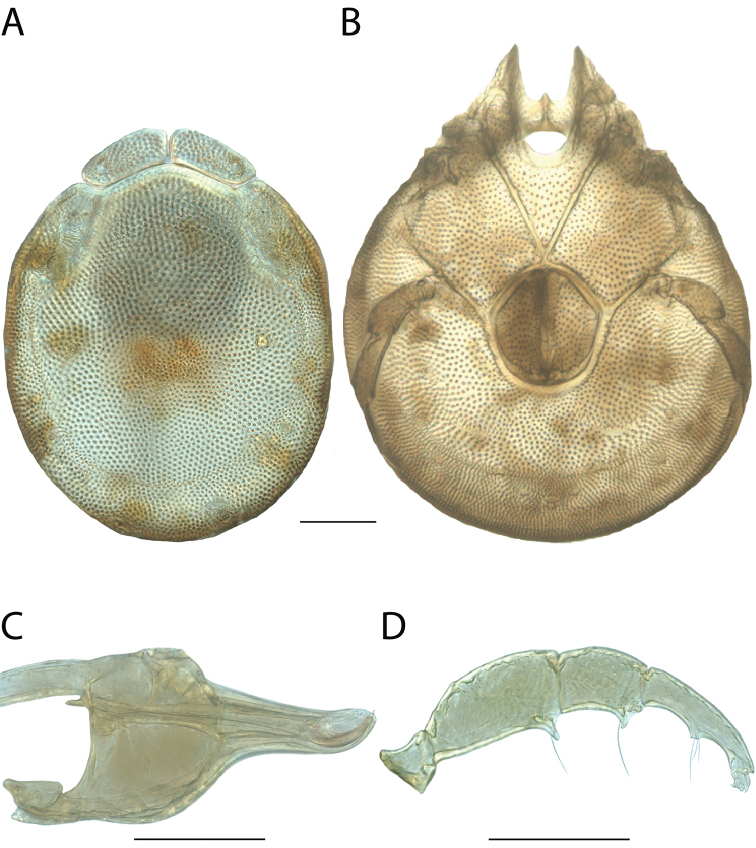
*Torrenticola ululata* sp. n. female: **A** dorsal plates **B** venter (legs removed) **C** subcapitulum **D** pedipalp (setae not accurately depicted). Scale = 100 µm.

**Figure 263.**
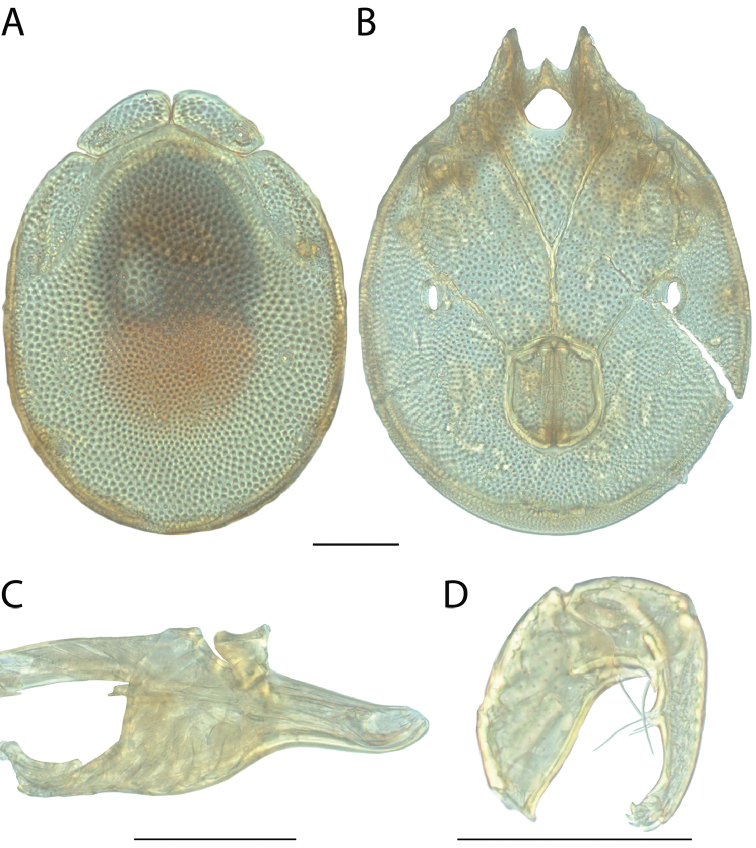
*Torrenticola ululata* sp. n. male: **A** dorsal plates **B** venter (legs removed) **C** subcapitulum **D** pedipalp (setae not accurately depicted). Scale = 100 µm.

######## Remarks.

*Torrenticola ululata* groups with other members of the Rusetria Complex with high support. The two specimens included in our molecular analyses were less than 1% different in COI sequence from each other and 11–12% different from sister species.

Based upon overall similarity, lateral platelets fused with the dorsal shield, and distribution, we were able to place this species within the Eastern 2-Plate Identification Group.

This species hypothesis is supported by low COI divergence within the species (0–2%) and high divergence between species (3–15%), and by the morphological characters outlined in the diagnosis.

####### Torrenticola unimaculata

Taxon classificationAnimaliaTrombidiformesTorrenticolidae

Fisher & Dowling sp. n.

http://zoobank.org/0B8A2822-71B9-4635-B799-96A7860C180F

######## Material examined.

HOLOTYPE (♀): from Canada, New Brunswick, York County, SW Mirimachi River, beside Highway 107 between Napdogan and Juniper, 21 Jun 2012, by IM Smith, IMS120036, DNA 3010.

PARATYPES (7 ♀; 7 ♂): **Arkansas, USA**: 2 ♀ and 1 ♂ from Montgomery County, Caddo Gap, access track off Manfred Road, 0.3 km west of Route 8, 29 Jul 2011, by IM Smith, IMS110037 • 4 ♀ from Montgomery County, Gaston, South Fork of Ouachita River, access off County Road 17 at Forest Road 903, 29 Jul 2011, by IM Smith, IMS110040 • 2 ♂ from Montgomery County, Ouachita River, Pine Ridge, 5 Oct 2007, by AJ Radwell, & HW Robison, AJR070300A • 2 ♂ from Montgomery County, Ouachita National Forest, South Fork of Ouachita River, 29 Jul 2011, by AJ Radwell, & B Crump, AJR110302 • **New Brunswick, Canada**: 1 ♂ (ALLOTYPE) from York County, SW Mirimachi River, beside Highway 107 between Napdogan and Juniper, 21 Jun 2012, by IM Smith, IMS120036, DNA 3011 • 1 ♀ and 1 ♂ from York County, SW Mirimachi River, beside Highway 107 between Napdogan and Juniper, 21 Jun 2012, by IM Smith, IMS120036.

######## Type deposition.

Holotype (♀), allotype (♂), and most paratypes (4 ♀; 3 ♂) deposited in the CNC; other paratypes (3 ♀; 3 ♂) deposited in ACUA.

######## Diagnosis.

*Torrenticola unimaculata* are similar to other members of the Tricolor Complex (*T. bittikoferae*, *T. hoosieri*, *T. larvata*, *T. pearsoni*, *T. olliei*, *T. sierrensis*, *T. tricolor*, *T. trimaculata*, *T. cardia*, *T. kringi*, *T. dimorpha*, and *T. mohawk*) in having a short, conical rostrum. *T. unimaculata* can be differentiated from most *Torrenticola*, including other members of the Tricolor Complex, by having a distinct dorsal pattern of a large anterior dorsal spot. The only other species with this pattern is *T. ululata*, which, like all Rusetria 2-Plates, have anterio-lateral platelets fused to the dorsal plate and *T. kringi*, which has a more elongate rostrum (length/width = 1.9–2.2 in *T. unimaculata*, 2.6–3.2 in *T. kringi*). *T. unimaculata* are most similar to other members of the Tricolor Complex that have bold patterning (*T. larvata*, *T. tricolor*, *T. trimaculata*, *T. cardia*, *T. kringi*, and *T. mohawk*). Female *T. unimaculata* can be further differentiated from these members of the complex by having a longer medial suture (♀ = 40–47.5 in *T. unimaculata*, 15–35 in others), except *T. cardia* (25–50) and *T. mohawk* (30–45).

######## Description.

**Female (Figure 265)** (n = 6) (holotype measurements in parentheses when available) with characters of the genus with following specifications.

**Dorsum** — (650–730 (720) long; 490–600 (600) wide) ovoid with coloration restricted to a single dark spot anteriorly (occasionally extending medially), with an orange spot posterior to the dark spot. Anterio-medial platelets (127.5–145 (140) long; 62.5–70 (67.5) wide). Anterio-lateral platelets (172.5–200 (200) long; 72.5–80 (80) wide) free from dorsal plate. Dgl-4 much closer to the edge of the dorsum than to the muscle scars (distance between Dgl-4 350–425 (425)). Dorsal plate proportions: dorsum length/width 1.20–1.39 (1.20); dorsal width/distance between Dgl-4 1.34–1.41 (1.41); anterio-medial platelet length/width 1.89–2.08 (2.07); anterio-lateral platelet length/width 2.29–2.57 (2.50); anterio-lateral/anterio-medial length 1.35–1.44 (1.43).

**Gnathosoma — Subcapitulum** (242.5–265 (265) long (ventral); 176.25–194 (190) long (dorsal); 110–125 (125) tall) colorless. Rostrum (85–100 (92.5) long; 42.5–47.5 (47.5) wide) short and conical. Chelicerae (236–252 (250) long) with curved fangs (51–61.5 (55) long). Subcapitular proportions: ventral length/height 2.12–2.26 (2.12); rostrum length/width 1.89–2.18 (1.95). **Pedipalps** with tuberculate ventral extensions on femora and genua. Palpomeres: trochanter (35–42.5 (42.5) long); femur (91.25–100 (100) long); genu (62.5–67.5 (67.5) long); tibia (80–87.5 (87.5) long; 25–26.25 (26.25) wide); tarsus (20–25 (22.5) long). Palpomere proportions: femur/genu 1.46–1.52 (1.48); tibia/femur 0.81–0.93 (0.88); tibia length/width 3.20–3.40 (3.33).

**Venter** — (700–860 (860) long; 539–630 (630) wide) colorless. Gnathosomal bay (110–140 (140) long; 75–87.5 (80) wide). Cxgl-4 subapical. **Medial suture** (40–47.5 (40) long). **Genital plates** (180–210 (210) long; 152.5–170 (170) wide). Additional measurements: Cx-1 (243–290 (290) long (total); 128–162 (145) long (medial)); Cx-3 (358–426 (390) wide); anterior venter (182.5–205 (195) long). Ventral proportions: gnathosomal bay length/width 1.40–1.75 (1.75); anterior venter/genital field length 0.93–1.14 (0.93); anterior venter length/genital field width 1.15–1.30 (1.15); anterior venter/medial suture 4.06–4.88 (4.88).

**Male (Figure 266)** (n = 6) (allotypic measurements in parentheses when available) with characters of the genus with following specifications.

**Dorsum**— (525–690 (690) long; 400–520 (520) wide) ovoid with coloration restricted to a single dark spot anteriorly (occasionally extending medially), with an orange spot posterior to the dark spot. Anterio-medial platelets (112.5–137.5 (137.5) long; 57.5–72.5 (72.5) wide). Anterio-lateral platelets (160–205 (205) long; 65–82.5 (82.5) wide) free from dorsal plate. Dgl-4 much closer to the edge of the dorsum than to the muscle scars (distance between Dgl-4 320–405 (405)). Dorsal plate proportions: dorsum length/width 1.30–1.43 (1.33); dorsal width/distance between Dgl-4 1.23–1.28 (1.28); anterio-medial platelet length/width 1.90–2.09 (1.90); anterio-lateral platelet length/width 2.28–2.54 (2.48); anterio-lateral/anterio-medial length 1.35–1.43 (1.33).

**Gnathosoma — Subcapitulum** (212.5–255 (255) long (ventral); 155–185 (185) long (dorsal); 90–110 (110) tall) colorless. Rostrum (80–95 (95) long; 37.5–48.75 (48.75) wide) short and conical. Chelicerae (195–235 (235) long) with curved fangs (42–55 (55) long). Subcapitular proportions: ventral length/height 2.32–2.42 (2.32); rostrum length/width 1.95–2.2 (1.95). **Pedipalps** with tuberculate ventral extensions on femora and genua. Palpomeres: trochanter (32.5–40 (40) long); femur (80–97.5 (97.5) long); genu (55–67.5 (67.5) long); tibia (70–80 (80) long; 22.5–27.5 (27.5) wide); tarsus (20–22.5 (22.5) long). Palpomere proportions: femur/genu 1.39–1.48 (1.44); tibia/femur 0.82–0.91 (0.82); tibia length/width 2.90–3.11 (2.91).

**Venter** — (640–800 (800) long; 448–570 (570) wide) colorless. Gnathosomal bay (97.5–132.5 (132.5) long; 62.5–77.5 (72.5) wide). Cxgl-4 subapical. **Medial suture** (107.5–125 (125) long). **Genital plates** (130–161.25 (161.25) long; 92.5–112.5 (112.5) wide). Additional measurements: Cx-1 (218–338 (290) long (total); 109–165 (165) long (medial)); Cx-3 (218–338 (290) wide); anterior venter (265–300 (300) long). Ventral proportions: gnathosomal bay length/width 1.44–1.83 (1.83); anterior venter/genital field length 1.86–2.13 (1.86); anterior venter length/genital field width 2.67–2.92 (2.67); anterior venter/medial suture 2.30–2.49 (2.40).

**Immatures** unknown.

######## Etymology.

Specific epithet (*unimaculata*) named for the single dark dorsal spot of this species (*unus*, L. one; *macula*, L. spot).

######## Distribution.

**Figure 264.**
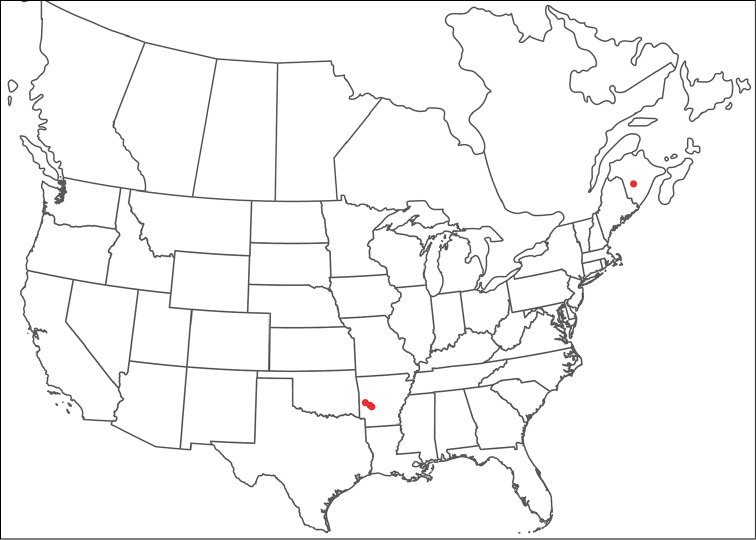
*Torrenticola unimaculata* sp. n. distribution.

Known only from Arkansas and New Brunswick (Figure 264).

**Figure 265.**
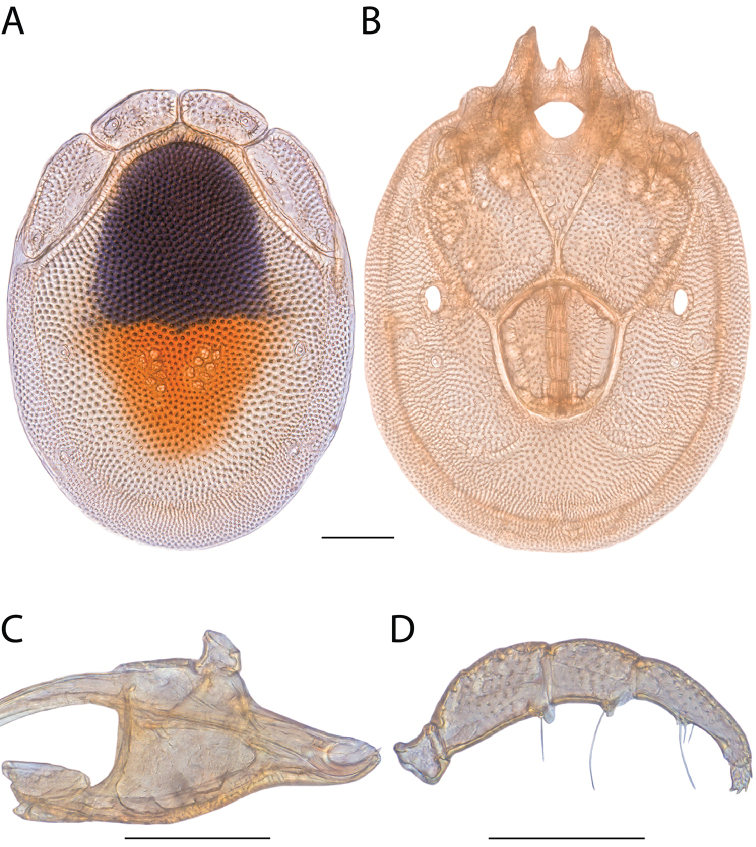
*Torrenticola unimaculata* sp. n. female: **A** dorsal plates **B** venter (legs removed) **C** subcapitulum **D** pedipalp (setae not accurately depicted). Scale = 100 µm.

**Figure 266.**
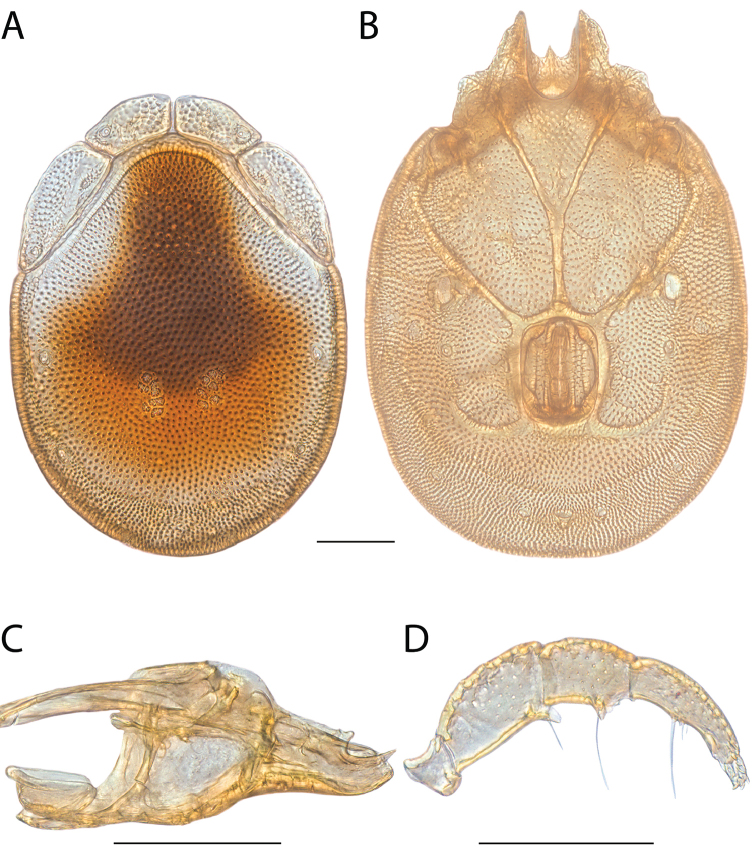
*Torrenticola unimaculata* sp. n. male: **A** dorsal plates **B** venter (legs removed) **C** subcapitulum **D** pedipalp (setae not accurately depicted). Scale = 100 µm.

######## Remarks.

*Torrenticola unimaculata* groups with other members of the Tricolor Complex with high support and specimens of this species are less than 2% different in COI sequence from each other. In all analyses, *T. unimaculata* groups with two other species which also have dorsal spots: *T. trimaculata* and *T. tricolor*. This clade represents some of the most distinctive of all *Torrenticola*. This species is greater than 7% different in COI from sister species.

This species hypothesis is supported by high divergence between species (3–15%), and by the morphological characters outlined in the diagnosis.

####### Torrenticola ventura

Taxon classificationAnimaliaTrombidiformesTorrenticolidae

Habeeb, 1973

Torrenticola ventura Habeeb, 1973: 1.

######## Material examined.

LECTOTYPE (1 ♀): from USA, California, Ventura County, Upper Ojai, Sisar Canyon, 4 Nov 1973, by H Habeeb, HH730009.

PARALECTOTYPES (1 ♀; 2 ♂): California, USA: 1 ♂ (ALLOTYPE) from Ventura County, Upper Ojai, Sisar Canyon, 4 Nov 1973, by H Habeeb, HH730009 • 1 ♀ and 1 ♂ from Ventura County, Upper Ojai, Sisar Canyon, 4 Nov 1973, by H Habeeb, HH730009.

OTHER MATERIAL (30 ♀; 37 ♂): **California, USA**: 1 ♀ and 1 ♂ from Alpine County, Markleeville Creek (38°41'39"N, 119°46'41"W), 30 Aug 2013, by JR Fisher, JRF 13-0830-001 • 3 ♀ and 3 ♂ from Humboldt County, Honeydew, Mattole River, beside road to Bull Creek on east side of bridge, 8 Aug 1987, by IM Smith, IMS870135A • 1 ♀ and 1 ♂ from Los Angeles County, Azusa, San Gabriel Canyon, East Fork of San Gabriel River at East Fork Station, 24 Jul 1987, by IM Smith, IMS870108 • 1 ♀ and 1 ♂ from Mariposa County, El Portal, Indian Flat campground, Merced River, 9-10 Jun 1976, by IM Smith, IMS760087 • 2 ♀ and 3 ♂ from Mendocino County, Paul M. Dimmick Recreation Area, North Fork of Navarro River, beside Route 128, 4 Aug 1987, by IM Smith, IMS870127A • 3 ♀ and 3 ♂ from Mendocino County, Rancheria Creek, beside Route 128, 7.3 kilometers south of Boonville, 4 Aug 1987, by IM Smith, IMS870126A • 2 ♀ and 2 ♂ from Monterey County, Big Sur River, beside Route 1 near Pfeiffer-Big Sur State Park, 28-29 Jul 1987, by IM Smith, IMS870116A • 1 ♀ from Plumas County, Plumas National Forest, Silver Creek (39°56'60"N, 121°2'17"W), 24 Aug 2013, by JR Fisher, JRF 13-0824-005 • 2 ♀ and 4 ♂ from Trinity County, Shasta-Trinity National Forest, Wilson Creek (40°25'17"N, 123°3'5"W), 20 Aug 2013, by JR Fisher, JRF 13-0820-003 • 1 ♂ from Trinity County, small cascading trickle beside Route 36, 5.2 kilometers west of Forest Glen Station, 6 Aug 1987, by IM Smith, IMS870132 • 2 ♀ and 3 ♂ from Trinity County, South Fork of Trinity River, beside Route 36 at Forest Glen campground, 6 Aug 1987, by IM Smith, IMS870131 • 2 ♀ and 4 ♂ from Trinity County, Trinity River, beside Route 299, 8.7 kilometers northwest of Del Loma, 9 Aug 1987, by IM Smith, IMS870137A • 1 ♀ and 1 ♂ from Trinity County, Weaver Creek, beside Route 299, 4.3 kilometers north of Route 3 West, 9 Aug 1987, by IM Smith, IMS870138A & IMS870138B • 1 ♀ and 4 ♂ from Ventura County, Ojai, North Fork of Ventura River, beside Route 33 just above Wheeler Gorge, 25-26 Jul 1987, by IM Smith, IMS870109A & IMS870109B • 1 ♀ and 1 ♂ from Ventura County, Ojai, North Fork of Ventura River, beside Route 33, 9.8 kilometers north of Ojai, 25 Jul 1987, by IM Smith, IMS870110 • 1 ♀ and 1 ♂ from Ventura County, Ojai, North Fork of Ventura River just below Wheeler Gorge campground, beside Route 33, 27 Jul 1987, by IM Smith, IMS870112 • 1 ♀ and 1 ♂ from Ventura County, Sespe Creek at Middle Lion campground, off Road 6N31, 26 Jul 1987, by IM Smith, IMS870111A • 1 ♀ from Yuba County, Tahoe National Forest, Oregon Creek (39°23'50"N, 121°4'54"W), 25 Aug 2013, by JR Fisher, JRF 13-0825-006 • **Oregon, USA**: 1 ♀ and 1 ♂ from Coos County, Gaylord, Coquille Myrtle Grove State Park, Coquille River, 2 Jul 1983, by IM Smith, IMS830014 • 1 ♀ from Curry County, Quosatana Creek (42°29'21"N, 124°14'2"W), 14 Aug 2013, JR Fisher, JRF 13-0814-003 • 2 ♀ and 2 ♂ from Curry County, Sixes, Sixes River, beside road at mouth of Edson Creek, 4 Jul 1983, by IM Smith, IMS830021A.

######## Type deposition.

Types (2 ♀; 2 ♂) deposited in the CNC.

######## Diagnosis.

*Torrenticola ventura* is unlike all other western species by having the following combination of characters: anterio-lateral platelets free (fused to dorsal plate in Rusetria Complex); dorsal coloration separated into anterior and posterior portions (restricted posteriorly in *T. tahoei*, *T. oregonensis*, *T. raptoroides*, and *T. sharkeyi*, without pattern in *T. regalis*, colorless in *T. occidentalis*, Rala Group, *T. wiedenmanni*); ellipsoid body (circular in *T. sierrensis* and *T. raptoroides*, rectangular in *T. ellipsoidalis*); unmodified rostrum (short and conical in *T. olliei*, *T. sierrensis*, *T. ellipsoidalis* and *T. leviathan*); tuberculate ventral extensions on pedipalp genua (flanged in Miniforma group); and smaller body size than *T. multiforma* (dorsum length ♀ = 650–780 in *T. ventura*, 765–885 in *T. multiforma*; ♂ = 540–630 in *T. ventura*, 725–850 in *T. multiforma*). *T. ventura* are most similar to members of the Neoanomala Group, which are eastern.

######## Re-description.

**Female (Figure 268)** (n = 7) with characters of the genus with following specifications.

**Dorsum** — (650–780 long; 470–550 wide) ovoid with bluish-purple coloration (often faint) separated into anterior and posterior portions with orange medially. Anterio-medial platelets (145–152.5 long; 57.5–62.5 wide). Anterio-lateral platelets (195–227.5 long; 67.5–80 wide) free from dorsal plate. Dgl-4 much closer to the edge of the dorsum than to the muscle scars (distance between Dgl-4 355–400). Dorsal plate proportions: dorsum length/width 1.34–1.46; dorsal width/distance between Dgl-4 1.29–1.42; anterio-medial platelet length/width 2.32–2.65; anterio-lateral platelet length/width 2.70–3.03; anterio-lateral/anterio-medial length 1.28–1.57.

**Gnathosoma — Subcapitulum** (350–397.5 long (ventral); 267–297 long (dorsal); 147.5–167.5 tall) colorless. Rostrum (137.5–155 long; 50–60 wide). Chelicerae (375–415 long) with curved fangs (63–80 long). Subcapitular proportions: ventral length/height 2.30–2.47; rostrum length/width 2.58–2.95. **Pedipalps** with tuberculate ventral extensions on femora and genua. Palpomeres: trochanter (47.5–60 long); femur (125–143.75 long); genu (75–82.5 long); tibia (92.5–102.5 long; 25–28.75 wide); tarsus (17.5–25 long). Palpomere proportions: femur/genu 1.67–1.80; tibia/femur 0.71–0.75; tibia length/width 3.36–3.73.

**Venter** — (740–910 long; 550–667 wide) often with faint bluish-purple coloration. Gnathosomal bay (175–202.5 long; 87.5–122.5 wide). Cxgl-4 subapical. **Medial suture** (22.5–30 long). **Genital plates** (170–200 long; 167.5–182.5 wide). Additional measurements: Cx-1 (322–365 long (total); 137–173 long (medial)); Cx-3 (22.5–30 wide); anterior venter 197.5–220 long). Ventral proportions: gnathosomal bay length/width 1.49–2.25; anterior venter/genital field length 1.05–1.16; anterior venter length/genital field width 1.08–1.22; anterior venter/medial suture 7.18–8.78.

**Male (Figure 269)** (n = 7) (allotypic measurements in parentheses when available) with characters of the genus with following specifications.

**Dorsum** — (540–630 long; 370–430 wide) ovoid with bluish-purple coloration (often faint) separated into anterior and posterior portions with orange medially. Anterio-medial platelets (105–135 long; 47.5–55 wide). Anterio-lateral platelets (172.5–197.5 long; 60–62.5 wide) free from dorsal plate. Dgl-4 much closer to the edge of the dorsum than to the muscle scars (distance between Dgl-4 280–315). Dorsal plate proportions: dorsum length/width 1.43–1.53; dorsal width/distance between Dgl-4 1.30–1.38; anterio-medial platelet length/width 2.21–2.63; anterio-lateral platelet length/width 2.76–3.16; anterio-lateral/anterio-medial length 1.40–1.64.

**Gnathosoma — Subcapitulum** (300–332.5 long (ventral); 221.05–247.5 long (dorsal); 106.25–122.5 tall) colorless. Rostrum (120–135 long; 42.5–47.5 wide). Chelicerae (289–340 long) with curved fangs (56–65 long). Subcapitular proportions: ventral length/height 2.65–2.93; rostrum length/width 2.67–3.06. **Pedipalps** with tuberculate ventral extensions on femora and genua. Palpomeres: trochanter (40–45 long); femur (106.25–117.5 long); genu (65–72.5 long); tibia (81.25–91.25 long; 23.75–27.5 wide); tarsus (15–21.25 long). Palpomere proportions: femur/genu 1.62–1.70; tibia/femur 0.74–0.81; tibia length/width 3.32–3.42.

**Venter** — (650–785 long; 436–500 wide) often with faint bluish-purple coloration. Gnathosomal bay (122.5–162.5 long; 78.75–85 wide). Cxgl-4 subapical. **Medial suture** (97.5–125 long). **Genital plates** (122.5–142.5 long; 100–112.5 wide). Additional measurements: Cx-1 (263.5–325 long (total); 155–164 long (medial)); Cx-3 (319–357.5 wide); anterior venter (265–305 long). Ventral proportions: gnathosomal bay length/width 1.53–1.97; anterior venter/genital field length 2.02–2.25; anterior venter length/genital field width 2.55–2.81; anterior venter/medial suture 2.36–2.72.

**Immatures** unknown.

######## Etymology.

Although Habeeb (1973) did not specify an etymology for the specific epithet (*ventura*), it surely refers to the type locality—Ventura River in Ventura County, California.

######## Distribution.

**Figure 267.**
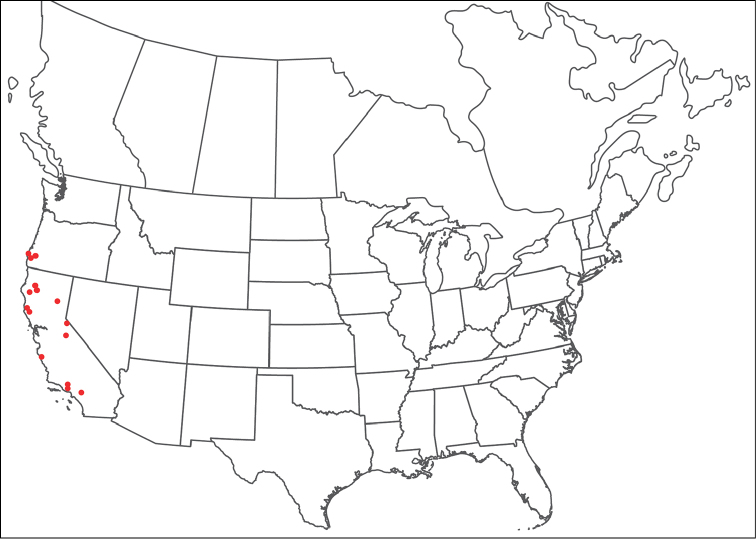
*Torrenticola ventura* distribution.

California and southwest Oregon (Figure 267). *T. ventura* was previously known only from Ventura County in southwestern California; we extend the range northward.

**Figure 268.**
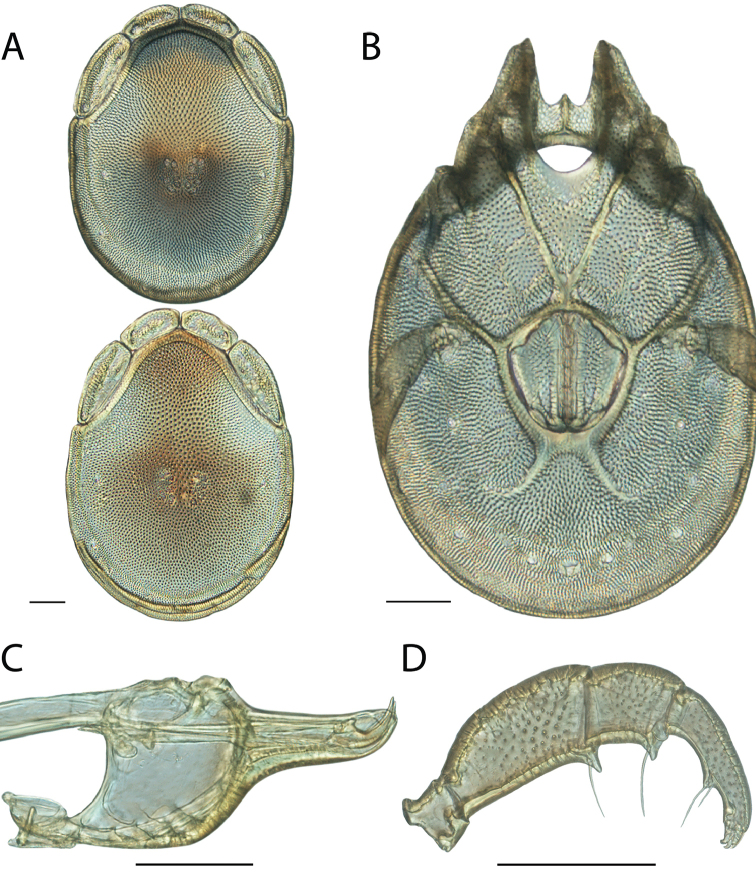
*Torrenticola ventura* female: **A** dorsal plates, note color variation **B** venter (legs removed) **C** subcapitulum **D** pedipalp (setae not accurately depicted). Scale = 100 µm.

**Figure 269.**
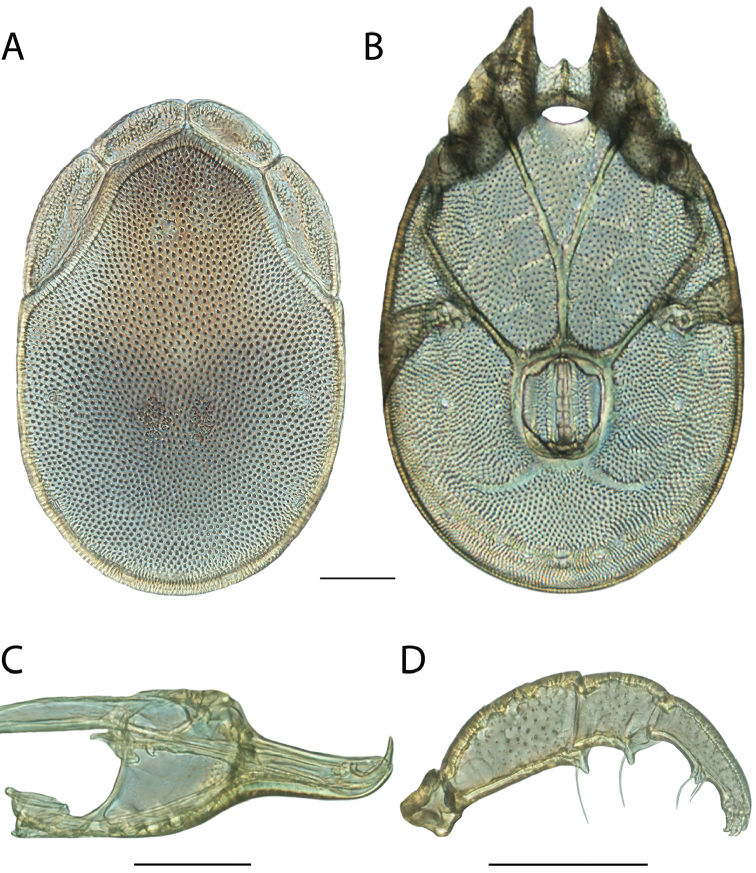
*Torrenticola ventura* male: **A** dorsal plates **B** venter (legs removed) **C** subcapitulum **D** pedipalp (setae not accurately depicted). Scale = 100 µm.

######## Remarks.

Our analyses were unable to confidently place *Torrenticola ventura* phylogenetically. Both analyses place this species at the base of the Raptor Complex, but this relationship was not well-supported. Because of this ambiguity, we refrain from placing this species in a species complex. Furthermore, because of the unique morphology, we are also unable to place this species within an identification group.

All specimens are less than 1% different in COI sequence and are greater than 15% different from sister species. This species hypothesis is supported by biogeography, low COI divergence within the species (0–2%) and high divergence between species (3–15%), and by the morphological characters outlined in the diagnosis.

####### Torrenticola walteri

Taxon classificationAnimaliaTrombidiformesTorrenticolidae

Fisher & Dowling sp. n.

http://zoobank.org/47C0C26D-96DA-4C6F-B336-CD902D03DDB3

######## Material examined.

HOLOTYPE (♀): from Canada, British Columbia, Ryan Rest Area off Hwy 3, East of Yahk Moyie River, 15 Aug 2012, by IM Smith, IMS120071, DNA 2955.

PARATYPES (12 ♀; 4 ♂): **California, USA**: 1 ♀ and 1 ♂ from El Dorado County, Upper Truckee River (38°50'56"N, 120°1'39"W), 29 Aug 2013, by JR Fisher, JRF 13-0829-003 • 2 ♀ and 2 ♂ El Dorado County, Upper Truckee River (38°50'56"N, 120°1'39"W), 29 Aug 2013, by JR Fisher, JRF 13-0829-004 • 2 ♀ from Mono County, Humboldt-Toiyabe National Forest, West Walker River (38°21'59"N, 119°28'55"W), 31 Aug 2013, by JR Fisher, JRF 13-0831-003 • 2 ♀ from Nevada County, Tahoe National Forest, Sagehen Creek (39°26'2"N, 120°12'17"W), 26 Aug 2013, by JR Fisher, JRF 13-0826-006 • **Oregon, USA**: 1 ♂ (ALLOTYPE) from Douglas County, Umpqua NF, Umpqua River (43°17'28"N, 122°37'12"W), 12 Aug 2013, by JC O’Neill, & WA Nelson, JNOW 13-0812-006 • 1 ♀ from Coos County, Middle Fork of Coquille River (43°1'56"N, 124°6'1"W), 12 Aug 2013, by JR Fisher, JRF 13-0812-001 • 2 ♀ from Douglas County, Umpqua NF, Umpqua River (43°17'28"N, 122°37'12"W), 12 Aug 2013, by JC O’Neill, & WA Nelson, JNOW 13-0812-006 • 2 ♀ from Lane County, Gate Creek (44°8'48"N, 122°34'20"W), 11 Aug 2013, by JC O’Neill, & WA Nelson, JNOW 13-0811-001.

######## Type deposition.

Holotype (♀), allotype (♂), and other paratypes (8 ♀; 2 ♂) deposited in the CNC; other paratypes (4 ♀; 1 ♂) deposited in ACUA.

######## Diagnosis.

*Torrenticola walteri* are similar to other members of the Rusetria “Western 2-Plates” group (*T. mulleni*, *T. nortoni*, and *T. welbourni*) in having anterio-lateral platelets fused to the dorsal plate, having faint dorsal coloration, and being distributed in the west. Female *T. walteri* can be differentiated from *T. welbourni* (female only known) by having stockier pedipalp tibiae (3.09–3.23 in *A32*, 3.73 in *A30*); shorter pedipalp femora (112.5–125 in *A32*, 137.5 in *A30*); and a more elongate subcapitulum (ventral length/height: 2.21–2.34 in *A32*, 2.47 in *A30*). *T. walteri* can be differentiated from *T. mulleni* by having a slightly stockier gnathosomal bay (♀ = 1.57–1.84 in *T. walteri*, 1.89–2.16 in *T. mulleni*, ♂ = 1.55–1.73 in *T. walteri*, 1.77–1.93 in *T. mulleni*) and by being distributed in California, Oregon and British Columbia, instead of in the Rocky Mountains (Idaho, Montana, Utah and Wyoming). Additionally, male *T. walteri* can be differentiated from male *T. mulleni* by having a shorter genital field (115–117.5 in *A32*, 130–140 in *A31*), and female *T. walteri* can be differentiated from female *T. mulleni* by having a shorter medial suture (10–12.5 in *T. walteri*, 20—22.5 in *T. mulleni*). Female *T. walteri* can be differentiated from female *T. nortoni* by having slightly shorter pedipalp femora with respect to genua (1.52–1.64 in *T. walteri*, 1.69–1.82 in *T. nortoni*) and slightly stockier anterio-medial platelets (2.58–2.72 in *T. walteri*, 2.74–3.06 in *T. nortoni*). Male *T. walteri* can be differentiated from male *T. nortoni* by having longer pedipalp femora (95–100 in *T. walteri*, 85–92.5 in *T. nortoni*) and slightly more elongate pedipalp tibiae (3.05–3.10 in *T. walteri*, 2.73–3.0 in *T. nortoni*).

######## Description.

**Female (Figure 271)** (n = 5) (holotype measurements in parentheses when available) with characters of the genus with following specifications.

**Dorsum** — (580–640 (590) long; 420–450 (420) wide) ovoid with orange coloration separated into anterior and posterior portions, occasionally faint. Anterio-medial platelets (120–137.5 (120) long; 45–51.25 (45) wide). Anterio-lateral platelets (157.5–190 (157.5) long; 55–65 (55) wide) fused with dorsal plate. Dgl-4 much closer to the edge of the dorsum than to the muscle scars (distance between Dgl-4 300–325 (300)). Dorsal plate proportions: dorsum length/width 1.35–1.44 (1.40); dorsal width/distance between Dgl-4 1.37–1.45 (1.40); anterio-medial platelet length/width 2.58–2.72 (2.67); anterio-lateral platelet length/width 2.63–3.27 (2.86); anterio-lateral/anterio-medial length 1.29–1.46 (1.31).

**Gnathosoma — Subcapitulum** (305–335 (317.5) long (ventral); 223–243 (224) long (dorsal); 135–147.5 (137.5) tall) colorless. Rostrum (122.5–127.5 (123.75) long; 45–50 (47.5) wide). Chelicerae (304–328 (304) long) with curved fangs (55.75–64 (63) long). Subcapitular proportions: ventral length/height 2.21–2.34 (2.31); rostrum length/width 2.45–2.72 (2.61). **Pedipalps** with tuberculate ventral extensions on femora and genua. Palpomeres: trochanter (47.5–52.5 (50) long); femur (112.5–125 (115) long); genu (70–82.5 (70) long); tibia (85–88.75 (88.75) long; 27.5–27.5 (27.5) wide); tarsus (17.5–20 (20) long). Palpomere proportions: femur/genu 1.52–1.64 (1.64); tibia/femur 0.70–0.77 (0.77); tibia length/width 3.09–3.23 (3.23).

**Venter** — (690–730 (725) long; 467–520 (467) wide) colorless. Gnathosomal bay (141.25–175 (157.5) long; 86.25–95 (86.25) wide). Cxgl-4 subapical. **Medial suture** (10–12.5 (10) long). **Genital plates** (185–197.5 (197.5) long; 158.75–181.25 (180) wide). Additional measurements: Cx-1 (250–300 (285) long (total); 102–127 (119) long (medial)); Cx-3 (313–362 (313) wide); anterior venter (140–158.75 (150) long). Ventral proportions: gnathosomal bay length/width 1.57–1.84 (1.83); anterior venter/genital field length 0.75–0.86 (0.76); anterior venter length/genital field width 0.83–0.96 (0.83); anterior venter/medial suture 11.20–15.88 (15.00).

**Male (Figure 272)** (n = 4) (allotypic measurements in parentheses when available) with characters of the genus with following specifications.

**Dorsum** — (490–510 (510) long; 340–350 (350) wide) ovoid with orange coloration separated into anterior and posterior portions, occasionally faint. Anterio-medial platelets (105–115 (115) long; 40–42.5 (42.5) wide). Anterio-lateral platelets (125–142.5 (142.5) long; 47.5–52.5 (52.5) wide) fused with dorsal plate. Dgl-4 much closer to the edge of the dorsum than to the muscle scars (distance between Dgl-4 255–270 (270)). Dorsal plate proportions: dorsum length/width 1.43–1.46 (1.46); dorsal width/distance between Dgl-4 1.30–1.33 (1.30); anterio-medial platelet length/width 2.63–2.71 (2.71); anterio-lateral platelet length/width 2.63–2.71 (2.71); anterio-lateral/anterio-medial length 1.19–1.36 (1.24).

**Gnathosoma — Subcapitulum** (265–270 (270) long (ventral); 179–202.5 (195) long (dorsal); 93.75–105 (95) tall) colorless. Rostrum (100–102.5 (102.5) long; 37.5–38.75 (37.5) wide). Chelicerae (240–265 (265) long) with curved fangs (38–50 (50) long). Subcapitular proportions: ventral length/height 2.52–2.85 (2.84); rostrum length/width 2.65–2.73 (2.73). **Pedipalps** with tuberculate ventral extensions on femora and genua. Palpomeres: trochanter (35–40 (35) long); femur (95–100 (100) long); genu (57.5–60 (57.5) long); tibia (72.5–77.5 (72.5) long; 23.75–25 (23.75) wide); tarsus (17.5–20 (17.5) long). Palpomere proportions: femur/genu 1.65–1.74 (1.74); tibia/femur 0.73–0.82 (0.73); tibia length/width 3.05–3.10 (3.05).

**Venter** — (590–625 (625) long; 387.5–410 (410) wide) colorless. Gnathosomal bay (112.5–127.5 (127.5) long; 72.5–78.75 (73.75) wide). Cxgl-4 subapical. **Medial suture** (85–107.5 (107.5) long). **Genital plates** (115–117.5 (116.25) long; 95–98.75 (95) wide). Additional measurements: Cx-1 (255–273 (270) long (total); 136–157 (150) long (medial)); Cx-3 (253–316 (285) wide); anterior venter (245–262.5 (262.5) long). Ventral proportions: gnathosomal bay length/width 1.55–1.73 (1.73); anterior venter/genital field length 2.12–2.26 (2.26); anterior venter length/genital field width 2.48–2.76 (2.76); anterior venter/medial suture 2.44–2.88 (2.44).

**Immatures** unknown.

######## Etymology.

Specific epithet (*walteri*) named in honor of acarologist Dave Walter for solidifying JRF’s interest in mites with his popular book on mites (*Mites: Ecology*, *Evolution & Behaviour – Life at a Microscale*) and by teaching JRF the mesostigmatan mite section of the Acarology Summer Program at The Ohio State University in 2009.

######## Distribution.

**Figure 270.**
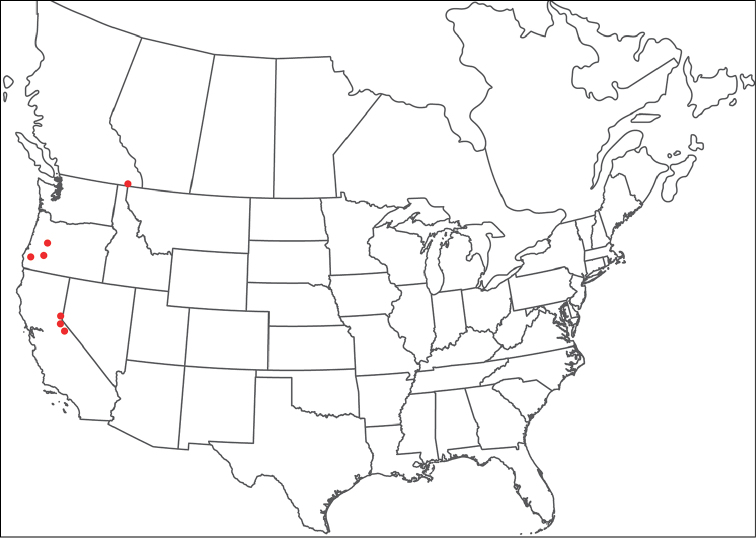
*Torrenticola walteri* sp. n. distribution.

Probably throughout the Pacific Coastal Ranges of California, Oregon, Washington, and southern British Columbia (Figure 270). We also collected *T. walteri* in the Rocky Mountains of British Columbia, indicating this species might occur in the northern Rockies of Canada. However, given our sampling effort in the Rockies, we doubt the occurrence of this species in most of the US Rockies.

**Figure 271.**
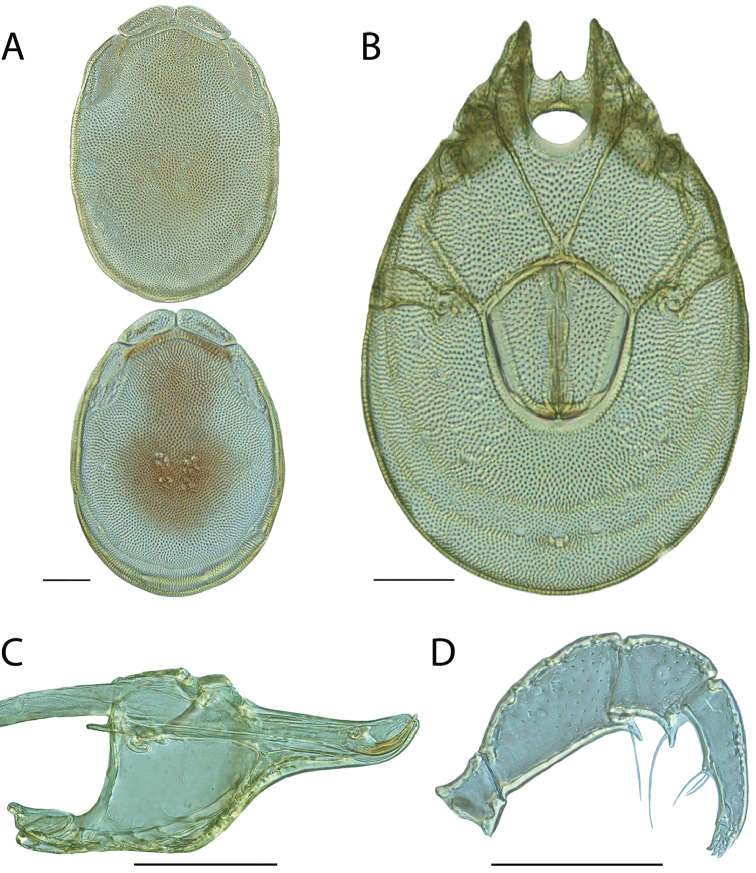
*Torrenticola walteri* sp. n. female: **A** dorsal plates, note color variation **B** venter (legs removed) **C** subcapitulum **D** pedipalp (setae not accurately depicted). Scale = 100 µm.

**Figure 272.**
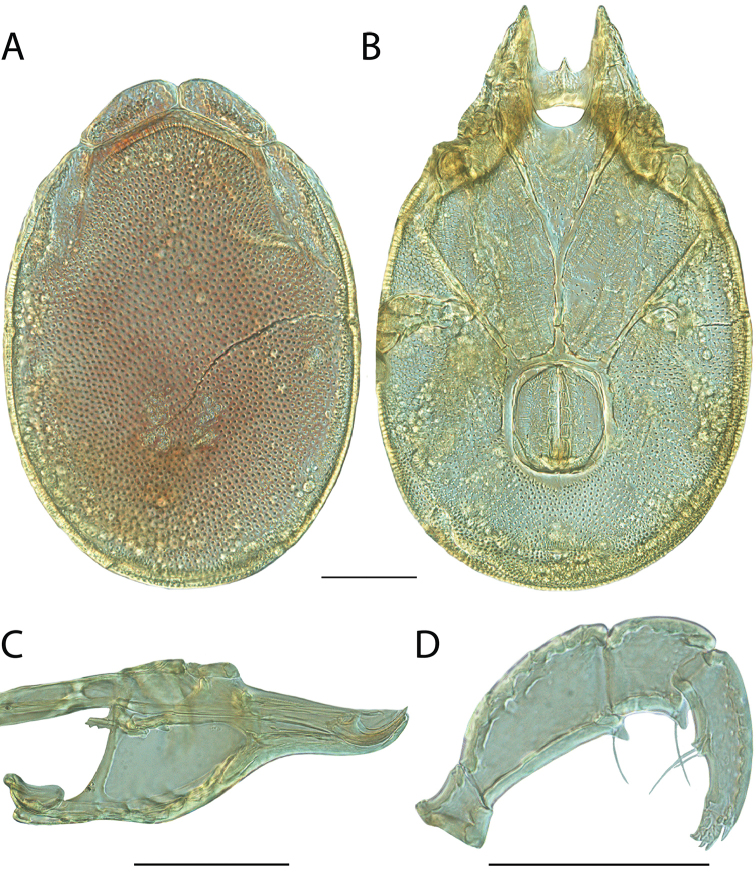
*Torrenticola walteri* sp. n. male: **A** dorsal plates **B** venter (legs removed) **C** subcapitulum **D** pedipalp (setae not accurately depicted). Scale = 100 µm.

######## Remarks.

*Torrenticola walteri* groups with other members of the Rusetria Complex with high support and specimens are less than 2% different in COI sequence from each other. In the all analyses, *T. walteri* groups with the three other members of the Rusetria Complex that are found in western North America: *T. mulleni*, *T. nortoni*, and *T. welbourni*. These species are 5–7% different in COI sequence from each other and together make up the Western 2-Plate Identification Group. *Torrenticola walteri* is one of three of these that occur in California (including *T. nortoni* and *T. welbourni*).

This species hypothesis is supported by low COI divergence within the species (0–2%) and high divergence between species (3–15%), and by the morphological characters outlined in the diagnosis.

####### Torrenticola welbourni

Taxon classificationAnimaliaTrombidiformesTorrenticolidae

Fisher & Dowling sp. n.

http://zoobank.org/084524AE-73BB-4940-BFD2-53D9DEB897B1

######## Material examined.

HOLOTYPE (♀): from USA, California, Trinity County, Shasta-Trinity National Forest, Wilson Creek (40°25'17"N, 123°3'5"W), 20 Aug 2013, by JR Fisher, JRF 13-0820-003, DNA 1638.

######## Type deposition.

Holotype (♀) deposited in the CNC.

######## Diagnosis.

*Torrenticola welbourni* are similar to other members of the Rusetria “Western 2-Plates” group (*T. mulleni*, *T. nortoni*, and *T. walteri*) in having anterio-lateral platelets fused to the dorsal plate, having faint dorsal coloration, and being distributed in the west. *T. welbourni* (only female known) can be differentiated from all other Western 2-plates by being larger (dorsal length: 690 in *T. welbourni*, 570–645 in others; dorsal width: 500 in *T. welbourni*, 415–480 in others), having more elongate pedipalp tibiae (length/width: 3.73 in *T. welbourni*, 3.0–3.33 in others) and longer pedipalp femora (137.5 in *A30*, 112.5–125 in others).

######## Description.

**Female (Figure 274)** (n = 1) (holotype only) with characters of the genus with following specifications.

**Dorsum** — (690 long; 500 wide) ovoid with faint orange coloration separated into anterior and posterior portions. Anterio-medial platelets (152.5 long; 57.5 wide). Anterio-lateral platelets (195 long; 72.5 wide) fused to dorsal plate. Dgl-4 closer to the edge of the dorsum than to the muscle scars (distance between Dgl-4/dorsal width 325). Dorsal plate proportions: dorsum length/width 1.38; dorsum width/distance between Dgl-4 1.54; anterio-medial platelet length/width 2.65; anterio-lateral platelet length/width 2.69; anterio-lateral/anterio-medial length 1.28.

**Gnathosoma — Subcapitulum** (357.5 long (ventral); 270 long (dorsal); 145 tall) colorless. Rostrum (145 long; 52.5 wide). Chelicerae (356 long) with curved fangs (70 long). Subcapitular proportions: ventral length/height 2.47; rostrum length/width 2.76. **Pedipalps** with tuberculate ventral extensions on femora and genua. Palpomeres: trochanter (50 long); femur (137.5 long); genu (75 long); tibia (102.5 long; 27.5 wide); tarsus (20 long). Palpomere proportions: femur/genu 1.83; tibia/femur 0.75; tibia length/width 3.73.

**Venter** — (820 long; 580.25 wide) colorless. Gnathosomal bay (180 long; 100 wide). Cxgl-4 subapical. **Medial suture** (12.5 long). **Genital plates** (180 long; 167.5 wide). Additional measurements: Cx-1 (324 long (total); 122 long (medial)); Cx-3 (371 wide); anterior venter (177.5 long). Ventral proportions: gnathosomal bay length/width 1.80; anterior venter/genital field length 0.99; anterior venter length/genital field width 1.06; anterior venter/medial suture 14.20.

**Male** unknown.

**Immatures** unknown.

######## Etymology.

Specific epithet (*welbourni*) named in honor of acarologist Cal Welbourn, who has been instrumental in teaching terrestrial Parasitengona to JRF and for teaching JRF the prostigmatan mite section of the Acarology Summer Program at The Ohio State University in 2009.

######## Distribution.

**Figure 273.**
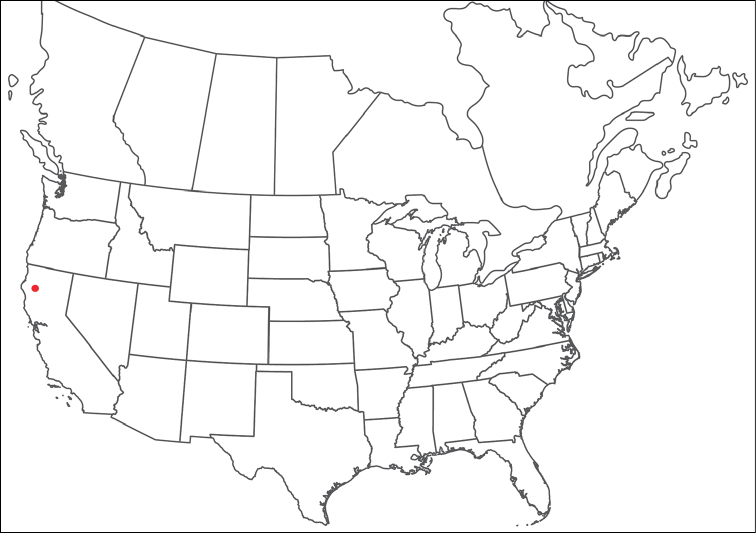
*Torrenticola welbourni* sp. n. distribution.

Only known from Trinity County, California (Figure 273).

**Figure 274.**
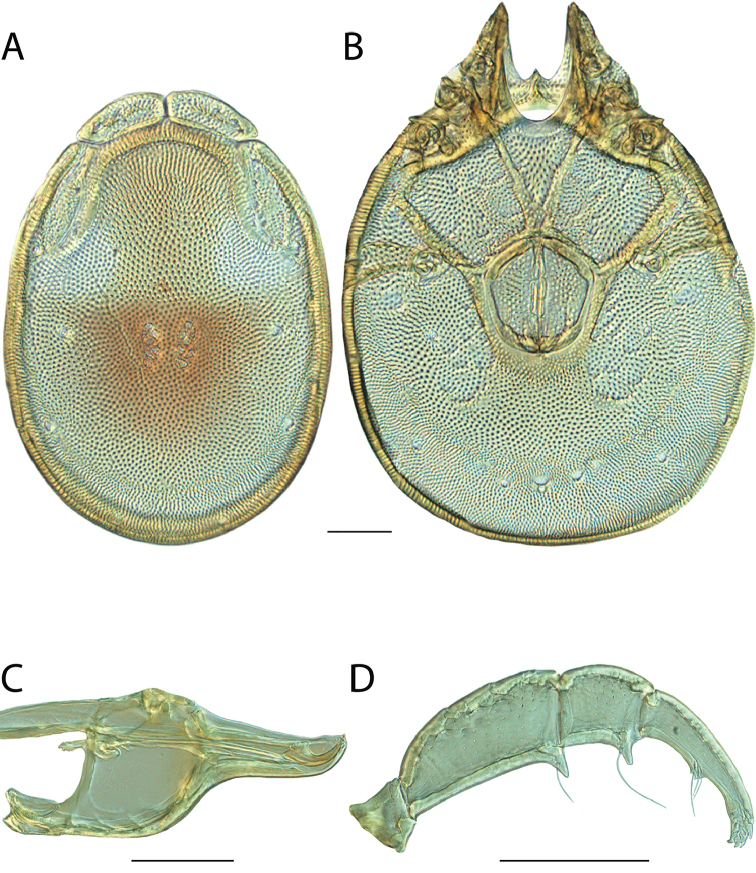
*Torrenticola welbourni* sp. n. female: **A** dorsal plates **B** venter (legs removed) **C** subcapitulum **D** pedipalp (setae not accurately depicted). Scale = 100 µm.

######## Remarks.

*Torrenticola welbourni* groups with other members of the Rusetria Complex with high support. Unfortunately, only a single specimen is known of this species, so variation in COI sequence could not be investigated. This specimen was collected from the sample that contained specimens of *T. walteri*. It is interesting to note that these two species are the only Rusetria Complex members collected from Trinity County. In the all analyses, *T. welbourni* groups with the three other members of the Rusetria Complex that are found in western North America: *T. mulleni*, *T. nortoni*, and *T. walteri*. These species are 5–7% different in COI sequence from each other and together make up the Western 2-Plate Identification Group. *Torrenticola welbourni* is one of three of these that occur in California (including *T. nortoni* and *T. walteri*).

This species hypothesis is supported by low COI divergence within the species (0–2%) and high divergence between species (3–15%), and by the morphological characters outlined in the diagnosis.

####### Torrenticola whitneyae

Taxon classificationAnimaliaTrombidiformesTorrenticolidae

Fisher & Dowling sp. n.

http://zoobank.org/EA7DE1AF-7A8D-4644-A7BB-D8601472CE09

######## Material examined.

HOLOTYPE (♀): from USA, North Carolina, Yancey County, Lost Cove Picnic Area beside Forest Road 472, 2.8 km west of Rt. 80, (35°45'45"N, 82°12'12"W), 12 September 2005, by IM Smith, IMS050074

PARATYPES (4 ♀; 3 ♂): **North Carolina, USA**: 1 ♀ and 1 ♂ from Haywood County, Great Smoky Mountains National Park; Cataloochee; beside Mt. Sterling Rd. near bridge 1.7 km n. of road to Campground, (35°38'38"N, 83°4'4"W), 6 September 2009, by IM Smith, IMS090099 • 2 ♀ from Swain County, Great Smokey Mountain National Park, Deep Creek upstream of picnic area, (35°27'27"N, 83°26'26"W), 14 September 2009, by AJ Radwell, AJR090007A • 1 ♂ (ALLOTYPE) from Yancey County, Lost Cove Picnic Area beside Forest Road 472, 2.8 km west of Rt. 80, (35°45'45"N, 82°12'12"W), 12 September 2005, by IM Smith, IMS050074 • 1 ♀ and 1 ♂ from Yancey County, Lost Cove Picnic Area beside Forest Road 472, 2.8 km west of Rt. 80, (35°45'45"N, 82°12'12"W), 12 September 2005, by IM Smith, IMS050074.

######## Type deposition.

Holotype (♀), allotype (♂), and some paratypes (2 ♀; 1 ♂) deposited in the CNC; other paratypes (2 ♀; 1 ♂) deposited in the ACUA.

######## Diagnosis.

*Torrenticola whitneyae* are similar to other members of the Rusetria “Eastern 2-Plates” group (*T. biscutella*, *T. caerulea*, *T. delicatexa*, *T. indistincta*, *T. malarkeyorum*, *T. pendula*, *T. sellersorum*, *T. tysoni*, *T. ululata*, *T. microbiscutella*, and *T. feminellai*) in having anterio-lateral platelets fused to the dorsal plate, having dorsal coloration separated into anterior and posterior portions (except *T. ululata* and *T. indistincta*), and being distributed in the east. *T. whitneyae* is most similar to *T. pendula*, which also has purple coloration separated into anterior and posterior portions and often connected by a stripe medially. *T. whitneyae* is best differentiated from *T. pendula* by having a stockier rostrum (length/width = 2.41–2.69 in *T. whitneyae*, 2.87–3.06 in *T. pendula*). *T. whitneyae* can be differentiated from *T. ululata*, *T. indistincta*, and *T. feminellai* by dorsal coloration and pattern. *T. whitneyae* can be differentiated from all other Eastern 2-Plates by having stockier pedipalpal tibiae (♀ = 2.42–2.95 in *T. whitneyae*, 3.00–4.59 in others; ♂ = 2.48–2.70 in *T. whitneyae*, 2.78–4.25 in others), except female *T. delicatexa* (♀ = 2.92–3.61). *T. whitneyae* can be differentiated from *T. delicatexa* by having a slightly rounder dorsum (length/width ♀ = 1.26–1.38 in *T. whitneyae*, 1.38–1.44 in *T. delicatexa*; ♂ = 1.35–1.37 in *T. whitneyae*, 1.44–1.56 in *T. delicatexa*) and by dorsal coloration.

######## Description.

**Female (Figure 276)** (n = 5) (holotype measurements in parentheses when available) with characters of the genus with following specifications.

**Dorsum** — (550–690 (630) long; 400–520 (500) wide) ovoid with reddish-purple coloration both anteriorly and posteriorly connected medially. Anterio-medial platelets (120–163.75 (142.5) long; 41.25–52.5 (52.5) wide). Anterio-lateral platelets (170–220 (200)) long; 65–85 (85) wide) partially fused, at least posteriorly, to dorsal plate. Dgl-4 much closer to the edge of the dorsum than to the muscle scars (distance between Dgl-4 310–385 (370)). Dorsal plate proportions: dorsum length/width 1.26–1.38 (1.26); dorsal width/distance between Dgl-4 1.29–1.37 (1.35); anterio-medial platelet length/width 2.71–3.12 (2.71); anterio-lateral platelet length/width 2.35–2.84 (2.35); anterio-lateral/anterio-medial length 1.32–1.42 (1.40).

**Gnathosoma — Subcapitulum** (310–385 (362.5) long (ventral); 237.5–285 (270) long (dorsal); 145–185 (177.5) tall) colorless. Rostrum (125–140 (135) long; 47.5–55 (55) wide). Chelicerae (310–415 (395) long) with curved fangs (65–80 (75) long). Subcapitular proportions: ventral length/height 1.95–2.14 (2.04); rostrum length/width 2.41–2.63 (2.45). **Pedipalps** stocky with short, tuberculate ventral extensions on femora and genua. Palpomeres: trochanter (50–56.25 (55) long); femur (67.5–80 (75) long); genu (67.5–80 (75) long); tibia (72.5–80 (80) long; 26.25–30 (28.75) wide); tarsus (17.5–20 (17.5) long). Palpomere proportions: femur/genu 1.59–1.71 (1.63); tibia/femur 0.59–0.68 (0.65); tibia length/width 2.42–2.95 (2.78).

**Venter** — (630–810 (780) long; 470–630 (590) wide) with faint reddish-purple coloration. Gnathosomal bay (175–222.5 (210) long; 80–105 (105) wide). Cxgl-4 subapical. **Medial suture** absent. **Genital plates** (185–195 (187.5) long; 165–195 (180) wide). Additional measurements: Cx-1 (280–360 (350) long (total); 110–140 (135) long (medial)); Cx-3 (320–435 (405) wide); anterior venter (110–145 (140) long). Ventral proportions: gnathosomal bay length/width 2.00–2.59 (2.00); anterior venter/genital field length 0.59–0.75 (0.75); anterior venter length/genital field width 0.67–0.80 (0.78).

**Male (Figure 277)** (n = 3) (allotypic measurements in parentheses when available) with characters of the genus with following specifications.

**Dorsum** — (540–560 (540) long; 395–410 (395) wide) ovoid with reddish-purple coloration both anteriorly and posteriorly connected medially. Anterio-medial platelets (122.5–135 (135) long; 45–50 (50) wide). Anterio-lateral platelets (170–170 (170) long; 60–67.5 (60) wide) partially fused, at least posteriorly, to dorsal plate. Dgl-4 much closer to the edge of the dorsum than to the muscle scars (distance between Dgl-4 290–310 (295)). Dorsal plate proportions: dorsum length/width 1.35–1.37 (1.37); dorsal width/distance between Dgl-4 1.32–1.38 (1.34); anterio-medial platelet length/width 2.55–2.72 (2.70); anterio-lateral platelet length/width 2.52–2.83 (2.83); anterio-lateral/anterio-medial length 1.26–1.39 (1.26).

**Gnathosoma — Subcapitulum** (290–305 (300) long (ventral); 220–225 (222.5) long (dorsal); 120–135 (135) tall) colorless. Rostrum (107.5–112.5 (112.5) long; 40–42.5 (42.5) wide). Chelicerae (310–310 (310) long) with curved fangs (60–60 (60) long). Subcapitular proportions: ventral length/height 2.22–2.42 (2.22); rostrum length/width 2.65–2.69 (2.65). **Pedipalps** stocky with short, tuberculate ventral extensions on femora and genua. Palpomeres: trochanter (47.5–47.5 (47.5) long); femur (97.5–100 (97.5) long); genu (62.5–62.5 (62.5) long); tibia (65–67.5 (67.5) long; 25–26.25 (25) wide); tarsus (16.25–17.5 (16.25) long). Palpomere proportions: femur/genu 1.56–1.60 (1.56); tibia/femur 0.65–0.69 (0.69); tibia length/width 2.48–2.70 (2.70).

**Venter** — (640–680 (640) long; 440–495 (495) wide) with faint reddish-purple coloration. Gnathosomal bay (170–175 (170) long; 70–75 (70) wide). Cxgl-4 subapical. **Medial suture** (55–75 (55) long). **Genital plates** (140–150 (140) long; 130–140 (130) wide). Additional measurements: Cx-1 (290–300 (300) long (total); 110–135 (135) long (medial)); Cx-3 (325–360 (360) wide); anterior venter (195–210 (200) long). Ventral proportions: gnathosomal bay length/width 2.33–2.50 (2.43); anterior venter/genital field length 1.37–1.43 (1.43); anterior venter length/genital field width 1.50–1.54 (1.54); anterior venter/medial suture 2.60–3.64 (3.64).

**Immatures** unknown.

######## Etymology.

Specific epithet (*whitneyae*) named in honor of Whitney Nelson, one of two students (including JRF) of APGD studying water mite taxonomy for their doctoral studies.

######## Distribution.

**Figure 275.**
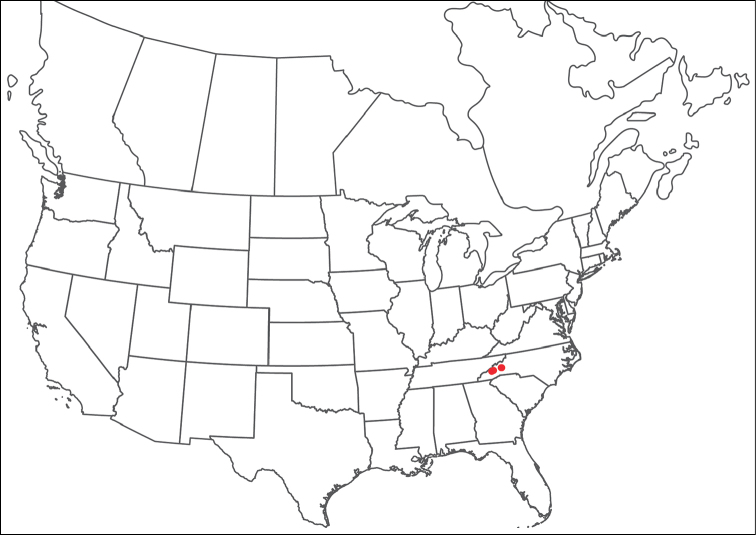
*Torrenticola whitneyae* sp. n. distribution.

Southern Appalachians (Figure 275).

**Figure 276.**
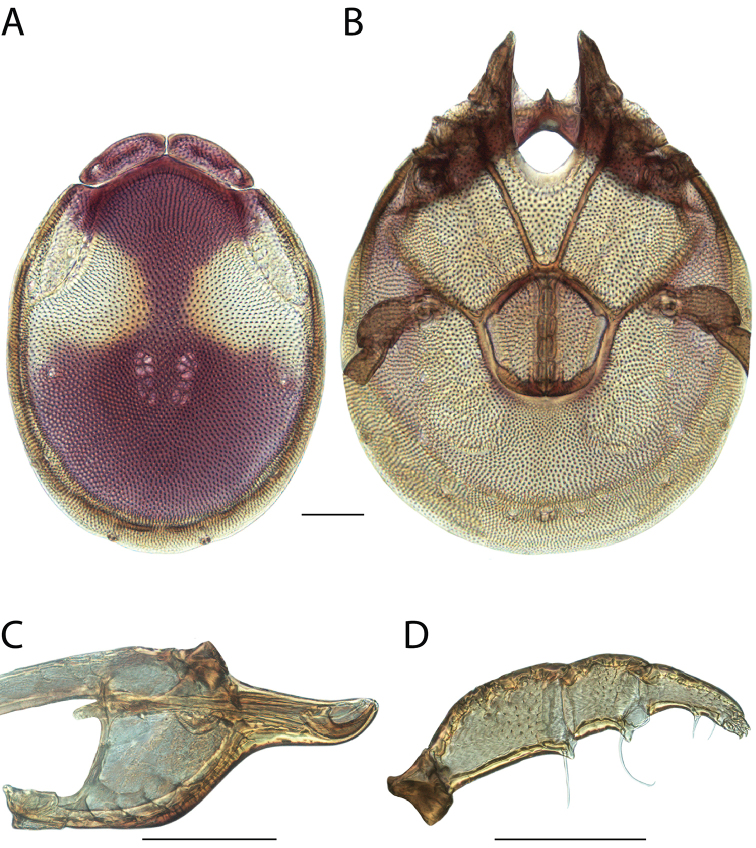
*Torrenticola whitneyae* sp. n. female: **A** dorsal plates **B** venter (legs removed) **C** subcapitulum **D** pedipalp (setae not accurately depicted). Scale = 100 µm.

**Figure 277.**
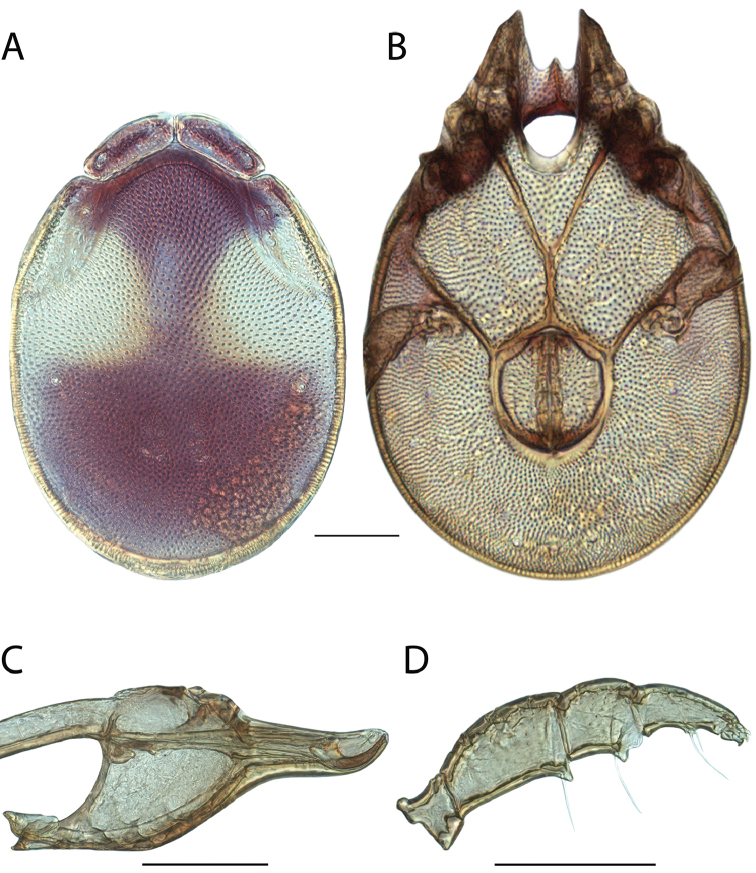
*Torrenticola whitneyae* sp. n. male: **A** dorsal plates **B** venter (legs removed) **C** subcapitulum **D** pedipalp (setae not accurately depicted). Scale = 100 µm.

######## Remarks.

Unfortunately, we were unable to acquire fresh material of *Torrenticola whitneyae* and therefore this species is not included in our phylogenetic analyses. However, we were able to examine morphology with material preserved in GAW. The overall similarity, distribution in the east, and fusion of the dorso-lateral platelets to the dorsal plate, are consistent with placing this species in the Rusetria Complex and within the Eastern 2-Plate Identification Group.

####### Torrenticola wiedenmanni

Taxon classificationAnimaliaTrombidiformesTorrenticolidae

Fisher & Dowling sp. n.

http://zoobank.org/76706F18-6CDE-4A99-93C2-76BBE372E10A

######## Material examined.

HOLOTYPE (♀): from USA, California, Monterey County, beside Rt. 1 south of Gorda, south side of Salmon Creek, (35°49'49"N, 121°22'22"W), 28 July 1987, by IM Smith, IMS870115.

PARATYPES (5 ♀; 4 ♂): California, USA: 1 ♂ (ALLOTYPE) from Monterey County, beside Rt. 1 south of Gorda, south side of Salmon Creek, (35°49'49"N, 121°22'22"W), 28 July 1987, by IM Smith, IMS870115 • 1 ♀ and 1 ♂ from San Bernardino County, Claremont; Mount Baldy; beside road 3.5 km east of Mount Baldy Village, (34°15'15"N, 117°39'39"W), 24 July 1987, by IM Smith, IMS870107 • 2 ♀ and 2 ♂ from San Bernardino County, Lytle Creek Recreation Area off Rt. 15 west of Devore; Applewhite Picnic Grounds, (34°16'16"N, 117°30'30"W), 23 July 1987, by IM Smith, IMS870103 • 1 ♀ from Tulare County, beside Rt. 180 at Stony Creek Picnic Area east of Sequoia National Park boundary, (36°40'40"N, 118°50'50"W), 1 August 1987, by IM Smith, IMS870124A • 1 ♀ from Ventura County, Ojai; beside Rt. 33 just above Wheeler Gorge Campground, (34°31'31"N, 119°16'16"W), 25 July 1987, by IM Smith, IMS870109B.

######## Type deposition.

Holotype (♀), allotype (♂), and some paratypes (3 ♀; 2 ♂) deposited in the CNC; other paratypes (2 ♀; 1 ♂) deposited in the ACUA.

######## Diagnosis.

*Torrenticola wiedenmanni* is unlike all other western species by having this combination of characters: anterio-lateral platelets free (fused to dorsal plate in Rusetria Complex); colorless (dorsum with distinct coloration in Tahoei group, *T. ventura*, and *T. raptoroides*); distinct and complete hind coxal margins (incomplete in Rala Group, and *T. sharkeyi*); round body (rectangular or ovoid in Ellipsoidalis Group); unmodified rostrum (short and conical in *T. sierrensis*, *T. olliei*, *T. leviathan*, and *T. ellipsoidalis*); and tuberculate ventral extensions on pedipalpal genua (dentate and flanged in Miniforma group).

######## Description.

**Female (Figure 279)** (n = 5) (holotype measurements in parentheses when available) with characters of the genus with following specifications.

**Dorsum** — (560–640 (610) long; 445–540 (530) wide) circular and colorless. Anterio-medial platelets (110–150 (135) long; 55–75 (67.5) wide). Anterio-lateral platelets (182.5–200 (190) long; 65–85 (80) wide) free from dorsal plate. Dgl-4 approximately halfway between the edge of the dorsum and the muscle scars and occasionally anterior to the muscle scars (distance between Dgl-4 295–350 (315)). Dorsal plate proportions: dorsum length/width 1.15–1.26 (1.15); dorsal width/distance between Dgl-4 1.33–1.73 (1.68); anterio-medial platelet length/width 2.00–2.11 (2.00); anterio-lateral platelet length/width 2.35–2.81 (2.38); anterio-lateral/anterio-medial length 1.31–1.66 (1.41).

**Gnathosoma — Subcapitulum** (302.5–355 (352.5) long (ventral); 222.5–267.5 (260) long (dorsal); 110–130 (120) tall) colorless. Rostrum (120–142.5 (142.5) long; 37.5–42.5 (40) wide). Chelicerae (310–385 (385) long) with curved fangs (47.5–50 (50) long). Subcapitular proportions: ventral length/height 2.73–2.94 (2.94); rostrum length/width 3.20–3.56 (3.56). **Pedipalps** with tuberculate ventral extensions on femora and genua, with femora sharply taper proximally and tibial setae near tarsi. Palpomeres: trochanter (30–38.75 (36.25) long); femur (90–107.5 (102.5) long); genu (65–75 (72.5) long); tibia (72.5–87.5 (87.5) long; 17.5–21.25 (21.25) wide); tarsus (13.75–17.5 (16.25) long). Palpomere proportions: femur/genu 1.37–1.43 (1.41); tibia/femur 0.79–0.85 (0.85); tibia length/width 4.00–4.27 (4.12).

**Venter** — (710–810 (780) long; 560–630 (615) wide) colorless. Gnathosomal bay (132.5–160 (160) long; 62.5–65 (62.5) wide). Cxgl-4 subapical. **Medial suture** (40–60 (40) long). **Genital plates** (172.5–187.5 (180) long; 150–165 (155) wide). Additional measurements: Cx-1 (270–320 (320) long (total); 140–162.5 (162.5) long (medial)); Cx-3 (330–385 (380) wide); anterior venter (200–235 (220) long). Ventral proportions: gnathosomal bay length/width 2.42–2.56 (2.56); anterior venter/genital field length 1.16–1.25 (1.22); anterior venter length/genital field width 1.32–1.42 (1.42); anterior venter/medial suture 3.92–5.50 (5.50).

**Male (Figure 280)** (n = 4) (allotypic measurements in parentheses when available) with characters of the genus with following specifications.

**Figure 278.**
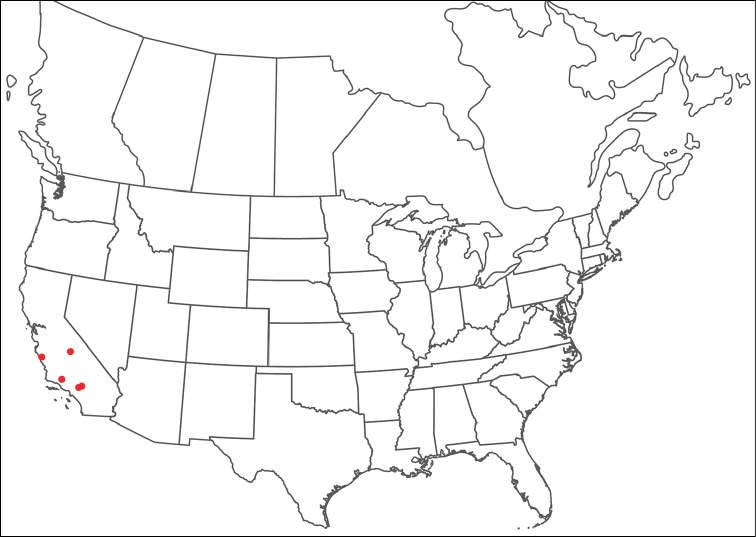
*Torrenticola wiedenmanni* distribution.

**Figure 279.**
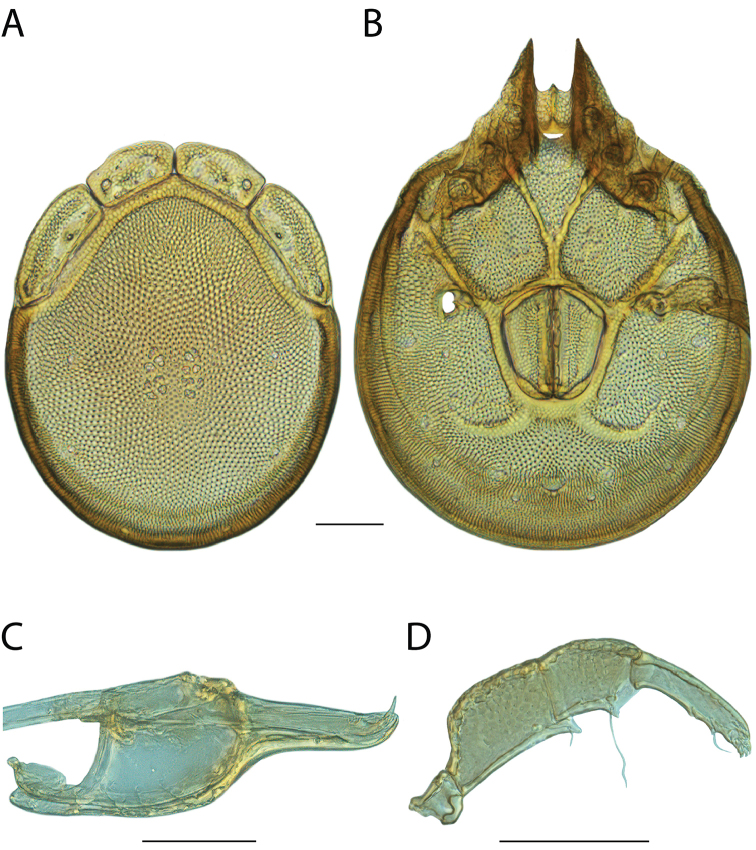
*Torrenticola wiedenmanni* sp. n. female: **A** dorsal plates **B** venter (legs removed) **C** subcapitulum **D** pedipalp (setae not accurately depicted). Scale = 100 µm.

**Figure 280.**
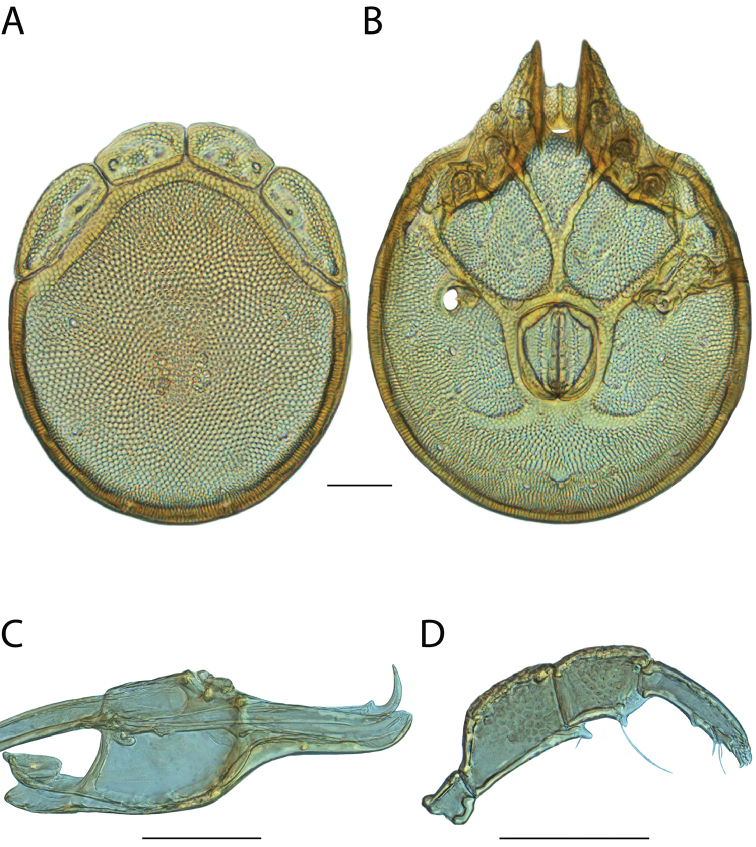
*Torrenticola wiedenmanni* sp. n. male: **A** dorsal plates **B** venter (legs removed) **C** subcapitulum **D** pedipalp (setae not accurately depicted). Scale = 100 µm.

**Dorsum** — (560–610 (575) long; 470–515 (480) wide) circular and colorless. Anterio-medial platelets (130–140 (135) long; 62.5–70 (65) wide). Anterio-lateral platelets (165–195 (180) long; 70–75 (75) wide) free from dorsal plate. Dgl-4 approximately halfway between the edge of the dorsum and the muscle scars and occasionally anterior to the muscle scars (distance between Dgl-4 285–315 (300)). Dorsal plate proportions: dorsum length/width 1.18–1.20 (1.20); dorsal width/distance between Dgl-4 1.54–1.67 (1.60); anterio-medial platelet length/width 2.00–2.08 (2.08); anterio-lateral platelet length/width 2.20–2.60 (2.40); anterio-lateral/anterio-medial length 1.27–1.39 (1.33).

**Gnathosoma — Subcapitulum** (307.5–345 (325) long (ventral); 225–257.5 (240) long (dorsal); 107.5–117.5 (107.5) tall) colorless. Rostrum (125–140 (130) long; 35–40 (37.5) wide). Chelicerae (335–357.5 (345) long) with curved fangs (45–50 (45) long). Subcapitular proportions: ventral length/height 2.80–3.02 (3.02); rostrum length/width 3.33–3.57 (3.47). **Pedipalps** with tuberculate ventral extensions on femora and genua, femora sharply taper proximally and tibial setae near tarsi. Palpomeres: trochanter (32.5–35 (32.5) long); femur (90–100 (90) long); genu (65–72.5 (65) long); tibia (75–80 (75) long; 20–20 (20) wide); tarsus (13.75–15 (15) long). Palpomere proportions: femur/genu 1.36–1.38 (1.38); tibia/femur 0.80–0.89 (0.83); tibia length/width 3.75–4.00 (3.75).

**Venter** — (690–755 (715) long; 465–590 (465) wide) colorless. Gnathosomal bay (140–152.5 (145) long; 55–60 (60) wide). Cxgl-4 subapical. **Medial suture** (70–85 (80) long). **Genital plates** (145–155 (150) long; 115–125 (117.5) wide). Additional measurements: Cx-1 (280–300 (300) long (total); 140–160 (160) long (medial)); Cx-3 (335–370 (345) wide); anterior venter (230–262.5 (260) long). Ventral proportions: gnathosomal bay length/width 2.42–2.64 (2.42); anterior venter/genital field length 1.53–1.73 (1.73); anterior venter length/genital field width 1.92–2.21 (2.21); anterior venter/medial suture 3.09–3.29 (3.25).

**Immatures** unknown.

######## Etymology.

Specific epithet (*wiedenmanni*) named in honor of Rob Wiedenmann, Professor of Entomology at University of Arkansas, whose advice, guidance, and friendship to JRF was an inspiration in how to be a true mentor—lessons that JRF anticipates paying forward to his own students someday.

######## Distribution.

Southern California (Figure 278).

######## Remarks.

Unfortunately, we were unable to acquire fresh material of *Torrenticola wiedenmanni* and therefore this species is not included in our phylogenetic analyses, but we were able to examine morphology with material preserved in GAW. However, due to its unique set of characteristics, we are unable to place this species into a species complex or an identification group.

###### Key to *Torrenticola* of the United States and Canada

Species Groups are placed within key to aid identification. However, not all groups are included and some appear multiple times (Ellipsoidalis Group; male members of the Elongata, Rala, Raptor, and Tricolor Groups).

Known only from females: *T. occidentalis*; *T. elusiva*; *T. welbourni*

Known only from males: *T. bittikoferae*; *T. longitibia*; *T. dolichodactyla*; *T. anoplopalpa*

**Table:** 

1	Female: genital field pentagonal (a); medial suture usually short (b), occasionally long (c), but never as long as conspecific male	**2**
–	Male: genital field rectangular (aa); medial suture long (bb, cc)	**89**
2 (1)	Dorso-lateral platelets entirely (a) or partially (b) fused to dorsal plate; hind coxal margin indistinct (c); subcapitulum unmodified (d)	**2-Plate Groups**...**3**
–	Dorso-lateral platelets free from dorsal plate (aa); hind coxal margin indistinct (c) or distinct (cc); subcapitulum unmodified (d) or modified (e.g., dd)	**23**
3 (2)	Medial suture present (a)*	**4**
–	Medial suture absent (aa)*	**Eastern 2-Plates (in part)**...**17**
	*T. magnexa and *T. sellersorum* key in both directions
4 (5)	Western (west of 100^th^ Meridian)* (a); dorsal coloration indistinct (b) or colorless (c)	**5**
–	Eastern (east of 100^th^ Meridian)* (aa); dorsal coloration distinct (except *T. folkertsae*) (bb)	**Eastern (in part) & Partial 2-Plates**...**9**
	*T. sellersorum keys in both directions
5 (6)	Shorter anterior venter (120–143) **and** subcapitulum ventral length/width = 2.05–2.17; dorsal pattern of eastern specimens distinctive, but specimens from New Mexico with standard pattern (i.e., without distinctive shape of posterior portion); mostly eastern, but also known from Manitoba, South Dakota, and New Mexico	*T. sellersorum* **sp. n.**
–	Longer anterior venter (145–178) (but 140–159 in *T. walteri*), **and/or** subcapitulum ventral length/width = 2.21–2.47 (but 2.14–2.4 in *T. mulleni*); western	**Western 2-Plates**...**6**
6 (5)	Dorsum larger (690 long, 500 wide); pedipalpal tibia length/width = 3.7; dorsum with faint coloration and separated into anterior and posterior portions; single specimen from Wilson Creek, Trinity County, California	*T. welbourni* **sp. n.**
–	Dorsum smaller (570–645 long, 420–480 wide); pedipalpal tibia length/width = 3.0–3.4; dorsum coloration variable	**7**
7 (6)	Medial suture longer (20.0–22.5); dorsum with faint coloration and separated into anterior and posterior portions; Rocky Mountains	*T. mulleni* **sp. n.**
–	Medial suture shorter (10.0–12.5); coloration variable; California & Oregon	**8**
8 (7)	Pedipalpal femur/genu = 1.52–1.64; anterio-medial platelets less elongate, length/width = 2.58–2.72; dorsal coloration usually orange and usually separated anteriorly and posteriorly; California, Oregon, & British Columbia	*T. walteri* **sp. n.**
–	Pedipalpal femur/genu = 1.69–1.82; anterio-medial platelets more elongate, length/width = 2.74–3.06; dorsal coloration faint orange or colorless; California	*T. nortoni* **sp. n.**
9 (4)	Dorsum more elongate, length/width = 1.6–1.8; anterior venter/genital field width = 1.25–1.33; southern Appalachians	*T. microbiscutella* **sp. n.**
–	Dorsum more ovoid, length/width = 1.1–1.5; anterior venter/genital field width = 0.74–1.13	**10**
10 (9)	Dorsal coloration diagnostic, with large dark spot anteriorly and adjoining red spot posteriorly; surface sculpturing, at least on dorsum, with pits large and distinct; dorsum **usually** shorter (540–580); southeastern (Mississippi & Alabama)	*T. ululata* **sp. n.**
–	Dorsal coloration not as above; surface pits not as large and distinct as above; dorsum **usually** longer (600–810) (540–650 in *T. sellersorum*)	**11**
11 (10)	Pedipalpal tibia more elongate, length/width = 4.5–4.9; dorsal coloration with faint, diffuse darkening centrally; known only from Coos County, New Hampshire	*T. folkertsae* **sp. n.**
–	Pedipalpal tibia less elongate, length/width = 3.0–4.2; dorsal coloration bold	**12**
12 (11)	Dorsal coloration distinctive, with bluish color separated into anterior and posterior portions and with central stripe bold red; known only from Pope County, Indiana	*T. pulchra* **sp. n.**
–	Dorsal coloration not as above, especially with central reddish stripe, when present, not as bold	**13**
13 (12)	Dorsum rounder, length/width = 1.19–1.21; pedipalpal tibia **usually** more elongate, length/width = 3.9–4.2; dorsal coloration with anterior and posterior portions connected medially; eastern (New Hampshire & Texas)	*T. priapus* **sp. n.**
–	Dorsum more ovoid, length/width = 1.23–1.42; pedipalpal tibia **usually** less elongate, length/width = 3.0–3.8, except *T. magnexa* (3.2–4.0), which has a stockier rostrum (2.2–3.0 in *T. magnexa*; 3.1–3.4 *T. priapus*)	**14**
14 (13)	Dorso-lateral platelets only partially fused to dorsal plate; dorsum longer (680–810); pedipalpal tibia longer (102–113); dorsal coloration highly variable in color (bluish, orange, or reddish) and pattern (anterior and posterior portions separated or connected; or dorsum uniformly purple); widespread in the east	*T. magnexa*
–	Dorso-lateral platelets entirely fused with dorsal plate; dorsum smaller (540–670); pedipalpal tibia shorter (72–95)	**15**
15 (14)	Rostrum stockier (length/width = 2.44–2.68); anterior venter/genital field width = 0.74–0.80; dorsal pattern often distinctive, with bold dark coloration separated into anterior and posterior portions **and** posterior portion not reaching the posterior of the dorsal plate and not encompassing the muscle scars; mostly eastern, but also found west of the 100^th^ Meridian	*T. sellersorum* **sp. n.**
–	Rostrum more elongate (length/width = 3.00–3.31); anterior venter/genital field width = 0.86–1.11; dorsal pattern not as above	**16**
16 (15)	Pedipalpal tibia more elongate, length/width = 3.5–3.7; genital field thinner (152–155); dorsal coloration separated into anterior and posterior portions with orange stripe medially; Alabama & Tennessee	*T. tysoni* **sp. n.**
–	Pedipalpal tibia less elongate, length/width = 3.0–3.1; genital field wider (168–173); dorsal coloration with anterior and posterior portions connected medially; known only from Washington County, Maine	*T. pendula* **sp. n.**
17 (3)	Dorsal pattern distinctive, with bold dark coloration separated into anterior and posterior portions **and** posterior portion not reaching the posterior of the dorsal plate and not encompassing the muscle scars; mostly eastern, but also found west of the 100^th^ Meridian	*T. sellersorum* **sp. n.**
–	Dorsal pattern not as above, when similar, posterior portion encompasses muscle scars; east of the 100^th^ Meridian	**18**
18 (17)	Genital field longer (175–230)	**19**
–	Genital field shorter (150–170)	**22**
19 (18)	Dgl-4 further from edge of dorsum, dorsum width/distance between Dgl-4 = 1.59–1.74; rostrum less elongate, length/width = 2.33–3.00; dorsal coloration hour-glass shaped; known only from Chattooga County, Georgia	*T. feminellai* **sp. n.**
–	Dgl-4 closer to edge of dorsum, dorsum width/distance between Dgl-4 = 1.26–1.48; rostrum **usually** more elongate, length/width = 3.05–3.38, except *T. magnexa* (2.75–3.00)	**20**
20 (19)	Anterior venter/genital field width = 0.93–1.03; dorsal coloration highly variable in color (bluish, orange, reddish) and pattern (anterior and posterior portions separated or connected; or dorsum uniformly purple); anterio-lateral platelets partially fused to dorsal plate; widespread in the east	*T. magnexa*
–	Anterior venter/genital field width = 0.66–0.83; anterio-lateral platelets fused or partially fused to dorsal plate	**21**
21 (20)	Dorsal coloration faint, with anterior and posterior portions connected medially; dorsum length/width = 1.21–1.32; pedipalpal tibia length/width = 3.50–3.92; lentic (slow-moving lakes & rivers); Midwestern (Wisconsin & Manitoba)	*T. indistincta*
–	Dorsal coloration highly variable, reddish-purple to purple (occasionally bluish) separated into anterior and posterior portions (rarely connected medially, but if so, then only faintly); dorsum length/width = 1.38–1.44; pedipalpal tibia length/width = 2.92–3.61; lotic (fast-moving streams); Appalachians	*T. delicatexa*
–	Dorsal coloration bold, dark purple, anterior and posterior portions connected medially; dorsum length/width = 1.26–1.38; pedipalpal tibia length/width = 2.42–2.95; lotic (fast-moving streams); southern Appalachians (North Carolina)	*T. whitneyae* **sp. n.**
22 (18)	Dorsal coloration either reddish- or bluish-purple, prominent, and with anterior and posterior portions separate; genital field width 152.5–165.0; subcapitular ventral length 317–335; eastern (Missouri, New Brunswick, and Tennessee)	*T. malarkeyorum* **sp. n.**
–	Body distinctive for having a bluish tinge, dorsal coloration bluish, with anterior and posterior portions connected medially; genital field width 120–145.0; subcapitular ventral length 310–330; known only from an upper tributary of Factory Creek, Wayne County, Tennessee	*T. caerulea* **sp. n.**
–	Dorsal coloration bluish and bold, with anterior and posterior portions separate, exposing red medially; genital field width 142.5–160; subcapitular ventral length 290–315; Interior Highlands (Arkansas and Missouri)	*T. biscutella* **sp. n.**
23 (2)	Dgl-4 further from edge of dorsum, dorsum width/distance between Dgl-4 = 1.80–3.30; dorsum rounder, length/width = 1.16–1.31; east of the Rocky Mountains	Raptor Group (in part*)...**24**
–	Dgl-4 closer to edge of dorsum, dorsum width/distance between Dgl-4 = 1.17–1.73; body variable; eastern or western	**30**
	*Except *T. daemon* (couplet 78) and *T. danielleae* (couplet 67)
24 (23)	Anterior venter longer (205–240); subcapitulum more elongate, ventral length/height = 2.98–3.18; pedipalpal tibia more elongate, length/width = 6.0–7.6; dorsal coloration restricted posteriorly, usually with anterior extension; Appalachians	*T. raptor* **sp. n.**
–	Anterior venter shorter (150–200); subcapitulum less elongate, ventral length/height = 2.26–2.90; pedipalpal tibia less elongate, length/width = 4.0–6.0; dorsal coloration variable	**25**
25 (24)	Dgl-4 closer from edge of dorsum, dorsum width/distance between Dgl-4 = 1.8–2.1; dorsal coloration separated into anterior and posterior portions; east of Rocky Mountains	*T. irapalpa* **sp. n.**
–	Dgl-4 further from edge of dorsum, dorsum width/distance between Dgl-4 = 2.2–3.3; dorsal coloration variable; Appalachians	**26**
26 (25)	Rostrum less elongate, length/width = 2.7–3.2; femoral tubercles shorter (12–14); dorsal coloration reddish and usually centralized, but occasionally resembling *T. raptor*; eastern, not known from mountain streams	*T. gnoma* **sp. n.**
–	Rostrum more elongate, length/width = 3.5–4.3; femoral tubercles longer (15–25), except for *T. ivyae* (13–17); dorsal coloration variable	27
27 (26)	Anterior venter longer (180–195); dorsal coloration with anterior and posterior **usually** connected; northeast	*T. mjolniri* **sp. n.**
–	Anterior venter shorter (152–170); dorsal coloration variable	**28**
28 (27)	Rostrum more elongate, length/width = 4.0–4.2; anterior venter/medial suture = 6–7; dorsal coloration purple posteriorly that extends anteriorly to the anterio-medial platelets; Florida	*T. ivyae* **sp. n.**
–	Rostrum less elongate, length/width = 3.5–3.9; anterior venter/medial suture = 8–10; dorsal coloration variable; Appalachians	**29**
29 (28)	Pedipalpal femoral tubercles shorter (17.50); subcapitulum ventral length/height = 2.39; dorsal coloration restricted posteriorly with anterior extension, venter colorless; single specimen from Charlotte County, New Brunswick	*T. elusiva* **sp. n.**
–	Pedipalpal femoral tubercles longer (21–25); subcapitulum ventral length/height = 2.48–2.73; dorsal coloration separated into anterior and posterior portions, venter coloration bold; known from Tennessee and Virginia	*T. racupalpa* **sp. n.**
30 (23)	Rostrum strongly upturned; subcapitulum (including rostrum) expanded laterally, nearly square in cross-section; eastern	**Erectirostra Group**...**31**
–	Rostrum usually straight, occasionally upturned distally or rarely slightly upturned (*T. reduncarostra*); subcapitulum unmodified, usually laterally compressed, at least not square in cross-section; eastern or western	33
31 (30)	Rostrum length/width = 1.57–1.62; dorsal coloration faint to absent; Sevier County, Tennessee	*T. karambita* **sp. n.**
–	Rostrum length/width = 1.72–2.09; dorsal coloration bolder	**32**
32 (31)	Anterio-lateral platelets less elongate, length/width = 2.52–2.69; Appalachians	*T. erectirostra* **sp. n.**
–	Anterio-lateral platelets more elongate, length/width = 2.96–3.00; Ouachitas (Arkansas & Oklahoma)	*T. robisoni* **sp. n.**
33 (30)	Dorsal plate with anterio-medial extension covering nearly half the length of the anterio-medial platelets; pedipalpal femur with large, tuberculate ventral extension **and** genu without ventral extension	*T. dimorpha* **sp. n.**
–	Dorsal plate without anterior extension; pedipalpal ventral extensions not as above (femoral tubercle never as pronounced; if genu without ventral extension, then femoral extension either absent or lamellate)	**34**
34 (33)	Rostrum dorsally with patch of strong dentation; southern Appalachians	*T. dentirostra* **sp. n.**
–	Rostrum without dentation; eastern or western	**35**
35 (34)	Pedipalpal tibia more elongate, length/width = 5.3–5.9; dorsum short (475–530); dorsal coloration purple posteriorly	Nigroalba Group (in part*)...**36**
–	Pedipalpal tibia less elongate, length/width = 1.7–5.0; dorsum variable (495–900); coloration variable	**37**
	*except for *T. dentirostra* (couplet 34) and *T. flangipalpa* (couplet 66)
36 (35)	Dorsum larger (500–530); gnathosomal bay length/width = 1.52–1.55; subcapitulum ventral length/width = 3.00–3.14; dorsum lacking reddish coloration; eastern	*T. nigroalba*
–	Dorsum smaller (475–500); gnathosomal bay length/width = 1.30–1.50; subcapitulum ventral length/width = 3.14–3.30; dorsum with reddish coloration just anterior to purple; Ouachitas (Arkansas)	*T. solisorta* **sp. n.**
37 (35)	Hind coxal margin indistinct **or** incomplete	**38**
–	Hind coxal margin distinct and complete	**52**
38 (37)	Gnathosomal bay elongate (a), length/width = 4.4–5.4; pedipalpal tibia less elongate, length/width = 1.7–2.0; pedipalpal femur and genu without ventral extensions; southwestern	**Rala Group (in part)**...**39**
–	Gnathosomal bay unmodified (aa), length/width = 1.3–2.8; pedipalpal tibia more elongate, length/width = 2.7–5.0; pedipalpal femur with ventral extension and genu with or without ventral extension; eastern or western	**40**
39 (38)	Dorsum elongate, length/width = 1.53–1.82; pedipalpal femur/genu = 1.04–1.17; subcapitulum less elongate, ventral length/width = 1.96; known only from Catron County, New Mexico	*T. boettgeri*
–	Dorsum ovoid, length/width = 1.29–1.34; pedipalpal femur/genu = 1.3–1.5; subcapitulum more elongate, ventral length/width = 2.5–2.7; Arizona and Mexico	*T. kurtvietsi*
40 (38)	Rostrum short and conical, length/height = 1.70–1.83; pedipalpal tibia short and thick, length/width = 2.75–2.88; body colorless; known from Arizona and New Mexico	**Rala Group (in part)**...*T. rala*
–	Rostrum unmodified, length/width = 2.45–3.8; pedipalpal tibia unmodified, length/width = 3.0–5.0; body coloration variable	**41**
41 (40)	Pedipalpal femur with large flanged ventral extension, and pedipalpal genu either with flanged, dentate ventral extension, or without ventral extension; subcapitulum ventral length/width = 3.0–3.5; southwestern	**Rala Group (in part)**...**42**
–	Pedipalpal femur and genu with tuberculate ventral extensions; subcapitulum ventral length/width = 2.0–2.9; eastern or western	**43**
42 (41)	Pedipalpal femur with large flanged ventral extension that extends to at least mid-length of genu, and genu without ventral extension; dorsum more elongate, length/width = 1.4–1.6	*T. lamellipalpis*
–	Pedipalpal femur and genu with flanged, dentate ventral extensions that are not greatly expanded; dorsum rounder, length/width = 1.2–1.3	*T. keesdavidsi*
43 (41)	Hind coxal margins distinct and complete; dorsal coloration restricted to a single dark spot anteriorly with an orange spot posterior to the dark spot (similar only to *T. unimaculata* and *T. ululata*); known only from Tyler County, Texas	*T. kringi* **sp. n.**
–	Hind coxal margins instinct or incomplete; dorsal coloration not as above	**44**
44 (43)	Pedipalpal tibia longer (100.0–107.5); dorsal coloration faint, restricted posteriorly; Catron & Grant Counties, New Mexico	*T. sharkeyi* **sp. n.**
–	Pedipalpal tibia shorter (80.0–95); dorsal coloration variable; eastern or Pacific northwest	**45**
45 (44)	Pedipalpal femoral tubercle truncate; anterior venter shorter (140–152.5); dorsal coloration bold, reddish purple, and separated into anterior and posterior portions that are connected by yellowish band; Fayette & Somerset Counties, Pennsylvania	*T. skvarlai* **sp. n.**
–	Pedipalpal femoral tubercle conical; anterior venter longer (155–213); dorsal coloration variable; eastern or western	**46**
46 (45)	Pedipalpal tibia less elongate, length/width = 3.00–3.13; gnathosomal bay length/width = 2.4–2.8; dorsal coloration bold purple, excepting platelets; known only from Curry County, Oregon	*T. regalis* **sp. n.**
–	Pedipalpal tibia more elongate, length/width = 3.27–4.50; gnathosomal bay length/width = 1.3–2.2; dorsal coloration bluish or reddish purple separated into anterior and posterior portions; eastern	**Eastern 4-Plates**...**47**
47 (46)	Anterior venter longer (202–213); subcapitulum shorter (112–120); coloration bold; Appalachians (Tennessee & Pennsylvania)	*T. glomerabilis* **sp. n.**
–	Anterior venter shorter (155–190); subcapitulum taller (122–150); coloration faint or bold	**48**
48 (47)	Pedipalpal tibia length/width = 3.9–4.2; rostrum length/width = 3.25–3.85; dorsal coloration bold, bluish-purple, and separated into anterior and posterior portions that may or may not be connected; Lauderdale County, Alabama	*T. pollani* **sp. n.**
–	Pedipalpal tibia length/width = 3.2–3.6; rostrum length/width = 2.5–3.0; dorsal coloration variable	**49**
49 (48)	Genital field thinner (137–145); rostrum less elongate, length/width = 2.5–2.7; dorsal coloration faint and bluish-purple; Appalachians (Tennessee & Pennsylvania)	*T. shubini* **sp. n.**
–	Genital field wider (145–160); rostrum more elongate, length/width= 2.8–3.1; dorsal coloration bold and reddish-purple	**50**
50 (49)	Body smaller (dorsum 550); female known from single specimen from Little Flat Brook, Sussex County, New Jersey	*T. rufoalba*
–	Body larger (dorsum 605–670)	**51**
51 (50)	Pedipalpal genu shorter (63.8); subcapitulum shorter, ventral length 310; rostrum shorter (126); anterio-medial platelets more elongate, length/width = 2.83; female known from a single specimen from Morris County, New Jersey	*T. kittatinniana*
–	Pedipalpal genu longer (70.0–75.0); subcapitulum longer, ventral length 330–355; rostrum longer (130–140); anterio-medial platelets less elongate, length/width = 2.33–2.54; southern Appalachians (Tennessee & Carolinas)	*T. dunni* **sp. n.**
52 (37)	Rostrum short and conical, length/width = 1.60–2.42	Tricolor Group (in part*)...**53**
–	Rostrum unmodified or elongate, length/width = 2.48–4.83	**65**
	*except for *T. dimorpha* (couplet 33)
53 (52)	Pedipalpal femur and genu with tubercles absent or indistinct; pedipalpal femur/genu = 1.72–1.79; colorless, or rarely with dorsum pinkish centrally; Wayne County, Indiana	*T. hoosieri* **sp. n.**
–	Pedipalpal femur and genu with tubercles present and obvious; pedipalpal femur/genu = 1.35–1.69, (except *T. leviathan* = 1.68–1.73); coloration variable	**54**
54 (53)	Dorsum colorless or with diffuse coloration conforming to the standard pattern for most *Torrenticola*, separated into anterior and posterior portions; eastern or western	**55**
–	Dorsum colorful, adorned with distinctive patterning; eastern	**60**
55 (54)	Pedipalpal tibia more elongate, length/width = 3.9–4.2; known only from Mendocino County, California	*T. leviathan* **sp. n.**
–	Pedipalpal tibia less elongate, length/width = 2.9–3.5; eastern or western	**56**
56 (55)	Pedipalpal femur/genu = 1.60–1.69; Interior Highlands	*T. pearsoni* **sp. n.**
–	Pedipalpal femur/genu = 1.32–1.55; western	**57**
57 (56)	Dorsum rectangular; anterio-medial platelets square-shaped, length/width = 1.4–1.7; widespread in the west	*T. ellipsoidalis*
–	Dorsum ovoid or round; anterio-medial platelets more rectangular, length/width = 1.9–2.5	**58**
58 (57)	Rostrum very short, length/width = 1.62–1.76; subcapitulum taller, ventral length/height = 1.78–1.92; dorsum colorless, or with faint coloration separated into anterior and posterior portions; British Columbia, Oregon, & California	*T. olliei* **sp. n.**
–	Rostrum more elongate, length/width = 1.96–2.15; subcapitulum unmodified, ventral length/height = 2.18–2.39	**59**
59 (58)	Anterio-lateral platelets/anterio-medial platelets = 1.62; rostrum length/width = 2.15; known from single specimen collected from trout stomachs in Wyoming	*T. occidentalis*
–	Anterio-lateral platelets/anterio-medial platelets = 1.18–1.27; rostrum length/width = 1.96–2.08; widespread in the west	*T. sierrensis*
60 (54)	Dorsal coloration diagnostic, with dark anterior portion nearly restricted to anterio-medial platelets, either with or without dark posterior spot, and with dark portions connected by bold red stripe; body more elongate, dorsum more ovoid, length/width = 1.41–1.54; Polk & Montgomery Counties, Arkansas	*T. larvata*
–	Dorsal coloration variable, but not as above; body rounder, dorsum length/width = 1.20–1.39, except *T. cardia* (1.39–1.47)	**61**
61 (60)	Dorsal coloration diagnostic, with single dark, bell-shaped spot anteriorly, with bold red posteriorly (similar only to *T. ululata* and *T. kringi*); Arkansas & New Brunswick	*T. unimaculata* **sp. n.**
–	Dorsal coloration variable, but never as above	**62**
62 (61)	Dorsal coloration diagnostic, with three dark spots (two posterior, one anterior); widespread east of the 100^th^ Meridian	*T. trimaculata*
–	Dorsal coloration variable, but never with three separate spots	**63**
63 (62)	Dorsal coloration diagnostic, restricted to a dark stripe anteriorly; rostrum less elongate, length/width = 1.8–2.0; known only from Aroostook County, Maine	*T. mohawk* **sp. n.**
–	Dorsal coloration not as above; rostrum more elongate, length/width = 2.1–2.5	**64**
64 (63)	Dorsal coloration variable, but diagnostic, often appearing as two posterior spots and an anterior spot that are merged together, occasionally anterior spot is reduced to a stripe connected to the posterior spots; dorsum rounder, length/width = 1.20–1.35; subcapitulum ventral length/width = 2.15–2.42; Appalachians	*T. tricolor*
–	Dorsal coloration with reddish-purple, bluish-purple, or orange spot medially extending in a strip anteriorly often to the anterio-medial platelets; dorsum more ovoid, length/width = 1.39–1.47; subcapitulum ventral length/width = 2.04–2.12; Appalachians	*T. cardia* **sp. n.**
65 (52)	Pedipalpal tibia more elongate, length/width = 4.4–5.0	**66**
–	Pedipalpal tibia less elongate, length/width = 2.5–4.3	**68**
66 (65)	Anterior venter longer (235–255); dorsum shorter (530–580); subcapitulum ventral length/width = 3.4–3.5; southeastern	*T. flangipalpa* **sp. n.**
–	Anterior venter shorter (150–195); dorsum longer (610–690); subcapitulum ventral length/width = 2.2–2.7	**67**
67 (66)	Anterior venter/medial suture = 10–17; rostrum less elongate, length/width = 3.17–3.38; dorsal coloration bluish purple, restricted posteriorly; Arizona & New Mexico	*T. raptoroides* **sp. n.**
–	Anterior venter/medial suture = 6–8; rostrum more elongate, length/width = 3.44–3.75; dorsal coloration reddish-purple posteriorly, extending anteriorly to the edge of the dorsal plate; Floyd & Chattooga Counties, Georgia	*T. danielleae* **sp. n.**
68 (65)	Anterior venter longer (250–340)*	**69**
–	Anterior venter shorter (150–235)*	**72**
	*T. oregonensis (230–270) key in both directions
69 (68)	Pedipalpal femur and genu without ventral extensions; pedipalpal tibia less elongate, length/width = 2.8–3.1; dorsum more elongate, length/width = 1.52–1.68; eastern	*T. projector*
–	Pedipalpal femur and genu with ventral extensions; pedipalpal tibia more elongate, length/width = 3.6–4.2; dorsum more ovoid, length/width = 1.24–1.44; western	**70**
70 (69)	Anterior venter/genital field length = 1.7–1.9; gnathosomal bay length/width = 1.2–1.6; subcapitulum more elongate, ventral length/width = 3.41–4.11; dorsal coloration bold purple restricted posteriorly; widespread in the west	*T. tahoei*
–	Anterior venter/genital field length = 1.0–1.5; gnathosomal bay length/width = 1.7–2.3; subcapitulum less elongate, ventral length/width = 2.63–3.38; dorsal coloration faint or colorless	**71**
71 (70)	Dorsum shorter (615–710); subcapitulum more elongate, ventral length/width = 3.0–3.4; dorsal coloration nearly colorless, faint purple on entire dorsal plate; Pacific coastal ranges	*T. oliveri* **sp. n.**
–	Dorsum longer (760–840); subcapitulum less elongate, ventral length/width = 2.6–2.7; dorsal coloration with faint purple restricted posteriorly; Oregon	*T. oregonensis* **sp. n.**
72 (68)	Pedipalpal femur/genu = 1.60–1.90	**73**
–	Pedipalpal femur/genu = 1.37–1.53	**80**
73 (72)	Body elongate, dorsum length/width = 1.9–2.1; anterior venter/genital field length = 1.4–1.5; dorsal coloration purple and separated into anterior and posterior portions; Tishomingo County, Mississippi	**Elongata Group (in part)**...*T. elongata* **sp. n.**
–	Body round or ovoid, dorsum length/width = 1.2–1.65; anterior venter/genital field length = 0.9–1.3; eastern or western	74
74 (75)	Rostrum upturned; pedipalpal tibia less elongate, length/width = 2.6–2.9; Appalachians	**Elongata Group (in part)**...*T. reduncarostra* **sp. n.**
–	Rostrum straight, or only slightly upturned distally; pedipalpal tibia more elongate, length/width = 3.2–4.4; eastern or western	**75**
75 (74)	Rostrum more elongate, length/width = 3.3–3.8; dorsum thinner (380–390); dorsal coloration either reddish-purple separated into anterior and posterior portions, or diagnostic, dark blue with red central oval; red morph from Appalachians (Tennessee, South Carolina, New Brunswick); dark morph known only from Tellico River, Monroe County, Tennessee	**Elongata Group (in part)**...*T. gorti* **sp. n.**
–	Rostrum less elongate, length/width = 2.5–3.2; dorsum wider (420–550); dorsal coloration not as above	**76**
76 (75)	Genital field wider (167–183); dorsal coloration bluish-purple separated into anterior and posterior portions, often faint, and connected by red band; California & southwestern Oregon	*T. ventura*
–	Genital field thinner (137–160); dorsal coloration bluish-purple or reddish-purple, separated into anterior and posterior portions, usually bold, and connected by faint red band; eastern	**77**
77 (76)	Medial suture shorter (10.0–15.0); anterior venter shorter (155–188)	**78**
–	Medial suture longer (20.5–30.0); anterior venter longer (190–225)	**Neoanomala Group**...**79**
78 (77)	Dorsum less elongate, length/width = 1.26–1.34; dorsal coloration reddish purple separated into anterior and posterior portions, but with reddish color of anterio-medial platelets distinctly brighter; known only from Clay County, Alabama	*T. daemon* **sp. n.**
–	Dorsum more elongate 1.35–1.41; dorsal coloration purple and separated into anterior and posterior portions; known only from Haywood County, North Carolina	*T. bondi* **sp. n.**
79 (77)	Anterio-lateral platelets less elongate, length/width = 2.62–2.68; Montgomery & Newton Counties, Arkansas	*T. interiorensis* **sp. n.**
–	Anterio-lateral platelet more elongate, length/width = 2.86–3.09; eastern, including Arkansas	*T. neoanomala*
80 (72)	Dorsal plate with longitudinal striations anteriorly; dorsal coloration bold purple separated into anterior and posterior portions, with faint orange medially; southern Appalachians	*T. arktonyx* **sp. n.**
–	Dorsal plate without longitudinal striations anteriorly; dorsal coloration variable, but not as above; western	**81**
81 (80)	Dorsum longer (700–850)	**82**
–	Dorsum shorter (530–640)	**83**
82 (81)	Anterior venter shorter (175–213); pedipalpal tibia less elongate, length/width = 2.7–3.2; subcapitulum less elongate, ventral length/width = 2.2–2.5; dorsal coloration variable (faint orange or purple throughout dorsal plate, or colorless), but rarely restricted posteriorly; widespread in the west	*T. multiforma*
–	Anterior venter longer (230–270); pedipalpal tibia more elongate, length/width = 3.8–4.0; subcapitulum more elongate, ventral length/width = 2.6–2.7; dorsal coloration with faint purple restricted posteriorly; Oregon	*T. oregonensis* **sp. n.**
83 (81)	Dorsum rounder, length/width = 1.15–1.26; pedipalpal tibia more elongate, length/width = 4.0–4.3; dorsum colorless; southern California	*T. wiedenmanni* **sp. n.**
–	Dorsum ovoid or elongate, length/width = 1.29–1.64; pedipalpal tibia less elongate, length/width = 2.6–3.8; dorsal coloration variable; eastern or western	Miniforma Group (in part*)...**84**
	*except *T. oliveri* (couplet 71)
84 (83)	Rostrum elongate, length/width = 4.5–4.9; gnathosomal bay length/width = 2.42–2.59; dorsum colorless; California & Oregon	*T. pinocchio* **sp. n.**
–	Rostrum unmodified, length/width = 2.6–3.2; gnathosomal bay length/width = 1.49–2.38; dorsum often purplish	85
85 (84)	Pedipalpal femoral tubercle broad and flat; pedipalpal tibia/femur = 0.55–0.61; anterior venter/genital field length = 1.19–1.28; dorsal coloration usually absent, rarely with purple posteriorly; California & Oregon	*T. copipalpa* **sp. n.**
–	Pedipalpal femoral tubercle tuberculate; pedipalpal tibia/femur = 0.65–0.74; anterior venter/genital field length = 0.99–1.11	**86**
86 (85)	Pedipalpal tibia more elongate, length/width = 3.13–3.29; subcapitulum ventral length/height = 2.77–2.82; dorsal coloration faint purple posteriorly; known only from Whitewater Creek, Catron County, New Mexico	*T. manni* **sp. n.**
–	Pedipalpal tibia less elongate, length/width = 2.71–3.11; subcapitulum ventral length/height = 2.49–2.68; Rocky Mountains & Pacific Ranges	87
87 (86)	Rostrum more elongate, length/width = 3.13; anterio-lateral platelet/anterio-medial platelet = 1.36; female known from single specimen from Prairie Creek in Humboldt County, California	*T. miniforma*
–	Rostrum less elongate, length/width = 2.59–2.91; anterio-lateral platelet/anterio-medial platelet = 1.43–1.55	**88**
88 (87)	Rostrum more elongate, length/width = 2.72–2.91; dorsal coloration purple posteriorly and encroaching anteriorly nearly to the platelets, occasionally including the platelets; Rocky Mountains (Idaho & Montana)	*T. rockyensis* **sp. n.**
–	Rostrum less elongate, length/width = 2.59–2.68; dorsal coloration purple posteriorly, but usually not encroaching anteriorly; Oregon & Washington	*T. pacificensis* **sp. n.**
89 (1)	Dorso-lateral platelets partially or entirely fused to dorsal plate; hind coxal margin indistinct; subcapitulum unmodified	**2-Plate Groups**...**90**
–	Dorso-lateral platelets free from dorsal plate; hind coxal margin indistinct or distinct; subcapitulum unmodified or modified	**109**
90 (89)	Anterior venter longer (220–275)*; eastern or western	**91**
–	Anterior venter shorter (150–210)*; primarily eastern, but *T. sellersorum*, which is mostly eastern, is also known from Manitoba, South Dakota, and New Mexico	**Eastern 2-Plates (in part)**...**100**
	*T. magnexa (207–240) and *T. indistincta* (190–235) key in both directions
91 (90)	Eastern (east of the 100^th^ Meridian)	**Eastern (in part) & Partial 2-Plates**...**92**
–	Western (west of the 100^th^ Meridian)	**Western 2-Plates**...**98**
92 (91)	Dorsal coloration with single dark, bell-shaped spot anteriorly, with bold red posteriorly (similar only to *T. unimaculata*); southeastern (Alabama & Mississippi)	*T. ululata* **sp. n.**
–	Dorsal coloration not as above	**93**
93 (92)	Anterior venter/genital field width = 2.5–2.9	**94**
–	Anterior venter/genital field width = 1.4–2.2	**95**
94 (93)	Dorsum more elongate, length/width = 1.39–1.48; pedipalpal tibia/femur = 0.75–0.81; dorsum with bluish color separated into anterior and posterior portions, with **faint** reddish coloration medially; Alabama & Tennessee	*T. tysoni* **sp. n.**
–	Dorsum less elongate, length/width = 1.53–1.61; pedipalpal tibia/femur = 0.69–0.73; dorsum with bluish color separated into anterior and posterior portions, with **bold** red coloration medially; known only from Pope County, Indiana	*T. pulchra* **sp. n.**
95 (93)	Rostrum more elongate, length/width = 3.1–3.3; dorsum rounder, length/width = 1.18–1.22; dorsal coloration dark purple posteriorly that extends anteriorly; eastern (New Hampshire & Texas)	*T. priapus* **sp. n.**
–	Rostrum less elongate, length/width = 2.5–3.0; dorsum more ovoid, length/width = 1.27–1.52	**96**
96 (95)	Pedipalpal tibia less elongate, length/width = 3.1–3.5; anterior venter/genital field width = 1.5–1.7; lentic (slow-moving lakes & rivers); Midwestern (Wisconsin & Manitoba)	*T. indistincta*
–	Pedipalpal tibia more elongate, length/width = 3.7–4.4; anterior venter/genital field width = 1.8–2.2; lotic (fast-moving streams)	**97**
97 (96)	Dorsum more ovoid, length/width = 1.35–1.43; medial suture shorter (62–85); dorsal coloration separated into anterior and posterior portions, but color variable (bluish or reddish purple); widespread in the east	*T. magnexa*
–	Dorsum rounder, length/width = 1.27–1.29; medial suture longer (105–125); dorsal coloration with faint, diffuse darkening centrally; known only from Coos County, New Hampshire	*T. folkertsae* **sp. n.**
98 (91)	Genital field longer (130–140); Rocky Mountains	*T. mulleni* **sp. n.**
–	Genital field shorter (115–125); west of Rocky Mountains & northern Rocky Mountains (British Columbia)	**99**
99 (98)	Pedipalpal femur longer (95.0–100.0); pedipalpal tibia more elongate, length/width = 3.05–3.10; California, Oregon, Washington, British Columbia	*T. walteri* **sp. n.**
–	Pedipalpal femur shorter (85.0–92.5); pedipalpal tibia less elongate, length/width = 2.73–3.00; California	*T. nortoni* **sp. n.**
100 (90)	Dorsum longer (480–630)	**101**
–	Dorsum shorter (390–470)	**105**
101 (100)	Dgl-4 further from edge of dorsum, dorsum width/distance between Dgl-4 = 1.5–1.7; rostrum more elongate, length/width = 3.1–3.4; dorsal coloration hour-glass shaped; known only from Chattooga County, Georgia	*T. feminellai* **sp. n.**
–	Dgl-4 closer to edge of dorsum, dorsum width/distance between Dgl-4 = 1.2–1.4; rostrum less elongate, length/width = 2.5–2.9	**102**
102 (101)	Pedipalpal tibia more elongate, length/width = 3.7–4.0; anterior venter/genital field width = 1.80–1.98; dorsal coloration variable, but anterior and posterior portions rarely faint and rarely connected medially; widespread in the east	*T. magnexa*
–	Pedipalpal tibia less elongate, length/width = 2.4–3.5; anterior venter/genital field width = 1.50–1.73; dorsal coloration with anterior and posterior portions connected medially	**103**
103 (102)	Pedipalpal tibia more elongate, length/width = 3.1–3.5; gnathosomal bay less elongate, length/width = 1.8–2.0; dorsal coloration faint; lentic (slow-moving lakes & rivers); Midwestern (Wisconsin & Manitoba)	*T. indistincta*
–	Pedipalpal tibia less elongate, length/width = 2.4–2.8; gnathosomal bay more elongate, length/width = 2.3–2.9; dorsal coloration bold; lotic (fast-moving streams)	**104**
104 (103)	Gnathosomal bay length/width = 2.3–2.5; anterior venter/genital field width = 1.5–1.6; southern Appalachians (North Carolina)	*T. whitneyae* **sp. n.**
–	Gnathosomal bay length/width = 2.90; anterior venter/genital field width = 1.73; dorsal coloration with anterior and posterior portions connected medially; known only from Washington County, Maine	*T. pendula* **sp. n.**
105 (100)	Anterior venter/genital field width = 2.0–2.3; dorsum length/width = 1.63–1.69; southern Appalachians	*T. microbiscutella* **sp. n.**
–	Anterior venter/genital field width = 1.3–1.9; dorsum length/width = 1.37–1.56	**106**
106 (105)	Body with bluish tinge; dorsal coloration bluish and anterior and posterior portions connected medially; known only from an upper tributary of Factory Creek, Wayne County, Tennessee	*T. caerulea* **sp. n.**
–	Body without bluish tinge; dorsal coloration separated into anterior and posterior portions, not connected medially, and either bluish-purple or reddish-purple, never similar to *T. caerulea*	**107**
107 (106)	Dorsal coloration reddish-purple (occasionally more purple than reddish, but never bluish purple); Appalachians	*T. delicatexa*
–	Dorsal coloration bluish-purple	**108**
108 (107)	Dorsal coloration bold and distinct, with bright red medially; dorsum length/width = 1.37–1.42; anterio-lateral platelet length/width = 2.58–2.74; pedipalpal femur/genu = 1.62–1.64; Interior Highlands (Arkansas and Missouri)	*T. biscutella* **sp. n.**
–	Dorsal coloration of medium boldness; dorsum length/width = 1.42–1.54; anterio-lateral platelet length/width = 2.76–3.00; pedipalpal femur/genu = 1.60–1.67; mostly eastern, but also found just west of the 100^th^ Meridian	*T. sellersorum* **sp. n.**
–	Dorsal coloration faint; dorsum length/width = 1.42–1.56; anterio-lateral platelet length/width = 2.43–2.61; pedipalpal femur/genu = 1.39–1.62; eastern (Missouri, New Brunswick, and Tennessee)	*T. malarkeyorum* **sp. n.**
109 (89)	Pedipalps highly modified, greatly enlarged, femur with very large tubercle, genu without ventral extension; dorsal plate with medial extension that covers nearly half of the anterio-medial platelets; venter highly modified, with coxae forming large plate that covers insertions of leg IV, coxa III+IV suture incomplete, and genital field triangular; colorless, but legs occasionally with faint purple tinge; Texas	*T. dimorpha* **sp. n.**
–	Pedipalps unmodified (femoral tubercle never as robust; if genu without extension, then femoral extension is absent or lamellate); dorsal plate without medial extension; venter unmodified	**110**
110 (109)	Rostrum strongly upturned and with dentation dorsally; subcapitulum (including rostrum) expanded laterally, nearly square in cross-section; eastern	**Erectirostra Group**...**111**
–	Rostrum usually straight, occasionally upturned distally or slightly upturned (*T. reduncarostra*), usually without dentation (*T. dentirostra* has dentation, but a straight rostrum); subcapitulum unmodified, usually laterally compressed, at least not square in cross-section; eastern or western	**112**
111 (110)	Rostrum length/width = 1.63–1.95; dorsal coloration faint to absent; Sevier County, Tennessee	*T. karambita* **sp. n.**
–	Rostrum length/width = 2.00–2.17; dorsal coloration bold; Arkansas & New Brunswick	*T. erectirostra* **sp. n.**
112 (110)	Dgl-4 further from edge of dorsum, dorsum width/distance between Dgl-4 = 2.0–2.8; eastern	Raptor Group (in part*)...**111**
–	Dgl-4 closer to edge of dorsum, dorsum width/distance between Dgl-4 = 1.1–1.9; eastern or western	**117**
	*except *T. daemon* (couplet 131), *T. elusiva* (couplet 140), *T. raptor* (120)
113 (112)	Dorsum smaller (420–495 long; 355–375 wide); pedipalpal tibia less elongate, length/width = 3.8–4.5; rostrum less elongate, length/width = 2.5–3.1; eastern, not found in mountain streams	*T. gnoma* **sp. n.**
–	Dorsum larger (520–570 long; 390–475 wide); pedipalpal tibia more elongate, length/width = 4.7–5.5; rostrum more elongate, length/width = 3.8–4.3	**114**
114 (113)	Dgl-4 further from edge of dorsum, dorsum width/distance between Dgl-4 = 2.46–2.71; femur/genu = 2.1–2.2; known only from two males from Monroe County, Tennessee	*T. longitibia* **sp. n.**
–	Dgl-4 closer to edge of dorsum, dorsum width/distance between Dgl-4 = 2.19–2.39; femur/genu = 1.8–2.0	**115**
115 (116)	Anterior venter/medial suture = 4.4–4.6; pedipalp tibia longer (107–110); Tennessee and Virginia	*T. racupalpa* **sp. n.**
–	Anterior venter/medial suture = 2.7–3.7; pedipalp tibia shorter (90–103)	**116**
116 (115)	Subcapitulum more elongate, ventral length/width = 2.82–3.00; pedipalpal tibia longer (100–103); northeast	*T. mjolniri* **sp. n.**
–	Subcapitulum less elongate, ventral length/width = 2.57–2.75; pedipalpal tibia shorter (90–98); Florida	*T. ivyae* **sp. n.**
117 (112)	Pedipalpal femur with greatly expanded, flattened ventral extension that extends to at least mid-length of genu, and genu without ventral extension; New Mexico	*T. lamellipalpis*
–	Pedipalpal femur ventral extension not as above, genu with or without ventral extension	**118**
118 (117)	Pedipalpal tibia more elongate*, length/width = 5.0–5.6	**119**
–	Pedipalpal tibia less elongate*, length/width = 1.8–4.9	**122**
	*T. keesdavidsi (4.8–5.2) key in both directions
119 (118)	Dorsum larger (500–610 long; 410–450 wide); rostrum more elongate, length/width = 3.2–4.4	**120**
–	Dorsum smaller (425–460 long; 290–320 wide); rostrum less elongate, length/width = 2.7–3.0	Nigroalba Group (in part*)...**121**
	*except for *T. dentirostra* (couplet 162) and *T. flangipalpa* (couplet 142)
120 (119)	Pedipalp ventral extensions flanged; Dgl-4 further from edge of dorsum, dorsal width/distance between Dgl-4 = 1.6–1.9; rostrum more elongate, length/width = 3.8–4.4; dorsal coloration variable, but distinct; Appalachians	*T. raptor* **sp. n.**
–	Pedipalp ventral extensions tuberculate; Dgl-4 closer to edge of dorsum, dorsal width/distance between Dgl-4 =1.4–1.5; rostrum less elongate, length/width = 3.3–3.6; dorsal coloration indistinct or colorless; southwestern	*T. keesdavidsi*
121 (119)	Anterior venter/medial suture = 2.54–2.77; dorsum thinner, width = 290–300; dorsal coloration purple posteriorly, usually without red anteriorly; Ozarks (Missouri) & Appalachians	*T. nigroalba*
–	Anterior venter/medial suture = 2.87–3.26; dorsum thicker, width = 305–320; dorsal coloration purple posteriorly, usually with red anteriorly; Ouachitas (Arkansas)	*T. solisorta* **sp. n.**
122 (118)	Hind coxal margin indistinct or incomplete*	**123**
–	Hind coxal margin distinct and complete*	**137**
	* *T. daemon* (can appear incomplete) key in both directions
123 (122)	Gnathosomal bay thin, length/width = 3.8–5.0; pedipalpal tibia less elongate, length/width = 1.8–2.2; pedipalpal femur and genu without ventral extension; colorless; southwestern	**124**
–	Gnathosomal bay unmodified, length/width = 1.4–2.5; pedipalpal tibia more elongate, length/width = 2.7–4.4; pedipalpal femur with ventral extension and genu with or without ventral extension; coloration variable; eastern or western	**125**
124 (123)	Dorsum elongate, length/width = 1.7–1.8; subcapitulum ventral length/width = 2.0–2.1; known only from Catron County, New Mexico	*T. boettgeri*
–	Dorsum ovoid, length/width = 1.3–1.4; subcapitulum ventral length/width = 2.6–2.7; Arizona and Mexico	*T. kurtvietsi*
125 (123)	Pedipalpal femur with flange-like, dentate ventral extension; pedipalpal tibia more elongate, length/width = 4.8–5.2; southwestern	*T. keesdavidsi*
–	Pedipalpal femur with tuberculate ventral extension; pedipalpal tibia less elongate, length/width = 2.7–4.4	**126**
126 (125)	Genital field longer (160–220); western	**127**
–	Genital field shorter (90–155); eastern	**130**
127 (126)	Pedipalpal tibia short and thick, length/width = 2.7–3.0; pedipalpal femur/genu = 1.3–1.5; rostrum less elongate, length/height = 1.63–1.72; Arizona and New Mexico	*T. rala*
–	Pedipalpal tibia unmodified, length/width = 3.3–4.0; pedipalpal femur/genu = 1.6–1.8; rostrum more elongate, length/width = 1.81–2.91	**128**
128 (127)	Dorsum smaller (580–650 long; 415–470 wide); pedipalpal tibia more elongate, length/width = 3.8–4.0; Catron & Grant Counties, New Mexico	*T. sharkeyi* **sp. n.**
–	Dorsum larger (735–770 long; 510–590 wide); pedipalpal tibia less elongate, length/width = 3.3–3.5	**129**
129 (128)	Dorsum round, length/width = 1.25; anterior venter/genital field width = 1.79; pedipalpal tarsus longer (52.5); rostrum more elongate, length/width = 2.4; single specimen from Catron County, New Mexico	*T. dolichodactyla* **sp. n.**
–	Dorsum elongate, length/width = 1.51; anterior venter/genital field width = 2.54; pedipalpal tarsus shorter (20); rostrum less elongate, length/width = 1.8; known only from Mendocino County, California	*T. leviathan* **sp. n.**
130 (126)	Pedipalpal femoral tubercle broad and flat; dorsal coloration bold and reddish-purple; Fayette & Somerset Counties, Pennsylvania	*T. skvarlai* **sp. n.**
–	Pedipalpal femoral tubercle conical; dorsal coloration usually bold and reddish- or bluish-purple	**131**
131 (130)	Anterior venter/genital field width = 1.7–1.9; pedipalpal femur longer (117–123); subcapitulum longer (320–335, ventral length); dorsal coloration reddish purple separated into anterior and posterior portions, but with reddish color of anterio-medial platelets distinctly brighter; known only from Clay County, Alabama	*T. daemon* **sp. n.**
–	Anterior venter/genital field width = 2.0–3.4; pedipalpal femur longer (82–108); subcapitulum shorter (235–300, ventral length); dorsal coloration usually bold and reddish- or bluish-purple and anterio-medial platelets **not** distinctly bright	**Eastern 4-Plates**...**132**
132 (131)	Genital field wider (110–120); dorsum wider (395–430); Appalachians (Tennessee & Pennsylvania)	*T. glomerabilis* **sp. n.**
–	Genital field thinner (75–98); dorsum thinner (260–370)	**133**
133 (132)	Anterior venter longer (277–285); genital field longer (130–138); dorsum wider (350–370); southern Appalachians (Tennessee & Carolinas)	*T. dunni* **sp. n.**
–	Anterior venter shorter (195–250); genital field shorter (90–120); dorsum thinner (260–340)	**134**
134 (133)	Rostrum more elongate, length/width = 3.4–3.6; Lauderdale County, Alabama	*T. pollani* **sp. n.**
–	Rostrum less elongate, length/width = 2.2–3.0	**135**
135 (136)	Anterior venter shorter (195); genital field wider (97.5); pedipalpal tibia more elongate, length/width = 3.59; male known from a single specimen from Morris County, New Jersey	*T. rufoalba*
–	Anterior venter longer (215–238); genital field thinner (75–82.5); pedipalpal tibia less elongate, length/width = 2.8–3.22	**136**
136 (135)	Dorsum smaller (400–465 long; 260–305 wide); genital field shorter (90–108); pedipalpal tibia more elongate, length/width = 3.11–3.22; subcapitulum ventral length/height = 2.67–2.82; Appalachians (Tennessee & Pennsylvania)	*T. shubini* **sp. n.**
–	Dorsum larger (500 long; 340 wide); genital field longer (115); pedipalpal tibia less elongate, length/width = 2.80; subcapitulum ventral length/height = 2.38; male known only from a single specimen from Sussex County, New Jersey	*T. kittinianna*
137 (122). Dorsal plate with longitudinal striations anteriorly; dorsal coloration bold purple separated into anterior and posterior portions, with faint orange medially; southern Appalachians	*T. arktonyx* **sp. n.**
–	Dorsal plate without longitudinal striations anteriorly; dorsal coloration variable	**138**
138 (137)	Subcapitulum ventral length/width = 3.31–4.94; dorsum coloration purple restricted posteriorly, occasionally diffuse throughout dorsal plate or colorless*	**139**
–	Subcapitulum ventral length/width = 1.60–3.22; dorsum coloration variable*	**143**
	* *T. oliveri* (3.10–3.34) key in both directions
139 (138)	Pedipalpal femur/genu = 1.94; pedipalpal tibia shorter (47.5); single specimen from Catron County, New Mexico	*T. anoplopalpa* **sp. n.**
–	Pedipalpal femur/genu = 1.3–1.8; pedipalpal tibia longer (62–93)	**140**
140 (139)	Gnathosomal bay more elongate, length/width = 2.14–2.40; pedipalpal femur/genu = 1.34–1.40; dorsal coloration diffuse purple, not restricted posteriorly; Pacific Ranges	*T. oliveri* **sp. n.**
–	Gnathosomal bay less elongate, length/width = 1.27–2.04; pedipalpal femur/genu = 1.43–1.79; dorsal coloration purple restricted posteriorly or colorless	**141**
141 (142)	Pedipalpal tubercles absent; pedipalpal tibia length/width = 2.7–3.0; anterior venter/genital field width = 3.2–3.7; east of the Mississippi River	*T. projector*
–	Pedipalpal tubercles prominent; pedipalpal tibia length/width = 3.6–5.2; anterior venter/genital field width = 1.9–2.8	**142**
142 (141)	Body smaller (dorsum: 485–510 long; 340–370 wide); anterior venter shorter (247–265); anterio-medial platelets more elongate, length/width = 2.4–2.7; pedipalpal tibia more elongate, length/width = 4.4–4.9; southeastern	*T. flangipalpa* **sp. n.**
–	Body larger (dorsum: 560–650 long; 400–460 wide); anterior venter longer (305–325); anterio-medial platelets less elongate, length/width = 1.7–2.0; pedipalpal tibia less elongate, length/width = 3.6–4.2; western (not southwestern)	*T. tahoei*
143 (138)	Pedipalpal tubercles, especially genual tubercle consisting of a dentate flange; dorsal coloration either purple that is usually restricted posteriorly, or absent; western	**144**
–	Pedipalpal tubercles, when present, conical, sometimes dentate; dorsal coloration variable; eastern or western	**Miniforma Group**...**111**
144 (143)	Pedipalpal tibia elongate, length/width = 4.0–4.2; dorsum wider (445–470); Pacific Ranges	*T. oliveri* **sp. n.**
–	Pedipalpal tibia unmodified, length/width = 2.4–3.5; dorsum thinner (335–400)	**145**
145 (144). Rostrum elongate, length/width = 4.7–4.9; genital field shorter (117–125); Pacific Ranges	*T. pinocchio* **sp. n.**
–	Rostrum unmodified, length/width = 2.5–3.2; genital field longer (130–150)	**146**
146 (145)	Pedipalpal tibia more elongate (3.19–3.38); Catron County, New Mexico	*T. manni* **sp. n.**
–	Pedipalpal tibia less elongate (2.38–2.94)	**147**
147 (146)	Pedipalpal femoral tubercle broader and anteriorly-directed; anterior venter/genital field width = 2.04–2.21; dorsal coloration usually absent, rarely with purple posteriorly; California & Oregon	*T. copipalpa* **sp. n.**
–	Pedipalpal femoral tubercle more tuberculate and not anteriorly-directed; anterior venter/genital field width = 1.82–1.98	**148**
148 (147)	Dorsum shorter (485); rostrum more elongate, length/width = 3.19; pedipalpal tibia less elongate, length/width = 2.38; dorsal coloration absent; female known from single specimen from Prairie Creek in Humboldt County, California	*T. miniforma*
–	Dorsum longer (525–590); rostrum less elongate, length/width = 2.76–3.07; pedipalpal tibia more elongate, length/width = 2.47–2.94; dorsal coloration usually present, purple that is especially prominent posteriorly	**149**
149 (148)	Dorsum thinner (335–350 wide); dorsal coloration purple posteriorly, usually encroaching anteriorly nearly to the platelets, occasionally including the platelets; Rocky Mountains (Idaho & Montana)	*T. rockyensis* **sp. n.**
–	Dorsum thicker (355–390 wide); dorsal coloration purple posteriorly, but usually not encroaching as far anteriorly; Oregon & Washington	*T. pacificensis* **sp. n.**
150 (143)	Dorsum smaller (450–460 long; 265–270 wide) and elongate, length/width = 1.70; dorsal coloration purple and separated into anterior and posterior portions; Tishomingo County, Mississippi	*T. elongata* **sp. n.**
–	Dorsum larger (480–850 long; 295–605 wide) and round or less elongate, length/width = 1.17–1.68; dorsal coloration variable	**151**
151 (150)	Pedipalpal tubercles absent; dorsum colorless, or rarely with pinkish centrally; Wayne County, Indiana	*T. hoosieri* **sp. n.**
–	Pedipalpal tubercles present; dorsal coloration variable	**152**
152 (151)	Eastern (east of the 100^th^ Meridian, but including Texas and Saskatchewan)	**153**
–	Western (west of the 100^th^ Meridian)	**170**
153 (152)	Rostrum short and conical, length/width = 1.80–2.53	**154**
–	Rostrum unmodified, length/width = 2.67–3.5	**161**
154 (153)	Dorsum colorless	**155**
–	Dorsum patterned, usually with dark, bold color	**156**
155 (154)	Dgl-4 closer to edge of dorsum, dorsum width/distance between Dgl-4 = 1.2–1.3; anterior venter/genital field width = 2.9–3.2; pedipalpal tibia more elongate, length/width = 3.1–3.3; lotic (streams); Interior Highlands (Missouri & Arkansas)	*T. pearsoni* **sp. n.**
–	Dgl-4 further from edge of dorsum, dorsum width/distance between Dgl-4 = 1.6–1.7; anterior venter/genital field width = 2.3–2.5; pedipalpal tibia less elongate, length/width = 2.7–2.8; lentic (lakes); known only from Middle Bass Island, Lake Erie, Ottawa County, Ohio	*T. bittikoferae*
156 (154)	Dorsal coloration diagnostic, with three dark spots (two posterior, one anterior); east of the Rocky Mountains	*T. trimaculata*
–	Dorsal coloration not as above	**157**
157 (158)	Dorsal coloration diagnostic, with dark, bold, bell-shaped spot anteriorly and bold red posteriorly (similar only to *T. ululata*, which have anterio-lateral platelets fused to the dorsal plate, and *T. kringi*, which have more elongate rostra, length/width = 2.8–3.0); Arkansas & New Brunswick	*T. unimaculata* **sp. n.**
–	Dorsal coloration not as above;	**158**
158 (157)	Dorsal coloration diagnostic, with dark anterior coloration nearly restricted to anterio-medial platelets, either with or without dark posterior spot, and with dark portions connected by bold red stripe; body elongate, dorsum length/width = 1.53–1.57; Polk & Montgomery Counties, Arkansas	*T. larvata*
–	Dorsal coloration not as above; body more ovoid, dorsum length/width = 1.23–1.44, except *T. cardia* (1.43–1.54)	**159**
159 (158)	Dorsum more elongate, length/width = 1.43–1.54; gnathosomal bay more elongate, length/width = 1.85–1.93; dorsal coloration with dark spot posteriorly connected to a faint spot anteriorly; Appalachians	*T. cardia* **sp. n.**
–	Dorsum less elongate, length/width = 1.23–1.39; gnathosomal bay less elongate, length/width = 1.33–1.82	**160**
160 (159)	Rostrum more elongate, length/width = 2.3–2.5; dorsal coloration variable, but usually distinct, appearing as two large posterior spots and a smaller anterior spot that are all merged together (spot color varies from pinkish to dark), however, occasionally similar to *T. cardia* and *T. mohawk*; Appalachians (Tennessee, South Carolina, Nova Scotia)	*T. tricolor*
–	Rostrum less elongate, length/width = 2.0–2.2; dorsal coloration appearing as an anterior, diffuse, dark stripe, often expanded posteriorly; known only from Aroostook County, Maine	*T. mohawk* **sp. n.**
161 (153)	Pedipalpal tibia more elongate, length/width = 4.00–4.80	**162**
–	Pedipalpal tibia less elongate, length/width = 2.70–3.94	**165**
162 (161)	Rostrum dorsally with patch of strong dentation; subcapitulum more elongate, length/width = 3.5–3.7; femur shorter (90–93); dorsal coloration purple and restricted posteriorly; southern Appalachians	*T. dentirostra* **sp. n.**
–	Rostrum without dentation; subcapitulum less elongate, length/width = 2.5–2.9; femur longer (100–124); dorsal coloration variable, but not restricted posteriorly	**Raptor Group (in part)**...**163**
163 (162)	Pedipalpal tibia shorter (80–83); rostrum length/width = 3.43–3.57; dorsal coloration reddish-purple posteriorly, extending anteriorly to the edge of the dorsal plate; Floyd & Chattooga Counties, Georgia	*T. danielleae* **sp. n.**
–	Pedipalpal tibia longer (87–110); rostrum length/width = 2.81–3.38	164
164 (163)	Subcapitulum dorsal length longer (247–255); pedipalpal tibia/femur = 0.76–0.77; dorsum more ovoid, length/width = 1.31–1.34; dorsal coloration reddish-purple; known only from Clay County, Alabama	*T. daemon* * **sp. n.**
–	Subcapitulum dorsal length shorter (207–240); pedipalpal tibia/femur = 0.81–0.91; dorsum rounder, length/width = 1.26–1.30; dorsal coloration variable, but usually dark, bold bluish-purple; eastern, but not known from south of Kentucky (east of Mississippi River)	*T. irapalpa* * **sp. n.**
	*T. daemon and *T. irapalpa* are best differentiated using female specimens
165 (161)	Pedipalpal femur/genu = 1.3–1.5; dorsum rounder, length/width = 1.19–1.30; dorsal coloration restricted to a single dark spot anteriorly with an orange spot posterior to the dark spot (similar only to *T. unimaculata* and *T. ululata*); known only from Tyler County, Texas	*T. kringi* **sp. n.**
–	Pedipalpal femur/genu = 1.6–1.9; dorsum more ovoid, length/width = 1.32–1.68; dorsal coloration separated into anterior and posterior portions	**166**
166 (165)	Rostrum upturned; rostrum more elongate, length/width = 3.54–3.73; pedipalpal tibia length/width = 2.70–3.11; Appalachians	*T. reduncarostra* **sp. n.**
–	Rostrum straight; rostrum more elongate, length/width = 2.67–3.50; pedipalpal tibia length/width = 3.14–3.94	**167**
167 (166)	Dorsum elongate, dorsal length/width = 1.54–1.58; pedipalpal tibia shorter (62–73); dorsal coloration either reddish-purple separated into anterior and posterior portions, or diagnostic, dark blue with red oval medially; red morph from Appalachians (Tennessee, South Carolina, New Brunswick); dark morph known only from Tellico River in Monroe County, Tennessee	*T. gorti* **sp. n.**
–	Dorsum ovoid, dorsal length/width = 1.32–1.50; pedipalpal tibia longer (77–88); dorsal coloration separated into anterior and posterior portions, bluish or reddish purple sometimes with faint orange medially; eastern	**168**
168 (169)	Rostrum more elongate, length/width = 3.00–3.13; medial suture (55–70); known only from Haywood County, North Carolina	*T. bondi* **sp. n.**
–	Rostrum less elongate, length/width = 2.6–2.9; medial suture (75–108); eastern	**Neoanomala Group**...**169**
169 (168)	Anterior venter shorter (220–240); genital field shorter (132–138); anterio-medial platelet length/width = 2.45–2.72; Montgomery & Newton Counties, Arkansas	*T. interiorensis* **sp. n.**
–	Anterior venter longer (267.5–290); genital field longer (145–160); anterio-medial platelet length/width = 2.08–2.46; eastern	*T. neoanomala* **sp. n.**
170 (152)	Rostrum less elongate, length/width = 1.8–2.2	**171**
–	Rostrum more elongate, length/width = 2.5–3.2	**173**
171 (170)	Subcapitulum more elongate, ventral length/width = 2.2–2.5; dorsum rounder, length/width = 1.17–1.28; dorsal coloration usually orangish and separated into anterior and posterior portions, occasionally very faint to colorless, rarely darker	*T. sierrensis*
–	Subcapitulum less elongate, ventral length/width = 1.6–2.0; dorsum more ovoid, length/width = 1.33–1.67	**172**
172 (171)	Dorsum longer (725–850); pedipalpal femur longer (92.5–100); rostrum more elongate, length/width = 1.86–2.02; dorsum colorless or nearly so; widespread in the west	*T. ellipsoidalis*
–	Dorsum shorter (560–640); pedipalpal femur shorter (75–78); rostrum short, length/width = 1.56–1.81; dorsum colorless, or with faint coloration separated into anterior and posterior portions; British Columbia, Oregon, & California	*T. olliei* **sp. n.**
173 (172)	Pedipalpal tibia less elongate, length/width = 2.9–3.5	**174**
–	Pedipalpal tibia more elongate, length/width = 3.7–4.8	**176**
174 (173)	Dorsum longer (665–790); subcapitulum less elongate, ventral length/width = 2.1–2.4; dorsal coloration orangish-pinkish; widespread throughout the west	*T. multiforma*
–	Dorsum shorter (540–640); subcapitulum more elongate, ventral length/width = 2.5–3.0; California & Oregon	**175**
175 (174)	Hind coxal margins distinct and complete; dorsum more ovoid, length/width = 1.4–1.6; anterior venter/ genital field length = 2.0–2.3; subcapitulum ventral length shorter (300–335); dorsal coloration bluish-purple divided into anterior and posterior portions, often faint, and connected by red band; California & southwestern Oregon	*T. ventura*
–	Hind coxal margins indistinct or incomplete; dorsum rounder, length/width = 1.2–1.3; anterior venter/ genital field length = 1.2–1.5; subcapitulum ventral length longer (355–375); dorsal coloration bright purple; known only from Curry County, Oregon	*T. regalis* **sp. n.**
176 (173)	Dorsum longer (690–820); anterior venter/medial suture = 2.7–2.8; rostrum less elongate, length/width = 2.5–2.8; dorsal coloration with faint purple restricted posteriorly; Oregon	*T. oregonensis* **sp. n.**
–	Dorsum shorter (530–610); anterior venter/medial suture = 3.0–3.7; rostrum more elongate, length/width = 2.9–3.6; southwest	**177**
177 (176)	Pedipalpal tibia less elongate, length/width = 3.7–4.0; rostrum more elongate, length/width = 3.3–3.6; Dgl-4 further from edge of dorsum, dorsum width/distance between Dgl-4 = 1.54–1.67; dorsum colorless; southern California	*T. wiedenmanni* **sp. n.**
–	Pedipalpal tibia more elongate, length/width = 4.2–4.8; rostrum less elongate, length/width = 2.9–3.1; Dgl-4 closer to edge of dorsum, dorsum width/distance between Dgl-4 = 1.34–1.43; dorsal coloration bluish purple, restricted posteriorly; Arizona & New Mexico	*T. raptoroides* **sp. n.**


## Supplementary Material

XML Treatment for Torrenticola anoplopalpa

XML Treatment for Torrenticola arktonyx

XML Treatment for Torrenticola biscutella

XML Treatment for Torrenticola bittikoferae

XML Treatment for Torrenticola boettgeri

XML Treatment for Torrenticola bondi

XML Treatment for Torrenticola caerulea

XML Treatment for Torrenticola cardia

XML Treatment for Torrenticola copipalpa

XML Treatment for Torrenticola daemon

XML Treatment for Torrenticola danielleae

XML Treatment for Torrenticola delicatexa

XML Treatment for Torrenticola dentirostra

XML Treatment for Torrenticola dimorpha

XML Treatment for Torrenticola dolichodactyla

XML Treatment for Torrenticola dunni

XML Treatment for Torrenticola ellipsoidalis

XML Treatment for Torrenticola elongata

XML Treatment for Torrenticola elusiva

XML Treatment for Torrenticola erectirostra

XML Treatment for Torrenticola feminellai

XML Treatment for Torrenticola flangipalpa

XML Treatment for Torrenticola folkertsae

XML Treatment for Torrenticola glomerabilis

XML Treatment for Torrenticola gnoma

XML Treatment for Torrenticola gorti

XML Treatment for Torrenticola hoosieri

XML Treatment for Torrenticola indistincta

XML Treatment for Torrenticola interiorensis

XML Treatment for Torrenticola irapalpa

XML Treatment for Torrenticola ivyae

XML Treatment for Torrenticola karambita

XML Treatment for Torrenticola keesdavidsi

XML Treatment for Torrenticola kittatinniana

XML Treatment for Torrenticola kringi

XML Treatment for Torrenticola kurtvietsi

XML Treatment for Torrenticola lamellipalpis

XML Treatment for Torrenticola larvata

XML Treatment for Torrenticola leviathan

XML Treatment for Torrenticola longitibia

XML Treatment for Torrenticola magnexa

XML Treatment for Torrenticola malarkeyorum

XML Treatment for Torrenticola manni

XML Treatment for Torrenticola microbiscutella

XML Treatment for Torrenticola miniforma

XML Treatment for Torrenticola mjolniri

XML Treatment for Torrenticola mohawk

XML Treatment for Torrenticola mulleni

XML Treatment for Torrenticola multiforma

XML Treatment for Torrenticola neoanomala

XML Treatment for Torrenticola nigroalba

XML Treatment for Torrenticola nortoni

XML Treatment for Torrenticola occidentalis

XML Treatment for Torrenticola oliveri

XML Treatment for Torrenticola olliei

XML Treatment for Torrenticola oregonensis

XML Treatment for Torrenticola pacificensis

XML Treatment for Torrenticola pearsoni

XML Treatment for Torrenticola pendula

XML Treatment for Torrenticola pinocchio

XML Treatment for Torrenticola pollani

XML Treatment for Torrenticola priapus

XML Treatment for Torrenticola projector

XML Treatment for Torrenticola pulchra

XML Treatment for Torrenticola racupalpa

XML Treatment for Torrenticola rala

XML Treatment for Torrenticola raptor

XML Treatment for Torrenticola raptoroides

XML Treatment for Torrenticola reduncarostra

XML Treatment for Torrenticola regalis

XML Treatment for Torrenticola robisoni

XML Treatment for Torrenticola rockyensis

XML Treatment for Torrenticola rufoalba

XML Treatment for Torrenticola sellersorum

XML Treatment for Torrenticola sharkeyi

XML Treatment for Torrenticola shubini

XML Treatment for Torrenticola sierrensis

XML Treatment for Torrenticola skvarlai

XML Treatment for Torrenticola solisorta

XML Treatment for Torrenticola tahoei

XML Treatment for Torrenticola tricolor

XML Treatment for Torrenticola trimaculata

XML Treatment for Torrenticola tysoni

XML Treatment for Torrenticola ululata

XML Treatment for Torrenticola unimaculata

XML Treatment for Torrenticola ventura

XML Treatment for Torrenticola walteri

XML Treatment for Torrenticola welbourni

XML Treatment for Torrenticola whitneyae

XML Treatment for Torrenticola wiedenmanni

## Supplementary materials

Supplementary material 1 Deposited sequences in GenBank Data type: molecular data This dataset is made available under the Open Database License (http://opendatacommons.org/licenses/odbl/1.0/). The Open Database License (ODbL) is a license agreement intended to allow users to freely share, modify, and use this Dataset while maintaining this same freedom for others, provided that the original source and author(s) are credited.J. Ray Fisher, Danielle M. Fisher, Michael J. Skvarla, Whitney A. Nelson, Ashley P.G. Dowling
