# Supplementary material for: Revision of torrent mites (Parasitengona, Torrenticolidae, Torrenticola) of the United States and Canada: 90 descriptions, molecular phylogenetics, and a key to species
Source: Zookeys. 2017 Sep 21;(701):1–496. doi: 10.3897/zookeys.701.13261 (PMC5674090; doi:10.3897/zookeys.701.13261)
Supplement: Supplementary material 1 — Deposited sequences in GenBank [file zookeys-701-001-s001.pdf]

Species	Genbank Accession #		DNA Number & Specimen Catalog Number	GenSeq Nomenclature
	COI	28S		
T. biscutella	KX065468		DNA1262, ACUA135827 (paratype)	genseq-2 COI
T. biscutella	KX065469		DNA1263, ACUA135828 (holotype)	genseq-1 COI
T. biscutella	KX065474	KX078611	DNA1275, ACUA135851 (paratype)	genseq-2 COI, 28S
T. bondi	KX065501	KX078611	DNA1431, ACUA143160 (holotype)	genseq-1 COI, 28S
T. caerulea	KX065720	KX078627	DNA1882, ACUA139945 (holotype)	genseq-1 COI, 28S
T. caerulea	KX065721	KX078628	DNA1883, ACUA139946 (paratype)	genseq-2 COI, 28S
T. copipalpa	KX065596		DNA1614, ACUA138898 (paratype)	genseq-2 COI
T. copipalpa	KX065597		DNA1615, ACUA138899 (paratype)	genseq-2 COI, 28S
T. copipalpa	KX065604		DNA1626, ACUA138925 (paratype)	genseq-2 COI
T. copipalpa	KX065644		DNA1720, ACUA140802 (paratype)	genseq-2 COI
T. copipalpa	KX065645		DNA1721, ACUA140803 (paratype)	genseq-2 COI
T. copipalpa	KX065722		DNA1901, ACUA139954 (paratype)	genseq-2 COI
T. copipalpa	KX065738		DNA1931, ACUA139982 (paratype)	genseq-2 COI
T. copipalpa	KX065746		DNA1941, ACUA140715 (paratype)	genseq-2 COI
T. copipalpa	KX065753		DNA1950, ACUA140722 (paratype)	genseq-2 COI
T. copipalpa	KX065757		DNA1958, ACUA140718 (paratype)	genseq-2 COI
T. delicatexa	KX065485		DNA1292, ACUA135816 (non-type voucher)	genseq-4 COI
T. delicatexa	KX065671	KX078622	DNA1813, ACUA139901 (non-type voucher)	genseq-4 COI, 28S
T. delicatexa	KX065687		DNA1839, ACUA139907 (non-type voucher)	genseq-4 COI
T. delicatexa	KX065688		DNA1841, ACUA139909 (non-type voucher)	genseq-4 COI
T. delicatexa	KX065689		DNA1842, ACUA139910 (non-type voucher)	genseq-4 COI
T. delicatexa	KX065690		DNA1843, ACUA139911 (non-type voucher)	genseq-4 COI
T. delicatexa	KX065691		DNA1844, ACUA139912 (non-type voucher)	genseq-4 COI
T. delicatexa	KX065705		DNA1865, ACUA139928 (non-type voucher)	genseq-4 COI
T. delicatexa	KX065769		DNA1981, ACUA138968 (non-type voucher)	genseq-4 COI
T. delicatexa	KX065770		DNA1982, ACUA138978 (non-type voucher)	genseq-4 COI
T. delicatexa	KX065817		DNA2828, ACUA148519 (non-type voucher)	genseq-4 COI
T. delicatexa	KX065818		DNA2830, ACUA148520 (non-type voucher)	genseq-4 COI
T. delicatexa	KX065825		DNA2846, ACUA148526 (non-type voucher)	genseq-4 COI
T. dimorpha			DNA3345, ACUA151211 (paratype)	genseq-2 COI
T. dimorpha			DNA3346, ACUA151210 (paratype)	genseq-2 COI, 28S
T. dimorpha			DNA3351, ACUA151216 (holotype)	genseq-1 COI
T. dimorpha			DNA3352, ACUA151215 (paratype)	genseq-2 COI
T. dimorpha			DNA3353, ACUA151217 (paratype)	genseq-2 COI
T. dunni	KX065482		DNA1289, ACUA135818 (holotype)	genseq-1 COI
T. dunni	KX065484		DNA1291, ACUA135815 (paratype)	genseq-2 COI
T. dunni	KX065649		DNA1729, ACUA143026 (paratype)	genseq-2 COI
T. dunni	KX065652		DNA1734, ACUA138960 (paratype)	genseq-2 COI
T. dunni	KX065653	KX078619	DNA1735, ACUA138959 (paratype)	genseq-2 COI, 28S
T. dunni	KX065670		DNA1812, ACUA139900 (paratype)	genseq-2 COI
T. dunni	KX065672		DNA1814, ACUA139902 (paratype)	genseq-2 COI
T. dunni	KX065685		DNA1837, ACUA139905 (paratype)	genseq-2 COI
T. dunni	KX065701		DNA1861, ACUA139925 (paratype)	genseq-2 COI
T. dunni	KX065702		DNA1862, ACUA138992 (paratype)	genseq-2 COI
T. ellipsoidalis	KX065585		DNA1603, ACUA138904 (non-type voucher)	genseq-4 COI
T. ellipsoidalis	KX065586		DNA1604, ACUA138910 (non-type voucher)	genseq-4 COI
T. ellipsoidalis	KX065587		DNA1605, ACUA138896 (non-type voucher)	genseq-4 COI
T. ellipsoidalis	KX065589	KX078595	DNA1607, ACUA138913 (non-type voucher)	genseq-4 COI, 28S
T. ellipsoidalis	KX065590		DNA1608, ACUA138914 (non-type voucher)	genseq-4 COI
T. ellipsoidalis	KX065614		DNA1643, ACUA138936 (non-type voucher)	genseq-4 COI
T. ellipsoidalis	KX065632		DNA1708, ACUA147065 (non-type voucher)	genseq-4 COI
T. ellipsoidalis	KX065634		DNA1710, ACUA140750 (non-type voucher)	genseq-4 COI
T. ellipsoidalis	KX065635		DNA1711, ACUA140800 (non-type voucher)	genseq-4 COI
T. ellipsoidalis	KX065636		DNA1712, ACUA140801 (non-type voucher)	genseq-4 COI
T. ellipsoidalis	KX065723		DNA1908, ACUA139962 (non-type voucher)	genseq-4 COI

T. ellipsoidalis	KX065732		DNA1925, ACUA139977 (non-type voucher)	genseq-4 COI
T. ellipsoidalis	KX065737		DNA1930, ACUA139981 (non-type voucher)	genseq-4 COI
T. ellipsoidalis	KX065805		DNA2636, ACUA148000 (non-type voucher)	genseq-4 COI
T. ellipsoidalis	KX065806		DNA2637, ACUA148001 (non-type voucher)	genseq-4 COI
T. ellipsoidalis	KX065807		DNA2640, ACUA148002 (non-type voucher)	genseq-4 COI
T. ellipsoidalis	KX065808		DNA2641, ACUA148003 (non-type voucher)	genseq-4 COI
T. ellipsoidalis	KX065878		DNA2919, ACUA148582 (non-type voucher)	genseq-4 COI
T. ellipsoidalis	KX065879		DNA2920, ACUA148583 (non-type voucher)	genseq-4 COI
T. ellipsoidalis	KX065880		DNA2921, ACUA148584 (non-type voucher)	genseq-4 COI
T. ellipsoidalis	KX065882		DNA2927, ACUA148586 (non-type voucher)	genseq-4 COI
T. ellipsoidalis	KX065891		DNA2940, ACUA148595 (non-type voucher)	genseq-4 COI
T. ellipsoidalis	KX065892		DNA2941, ACUA148596 (non-type voucher)	genseq-4 COI
T. ellipsoidalis	KX065893		DNA2942, ACUA148597 (non-type voucher)	genseq-4 COI
T. elongata	KX065575		DNA1591, ACUA138879 (paratype)	genseq-2 COI
T. elongata	KX065576	KX078590	DNA1593, ACUA138965 (paratype)	genseq-2 COI, 28S
T. elusiva	KX065698	KX078606	DNA1857, ACUA138994 (holotype)	genseq-1 COI, 28S
T. erectirostra	KX065909		DNA2962, ACUA148663 (holotype)	genseq-1 COI
T. erectirostra	KX065910		DNA2963, ACUA148665 (paratype)	genseq-2 COI
T. erectirostra	KX065911		DNA2964, ACUA148664 (paratype)	genseq-2 COI
T. erectirostra	KX065912	KX078600	DNA2966, ACUA148666 (paratype)	genseq-2 COI, 28S
T. flangipalpa	KX065494		DNA1310, ACUA143120 (holotype)	genseq-1 COI
T. flangipalpa	KX065559		DNA1567, ACUA138868 (paratype)	genseq-2 COI
T. flangipalpa	KX065560		DNA1568, ACUA138867 (paratype)	genseq-2 COI
T. flangipalpa	KX065679	KX078596	DNA1822, ACUA139877 (paratype)	genseq-2 COI, 28S
T. flangipalpa	KX065680		DNA1823, ACUA139878 (paratype)	genseq-2 COI
T. flangipalpa	KX065681		DNA1824, ACUA139879 (paratype)	genseq-2 COI
T. glomerabilis	KX065558		DNA1565, ACUA138872 (paratype)	genseq-2 COI
T. glomerabilis	KX065666	KX078621	DNA1756, ACUA139897 (paratype)	genseq-2 COI, 28S
T. glomerabilis	KX065667		DNA1757, ACUA139898 (paratype)	genseq-2 COI
T. glomerabilis	KX065765		DNA1971, ACUA138970 (paratype)	genseq-2 COI
T. gnoma	KX065718		DNA1880, ACUA139943 (paratype)	genseq-2 COI
T. gnoma	KX065719		DNA1881, ACUA139944 (paratype)	genseq-2 COI
T. gnoma	KX065771	KX078630	DNA1983, ACUA138984 (paratype)	genseq-2 COI, 28S
T. gnoma	KX065772		DNA1984, ACUA138985 (paratype)	genseq-2 COI
T. gnoma	KX065773		DNA1985, ACUA138973 (paratype)	genseq-2 COI
T. gorti	KX065674		DNA1816, ACUA139871 (paratype)	genseq-2 COI
T. gorti	KX065676		DNA1819, ACUA139874 (paratype)	genseq-2 COI
T. gorti	KX065677	KX078623	DNA1820, ACUA139875 (paratype)	genseq-2 COI, 28S
T. gorti	KX065678		DNA1821, ACUA139876 (paratype)	genseq-2 COI
T. gorti	KX065913		DNA2970, ACUA148673 (holotype)	genseq-1 COI
T. gorti	KX065914		DNA2972, ACUA148674 (paratype)	genseq-2 COI
T. gorti	KX065915		DNA2974, ACUA148671 (paratype)	genseq-2 COI
T. hoosieri	KX065762		DNA1966, ACUA138989 (paratype)	genseq-2 COI, 28S
T. interiorensis	KX065551		DNA1556, ACUA148628 (paratype)	genseq-2 COI
T. interiorensis	KX065554	KX078603	DNA1561, ACUA138876 (paratype)	genseq-2 COI, 28S
T. interiorensis	KX065555		DNA1562, ACUA140740 (paratype)	genseq-2 COI
T. interiorensis	KX065552		DNA1557, ACUA140709 (paratype)	genseq-2 COI
T. irapalpa	KX065471		DNA1269, (non-type voucher)	genseq-4 COI
T. irapalpa	KX065477		DNA1279, ACUA135841 (paratype)	genseq-2 COI
T. irapalpa	KX065500		DNA1430, ACUA138997 (paratype)	genseq-2 COI
T. irapalpa	KX065515		DNA1447, ACUA138318 (paratype)	genseq-2 COI
T. irapalpa	KX065544		DNA1546, ACUA140713 (paratype)	genseq-2 COI
T. irapalpa	KX065545		DNA1549, ACUA148609 (paratype)	genseq-2 COI
T. irapalpa	KX065546		DNA1551, ACUA138340 (paratype)	genseq-2 COI
T. irapalpa	KX065547		DNA1552, ACUA139000 (paratype)	genseq-2 COI
T. irapalpa	KX065548		DNA1553, ACUA138878 (paratype)	genseq-2 COI
T. irapalpa	KX065553		DNA1558, ACUA139001 (paratype)	genseq-2 COI

T. irapalpa	KX065776		DNA1988, ACUA138971 (paratype)	genseq-2 COI
T. irapalpa	KX065905		DNA2956, ACUA148657 (holotype)	genseq-1 COI
T. irapalpa	KX065906		DNA2957, ACUA148656 (paratype)	genseq-2 COI
T. irapalpa	KX065907	KX078605	DNA2959, ACUA148660 (paratype)	genseq-2 COI, 28S
T. irapalpa	KX065908		DNA2961, ACUA148659 (paratype)	genseq-2 COI
T. karambita	KX065668		DNA1758, ACUA138937 (holotype)	genseq-1 COI
T. karambita	KX065692	KX078597	DNA1846, ACUA139914 (paratype)	genseq-2 COI, 28S
T. larvata	KX065510	KX078604	DNA1441, ACUA138336 (paratype)	genseq-2 COI, 28S
T. larvata	KX065528		DNA1460, ACUA138315 (paratype)	genseq-2 COI
T. longitibia	KX065918		DNA2979, ACUA148655 (holotype)	genseq-1 COI, 28S
T. magnexa	KX065483		DNA1290, ACUA135814 (non-type voucher)	genseq-4 COI
T. magnexa	KX065513		DNA1444, ACUA138321 (non-type voucher)	genseq-4 COI
T. magnexa	KX065656		DNA1738, ACUA142986 (non-type voucher)	genseq-4 COI
T. magnexa	KX065686		DNA1838, ACUA139904 (non-type voucher)	genseq-4 COI
T. magnexa	KX065703		DNA1863, ACUA139926 (non-type voucher)	genseq-4 COI
T. magnexa	KX065704		DNA1864, ACUA139927 (non-type voucher)	genseq-4 COI
T. magnexa	KX065708	KX078626	DNA1870, ACUA139933 (non-type voucher)	genseq-4 COI, 28S
T. magnexa	KX065709		DNA1871, ACUA139934 (non-type voucher)	genseq-4 COI
T. magnexa	KX065766		DNA1972, ACUA138974 (non-type voucher)	genseq-4 COI
T. magnexa	KX065826		DNA2848, ACUA148528 (non-type voucher)	genseq-4 COI
T. magnexa	KX065871		DNA2912, ACUA148574 (non-type voucher)	genseq-4 COI
T. magnexa	KX065872		DNA2913, ACUA148575 (non-type voucher)	genseq-4 COI
T. magnexa	KX065873		DNA2914, ACUA148576 (non-type voucher)	genseq-4 COI
T. magnexa	KX065874		DNA2915, ACUA148577 (non-type voucher)	genseq-4 COI
T. malarkeyorum	KX065475		DNA1277, ACUA135844 (paratype)	genseq-2 COI
T. malarkeyorum	KX065476		DNA1278, ACUA135845 (paratype)	genseq-2 COI
T. malarkeyorum	KX065664	KX078620	DNA1749, ACUA139894 (paratype)	genseq-2 COI, 28S
T. malarkeyorum	KX065693		DNA1849, ACUA139917 (paratype)	genseq-2 COI
T. malarkeyorum	KX065715		DNA1877, ACUA139940 (paratype)	genseq-2 COI
T. malarkeyorum	KX065716		DNA1878, ACUA139941 (paratype)	genseq-2 COI
T. malarkeyorum	KX065779		DNA2119, ACUA147043 (paratype)	genseq-2 COI
T. malarkeyorum	KX065780		DNA2120, ACUA147044 (holotype)	genseq-1 COI
T. manni	KX065864		DNA2904, ACUA148567 (paratype)	genseq-2 COI, 28S
T. manni	KX065865		DNA2906, ACUA148568 (holotype)	genseq-1 COI
T. manni	KX065866		DNA2907, ACUA148569 (paratype)	genseq-2 COI
T. mjolniri	KX065665	KX078594	DNA1753, ACUA139895 (paratype)	genseq-2 COI, 28S
T. mjolniri	KX065714		DNA1876, ACUA139939 (paratype)	genseq-2 COI
T. mjolniri	KX065827		DNA2849, ACUA148529 (paratype)	genseq-2 COI
T. mjolniri	KX065828		DNA2850, ACUA148530 (paratype)	genseq-2 COI
T. mjolniri	KX065829		DNA2851, ACUA148531 (paratype)	genseq-2 COI
T. mjolniri	KX065830		DNA2852, ACUA148532 (paratype)	genseq-2 COI
T. mjolniri	KX065831		DNA2853, ACUA148533 (paratype)	genseq-2 COI
T. mjolniri	KX065832		DNA2855, ACUA148534 (paratype)	genseq-2 COI
T. mjolniri	KX065833		DNA2857, ACUA148535 (paratype)	genseq-2 COI
T. mjolniri	KX065834		DNA2858, ACUA148536 (paratype)	genseq-2 COI
T. mjolniri	KX065835		DNA2859, ACUA148537 (paratype)	genseq-2 COI
T. mjolniri	KX065836		DNA2860, ACUA148538 (holotype)	genseq-1 COI
T. mulleni	KX065789		DNA2603, ACUA148588 (paratype)	genseq-2 COI
T. mulleni	KX065790		DNA2604, ACUA147983 (paratype)	genseq-2 COI
T. mulleni	KX065791		DNA2606, ACUA147984 (paratype)	genseq-2 COI
T. mulleni	KX065792		DNA2609, ACUA147985 (paratype)	genseq-2 COI
T. mulleni	KX065793		DNA2610, ACUA147986 (paratype)	genseq-2 COI
T. mulleni	KX065794		DNA2611, ACUA147987 (paratype)	genseq-2 COI
T. mulleni	KX065881		DNA2922, ACUA148585 (paratype)	genseq-2 COI, 28S
T. mulleni	KX065883		DNA2928, ACUA148587 (holotype)	genseq-1 COI
T. mulleni	KX065884		DNA2929, ACUA148588 (paratype)	genseq-2 COI
T. mulleni	KX065885		DNA2930, ACUA148589 (paratype)	genseq-2 COI

T. multiforma	KX065582		DNA1600, ACUA138915 (non-type voucher)	genseq-4 COI
T. multiforma	KX065584		DNA1602, ACUA138906 (non-type voucher)	genseq-4 COI
T. multiforma	KX065602		DNA1622, ACUA138921 (non-type voucher)	genseq-4 COI
T. multiforma	KX065603		DNA1623, ACUA138922 (non-type voucher)	genseq-4 COI
T. multiforma	KX065605		DNA1627, ACUA138923 (non-type voucher)	genseq-4 COI
T. multiforma	KX065610		DNA1639, ACUA138942 (non-type voucher)	genseq-4 COI
T. multiforma	KX065615		DNA1644, ACUA138935 (non-type voucher)	genseq-4 COI
T. multiforma	KX065630		DNA1706, ACUA138909 (non-type voucher)	genseq-4 COI
T. multiforma	KX065631		DNA1707, ACUA138916 (non-type voucher)	genseq-4 COI
T. multiforma	KX065648		DNA1724, ACUA138944 (non-type voucher)	genseq-4 COI
T. multiforma	KX065733		DNA1926, ACUA139972 (non-type voucher)	genseq-4 COI
T. multiforma	KX065734		DNA1927, ACUA139973 (non-type voucher)	genseq-4 COI
T. multiforma	KX065735		DNA1928, ACUA139979 (non-type voucher)	genseq-4 COI
T. multiforma	KX065736		DNA1929, ACUA139980 (non-type voucher)	genseq-4 COI
T. multiforma	KX065742	KX078599	DNA1935, ACUA139987 (non-type voucher)	genseq-4 COI, 28S
T. multiforma	KX065743		DNA1936, ACUA139983 (non-type voucher)	genseq-4 COI
T. multiforma	KX065755		DNA1952, ACUA140732 (non-type voucher)	genseq-4 COI
T. multiforma	KX065760		DNA1962, ACUA140730 (non-type voucher)	genseq-4 COI
T. multiforma	KX065783		DNA2590, ACUA147976 (non-type voucher)	genseq-4 COI
T. multiforma	KX065784		DNA2593, ACUA147977 (non-type voucher)	genseq-4 COI
T. multiforma	KX065785		DNA2595, ACUA147978 (non-type voucher)	genseq-4 COI
T. multiforma	KX065786		DNA2596, ACUA147979 (non-type voucher)	genseq-4 COI
T. multiforma	KX065787		DNA2598, ACUA147980 (non-type voucher)	genseq-4 COI
T. multiforma	KX065788		DNA2601, ACUA147981 (non-type voucher)	genseq-4 COI
T. multiforma	KX065799		DNA2621, ACUA147992 (non-type voucher)	genseq-4 COI
T. multiforma	KX065800		DNA2622, ACUA147993(non-type voucher)	genseq-4 COI
T. multiforma	KX065867		DNA2908, ACUA148570 (non-type voucher)	genseq-4 COI
T. multiforma	KX065868		DNA2909, ACUA148571 (non-type voucher)	genseq-4 COI
T. multiforma	KX065869		DNA2910, ACUA148572 (non-type voucher)	genseq-4 COI
T. multiforma	KX065870		DNA2911, ACUA148573 (non-type voucher)	genseq-4 COI
T. multiforma	KX065875		DNA2916, ACUA148578 (non-type voucher)	genseq-4 COI
T. multiforma	KX065876		DNA2917, ACUA148579 (non-type voucher)	genseq-4 COI
T. multiforma	KX065877		DNA2918, ACUA148580 (non-type voucher)	genseq-4 COI
T. multiforma	KX065886		DNA2932, ACUA148590 (non-type voucher)	genseq-4 COI
T. multiforma	KX065887		DNA2933, ACUA148591 (non-type voucher)	genseq-4 COI
T. multiforma	KX065894		DNA2943, ACUA148598 (non-type voucher)	genseq-4 COI
T. multiforma	KX065895		DNA2944, ACUA148599 (non-type voucher)	genseq-4 COI
T. multiforma	KX065896		DNA2945, ACUA148600 (non-type voucher)	genseq-4 COI
T. neoanomala	KX065473		DNA1272, ACUA135849 (non-type voucher)	genseq-4 COI
T. neoanomala	KX065557		DNA1564, ACUA138995 (non-type voucher)	genseq-4 COI
T. neoanomala	KX065572		DNA1588, ACUA140711 (non-type voucher)	genseq-4 COI
T. neoanomala	KX065658	KX078593	DNA1743, ACUA138946 (non-type voucher)	genseq-4 COI, 28S
T. neoanomala	KX065661		DNA1746, ACUA138947 (non-type voucher)	genseq-4 COI
T. neoanomala	KX065663		DNA1748, ACUA139893 (non-type voucher)	genseq-4 COI
T. neoanomala	KX065710		DNA1872, ACUA139935 (non-type voucher)	genseq-4 COI
T. neoanomala	KX065717		DNA1879, ACUA139942 (non-type voucher)	genseq-4 COI
T. neoanomala	KX065767		DNA1974, ACUA138977 (non-type voucher)	genseq-4 COI
T. neoanomala	KX065848		DNA2877, ACUA148550 (non-type voucher)	genseq-4 COI
T. nigroalba	KX065486		DNA1294, ACUA143148 (non-type voucher)	genseq-4 COI
T. nigroalba	KX065487	KX078587	DNA1295, ACUA143136 (non-type voucher)	genseq-4 COI, 28S
T. nigroalba	KX065492		DNA1305, ACUA143134 (non-type voucher)	genseq-4 COI
T. nigroalba	KX065493		DNA1308, ACUA143131 (non-type voucher)	genseq-4 COI
T. nigroalba	KX065496		DNA1426, ACUA138303 (non-type voucher)	genseq-4 COI
T. nigroalba	KX065502		DNA1432 (non-type voucher)	genseq-4 COI
T. nigroalba	KX065522		DNA1454 (non-type voucher)	genseq-4 COI
T. nigroalba	KX065514		DNA1445, ACUA138320 (non-type voucher)	genseq-4 COI
T. nigroalba	KX065781		DNA2121, ACUA147047 (non-type voucher)	genseq-4 COI

T. nigroalba	KX065782		DNA2122, ACUA147048 (non-type voucher)	genseq-4 COI
T. nigroalba	KX065851		DNA2884, ACUA148553 (non-type voucher)	genseq-4 COI
T. nortoni	KX065725		DNA1914, ACUA139965 (paratype)	genseq-2 COI
T. nortoni	KX065730	KX078629	DNA1921, ACUA139974 (paratype)	genseq-2 COI, 28S
T. nortoni	KX065919		DNA2981, ACUA148694 (paratype)	genseq-2 COI
T. nortoni	KX065920		DNA2985, ACUA148677 (paratype)	genseq-2 COI
T. nortoni	KX065921		DNA2988, ACUA148678 (paratype)	genseq-2 COI
T. nortoni	KX065922		DNA2989, ACUA148680 (paratype)	genseq-2 COI
T. nortoni	KX065923		DNA2990, ACUA148679 (paratype)	genseq-2 COI
T. nortoni	KX065924		DNA2994, ACUA148686 (paratype)	genseq-2 COI
T. nortoni	KX065925		DNA2995, ACUA148687 (paratype)	genseq-2 COI
T. nortoni	KX065926		DNA2996, ACUA148688 (holotype)	genseq-1 COI
T. nortoni	KX065927		DNA2999, ACUA148689 (paratype)	genseq-2 COI
T. nortoni	KX065928		DNA3000, ACUA148690 (paratype)	genseq-2 COI
T. olliei	KX065647	KX078618	DNA1723, ACUA140806 (paratype)	genseq-2 COI, 28S
T. pacificensis	KX065580		DNA1597, ACUA147055 (paratype)	genseq-2 COI
T. pacificensis	KX065581	KX078591	DNA1599, ACUA138895 (paratype)	genseq-2 COI, 28S
T. pacificensis	KX065583		DNA1601, ACUA138905 (paratype)	genseq-2 COI
T. pacificensis	KX065626		DNA1702, ACUA140798 (paratype)	genseq-2 COI
T. pacificensis	KX065627		DNA1703, ACUA140749 (paratype)	genseq-2 COI
T. pacificensis	KX065628		DNA1704, ACUA147056 (paratype)	genseq-2 COI
T. pacificensis	KX065629		DNA1705, ACUA140799 (paratype)	genseq-2 COI
T. pacificensis	KX065633		DNA1709, ACUA147064 (paratype)	genseq-2 COI
T. pearsoni	KX065472		DNA1271, ACUA135848 (paratype)	genseq-2 COI
T. pearsoni	KX065499		DNA1429, ACUA141188 (paratype)	genseq-2 COI
T. pearsoni	KX065549		DNA1554, ACUA140712 (paratype)	genseq-2 COI
T. pearsoni	KX065550	KX078612	DNA1555, ACUA140710 (paratype)	genseq-2 COI, 28S
T. pendula	KX065846		DNA2873, ACUA148548 (holotype)	genseq-1 COI
T. pendula	KX065847		DNA2874, ACUA148549 (paratype)	genseq-2 COI, 28S
T. pollani	KX065481		DNA1288, ACUA135813 (holotype)	genseq-1 COI
T. pollani	KX065561		DNA1569, ACUA138869 (paratype)	genseq-2 COI
T. pollani	KX065564	KX078613	DNA1576, ACUA138870 (paratype)	genseq-2 COI, 28S
T. pollani	KX065711		DNA1873, ACUA139936 (paratype)	genseq-2 COI
T. pollani	KX065712		DNA1874, ACUA139937 (paratype)	genseq-2 COI
T. pollani	KX065713		DNA1875, ACUA139938 (paratype)	genseq-2 COI
T. projector	KX065495		DNA1340, ACUA135541 (non-type voucher)	genseq-4 COI
T. projector	KX065497		DNA1427, ACUA138304 (non-type voucher)	genseq-4 COI
T. projector	KX065498		DNA1428, ACUA138305 (non-type voucher)	genseq-4 COI
T. projector	KX065573	KX078615	DNA1589, ACUA138886 (non-type voucher)	genseq-4 COI, 28S
T. projector	KX065574		DNA1590, ACUA138887 (non-type voucher)	genseq-4 COI
T. projector	KX065669		DNA1759, ACUA138972 (non-type voucher)	genseq-4 COI
T. projector	KX065763		DNA1967, ACUA138988 (non-type voucher)	genseq-4 COI
T. racupalpa	KX065706		DNA1867, ACUA139930 (holotype)	genseq-1 COI
T. rala	KX065852	KX078633	DNA2887, ACUA148555 (holotype)	genseq-1 COI, 28S
T. raptor	KX065465		DNA1257, ACUA135819 (paratype)	genseq-2 COI
T. raptor	KX065466		DNA1258, ACUA135820 (paratype)	genseq-2 COI
T. raptor	KX065467		DNA1259, ACUA135821 (paratype)	genseq-2 COI
T. raptor	KX065478		DNA1282, ACUA142966 (paratype)	genseq-2 COI
T. raptor	KX065479		DNA1283, ACUA142905 (paratype)	genseq-2 COI
T. raptor	KX065512		DNA1443, ACUA138998 (paratype)	genseq-2 COI
T. raptor	KX065562		DNA1570, ACUA138873 (paratype)	genseq-2 COI
T. raptor	KX065563		DNA1571, ACUA138871 (paratype)	genseq-2 COI
T. raptor	KX065655	KX078592	DNA1737, ACUA142906 (paratype)	genseq-2 COI, 28S
T. raptor	KX065657		DNA1742, ACUA138949 (paratype)	genseq-2 COI
T. raptor	KX065659		DNA1744, ACUA136948 (non-type voucher)	genseq-4 COI
T. raptor	KX065660		DNA1745, ACUA138957 (paratype)	genseq-2 COI
T. raptor	KX065662		DNA1747, ACUA139892 (paratype)	genseq-2 COI

T. raptor	KX065675		DNA1818, ACUA139873 (paratype)	genseq-2 COI
T. raptor	KX065916		DNA2976, ACUA148654 (paratype)	genseq-2 COI
T. raptor	KX065837		DNA2864, ACUA148539 (holotype)	genseq-1 COI
T. raptor	KX065838		DNA2865, ACUA148540 (paratype)	genseq-2 COI
T. raptor	KX065849		DNA2879, ACUA148551 (paratype)	genseq-2 COI
T. raptor	KX065850		DNA2880, ACUA148552 (paratype)	genseq-2 COI
T. raptor	KX065917		DNA2978, ACUA148658 (paratype)	genseq-2 COI
T. raptoroides	KX065856	KX078607	DNA2894, ACUA148559 (paratype)	genseq-2 COI, 28S
T. raptoroides	KX065857		DNA2895, ACUA148560 (holotype)	genseq-1 COI
T. raptoroides	KX065861		DNA2900, ACUA148564 (paratype)	genseq-2 COI
T. regalis	KX065511		DNA1442, ACUA138996 (holotype)	genseq-1 COI, 28S
T. robisoni	KX065470	KX078586	DNA1267, ACUA135825 (paratype)	genseq-2 COI, 28S
T. rockyensis	KX065507	KX078602	DNA1437, ACUA138310 (paratype)	genseq-2 COI, 28S
T. rockyensis	KX065801		DNA2623, ACUA147994 (holotype)	genseq-1 COI
T. rockyensis	KX065802		DNA2626, ACUA147995 (paratype)	genseq-2 COI
T. rockyensis	KX065803		DNA2628, ACUA147996 (paratype)	genseq-2 COI
T. rockyensis	KX065804		DNA2630, ACUA147997 (paratype)	genseq-2 COI
T. rockyensis	KX065809		DNA2644, ACUA148004 (paratype)	genseq-2 COI
T. rockyensis	KX065810		DNA2645, ACUA148005 (paratype)	genseq-2 COI
T. sellersorum	KX065774		DNA1986, ACUA138981 (paratype)	genseq-2 COI
T. sellersorum	KX065775	KX078631	DNA1987, ACUA138980 (paratype)	genseq-2 COI, 28S
T. sellersorum	KX065777		DNA1989, ACUA138983 (paratype)	genseq-2 COI
T. sellersorum	KX065816		DNA2827, ACUA148518 (paratype)	genseq-2 COI
T. sellersorum	KX065819		DNA2831, ACUA148521 (holotype)	genseq-1 COI
T. sellersorum	KX065820		DNA2835, ACUA148522 (paratype)	genseq-2 COI
T. sellersorum	KX065821		DNA2836, ACUA148523 (paratype)	genseq-2 COI
T. sellersorum	KX065822		DNA2838, ACUA148524 (paratype)	genseq-2 COI
T. sellersorum	KX065823		DNA2839, ACUA148525 (paratype)	genseq-2 COI
T. sellersorum	KX065858		DNA2897, ACUA148561 (paratype)	genseq-2 COI
T. sellersorum	KX065862		DNA2901, ACUA148565 (paratype)	genseq-2 COI
T. sellersorum	KX065863		DNA2902, ACUA148566 (paratype)	genseq-2 COI
T. sellersorum	KX065888		DNA2934, ACUA148592 (paratype)	genseq-2 COI
T. sellersorum	KX065889		DNA2935, ACUA148593 (paratype)	genseq-2 COI
T. sellersorum	KX065890		DNA2937, ACUA148594 (paratype)	genseq-2 COI
T. sharkeyi	KX065853		DNA2891, ACUA148556 (holotype)	genseq-1 COI
T. sharkeyi	KX065854		DNA2892, ACUA148557 (paratype)	genseq-2 COI
T. sharkeyi	KX065855		DNA2893, ACUA148558 (paratype)	genseq-2 COI
T. sharkeyi	KX065859		DNA2898, ACUA148562 (paratype)	genseq-2 COI, 28S
T. sharkeyi	KX065860		DNA2899, ACUA148563 (paratype)	genseq-2 COI
T. shubini	KX065694		DNA1850, ACUA139918 (paratype)	genseq-2 COI
T. shubini	KX065695		DNA1851, ACUA139919 (paratype)	genseq-2 COI
T. shubini	KX065699	KX078624	DNA1858, ACUA139922 (paratype)	genseq-2 COI, 28S
T. shubini	KX065700		DNA1860, ACUA139924 (paratype)	genseq-2 COI
T. shubini	KX065824		DNA2845, ACUA148527 (paratype)	genseq-2 COI
T. sierrensis	KX065588		DNA1606, ACUA147061 (non-type voucher)	genseq-4 COI
T. sierrensis	KX065591		DNA1609, ACUA138892 (non-type voucher)	genseq-4 COI
T. sierrensis	KX065592		DNA1610, ACUA138893 (non-type voucher)	genseq-4 COI
T. sierrensis	KX065593		DNA1611, ACUA138889 (non-type voucher)	genseq-4 COI
T. sierrensis	KX065594		DNA1612, ACUA138890 (non-type voucher)	genseq-4 COI
T. sierrensis	KX065595		DNA1613, ACUA138888 (non-type voucher)	genseq-4 COI
T. sierrensis	KX065601	KX078616	DNA1619, ACUA138924 (non-type voucher)	genseq-4 COI, 28S
T. sierrensis	KX065607		DNA1634, ACUA138930 (non-type voucher)	genseq-4 COI
T. sierrensis	KX065608		DNA1635, ACUA138926 (non-type voucher)	genseq-4 COI
T. sierrensis	KX065612		DNA1641, ACUA138941 (non-type voucher)	genseq-4 COI
T. sierrensis	KX065613		DNA1642, ACUA138934 (non-type voucher)	genseq-4 COI
T. sierrensis	KX065616		DNA1646, ACUA138938 (non-type voucher)	genseq-4 COI
T. sierrensis	KX065637		DNA1713, ACUA147063 (non-type voucher)	genseq-4 COI

T. sierrensis	KX065638		DNA1714, ACUA147062 (non-type voucher)	genseq-4 COI
T. sierrensis	KX065639		DNA1715, ACUA147060 (non-type voucher)	genseq-4 COI
T. sierrensis	KX065640		DNA1716, ACUA138901 (non-type voucher)	genseq-4 COI
T. sierrensis	KX065641		DNA1717, ACUA138902 (non-type voucher)	genseq-4 COI
T. sierrensis	KX065642		DNA1718, ACUA138903 (non-type voucher)	genseq-4 COI
T. sierrensis	KX065643		DNA1719, ACUA140805 (non-type voucher)	genseq-4 COI
T. sierrensis	KX065731		DNA1923, ACUA139976 (non-type voucher)	genseq-4 COI
T. sierrensis	KX065749		DNA1946, ACUA140724 (non-type voucher)	genseq-4 COI
T. sierrensis	KX065750		DNA1947, ACUA140726 (non-type voucher)	genseq-4 COI
T. sierrensis	KX065751		DNA1948, ACUA140721 (non-type voucher)	genseq-4 COI
T. sierrensis	KX065752		DNA1949, ACUA140728 (non-type voucher)	genseq-4 COI
T. sierrensis	KX065758		DNA1959, ACUA140727 (non-type voucher)	genseq-4 COI
T. sierrensis	KX065795		DNA2614, ACUA147988 (non-type voucher)	genseq-4 COI
T. sierrensis	KX065796		DNA2616, ACUA147989 (non-type voucher)	genseq-4 COI
T. sierrensis	KX065797		DNA2617, ACUA147990 (non-type voucher)	genseq-4 COI
T. sierrensis	KX065798		DNA2619, ACUA147991 (non-type voucher)	genseq-4 COI
T. sierrensis	KX065900		DNA2949, ACUA148604 (non-type voucher)	genseq-4 COI
T. sierrensis	KX065901		DNA2950, ACUA148605 (non-type voucher)	genseq-4 COI
T. sierrensis	KX065902		DNA2953, ACUA148606 (non-type voucher)	genseq-4 COI
T. sierrensis	KX065903		DNA2954, ACUA148607 (non-type voucher)	genseq-4 COI
T. skvarlai	KX065761		DNA1964, ACUA138982 (paratype)	genseq-2 COI
T. skvarlai	KX065764	KX078610	DNA1969, ACUA138969 (paratype)	genseq-2 COI, 28S
T. solisorta	KX065488		DNA1298, ACUA143125 (paratype)	genseq-2 COI
T. solisorta	KX065489		DNA1300, ACUA143121 (holotype)	genseq-1 COI
T. solisorta	KX065490		DNA1301, ACUA143123 (paratype)	genseq-2 COI
T. solisorta	KX065491		DNA1302, ACUA143124 (paratype)	genseq-2 COI
T. solisorta	KX065509		DNA1440, ACUA138308 (paratype)	genseq-2 COI
T. solisorta	KX065522		DNA1454, ACUA138317 (paratype)	genseq-2 COI
T. solisorta	KX065523	KX078589	DNA1455, ACUA138316 (paratype)	genseq-2 COI, 28S
T. solisorta	KX065526		DNA1458, ACUA138314 (paratype)	genseq-2 COI
T. solisorta	KX065527		DNA1459, ACUA138312 (paratype)	genseq-2 COI
T. solisorta	KX065556		DNA1563 (paratype)	genseq-2 COI
T. tahoei	KX065503		DNA1433 (non-type voucher)	genseq-4 COI
T. tahoei	KX065506		DNA1436, ACUA138311 (non-type voucher)	genseq-4 COI
T. tahoei	KX065577		DNA1594, ACUA147058 (non-type voucher)	genseq-4 COI
T. tahoei	KX065578		DNA1595, ACUA138891 (non-type voucher)	genseq-4 COI
T. tahoei	KX065579		DNA1596, ACUA138894 (non-type voucher)	genseq-4 COI
T. tahoei	KX065619		DNA1694, ACUA138911 (non-type voucher)	genseq-4 COI
T. tahoei	KX065620		DNA1695, ACUA138912 (non-type voucher)	genseq-4 COI
T. tahoei	KX065621		DNA1696, ACUA138900 (non-type voucher)	genseq-4 COI
T. tahoei	KX065622		DNA1697, ACUA138907 (non-type voucher)	genseq-4 COI
T. tahoei	KX065623		DNA1698, ACUA138908 (non-type voucher)	genseq-4 COI
T. tahoei	KX065624		DNA1699, ACUA147057 (non-type voucher)	genseq-4 COI
T. tahoei	KX065625		DNA1700, ACUA140758 (non-type voucher)	genseq-4 COI
T. tahoei	KX065739		DNA1932, ACUA139984 (non-type voucher)	genseq-4 COI
T. tahoei	KX065740		DNA1933, ACUA139985 (non-type voucher)	genseq-4 COI
T. tahoei	KX065741	KX078598	DNA1934, ACUA139986 (non-type voucher)	genseq-4 COI, 28S
T. tahoei	KX065754		DNA1951, ACUA140725 (non-type voucher)	genseq-4 COI
T. tahoei	KX065756		DNA1957, ACUA140736 (non-type voucher)	genseq-4 COI
T. tahoei	KX065811		DNA2647, ACUA148006 (non-type voucher)	genseq-4 COI
T. tahoei	KX065812		DNA2650, ACUA148007 (non-type voucher)	genseq-4 COI
T. tahoei	KX065813		DNA2651, ACUA148008 (non-type voucher)	genseq-4 COI
T. tahoei	KX065814		DNA2652, ACUA148009 (non-type voucher)	genseq-4 COI
T. tahoei	KX065815		DNA2653, ACUA148010 (non-type voucher)	genseq-4 COI
T. tahoei	KX065897		DNA2946, ACUA148601 (non-type voucher)	genseq-4 COI
T. tahoei	KX065898		DNA2947, ACUA148602(non-type voucher)	genseq-4 COI
T. tahoei	KX065899		DNA2948, ACUA148603 (non-type voucher)	genseq-4 COI

T. tricolor	KX065650		DNA1732, ACUA138963 (non-type voucher)	genseq-4 COI
T. tricolor	KX065651	KX078632	DNA1733, ACUA138962 (non-type voucher)	genseq-4 COI, 28S
T. tricolor	KX065654		DNA1736, ACUA138961 (non-type voucher)	genseq-4 COI
T. tricolor	KX065673		DNA1815, ACUA139903 (non-type voucher)	genseq-4 COI
T. tricolor	KX065682		DNA1831, ACUA139889 (non-type voucher)	genseq-4 COI
T. tricolor	KX065683		DNA1832, ACUA139890 (non-type voucher)	genseq-4 COI
T. tricolor	KX065684		DNA1835, ACUA139888 (non-type voucher)	genseq-4 COI
T. tricolor	KX065696		DNA1854, ACUA138991 (non-type voucher)	genseq-4 COI
T. tricolor	KX065697		DNA1855, ACUA138958 (non-type voucher)	genseq-4 COI
T. trimaculata	KX065504		DNA1434, ACUA138999 (paratype)	genseq-2 COI
T. trimaculata	KX065505		DNA1435, ACUA138338 (paratype)	genseq-2 COI
T. trimaculata	KX065508		DNA1439 (paratype)	genseq-2 COI
T. trimaculata	KX065516		DNA1448, ACUA138325 (paratype)	genseq-2 COI
T. trimaculata	KX065517		DNA1449, ACUA138324 (paratype)	genseq-2 COI
T. trimaculata	KX065518		DNA1450, ACUA138329 (holotype)	genseq-1 COI
T. trimaculata	KX065519		DNA1451, ACUA138330 (paratype)	genseq-2 COI
T. trimaculata	KX065520		DNA1452, ACUA138323 (paratype)	genseq-2 COI
T. trimaculata	KX065521		DNA1453, ACUA138326 (paratype)	genseq-2 COI
T. trimaculata	KX065524		DNA1456, ACUA138331 (paratype)	genseq-2 COI
T. trimaculata	KX065525		DNA1457, ACUA138322 (paratype)	genseq-2 COI
T. trimaculata	KX065529	KX078601	DNA1531, ACUA138334 (paratype)	genseq-2 COI, 28S
T. trimaculata	KX065530		DNA1532, ACUA138328 (paratype)	genseq-2 COI
T. trimaculata	KX065531		DNA1533, ACUA138333 (paratype)	genseq-2 COI
T. trimaculata	KX065532		DNA1534, ACUA138337 (paratype)	genseq-2 COI
T. trimaculata	KX065533		DNA1535, ACUA138327 (paratype)	genseq-2 COI
T. trimaculata	KX065534		DNA1536 (paratype)	genseq-2 COI
T. trimaculata	KX065535		DNA1537, ACUA138332 (paratype)	genseq-2 COI
T. trimaculata	KX065536		DNA1538 (paratype)	genseq-2 COI
T. trimaculata	KX065537		DNA1539, ACUA138335 (paratype)	genseq-2 COI
T. trimaculata	KX065538		DNA1540, ACUA138339 (paratype)	genseq-2 COI
T. trimaculata	KX065539		DNA1541, ACUA148714 (paratype)	genseq-2 COI
T. trimaculata	KX065540		DNA1542, ACUA148711 (paratype)	genseq-2 COI
T. trimaculata	KX065541		DNA1543, ACUA148710 (paratype)	genseq-2 COI
T. trimaculata	KX065542		DNA1544, ACUA148709 (paratype)	genseq-2 COI
T. trimaculata	KX065543		DNA1545, ACUA148712 (paratype)	genseq-2 COI
T. trimaculata	KX065768		DNA1980, ACUA138990 (paratype)	genseq-2 COI
T. tysoni	KX065480		DNA1287, ACUA135811 (paratype)	genseq-2 COI
T. tysoni	KX065707	KX078625	DNA1869, ACUA139932 (paratype)	genseq-2 COI, 28S
T. tysoni	KX065778		DNA2117, ACUA147046 (paratype)	genseq-2 COI
T. tysoni	KX065839		DNA2866, ACUA148541 (paratype)	genseq-2 COI
T. tysoni	KX065840		DNA2867, ACUA148542 (paratype)	genseq-2 COI
T. tysoni	KX065841		DNA2868, ACUA148543 (paratype)	genseq-2 COI
T. tysoni	KX065842		DNA2869, ACUA148544 (paratype)	genseq-2 COI
T. tysoni	KX065843		DNA2870, ACUA148545 (paratype)	genseq-2 COI
T. tysoni	KX065844		DNA2871, ACUA148546 (holotype)	genseq-1 COI
T. tysoni	KX065845		DNA2872, ACUA148547 (paratype)	genseq-2 COI
T. ululata	KX065565		DNA1579, ACUA138885 (paratype)	genseq-2 COI
T. ululata	KX065570	KX078614	DNA1584, ACUA138884 (paratype)	genseq-2 COI, 28S
T. unimaculata	KX065566		DNA1580, ACUA138880 (paratype)	genseq-2 COI
T. unimaculata	KX065567		DNA1581, ACUA138881 (paratype)	genseq-2 COI
T. unimaculata	KX065568	KX078608	DNA1582, ACUA140741 (paratype)	genseq-2 COI, 28S
T. unimaculata	KX065569		DNA1583, ACUA138875 (paratype)	genseq-2 COI
T. unimaculata	KX065571		DNA1585, ACUA138874 (paratype)	genseq-2 COI
T. unimaculata	KX065929		DNA3008, ACUA148704 (paratype)	genseq-2 COI
T. unimaculata	KX065930		DNA3010, ACUA148705 (holotype)	genseq-1 COI
T. unimaculata	KX065931		DNA3011, ACUA148706 (paratype)	genseq-2 COI
T. ventura	KX065606	KX078609	DNA1632, ACUA138929 (non-type voucher)	genseq-4 COI, 28S

T. ventura	KX065744		DNA1939, ACUA139990 (non-type voucher)	genseq-4 COI
T. ventura	KX065745		DNA1940, ACUA140714 (non-type voucher)	genseq-4 COI
T. ventura	KX065747		DNA1942, ACUA140716 (non-type voucher)	genseq-4 COI
T. ventura	KX065748		DNA1943, ACUA140719 (non-type voucher)	genseq-4 COI
T. walteri	KX065598		DNA1616, ACUA138897 (paratype)	genseq-2 COI
T. walteri	KX065599		DNA1617, ACUA138917 (paratype)	genseq-2 COI
T. walteri	KX065600		DNA1618, ACUA138918 (paratype)	genseq-2 COI
T. walteri	KX065611		DNA1640, ACUA138943 (paratype)	genseq-2 COI
T. walteri	KX065617	KX078617	DNA1648, ACUA138939 (paratype)	genseq-2 COI, 28S
T. walteri	KX065618		DNA1649, ACUA138940 (paratype)	genseq-2 COI
T. walteri	KX065646		DNA1722, ACUA140804 (paratype)	genseq-2 COI
T. walteri	KX065724		DNA1911, ACUA139951 (paratype)	genseq-2 COI
T. walteri	KX065726		DNA1915, ACUA139966 (paratype)	genseq-2 COI
T. walteri	KX065727		DNA1916, ACUA139967 (paratype)	genseq-2 COI
T. walteri	KX065728		DNA1917, ACUA139968 (paratype)	genseq-2 COI
T. walteri	KX065729		DNA1920, ACUA139971 (paratype)	genseq-2 COI
T. walteri	KX065759		DNA1960, ACUA140737 (paratype)	genseq-2 COI
T. walteri	KX065904		DNA2955, ACUA148608 (holotype)	genseq-1 COI
T. welbourni	KX065609		DNA1638, ACUA138927 (holotype)	genseq-1 COI, 28S
